# The impact of ER^UPR^ on mitochondrial integrity mediated by PDK4

**DOI:** 10.1038/s41419-025-07743-5

**Published:** 2025-07-29

**Authors:** Priyanka Mallick, Sebabrata Maity, Rupsha Mondal, Trina Roy, Puyam Milan Meitei, Shashank Saxena, Bhavani Shankar Sahu, Oishee Chakrabarti, Saikat Chakrabarti

**Affiliations:** 1 Structural Biology and Bioinformatics Division, CSIR-Indian Institute of Chemical Biology, IICB TRUE Campus, CN-6, Sector 5, Kolkata, 700091 India; 2 Academy of Scientific and Innovative Research (AcSIR), Ghaziabad, 201002 India; 3 Biophysics and Structural Genomics Division, Saha Institute of Nuclear Physics, Saha Institute of Nuclear Physics, Kolkata, 700064 India; 4 Homi Bhabha National Institute, Mumbai, India; 5 Cell Biology and Physiology Division, CSIR-Indian Institute of Chemical Biology, IICB TRUE Campus, CN-6, Sector 5, Kolkata, 700091 India; 6 National Brain Research Centre, Manesar, 122051 India

**Keywords:** Mechanisms of disease, Molecular neuroscience, Cellular neuroscience

## Abstract

ER and mitochondrial stress are often interconnected and considered major contributors to aging as well as neurodegeneration. Coordinated induction of ER^UPR^ and mito^UPR^ has been observed in diabetes and pulmonary disorders. However, in the context of aging and neurodegeneration, regulation of this intra-organellar crosstalk has remained relatively elusive. Here, we demonstrate that pyruvate dehydrogenase kinase 4 (PDK4), a mitochondrial protein, accumulates at the ER-mitochondrial contact sites (MAMs) during ER stress. Classically, PDK4 is known to phosphorylate PDHA1 (pyruvate dehydrogenase E1 subunit alpha 1) and plays a significant role in regulating the oxidative phosphorylation-driven ATP production. In this study, we propose a non-canonical kinase-independent function of PDK4; we show that it acts as a connecting link between ER^UPR^ and mito^UPR^, with significance in aging and Alzheimer’s disease (AD) associated neurodegeneration. Transcriptomics analyses show increased PDK4 levels upon drug-induced ER stress. We detect elevated PDK4 levels in lysates from human AD patient and mouse models as well as in ex vivo AD models. Additionally, exogenous expression of PDK4 was found to refine ER-mitochondria communication, significantly altering mitochondrial morphology and function. Further, we also observe defective autophagic clearance of mitochondria under such conditions. It is prudent to suggest that elevated PDK4 levels could be one of the key factors connecting ER^UPR^ with mito^UPR^, a phenotypic contributor in aging and in AD-like neurodegenerative disorders.

## Introduction

Endoplasmic reticulum (ER) plays a central role in the folding process of membrane and secretory proteins; proteins that fail to attain their proper conformation due to misfolding, unfolding or erroneous modifications get cleared off from the cell. However, under certain physiological stresses, like nutrient deprivation, import-export or storage imbalance of Ca^2+^, aging or pathological insults, like bacterial or viral infections, neurodegeneration, to name a few, the folding capacity at the ER gets compromised. This leads to the accumulation of misfolded proteins within the ER lumen, termed as ER stress, and elicits a cellular response known as the unfolded protein response (UPR) [1]. Recent studies suggest that ER stress in various cellular contexts can alter mitochondrial morphology and bioenergetics through transcription-dependent and independent pathways [2–5].

Mitochondria are indispensable cellular organelles that regulate ATP generation, Ca^2+^ signaling [6, 7], apoptosis [6, 8], reactive oxygen species (ROS) production and neutralization [9], neurotransmitter metabolism [10], etc. Thus, maintaining mitochondrial homeostasis is extremely crucial for cellular homeostasis and viability. In neurons, spatio-temporal regulation of the mitochondrial functions is attained with mitochondrial localization [11, 12] and biogenesis [13], processes that are largely dependent on the fission-fusion competence of these organelles and their transport efficiency. Hence, perturbations in mitochondrial dynamics, bioenergetics and altered activity of their fission-fusion machineries are observed in most neurodegenerative diseases and neuropathies [5, 14–16]. However, the contribution of altered mitochondrial dynamics in disease etiology is still far from being fully understood. While significant efforts have been put in studying the contributions of the canonical ER^UPR^ pathways (ATF6, IRE1α and PERK) in the context of ER stress-mediated neuronal disease progression, identification and functional characterization of components from the other organelles, especially the mitochondria, remains to be studied.

ER forms membrane contact sites (MCS) with different organelles and plasma membrane. Among such contact sites, ER-mitochondria tethering points or MAM (mitochondria-associated endoplasmic reticulum membrane) junctions are vital in regulating Ca^2+^, metabolites, and lipid transfer between the two organelles [17]. Thereby, structural integrity and lipid composition of mitochondrial membrane are largely dependent upon imported lipids or precursor molecules from ER [18]. Further, previous studies have shown that autophagosomes could originate at these ER-mitochondria contact sites under starvation [19]. These junctions are also suggested to be critical in regulating reticulo-mito-phagy [20]. Atypical lipid trafficking between ER and mitochondria, as well as defects in autophagy, are implicated in ageing and neurodegenerative diseases, supporting the hypothesis that ER^UPR^ can directly affect mito^UPR^, a stress response pathway that provides mitochondrial quality control. Increasing evidences show that the number, duration, and dynamics of molecular composition of MAM junctions are vital to mitigate cellular stress and neurodegenerative disease pathology [21–23]. However, a detailed understanding of the molecular players in this is warranted.

The pyruvate dehydrogenase complex (PDHc) provides the link between glycolysis and the TCA cycle by catalyzing the end product of glycolysis, pyruvate into acetyl-CoA [24]. The activity of the PDHc is negatively regulated by its reaction products, acetyl-CoA and NADH, and pyruvate dehydrogenase kinase (PDK) dependent serine phosphorylation on the α-subunit (E1) [24–26]. Pyruvate dehydrogenase phosphatase (PDP) can dephosphorylate and re-activate PDHc [27]. PDK family of proteins comprises 4 members, PDK1 through 4; all of these have tissue-specific expression and activity [28]. PDKs play significant roles in maintaining the metabolic status of cells. Apart from their function in cellular metabolism, one of the PDKs, pyruvate dehydrogenase kinase 4 (PDK4), has been reported to localize at the ER-mito contact sites of muscle cells, regulating the insulin signaling during obesity [29], suggesting an unexplored role for this mitochondrial protein in cellular physiology. Here, we show that eliciting ER^UPR^ can affect PDK4 levels, interestingly at the ER-mitochondria contact sites. Such an alteration in PDK4 protein is physiologically relevant as its increased levels are detected in serum-starved cells. Hippocampi of 1-year-old rat brains as well as brain lysates from Alzheimer’s disease (AD) mouse models and AD human patient corroborate these results. While ER stress elicits higher PDK4 levels in cellular systems, the reciprocal is also true – increasing PDK4 protein levels, upregulates well-established ER stress markers, along with those of mito^UPR^. Increased expression of ER^UPR^ and mito^UPR^-associated players in the presence of high PDK4 levels occurs irrespective of its kinase activity. Elevated PDK4 levels at MAM junctions eventuate to mitochondrial fragmentation and compromised metabolism, enhanced commencement of autophagy but impaired clearance of autophagosomes. Our results unravel non-canonical activity of PDK4 in maintaining mitochondrial structure, function, and quality control; all factors in turn responsible for overall cellular homeostasis and dysregulation of which are often linked with aging and neurodegeneration.

## Results

### Effect of ER stress on PDK4

**Fig. 1:**
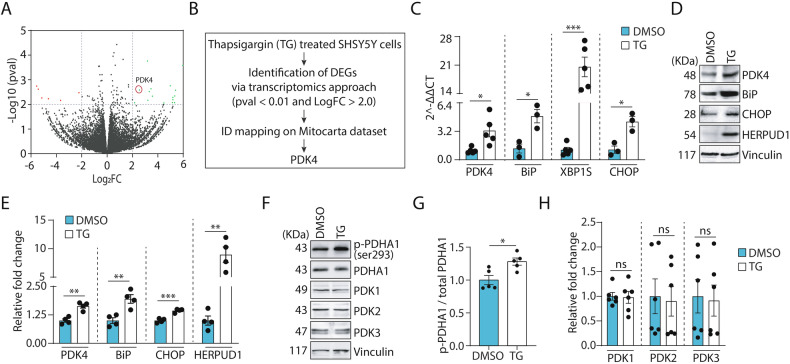
ER stress induces PDK4 expression. **A** SH-SY5Y cells were treated with TG (Thapsigargin, 0.5 µM for 3 h) or DMSO, and were subjected to RNA transcriptomics analysis. Green and red data points on the volcano plot represent the up- and downregulated genes (LogFC ± 2.0 and *p*-value ≤ 0.01 cutoffs marked in gray lines), obtained from three biological replicates of DMSO and TG-treated samples, respectively. **B** Flowchart for identification of PDK4 from the deregulated genes of transcriptomics data. **C** qRT-PCR analysis shows enhanced PDK4, BiP, CHOP, and XBP1-S mRNA level in TG-treated condition, as compared to the control. Data represent the mean ± SEM of ≥3 independent experiments. **D** Immunoblotting of cell lysates was carried out to analyze levels of indicated proteins in TG-induced ER stress. **E** Altered protein levels analyzed in (**D**), mean ± SEM of 4 independent experiments. **F** Cell lysates from DMSO and TG-treated samples were immunoblotted to detect expression and phosphorylation status of indicated proteins. **G**, **H** Graphs represent changes in expression of different proteins, as mentioned in (**F**). p-PDHA1 was normalized to total PDHA1 level. Data represent the mean ± SEM of ≥5 independent experiments. *p ≤ 0.05; **p ≤ 0.01; ***p ≤ 0.001; ns: not significant (estimated via unpaired two-tailed Student’s *t*-test).

To induce ER stress, human neuroblastoma SH-SY5Y cells were treated for 3 h with 0.5 µM thapsigargin (TG), a known chemical inhibitor of sarco-endoplasmic reticulum Ca^2+^ ATPase (SERCA) pumps. mRNA transcriptomes of DMSO (control) and TG (drug) treated cells were compared to identify the deregulated genes (log_2_FC ± 2.0 and *p*-value ≤ 0.01) affected by ER stress (Fig. 1A). These genes were further mapped onto the MitoCarta database version 3.0 [30] to identify mitochondrial genes that were altered during ER stress, using the human specific dataset. Our analyses showed significant upregulation in the mRNA level of the mitochondrial Pyruvate dehydrogenase kinase 4 (PDK4) under ER stress induced by TG treatment (Fig. 1B). This upregulation of PDK4 was further confirmed by RT-PCR and Western blot analyses (Fig. 1C–E). In addition, mRNA and protein levels of the canonical ER stress markers like BiP, CHOP, XBP1S, and HERPUD1 were also elevated under similar conditions (Fig. 1C–E). Increased protein levels of PDK4 were also detected upon treatment with 5 μg/ml tunicamycin (Tun) for 3 h, an antibiotic that induces ER stress by abrogating N-linked glycosylation of proteins (Supplementary Fig. 1A, B), suggesting a correlation between chemically induced ER stress and PDK4. Together, these results indicate that chemically induced ER stress can alter the expression of an important mitochondrial kinase, PDK4.

Additionally, a significant increase in phosphorylation of PDK4 substrate PDHA1, at Ser293, was detected in TG-treated cells; however, the total PDHA1 level remains marginally less in the latter (Fig. 1F, G). Increased phosphorylation of PDHA1 is linked to the deactivation of PDHc; this observation raises the possibility that under ER stress a probable role of PDK4 in mediating ER stress response that is independent of its usual enzymatic function. To verify if other isoforms of PDK were similarly affected during ER stress, levels of PDK1, PDK2 and PDK3 were compared during ER stress (Fig. 1F, H). We could not detect any significant change in the protein levels of any of the isoforms, other than PDK4, though all of them shared extensive sequence similarity (Supplementary Fig. 1C).

### Altered PDK4 in physiological models of ER stress

**Fig. 2:**
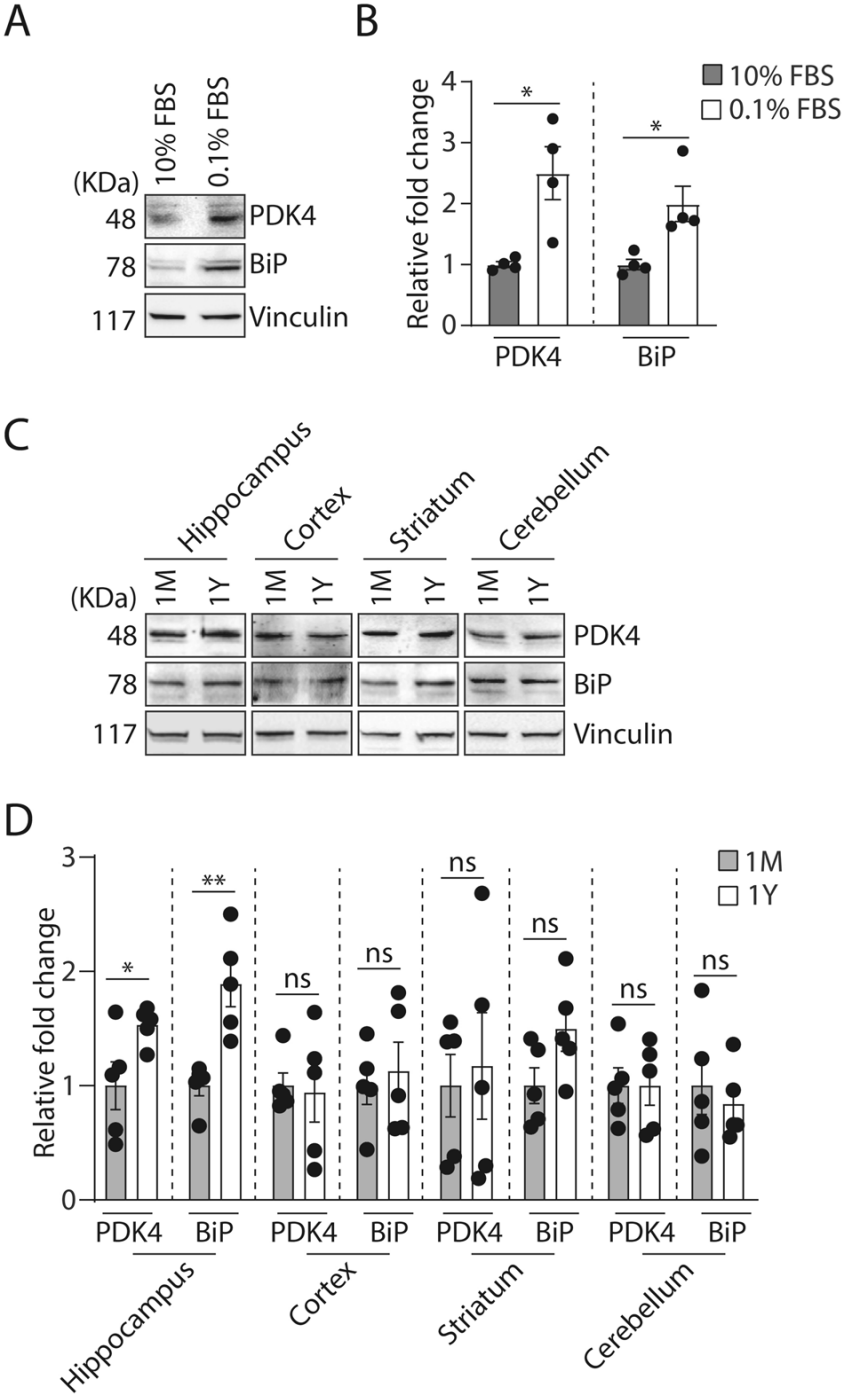
Serum starvation and aging-mediated physiological stress elevate PDK4 level. **A** SH-SY5Y cells were treated with high (10%) and low (0.1%) levels of FBS concentrations and immunoblotting of cell lysates were performed to detect PDK4 and BiP protein levels. **B** Graphs show the alterations in PDK4 and BiP proteins. Data represent the mean ± SEM of 4 independent experiments. **C** Immunoblotting of brain region-specific lysates (hippocampus, cortex, striatum, and cerebellum) of 1-month and 1-year-old rats was performed to check the expression of indicated proteins. **D** Graphs represent the altered protein level and represented as the mean ± SEM of 5 animals/group. *p ≤ 0.05; **p ≤ 0.01; ns not significant (estimated via unpaired two-tailed Student’s *t*-test).

Multiple reports suggest a close link between ER stress and a plethora of neuro-pathophysiological conditions [31–34]. Hence, we hypothesized a correlation between PDK4 and various pathophysiological stresses. To establish this, cells were subjected to serum withdrawal and analyzed for the protein levels of PDK4. Our results showed significant upregulation in PDK4 and BiP upon serum starvation (Fig. 2A, B). It is well-established that cellular proteostasis gets progressively compromised during the process of aging, which is also a known contributing factor in age-related pathologies, like neurodegeneration. Aging as well as neurodegenerative diseases are further associated with ER stress [35–37]. To explore if PDK4 was affected during aging, its protein levels were verified in different brain regions of young (1 month) and aged (1 year) rats (Fig. 2C, D). We detected significantly elevated PDK4 levels in aged hippocampus, though not in other brain regions (cortex, striatum, and cerebellum). This increase positively correlated with BiP levels in the corresponding brain region. Altered neurogenesis of hippocampus is prominent in several disease scenarios including Alzheimer’s disease (AD) [38]. Additionally, impaired UPR of hippocampus is suggested in aged rats [39]. Hence, region-specific elevation of PDK4 levels in aged rat brain is indicative of its probable involvement in ER stress-mediated hippocampal damage.

### Increase in PDK4 levels in pathological models of ER stress

**Fig. 3:**
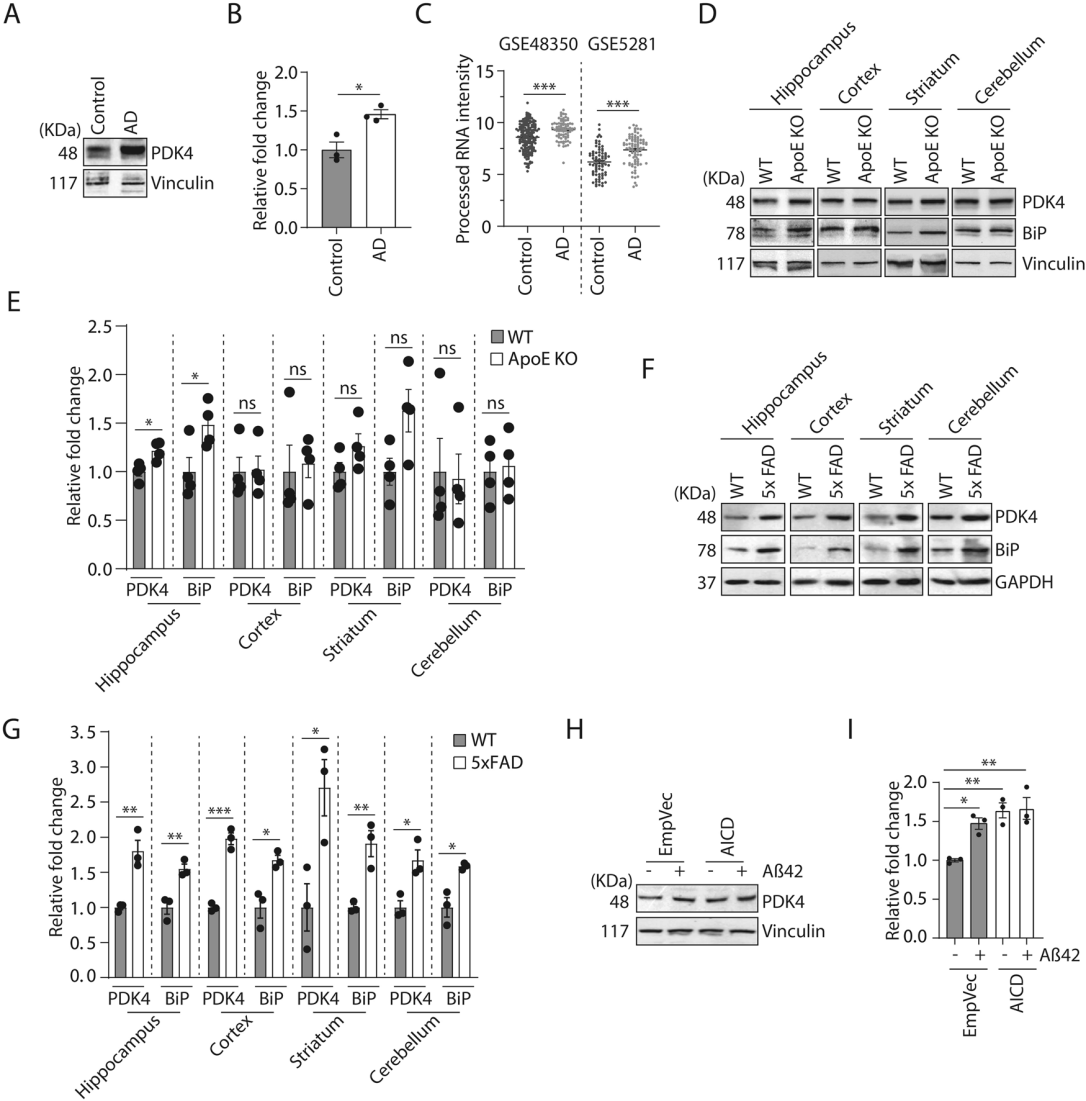
Increased PDK4 expression in Alzheimer’s Disease (AD) patients and animal models. **A** Whole-brain protein lysates of control and AD patient were immunoblotted to check expression of PDK4. **B** Histogram plotted with data from (**A**), mean ± SEM of 3 technical replicates. **C** Graph represents processed mRNA intensity of PDK4 in AD-specific GSE datasets (GSE48350, GSE5281) with processed data retrieved from BrainProt server. **D** Immunoblotting of brain region-specific lysates (hippocampus, cortex, striatum, and cerebellum) of wild type (WT) and ApoE knock out (KO) mice was performed to check the expression of indicated proteins. **E** Graph plotted with data from (**D**), mean ± SEM of 4 animals per group. **F** Similar experiment as (**D**) using region-specific brain lysates from 5xFAD mice. **G** Histogram plotted with data from (**F**), mean ± SEM of at least 3 animals per group. **H** SH-SY5Y cells transfected with control or AICD constructs, used with either DMSO or Aβ treatment for 48 h. Aβ treatment was done either 1 μM or 0.5 μM along with AICD. Cell lysates were immunoblotted to check PDK4 protein level. **I** Graph shows data as the mean ± SEM of 3 independent experiments. *p ≤ 0.05; **p ≤ 0.01; ***p ≤ *0.001;* ns not significant (estimated via unpaired two-tailed Student’s *t*-test, one-way ANOVA with Tukey’s corrections).

We next aimed to investigate the levels of PDK4 in pathologically relevant models. For this, whole-brain lysates of AD patient and control individual were compared; PDK4 protein level was seen to be significantly upregulated in AD brain lysate (Fig. 3A, B), but remained unaltered in PD patient sample as compared to the control (Supplementary Fig. 2A, B). Due to the unavailability of more diseased brain samples for validation, we looked at the already available AD brain-specific human data. We retrieved processed RNA intensity of PDK4 from two such GSE datasets (GSE48350, GSE5281) from BrainProt [40]. It showed significant upregulation of PDK4 in AD brains at the RNA level (Fig. 3C). Next, we validated region-specific alterations of PDK4 levels in the AD-specific animal model of Apolipoprotein E (ApoE) KO mice. Here again, we detected significantly elevated PDK4 protein levels in the hippocampal region of ApoE KO mice (Fig. 3D, E). The other brain regions did not reflect a similar change in PDK4 levels, recapitulating our observations in aging rat samples (Fig. 2C, D). Additionally, PDK4 level was checked in brain lysates from 5xFAD mice; another well-established animal model of AD, which shows robust and severe amyloid pathology [41]. It was observed that PDK4, along with the ER stress marker BiP was elevated in all four brain regions of the 5xFAD animals (Fig. 3F, G). It further strengthens the probable link between PDK4 and AD disease progression. PDK4 levels being affected in all brain regions of 5xFAD mice probably reflect the robustness of this AD model. A significant increase in PDK4 levels was observed in cell-based model of AD when compared with the control (Fig. 3H, I). For this, SH-SY5Y cells were transiently transfected with AICD (amyloid precursor protein intracellular domain) or control vector, with or without treatment with amyloid-β 42 (Aβ 42) fragment [16, 42].

Next, the status of PDK4 level was analyzed in an already characterized prion disease model of neurodegeneration [43, 44]. It has been reported that point-mutations like A117V and KHII (K110I, H111I) can enrich the population of a particular transmembrane form of prion protein (^Ctm^PrP) in cells; a form of PrP associated with sporadic prion diseases. Our results showed that PDK4 was upregulated in the presence of the natural Gerstmann-Sträussler-Scheinker syndrome (GSS) variant of PrP (A117V) that is known to generate ^Ctm^PrP in abundance and ER stress (Supplementary Fig. 2C, D). Thus, we detect differentially upregulated PDK4 in multiple neurodegeneration disease models. Moreover, the connection of PDK4 to PD progression cannot also be completely ruled out as sample number was limited in our study.

Together, these results suggest a plausible role for this mitochondrial protein in the context of ER stress; one of the probable roles could be mediated at the ER-mitochondria junctions. These membrane contact sites are known to be altered in multiple neurodegenerative conditions [21–23].

### PDK4 localizes at MAM junctions during ER stress

**Fig. 4:**
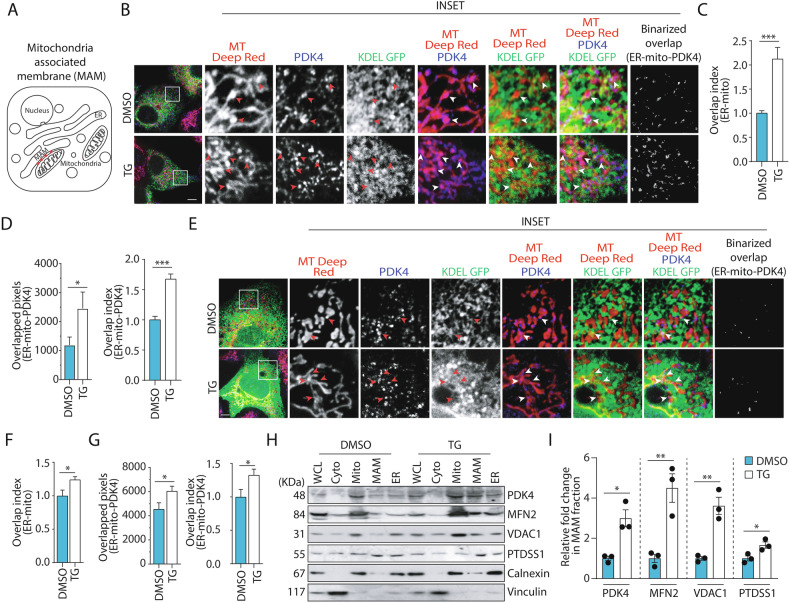
PDK4 localizes at MAM junction under ER stress. **A** Schematic representation of MAM (in red). **B** SH-SY5Y cells transfected with KDEL-GFP, treated with DMSO or TG (0.5 µM for 3 h), followed by staining with MitoTracker Deep Red FM. Cells were fixed and immuno-stained to detect PDK4. Enlarged views of the areas within the white boxes are shown (inset). Overlapped regions are marked with red arrowheads for black and white insets, and white arrowheads for colored insets. Scale bar: 5 µm. **C** Graph with data from (**B**) shows quantification of KDEL-GFP and MitoTracker Deep Red FM overlap; co-localization measured from the binarized images represented as relative fold change (overlap index) ± SEM. Data was calculated from ∼80 cells from 3 independent experiments. **D** Graph represents raw pixel count of overlapped KDEL-GFP and MitoTracker Deep Red FM and PDK4 signals (left). Graph (right) represents co-localization measured from the binarized images as relative fold change (overlap index) ± SEM. Data was calculated from ∼80 cells from 3 independent experiments. **E** U2OS cells transfected with KDEL-GFP, treated with DMSO or TG (0.5 µM for 3 h), followed by staining with MitoTracker Deep Red FM. Cells were fixed and immuno-stained to detect PDK4. Enlarged views of the areas within the white boxes are shown (inset). Overlapped regions are marked with red arrowheads for black and white insets, and white arrowheads for colored insets. Scale bar: 5 µm. **F** Graph with data from (**E**) represents KDEL-GFP and MitoTracker Deep Red FM overlap; co-localization measured from the binarized images represented as relative fold change (overlap index) ± SEM. Data was calculated from ∼20 cells from 3 independent experiments. **G** Graphs with data from (**E**) subjected to similar analyses and quantification as (**D**). Data was calculated from ∼20 cells from 3 independent experiments. **H** Different cellular fractions obtained from DMSO and TG (0.5 µM for 3 h) treated SH-SY5Y cells (as described in “Materials and methods”) were subjected to immunoblotting to check the expression of indicated proteins. MFN2, VDAC1 and PTDSS1 were used as MAM markers; whereas calnexin and vinculin were used as ER and cytosolic markers, respectively. MFN2 and VDAC1 served as mitochondrial markers, as well. WCL whole cell lysate, Cyto cytosol, Mito mitochondria. **I** Graphs represent the indicated protein levels (**H**) and represented as the mean ± SEM of 3 independent experiments. *p ≤ 0.05; **p ≤ 0.01; ***p ≤ 0.001 (estimated via unpaired two-tailed Student’s *t*-test).

An increase in ER-mitochondria junction (MAM) has been previously reported in AD [45]. In our study, altered presence of PDK4 at the MAM junctions was validated in multiple ways. First, super-resolution imaging indicated higher number of ER-mitochondria contacts in SH-SY5Y and U2OS cells under ER stress when compared with the control; a simultaneous increase in the localization of PDK4 at MAM junctions was also detected in these stressed cells (Fig. 4A–G). Like the SH-SY5Y cells, U2OS cells were also responsive to TG mediated ER stress; as we detected higher levels of BiP, along with increased PDK4 in them (Supplementary Fig. 3A). While no significant change was seen with the matrix protein, HSP60 between cells under ER stress conditions and controls, MAM localized outer mitochondrial protein, MFN2 showed enrichment at the ER-mitochondria contact sites (Supplementary Fig. 3B–E). Second, sub-cellular fractionation of cell lysates (Supplementary Fig. 3F) showed significantly higher levels of PDK4 protein in MAM-enriched fractions in TG-treated samples compared to the controls (Fig. 4H, I). Along with PDK4, other mitochondrial proteins known to be enriched in MAM fractions (MFN2, VDAC1 and PTDSS1) were also detected at higher levels in TG-treated samples than in the controls. Hence, both the imaging and fractionation-based protein estimation results showed an increase in MAM junctions and accompanying increase in PDK4 at these contact sites in cells subjected to ER stress. Presence of higher numbers of ER-mitochondria contacts is known to suggest increased mitochondrial fission [46] as was observed in cells treated with TG (Supplementary Fig. 3G, H).

### Elevated PDK4 level elicits ER stress-like response

Next, we investigated if enhanced PDK4 level could affect expression of the other PDK isoforms (Supplementary Fig. 4A, B). Under TG-induced ER stress condition, no other PDK isoforms except PDK4 were found to be enhanced (Fig. 1F, H). However, when PDK4 was exogenously expressed, a corresponding decrease in PDK2 levels could be detected. This suggested a probable antagonistic effect between the two isoforms. Hence, we checked for the presence of PDK2 in MAM fractions (Supplementary Fig. 4C). PDK2, however, could not be detected in MAM-enriched fractions across samples. This clearly rules out a direct effect of PDK2 on ER-mitochondria contact sites. We cannot, however, nullify the possibility of an indirect regulatory mechanism that involves PDK2 along with some other molecular players; this is beyond the scope of the present study.

**Fig. 5:**
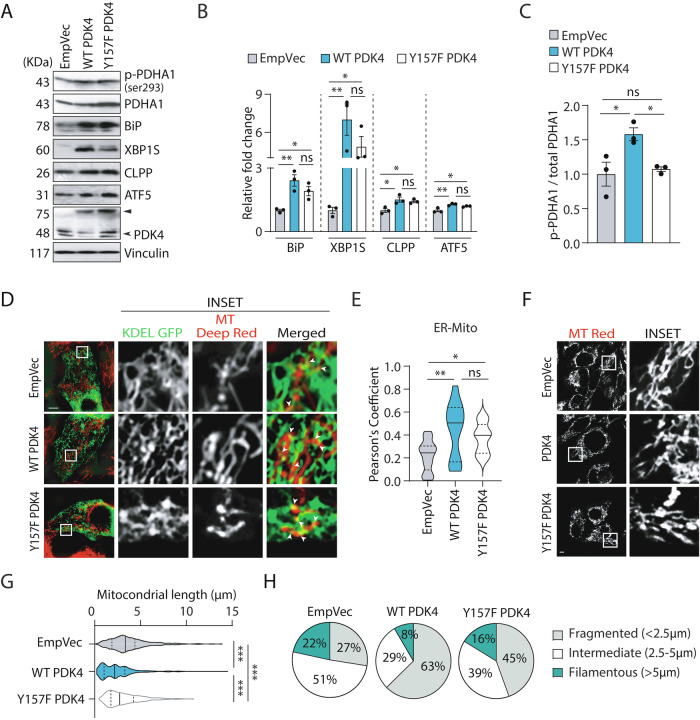
PDK4 overexpression affects mitochondrial morphology and homeostasis. **A** SH-SY5Y cell lysates from empty vector (EmpVec), WT PDK4 or Y157F PDK4-transfected samples were immunoblotted to detect the expression and phosphorylation status of indicated proteins. ► Exogenous (PDK4-RFP), ➤ endogenous. **B**, **C** Graphs represent change in expression of different proteins, as mentioned in (**A**). p-PDHA1 was normalized to total PDHA1 level. Data represent the mean ± SEM of 3 independent experiments. **D** Cells transfected with KDEL-GFP, along with PDK4 constructs or control (EmpVec), imaged under live-cell conditions using MitoTracker Deep Red FM. Enlarged views of the areas within the white boxes are shown (inset). Scale bar: 10 µm. **E** Violin plot shows Pearson’s correlation coefficient for co-localization of KDEL-GFP (ER) and MitoTracker Deep Red FM (mitochondria) in indicated samples (as described in **D**). Data shown are of ∼60 cells from 3 independent experiments. Solid and dashed black lines mark median and quartiles, respectively. **F** Cells transfected with indicated constructs were imaged under live-cell conditions using MitoTracker Red FM. Enlarged views of the areas within the white boxes are shown (inset). Scale bar: 5 µm. **G** Violin plot of the mitochondrial length (μm) in images as described in (**F**). Solid and dashed black lines mark median and quartiles, respectively. **H** Pie-charts show categorical representation of total mitochondrial pool depending on their lengths (<2.5 μm fragmented, 2.5–5 μm intermediate and >5 μm filamentous) in images shown in (**F**). *p ≤ 0.05; **p ≤ 0.01; ***p ≤ 0.001; ns not significant (estimated via unpaired two-tailed Student’s *t*-test, one-way ANOVA with Tukey’s corrections).

We wanted to verify if increased PDK4 levels alone via exogenous expression could evoke an ER stress-like response and also affect mitochondria. To address this, lysates from SH-SY5Y cells transiently transfected with either PDK4 wild type (WT) or its mutant (Y157F PDK4) were analyzed for known ER^UPR^ marker proteins (Fig. 5A, B). Phosphorylated PDHA1 was enhanced in the WT PDK4-transfected cells as compared to the control (Fig. 5A, C). As reported earlier [47], a significant decrease in PDHA1 phosphorylation in the presence of the Y157F PDK4 mutant clearly suggested its compromised kinase activity. We further observed significantly elevated levels of BiP, XBP1S, CLPP and ATF5 upon exogenous expression of either WT PDK4 or its mutant, while PDK4 levels remained similar in these samples. CLPP and ATF5 are known for their significance in mito^UPR^ [48]. Interestingly, we observed increased ER-mitochondria overlap in both WT and mutant PDK4 overexpressed cells, as compared to the control (Fig. 5D, E), similar to the TG-induced condition (Fig. 4B, E). Increase in mitochondrial fragmentation was detected in cells with exogenous WT PDK4 or PDK4 Y157F (Fig. 5F–H), confirming changes in mitochondrial morphology upon PDK4 overexpression [49], irrespective of its kinase activity. These observations suggest a direct correlation between PDK4, altered mitochondrial morphology and ER stress.

Since the Y157F point mutant did not completely abrogate kinase activity, we treated SH-SY5Y for 24 h with 5 mM DCA (Dichloroacetate, a pyruvate analog), a known inhibitor of PDKs (Supplementary Fig. 5A–C). As expected, phosphorylated PDHA1 levels were decreased in the DCA-treated cells; again, as reported before, a reduction in PDK4 protein level was also detected [50, 51]. Surprisingly, ER^UPR^ and mito^UPR^ markers analyzed also showed a simultaneous decrease in the DCA-treated cells. Additionally, the average mitochondrial length was more in the DCA-treated cells (Supplementary Fig. 5D–F). These effects could plausibly be attributed to reduced PDK4 levels, further confirming the importance of PDK4 in regulating the mitochondria. Our results suggest that DCA acts not only as a kinase inhibitor of PDK4, rather it also affects non-canonical kinase-independent activities of this protein by reducing the levels of PDK4.

### Altered PDK4 levels affect mitochondrial function

**Fig. 6:**
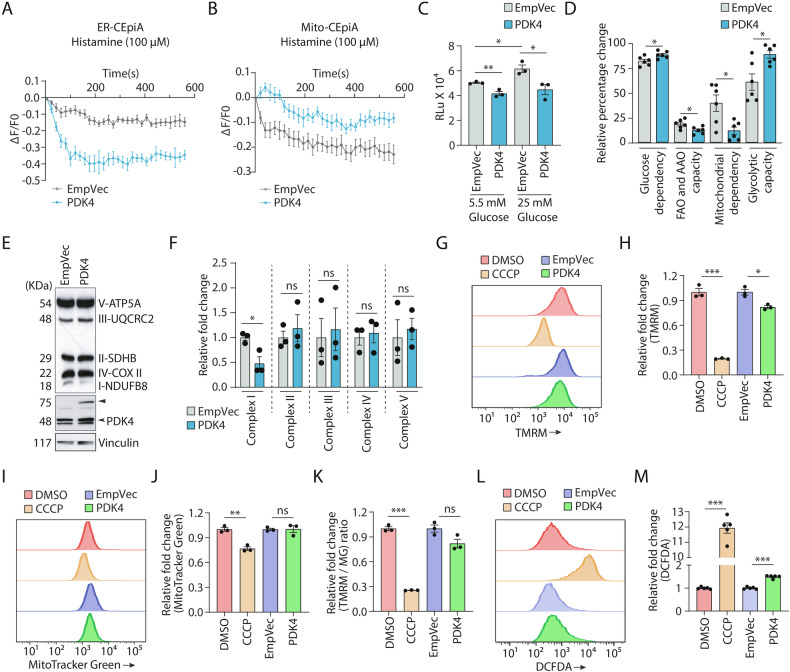
Increased PDK4 regulating mitochondrial functionality. **A** Graph showing fluorescence intensity of ER-CEpiA obtained from control (EmpVec) and PDK4-transfected cells after histamine (100 μM) treatment. **B** Graph showing fluorescence intensity of mito-CEpiA obtained from control (EmpVec) and PDK4-transfected cells after histamine (100 μM) treatment. Intensity of ER-CEpiA or mito-CEpiA was normalized at each time point with either ER-GFP or mitoRFP, respectively. ΔF = Intensity at time ‘t’ – Intensity at time ‘0’. F0 = Intensity at time ‘0’, F = Intensity at time ‘t’. ΔF/F0 was plotted against time from mean ± SEM of 3 independent experiments. **C** Graph represents total ATP production measured by luminescence-based study in SH-SY5Y cells, transfected with indicated constructs under normal (5.5 mM) or high glucose (25 mM) conditions, in RLu (relative luminescence) units. Data represent mean ± SEM of 3 independent experiments. **D** Plot represents the percentage of ATP generated from glucose dependence, FAO (fatty acid oxidation) and AAO (amino acid oxidation) capacity, mitochondrial dependence and glycolytic capacity of cells transfected with indicated constructs. Data is shown as mean ± SEM of 3 independent experiments. **E** Cells transfected with indicated constructs were immunoblotted to detect expression of indicated proteins. ► Exogenous (PDK4-RFP), ➤ endogenous. **F** Graphs represent change in expression of different proteins, as shown in (**E**). Data represent the mean ± SEM of 3 independent experiments. **G** Representative flow cytometry histogram plots the intensity of TMRM (250 nM) signals in cells either transfected with the indicated constructs or treated (DMSO or CCCP). **H** Graph shows quantification of TMRM mean fluorescence intensity measured by FACS, as shown in (**G**). Data represent mean ± SEM of 3 technical replicates. **I** Representative flow cytometry histogram plots intensity of MitoTracker Green FM (250 nM) signals in cells either transfected with the indicated constructs or treated (DMSO or CCCP). **J** Graph shows quantification of MitoTracker Green mean fluorescence intensity with data from (**I**). Data represent mean ± SEM of 3 technical replicates. **K** Histogram of TMRM/MitoTracker Green signal ratios with data from (**H**, **J**). Data represent mean ± SEM of 3 technical replicates. **L** Representative flow cytometry histogram plot shows the intensity of DCFDA (1 μM) signals in cells either transfected with the indicated constructs or treated (DMSO or CCCP). **M** Graph plots data of DCFDA mean fluorescence intensity from (**L**). Data represent mean ± SEM of 5 technical replicates. *p ≤ 0.05; **p ≤ 0.01; ***p ≤ 0.001; ns not significant (estimated via unpaired two-tailed Student’s *t*-test).

Not just morphology, increased PDK4 levels also altered mitochondrial function (Fig. 6). Since MAM junctions play an important role in Ca^2+^ buffering, we investigated the effect of PDK4 on this. SH-SY5Y cells were co-transfected with control vector or WT PDK4, along with ER-CEpiA (an ER Ca^2+^ indicator) and ER-GFP. Histamine (100 μM) was added and time lapse imaging was followed for 10 min (Fig. 6A). PDK4 over-expressing cells showed a rapid decline in the fluorescence intensity of ER-CEpiA over time; fluorescence intensity of ER-GFP remained unaltered. The rate of decrease in fluorescence intensity of ER-CEpiA was significantly lower in the control cells. Further, cells were co-transfected with mito-CEpiA (a mitochondrial Ca^2+^ indicator) and mitoRFP, along with WT PDK4 or control. These cells were similarly treated with 100 μM histamine and imaged live. Interestingly, an increment in fluorescence intensity of mito-CEpiA was observed in PDK4-expressing cells when compared with the controls (Fig. 6B). The fluorescence intensities were normalized with ER-GFP (KDEL-GFP) and mitoRFP for each time point, respectively. Histamine is known to induce Ca^2+^ release from the ER [5, 52, 53] with a subsequent increase in mitochondrial matrix Ca^2+^ [54, 55]. Rapid Ca^2+^ release from ER (declined ER-CEpiA intensity) and a concomitant increase in mito-CEpiA intensity in cells with exogenous PDK4 suggested the presence and involvement of larger number of MAM junctions, when compared with the controls.

We also observed that exogenous expression of PDK4 led to a reduction in total ATP production (Fig. 6C). For this, cells transfected with PDK4 were either maintained in normal (5.5 mM glucose) or high glucose (25 mM) media. Increased glucose concentration in culture media is known to positively regulate ATP production [56], as was evident in the control cells. However, in PDK4 over-expressing cells ATP production remained almost unaltered, irrespective of the glucose concentration (Fig. 6C). Metabolic profiling (as reported earlier [57]) was carried out in SH-SY5Y cells using 2-Deoxy-D-Glucose (100 mM), Oligomycin A (1 μM), or a combination of both for 60 min in PDK4 or control vector transfected cells, to systematically inhibit energy metabolic pathways and estimate differential cellular dependencies and contributions towards ATP production. PDK4 over-expressing cells showed a shift towards glycolytic dependence, indicative of a metabolic reprogramming that allows cells to generate ATP in the absence of efficient mitochondrial respiration. This was most importantly associated with a significant decrease in mitochondrial dependency and pronounced increase in glucose dependency and glycolytic capacity, when compared to the control (Fig. 6D). ATP generated from amino acid and fatty acid metabolism was reduced upon PDK4 overexpression. Our results fit well with previous observation that PDK4-mediated phosphorylation and inhibition of PDC complex can negatively affect mitochondria-mediated ATP generation [58–60]. Next, Western blot analyses of the five different OXPHOS complex subunit proteins (NDUFB8, SDHB, UQCRC2, COX-II, ATP5A for complex I-V, respectively) showed significantly low levels of Complex-I protein (NDUFB8) in PDK4 overexpressed condition; proteins of the other complexes remained unaltered across samples (Fig. 6E, F). However, we could not detect any significant alteration in expression of the representative subunits of OXPHOS complexes (ND2, SDHA, CYTB, COX-II, ATP8 for complex I-V, respectively) at the mRNA levels by qRT-PCR (Supplementary Fig. 6A). Additionally, flow cytometric analyses with TMRM and MitoTracker Green FM co-staining showed a reduction in mitochondrial potential in the presence of exogenous PDK4 (Fig. 6G, H) as compared to the control. This was also reflected in mitochondrial potential to mass ratio (Fig. 6K), since the mitochondrial mass did not get significantly altered in these cells (Fig. 6I, J). CCCP (10 μM) (carbonyl cyanide m-chlorophenyl hydrazone), a protonophore, was used as positive control. Treated with this drug, cells showed a significant reduction in the membrane potential, as well as mitochondrial mass and potential to volume ratio (Fig. 6G–K). These observations clearly suggested an abundance of dysfunctional depolarized mitochondria. Cells loaded with TMRM and MitoTracker Green FM, and imaged live corroborated the flow cytometry results (Supplementary Fig. 6B–E). Here again, lower TMRM intensity in PDK4 over-expressing cells compared to the controls, indicated reduced mitochondrial potential; similar signal intensities of MitoTracker Green FM suggested unaltered mitochondrial mass. Oligomycin (1 μM) and CCCP (10 μM) were used as controls, where treatment with the former drug leads to an increase in mitochondrial potential, while the latter one has an opposite effect [61]. Higher amounts of reactive oxygen species (ROS) were detected in cells with exogenous PDK4 using flow cytometric analyses with DCFDA staining, further supporting the hypothesis that elevated PDK4 levels promote mitochondrial stress. CCCP-treated cells served as the positive control (Fig. 6L, M).

### Excess PDK4 impairs autophagy

**Fig. 7:**
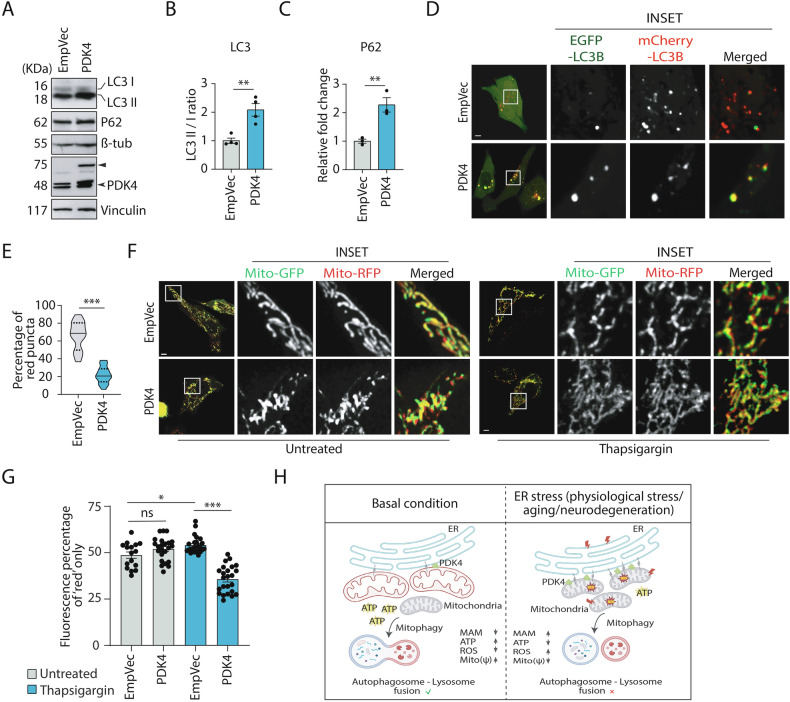
PDK4 overexpression regulates autophagic flux clearance. **A** SH-SY5Y cell lysates from empty vector (EmpVec) and PDK4-transfected samples were immunoblotted to detect expression of indicated proteins. ► Exogenous (PDK4-RFP), ➤ endogenous. **B** Graph represents change in ratio of LC3-II and LC3-I expression, as mentioned in A. Data represent the mean ± SEM of 4 independent experiments. **C** Graph represent change in expression of indicated protein, as mentioned in (**A**). Data represent the mean ± SEM of 3 independent experiments. **D** Cells were transfected with mCherry-EGFP-LC3B, PDK4 and control (EmpVec), imaged under live-cell conditions. Enlarged views of the areas within the white boxes are shown (inset). Scale bar: 5 µm. **E** Violin plot shows the percentage of mCherry-LC3B (red) puncta (red puncta/total puncta*100), as described in (**D**). Solid and dashed black lines mark median and quartiles, respectively. Data shown are of ∼50 cells from 3 independent experiments. **F** Cells co-transfected with mito-mRFP-EGFP, along with PDK4 or control (EmpVec), either treated with TG (0.5 μM) or left untreated, and imaged under live-cell conditions. Enlarged views of the areas within the white boxes are shown (inset). Scale bar: 5 µm. **G** Graph shows the percentage fluorescence intensity of Mito-mRFP (red only signal), as measured in (**F**). Data shown are of ∼40 cells from 3 independent experiments. **H** A graphical representation demonstrating the impact of increased PDK4 in maintaining ER-mitochondrial communication and mitochondrial homeostasis. In basal condition, PDK4 level is relatively low, which corresponds to increased mitochondrial ATP production, membrane potential, lower ROS level and also lesser number of ER-mitochondria contact sites (MAM); autophagosome-lysosome fusion remains functional. However, ER stress, aging, and neurodegeneration-induced overexpression of PDK4 leads to altered mitochondrial homeostasis (more fragmented and depolarized mitochondria) and ER-mitochondrial communication (increased MAMs); reduced ATP production, increased ROS level, impaired autophagosome-lysosome fusion. Subsequently, exerting stress-mediated disease progression. *p ≤ 0.05; **p ≤ 0.01; ***p ≤ 0.001; ns not significant (estimated via unpaired two-tailed Student’s *t*-test, one-way ANOVA with Tukey’s corrections).

The stressed and dysfunctional mitochondria are known to be cleared by mitophagy [62]. ER-mitochondria contact sites or MAM junctions can facilitate autophagosome formation [19]. Further, compromised autophagy and mitophagy are known to be associated with multiple neurodegenerative diseases, including AD [63]. LC3II/I ratio and P62 proteins were significantly higher in cells with exogenous expression of PDK4, suggesting impaired autophagic clearance (Fig. 7A–C). Inhibiting lysosomal acidification with bafilomycin A1 treatment (300 nM, 10 h) should block LC3 and P62 degradation. In control cells, this drug treatment significantly increased endogenous LC3II/I ratio and P62 protein levels compared with the untreated samples (Supplementary Fig. 7A–C). However, under PDK4 overexpressed conditions, no significant change was seen upon drug treatment. This confirmed that an increase in PDK4 levels was sufficient to block autophagic flux.

To measure the autophagy degradative competence, cells were treated with rapamycin (200 nM, 24 h) (Supplementary Fig. 7D–F). Rapamycin treatment led to elevated LC3II/I ratio but a decrease in P62 protein levels in control cells, suggesting normal autophagic flux. On the contrary, in PDK4 over-expressing cells, LC3II/I ratio as well as P62 levels did not get significantly altered upon rapamycin treatment. Live-cell imaging using dual tagged mCherry-EGFP-LC3B supported these data, where we observed significantly lower percentage of red puncta (marking acidic vesicles) when PDK4 was overexpressed, compared to the controls (Fig. 7D, E). Next, we checked if impaired autophagy could compromise the clearance of dysfunctional mitochondria. While live-cell imaging of cells co-transfected with control vector or PDK4 along with mito-mRFP-EGFP did not show any significant change across samples (Fig. 7F, G); treatment with 0.5 μM thapsigargin for 3 h elicited a drastic change in the autophagic clearance of dysfunctional mitochondria in both control vector and PDK4-transfected cells. Significantly low fluorescence intensities of “red only” mitochondria (marking mitochondria in lysosomes) in PDK4-expressing cells were observed. Two complimentary experiments, further supported perturbation of autophagic flux in cells exogenously expressing PDK4. First, cells co-transfected with PDK4 or control vectors along with GFP-LC3, were stained with MitoTracker Deep Red FM, and imaged live (Supplementary Fig. 7G, H). Second, in a similar imaging experiment, CD63-YFP (to mark acidic vesicles) was used instead of GFP-LC3 (Supplementary Fig. 7I, J). We detected a reduced number of puncta positive for CD63 and mitochondria, and an increase in the number of LC3 vesicles engulfing mitochondria. These observations together suggested that PDK4 overexpression-potentiated increase in mitochondrial fission led to more number of mitophagosome formation. However, decreased fusion with acidic vesicles (amphisomes and autolysosomes), as indicated by lower number of CD63 vesicles positive for mitochondria supported the hypothesis that a blockage in fusion of autophagosome/mitophagosome with lysosome was facilitated by PDK4 overexpression.

## Discussion

In this study, we not only propose a non-canonical role of PDK4 in affecting mitochondrial morphology, function and clearance, we identify this regulator of oxidative phosphorylation as one of the probable connecting links between ER^UPR^ and mito^UPR^. Comprehensive analyses under various chemical as well as physiological ER stress conditions reveal PDK4 as a novel marker that gets deregulated at the mitochondria, and hence may act as a contributing factor to aging and neurodegeneration. We show that ER stress-mediated elevated PDK4 levels affect ER-mitochondria contact sites, morphologically and functionally compromise mitochondria (Fig. 7H).

There are distinct organellar mechanisms for coping with cellular stress at the ER and mitochondria, termed ER^UPR^ and mito^UPR^, respectively. These primarily regulate the accumulation of misfolded protein within the respective compartments. Build-up of unfolded, misfolded, or erroneously modified proteins in the ER lumen triggers a multilayered cellular response, activating one or more of the three canonical pathways of ER stress (IRE1α, PERK and ATF6). These pathways work in a well-coordinated manner to elicit increased chaperone expression to enhance protein folding, reducing protein load by translational attenuation and initiating the ER-associated degradation (ERAD) process to get rid of the terminally misfolded proteins [64]. Similarly, mito^UPR^ controls the response towards impaired mitochondrial protein import or accumulation of proteins in the matrix, leading to aberrant mitochondrial function. This stress response helps to restore mitochondrial homeostasis by activating chaperones like HSPD1 (Heat Shock Protein Family D (Hsp60) Member 1), HSPE1 (Heat Shock Protein Family E (Hsp10) Member 1) and matrix proteases, like CLPP (Caseinolytic Mitochondrial Matrix Peptidase Proteolytic Subunit), LONP1 (Lon protease 1); and can also induce mitochondrial fission by increasing DRP1 expression. Similar to ERAD, defective parts of mitochondria or the entire organelle may be eliminated by various degradation pathways, including mitophagy, reticulo-mito-phagy, and mitochondria-associated degradation (MAD) [20, 61, 65, 66]. Our results posit PDK4 at a junction where it shows involvement in both ER and mitochondrial stresses.

Many neurodegenerative diseases are characterized by the presence of misfolded protein aggregates and upregulation of multiple ER stress markers [67–70]. It is well-established that ER is an extremely dynamic organelle, always in crosstalk with various other cellular compartments [71–74]. Hence, it is logical to hypothesize that any cellular insult that elicits ER stress should also percolate to other organelles, simultaneously destabilizing them. While the complexity of organellar interconnectivity is progressively being unraveled, our knowledge of the molecular executors that sense and transmit ER stress to other organelles (probably as a defense mechanism to alleviate it) remains sparse. Literature suggests increased mitochondrial fragmentation, abnormal functioning, cytosolic extrusion of mtDNA, impaired mitophagy—all features of deregulated mitochondria—are often associated with chronic neurodegeneration [16, 75–77]. Further, ER-mitochondria contact sites take part in regulating lipid, glucose, fatty acid metabolism, calcium signaling, autophagosome formation, and apoptosis, which are frequently found to be erroneous in neurodegeneration [19, 78–82]. So, it is crucial to identify proteins from other organelles, especially mitochondria, that would participate in organellar crosstalk during ER stress-mediated neuronal death. Our results suggest that PDK4 emerges as a potential mitochondrial sensor or effector of ER stress—future experiments, beyond the scope of this study, would be required to fully establish this.

Studies have shown that Aβ peptide, a hallmark of AD, may be generated at MAM junctions. Further, other essential molecular players, like amyloid-β protein precursor (AβPP), β-site APP cleaving enzyme 1 (BACE1), C-terminal fragment (CTF) of 99 amino acids (C99), the γ-secretase complex, and APP intracellular domain (AICD), closely associated with the pathogenesis of AD, are all detected at MAM junctions [45, 83]. Aβ oligomeric aggregations are reported in the ER of hippocampal neurons, possibly contributing to their accelerated loss during neurodegeneration [84]. Aβ plaque formation in mitochondria may lead to mitochondrial damage [85]. In addition, it has been reported that MAM resident proteins like MFN2, sigma-1 receptor deficiency can lead to decreased ER-mitochondria tethering, thereby reducing the deposition of toxic Aβ oligomers [86, 87]. These evidences support the significance of MAMs in AD pathogenesis. Other neurodegenerative diseases like Parkinson’s disease (PD), Huntington’s disease (HD), Amyotrophic lateral sclerosis (ALS), Charcot-Marie-Tooth (CMT) disease, Wolfram syndrome (WS) are also associated with altered MAM formation or function [88–92]. MAMs have also been suggested to be deregulated in various cancers [93, 94], viral infection [95] and insulin resistance and diabetes [96]. Interestingly, the presence of PDK4 in MAM junctions is reported during aberrant insulin signaling [29]. Together, these support our findings that PDK4 gets recruited to ER-mitochondria contacts during ER stress, and its elevated level increases MAM formation and disturbs mitochondrial homeostasis. Some features of neurodegeneration are also shared during the natural process of aging, one such being ER stress and consequential evocation of ER^UPR^ [97]. Increased PDK4 protein levels in different brain regions, especially in the hippocampal region of aged rats as well as AD mice models clearly support this hypothesis. It is well documented that while UPR might have an initial neuroprotective role, sustained stress can initiate or potentiate the progress of neurodegeneration [70].

Altered mitochondrial dynamics and imbalance in mitochondrial fission-fusion machinery are often implicated in neurodegenerative diseases, including AD [16, 98]. Here, we show that PDK4 overexpression could lead to mitochondrial fragmentation, which in turn could be executed either via DRP1 recruitment [49] or through some other novel molecular player. However, a detailed investigation of this is beyond the scope of the present study.

Kinase-compromised PDK4 mutant elicited expression of ER^UPR^ and mito^UPR^ markers, and mitochondrial fragmentation upon exogenous expression, very similar to our observations with wild-type PDK4 protein. These results strengthen our hypothesis that the involvement of PDK4 in this is probably independent of its known kinase activity in the TCA cycle. The presence of higher numbers of ER-mitochondria contact sites, along with enhanced Ca^2+^ efflux from the ER, supports the correlation of increased PDK4 levels in stress conditions. However, this does not rule out the involvement of other molecular partners, which could complement or regulate PDK4 at the MAM junctions.

In AD, various players of the glycolytic pathway, TCA cycle, and oxidative phosphorylation [99], interestingly, complex I of OXPHOS is significantly reduced [100]. The antagonistic effect of increased PDK4 on mitochondrial ATP production and functionality suggests that this could be one of the contributing factors in this neurodegenerative disease. We have also observed increased ROS production and more depolarized mitochondria in PDK4 overexpressed cells, which may generate a greater pool of damaged mitochondria. Elevated oxidative stress is often reported in AD [101, 102]. Neurodegenerative diseases are also associated with deregulated autophagic clearance [103]. Our results suggest attenuated fusion of autophagosomes with lysosomes upon exogenous expression of PDK4. More importantly, this drastically affects mitophagic clearance of dysfunctional mitochondria. There is evidence to suggest the involvement of PDK4 in the regulation of autophagic flux under cellular stress; while it is reported that higher PDK4 levels can elevate autophagy [50], it is also shown to impair autophagy in VSMCs (Vascular smooth muscle cells) calcification [104]. In our system, we find that while autophagy is induced upon PDK4 overexpression, autophagic degradation or the flux clearance is impaired. PDK4 emerges as one of the potential mitochondrial targets that are altered during ER stress, a molecular phenomenon central to aging and neurodegeneration. Not only does ER stress affect PDK4 levels, but this protein in turn can also positively regulate ER stress, while simultaneously deregulating mitochondrial morphology and function. Our study provides one of the probable avenues by which ER^UPR^ could affect mito^UPR^.

## Materials and methods

### Constructs

Full-length PDK4 in pcDNA 4.1 and pDsRed1-N1 vector was generated. Y157F PDK4 (kinase-mutant) construct was generated by site-directed mutagenesis. ER-GFP (GFP–KDEL) was a gift from Erik L. Snapp (Janelia, USA). Wild-type prion, prion mutant constructs (A117V, KHII) were gifts from Ramanujan S. Hegde (MRC-LMB, UK). mCherry-EGFP-LC3B was a gift from Terje Johansen (Arctic University, Norway). mito-mRFP-EGFP was a gift from Benu Brata Das (IACS, India). Amyloid precursor protein intracellular domain (AICD-GFP) and GFP-LC3 construct were gifts of Debashis Mukhopadhyay (SINP, India). CD63-YFP was a gift from Rafael Mattera and Juan Bonifacino (Bethesda, MD, USA). ER-CEpiA and mito-CEpiA were obtained from Addgene (Cambridge, MA, USA).

### Antibodies

Antibodies that were used in this study are provided in Table 1.

**Table 1:** List of used antibodies.

Antibody	Manufacturer (Cat no.)	Dilution
1 PDK4	Abcam, #110336^a^	1:1000
2 BiP	Cell Signaling Technology (CST), #3177	1:1000
3 CHOP	Cell Signaling Technology (CST), #2895	1:1000
4 HERPUD1	Invitrogen, #26301	1:1000
5 p-PDHA1(ser293)	Abcam, # 92696	1:2000
6 PDHA1	Abcam, # 110334	1:2000
7 PDK1	ABclonal, #A0834	1:1000
8 PDK2	ABclonal, #A4737	1:1000
9 PDK3	ABclonal, #A12480	1:500
1 0MFN2	Cell Signaling Technology (CST), #(D1E9), 11925	1:1000
11 VDAC1	Abcam, #15895	1:1000
12 Calnexin	Cell Signaling Technology (CST), #(C5C9), 2679	1:1000
13 OXPHOS cocktail	Abcam, #110411	1:1000
14 LC3	Novus Biologicals, #NB100-2220	1:1000
15 P62	Novus Biologicals, #NBP1-48320	1:2000
16 ATF5	Abcam, #184923	1:1000
17 CLPP	Abcam, #124822	1:1000
18 HSP60	Abcam, # 59457	1:1000
19 PTDSS1	antibodies.com, #A90494	1:1000
20 Vinculin	Abcam, #129002	1:5000
21 β-actin	Abcam, # 8226	1:5000
22 GAPDH	Santa Cruz Biotechnology (SCBT), #SC365062	1:5000
23 Anti-rabbit IgG, HRP-linked Antibody	Cell Signaling Technology (CST), #7074	1:3000
24 Anti-mouse IgG, HRP-linked Antibody	Cell Signaling Technology (CST), #7076	1:3000
25 RFP	Ramanujan S. Hegde (MRC-LMB, UK)	1:5000

^a^The PDK4 antibody (Abcam, #110336) was used in previously published reports [108–110] and its specificity was verified using knock-down experiments [109]. Here, we also checked the specificity of the PDK4 antibody (Abcam, #110336) using similar protocols by either knocking down (via siPDK4 and shPDK4) or inhibiting (via DCA) the PDK4. siRNA pool targeting Pdk4 was designed as mentioned in Sun et al. [108] and provided by GenScript. shPDK4 was obtained from Sigma-Aldrich (#TRCN0000006264). In both siRNA and shRNA-mediated PDK4 knock-down and in DCA-treated condition reductions in PDK4 protein levels could be observed as captured successfully by the PDK4 antibody (Abcam, #110336) (Supplementary Fig. 8).

### Reagents

Thapsigargin (Sigma-Aldrich, #T9033), Tunicamycin (Sigma-Aldrich, #T7765), Lipofectamine 2000 (Thermo Fisher Scientific, #11668019), MitoTracker Red FM (Thermo Fisher Scientific, #22425), MitoTracker Deep Red FM (Thermo Fisher Scientific, #22426), MitoTracker Green FM (Thermo Fisher Scientific, #M7514), CM-H2DCFDA (Thermo Fisher Scientific, #C6827), TMRM (Thermo Fisher Scientific, #T668), CCCP (Sigma-Aldrich, #C2759), percoll (Sigma-Aldrich, #P1644), Amyloid β Protein Fragment 1-42 (Aβ42) (Sigma-Aldrich, #A9810), Luminescent ATP detection assay kit (Abcam, #113849), 2-Deoxy-D-glucose (2-DG) (TCI, #154-17-6), Oligomycin A (Sigma-Aldrich, #75351), Rapamycin (Sigma-Aldrich, # R0395), Bafilomycin A1 (Sigma-Aldrich, #B1793), DCA (Sigma-Aldrich, #D54702), Histamine (Sigma-Aldrich, # H7125).

### Animal experiment

Sprague Dawley male rats (1 month and 12 months old), *Apoe*^*tm1Unc*^ mutant strain of C57BL/6J mice (6–9 months old) (MMRRC stock#002052, commonly referred as ApoE KO) were kept in the animal house (22 ± 2 °C, 60 ± 5% humidity, with 12 h light-dark cycle) of CSIR-Indian Institute of Chemical Biology, Kolkata, India. Food and water were provided ad libitum. Transgenic mouse line for AD (B6SJL-Tg (APPSwFlLon, PSEN1*M146L*L286V)6799Vas/Mmjax) acquired from the Jackson Laboratory was bred in the animal house facility of the National Brain Research Centre in 12-h light and 12-h dark cycle with ad libitum access to pelleted diet and water. This line (MMRRC stock#34840) is commonly referred to as 5xFAD (7–9 months old). All animals were used for experiments after proper genotyping. The hippocampus, cortex, striatum, and cerebellum were carefully isolated using microdissection tools under a stereomicroscope. The brain areas were dissected accurately and consistently by using the appropriate anatomical landmarks. Then the isolated brain regions were homogenized in ice-cold Radioimmunoprecipitation assay buffer (RIPA, 50 mM Tris-HCl containing 1 mM EDTA, 150 mM NaCl, 1% Nonidet p-40, 0.25% sodium deoxycholate; pH 7.5) supplemented with protease inhibitors (Invitrogen). The lysate was kept on ice for 30 min and centrifuged at 10,000×*g* for 10 min at 4 °C, and the supernatant was collected. Protein estimation was performed using Bradford protein assay, and 25 μg protein was used per sample for western blotting. ApoE KO mice and Sprague Dawley rats were provided by Arun Bandyopadhyay (CSIR-IICB) and Joy Chakraborty (CSIR-IICB), respectively. Isolation of different brain regions and lysate preparation for both animal strains were performed under the supervision of Joy Chakraborty (CSIR-IICB). Isolated 5xFAD mice brain regions were provided by Bhavani Shankar Sahu (NBRC); samples were prepared for Western blotting as described above.

### Human tissue sample

All three human brain lysates (one each from control, AD and PD patients) were obtained from Novus Biologicals, LLC (NB820-59177, NB820-59363, NB820-59407). Three technical replicates (with one biological replicate) were used for each group in the study. All three patient samples belong to Caucasian race (normal—77 years old male, AD—87 years old male, PD—78 years old female).

### Cell culture

SH-SY5Y Human neuroblastoma cells were a gift from Debashis Mukhopadhyay (SINP, Kolkata, India). U2OS Human osteosarcoma cells were a gift from Chandrama Mukherjee (Presidency University, Kolkata, India). Cells were grown in 10% fetal bovine serum (FBS - HI; Gibco) in Dulbecco’s modified Eagle’s medium (DMEM) (Gibco, #31600034, #12100046) at 37 °C and 5% CO_2_. For transfections of cells, Lipofectamine 2000 was used (Invitrogen, Carlsbad, CA, USA) as per the manufacturer’s instructions. 24 h after transfection, cells were lysed using a suitable lysis buffer.

### Immunocytochemistry

For immunocytochemistry, cells were fixed with either 4% formaldehyde or methanol as per the requirement of the antibody, as described previously [111, 112]. Cells were permeabilized using phosphate-buffered saline (PBS) containing 10% FBS and 0.1% saponin (Sigma-Aldrich) for 60 min, followed by overnight staining in primary antibody at 4 °C and 60 min incubation in secondary antibody at room temperature. The samples were then imaged using confocal microscope.

### Imaging and image analyses

Confocal imaging was performed using LSM 980, LSM710 ConfoCor 3 and super-resolution imaging was performed with LSM 900 and Nikon A1R+Ti-E with N-SIM and FCS microscope systems. A diode laser (BFP excitation with 405 nm line), an Ar-ion laser (GFP, Alexa Fluor 488 excitation with the 488 nm line) and He-Ne lasers (RFP, Alexa Fluor 546 excitation with the 543 nm line; MitoTracker Red FM excitation with the 561 nm line; Alexa Fluor 633 and MitoTracker Deep Red FM excitation with 640 nm line) were used with 63 × 1.4 NA oil immersion objectives. Transfected cells were imaged in CO_2_-independent medium (Thermo Fisher Scientific), maintaining conditions of live-cell imaging as described previously [20].

Mitochondrial length measurement was done manually by drawing lines along the entire length of each mitochondrion from 2D confocal micrographs of cells of resolution 1024 × 1024 at 300 dpi; where mitochondria were stained with MitoTracker Red FM, MitoTracker Deep Red FM, or MitoTracker Green FM (as indicated). mCherry-LC3B puncta were counted from 2D confocal micrographs of cells of resolution 1024 × 1024 at 300 dpi. Measurements were done using Fiji. For mito-mRFP-EGFP (mitophagy) experiment, fluorescence intensities in green and red channels were measured in Fiji; intensity of the red signal was represented as percentage of the total fluorescence intensity. For TMRM and MitoTracker Green FM co-stained live-cell imaging, fluorescence intensities in red and green channels were measured in Fiji and represented as fold change with respect to the controls. For LC3-mitochondria or CD63-mitochondria experiments, number of puncta per cell positive for LC3 or CD63, and mitochondria were counted manually. Co-localization analysis of ER-mitochondria signals was carried out from cell images of resolution 1024 × 1024 using Fiji and represented as Pearson’s coefficient. Super-resolution microscopic images of cells with a resolution of 1024 × 1024 pixels were utilized for quantitative image analysis. In this experiment, KDEL-GFP transfected cells under the specified conditions were loaded with MitoTracker Red FM, immune-stained against PDK4, MFN2 or HSP60. Each color channel image was processed separately using MATLAB’s image processing toolbox [105]. MATLAB tools like ‘*imadjust*’ was used to adjust, sharpen, and thresholding each image uniformly. Very small puncta (<10 pixels) were removed using morphological operation. Co-localization was quantified from the processed binary images using MATLAB inbuilt function ‘*intersect*’ to calculate the number of pixels of each color channel sharing same coordinates. Fold change of overlapping pixels across samples is represented as overlap index.

### Calcium imaging using ER-CEpiA and mito-CEpiA

SH-SY5Y cells were co-transfected with either ER-GFP (KDEL-GFP) and ER-CEpiA, or mitoRFP and mito-CEpiA to measure ER or mitochondrial calcium dynamics, respectively in PDK4 or empty vector-transfected cells. ER-CEpiA and mito-CEpiA are organelle-specific Ca^2+^ sensitive indicators. 24 h post-transfection, cells were imaged live over the indicated time periods. 100 μM Histamine was used to deplete Ca^2+^ from its intracellular ER store, and expected corresponding transient increase at mitochondrial was recorded. Fluorescence corresponding to ER-CEpiA (561 nm), mito-CEpiA (488 nm), ER-GFP (488 nm) and mitoRFP (543 nm) was monitored. Intensities of ER-CEpiA and mito-CEpiA were normalized with ER-GFP and mitoRFP, respectively, at all time points using Fiji software. ΔF/F0 was plotted against time to check for calcium dynamics between the ER and mitochondria upon treatment with histamine, across different conditions.

### Western blotting

The protocol for western blotting was as described previously [113]. 10% Tris-tricine gels or 7.5% Tris-glycine gels were used for SDS–PAGE followed by western blotting. Quantification of western blots was performed using GelQuant software. At least three independent experiments were performed, and band intensities were normalized to loading controls. *p*-values were determined using Student’s *t*-test or one-way ANOVA, whichever was applicable.

### Isolation of MAM, mitochondria, ER and cytosolic fractions

The MAM fraction was isolated following an established protocol [106]. Briefly, cells were cultured in 100 mm plates, followed by either TG or vehicle treatment, they were washed with ice-cold PBS, collected with cell scraper, and kept on ice. Cells were centrifuged and the pellet was re-suspended in homogenization buffer (30 mM Tris-HCl (pH 7.4) buffer containing 225 mM mannitol, 75 mM sucrose and protease inhibitors). Homogenization procedure was performed manually with 20 strokes of a 27-gauge needle, on ice. At first, nuclei and unlysed cells were pelleted by centrifugation at 600×*g* for 5 min. This was repeated, and after the second centrifugation, the supernatant was collected. This was followed by a spin at 7000×*g* for 10 min. The pellet was further processed to obtain pure mitochondria and MAM fraction. The supernatant was further centrifuged at 20,000×*g* for 30 min. Enriched ER and cytosolic fractions were separated in the pellet and supernatant, respectively, when the collected supernatant was subjected to ultra-centrifugation at 1,00,000×*g* for 1 h. The pellet containing mitochondrial fraction was re-suspended in Homogenization buffer, and then this was centrifuged at 7000×*g* for 10 min and again after re-suspension of the pellet in the same buffer, centrifugation of 10,000×*g* for 10 min was done. The collected pellet was re-suspended in MRB buffer (250 mM mannitol, 25 mM HEPES (pH 7.4) and 1 mM EGTA) and layered on top of 30% Percoll medium (225 mM mannitol, 5 mM HEPES (pH 7.4) and 0.5 mM EGTA) and centrifuged at 95,000×*g* for 30 min. The MAM fraction was extracted from Percoll gradient by centrifugation at 6300×*g* at 10 min and further purified by ultra-centrifugation at 1,00,000×*g* for 1 h to remove remaining mitochondrial fraction. Similarly, the pure mitochondria fraction was collected from Percoll gradient and centrifuged at 6300×*g* for 10 min to obtain mitochondrial pellet. The entire fractionation procedure was performed throughout at 4 °C. Fractions were analyzed by Western blotting with suitable antibodies for different fractions.

### Quantitative real-time PCR (qRT-PCR)

RNA extraction from SH-SY5Y cells is carried out using TRIzol reagent (Thermo Fisher Scientific) for control and drug-treated or transfected samples. cDNA synthesis from total RNA is performed using High-Capacity cDNA Reverse Transcriptase Kit (Applied Biosystems) following the manufacturer’s protocol.

Quantitative real-time experiments were performed using PowerUP SYBR Green Master Mix (Applied Biosystems, #A25742) with gene-specific primers, according to the manufacturer’s instructions. The primer sequences of the related genes are – PDK4 (forward primer, 5′-AGATACACTCATCAAAGTTCGAA-3′; reverse primer, 5′- TCATCAGCATCCGAGTAGA -3′), BiP (forward primer, 5′-GAAAGAAGGTTACCCATGCAGT-3′; reverse primer, 5′-CAGGCCATAAGCAATAGCAGC-3′), CHOP (forward primer, 5′-CCAGCAGAGGTCACAAGCAC-3′; reverse primer, 5′-TTCTCCTTCATGCGCTGC-3′), XBP1-s (forward primer, 5′-GAGTCCGCAGCAGGTGC-3′; reverse primer, 5′-TGATGACGTCCCCACTGAC-3′), β-Actin (forward primer, 5′-CACTGGCATCGTGATGGA-3′; reverse primer, 5′-CCGTGGCCATCTCTTGCT-3′). ND2, SDHA, CYTB, COX-II and ATP8 primers were gifts from Soumen Kanti Manna (SINP, Kolkata, India). β-Actin was used as the reference gene. Relative expression of each gene was measured using the gene’s 2^-ΔΔCt^.

### mRNA transcriptomics

RNA was isolated as described above. The quantity and quality of the isolated RNA were evaluated using Qubit 4.0 (Thermo Fisher Scientific). Transcriptome sequencing was performed using Illumina Novaseq 6000 and has been deposited in NCBI’s Gene Expression Omnibus [107] and is accessible through GEO Series accession number GSE266545 (https://www.ncbi.nlm.nih.gov/geo/query/acc.cgi?acc=GSE266545). A fold change of ±2 in the log2 scale and *p*-value of 0.01 were set as a cutoff for the categorization of Differentially Expressed Genes (DEGs). MitoCarta database (v3.0) was used to identify mitochondrial genes that were among the DEGs.

### Multiple sequence alignment (MSA)

Full-length FASTA sequences of proteins were retrieved from NCBI protein (https://www.ncbi.nlm.nih.gov/protein) database and provided as input in Clustal Omega (https://www.ebi.ac.uk/jdispatcher/msa/clustalo). Sequence identity matrix was obtained from the webserver.

### AD patient-specific GSE data retrieval

BrainProt webserver [40] was accessed to search for PDK4 RNA level in already available GSE datasets (https://www.ncbi.nlm.nih.gov/geo/query/acc.cgi?acc=GSE48350, https://www.ncbi.nlm.nih.gov/geo/query/acc.cgi?acc=GSE5281). Two AD-specific datasets (GSE48350, GSE5281) were considered to analyze the processed RNA intensity of PDK4 in AD brains compared to the control group. These two GSE datasets had significantly more patient numbers (>50) compared to the other datasets, hence they were selected for analysis.

### ATP measurement and metabolic profiling

The method for ATP measurement and cellular metabolic profiling was carried out as described in detail previously [57]. Cells were counted, and 1 × 10^5^ cells/ml were seeded for each sample. Transfection was performed next, followed by treatment with DMSO, 2-Deoxy-D-Glucose (DG, final concentration 100 mM), Oligomycin A (Oligo, final concentration 1 μM), or combined DG and Oligomycin A for 60 min. ATP production was measured with Luminescent ATP detection assay kit (Abcam, #113849), following manufacturer’s protocol. After cell lysis and substrate addition, luminescence was detected using BioTek SYNERGY HTX Reader at 540 nm wavelength.

2-Deoxyglucose (2-DG) and oligomycin A (OA) inhibit glucose metabolism and oxidative phosphorylation, respectively. Systematic inhibition of ATP production using 2-DG or OA or both (DGOA) was used to define the fractional contribution of the individual modes of ATP production. The fraction of ATP produced by using glucose in glycolysis and TCA cycle, termed as glucose dependence, can be obtained in presence of 2-DG. The rest is basically the fractional contribution of fatty acid and amino acid oxidation to generate ATP via oxidative phosphorylation. This is called fatty acid oxidation and amino acid oxidation capacity; it can be calculated by subtracting the fractional contribution from glucose metabolism (obtained in presence of 2-DG). Use of OA inhibits mitochondrial ATP production. Thus, the fractional contribution of mitochondrial metabolism can be calculated from the relative decrease in ATP level upon OA treatment; this is defined as mitochondrial dependence. The remaining fraction is from glycolysis, which is defined as the glycolytic capacity.

### ROS production analysis

ROS generation was measured using DCFDA (final conc. 1 μM). Cells were washed with PBS, stained with DCFDA, and incubated at 37 °C for 30 min. After diffusing into the cell, DCFDA gets deacetylated by esterases to a non-fluorescent compound, and subsequently oxidized by ROS into a fluorescent compound (DCF). After incubation, cells were washed with PBS and fluorescence was detected using a flow cytometer (BD LSRFortessa) with 485 nm laser and analyzed using FlowJo Software (v10.9.0).

### Mitochondrial potential and mass measurement

Mitochondrial membrane potential and mass were measured by flow cytometer with TMRM (sensitive to mitochondrial potential) and MitoTracker Green FM (used to detect mitochondrial mass, not affected by its potential) co-stained together (final conc. 250 nM) at 37 °C for 30 min, then washed with PBS and fluorescence was measured using a flow cytometer (BD LSRFortessa) with 485 nm and 565 nm lasers and analysed using FlowJo Software (v10.9.0). In imaging experiment, cells were stained as described before, imaged live with 488 nm and 546 nm lasers, and analysed with Fiji software.

### Statistical analysis

Data are expressed as mean ± SEM. Statistical comparisons were performed using a paired Student’s *t*-test and one-way ANOVA, whichever is applicable. Differences were considered statistically significant when the *p*-value was <0.05.

## Supplementary information

Supplementary materials

Main Figures raw source files

Supplementary Figures raw source files

Transcriptomics Data Source File

## Data Availability

All data generated or analysed in this study are included in the published article, supplementary information and source data files. The RNA sequencing data were deposited into the Gene Expression Omnibus database under accession number GSE266545 and are available at the following URL: https://www.ncbi.nlm.nih.gov/geo/query/acc.cgi?acc=GSE266545.
