# Supplementary material for: The impact of ERUPR on mitochondrial integrity mediated by PDK4
Source: Cell Death Dis. 2025 Jul 29;16(1):573. doi: 10.1038/s41419-025-07743-5 (PMC12307875; doi:10.1038/s41419-025-07743-5)

Supplementary Information

Supplementary Figure 1: Effect of ER stress inducing drug on cellular homeostasis

(A) Immunoblotting of lysates against PDK4 and GAPDH (control) from cells treated with Tun (Tunicamycin, 5µg/ml for 3 hrs) to induce ER stress, and compared with controls (DMSO). (B) Graph generated with data from panel A. Data represents mean ± SEM of 3 independent experiments. (C) Table shows sequence identity matrix (calculated in percentage) of indicated proteins, generated using the Clustal Omega software. *$p \leq 0.05$ (estimated via unpaired two-tailed Student's t-test).

Supplementary Figure 2: PDK4 expression in Parkinson's Disease (PD) patient sample and cellular prion disease models

(A) Whole brain protein lysates of control and PD patient were immunoblotted to check expression of PDK4. (B) Graph with data from panel A represents altered PDK4 protein levels, mean ± SEM of 3 technical replicates. (C) Immunoblotting of SH-SY5Y cell lysates, transfected with indicated constructs were subjected to immunoblotting for detecting protein expression of different proteins. (D) Graphs represent change in expression of different proteins, as mentioned in C. Data represent the mean ± SEM of 3 independent experiments. *$p \leq 0.05$; ns: not significant (estimated via unpaired two-tailed Student's t-test; one-way ANOVA with Bonferroni's corrections).

Supplementary Figure 3: Thapsigargin mediated effect on ER-mitochondria contact sites

(A) Lysates generated from U2OS cells with or without treatment with TG (Thapsigargin, 0.5µM for 3 hrs). Altered protein levels analysed as mean ± SEM of 3 independent experiments. (B) SH-SY5Y cells were transfected with KDEL-GFP, treated with DMSO or

TG (0.5μM for 3 hr), followed by staining with MitoTracker Red FM. Cells were fixed and immune-stained to detect HSP60. Co-localization of KDEL-GFP (ER), MitoTracker Red FM (mitochondria) and HSP60 signals was detected in white. Overlapped regions are marked with red arrowheads (black and white insets), and white arrowheads for insets in color. Scale bar: 10 μm. (C) Graph shows quantification of KDEL-GFP, MitoTracker Red FM and HSP60 overlap (with binarized data from panel B); overlap index represented as mean fold change ± SEM. Data was calculated from ~60 cells from 3 independent experiments. (D) Cells similarly treated as in panel B, except immuno-staining was against MFN2. Co-localization of KDEL-GFP (ER), MitoTracker Red FM (mitochondria) and MFN2 signals was detected in white. Overlapped regions are marked with red arrowheads (black and white insets), and white arrowheads for insets in color. Scale bar: 10 μm. (E) Graph shows quantification of KDEL-GFP, MitoTracker Red FM and MFN2 overlap (with binarized data from panel D); overlap index represented as mean fold change ± SEM. Data was calculated from ~55 cells from 3 independent experiments. (F) Flowchart shows the sequential steps of isolating different cellular fractions from the whole cell lysate [nuclei, cytosol (cyto), mitochondria (mito), MAM and ER]. SH-SY5Y cells were subjected to treatment with either DMSO or TG (0.5μM for 3 hrs) prior to the fractionation process. (G) Cells treated with TG or DMSO, imaged under live-cell conditions using MitoTracker Red FM. Enlarged views of the areas within the white boxes are shown (inset). Scale bar: 5 μm. (H) Violin plot of the mitochondrial length (μm) with data from panel G. Solid and dashed black lines mark median and quartiles respectively. *$p \leq 0.05$; ***$p \leq 0.001$; ns: not significant (estimated via unpaired two-tailed Student's t-test).

Supplementary Figure 4: Effect of PDK4 over-expression on other PDK isoforms

(A) SH-SY5Y cell lysates from empty vector (EmpVec), WT PDK4 transfected samples were immunoblotted to detect expression of indicated proteins. ◄exogenous (PDK4-RFP),

◄endogenous (B) Graph with data from panel A represents change in expression of indicated proteins. Data represent the mean ± SEM of 3 independent experiments. (C) Different cellular fractions obtained from DMSO and TG (0.5µM for 3 hrs) treated SH-SY5Y cells were subjected to immunoblotting to check the expression of the indicated proteins. MFN2 was used as mitochondrial and MAM marker; whereas calnexin and vinculin were used as ER and cytosolic markers, respectively. WCL: Whole cell lysate, Cyto: Cytosol, Mito: Mitochondria. *$p \leq 0.05$; ns: not significant (estimated via unpaired two-tailed Student's t-test).

Supplementary Figure 5: Effect of DCA on ER and mitochondria

(A) SH-SY5Y cell lysates from DCA or DMSO treated samples were immunoblotted against the indicated proteins. (B) and (C) Graphs represent change in expression of proteins analysed in A. p-PDHA1 was normalized to total PDHA1 level. Data represent mean ± SEM of 3 independent experiments. (D) Cells treated with DCA or DMSO were imaged under live-cell conditions using MitoTracker Green FM. Enlarged views of the areas within the white boxes are shown (inset). Scale bar: 5 µm. (E) Violin plot of the mitochondrial length (µm) with data from panel D. Solid and dashed black lines mark median and quartiles respectively. (F) Pie-charts show categorical representation of total mitochondrial pool depending on their lengths (<2.5 µm fragmented, 2.5- 5 µm intermediate and >5 µm filamentous) with data from panel D. *$p \leq 0.05$; **$p \leq 0.01$; ***$p \leq 0.001$ (estimated via unpaired two-tailed Student's t-test).

Supplementary Figure 6: Effect of PDK4 over-expression on mitochondrial activity

(A) qRT-PCR analyses show non-significant changes of indicated OXPHOS mRNA levels, in control (EmpVec) and PDK4 transfected SH-SY5Y cells. Data represent the mean ± SEM of 3 independent experiments. (B) SH-SY5Y cells transfected with control or PDK4 vectors were loaded with TMRM and MitoTracker Green FM, and imaged under live cell conditions. CCCP (10 µM) and oligomycin (1 µM) used as controls. (C) Graph with data from panel B

shows quantification of TMRM signal intensity. (D) Graph shows quantification of MitoTracker Green FM signal intensity with data from panel B. (E) Graph shows ratio of TMRM and MitoTracker Green signal intensity as obtained in panels C and D. Data was calculated from ~150 cells from 3 independent experiments. *$p \leq 0.05$; **$p \leq 0.01$; ***$p \leq 0.001$; ns: not significant (estimated via unpaired two-tailed Student's t-test, one-way ANOVA with Tukey's corrections).

Supplementary Figure 7: Effect of exogenous PDK4 on autophagy. (A) SH-SY5Y cells transfected with the indicated constructs were either treated with bafilomycin A1 or left untreated. Lysates from these samples were immunoblotted against the indicated proteins. ◄exogenous (PDK4-RFP), ➤endogenous. (B) Graph represents change in LC3-II/LC3-I with data from A; mean ± SEM of 3 independent experiments. (C) Histogram plotting change in P62 expression with data from panel A; mean ± SEM of 3 independent experiments. (D) SH-SY5Y cell transfected with the indicated constructs were either treated with rapamycin or left untreated. Lysates from these samples were immunoblotted against the indicated proteins. ◄exogenous (PDK4-RFP), ➤endogenous. (E) Histogram plots change in LC3-II/LC3-I with data from D; mean ± SEM of 3 independent experiments. (F) Graph represents change in P62 expression with data from panel D; mean ± SEM of 3 independent experiments. (G) SH-SY5Y cells co-transfected with GFP-LC3, along with PDK4 construct or control (EmpVec), were loaded with MitoTracker Deep Red FM and imaged live. Enlarged views of the areas within the white boxes are shown (inset). GFP-LC3 vesicles positive for mitochondria are marked with white arrowheads. Scale bar: 5 µm. (H) Graph with data from panel G plots the number of GFP-LC3 positive vesicle positive for MitoTracker Deep Red signal. Data represents mean puncta number/cell ± SEM obtained from ~20 cells from 3 independent experiments. (I) SH-SY5Y cells co-transfected with CD63-YFP, along with PDK4 construct or control (EmpVec), staining with MitoTracker Deep Red FM and imaged live. Enlarged

views of the areas within the white boxes are shown (inset). CD63-YFP vesicles positive for mitochondria are marked with white arrowheads. Scale bar: 5 µm. (J) Graph with data from panel I plots the number of CD63-YFP puncta positive for MitoTracker Deep Red signal. Data represents mean puncta number/cell ± SEM calculated from ~20 cells from 3 independent experiments. *p≤0.05; **p≤0.01; ***p≤0.001; ns: not significant (estimated via unpaired two-tailed Student's t-test, one-way ANOVA with Tukey's corrections).

Supplementary Figure 8: Verification of used PDK4 antibody specificity

SH-SY5Y cells were transfected with the indicated constructs, or treated with DCA or its vehicle control (DMSO). Cell lysates were immunoblotted against the indicated proteins. Note distinct reduction in levels of PDK4 and PDK4-RFP in knockdown and DCA treated samples, while the levels of the non-specific bands remained comparable across the samples (treated and controls). ◀ exogenous (PDK4-RFP), ➤endogenous, n.s: non-specific bands.

Supplementary Figure1

A B

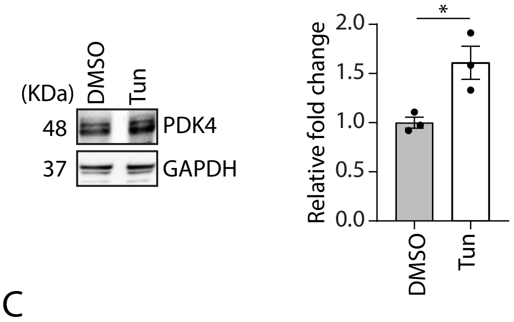

C

Sequence identity matrix (percentage)

	PDK4	PDK3	PDK1	PDK2
PDK4	100.00	62.59	64.72	64.52
PDK3	62.59	100.00	67.33	67.00
PDK1	64.72	67.33	100.00	68.06
PDK2	64.52	67.00	68.06	100.00

Supplementary Figure2

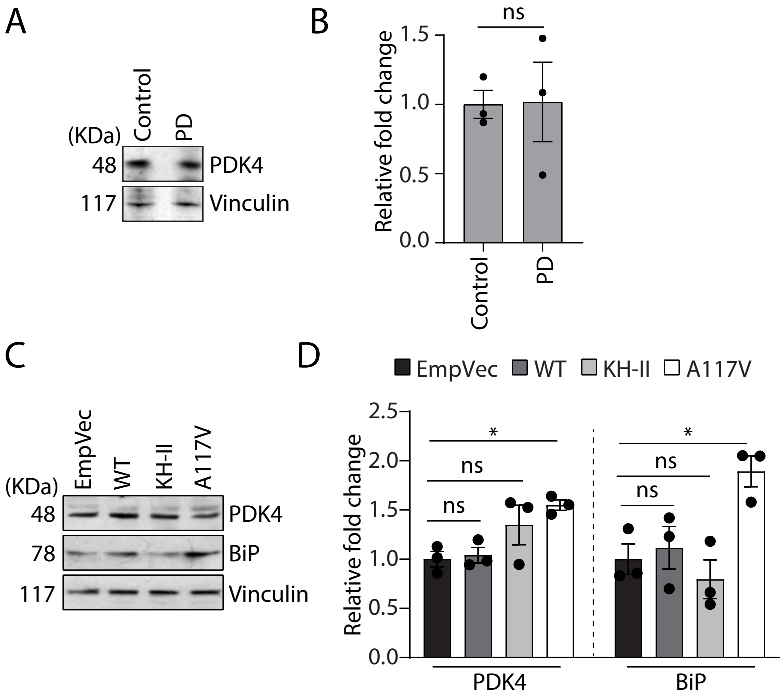

Supplementary Figure3

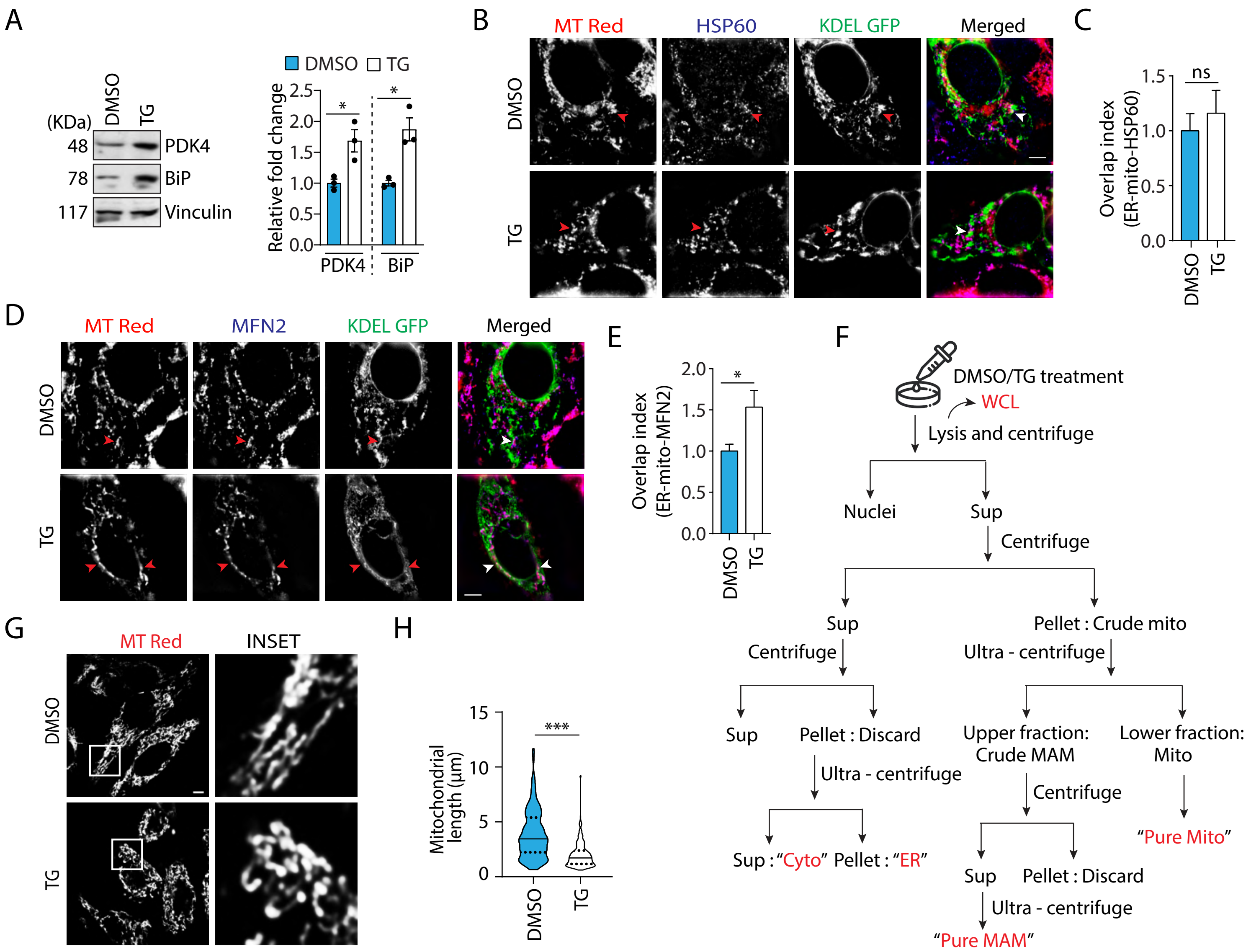

Supplementary Figure4

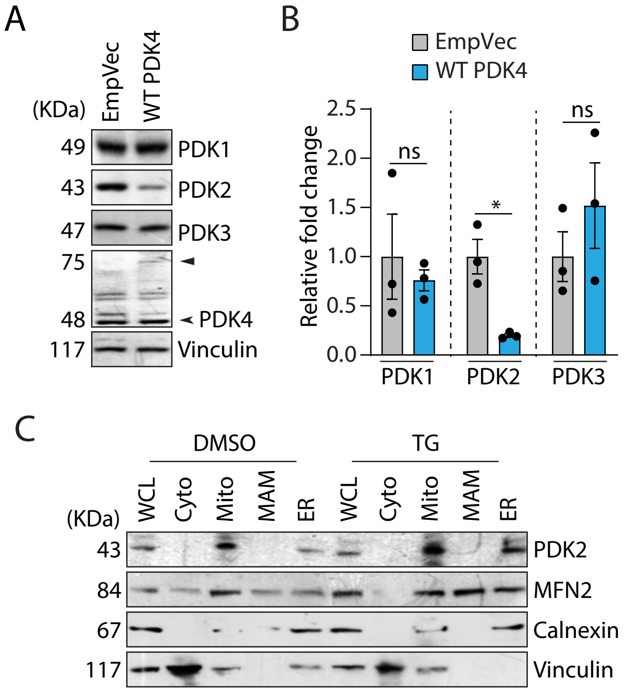

Supplementary Figure5

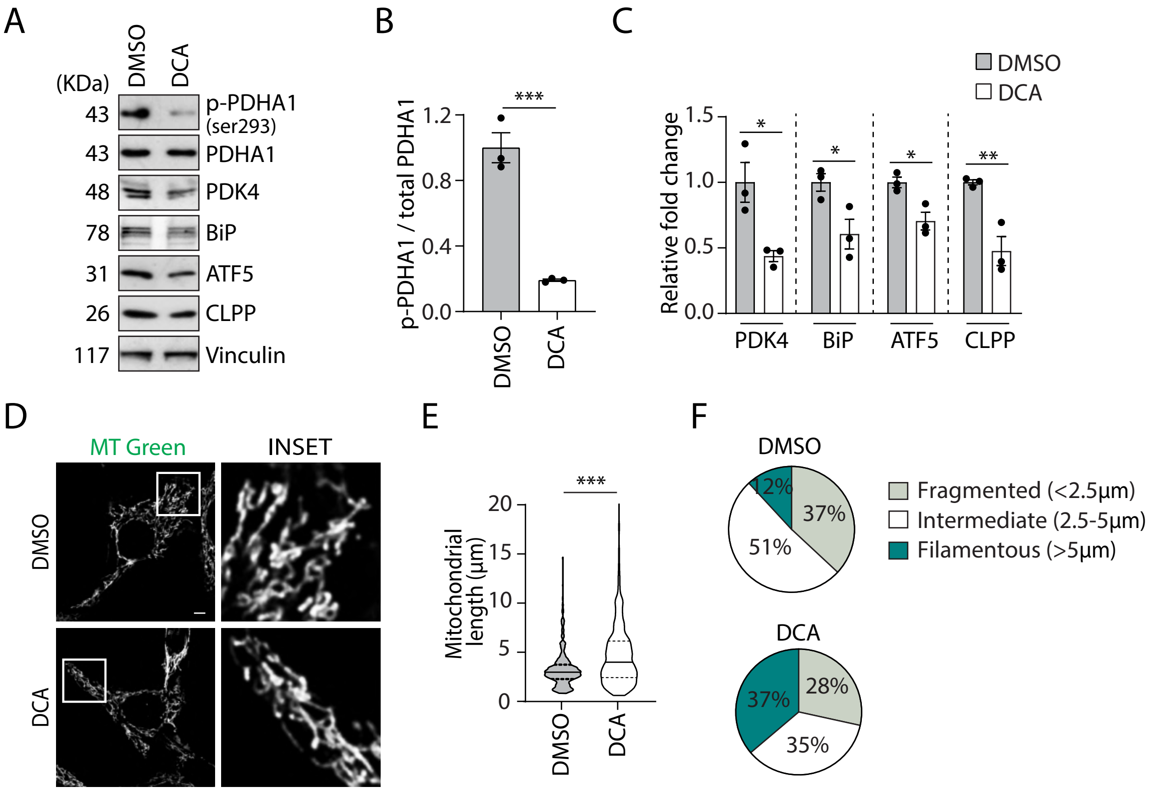

Supplementary Figure6

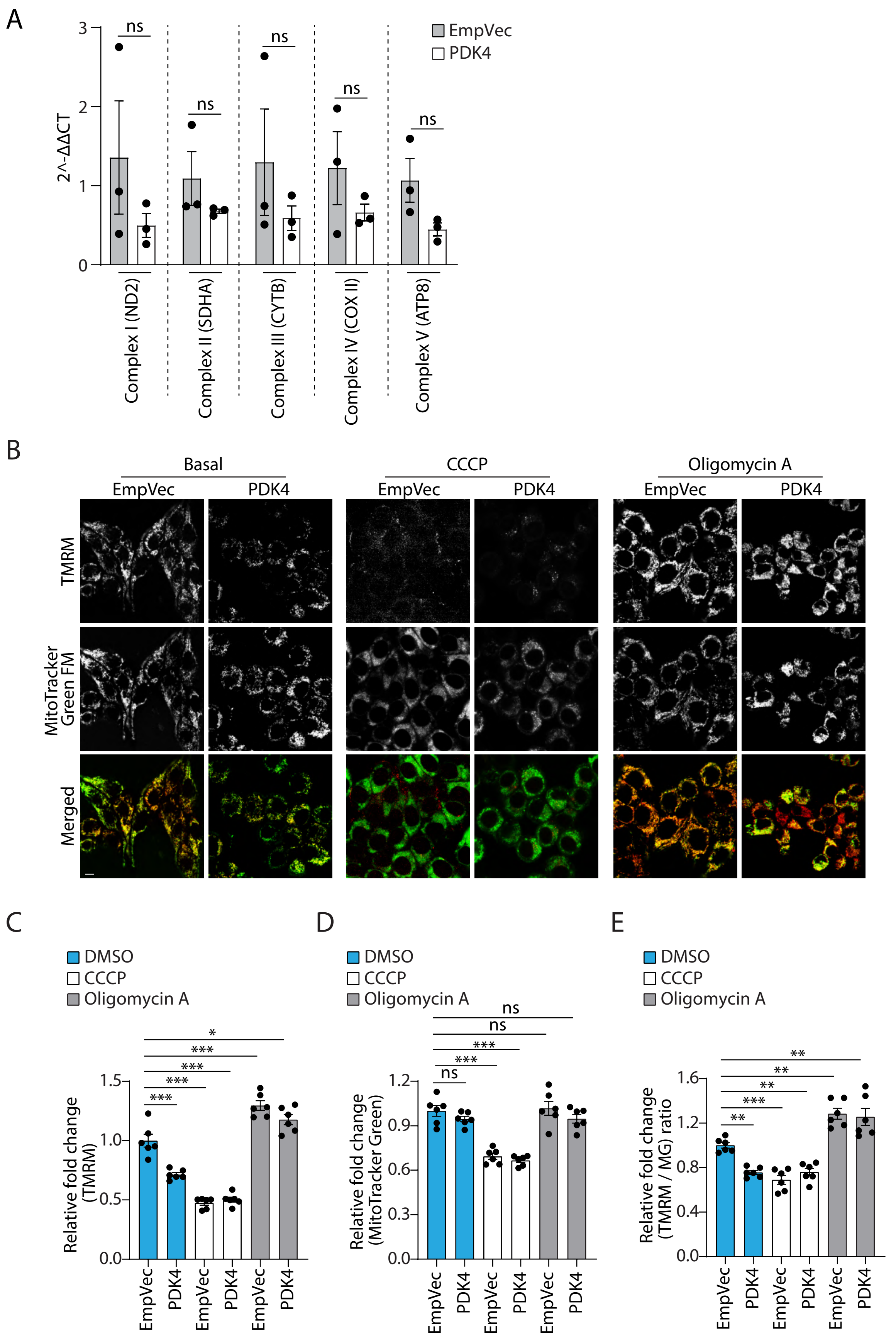

Supplementary Figure7

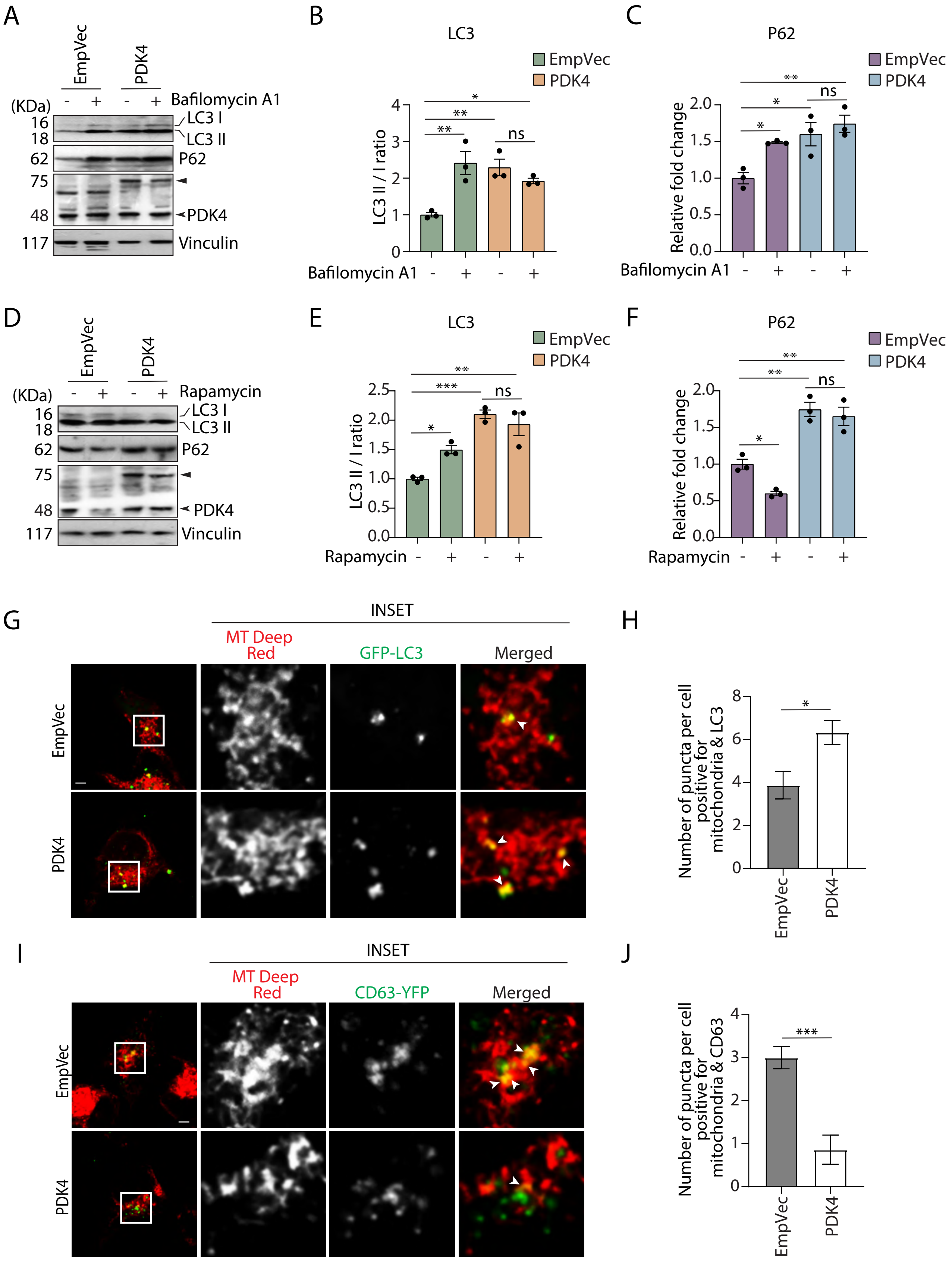

Supplementary Figure8

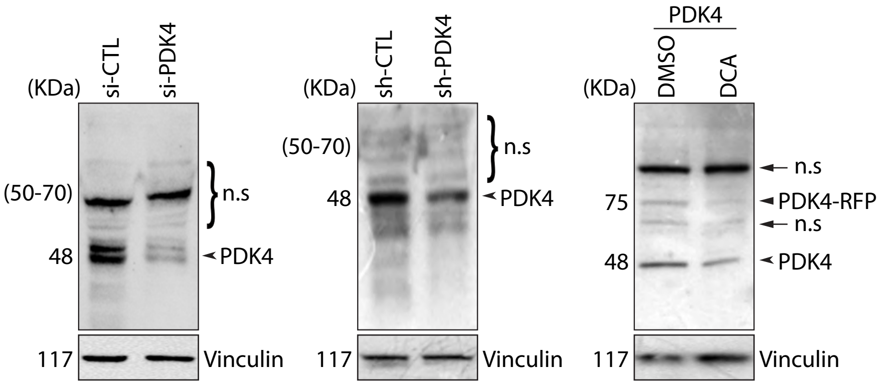

Supplement: Supplementary file 1 — Supplementary materials [file 41419_2025_7743_MOESM1_ESM.pdf]
