# Supplementary material for: The impact of ERUPR on mitochondrial integrity mediated by PDK4
Source: Cell Death Dis. 2025 Jul 29;16(1):573. doi: 10.1038/s41419-025-07743-5 (PMC12307875; doi:10.1038/s41419-025-07743-5)
Supplement: Supplementary file 2 — Main Figures raw source files [file 41419_2025_7743_MOESM2_ESM.pdf]

Fig1C

2^-ΔΔCT (PDK4)

	DMSO	TG
#1	0.964933	2.782794
#2	0.794657	3.958593
#3	1.580969	1.586007873
#4	0.796896	5.661367247
#5	1.035139	2.412018822

2^-ΔΔCT (BiP)

	DMSO	TG
#1	1.415374	6.122145243
#2	0.345536	5.052188143
#3	2.044727	3.537937019

2^-ΔΔCT (XBP1-S)

	DMSO	TG
#1	1.106017	14.93275
#2	1.073541	20.18096
#3	2.179081	15.73538524
#4	0.564977	24.93783752
#5	0.684093	27.02578207

2^-ΔΔCT (CHOP)

	DMSO	TG
#1	0.949088	4.429745
#2	1.930926	5.212186
#3	0.545667	3.218489

Fig1D

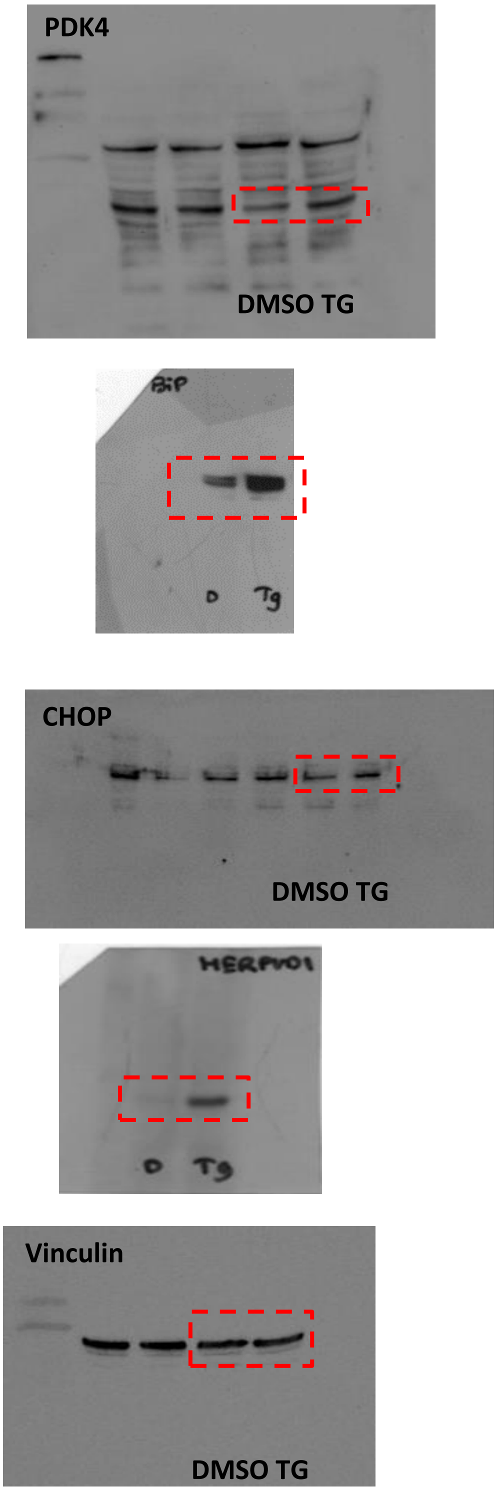

Fig1E

PDK4	DMSO	TG
#1	0.845641	1.638927
#2	0.916618	1.389721
#3	1.183012	1.775527
#4	1.054729	1.733257

BiP	DMSO	TG
#1	0.849448	1.471452
#2	1.248827	2.378619
#3	0.731644	1.892676
#4	1.170082	2.051089

CHOP	DMSO	TG
#1	1.056327	1.479651
#2	0.958425	1.31859
#3	1.093577	1.454196
#4	0.891671	1.490997

HERPUD1	DMSO	TG
#1	1.53872	11.96637
#2	0.966761	10.25094
#3	0.935735	7.703326
#4	0.558784	5.83324

Fig1F

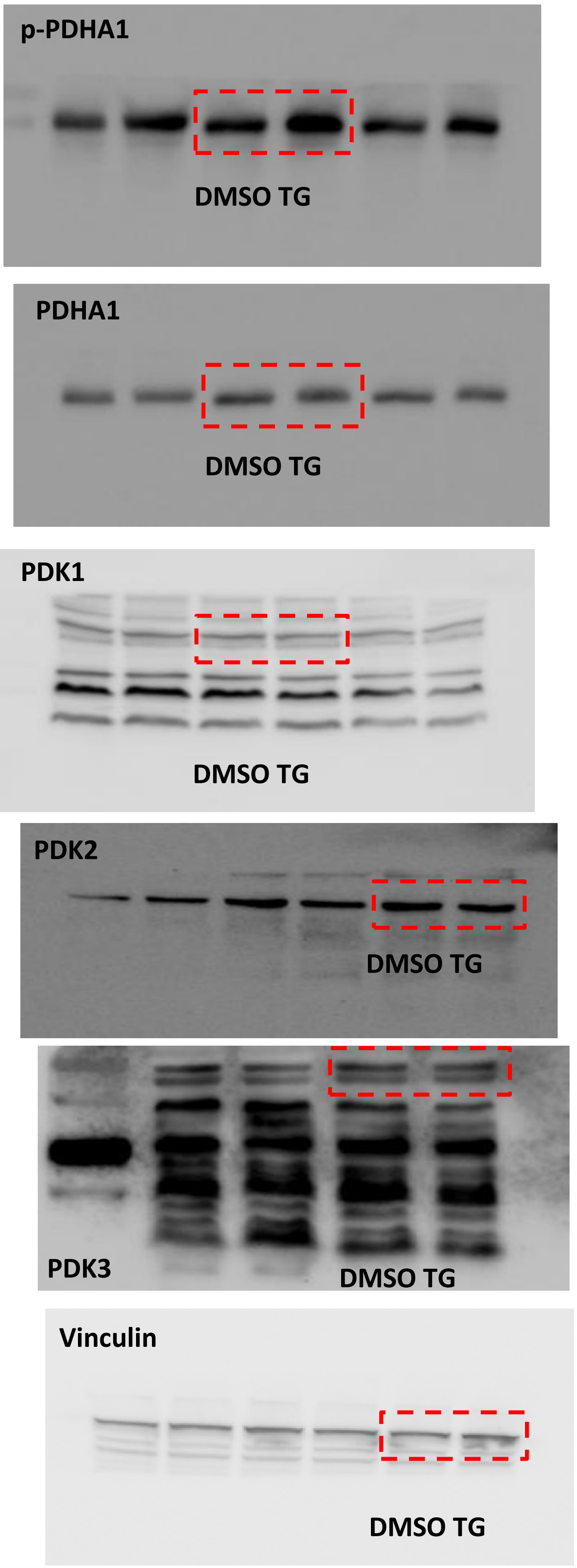

Fig1G

**p-PDHA1/PDHA**	**DMSO**	**TG**
#1	1.202496	1.446897
#2	1.133296	1.326777
#3	0.877875	1.291863
#4	0.884991	1.116974
#5	0.901343	1.221204

Fig1H

**PDK1**	**DMSO**	**TG**
#1	0.975896	0.974639
#2	1.356146	1.101694
#3	0.933597	0.577891
#4	1.026475	0.719835
#5	0.811892	1.093149
#6	0.895994	1.40171

**PDK2**	**DMSO**	**TG**
#1	0.940795	1.635201
#2	2.05527	1.803974
#3	2.083634	1.22235
#4	0.348269	0.234608
#5	0.327751	0.270842
#6	0.244281	0.241867

**PDK3**	**DMSO**	**TG**
#1	1.808316	1.444151
#2	1.322256	1.188432
#3	2.047373	2.069011
#4	0.346198	0.310532
#5	0.312423	0.230556
#6	0.163433	0.243899

Fig2A

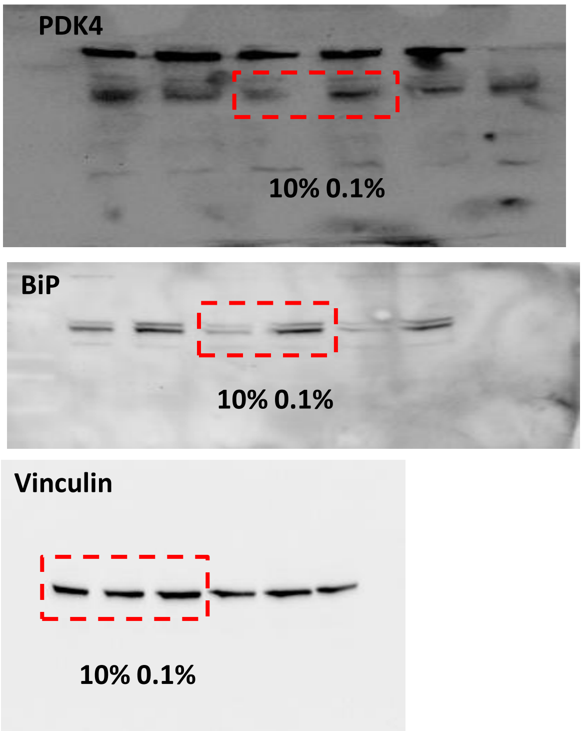

Fig2B

**PDK4**	**10%FBS**	**0.1%FBS**
#1	0.951772	3.39225
#2	0.905959	2.344185
#3	1.125671	2.902829
#4	1.016598	1.35925

**BiP**	**10%FBS**	**0.1%FBS**
#1	0.835734	1.721931
#2	0.946642	1.628524
#3	0.983805	1.767329
#4	1.233819	2.863482

Fig2C

Hippocampus

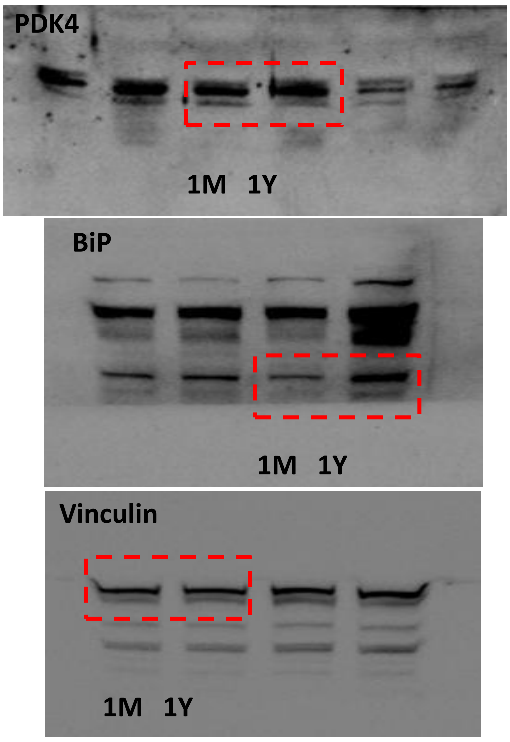

Cortex

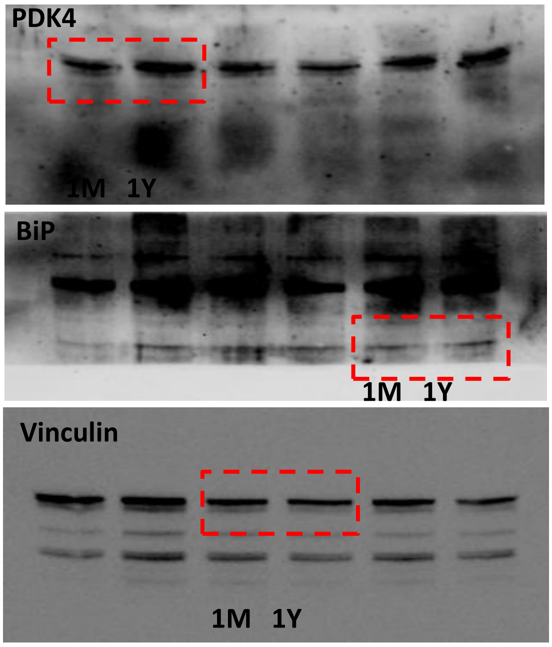

Fig2D

Hippocampus

**PDK4**	**1M**	**1Y**
#1	1.164523	1.271341
#2	1.092816	1.502197
#3	0.613394	1.581617
#4	0.485114	1.621933
#5	1.644153	1.677706
**BiP**	**1M**	**1Y**
#1	1.068533	2.114031
#2	1.101531	2.500112
#3	1.03174	1.889429
#4	1.149282	1.389906
#5	0.648914	1.555092

Cortex

**PDK4**	**1M**	**1Y**
#1	1.439946	1.642086
#2	0.863655	0.267003
#3	0.920862	1.114112
#4	0.82362	0.430418
#5	0.951917	1.237352

Striatum

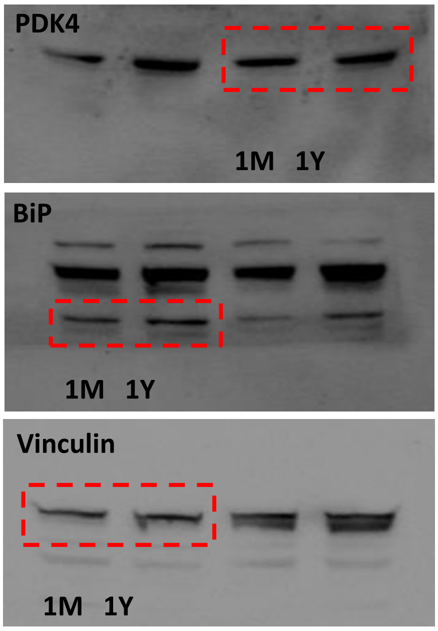

Cerebellum

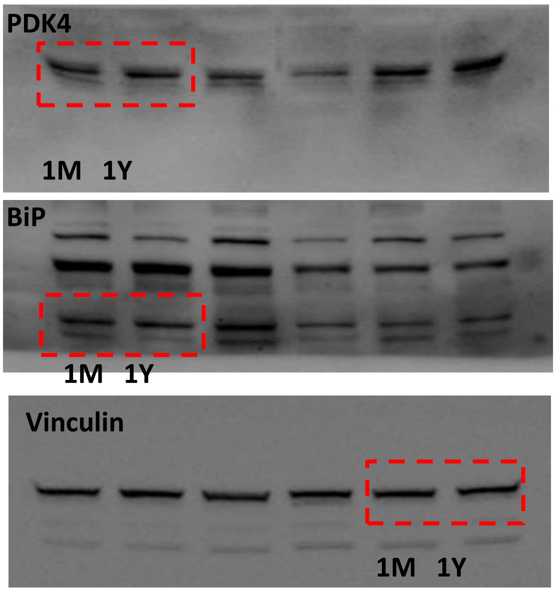

Fig3A

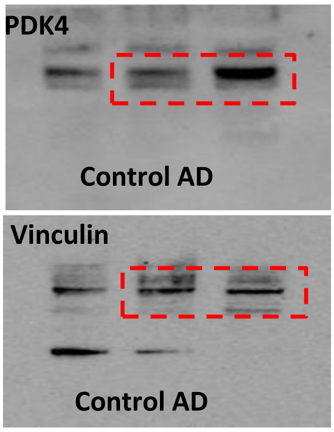

Striatum

PDK4	1M	1Y
#1	0.381241	0.297618
#2	0.286056	0.188034
#3	1.392238	1.705299
#4	1.557265	2.684797
#5	1.3832	0.983692
**BiP**	**1M**	**1Y**
#1	0.925601	0.949682
#2	1.31477	1.326796
#3	0.638658	1.411178
#4	1.41249	1.677559
#5	0.708482	2.112104

Cerebellum

PDK4	1M	1Y
#1	1.062972	1.129182
#2	0.791457	0.567002
#3	0.97947	1.405758
#4	1.540699	1.273624
#5	0.625401	0.620532

BiP	1M	1Y
#1	1.235571	0.962137
#2	1.834253	0.551973
#3	0.861338	0.655716
#4	0.686253	0.658985
#5	0.382586	1.362351

Fig3B

PDK4	Control	AD
#1	0.932068	1.425852
#2	1.198942	1.572079
#3	0.86899	1.377927

Fig3C

GSE48350		GSE5281	
**Control**	**AD**	**Control**	**AD**
9.574877296	9.927012467	7.4	7.35
10.36565839	8.660344334	4.98	7.75
9.031647203	10.43132555	5.43	6.07
9.263301896	9.153364025	5.47	7.21
9.274074698	10.54866884	5.82	6.06
9.76990972	10.28148669	3.89	5.79
10.18332161	8.604256902	5.01	6.64
7.146098837	9.322438235	4.23	7.39
7.551361601	10.33647907	4.15	6.91
7.16721895	7.814114389	3.98	7.49
7.707492999	10.81010718	5.97	6.38
8.814176752	9.32371951	4.29	7.5
9.854070367	9.495605548	5.55	8.08
8.351249283	7.701651504	5.92	4.74
9.182459982	9.31809866	5.89	6.79
8.660344334	9.823355147	8.66	5.97
8.606391182	10.81954267	7.03	6.01
8.169650958	9.556988515	6.65	5.37
9.681714284	10.13390053	7.02	4.69
10.1264859	8.606391182	6.55	8.2
10.15288857	10.17717177	4.97	9.69
8.449156667	10.41943762	5.95	8.54
10.41796378	9.697199618	4.97	8.74
10.76771662	9.476479829	4.51	7.88
7.761197587	9.884782863	6.58	7.54
8.135676774	9.948957084	7.74	9.45
10.1518965	9.354759494	6.26	8.74
9.24146894	9.745173587	5.13	7.33
9.636776006	11.23614601	5.28	6.22
8.181583525	8.58511995	4.03	8.62
7.512302058	9.41814715	5.97	8.65
7.538405766	9.269449491	4.63	9.77
8.53576572	10.26012862	4.88	9.5
9.841937046	8.71653246	4.2	8.33
9.511711492	10.21461853	7.63	7.19
9.72518676	10.62965575	4.52	9.29
9.375292569	8.194420869	6.88	8.26
8.118633015	8.169650958	7.92	5.68
8.305804353	6.117400328	6.48	5.92
7.859578112	10.86025712	6.26	5.18
8.277064393	8.09468408	7.19	5.13
9.378351914	9.80622379	5.77	4.18
8.164046529	9.439740627	6.73	6.24
8.006261369	8.896817344	8.64	7.57
6.907020657	9.918896908	4.29	3.94
10.94012701	7.99824708	6.45	6.96

8.512353341	8.830092871	7.03	8.43
9.386094612	9.403772337	8.04	8.32
9.783207896	9.625306981	6.71	7.54
9.066258254	8.826693699	7.13	7.75
9.226841238	9.21886939	8.25	7.95
7.236091679	9.399547734	6.31	9
10.05055371	7.757818032	4.75	9.11
8.883718326	9.871765758	5.28	5.92
7.790684744	9.133531599	6.83	7.95
8.545759431	7.793724216	5.07	8.48
10.14683682	9.395965243	5.63	6.84
8.346025881	8.440635064	5.88	7.54
8.729089516	8.15081554	7.46	8.14
9.579210864	10.96332796	6.56	7.9
9.469587285	8.778755276	7.72	7.72
10.56178272	8.218998198	3.88	6.88
9.576245797	9.961823165	6.83	7.89
9.050477134	8.129242849	7.01	7.86
10.08875254	9.204038754	5.81	9.41
9.875363518	9.909886825	6.74	7.89
8.446550818	9.559167176	9.8	9.37
9.786194187	9.035285074	5.19	8.37
9.466511539	9.21494076	6.84	8.6
9.174298136	9.18965544	7.09	8.86
8.981185027	8.728025778	8.82	7.41
10.21106097	9.408344174	7.29	6.45
10.54312438	9.485896686	9.34	6.07
9.300820457	8.893681876	8.19	8.41
9.510375058	9.884698379		8.04
7.785489064	9.577079628		3.81
10.23637306	10.44269943		6.08
7.272960069	9.85528156		5.98
7.913655231	8.077939043		4.78
7.226634504	8.484941349		8.04
7.468498348			8.02
10.88653493			7.96
10.4024986			6.64
9.249194625			8.24
9.369406531			7.1
9.758060379			8.33
7.317152586			8.88
6.698609354			
9.829509649			
9.320984927			
5.841284252			
6.152764461			
5.650345219			
8.935124524			
8.746388015			
7.601184484			

8.061476864
7.636083476
6.93589935
8.793480852
9.247814098
8.40457518
8.305169342
7.591644478
8.511108041
8.26030317
7.271760026
7.935171169
9.106624982
6.069739223
5.307571951
6.886509662
8.712461416
6.901029407
6.021241457
9.986599729
10.1380124
10.04041829
9.596307327
8.910702534
10.42422362
6.84244538
9.041395883
6.463376488
6.058990099
7.283670963
7.050518831
6.7482722
6.220526867
6.02903715
7.167167041
7.022827706
9.738548218
11.90245431
8.633664151
8.50164345
7.442008254
9.902146744
8.080076876
8.029204696
8.179446452
7.432917402
7.299545836
8.857533241
8.102068439
7.632664385

6.811417583
6.50878988
6.654110311
7.109201462
5.72849425
6.981271456
8.019874057
9.115480451
7.377764231
8.635047531
9.890354695
10.08944398
10.7578321
9.664383437
8.611173837
8.55616942
7.364841815
8.021117882
9.501971732
9.314595824
9.250571991
10.3783247
10.33469204
10.72340583
10.59339732
9.993115407
8.218998198

Fig3D

Hippocampus

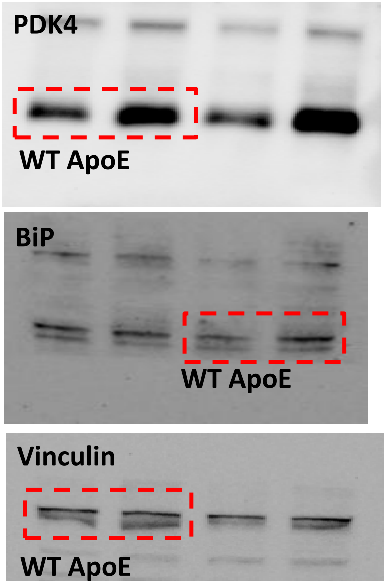

Cortex

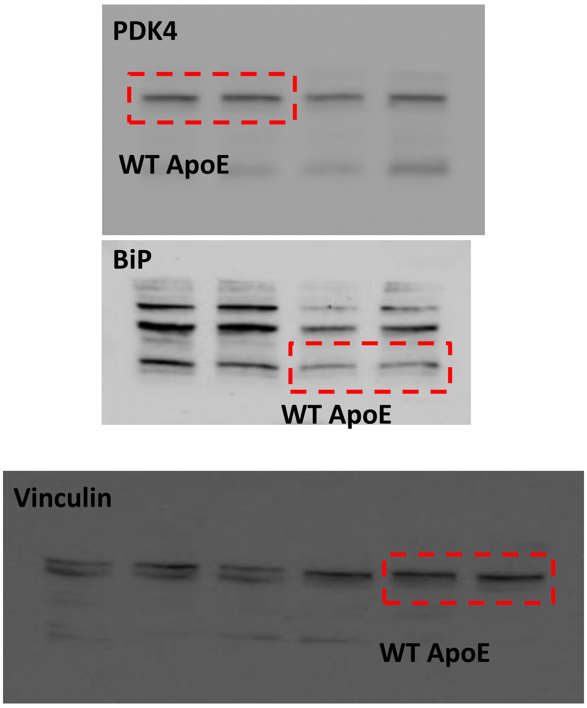

Fig3E

Hippocampus

PDK4	WT	ApoE KO
#1	0.874331	1.296185
#2	1.009136	1.183051
#3	1.082261	1.288293
#4	1.034272	1.096488

BiP	WT	ApoE KO
#1	0.85969	1.327107
#2	0.772147	1.264199
#3	0.94178	1.580202
#4	1.426382	1.754941

Cortex

PDK4	WT	ApoE KO
#1	0.84484	0.776974
#2	0.79067	0.914353
#3	0.923053	0.980309
#4	1.441437	1.417732

Striatum

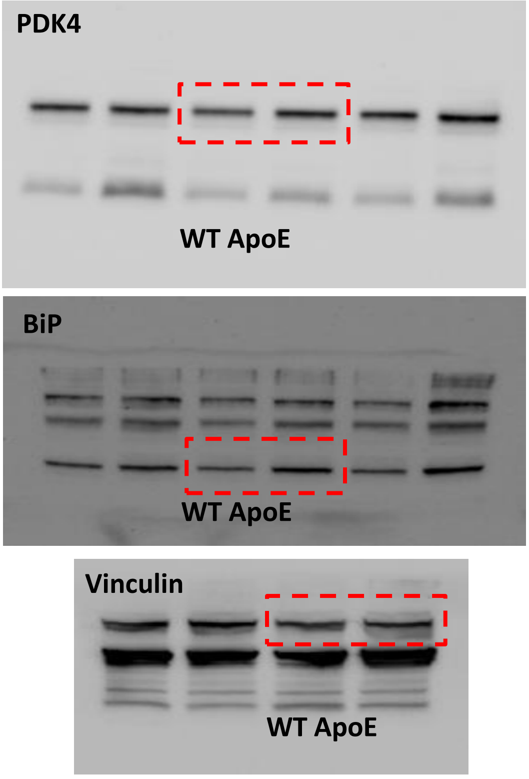

Cerebellum

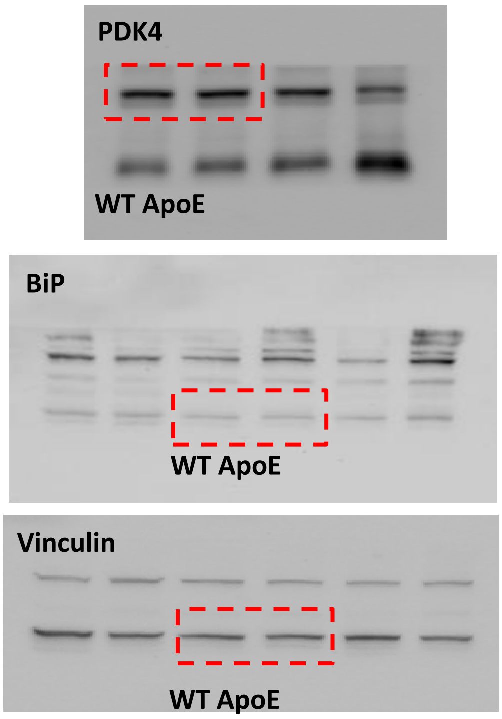

Striatum

**PDK4**	**WT**	**ApoE KO**
#1	1.249552	1.252551
#2	0.840095	1.030901
#3	1.037717	1.614883
#4	0.872636	1.159371

**BiP**	**WT**	**ApoE KO**
#1	1.327189	1.662433
#2	0.949899	1.640726
#3	1.062773	2.137181
#4	0.660138	1.070758

Cerebellum

**PDK4**	**WT**	**ApoE KO**
#1	0.549828	0.472559
#2	0.635759	0.751133
#3	0.798593	0.819154
#4	2.01582	1.662822

**BiP**	**WT**	**ApoE KO**
#1	1.159866	0.71678
#2	0.631099	0.890464
#3	0.887466	1.175669
#4	1.321569	1.453941

Fig3F

Hippocampus

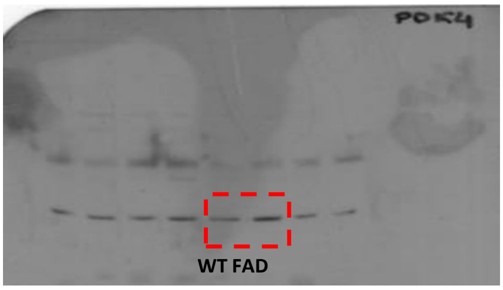

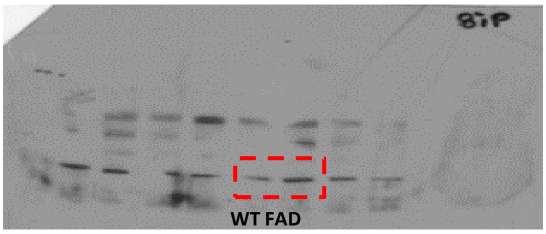

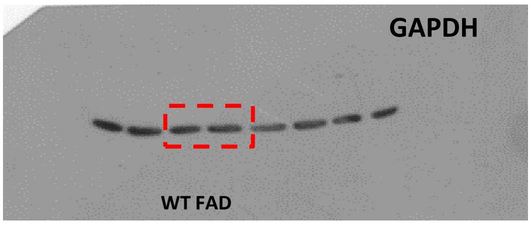

Cortex

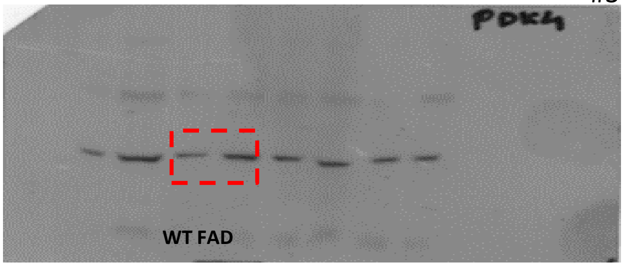

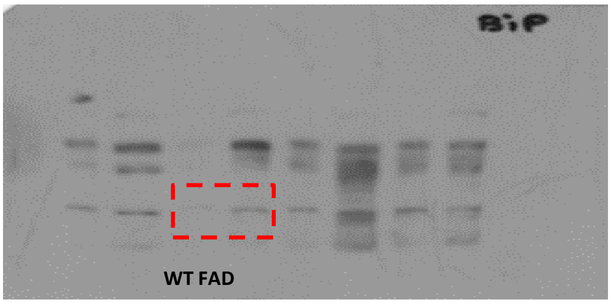

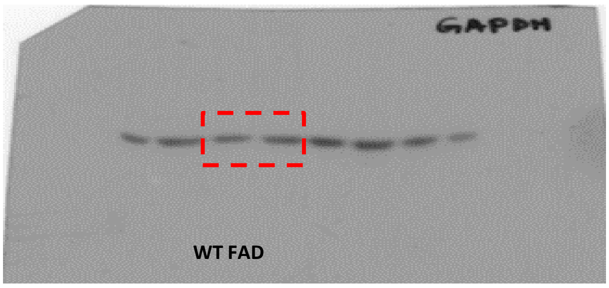

Fig3G

Hippocampus

**PDK4**	**WT**	**5x FAD**
#1	1.034234	1.59599
#2	1.030785	1.703581
#3	0.93498	2.105959

**BiP**	**WT**	**5x FAD**
#1	1.087222	1.464474
#2	1.107009	1.497434
#3	0.805769	1.682638

Cortex

**PDK4**	**WT**	**5x FAD**
#1	1.059982	2.117141
#2	0.990554	1.825473
#3	0.949464	1.989924

**BiP**	**WT**	**5x FAD**
#1	1.107681	1.803505
#2	0.700042	1.571359
#3	1.192277	1.644201

Striatum

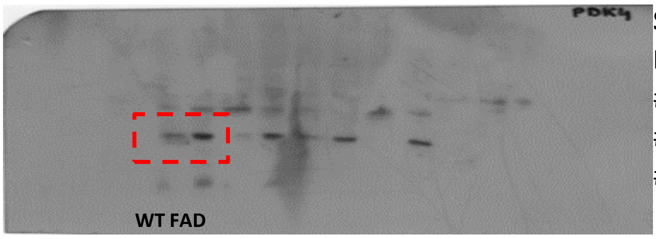

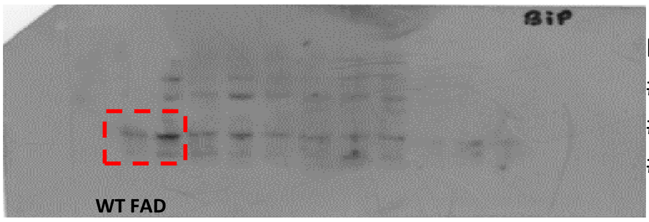

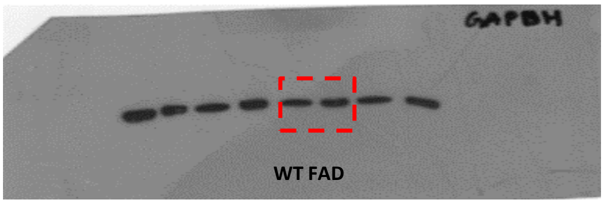

Cerebellum

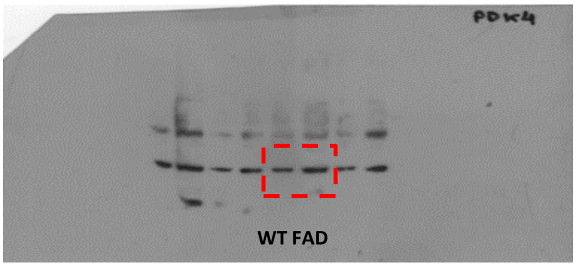

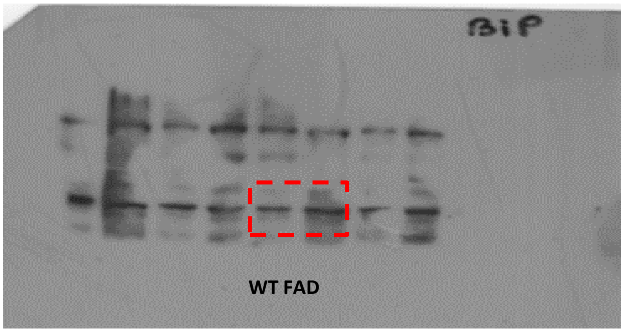

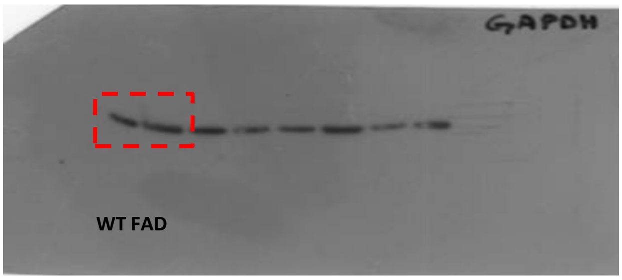

Striatum

PDK4	WT	5x FAD
#1	1.02583	3.251785
#2	0.404044	1.923123
#3	1.570126	2.939198

BiP	WT	5x FAD
#1	0.948793	2.172344
#2	0.966192	2.001036
#3	1.085015	1.551893

Cerebellum

PDK4	WT	5x FAD
#1	0.892202	1.966415
#2	1.190266	1.494592
#3	0.917532	1.548523

BiP	WT	5x FAD
#1	0.745595	1.534627
#2	1.218324	1.629037
#3	1.036081	1.595362

Fig3H

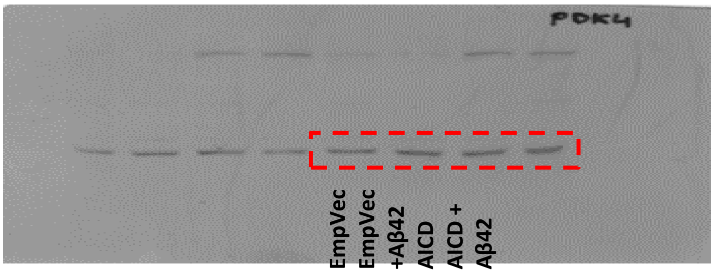

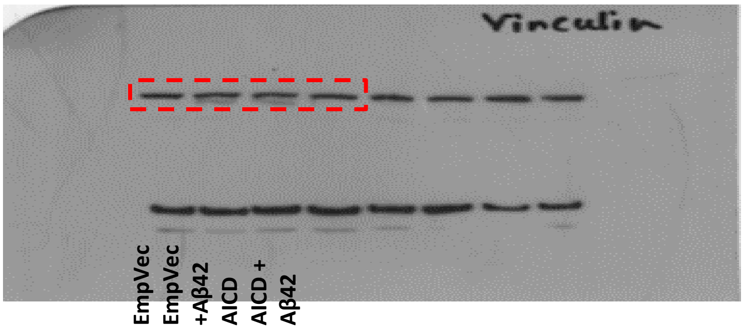

Fig3I

PDK4	EmpVec	EmpVec+Aβ42	AICD	AICD+Aβ42
#1	1.014852	1.570772319	1.685109	1.56875
#2	1.025479	1.329043015	1.781417	1.940991
#3	0.959669	1.517151896	1.456855	1.488756

Fig4C

Overlap index ER-Mito	
**DMSO**	**TG**
0.388406998	1.080257
0.308298055	1.388555
0.27916753	1.759969
0.262174724	1.706563
0.293732793	1.820658
0.24032683	1.750259
0.172355606	1.706563
0.177210693	1.667723
0.182065781	3.257764
0.169928062	4.053998
0.172355606	4.505521
1.082684508	4.408419
1.114242577	4.619616
1.255040114	4.459398
1.405547826	4.102549
1.546345363	7.146689
1.245329939	7.637053

Fig4D

Overlapped pixels ER-Mito-PDK4		Overlap index ER-Mito-PDK4	
**DMSO**	**TG**	**DMSO**	**TG**
83	38	0.821881	0.376283
66	96	0.653544	0.95061
68	169	0.673349	1.673469
52	185	0.514914	1.831904
68	148	0.673349	1.465523
55	166	0.54462	1.643763
38	150	0.376283	1.485328
38	174	0.376283	1.72298
33	285	0.326772	2.822123
43	363	0.425794	3.594493
37	276	0.366381	2.733003
192	245	1.90122	2.426036
178	263	1.762589	2.604275
165	218	1.633861	2.158676
142	183	1.40611	1.8121
134	362	1.326893	3.584591
145	341	1.435817	3.376645

0.956452234	8.258504	132	367	1.307089	3.634102
0.888481009	9.30963	97	355	0.960512	3.515276
0.956452234	9.423725	97	344	0.960512	3.406352
0.830219959	8.930933	83	315	0.821881	3.119189
0.873915746	8.964919	133	267	1.316991	2.643884
0.791379259	1.604606	106	256	1.049632	2.53496
0.67485716	1.441961	96	229	0.95061	2.267601
0.463660854	1.616744	58	226	0.574327	2.237894
0.451523136	1.619172	54	229	0.534718	2.267601
0.335001036	1.560911	33	204	0.326772	2.020046
0.35684893	1.458954	34	197	0.336674	1.950731
1.691997987	1.327866	116	183	1.148654	1.8121
1.548772906	1.276888	99	104	0.980316	1.029827
1.412830457	1.230765	137	106	1.356599	1.049632
1.160365908	1.077829	102	88	1.010023	0.871392
1.053553983	1.179786	101	117	1.000121	1.158556
1.060836614	1.094822	97	99	0.960512	0.980316
1.245329939	1.109387	82	106	0.811979	1.049632
1.167648539	1.121525	58	121	0.574327	1.198164
1.104532402	1.070547	57	125	0.564425	1.237773
1.153083277	1.119098	60	143	0.594131	1.416013
1.121525208	0.927322	40	93	0.396087	0.920903
1.170076083	0.9783	44	112	0.435696	1.109045
0.555907517	0.910329	46	110	0.455501	1.08924
0.677284704	0.995293	65	107	0.643642	1.059534
0.614168566	0.844785	48	93	0.475305	0.920903
0.67485716	0.791379	44	136	0.435696	1.346697
0.648154179	0.827792	32	125	0.31687	1.237773
0.703987685	0.929749	13	155	0.128728	1.534839
0.776813997	0.951597	13	135	0.128728	1.336795
0.820509784	1.068119	15	117	0.148533	1.158556
0.354421386	0.954025	30	94	0.297066	0.930805
0.521921904	1.063264	68	102	0.673349	1.010023
0.602030848	1.361852	77	141	0.762468	1.396208
0.631161372	1.590041	98	165	0.970414	1.633861
0.706415228	1.638592	85	167	0.841686	1.653665
0.803516978	1.556056	98	161	0.970414	1.594252
0.648154179	1.456526	64	184	0.63374	1.822002
0.716125403	1.366707	72	169	0.712957	1.673469
0.835075047	1.157938	80	124	0.792175	1.227871
0.720980491	1.284171	85	135	0.841686	1.336795
0.988010302	1.582915	63	18539	0.623838	2.132145
1.344859232	0.993755	123	14508	1.217969	1.668545
1.296308357	1.042713	138	12355	1.366502	1.420932
1.507504663	1.267488	189	13631	1.871513	1.567683
1.376417301	1.373705	174	15536	1.72298	1.786774
1.441960982	1.782637	150	18598	1.485328	2.13893
1.179786258	1.461132	102	19097	1.010023	2.19632
0.995292933	0.804192	95	3330	0.940708	0.43059
1.597323781	1.128751	68	6949	0.673349	0.89855

1.944462536	0.854665	115	5969	1.138751	0.77183
2.412978478	0.914646	197	6374	1.950731	0.824199
2.546493383	0.961461	212	7789	2.099263	1.007167
2.376565322	2.152681	253	19489	2.505253	2.520052
2.502797596	0.823796	265	7982	2.624079	1.032123
2.459101809	0.719706	280	7009	2.772612	0.906308
2.400840759	1.25695069	300	2086	2.970656	1.98309919
1.255040114	0.97565011	154	1247	1.524937	1.18548643
1.415258001	0.81703413	172	1572	1.703176	1.49445442
1.284170639	1.11743337	170	1638	1.683372	1.55719869
1.274460464	0.73643132	194	739	1.921024	0.70254569
0.968589952	0.91576449	120	1529	1.188262	1.45357558
0.980727671	1.66320181	106	1396	1.049632	1.32713637
0.81808224	0.94586915	64	1092	0.63374	1.03813246
0.85692294		91		0.901099	
0.960103771		7737		0.889822	
0.97548411		8475		0.974698	
1.06583897		5906		0.679241	
0.988826091		8550		0.983324	
1.202075419		14466		1.663715	
0.807671639		7036		0.809201	
1.014494		7939		1.026563	
0.733239		5608		0.72515	
1.201534		9298		1.202291	
0.757012		6076		0.785665	
1.156987		10385		1.342847	
1.168764		7436		0.961522	
0.967971		7393		0.955962	
1.80563249		1992		1.893736	
0.36028486		394		0.374564	
0.26835234		175		0.166367	
0.74161062		571		0.542833	
0.43344243		492		0.46773	
0.799554		822		0.781451	
0.79081394		427		0.405936	
1.64313204		1822		1.732122	
2.15717728		2772		2.635259	

Fig4F

Overlap index	
**ER-Mito**	
**DMSO**	**TG**
1.14614918	1.0924466
1.11252611	1.27518361
1.05093996	1.4647261
0.66929452	1.1908901
0.67468499	1.18368034
1.16703726	1.22734317
1.17936797	1.26211172

Fig4G

Overlapped pixels		Overlap index	
**ER-Mito-PDK4**		**ER-Mito-PDK4**	
**DMSO**	**TG**	**DMSO**	**TG**
3231	5019	0.708864	1.10114085
4983	6820	1.093243	1.49627029
5539	4815	1.215226	1.05638438
5779	6697	1.267881	1.46928477
2799	7339	0.614085	1.61013602
3451	4964	0.75713	1.08907416
6124	6678	1.343572	1.46511628

Fig4H

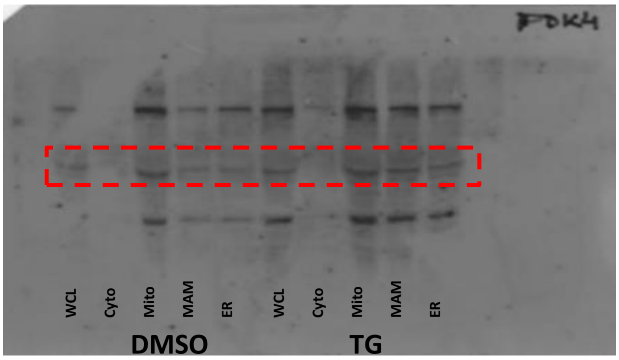

Fig4I

	MAM/WCL ratio	
**PDK4**	**DMSO**	**TG**
#1	1.095474	2.578146
#2	1.116634	3.839312
#3	0.787892	2.563414

	MAM/WCL ratio	
**MFN2**	**DMSO**	**TG**
#1	1.145093	3.109941
#2	0.588775	5.463871
#3	1.266132	4.921119

VDAC1 **DMSO** **TG**

	MAM/WCL ratio	
#1	1.149921	3.441786
#2	0.830808	2.975486
#3	1.019271	4.423358

	MAM/WCL ratio	
**PTDSS1(PSS1)**	**DMSO**	**TG**
#1	0.975295	1.604274
#2	0.79274	1.425631
#3	1.231965	1.961141

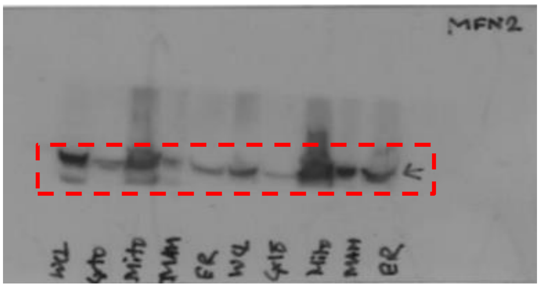

DMSO **TG**

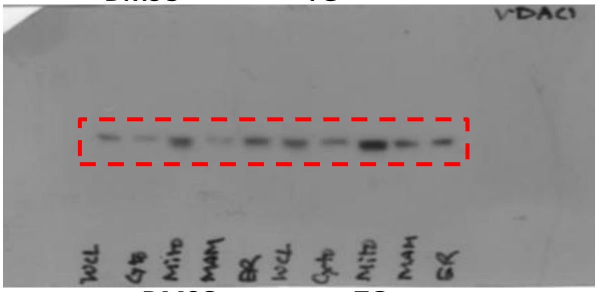

DMSO **TG**

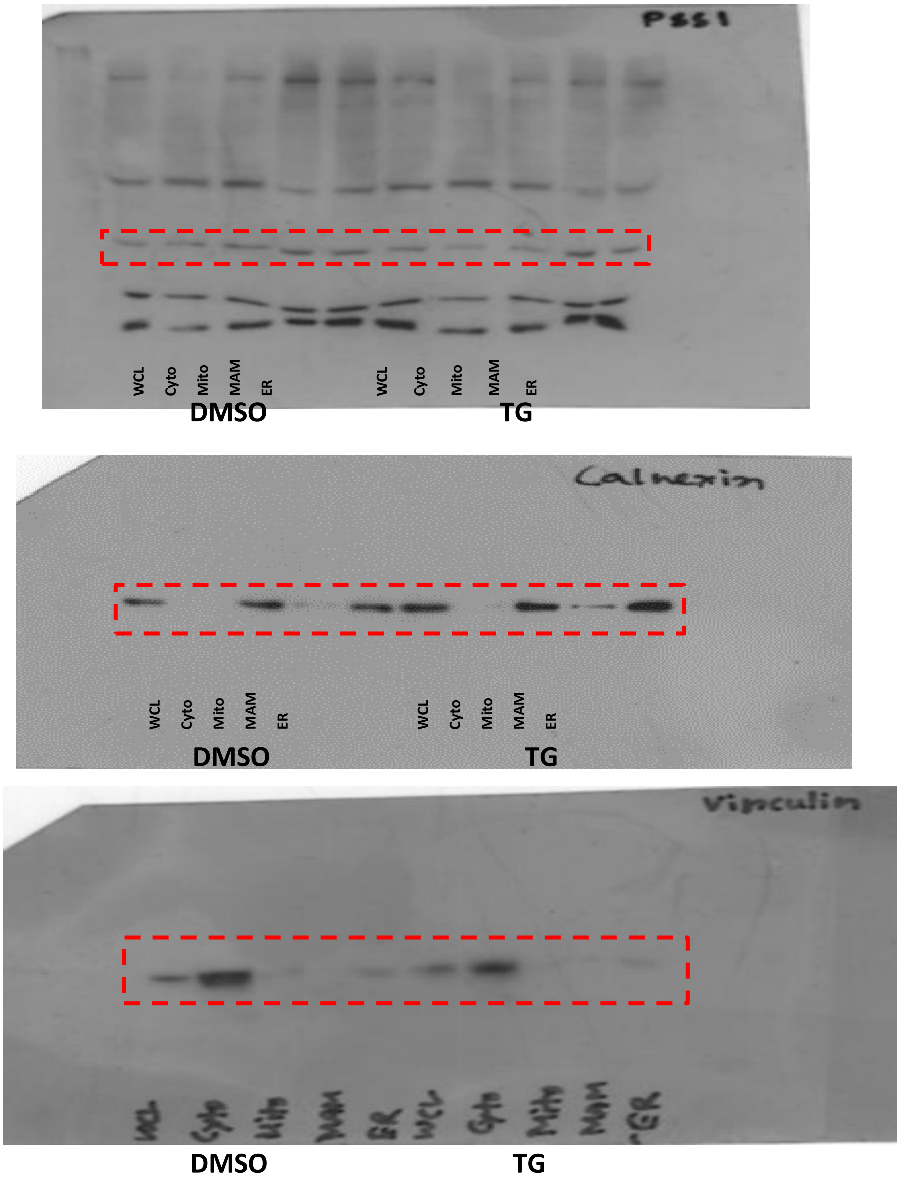
PSS1
WCL
Cyto
Mito
MAM
ER
DMSO
WCL
Cyto
Mito
MAM
ER
TG
Calnexin
WCL
Cyto
Mito
MAM
ER
DMSO
WCL
Cyto
Mito
MAM
ER
TG
Vinculin
DMSO
TG

Fig5A

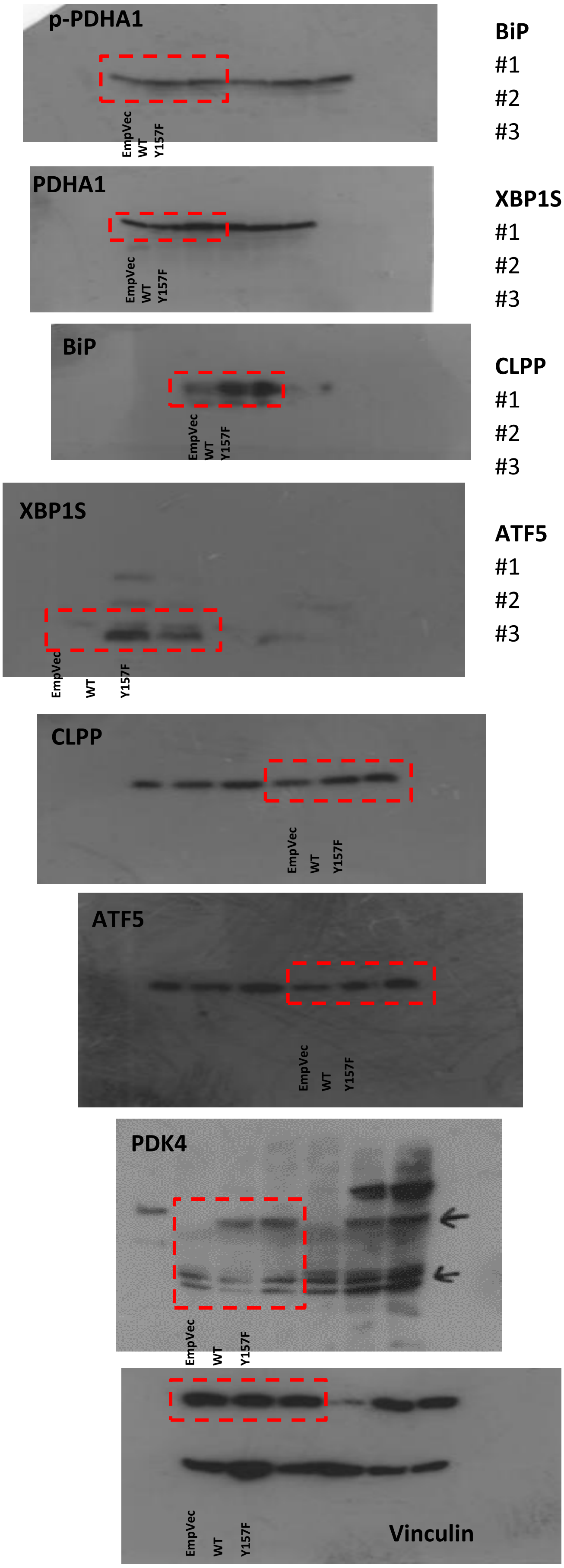

Fig5B

BiP	EmpVec	WT PDK4	Y157F PDK4
#1	1.030717	2.478407	2.292905
#2	1.079142	1.918576	1.523487
#3	0.890141	2.85601	1.93177

XBP1S	EmpVec	WT PDK4	Y157F PDK4
#1	0.703284	8.099338	4.094663
#2	1.188029	8.369386	6.487938
#3	1.108687	4.540061	4.008473

CLPP	EmpVec	WT PDK4	Y157F PDK4
#1	0.982756	1.741772	1.543404
#2	1.161942	1.441699	1.410105
#3	0.855302	1.345088	1.332279

ATF5	EmpVec	WT PDK4	Y157F PDK4
#1	0.940342	1.223163	1.209957
#2	1.124713	1.333778	1.211185
#3	0.934944	1.306944	1.160267

Fig5C

p-PDHA1/PDHA	EmpVec	WT PDK4	Y157F PDK4
#1	1.261299	1.763304	1.111702
#2	0.670997	1.518813	1.10124
#3	1.067703	1.458366	1.004054

Fig5E

Pearson's Co-efficient ER-Mito		
**EmpVec**	**WT PDK4**	**Y157F PDK4**
0.311	0.409	0.55
0.034	0.242	0.314
0.389	0.611	0.2
0.244	0.829	0.414
0.291	0.125	0.234
0.387	0.486	0.188
0.164	0.65	0.404
0.275	0.539	0.39
0.291	0.084	0.43
0.214	0.525	0.514
0.28	0.68	0.262
0.245	0.139	0.565
0.432		
0.112		
0.045		
0.302		
0.146		
0.038		
0.19		
0.404		
0.006		
0.035		

Fig5G

Mitochondrial length (µm)		
**EmpVec**	**WT PDK4**	**Y157F PDK4**
8.76	3.03	3.723
4.497	2.013	6.722
2.613	0.543	3.314
3.411	2.843	2.523
3.427	1.67	1.463
4.472	3.39	4.831
0.91	3.341	3.733
1.508	1.901	2.467
2.317	0.844	1.718
2.815	3.322	3.39
4.989	2.569	2.006
2.244	2.045	5.27
3.011	5.793	5.231
4.019	1.055	5.702
2.052	0.942	3.915
4.257	2.285	2.666

Fig5H

	Percentage population		
**Category**	**EmpVec**	**WT PDK4**	**Y157F PDK4**
**Fragmented**	27.5	62.60163	44.48052
**Intermediate**	50.9375	28.99729	39.28571
**Filamentous**	21.5625	8.401084	16.23377

1.388	2.903	2.146
3.697	2.352	1.773
5.513	1.372	1.857
3.217	0.263	2.425
6.4	0.777	4.247
5.825	1.647	2.143
6.333	0.684	3.124
1.885	2.481	1.126
4.543	2.239	2.477
3.649	3.233	7.034
3.658	3.102	4.887
3.294	2.354	2.735
5.152	0.971	6.929
3.276	2.138	4.697
3.099	0.623	1.279
1.118	0.837	5.802
5.695	2.173	0.662
3.421	0.329	3.078
1.954	2.365	2.677
2.673	3.304	7.297
5.914	2.271	4.574
2.398	0.897	6.411
3.749	2.472	2.004
2.96	3.785	0.857
2.964	3.007	0.932
4.933	0.562	2.49
1.696	3.356	3.502
4.038	1.069	5.779
3.392	0.75	3.479
4.719	0.661	5.866
1.7	3.186	1.091
4.054	0.621	2.417
4.08	1.085	1.856
2.518	1.588	4.236
4.008	2.001	8.699
4.762	0.843	1.318
5.07	3.279	2.362
5.26	1.177	5.037
1.324	1.096	3.208
11.418	1.515	2.05
9.745	1.369	2.272
2.428	3.274	2.359
3.192	8.259	8.861
3.147	0.942	4.645
4.226	1.951	2.102
3.001	0.743	3.863
5.741	1.165	5.123
1.021	0.607	4.649
4.488	1.071	3.117
3.104	1.48	2.646

7.095	0.607	8.088
4.051	1.658	5.714
3.584	0.648	5.712
5.564	1.264	3.382
4.895	0.514	4.034
3.545	0.92	4.353
6.547	1.158	4.587
2.871	0.607	3.223
7.161	3.252	7.7
4.881	3.174	4.194
2.636	2.18	2.658
4.955	0.558	2.632
5.321	2.821	2.019
6.993	2.307	0.959
3.306	1.541	0.659
8.325	4.101	4.541
5.876	1.055	1.799
4.258	3.752	1.97
5.23	1.552	3.283
4.689	0.592	7.252
4.651	1.998	1.538
4.542	5.001	2.616
0.728	1.023	0.71
2.476	0.461	2.507
3.46	4.132	1.179
3.492	1.21	3.526
2.097	0.651	3.553
1.238	1.096	1.447
2.932	0.543	2.154
2.772	2.529	5.622
2.566	3.163	2.089
1.747	2.839	3.125
4.537	1.712	2.794
2.936	3.915	3.634
2.704	3.701	3.24
2.48	2.575	9.402
0.788	3.04	3.656
2.402	1.854	1.463
4.639	0.614	1.975
0.974	1.925	1.138
3.286	3.415	1.957
2.507	1.91	1.771
2.862	1.372	2.882
3.881	0.329	3.054
4.621	0.942	5.02
2.96	2.163	3.849
3.52	2.668	1.915
3.082	1.004	2.719
3.011	3.671	2.565
4.512	1.986	1.504

1.979	3.379	2.004
2.862	1.36	2.575
1.86	4.85	1.659
3.005	1.143	1.793
3.257	2.495	2.19
1.91	1.04	1.594
3.577	3.861	5.094
3.532	5.267	7.924
2.954	9.728	3.125
3.447	3.578	3.465
3.311	8.071	2.917
3.702	1.196	4.578
3.015	5.239	2.046
2.734	3.568	2.509
1.569	0.885	4.233
3.279	4.533	2.487
5.025	0.767	7.943
2.485	3.755	2.66
3.381	3.775	5.305
5.397	2.054	4.569
2.618	3.74	3.185
1.598	4.147	6.473
3.407	2.359	5.023
4.903	5.223	2.724
5.085	1.384	5.72
3.711	3.604	5.992
3.072	4.6	4.488
2.465	2.126	7.58
3.285	3.769	3.889
2.026	3.571	3.627
3.309	0.343	7.072
3.285	5.939	2.532
5.829	4.212	5.847
1.696	2.151	2.284
4.228	3.618	5.697
1.837	1.495	5.369
3.976	1.794	3.89
3.196	0.514	3.803
3.212	6.463	5.23
11.064	0.712	5.655
7.896	1.332	1.463
8.899	3.869	2.357
12.167	1.12	8.344
5.584	4.758	2.606
4.133	3.44	2.359
1.257	4.374	0.937
5.462	1.59	4.804
9.113	0.824	3.196
6.458	4.21	1.741
4.825	7.674	3.379

9.195	8.23	1.874
8.278	1.298	4.986
3.922	2.414	3.915
6.691	1.369	3.428
1.423	1.027	4.706
2.112	0.858	1.538
2.988	6.529	3.639
3.172	1.508	4.7
3.161	1.981	6.11
12.677	2.046	3.197
4.239	9.185	5.293
6.086	3.457	5.157
4.519	2.86	9.151
2.432	1.213	1.627
4.588	1.542	4.833
3.325	4.083	2.068
2.778	4.852	3.174
4.841	2.035	3.753
4.259	0.971	4.026
2.731	2.138	4.978
3.855	0.623	2.791
2.19	0.837	1.238
2.939	2.173	2.393
2.557	0.329	1.779
2.705	2.365	5.478
3.893	3.304	1.771
7.27	2.271	4.265
7.446	0.897	3.823
2.24	2.472	2.125
1.568	3.785	3.427
5.047	3.007	2.03
4.52	0.562	10.752
3.469	3.356	0.751
7.226	1.069	5.105
4.22	0.75	4.623
3.297	0.661	1.166
3.754	3.186	5.668
5.244	0.621	2.007
4.303	1.085	2.657
2.233	1.588	9.338
7.021	0.971	2.041
2.73	2.138	7.803
3.043	0.623	1.081
4.213	0.837	2.625
3.059	2.173	3.435
4.256	0.329	4.248
5.96	2.365	5.573
1.658	3.304	3.964
2.75	2.271	1.832
4.441	0.897	5.882

6.767	2.472	2.755
2.881	3.785	4.891
0.923	3.007	8.792
4.607	0.562	1.78
2.509	3.356	7.311
0.744	1.069	1.445
3.403	0.75	2.899
3.514	0.661	1.793
2.814	3.186	1.68
3.143	0.621	2.376
5.221	1.085	2.215
3.074	1.588	3.205
5.359	3.341	1.334
2.424	1.901	1.651
2.89	0.844	2.264
3.935	3.322	1.544
4.712	2.569	1.215
3.923	2.045	2.154
2.658	5.793	1.226
1.873	1.055	1.266
2.714	0.942	3.255
3.044	2.285	2.209
4.916	2.903	2.79
2.57	2.352	1.992
1.089	1.372	2.834
3.902	0.263	2.366
2.809	0.777	2.706
2.054	1.647	1.409
4.687	0.684	3.479
3.612	2.481	1.688
1.64	2.239	2.811
3.516	3.233	1.921
13.828	3.102	3.789
0.566	2.354	4.254
4.467	0.971	3.784
1.241	2.138	2.129
2.437	0.623	7.029
2.827	0.837	2.153
3.572	2.173	0.48
5.055	3.341	2.314
4.057	1.901	4.55
6.601	0.844	1.964
5.575	3.322	1.439
8.58	2.569	2.852
7.946	2.045	2.176
3.959	5.793	2.882
0.983	1.055	4.443
4.114	0.942	5.335
3.619	2.285	2.918
0.123	2.903	2.717

3.999	2.352	7.025
0.916	1.372	4.314
2.942	0.263	0.844
5.297	0.777	1.977
1.892	1.647	3.166
2.537	0.684	3.733
3.831	1.481	3.168
2.625	2.239	0.884
4.86	0.233	1.097
3.026	3.102	1.091
3.893	2.354	5.342
3.203	0.971	2.202
0.923	2.138	1.793
2.939	0.623	2.936
8.735	0.837	5.825
4.219	2.173	2.081
3.494	3.341	1.12
13.028	1.901	1.063
0.658	0.844	6.74
3.186	3.322	4.609
8.76	2.569	3.709
7.497	2.045	3.597
4.613	5.793	2.049
4.411	1.055	5.326
6.427	0.942	0.771
6.472	2.285	1.134
0.91	1.903	5.606
1.508	2.352	2.102
2.317	1.372	2.834
6.815	0.263	2.161
4.989	0.777	4.687
2.644	1.647	4.163
5.011	0.684	3.66
4.019	2.481	4.435
3.052	2.239	4.729
4.257	3.233	1.279
1.388	3.102	2.259
3.697	2.354	3.178
5.513	0.971	3.317
7.217	2.138	3.482
6.4	0.623	2.746
5.825	0.837	1.371
6.333	2.173	2.744
1.885	3.341	6.772
4.543	1.901	1.238
3.649	0.844	4.704
3.658	3.322	6.49
3.294	2.569	4.109
7.152	2.045	3.706
3.276	5.793	3.101

3.099	1.055	3.865
1.118	0.942	3.332
5.695	2.285	1.902
6.421	2.903	2.876
1.954	2.352	3.306
6.673	1.372	3.051
2.914	0.263	5.412
2.398	0.777	2.563
3.749	1.647	2.669
6.96	0.684	1.684
3.964	2.481	3.714
5.933	2.239	7.782
1.696	3.233	7.282
8.038	3.102	2.754
3.392	2.354	5.088
4.719	0.971	4.716
1.7	2.138	4.354
4.054	0.623	1.031
4.08	0.837	2.068
2.518	2.173	1.768
4.008	5.052	6.192
4.762	2.929	3.193
5.07	7.928	1.774
5.26	4.299	3.038
1.324	6.445	0.768
11.418	4.077	1.409
3.745	5.425	4.058
7.428	2.945	2.452
4.192	9.286	1.385
8.147	4.595	1.451
4.226	3.492	2.254
2.501	8.976	1.358
5.741	2.912	1.333
1.021	13.54	2.164
4.488	7.221	4.955
6.104	4.052	1.126
7.095	2.929	1.473
3.051	7.928	2.068
3.584	4.299	1.065
5.564	6.445	2.861
4.895	4.077	1.627
3.545	5.425	2.05
6.547	2.945	2.063
2.871	2.286	5.492
3.161	4.595	1.298
4.881	0.492	5.02
4.636	8.976	9.629
4.955	2.912	5.555
7.321	14.54	2.337
6.993	3.221	9.009

3.306	2.945	1.358
7.325	3.492	6.741
5.876	4.077	7.56
4.258		7.817
5.23		4.998
4.689		1.551
4.651		6.503
4.542		5.267
0.728		6.09
2.476		2.47
3.46		3.873
3.492		2.063
2.097		4.996
1.238		1.456
2.932		4.996
2.072		1.912
2.566		1.758
1.747		1.521
1.537		1.718
2.636		1.983
2.204		3.467
2.48		2.632
0.788		3.339
2.402		2.293
4.639		4.82
0.974		4.105
1.286		3.861
2.407		10.801
2.062		1.701
1.881		1.324
1.621		3.056
2.96		2.445
3.52		4.16
3.082		2.951
3.011		3.818
1.512		1.298
1.979		4.843
2.862		6.296
1.86		1.517
3.005		6.405
1.257		2.535
1.91		7.149
3.577		1.439
5.532		4.016
2.954		2.623
3.447		1.865
3.311		1.813
3.702		0.589
3.015		4.623
2.734		3.261

1.569	4.197
3.279	4.329
4.025	1.538
2.485	4.351
3.381	1.551
5.397	1.919
2.618	3.672
1.598	3.237
3.407	7.133
4.903	3.043
5.085	1.506
3.711	3.524
3.072	1.946
2.465	2.815
3.285	1.668
2.026	2.281
3.309	1.105
3.285	2.522
3.829	4.29
1.696	2.109
4.228	1.667
1.837	2.864
3.976	2.024
3.196	1.179
3.212	0.915
11.064	1.371
7.896	1.426
3.899	0.802
7.167	1.887
6.584	1.959
3.133	3.168
1.257	1.504
5.462	2.451
4.113	6.403
6.458	0.915
4.825	3.011
3.195	3.498
8.278	6.704
13.922	4.095
6.691	2.34
1.423	1.846
2.112	3.022
2.988	1.352
3.172	4.065
6.161	4.017
2.677	4.06
4.239	3.039
6.086	5.468
4.519	2.553
2.432	5.647

4.588	1.533
3.325	6.316
2.778	7.723
1.841	2.129
4.259	1.865
2.731	1.504
3.855	5.375
2.19	0.771
2.039	0.672
2.357	1.371
2.105	1.358
2.893	2.193
7.27	1.74
3.446	1.097
2.24	1.725
1.568	3.639
5.047	2.043
1.52	1.358
2.469	5.551
7.226	4.113
4.22	2.629
3.297	1.73
3.754	2.156
5.244	0.932
4.303	1.456
2.233	1.308
7.021	2.047
2.73	3.232
3.043	1.755
2.213	1.701
2.059	1.358
4.256	1.463
5.96	2.643
1.658	1.503
1.75	1.68
0.941	3.974
2.967	1.186
0.881	1.385
0.923	3.303
1.607	2.264
2.109	2.418
1.744	1.376
3.403	3.074
1.514	2.109
0.814	4.667
3.143	2.566
1.221	5.909
5.074	6.852
5.359	1.615
2.424	4.365

2.09	3.103
0.935	1.815
2.712	3.649
0.923	3.173
1.658	1.576
3.873	1.426
2.014	3.226
3.044	1.168
1.916	1.267
2.57	2.928
1.089	5.503
2.902	2.822
2.809	4.721
2.054	2.109
1.687	3.475
3.612	1.667
1.64	3.041
3.516	5.675
9.828	2.845
0.866	4.035
4.467	1.856
1.241	3.703
2.437	1.615
2.827	4.275
2.572	2.8
5.055	4.777
4.057	1.065
2.601	1.376
5.575	1.888
8.58	2.948
3.946	4.767
3.959	4.985
0.983	2.586
4.114	4.994
3.619	1.168
0.723	2.602
3.999	3.686
0.916	4.271
2.942	2.292
5.297	2.616
1.892	1.097
1.537	3.618
1.831	0.867
1.625	1.358
4.86	7.173
6.026	1.915
3.893	2.432
6.203	1.86
0.923	1.004
2.939	3.777

8.735	1.715
9.219	5.191
3.494	2.785
3.028	4.006
0.658	0.937
8.186	1.597
10.529	2.169
11.394	1.087
6.778	1.396
2.177	1.318
2.267	1.385
1.527	3.102
3.607	0.98
1.132	3.57
1.558	2.895
0.824	3.487
2.291	1.74
2.26	1.463
4.229	1.667
1.268	3.739
0.727	1.921
4.432	1.621
2.96	1.231
1.72	2.334
3.218	1.445
7.962	8.763
4.546	1.371
0.671	2.272
3.536	3.562
2.516	3.465
5.579	1.211
2.284	5.104
2.092	3.108
2.624	1.911
3.518	2.911
2.055	1.68
2.266	1.595
7.972	2.122
4.674	1.166
9.726	0.932
7.292	2.614
3.122	1.259
1.959	1.651
8.997	2.129
4.188	2.985
1.578	1.631
11.241	1.481
1.012	2.106
5.781	1.701
3.796	4.389

4.092
5.221
1.699
0.441
3.982
3.73
2.642
4.049
8.821
1.21
3.133
0.607
3.972
2.321
2.712
4.219
9.239
4.095
2.722
3.958
5.327
2.605
1.437
0.621

Fig6A

ER-CEpiA			ΔF/F0			
**Time(s)**		**EmpVec**			**PDK4**	
0	0	0	0	0	0	0
19.138	-0.0441	-0.01742	-0.00499	-0.06167	-0.02987	-0.09937
38.276	-0.05305	-0.09305	-0.01205	-0.1208	-0.17154	-0.21098
57.414	-0.08605	-0.12905	-0.03409	-0.24098	-0.19399	-0.32897
76.552	-0.0739	-0.12827	-0.02312	-0.27779	-0.20986	-0.32091
95.691	-0.07663	-0.04346	-0.09346	-0.29984	-0.34429	-0.4378
114.829	-0.06793	-0.10934	-0.02213	-0.39068	-0.33468	-0.25278
133.967	-0.0804	-0.05493	-0.11674	-0.30178	-0.34362	-0.3901
153.105	-0.09963	-0.04323	-0.1289	-0.30334	-0.35143	-0.4286
172.243	-0.12714	-0.08434	-0.17192	-0.30908	-0.39108	-0.47138
191.381	-0.13027	-0.09795	-0.17125	-0.30912	-0.37859	-0.48109
210.519	-0.14214	-0.17897	-0.12478	-0.26128	-0.3837	-0.50063
229.657	-0.13445	-0.1689	-0.10965	-0.32912	-0.38337	-0.46314
248.796	-0.10955	-0.17987	-0.08834	-0.35865	-0.3029	-0.42932
267.934	-0.18902	-0.11267	-0.15952	-0.29213	-0.3491	-0.43101
287.072	-0.15175	-0.1198	-0.09658	-0.36466	-0.32904	-0.39927
306.21	-0.12633	-0.10873	-0.14987	-0.44007	-0.38601	-0.32238
325.348	-0.16785	-0.12322	-0.0986	-0.33097	-0.39271	-0.45985
344.486	-0.12601	-0.1081	-0.15781	-0.39954	-0.36946	-0.2885
363.624	-0.08655	-0.11509	-0.16657	-0.3198	-0.35706	-0.39689
382.762	-0.11984	-0.14838	-0.17983	-0.31109	-0.36763	-0.39368
401.9	-0.11278	-0.0769	-0.14674	-0.33945	-0.37788	-0.4202
421.039	-0.12983	-0.15023	-0.179	-0.31997	-0.35508	-0.38903
440.177	-0.19224	-0.14724	-0.10112	-0.26127	-0.3278	-0.40289
459.315	-0.1076	-0.14165	-0.1779	-0.37427	-0.33701	-0.29457
478.453	-0.10976	-0.13052	-0.18014	-0.30878	-0.33618	-0.38987
497.591	-0.149	-0.08001	-0.19976	-0.31534	-0.34502	-0.3843
516.729	-0.08005	-0.13465	-0.18987	-0.25901	-0.34368	-0.46167
535.867	-0.06945	-0.12144	-0.19987	-0.30898	-0.34763	-0.39834
555.005	-0.0963	-0.13593	-0.18673	-0.28945	-0.33681	-0.38789

Fig6B

Mito-CEpiA			ΔF/F0			
Time(s)		EmpVec			PDK4	
0	0	0	0	0	0	0
19.138	-0.07691	-0.03789	-0.12009	0.0598	0.024335	-0.01865
38.276	-0.18494	-0.11091	-0.08954	0.07544	0.039318	0.01586
57.414	-0.18614	-0.07998	-0.12094	0.023982	-0.03115	0.07996
76.552	-0.11778	-0.1657	-0.07267	0.01613	-0.03887	0.06393
95.691	-0.12792	-0.06891	-0.19286	-0.01968	0.01446	0.04559
114.829	-0.17438	-0.13181	-0.08904	-0.03241	0.00791	-0.07334
133.967	-0.1507	-0.10017	-0.21779	-0.09915	-0.04132	-0.00877
153.105	-0.11084	-0.16233	-0.21881	-0.05644	-0.01266	-0.10194
172.243	-0.17069	-0.1075	-0.21638	-0.0167	-0.07284	-0.11832
191.381	-0.1388	-0.17814	-0.22897	-0.13773	-0.08567	-0.01774
210.519	-0.16958	-0.11989	-0.22656	-0.0223	-0.06567	-0.10779
229.657	-0.14341	-0.19031	-0.25667	-0.04581	-0.07848	-0.1107
248.796	-0.17228	-0.12565	-0.23998	-0.08391	-0.05739	-0.01982
267.934	-0.23328	-0.18844	-0.13009	-0.08185	-0.12245	-0.04119
287.072	-0.15998	-0.1911	-0.2355	-0.13796	-0.04599	-0.08969
306.21	-0.18349	-0.13979	-0.24556	-0.15414	-0.10054	-0.0453
325.348	-0.1964	-0.13776	-0.25594	-0.04412	-0.0903	-0.13323
344.486	-0.1214	-0.18485	-0.25608	-0.06559	-0.10659	-0.14427
363.624	-0.21615	-0.2695	-0.16899	-0.15901	-0.12026	-0.08992
382.762	-0.16798	-0.2789	-0.21858	-0.1026	-0.14598	-0.07021
401.9	-0.14008	-0.19896	-0.25437	-0.11865	-0.14185	-0.08612
421.039	-0.14478	-0.18799	-0.23763	-0.04793	-0.08122	-0.13012
440.177	-0.15878	-0.20062	-0.25892	-0.05435	-0.0945	-0.1319
459.315	-0.1469	-0.19962	-0.25767	-0.07023	-0.03367	-0.1165
478.453	-0.13308	-0.19403	-0.2487	-0.0578	-0.09372	-0.12014
497.591	-0.25998	-0.20131	-0.17478	-0.0432	-0.07803	-0.11984
516.729	-0.27709	-0.21106	-0.16879	-0.03912	-0.0818	-0.12218
535.867	-0.2765	-0.1583	-0.21174	-0.04145	-0.08408	-0.13997
555.005	-0.29768	-0.21214	-0.16895	-0.04991	-0.07957	-0.11099

Fig6C

	Relative Luminescence (RLu)			
	5.5mM Glucose		25mM Glucose	
	**EmpVec**	**PDK4**	**EmpVec**	**PDK4**
#1	50054	39275	59700	36698
#2	51337	40645	57642	49063
#3	49587	44918	67535	48780

Fig6D

	Glucose dependency		FAO and AAO Capacity	
	**EmpVec**	**PDK4**	**EmpVec**	**PDK4**
#1	75.58660559	92.73963355	24.41339441	7.260366445
#2	82.71377214	83.23456402	17.28622786	16.76543598
#3	87.93062424	91.13041658	12.06937576	8.869583419
#4	84.82512	87.35478	15.17488	12.64522
#5	77.7694	87.91026	22.2306	12.08974
#6	80.7225	85.45546	19.2775	14.54454

	Mitochondrial dependency		Glycolytic capacity	
	**EmpVec**	**PDK4**	**EmpVec**	**PDK4**
#1	38.79780778	2.493839006	61.20219222	97.50616099
#2	9.863159749	6.016188871	90.13684025	93.98381113
#3	20.66291946	2.132977635	79.33708054	97.86702237
#4	57.53236	18.99017	42.46764	81.00983
#5	57.91258	23.59784	42.08742	76.40216
#6	51.55993	16.35632	48.44007	83.64368

Fig6E

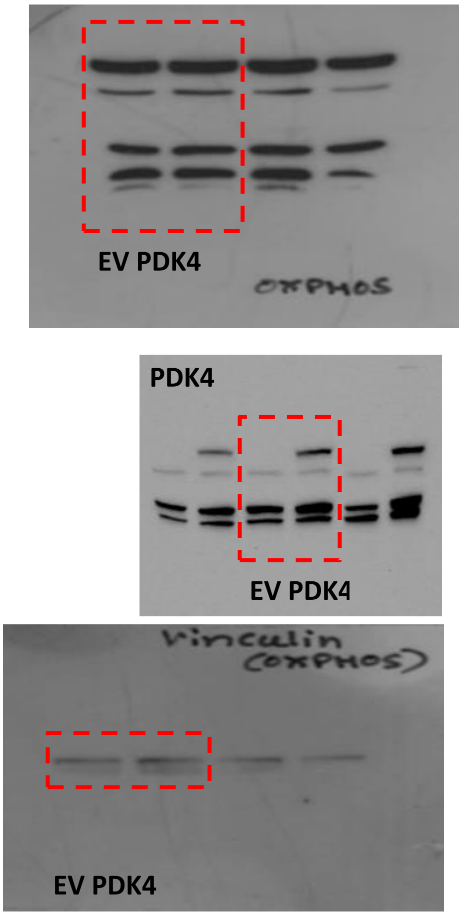

Fig6F

Complex I	EmpVec	PDK4
#1	1.03825	0.341428
#2	1.077117	0.749611
#3	0.884633	0.342262

Complex II	EmpVec	PDK4
#1	0.917792	1.021336
#2	1.256366	1.716826
#3	0.825842	0.827917

Complex III	EmpVec	PDK4
#1	1.752418	2.007959
#2	0.754929	0.894125
#3	0.492654	0.59431

Complex IV	EmpVec	PDK4
#1	1.149434	1.323052
#2	1.151444	1.264876
#3	0.699122	0.69295

Complex V	EmpVec	PDK4
#1	1.702951	1.556063
#2	0.772744	1.154516
#3	0.524305	0.808731

Fig6H

Mito potential (	DMSO	CCCP	EmpVec	PDK4
#1	1.088883	0.19138	1.066777	0.853045
#2	0.960868	0.191964	0.948726	0.790698
#3	0.950249	0.204217	0.984496	0.812514

Fig6J

Mito mass (MG	DMSO	CCCP	EmpVec	PDK4
#1	1.043759	0.749378	0.991908	0.923314
#2	0.987071	0.742914	1.02659	1.039692
#3	0.96917	0.811537	0.981503	1.047399

Fig6K
(TMRM/MG)

Normalized Mit	DMSO	CCCP	EmpVec	PDK4
#1	1.044219	0.255627	1.074519	0.923069
#2	0.974375	0.258637	0.923328	0.759832
#3	0.981406	0.25188	1.002153	0.775051

Fig6M

ROS (DCFDA)	DMSO	CCCP	EmpVec	PDK4
#1	1.071092	12.82486	1.02375704	1.378888
#2	0.989878	11.83498	0.95762919	1.512368
#3	0.95339	12.15513	1.06784227	1.522165
#4	1.012241	12.12688	0.95885378	1.533186
#5	0.973399	10.74976	0.99191771	1.522165

Fig7A

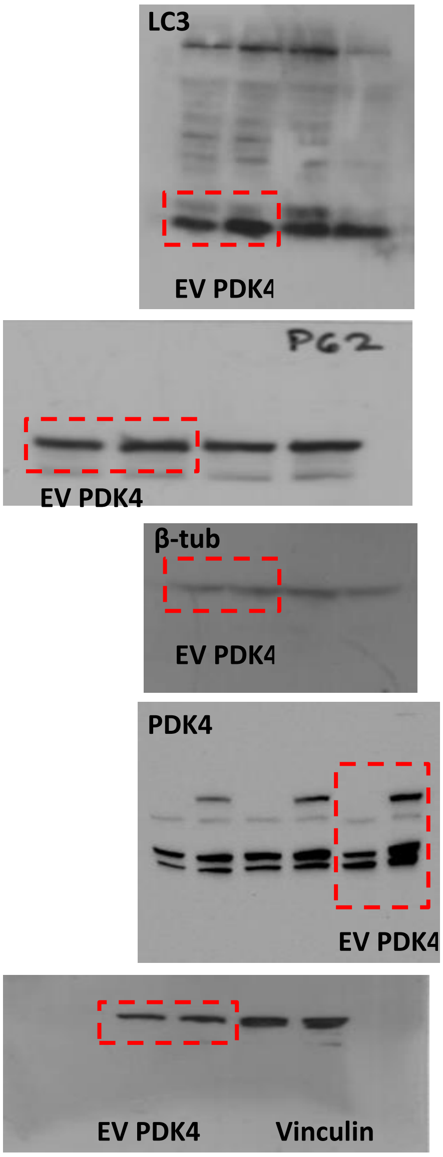

Fig7B

LC3 II/I	EmpVec	PDK4
#1	1.271847	2.530604
#2	0.938963	2.362037
#3	0.888194	1.85344
#4	0.900996	1.582677

Fig7C

P62	EmpVec	PDK4
#1	1.004403	2.072029
#2	0.874206	1.974691
#3	1.12139	2.77357

Fig7E

Percentage of red puncta

EmpVec	PDK4
69.69697	29.5082
65.21739	19.44444
89.58333	38.23529
81.48148	34.61538
36.66667	10.52632
80	26.08696
85.10638	23.07692
74.19355	35.8209
69.93007	20
75	21.42857
49.23077	28.57143
62.5	14.94253
41.86047	12.12121
45	17.94872
50	10.71429
81.48148	24.44444
63.82979	18.51852
67.64706	5.714286

Fig7G

	Fluoroscence percentage of red only		
**EmpVec**	**PDK4**	**EmpVec (TG)**	**PDK4 (TG)**
45.64413	54.7741	66.96349065	29.07232
54.02207	54.79866	50.28157683	37.19403
40.04132	54.05943	50.48390727	38.84786
37.7041	61.6931	52.73804202	24.35233
54.26965	61.79882	51.09327655	40.25454
49.89206	61.64105	57.54276827	26.95909
44.01174	42.64625	58.34418756	26.55337
54.96015	57.20708	59.91774755	30.43294
41.67487	56.41587	52.88996718	49.1257
40.39196	39.7117	64.37544107	43.19784
50.71058	52.58388	50.80166271	36.55285
52.9075	49.10502	51.56057133	27.44108
60.94721	50.62677	55.11298353	42.22699
52.97286	42.97507	48.67724868	28.02528
55.30556	55.92444	51.20299843	37.70205
43.2105	44.05655	53.45631672	31.79612
	50.98859	51.56330973	40.30313
	53.59433	55.55555556	31.83254
	45.46074	52.12341198	27.72392
	53.29649	52.00617284	42.40838
	44.94505	55.66807314	46.79853
	51.52542	54.6981389	45.59122
	55.28384	50.69167644	32.68775
		49.75814253	40.21874
