# Supplementary material for: The impact of ERUPR on mitochondrial integrity mediated by PDK4
Source: Cell Death Dis. 2025 Jul 29;16(1):573. doi: 10.1038/s41419-025-07743-5 (PMC12307875; doi:10.1038/s41419-025-07743-5)

Suppl. Fig1A

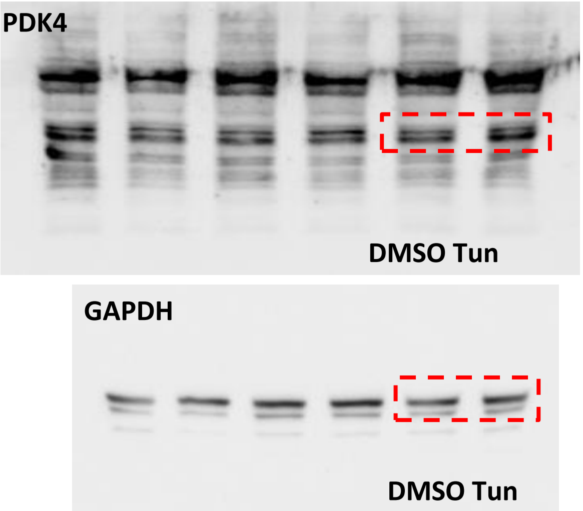

Suppl. Fig1B

PDK4	DMSO	Tun
#1	0.974091	1.330817249
#2	0.918691	1.583498546
#3	1.107218	1.912067044

Suppl. Fig2A

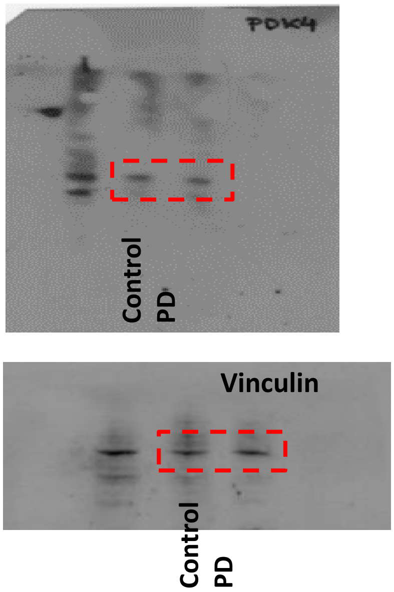

Suppl. Fig2B

PDK4	Control	PD
#1	0.932068	0.489823
#2	1.198942	1.476704
#3	0.86899	1.085101

Suppl. Fig2C

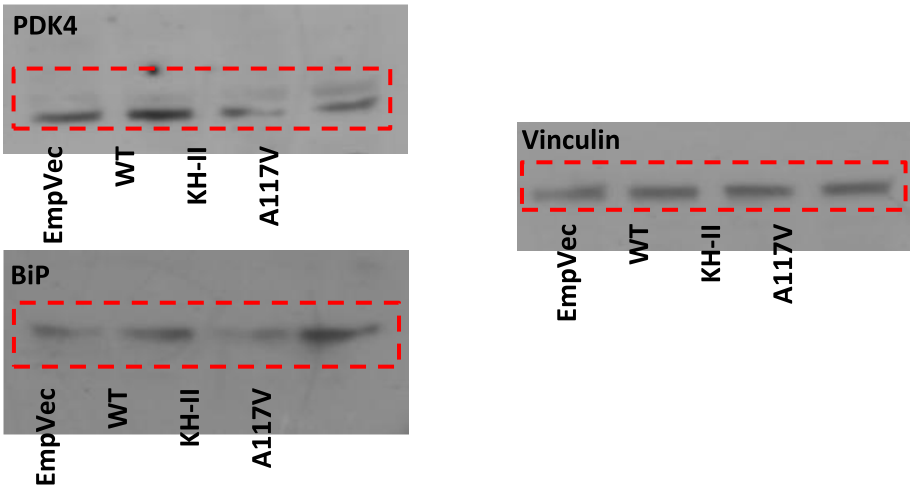

Suppl. Fig2D

PDK4	EmpVec	WT	KH-II	A117V
#1	0.856402	0.982487	1.573395	1.64
#2	1.127385	0.941682	0.946345	1.552719
#3	1.016213	1.197017	1.525381	1.4569

BiP	EmpVec	WT	KH-II	A117V
#1	0.858020414	0.699865268	0.662445125	1.57952207
#2	1.308888759	1.22936995	1.182200042	2.04849391
#3	0.833090827	1.419828258	0.540125793	2.05245562

Suppl. Fig3A

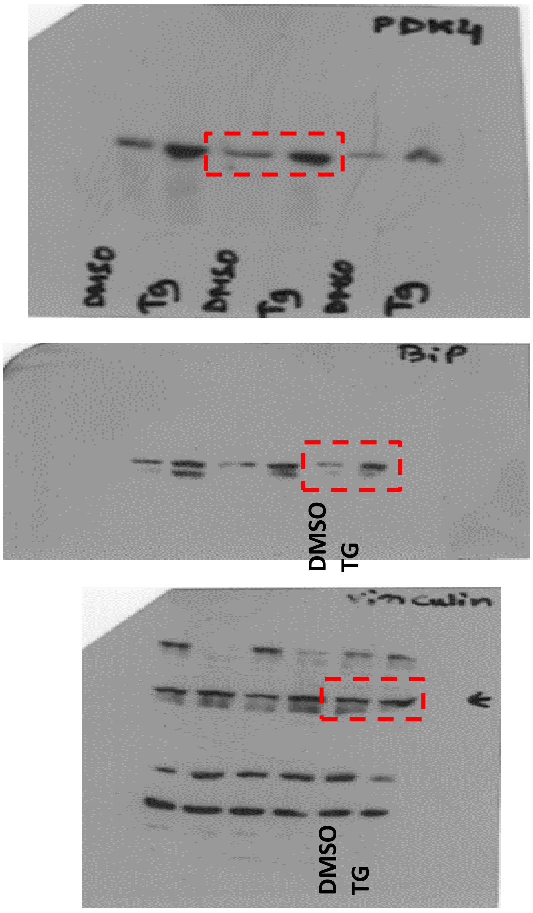

PDK4	DMSO	TG
#1	1.002308	1.68803
#2	1.111007	1.369337
#3	0.886685	1.995278

BiP	DMSO	TG
#1	0.97399	1.66361
#2	1.088881	1.703168
#3	0.937129	2.240804

Suppl. Fig3C

Overlap index

ER-Mito-HSP60

DMSO	TG
0.972446	1.392884
0.753718	1.308631
0.813069	0.885079
0.671955	2.12585
1.402638	0.613434
0.690424	0.811409
1.187853	2.44149
1.998847	0.605133
0.50905	0.807881
	0.618414

Suppl. Fig3E

Overlap index

ER-Mito-MFN2

DMSO	TG
1.229819	1.963512
0.717309	1.013756
0.909308	2.410144
0.717821	0.96614
1.264293	1.330682
1.282213	1.0199
0.833191	1.226235
1.047206	2.513227
1.33	1.366522
0.66884	

Suppl. Fig3H

Mitochondrial length (µm)

DMSO	TG
0.943	1.058
0.988	1.146
1.073	2.25
5.878	0.866
7.842	1.713
3.169	2.7
1.415	1.875
5.173	0.872
4.086	1.705
3.37	1.025
2.78	0.845
1.073	1.435
4.402	0.896
3.246	3.695
6.233	1.208
9.023	1.025
2.302	0.971
3.293	1.659
1.376	1.397
1.79	2.37
6.776	1.641
2.249	1.002
2.318	1.101
2.446	1.829
2.287	1.181
2.95	1.514
3.527	1.179
5.816	0.779
5.442	1.187

3.311	1.101
3.308	3.406
2.432	2.174
3.507	0.924
4.434	1.397
6.002	1.713
5.649	0.924
7.956	1.085
0.662	1.373
6.729	1.75
2.33	1.922
3.749	0.994
1.537	3.192
4.339	2.664
2.096	1.935
2.968	0.858
1.358	4.838
1.566	2.105
3.69	0.8
1.259	2.93
4.079	1.208
6.51	1.417
2.487	1.383
7.569	1.628
0.988	4.18
3.193	1.332
0.943	2.794
3.025	2.139
3.444	1.829
7.391	1.757
1.647	3.436
6.921	1.449
0.95	9.213
1.898	1.449
6.595	2.504
3.668	2.094
5.193	4.812
4.515	2.001
7.139	1.35
11.68	3.386
1.231	2.023
1.385	1.085
4.179	3.792
5.833	0.914
0.95	0.916
8.496	1.126
7.77	1.028
4.926	1.882
1.445	0.855
3.614	2.053

1.029	2.354
7.446	2.124
4.236	1.853
5.17	1.03
1.118	2.535
4.544	2.207
1.82	1.601
6.024	0.832
3.061	1.186
4.247	1.797
7.664	2.009
4.1	1.66
6.768	1.971
2.442	2.22
4.678	2.913
0.792	0.865
3.06	2.041
2.979	1.089
2.391	1.149
4.57	1.534
4.701	3.12
2.292	0.971
2.001	0.916
6.359	2.302
2.183	1.691
3.451	4.107
3.475	1.534
6.451	0.858
3.849	2.124
2.445	0.935
4.49	1.164
5.409	0.624
6.218	1.079
5.861	3.208
3.575	1.324
4.464	2.405
1.799	0.971
4.815	2.871
1.685	2.06
6.482	1.399
5.856	1.399
4.325	2.364
5.859	3.702
4.585	1.696
2.425	1.882
1.606	1.164
1.541	1.453
6.551	0.814
1.399	1.208
1.443	2.269

1.602	3.005
1.625	3.338
6.225	1.399
3.654	1.298
8.77	1.293
3.358	2.36
2.578	2.476
2.291	2.323
2.066	0.935
4.059	3.11
1.502	1.316
8.572	3.744
1.593	1.568
1.396	2.695
2.672	2.241
7.126	2.432
3.013	1.484
1.229	3.079
3	4.664
1.534	1.59
5.449	1.929
1.829	5.05
4.419	1.213
2.759	1.83
3.924	4.292
0.921	1.444
6.214	3.184
2.902	1.712
3.383	2.782
4.844	1.718
1.23	2.784
1.03	1.796
6.74	1.793
1.625	1.264
3.143	1.085
3.414	0.949
3.331	0.792
2.701	1.584
2.585	1.23
1.564	3.169
2.811	2.607
1.397	3.695
1.252	1.711
1.324	1.136
4.079	0.978
3.625	1.569
4.055	3.894
3.751	1.629
3.64	3.034
0.987	1.65

1.332	5.457
3.388	3.394
3.835	2.143
1.781	4.072
1.382	1.004
0.978	2.808
3.205	3.406
2.332	2.174
2.687	0.924
4.658	1.397
1.5	1.713
2.237	0.924
4.748	1.085
2.143	1.373
2.695	1.75
5.57	1.922
5.388	0.994
5.435	3.192
2.514	2.664
3.247	1.935
3.008	0.858
2.303	4.838
3.149	2.105
1.979	0.8
2.658	2.93
0.837	1.208
4.206	1.417
1.369	1.383
6.747	1.628
4.283	4.18
3.396	1.332
5.252	2.794
2.661	2.139
6.613	1.829
3.774	1.757
6.455	3.436
6.88	1.449
3.238	9.213
1.35	1.449
1.82	2.504
6.358	2.094
5.036	4.812
2.854	2.001
3.529	1.35
3.708	3.386
10.638	2.023
3.444	1.085
1.166	3.792
1.374	0.914
3.649	0.916

1.844	1.126
4.018	1.028
1.771	1.882
1.5	0.855
2.433	2.053
4.388	2.354
5.997	2.124
6.072	1.853
3.74	1.03
2.408	2.535
2.098	2.207
3.738	1.601
5.27	0.832
7.278	1.186
3.964	1.797
3.602	2.009
3.117	1.66
1.515	1.971
5.571	2.22
5.565	2.913
8.025	0.865
2.254	2.041
2.582	1.089
3.273	1.149
3.205	1.534
2.332	3.12
2.687	0.971
4.658	0.916
1.5	2.302
2.237	1.691
4.748	4.107
2.143	1.534
2.695	0.858
5.57	2.124
5.388	0.935
5.435	1.164
2.514	0.624
3.247	1.079
3.008	3.208
2.303	1.324
3.149	2.405
1.979	0.971
2.658	2.871
0.837	2.06
4.206	1.399
1.369	1.399
6.747	2.364
4.283	3.702
5.565	1.696
8.025	1.882

2.254	1.164
2.582	1.453
3.273	0.814
3.205	1.208
2.332	2.269
2.687	3.005
4.658	3.338
4.085	1.399
4.35	1.298
1.712	1.293
5.63	2.36
3.497	2.476
6.356	2.323
3.89	0.935
11.239	3.11
5.858	1.316
5.106	3.744
4.357	1.568
3.164	2.695
4.646	2.241
3.176	2.432
6.036	1.484
9.443	3.079
3.756	4.664
6.192	1.59
11.515	1.929
3.491	5.05
1.127	1.213
5.354	1.83
2.707	4.292
11.277	1.444
3.791	3.184
1.593	1.712
4.021	2.782
7.763	1.718
3.29	2.784
4.832	1.796
3.556	1.793
10.602	1.264
2.769	1.085
3.861	0.949
8.929	0.792
5.992	1.584
4.341	1.23
1.716	3.169
5.966	2.607
5.6	3.695
4.529	1.711
2.838	1.136
4.523	0.978

6.66	1.569
5.706	3.894
9.211	2.794
7.45	2.139
5.116	1.829
5.99	1.757
8.234	3.436
3.451	1.449
3.475	9.213
6.451	1.449
3.849	2.504
2.445	2.094
4.49	4.812
5.409	2.001
6.218	1.35
5.861	3.386
3.575	2.023
4.464	1.085
1.799	3.792
4.815	0.914
1.685	0.916
6.482	1.126
5.856	1.028
4.325	1.882
5.859	0.855
4.585	2.053
2.425	2.354
1.606	2.124
1.541	1.853
6.551	1.03
1.399	2.535
1.443	2.207
1.602	1.601
1.625	0.832
6.225	1.186
3.654	1.797
8.77	2.009
3.358	1.66
2.578	1.971
2.291	2.22
2.066	2.913
4.059	0.865
1.502	2.041
8.572	1.089
1.593	1.149
1.396	1.534
2.672	3.12
7.126	0.971
3.013	0.916
1.229	2.302

3	1.691
1.534	4.107
5.449	1.534
1.829	0.858
	2.124
	0.935
	1.164
	0.624
	1.079
	3.208
	1.324
	2.405
	0.971
	2.871
	2.06
	1.399
	1.399
	2.364
	3.702
	1.696
	1.882
	1.164

Suppl. Fig4A

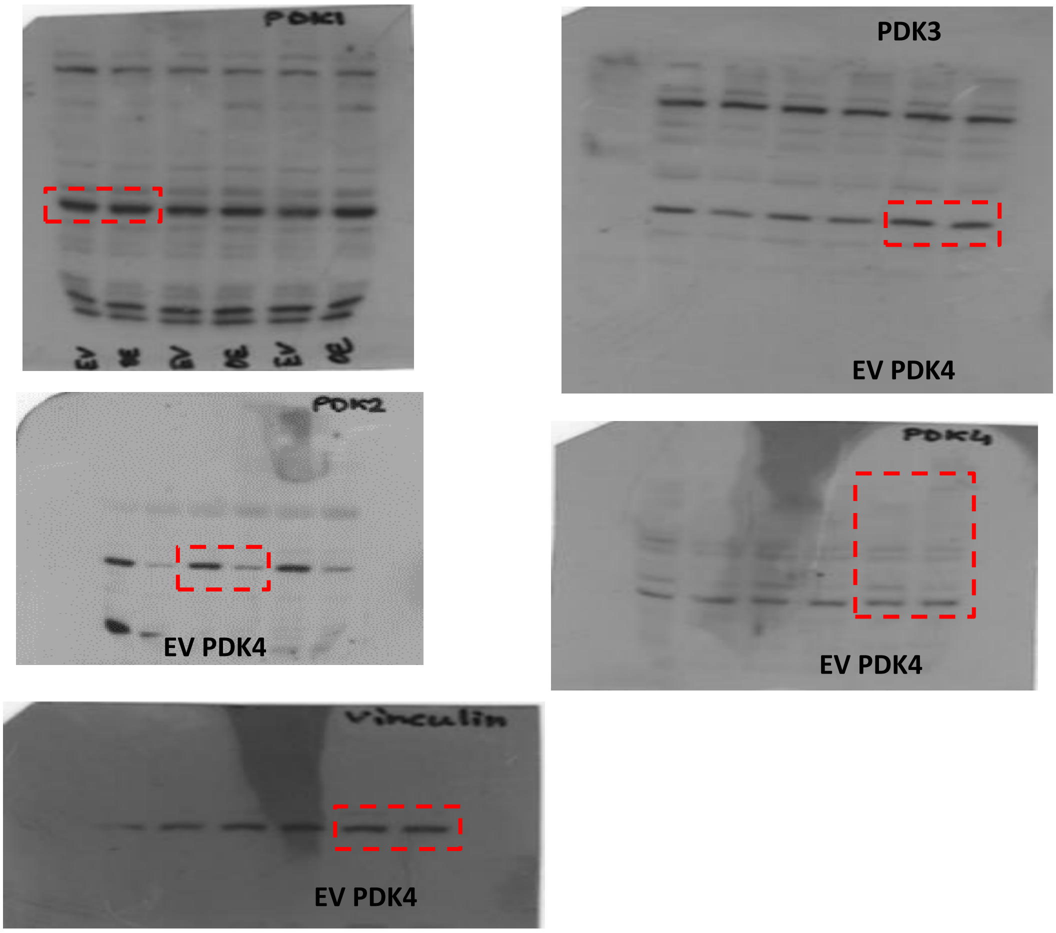

Suppl. Fig4B

PDK1	EmpVec	PDK4
#1	1.849567	0.932506
#2	0.721162	0.564997
#3	0.42927	0.780987

PDK2	EmpVec	PDK4
#1	1.327041	0.234821
#2	0.946268	0.171684
#3	0.72669	0.190941

PDK3	EmpVec	PDK4
#1	0.85303	0.755432
#2	0.653319	1.540603
#3	1.493651	2.25851

Suppl. Fig4C

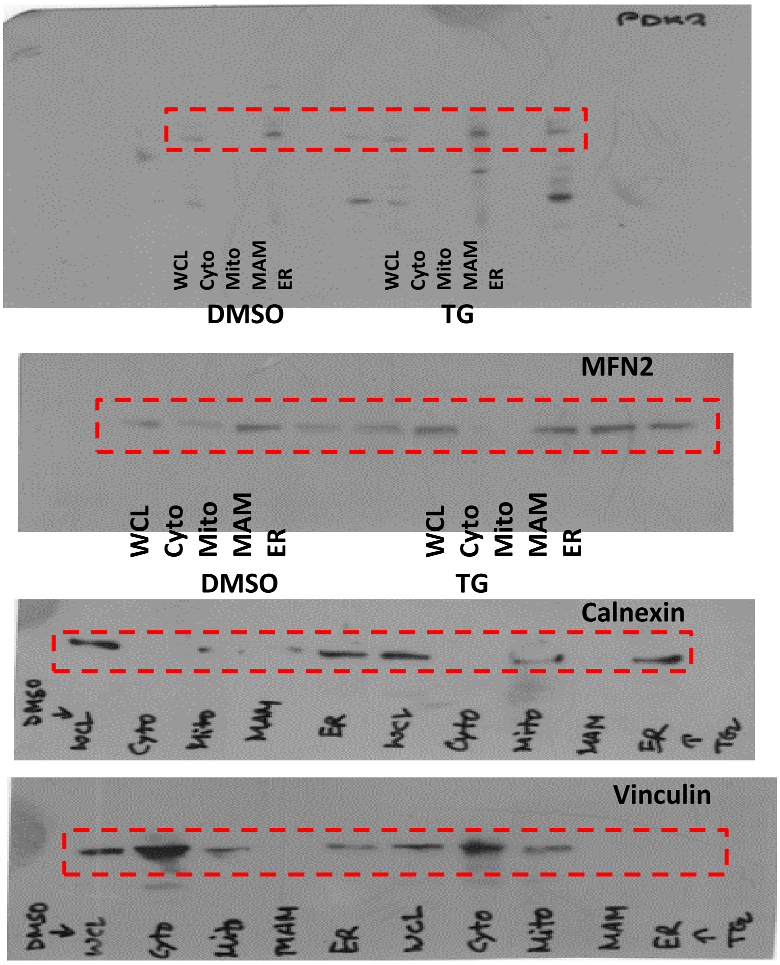

Suppl. Fig5A

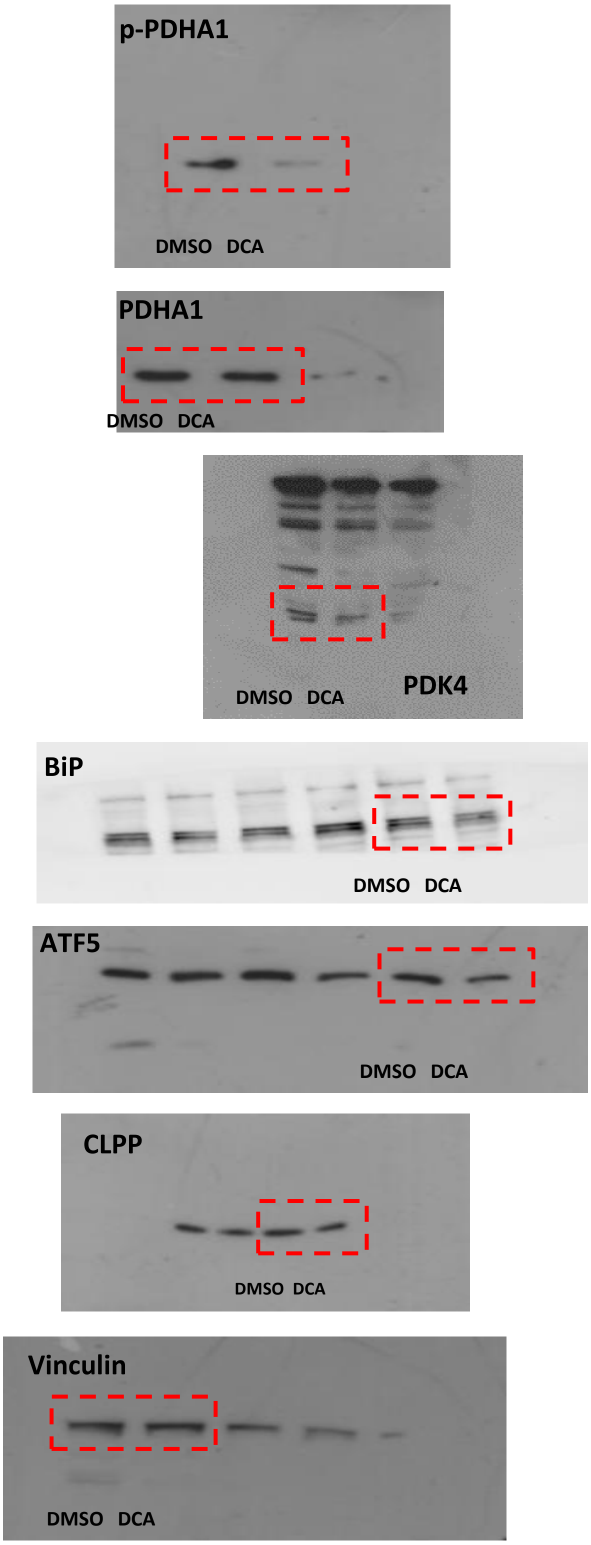

Suppl. Fig5B

p-PDHA1/PDH/	DMSO	DCA
#1	1.181485	0.179657
#2	0.883784	0.202822
#3	0.934731	0.191565

Suppl. Fig5C

PDK4	DMSO	DCA
#1	1.29423	0.475591
#2	0.788162	0.483319
#3	0.917608	0.353454

BiP	DMSO	DCA
#1	0.868247	0.428686
#2	1.089983	0.815426
#3	1.04177	0.571347

ATF5	DMSO	DCA
#1	0.989398	0.614242
#2	1.074402	0.836413
#3	0.936199	0.663718

CLPP	DMSO	DCA
#1	1.028076	0.337327
#2	0.96357	0.395538
#3	1.008354	0.694496

Suppl. Fig5E

Mitochondrial length (µm)	
**DMSO**	**DCA**
2.684	5.925
1.158	1.186
1.143	3.969
3.785	7.369
1.901	4.675
1.743	9.822
3.322	2.82
3.102	4.857
2.903	6.765
4.121	2.43
1.981	0.659
8.976	4.129
8.113	6.999
3.329	6.007
0.844	3.493
1.542	5.881
3.897	9.52
2.239	4.611
4.083	5.819
7.052	1.254
2.239	3.156
2.352	1.981
2.138	4.494
4.016	5.703
2.903	1.326
2.173	4.486
2.271	4.485
2.942	1.766
1.59	3.169
1.552	4.975
3.507	3.579
0.75	3.306
1.055	3.975
5.425	0.98
1.647	8.423
0.942	1.809
3.174	12.591
2.942	1.87
3.069	1.503
1.508	5.367
1.372	3.488
4.163	4.845
2.837	5.233
1.12	1.081
2.138	3.449

Suppl. Fig5F

	Percentage population	
**Category**	**DMSO**	**DCA**
**Fragmented**	36.93694	28.43296
**Intermediate**	51.2012	35.37964
**Filamentous**	11.86186	36.1874

0.837	0.475
1.712	5.907
4.136	5.244
2.535	4.427
2.307	17.839
2.552	2.518
5.793	0.859
5.425	5.561
2.365	9.737
2.514	10.092
2.986	7.705
0.971	3.757
2.538	6.014
5.425	1.68
2.569	7.486
6.445	13.682
2.903	17.673
2.684	5.926
2.854	3.655
5.425	3.59
5.095	6.529
7.052	4.625
2.359	4.214
3.356	1.358
0.971	5.291
3.322	5.841
1.177	6.389
4.374	9.1
5.793	4.848
2.285	1.566
5.531	6.607
7.6	6.328
2.554	6.989
4.132	1.919
9.286	3.532
2.585	4.989
3.769	6.166
2.045	7.882
2.138	5.377
1.12	2.563
2.673	1.584
3.233	1.029
2.045	3.188
4.712	10.536
1.67	3.094
2.86	7.659
2.575	2.509
2.481	7.713
2.843	6.142
3.305	4.735

5.223	3.692
2.173	10.251
0.971	10.537
2.365	2.962
3.341	3.278
4.212	1.809
2.569	1.352
2.285	7.962
1.372	1.605
1.055	5.675
3.415	3.595
0.895	6.233
1.023	1.503
2.543	9.427
2.998	10.407
1.614	12.953
3.785	5.098
3.233	4.365
2.684	12.979
3.769	4.24
3.568	4.136
2.173	2.577
3.568	9.107
3.341	20.189
1.023	7.629
5.793	4.696
4.427	4.949
3.457	12.407
0.971	6.221
2.365	14.077
2.126	1.352
7.221	8.245
2.767	4.581
3.785	5.07
5.578	6.942
2.929	4.77
1.372	1.494
2.271	2.697
2.292	5.841
9.286	4.535
3.263	3.15
3.356	5.166
1.096	1.063
2.045	1.215
2.878	0.659
1.055	4.242
2.668	8.512
0.949	5.339
1.854	6.555
0.942	2.193

2.837	1.566
1.951	4.942
1.743	8.018
2.563	2.724
2.854	2.405
3.843	4.667
2.352	1.543
1.901	6.062
1.165	2.174
3.848	11.157
2.912	2.244
3.996	3.597
2.903	8.751
2.942	5.293
2.472	6.137
2.777	8.104
3.869	6.891
2.138	9.819
3.74	7.429
5.758	4.806
2.045	7.17
1.21	6.987
2.91	4.568
1.48	7.077
4.125	1.447
0.897	7.973
1.04	3.879
3.861	2.007
2.945	1.915
1.925	2.861
1.536	3.023
2.515	4.854
1.027	8.901
2.536	4.397
3.263	5.47
0.75	3.565
3.356	1.975
3.39	4.313
1.004	15.386
3.329	2.834
4.595	9.565
2.352	4.213
8.23	4.053
2.239	5.151
2.929	10.043
4.147	2.007
2.173	2.145
0.684	3.284
4.212	2.452
2.843	5.754

1.36	3.324
2.588	4.747
0.837	2.983
7.676	6.444
3.356	5.561
3.252	8.874
2.013	10.719
9.606	9.604
1.647	10.332
1.213	0.98
2.239	1.004
2.354	8.858
2.352	5.46
3.322	0.768
2.687	4.858
3.233	3.856
2.271	3.312
3.547	2.518
2.942	5.199
0.684	6.134
2.472	6.547
2.831	5.659
0.942	7.776
0.844	3.477
3.617	3.089
5.723	10.014
2.542	8.709
3.901	17.173
6.445	2.337
4.101	4.093
2.514	10.335
3.098	8.206
2.925	0.672
3.118	6.142
3.785	1.799
4.533	1.741
3.356	4.596
2.824	12.085
3.304	3.565
8.976	11.553
3.803	2.73
3.322	1.921
3.001	6.445
3.369	7.633
2.839	2.008
3.869	2.387
3.885	2.249
2.001	0.867
1.614	2.416
2.897	2.081

3.775	3.471
1.711	2.786
2.239	3.595
2.035	2.624
3.186	2.856
3.102	8.074
2.571	2.746
2.529	5.409
3.341	2.689
1.647	2.314
2.986	10.344
2.173	2.295
5.793	1.733
1.096	4.655
8.071	1.72
3.03	12.09
1.794	5.083
4.852	6.477
2.684	3.76
1.541	2.133
3.379	6.822
2.352	3.498
0.844	3.213
3.082	3.956
4.077	1.333
10.328	1.475
3.618	1.982
10.343	5.596
2.292	3.746
2.929	3.439
1.998	4.674
2.481	5.057
1.901	5.154
2.777	8.476
1.004	2.742
4.132	3.298
4.606	2.324
2.045	0.688
3.695	3.26
3.196	8.03
3.352	5.891
2.163	5.701
2.971	4.675
1.48	3.373
2.472	7.386
2.971	2.665
2.971	1.385
2.138	3.465
4.427	6.384
3.571	2.535

4.299	2.789
1.647	3.079
2.671	3.425
4.147	2.357
2.658	2.194
14.54	2.647
3.716	6.268
2.173	1.74
3.44	8.965
2.623	7.367
6.463	1.395
7.676	2.925
2.515	2.897
6.529	2.693
3.341	6.045
2.903	4.549
1.541	9.338
2.873	5.099
4.374	5.28
2.529	1.358
2.778	7.205
1.495	3.272
2.569	5.571
5.758	2.113
2.495	4.659
4.595	10.028
4.132	9.754
5.439	12.091
1.854	5.853
5.267	0.793
1.384	1.122
2.897	5.396
2.651	2.462
2.352	3.903
2.75	1.713
2.929	2.951
2.942	1.768
2.611	0.608
3.274	0.659
0.684	4.976
2.806	6.765
2.365	3.044
2.92	3.734
3.915	1.439
3.186	1.445
1.027	2.656
3.007	5.585
2.903	3.452
1.21	2.656
2.912	2.566

2.569	3.136
2.92	2.176
2.354	1.661
4.077	1.653
2.352	7.933
4.595	3.463
2.569	6.419
2.211	3.591
1.055	4.317
3.264	2.125
1.508	1.959
2.572	9.326
2.825	1.031
4.712	1.073
3.322	2.466
4.595	4.68
0.844	1.538
3.949	6.572
2.354	3.526
5.793	4.316
0.777	1.004
2.839	1.995
6.776	3.969
2.354	7.813
6.463	6.042
0.971	2.665
2.271	1.875
3.492	5.962
3.233	1.823
1.085	4.322
3.233	3.272
2.352	6.32
3.803	5.43
1.096	1.91
1.423	1.483
5.223	1.215
2.575	1.537
3.165	6.212
5.758	2.717
0.777	5.089
3.785	4.17
9.728	0.923
3.915	5.87
3.752	0.713
2.903	2.42
7.674	2.728
2.971	1.3
2.569	1.551
2.912	1.456
2.569	3.292

2.481	1.042
2.365	1.68
4.374	2.96
1.901	1.228
5.001	2.084
2.239	0.997
3.618	3.225
2.013	4.193
3.279	4.842
1.213	5.185
8.185	1.398
2.794	1.063
2.67	2.474
1.372	0.751
1.196	1.318
2.046	2.92
2.821	1.562
2.569	2.749
2.359	2.057
3.571	2.965
2.354	0.884
2.784	2.563
3.701	1.065
1.384	0.879
1.712	0.559
3.775	4.949
2.942	3.07
1.213	0.527
3.322	0.678
1.901	4.839
0.777	2.69
2.824	4.051
1.027	2.596
2.285	4.574
1.36	3.564
0.895	1.295
3.492	2.145
1.171	2.73
3.492	2.687
6.618	3.659
2.569	2.449
5.468	2.668
1.069	4.083
6.529	3.078
1.981	3.793
0.942	4.884
4.85	5.176
2.414	2.386
3.716	4.181
8.998	2.34

3.41	7.239
2.945	1.419
2.613	2.233
2.054	4.185
3.492	3.065
3.915	7.711
1.085	3.566
2.481	1.422
2.901	1.818
2.971	2.299
2.542	1.78
2.821	3.652
2.668	3.169
3.085	2.909
3.888	7.708
3.186	5.994
6.445	1.451
1.004	0.915
7.928	2.275
3.422	2.43
1.055	6.676
3.186	3.283
1.069	8.658
3.007	2.634
3.329	4.201
6.776	1.39
7.786	5.866
5.939	4.627
3.415	0.659
0.684	3.311
2.942	0.649
2.173	3.135
3.082	3.645
2.514	9.254
0.777	3.602
2.86	2.202
2.472	4.157
3.162	1.688
14.54	6.224
1.552	1.031
5.818	7.434
1.21	2.802
1.901	1.063
3.102	1.713
1.508	1.712
5.267	0.531
7.928	4.845
3.252	2.758
2.981	1.426
1.901	1.791

2.684	2.807
3.341	3.402
2.045	4.844
1.04	6.357
2.569	2.411
2.614	1.087
7.221	1.741
2.945	15.161
2.623	6.326
1.213	7.183
2.271	9.812
1.096	5.967
2.481	10.314
0.942	2.88
2.981	9.478
5.239	11.115
2.903	8.345
3.604	11.246
1.369	2.923
3.372	8.835
2.837	6.651
5.468	8.624
7.928	23.474
2.365	13.514
0.942	5.615
8.23	5.669
5.869	11.457
8.071	14.676
1.59	5.555
3.102	3.513
3.322	3.985
3.264	5.279
2.472	5.503
3.671	5.573
2.567	1.344
3.233	9.807
0.837	6.343
8.259	1.73
4.224	5.891
1.372	6.498
1.647	6.605
4.85	10.029
2.481	5.316
1.67	7.083
2.239	7.781
2.668	3.936
1.552	5.565
7.031	5.365
4.224	4.446
4.606	10.029

2.354	6.891
1.372	10.184
3.755	3.617
1.588	3.82
3.026	4.64
1.59	1.831
2.352	5.275
3.102	5.3
1.055	5.353
3.44	7.411
2.647	5.027
2.839	5.931
4.299	10.172
3.356	0.884
4.083	5.739
1.055	5.223
3.425	3.391
3.848	5.713
3.007	2.036
1.91	0.793
2.384	4.388
3.233	2.683
2.986	3.458
2.239	2.301
5.793	4.168
2.647	3.756
1.372	2.309
7.052	2.907
3.233	6.067
3.163	3.878
7.214	1.734
3.304	6.493
2.565	4.686
4.151	4.327
3.304	4.174
2.575	4.67
2.138	4.375
2.329	4.265
2.502	7.643
2.623	3.274
11.677	7.597
4.21	4.429
1.711	8.767
3.004	2.505
3.604	1.751
2.173	1.774
2.481	2.609
4.533	1.179
2.92	0.98
2.858	3.315

0.971	5.827
2.581	1.959
2.495	2.282
9.728	4.717
2.285	7.487
2.945	2.787
2.18	1.966
2.354	9.033
2.569	2.719
3.341	3.401
5.053	3.148
2.239	5.784
2.912	2.118
1.588	3.301
1.023	2.73
2.173	0.796
2.951	1.609
3.755	6.038
3.007	1.875
5.793	3.991
3.102	1.166
4.077	0.793
3.785	8.363
7.928	0.502
4.21	
4.299	
2.352	
2.354	
2.569	
3.322	
0.844	
2.743	
2.045	
1.213	
3.102	
3.75	
3.304	
3.379	
1.495	
3.457	
1.158	
2.541	
8.998	
1.332	
3.102	
2.771	
3.165	
1.372	
3.37	
2.621	

3.837
2.543
1.998
5.793
2.495
3.332
2.837
2.588
3.143
3.389
2.481
2.772
4.679
2.18
1.177
2.86
3.04
1.298
1.196
1.071
3.492

Suppl. Fig6A

**2^-ΔΔCT (ND2)**	**EmpVec**	**PDK4**
#1	0.926063	0.776821
#2	0.39207	0.453503
#3	2.754202	0.260469

**2^-ΔΔCT (SDHA)**	**EmpVec**	**PDK4**
#1	1.769367	0.693394
#2	0.764842	0.713623
#3	0.738942	0.621245

**2^-ΔΔCT (CYTB)**	**EmpVec**	**PDK4**
#1	0.744971	0.876265
#2	0.50883	0.541584
#3	2.63808	0.352393

**2^-ΔΔCT (COX II)**	**EmpVec**	**PDK4**
#1	1.304309	0.867878
#2	0.387773	0.529714
#3	1.977159	0.583695

**2^-ΔΔCT (ATP8)**	**EmpVec**	**PDK4**
#1	0.941844	0.293922
#2	0.665984	0.571056
#3	1.594253	0.477496

Suppl. Fig6C

TMRM					
EmpVec+DMSO	PDK4+DMSO	EmpVec+CCCP	PDK4+CCCP	EmpVec+Oligomycir	PDK4+Oligomycin
1.227914	0.747802	0.422033	0.42615264	1.383162	1.217967
1.060106	0.708312	0.40907	0.49960644	1.269716	1.041215
0.980824	0.694847	0.499908	0.50995629	1.196563	1.08382
0.939224	0.695651	0.508248	0.50774564	1.201588	1.303378
0.951985	0.773928	0.503425	0.486644	1.282477	1.1419
0.839946	0.659577	0.502721	0.58642461	1.443654	1.276247

Suppl. Fig6D

MG					
EmpVec+DMSO	PDK4+DMSO	EmpVec+CCCP	PDK4+CCCP	EmpVec+Oligomycir	PDK4+Oligomycin
1.128106	0.996153	0.717895	0.68378912	0.971197	0.995113
1.036602	0.99085	0.723406	0.69387543	1.103463	0.919725
1.032235	0.871166	0.639908	0.61432879	1.014454	1.000936
1.008215	0.924301	0.764064	0.63138193	0.843402	0.904128
0.918374	0.948217	0.638557	0.67307892	0.996361	1.025684
0.876469	0.939482	0.674015	0.69574711	1.177394	0.835084

Suppl. Fig6E

TMRM/MG					
EmpVec+DMSO	PDK4+DMSO	EmpVec+CCCP	PDK4+CCCP	EmpVec+Oligomycir	PDK4+Oligomycin
1.090684	0.752214	0.589069	0.624488	1.427075	1.226433
1.024751	0.716304	0.566626	0.721485	1.153001	1.134392
0.952124	0.799225	0.782804	0.831788	1.18191	1.085005
0.933463	0.754152	0.666541	0.805814	1.427583	1.444512
1.038704	0.81785	0.78998	0.72448	1.289775	1.115567
0.960275	0.70349	0.747375	0.844582	1.228632	1.531389

Suppl. Fig7A

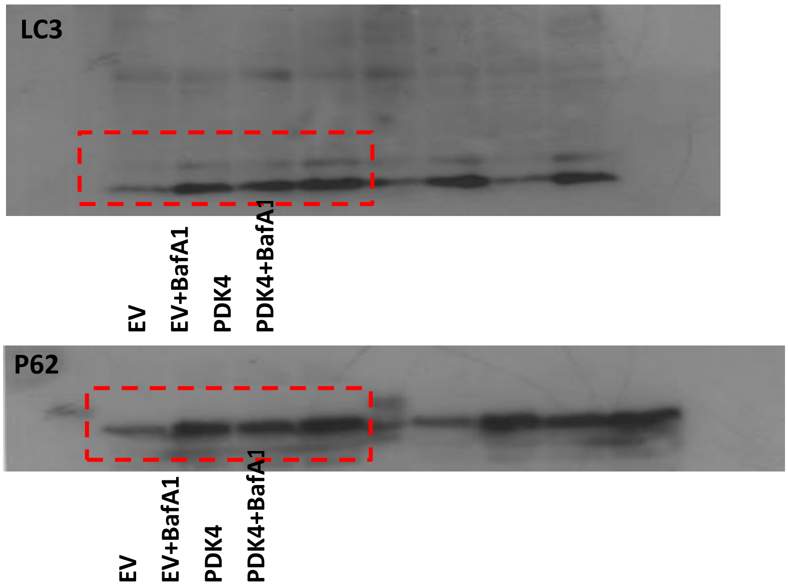

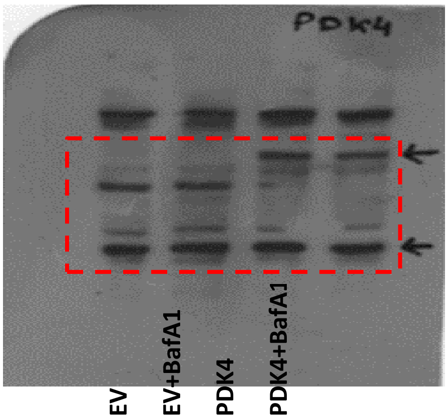

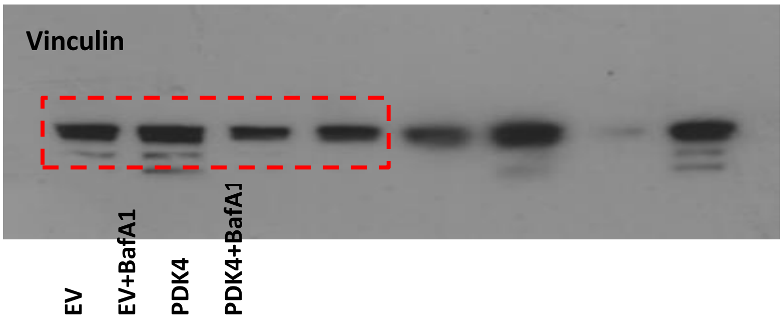

Suppl. Fig7B

LC3 II/I	EmpVec	Baf(+) EmpVec	PDK4	Baf(+) PDK4
#1	0.968505	1.928788	2.063665	1.862894
#2	1.113654	2.304724	2.063011	1.834039
#3	0.917842	3.007547	2.745798	2.070411

Suppl. Fig7C

P62	EmpVec	Baf(+) EmpVec	PDK4	Baf(+) PDK4
#1	1.130366	1.514233	1.846401	1.972932
#2	1.007913	1.471088	1.651616	1.665048
#3	0.86172	1.476389	1.301161	1.589852

Suppl. Fig7D

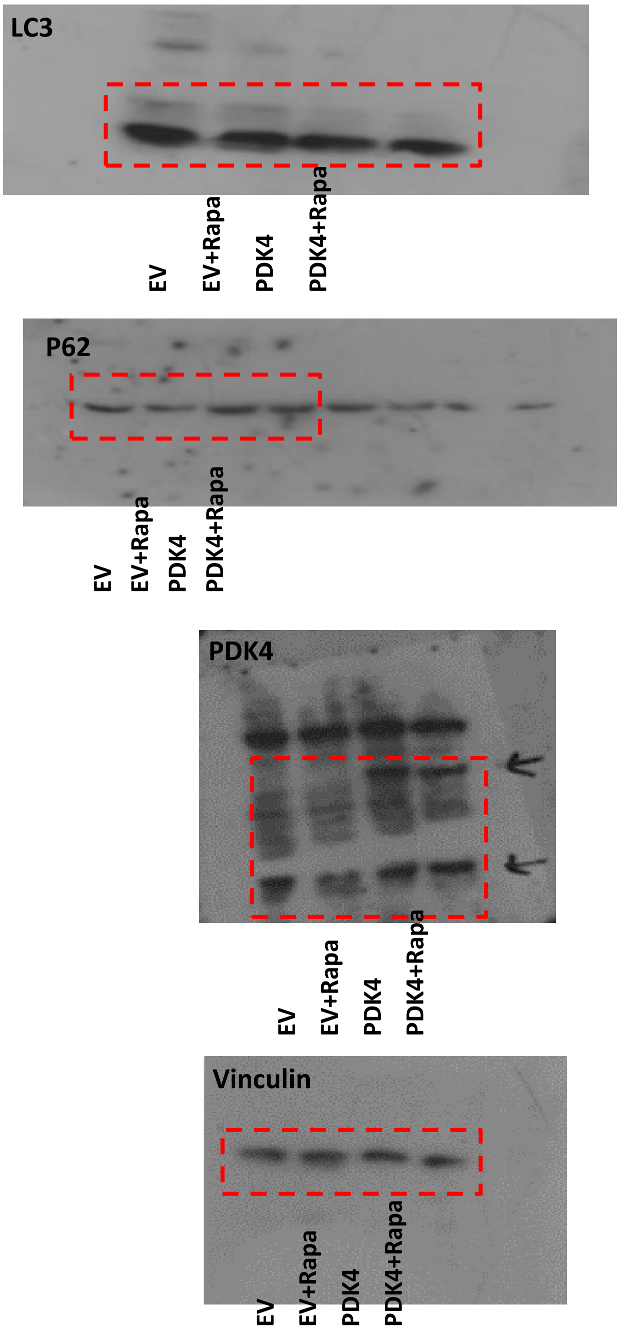

Suppl. Fig7E

		Rapa(+)		Rapa(+)
**LC3 II/I**	**EmpVec**	**EmpVec**	**PDK4**	**PDK4**
#1	1.026085	1.430895	2.212846	2.126048
#2	1.031399	1.633385	2.127385	2.120913
#3	0.942516	1.426299	1.968156	1.547383

Suppl. Fig7F

		Rapa(+)		Rapa(+)
**P62**	**EmpVec**	**EmpVec**	**PDK4**	**PDK4**
#1	1.132099	0.657594	1.926411	1.876331
#2	0.950089	0.586338	1.721323	1.637402
#3	0.917812	0.555579	1.591285	1.443111

Suppl. Fig7H

Mito within LC3 puncta/cell count

EmpVec	PDK4
4	8
2	6
4	8
7	6
6	5
2	5
3	
3	

Suppl. Fig7J

Mito within CD63 puncta/cell count

EmpVec	PDK4
3	2
3	0
3	1
2	2
3	0
4	1
	0

Suppl. Fig8

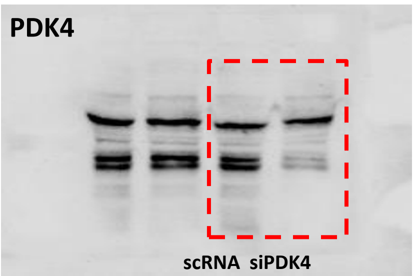

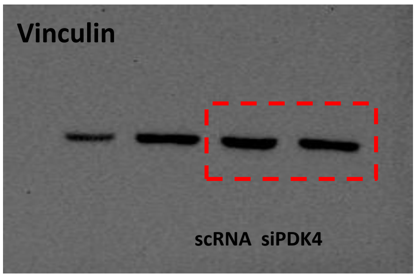

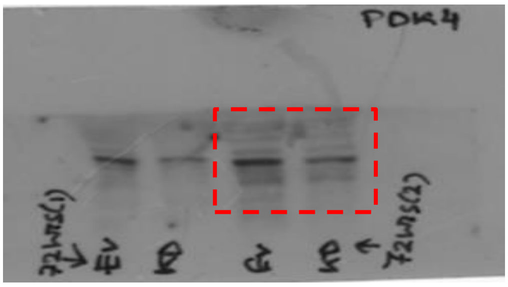

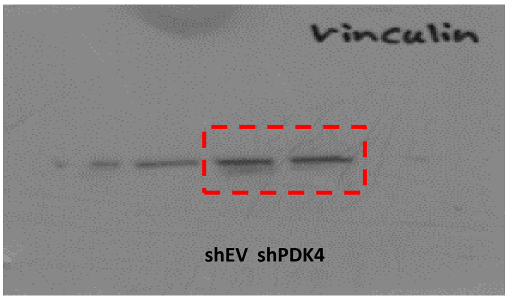

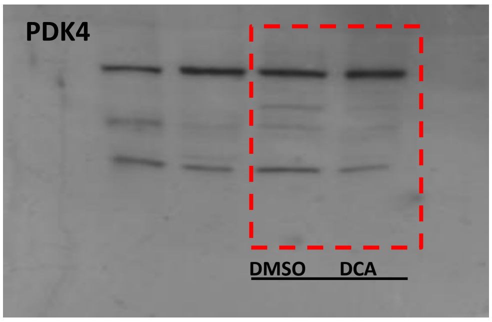

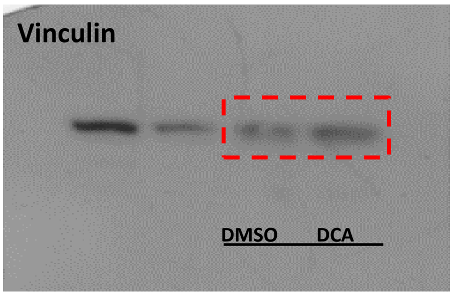

Supplement: Supplementary file 3 — Supplementary Figures raw source files [file 41419_2025_7743_MOESM3_ESM.pdf]
