# Supplementary material for: The impact of ERUPR on mitochondrial integrity mediated by PDK4
Source: Cell Death Dis. 2025 Jul 29;16(1):573. doi: 10.1038/s41419-025-07743-5 (PMC12307875; doi:10.1038/s41419-025-07743-5)
Supplement: Supplementary file 4 — Transcriptomics Data Source File [file 41419_2025_7743_MOESM4_ESM.pdf]

Fig1A

	log2FC	LOG10(pval)
HERPUD1	1.478353	17.4737
HSPA5	0.945263	9.992156
DDIT3	1.541702	9.831273
DNAJB9	0.767663	8.873992
SDF2L1	1.277063	7.255666
XBP1	0.691824	6.957902
CRELD2	0.765047	4.433214
MANF	0.544725	4.096178
PDIA4	0.466876	3.954476
CHAC1	1.265474	3.827949
AC087442.1	3.185423	3.760543
DNAJC3	0.645216	3.591149
NA	5.972139	3.58285
PODXL	-0.60407	3.551847
COL1A1	-0.6644	3.549364
HSP90B1	0.321595	3.211631
PFKFB3	-0.39969	3.210985
GALNT10	-0.56327	3.206366
COL6A1	-0.40248	2.971934
DNAJB11	0.425744	2.961938
COL18A1	-0.48295	2.95633
SLC7A11	1.68423	2.798133
DNM1P47	4.98203	2.755103
AC055713.1	0.442415	2.751051
EGFL6	-5.55271	2.74493
HSPG2	-0.63812	2.739669
WDR66	1.689958	2.710347
TRIM5	0.788505	2.681902
KCNK15	3.462865	2.636616
C11orf91	-5.42464	2.619666
PDK4	2.553132	2.584
AC093510.2	5.403776	2.578138
DDIT4	0.547029	2.573651
AC104134.1	0.755663	2.553049
COL5A1	-0.55624	2.532338
AC008591.1	1.101647	2.525407
AC114763.1	3.325149	2.520674
IFRD1	0.352563	2.501248
NARF-IT1	-2.21655	2.478125
FAM129B	-0.37019	2.46327
FLNB-AS1	0.745013	2.407588
ITGA3	-0.5513	2.404717
GBP1	0.862824	2.392801
EIF2AK3	0.47194	2.390634
CREB3L1	-1.5455	2.348491
RPL37AP1	5.333586	2.344277
SIMC1	-0.51877	2.337606

AC012181.2	3.570327	2.336838
AC010319.4	-1.41436	2.329684
BCL6	-1.96508	2.315368
AL360182.2	1.14633	2.291926
TUSC7	5.192683	2.285232
NA	1.196238	2.279237
NAV1	-0.38254	2.274912
SLITRK6	0.50729	2.270752
SPN	-5.14526	2.26341
SRPX	-1.12722	2.258192
LTBP1	-0.72682	2.24919
DLGAP1-AS2	1.217571	2.248692
AC110609.1	-4.63052	2.232616
EGR2	1.442715	2.224478
IMPDH1	-0.36514	2.213236
LURAP1L	1.083249	2.211273
PHETA1	-0.3357	2.204109
TNS3	-0.44543	2.200241
JPH3	0.815455	2.200153
RWDD4P1	5.190278	2.195559
WNT5B	-1.17191	2.189279
EGR3	1.998055	2.188209
HMGA2	-0.98959	2.17556
APOB	3.217418	2.169221
VXN	-3.66098	2.164634
NA	-1.08003	2.162229
AL358472.2	0.797967	2.160421
EPHA2	-0.38274	2.123141
MEGF6	-0.6784	2.098265
UTRN	0.539328	2.097132
NA	5.149903	2.077748
ZNF774	0.803471	2.076323
EBAG9P1	5.322044	2.072037
C17orf107	2.167767	2.06861
PRRT4	-0.28029	2.06616
TCIM	0.658208	2.065662
PDE4B	0.431114	2.06296
SCT	4.480778	2.062193
STUM	1.061107	2.060763
AP006284.1	1.514617	2.04976
AP001107.5	1.718355	2.03931
FAM131B	-0.895	2.02813
TXNDC5	0.845265	2.01424
SKINT1L	4.451405	1.999943
ATF1	0.361842	1.995051
RIPOR3	0.529236	1.991754
CDKN1A	-0.3881	1.98732
EHD2	-0.68735	1.983219
APOE	0.392248	1.977658
THOC3	0.347698	1.976606

AS3MT	4.422217	1.972105
ATF3	0.983571	1.970168
SLC38A6	-0.8776	1.959491
ADM	0.620075	1.958147
AC098934.2	2.301792	1.949365
ENC1	-0.31341	1.939979
RPL30	0.283276	1.930236
NA	5.19167	1.921414
FBLN5	-1.1059	1.914532
PRNP	-0.73811	1.912856
CNFN	1.160113	1.911542
RNU4-47P	4.380243	1.896083
POPDC2	0.512052	1.889833
LYPLAL1-DT	1.265499	1.880747
PLAU	-0.63899	1.869144
MIOXP1	5.147949	1.868092
RPS2P46	0.250197	1.865736
IL7R	-1.44548	1.858031
PSMB9	0.829868	1.85538
GLIPR1L2	5.136312	1.851979
KRT15	-5.11059	1.848193
AL356273.2	-3.36737	1.842744
GNAO1	-0.33583	1.835415
AL117209.1	-0.74503	1.818608
AC026904.2	-4.81905	1.818464
NAMPT	0.299356	1.815755
ZNF469	-0.72265	1.815264
DUSP28	-0.64881	1.812266
GFRA1	-0.92314	1.810137
DNMT3B	-0.66422	1.810121
TBC1D2	-1.10251	1.809892
NA	-1.36412	1.799562
VCL	0.367535	1.79126
NA	-0.42528	1.790058
ZKSCAN2	-0.39041	1.785318
AGBL2	-1.84478	1.778199
ACE	-0.70367	1.776879
AC018638.7	-2.16472	1.773031
AC090772.1	-3.77301	1.766354
HIF3A	0.947975	1.763321
MLXIPL	4.487398	1.76285
URGCP	-0.27877	1.754618
QRICH2	-0.845	1.750377
CORIN	1.205101	1.749598
AC022915.2	-3.32286	1.748382
CDKL4	-0.86418	1.747587
TRIP6	-0.73663	1.744308
C11orf88	-2.07428	1.743657
IGDCC3	-1.09831	1.743458
KIAA1549L	-0.56307	1.742332

GSTT2B	-2.00978	1.736542
CARS	0.292604	1.732644
CCDC68	4.225583	1.732489
ATP23	-0.83983	1.732356
AMD1P3	2.553985	1.730162
MME-AS1	-3.25346	1.728789
AC022400.1	-0.62868	1.728313
PRR13P5	2.407099	1.725141
AP000692.2	1.366201	1.722519
LPCAT2	-0.44194	1.721693
AC094019.1	-4.75908	1.720233
BANF1P2	3.717296	1.71722
GPR35	1.316658	1.713934
NA	-1.27432	1.707761
NA	-0.75458	1.701844
PCDH7	-0.9526	1.699849
MAP4K2	-0.43896	1.698441
GPR158-AS1	-4.60269	1.698304
ABLIM3	-1.10717	1.694144
AL353662.2	4.989408	1.693662
ANKRD20A17P	-1.56423	1.69357
LARP6	0.382313	1.693356
LINC02605	1.342985	1.689456
STK38L	0.336017	1.686621
AC019155.1	-4.77833	1.683251
DENND1B	-2.50816	1.681662
LSP1	-0.88359	1.675385
F2R	-0.44511	1.675256
EXPH5	1.758493	1.670664
AC009053.2	1.473712	1.666407
NA	-0.60111	1.660101
FER1L4	0.987287	1.659283
EFS	-1.95751	1.659118
CD248	-0.56872	1.656627
NES	-0.23357	1.656335
CNTN3	-0.67334	1.652102
C18orf54	-0.48673	1.651033
CSRP2	0.351983	1.646546
NBAT1	0.439412	1.645459
AC080038.1	-0.25717	1.63664
TPGS1	0.329879	1.635574
AC073130.2	-4.14519	1.634531
PLXDC1	-2.46314	1.633959
AC007040.2	-4.80116	1.633821
SLC6A2	-0.40772	1.628359
PDZD2	0.847192	1.625918
FAM30A	-1.45441	1.623024
AC010319.1	-5.01828	1.620481
TMEM26	1.697042	1.620072
KRT18P34	-2.69761	1.619707

AL583844.1	4.26744	1.618336
SPIN2B	0.627225	1.618059
AC009812.3	1.653636	1.617504
NPW	0.6273	1.617383
COL4A2	-0.28488	1.615237
KCNQ1OT1	0.730792	1.614615
PCDHB10	-0.56259	1.614548
AEBP1	-0.29854	1.612516
NCKAP5-AS2	-3.52984	1.612308
EXOSC1	0.341537	1.608375
RNU6-1048P	-4.66572	1.605396
TSEN34	0.309133	1.604976
ZMIZ1	-0.28023	1.603029
AC138028.5	-3.62218	1.600903
IFI6	0.450158	1.59823
C19orf47	-0.36838	1.597346
TRAF1	0.731509	1.592707
RPL35	0.258325	1.590716
BACE1	-0.33109	1.590473
SLC7A5	0.46608	1.590181
NUP210	-0.32526	1.58832
PRSS23	-0.37509	1.587149
NTSR1	-3.50897	1.58584
AKAP17A	0.272668	1.585407
AC109322.1	0.349968	1.584171
AL450313.1	-3.13376	1.58232
RPS21	0.295803	1.582035
AC024451.2	4.132934	1.57746
AC127070.2	-3.32445	1.572952
CRYAB	-0.93081	1.565827
PHLDA3	0.363619	1.565812
TRIM22	-1.02115	1.56525
SERPINH1P1	4.092149	1.564799
ITGA6	-0.6212	1.564747
MAPK12	-0.32242	1.564683
SLC6A9	0.601502	1.559589
XYLB	-0.57158	1.558658
C12orf50	-4.0295	1.558313
SLC16A3	-0.68852	1.558053
HSPA1A	-0.47822	1.553726
SSC5D	-0.61374	1.553621
VIP	0.439947	1.550268
RPP38-DT	1.509096	1.549951
ELN-AS1	-0.45808	1.541278
LINC02151	3.547576	1.540349
CRIM1-DT	-1.92012	1.535064
TPM2	-0.34125	1.530804
RAMP1	0.533434	1.528651
AF213884.3	2.419523	1.528261
GPR83	3.72088	1.527285

AL139339.2	-1.50652	1.525088
GDF15	0.576335	1.523825
CDH11	-0.33162	1.523735
AP002414.2	1.234775	1.523488
AEBP2	0.315055	1.523066
TMEFF2	-1.1278	1.521086
NA	0.599919	1.52064
GPANK1	0.269496	1.518545
TMEM50B	0.282396	1.514188
RTL5	-0.29275	1.509912
AC092641.1	4.609178	1.509808
LIMS2	-2.08947	1.508953
SLC38A1	0.211985	1.506568
LINC00960	-3.48127	1.503243
FASTKD3	-0.46155	1.502508
MT1G	2.517961	1.500947
AC005632.2	-2.28637	1.500802
C3orf80	-1.5653	1.498203
AL592430.1	1.309315	1.498192
CRISPLD1	0.357729	1.496331
NRP1	-0.36701	1.493523
WFDC21P	-1.00853	1.492892
AC108134.2	-4.52986	1.491901
AC087289.6	0.942271	1.488996
YAP1	-0.48595	1.488829
HMGB3P22	2.768462	1.487124
AL451064.1	-4.58656	1.486529
AL645608.7	-4.58656	1.486529
HYOU1	0.280496	1.486504
ASTN2	-0.36629	1.485647
PGF	-0.72983	1.483667
SEC14L1P1	1.200034	1.483264
ADAMTS19-AS1	0.892975	1.48245
NFYC-AS1	0.517889	1.479487
ZCWPW2	-1.42926	1.479423
DISP2	-0.53342	1.477697
PCDHGC3	-0.29844	1.477024
NA	0.969544	1.476147
HTRA1	-0.35152	1.471979
IER5L	0.391993	1.471141
CHRNB4	-0.3393	1.471061
CDK2AP2	0.449563	1.470528
SOX21-AS1	3.767969	1.470516
USP27X-AS1	-1.68997	1.467614
WARS2-AS1	0.575852	1.46673
AC100830.3	-1.39667	1.46649
DKK3	-0.3657	1.463482
AC084357.2	3.460342	1.462578
STOX1	0.780419	1.462574
TMEM178B	-0.34819	1.462196

AC104825.1	-1.31727	1.460449
AL158212.2	1.511893	1.459726
NA	-2.02726	1.459386
ALKBH3-AS1	-1.91003	1.457964
MROH8	0.376633	1.457792
HIST3H3	4.194981	1.456515
AL158206.1	1.327786	1.454523
CAVIN1	-0.35452	1.454035
ZCCHC12	1.257956	1.452644
AC079385.2	3.543693	1.452103
ZBTB49	0.485679	1.448776
AC087500.1	0.788534	1.448665
EIF6	0.259192	1.445481
APLP2	-0.31549	1.444605
CASP4	-0.9102	1.44417
LINC01481	4.049567	1.442198
WWC2-AS2	4.73591	1.44088
MYCL	2.274776	1.440131
AC009269.4	3.448325	1.439279
FDPSP2	-3.95754	1.438378
Z98949.1	-4.54017	1.438323
ZFHX4	-0.32135	1.436784
KCNJ5	-4.49309	1.434626
AL136162.1	-2.23278	1.434395
AC121493.1	2.798101	1.434101
AC009831.1	0.716705	1.431065
SQSTM1	0.327178	1.430586
WNT5A	-0.46077	1.429327
PARD3-AS1	4.488303	1.428859
BCAS3	0.335157	1.426234
C1R	0.622494	1.42507
PAPPA-AS1	-0.50215	1.421236
HDAC9	0.21662	1.419201
ACBD7	0.898909	1.419134
AL357140.1	0.561143	1.418488
ZFP41	-0.3806	1.417507
DIO2	0.765645	1.417244
GAS2L1	-0.37914	1.417169
LINC01250	-0.89319	1.417029
TFPT	0.319682	1.416279
AVPR2	2.025155	1.415819
HAUS5	-0.35957	1.415212
SEMA6C	-0.36598	1.415205
CD44	-0.37205	1.414844
TRIM52-AS1	0.685716	1.414436
AC012158.1	3.624604	1.413833
FKBP9	-0.25612	1.409646
SDC3	-0.29127	1.408464
TBCAP1	3.90423	1.407545
PFDN5	0.211403	1.406958

FLRT3	0.742244	1.406957
SNAP25	0.432767	1.406678
RPL37	0.288344	1.405362
GADD45A	0.397376	1.404195
GCNT1P1	-2.65904	1.403025
CCNA1	-1.55475	1.401087
DPM3	0.305995	1.399999
ACHE	1.490439	1.399485
AL110118.2	-4.67213	1.398119
NDUFV2-AS1	-1.34648	1.395987
AC091132.5	2.2093	1.394104
MZT2B	0.240103	1.393901
MEF2C-AS2	1.531679	1.393806
MKKS	0.279811	1.392467
RTL8C	-0.29494	1.392204
MAGEB17	-1.75428	1.391642
KCTD16	0.325313	1.391142
PPP1R15A	0.392694	1.390906
FBXL8	0.625435	1.39068
AC009226.1	2.671113	1.390581
GRAMD1B	-0.52567	1.388889
AP5B1	-0.57593	1.388172
RPS5P2	-3.94766	1.386315
CHST7	-0.83243	1.38536
AL121672.3	-4.48167	1.385014
TPRG1L	-0.37417	1.384065
BTG2	-0.39762	1.38317
NA	3.387751	1.380583
AC016949.1	1.921684	1.379714
TMEM26-AS1	3.629844	1.379091
C14orf28	1.229834	1.377701
LINC00954	3.871794	1.376483
ZNF681	-0.42427	1.375735
KIRREL1	-0.52973	1.375001
AC099850.1	0.614221	1.37466
KIT	1.011299	1.374337
CHST1	1.865642	1.374321
SLC3A2	0.314856	1.372159
GFPT2	-0.50999	1.371992
HIST1H2APS4	2.584865	1.370815
MYOZ1	1.610256	1.369712
WDPCP	0.642926	1.367607
SLC1A4	0.576481	1.366922
FAM86JP	-0.67676	1.366775
RPS14	0.210767	1.366696
MCF2	4.657015	1.366347
BHLHE40	0.576472	1.365835
FSTL3	-0.3259	1.364995
FOXD4L3	-4.45798	1.363652
CBL	-0.25947	1.362684

YWHAZP4	1.356086	1.361949
TBX20	-0.50613	1.361211
NA	-2.50465	1.360824
SLC7A14	-0.25762	1.358275
AC093726.1	-1.38406	1.358113
AC092944.1	2.448325	1.357053
YBX1P10	1.096532	1.35348
RPL37A	0.225291	1.353161
MRPL36	0.381481	1.3531
AL606468.1	4.426464	1.35229
MIR433	-2.71168	1.351417
METTL6	0.340791	1.350753
CA8	2.542484	1.345692
MATN2	-0.6167	1.344288
UGT3A2	-1.03305	1.3427
SYN3	4.620946	1.342488
RF00564	-4.61774	1.341331
AL645608.1	4.42935	1.341181
AC018690.1	-1.39443	1.340882
SEL1L	0.240559	1.339655
PLEKHA6	-0.22382	1.337457
ERAS	4.900543	1.337109
AL139095.2	1.477437	1.33679
HOXD9	-0.34637	1.336347
BORCS6	0.426279	1.335231
ROR2	-0.32623	1.334381
PMAIP1	0.361971	1.333439
TMED7	0.288308	1.333003
SH2D3A	-1.81429	1.3294
SLC35E4	0.537365	1.329039
NA	-4.42143	1.32858
CDKL1	0.661798	1.326909
TMEM184B	-0.40269	1.324237
AC027307.2	-0.49246	1.323817
AC055733.1	4.881761	1.323562
AC080078.1	4.881761	1.323562
PLXNA2	-0.22538	1.322025
AC026470.3	4.433995	1.322008
KDM5C-IT1	4.600595	1.321939
APTR	0.429941	1.321672
AC026803.1	4.378253	1.317312
ARMH1	1.129184	1.317179
LINC02492	3.887982	1.316219
SHANK2	-0.49559	1.316145
TBL1X	-0.3473	1.313677
AF129075.2	0.751795	1.313586
KRT18P59	-4.40878	1.31352
GPRC5D	2.501155	1.311383
CHRNA5	-0.29201	1.310836
RPL10P9	1.019767	1.310644

CD93	-3.77635	1.308581
GCA	0.403531	1.308104
TMED10P1	-1.08472	1.307795
CSF1	-0.37298	1.307586
FAM184A	0.42268	1.307453
LINC01786	3.069993	1.307174
CEBPB	0.438921	1.307099
LINC02458	3.818098	1.30621
PSG4	-0.80366	1.305979
LINC01578	0.296695	1.305376
ITGB2	1.370314	1.30535
SLC39A14	0.262511	1.30492
CXorf40B	0.367937	1.301685
GDF9	0.28258	1.301514
RPS18	0.207478	1.300131
AC127024.4	-1.90493	1.29902
NA	-1.86915	1.298758
C17orf53	-0.54188	1.294776
AC092683.1	-0.73591	1.293289
AP000925.1	4.403053	1.292804
HRH2	1.185221	1.292787
KLHL15	-0.34207	1.292551
DRP2	-1.81055	1.291618
RPS27	0.233564	1.291553
AP003119.2	-2.41012	1.290321
AP001107.1	0.25793	1.289728
FDCSP	2.267026	1.288745
PEX3	-0.446	1.287814
NA	-4.04917	1.286933
SUGT1P1	1.347292	1.285231
SSR4	0.221528	1.28479
CPNE4	3.984896	1.284651
AC026369.2	2.14341	1.284368
MAP1B	-0.24529	1.284248
NA	0.692001	1.28155
MICU3	0.441004	1.279852
C2CD2L	-0.3763	1.27893
ARC	1.232039	1.278904
PDE5A	0.341876	1.27799
DDX11L8	-4.36529	1.277662
AC106870.2	3.313913	1.277498
RGPD8	-1.24512	1.277386
NA	2.610733	1.2773
EBF1	0.927377	1.276422
AC106794.2	-2.8026	1.274838
MSC	0.507629	1.274451
CMTM7	-0.30425	1.274374
SMIM6	2.321708	1.273882
LTBP2	-0.29146	1.273448
EIF3J-DT	0.370138	1.268893

MFSD2A	0.558851	1.266458
ZNF430	-0.47272	1.266092
RN7SL336P	3.249427	1.264564
ERO1B	0.439504	1.264216
FAM222A-AS1	-0.48419	1.260019
AC010904.1	4.53928	1.259575
AC019171.1	3.84444	1.259452
AC116667.1	-1.23411	1.25887
NA	-1.73324	1.258435
ASRGL1	0.352453	1.256011
MYADM	0.313667	1.255832
PPP1R18	0.261723	1.255649
F2RL2	-0.69113	1.254516
AC141586.3	-0.34989	1.253383
NA	-3.73434	1.25161
EYA4	0.824641	1.25136
AC098487.1	2.220168	1.251351
RPS28	0.237206	1.251198
SERPINF2	1.348628	1.250662
FADS3	0.360088	1.249626
AL365440.1	3.754386	1.249445
CACNB4	-0.4641	1.249193
DDIT4-AS1	0.422142	1.249121
AC093677.2	1.028169	1.248683
AL163636.1	-2.54912	1.248046
AL158835.1	1.058402	1.247971
SHROOM3	-0.23305	1.247214
GPR158	-1.33724	1.246144
AL009181.1	0.430125	1.245768
TRERF1	-0.45402	1.245575
DNAJB5	0.270669	1.245175
LRRIQ4	3.553333	1.244803
CRABP2	-0.3256	1.239283
TMEM100	0.411724	1.238526
CD274	0.497756	1.238388
AC026774.1	1.129543	1.237761
AC012087.2	3.96564	1.236827
AC090229.1	-1.66561	1.236156
DLX3	3.355485	1.235996
RPS3P6	-4.27048	1.235826
AC008731.1	2.264757	1.235476
MTCO3P24	4.499378	1.234923
FAM13A	0.422228	1.234636
RNF182	0.464597	1.234016
TTC21B-AS1	0.832111	1.233906
ADAMTS1	0.287932	1.232244
RNF26	-0.27075	1.232102
CCDC85A	-1.14377	1.231067
HYAL3	-0.56715	1.230748
RPS20P22	-2.20972	1.22863

LOXL2	-0.23425	1.227606
CELF5	0.550436	1.22687
AC004477.1	0.637027	1.225216
AL157935.1	-0.79237	1.222631
METRNL	0.440127	1.22206
CDK5R2	-0.37884	1.221472
CRB1	-1.03195	1.220957
AC005829.1	2.214335	1.220364
COL18A1-AS2	-4.46935	1.218909
RPL5P34	1.234501	1.218334
AP000487.1	-0.39787	1.218088
DERL3	1.448813	1.217974
DOP1B	0.52215	1.217954
REX1BD	0.276222	1.217535
AP003730.2	-3.70388	1.216954
LINC01852	-1.32507	1.215748
FTH1P16	-3.75325	1.215655
AP001107.9	0.570797	1.215545
GRIN2D	-0.8691	1.21418
AC009996.1	1.560373	1.214021
AOPEP	0.264106	1.212939
RNU6-1289P	4.333731	1.212852
ST6GAL2	0.315907	1.212806
SEZ6	0.492295	1.212629
RPL35P3	4.51296	1.212579
CASP10	3.748904	1.211713
TUBB8P1	-2.22919	1.211326
RP9	0.303587	1.210431
PRR5-ARHGAP8	-4.46059	1.209987
RNU6-828P	4.468258	1.208857
NA	4.476216	1.20737
AC079781.2	-3.70266	1.207041
CEBPD	0.489415	1.206706
CLCC1	-0.23494	1.205561
SHBG	1.841675	1.204138
FAM222A	-0.20695	1.203911
TLL2	1.41199	1.203333
FO393422.1	-3.97954	1.202978
AC092040.1	-2.9078	1.20261
MBNL3	-0.37131	1.201861
TMEM132C	4.259153	1.200489
PKP3	-1.67648	1.199783
AL353658.1	-3.72571	1.199598
EIF2S2	0.220451	1.199138
PPP1R32	-1.2253	1.198575
HRH3	0.512532	1.198434
NA	1.16273	1.19816
AP003396.1	-0.41073	1.197572
LINC02094	-3.66893	1.197545
ARHGAP27	1.001275	1.197494

LINC00682	0.382777	1.197277
TSPYL2	0.284954	1.196221
NECTIN1	-0.29785	1.193808
CYB5D1	-0.33344	1.193702
RRAGB	0.40329	1.193163
AL021331.1	-4.43061	1.192993
AP001033.1	-4.43061	1.192993
AL022394.1	-3.86333	1.192657
AL807757.2	4.347713	1.192619
AL139094.1	1.620762	1.1924
SLC9A5	-0.31572	1.192389
SYNPO	-0.26336	1.190472
EFR3B	-0.39889	1.190085
MYLIP	-0.38587	1.190005
AC092720.2	3.736739	1.188548
SCMH1	0.24113	1.188117
IRAIN	-3.70215	1.187277
CORO2B	-0.87288	1.186708
RF00019	1.506301	1.186603
PSME2P2	-2.25861	1.184491
RPS24	0.185216	1.183408
AP003352.1	0.204065	1.182011
LINC00574	-1.85511	1.181701
SOX13	-0.53325	1.179825
LAMP5	-0.33092	1.179675
LGI3	2.64145	1.179257
AC104964.4	-1.16293	1.178665
PLAAT3	0.326279	1.178655
RPSAP54	1.92334	1.178338
RPL41	0.208606	1.177976
TCEANC	-0.7102	1.177144
PCP4	1.834389	1.176811
AC098591.1	1.3001	1.176381
DLX1	4.68916	1.175548
AC008813.1	4.68916	1.175548
MRPS18A	0.29792	1.17535
AC231981.1	0.322537	1.174824
FNDC5	0.419208	1.174246
GALK1	0.366358	1.174242
PROKR2	-3.63664	1.174096
PDP1	0.310207	1.173788
LINC00205	-0.34324	1.173679
NFIC	-0.2252	1.173494
STON1	-0.4524	1.173177
HPD	2.404008	1.17231
CRABP1	0.32551	1.171608
CAD	-0.23763	1.170776
ATP2B4	-0.24467	1.170613
DOCK3	1.694868	1.170524
AL031283.2	1.887815	1.170233

CCDC169	-0.67281	1.169428
NA	2.014586	1.168877
AC079594.2	-3.10136	1.168837
CALD1	0.175979	1.16856
FBXW7-AS1	0.741868	1.168413
CASP1	-1.28029	1.168296
AP006623.1	-0.75711	1.168266
RN7SL838P	-4.47534	1.167644
AL118506.1	-0.99356	1.166274
AC244033.1	1.424719	1.164234
AC138035.1	-0.72925	1.164032
AC069236.1	3.833408	1.163525
PID1	-3.66831	1.163235
PEX12	-0.36784	1.162838
AL133406.2	-0.58621	1.162078
AL356489.1	4.414163	1.161844
ICK	-0.25212	1.161737
DYNC1I1	0.671799	1.160766
PRKCQ-AS1	1.283132	1.159779
RPL10P7	-2.41885	1.159745
AL359091.4	-1.9289	1.159399
DTX3L	-0.33991	1.158397
C3AR1	2.538817	1.157949
ST3GAL5	-0.41616	1.157264
RIN2	-0.77803	1.157142
WEE2	-1.45523	1.156862
AC117500.2	2.569474	1.156715
ADAM33	-1.33196	1.156632
FAM174B	0.398779	1.156293
EPCAM	-1.22819	1.155504
SERTAD1	0.497345	1.155465
PDGFA	-0.62509	1.155067
SUMO4	-3.42853	1.154857
CMAHP	0.33283	1.154685
C1orf52	-0.26169	1.154038
U91328.1	0.640475	1.15313
TNFAIP3	-0.4453	1.152848
HCN2	-0.35297	1.152581
AC024270.1	3.818806	1.152008
CORO2A	0.506928	1.151617
POLG	-0.22173	1.151609
ITPR2	0.225059	1.150608
CYP20A1	0.420616	1.150491
SLC33A1	0.254534	1.150243
COLGALT2	-0.46462	1.149942
TUBA1C	0.225063	1.148519
GLB1L2	-0.41097	1.148293
HOXC6	-0.42182	1.147434
AC104113.1	2.397479	1.146121
MGP	-0.39204	1.145936

PSMB8	-0.51436	1.145289
AC138028.1	-2.72497	1.144536
FBXO8	0.193251	1.14387
NCR3LG1	0.311953	1.143702
ZAR1	-0.88161	1.143338
DUSP1	-0.32083	1.142352
ERCC6	0.324697	1.141896
NA	0.440596	1.141803
MYO10	-0.34125	1.141767
NFKB1	0.267295	1.141387
TNFSF4	-0.69233	1.140715
MTCYBP18	-2.22543	1.140057
AADACP1	-4.16967	1.139569
NPAS3	0.727073	1.138466
TBC1D12	-0.32128	1.137883
SNORD91A	3.173025	1.137655
PLCL2-AS1	1.704181	1.137369
AC073046.1	0.994986	1.136961
SPRY4	-0.29873	1.136164
AL121748.1	-0.50394	1.136124
INHA	-1.65053	1.135781
AL138963.3	0.170365	1.135104
ASPH	-0.21787	1.134589
AC104667.1	-2.70256	1.133315
CLDN9	-3.15681	1.13102
ENO4	-1.74458	1.130819
CYP2C8	-3.62545	1.130776
MAP2K3	0.383907	1.129999
TNFSF9	-4.1528	1.129913
AC090519.1	3.643741	1.129784
AC138207.6	3.785382	1.128918
TNF	-1.65456	1.128413
ZC3HAV1	-0.27012	1.128282
NA	0.535211	1.127108
HNRNPCP2	-0.94432	1.126832
CIAO2B	0.237285	1.126544
CHDH	0.649452	1.126412
AC009955.2	-4.35035	1.126321
GTSE1	-0.23401	1.12417
CRYBB1	-1.49401	1.124049
ARR3	-1.66553	1.123965
SLC9B1	0.302624	1.12379
LRRC66	-4.23843	1.123514
ZDHHC19	2.034298	1.122944
AC005544.2	-0.40519	1.122506
TERT	-0.37247	1.1216
HMGB3P4	3.785695	1.120806
SRD5A3	-0.34438	1.120682
PTPN14	-0.30256	1.12054
NA	-2.09106	1.119224

C4A	1.515771	1.118798
AC025279.1	-1.27008	1.118291
AC005534.2	-3.23748	1.118179
ZCCHC14	-0.23161	1.118095
RELL2	0.310242	1.117673
ADGRE1	-1.14397	1.117661
TTC41P	1.57728	1.115179
SP100	-0.45132	1.114767
AC112187.3	-4.13671	1.114672
GOSR1	-0.22771	1.114628
AHNAK2	-0.68694	1.114564
TSLP	0.630365	1.114452
AC022239.1	-0.86293	1.114283
OGDH	-0.21569	1.113115
TWSG1	-0.2415	1.112693
OMP	-3.12373	1.112092
CYP26B1	-2.49678	1.111986
CD300LF	-3.55498	1.111844
LINC00623	1.447495	1.111381
C4orf47	1.645917	1.111265
ARL13B	-0.32841	1.111188
AC026369.1	1.380731	1.110454
AC010618.4	0.390177	1.109328
AC092069.1	0.207117	1.10919
SSH3	-0.39013	1.108337
ANKRD12	0.289025	1.108287
LRMP	3.209518	1.108065
NA	-4.15567	1.1077
AC026358.1	-4.15567	1.1077
ITPK1	-0.28521	1.107578
RPL5P17	3.374652	1.107517
ANAPC1P1	-4.12235	1.106955
NA	-4.12235	1.106955
AL356441.1	4.371574	1.106835
CHST3	-0.28532	1.106462
PARVA	-0.5987	1.104581
NA	-3.54273	1.104555
PRIMA1	-0.29217	1.104475
PLPPR3	1.041062	1.104181
ACVRL1	-0.55931	1.103579
CAPN14	1.616828	1.103479
LINC01103	3.642566	1.099542
ADAMTS13	0.411606	1.098841
RPS10P5	3.567782	1.09864
DRG1	0.243943	1.098555
SLC25A36P1	-3.63349	1.096605
MARC2	-0.50966	1.096245
MYH11	-0.83874	1.09615
PRR19	0.319727	1.096089
NA	0.438255	1.095627

AC104118.1	-0.93491	1.095511
SELENOK	0.231026	1.095012
SENCR	-2.20191	1.094975
ARID5B	0.235809	1.094585
AC007552.2	3.596922	1.094524
ZFAND4	0.436101	1.094401
RIC8B	0.234205	1.09412
MARCH4	-0.30779	1.094054
RPS4XP1	-2.64066	1.093857
ADAMTS12	-0.59426	1.093607
ATP13A4	3.733231	1.093059
TMEM41B	0.231081	1.092543
AC018878.1	3.731821	1.091326
AQP4-AS1	3.731821	1.091326
DIO1	4.17466	1.091269
TUBBP2	3.352335	1.091152
RIN3	-1.84926	1.091031
PCAT7	3.197586	1.08464
FUT8	-0.27945	1.08461
NA	-0.33332	1.084506
SVOPL	1.988527	1.084032
AC011498.1	2.566036	1.083918
ZNF667-AS1	0.320545	1.083555
FAT3	-0.61856	1.083271
TTC12	-0.39528	1.083234
AC005034.5	0.579593	1.081927
AC119396.1	3.017093	1.081382
AGRN	-0.25356	1.08135
TAF9BP1	3.816947	1.081217
CANT1	-0.19955	1.080821
AC026436.1	1.808247	1.080611
RPL10AP1	4.158839	1.080071
AL049828.1	4.158839	1.080071
DCLK3	0.457463	1.079927
WDR70	0.261176	1.079852
NA	-1.85628	1.079351
AC005304.1	4.330454	1.078634
AC097724.1	4.330454	1.078634
KIAA1614	-0.30419	1.07844
TMEM260	-0.30172	1.077518
PAPPA	-0.35385	1.076672
NA	3.81185	1.076225
CAMKV	0.288782	1.075397
AC130709.1	-4.28274	1.07536
CD38	-3.01132	1.075332
GNAS-AS1	0.783122	1.074593
NPM1P35	4.361698	1.074271
AC009108.3	4.361698	1.074271
AC138473.1	1.042313	1.073079
SYT14P1	3.721196	1.072998

AC106028.3	2.302666	1.07289
SLC8A3	-0.30983	1.072062
MOCS3	-0.27133	1.07203
NLRP3	-4.10675	1.071995
HGF	0.441375	1.071785
NA	1.985638	1.071403
CCDC124	0.217917	1.071327
NA	1.609756	1.070625
TMSB4XP6	-3.55353	1.069569
LKAAEAR1	1.446793	1.069372
UFSP2	0.265277	1.069173
AC010463.3	-1.65624	1.06905
ZNF318	-0.24837	1.068896
ADM2	0.631064	1.068859
ACYP1	-0.48991	1.068801
PFN1P1	1.230102	1.06853
PTK2B	-0.55501	1.06827
MIR320A	4.298441	1.067777
NAV2-AS1	-0.35619	1.067664
GRM6	-2.36961	1.067009
CASC15	0.244233	1.066633
AC090772.3	-0.717	1.066584
RNU6-262P	4.290045	1.066575
ZNF423	-0.25777	1.066443
PREB	0.201648	1.065942
IGSF3	-0.21679	1.065498
AC120114.1	0.860263	1.065241
AC016571.1	2.020817	1.064931
SLC2A10	-0.43029	1.063466
AC004982.1	1.555474	1.063462
PTMAP1	1.457758	1.063205
HOOK1	0.479294	1.062684
AL109955.1	0.637453	1.06199
TNS2	-0.43865	1.061901
DYRK4	0.407869	1.061312
PLXNA4	-0.31051	1.061179
LAMB2	-0.21102	1.0611
AC097382.2	-0.20568	1.061053
RFT1	-0.2594	1.060977
ADAMTS2	-0.33904	1.060318
AC105339.2	0.416461	1.060211
SMTNL1	1.565164	1.060086
AC008894.2	0.267082	1.060044
DLL4	2.797669	1.060023
INSRR	-0.26561	1.059592
PIH1D2	-0.79962	1.059429
TBC1D9	-0.25039	1.058978
TMEM135	0.343108	1.057454
HLA-B	0.445741	1.056363
AC008443.2	0.441855	1.055681

SORCS2	-1.04326	1.055298
ADAMTS17	0.453841	1.054878
EIF3CL	0.834104	1.054807
NA	4.272712	1.054804
HCG27	-1.96444	1.054383
COQ6	-0.28172	1.053226
ZNF846	0.659824	1.053199
SPDYE18	-1.48081	1.052962
SSBP2	0.321799	1.0529
TGFB2-AS1	1.895914	1.05271
AC079781.3	-2.36609	1.052227
ANKRD34B	2.824124	1.052195
SIM2	-0.93386	1.051491
GPR155	0.308481	1.051128
AC044787.1	1.984229	1.051059
FCF1P7	4.327262	1.050563
AL162430.2	4.327262	1.050563
AC064836.1	2.947242	1.050279
BRD3OS	-0.23775	1.049045
TSPOAP1-AS1	0.206452	1.049044
AC020909.1	0.324739	1.048981
CANX	0.149893	1.048845
DENND4A	0.256352	1.048769
CDRT15	-1.61221	1.048459
SDC2	0.234405	1.048198
PPP2R5A	0.308905	1.047331
AL008721.2	-1.97183	1.047207
PITPNA-AS1	0.352521	1.046397
FRMD1	3.683215	1.046139
MRO	4.254013	1.045952
FCRLA	-1.99149	1.045763
EEF1A1P14	-3.2375	1.045329
OSBP2	-0.659	1.04399
RPS7	0.187792	1.043728
JAG2	-0.56484	1.042103
HSD17B8	0.574399	1.042044
ABCB9	0.204086	1.041717
FO704657.1	-0.58646	1.041314
C1QTNF12	-2.43491	1.041267
FOSL1	-0.65956	1.041249
PDF	0.36476	1.040909
ZFP36L2	0.313217	1.040888
GPR180	0.329276	1.04027
HERC5	0.376243	1.040134
MYBPHL	4.056419	1.039825
RPL27	0.192247	1.038822
RPL21P132	-3.47067	1.038695
GADD45G	0.684	1.038324
KARSP2	-3.27232	1.038027
GNPTAB	-0.23119	1.037021

EPHA10	0.756156	1.036723
NA	-1.34969	1.035535
LINGO1	-0.69927	1.035473
ZBTB11-AS1	0.373447	1.035252
AC105020.4	1.037009	1.034463
AC005291.2	0.643104	1.034309
RHPN1	-0.36695	1.034233
CTRC	3.174535	1.033435
KYNU	-3.53514	1.033372
RPSAP14	-3.51747	1.033133
AL132709.1	-3.44374	1.032807
FAM163A	-0.18934	1.032726
FAM110A	0.449684	1.032048
NA	1.573306	1.031968
LRRC32	-0.53881	1.031811
BX005266.1	3.225256	1.031163
NDUFAF4P3	-2.60066	1.031016
FMN2	-0.33747	1.030443
STK10	-0.22694	1.029866
ROMO1	0.249978	1.029838
SOWAHA	-3.44374	1.028898
AP003084.1	2.047061	1.028502
GAL	0.220068	1.028333
RPS20P15	-4.22423	1.027725
AL121759.1	-4.22423	1.027725
CYBRD1	-0.33322	1.027549
SLC9A3R2	-0.37009	1.027012
AL158071.1	4.08328	1.026609
CACNG2	-1.04404	1.025851
ZNF257	-1.44921	1.025312
DOK7	3.534829	1.025167
MREG	0.458222	1.024917
AL359853.1	-0.33134	1.024152
TRAM1	0.202654	1.024055
CYLD	0.204776	1.023662
NA	-2.29666	1.023538
AC055854.1	4.25119	1.02337
AL024498.1	-0.92097	1.022995
LINC01085	-2.35098	1.022873
CACNG5	-2.60618	1.022804
NKPD1	-1.42092	1.02259
ERC2	0.908258	1.022306
LRRC37BP1	-0.36542	1.022252
AC137695.1	-1.85947	1.021992
ATP8B4	2.281318	1.021899
AC087752.4	-1.04565	1.021069
SLIT2	-0.40677	1.020897
C1orf53	0.758777	1.02084
PAX5	-0.35955	1.020726
AL136968.1	4.247822	1.020288

AL138921.2	-0.76423	1.019989
MTCO3P15	3.644201	1.01985
HMGCS1	0.285415	1.018394
AP003465.1	0.369284	1.018312
MRE11	0.291038	1.01819
AL596330.1	-0.81458	1.017931
NA	2.657349	1.017565
ESR1	1.681784	1.017549
ADAM12	-0.33302	1.01749
LARGE2	-0.8934	1.017325
NRIP1	-3.70793	1.016934
NARF-AS1	1.573353	1.016805
NEBL-AS1	2.610885	1.016561
TNXB	-1.48198	1.016392
RPSAP18	2.97839	1.016219
LINC02599	-3.61334	1.015698
AC007383.3	0.456503	1.01544
AC083967.1	-1.06805	1.015321
SAT1	0.373256	1.015171
SNRPD3	0.195677	1.014665
KIAA0753	0.218299	1.014323
EXTL3	-0.1975	1.014185
FAM204A	0.27435	1.013616
P3H2	-0.77444	1.013551
SPARC	-0.21727	1.013405
MTERF2	0.266973	1.013341
UBXN7-AS1	0.936376	1.013238
SPDYE1	0.676436	1.013207
BRWD1-AS2	1.69337	1.012049
CACNA1C	-0.42053	1.011934
RPS11	0.190262	1.011323
NTRK3	-0.41578	1.011314
AL135910.1	-4.2174	1.011147
SZT2-AS1	-0.7403	1.011113
AC006111.2	0.778312	1.010777
ATF4	0.170308	1.010554
GAPDHP65	-2.13354	1.010025
TMCO6	0.310102	1.009927
MIR3136	3.08662	1.00982
SLA	0.883381	1.0094
ALOX12-AS1	-0.82071	1.009149
NLRC5	-0.5492	1.008632
INCENP	-0.23093	1.008545
AC027176.2	3.028331	1.008518
ZNF468	0.372931	1.008246
TNFRSF11B	-1.65608	1.008205
TRIM74	-1.78069	1.00775
SDC1	-0.21183	1.007668
TMLHE	-0.3812	1.007382
AC145285.5	-4.18739	1.006972

DENND1C	1.530509	1.006741
AL109659.2	-3.07461	1.006478
MTCO1P40	1.750169	1.005694
FAM218A	3.609924	1.005623
ABCA5	0.3545	1.005555
BATF2	3.479248	1.005413
C3orf20	-3.9903	1.004716
C12orf57	0.217433	1.004574
ATP6V0E1P1	-4.18365	1.004454
AC069444.1	1.28026	1.004247
ADCK2	0.443861	1.004055
ATP2A2	0.205133	1.003478
AGO1	-0.19388	1.003147
RN7SL145P	4.015032	1.002488
IRX3	0.512702	1.002297
ELL3	1.542037	1.002281
AARS	0.23534	1.002225
INO80B	0.237777	1.002072
AC007406.5	-0.36346	1.001844
FZD5	0.915753	1.001771
COL1A2	-0.64605	1.00166
MIF	0.283723	1.001466
ITIH5	-4.23686	1.001324
RNU6-790P	-4.23686	1.001324
MIR4777	-4.23686	1.001324
IER3-AS1	0.586296	1.001257
COL23A1	-1.44458	1.001126
PRKCQ	1.336456	1.000858
WDR64	-4.01023	1.000337
AC005841.1	0.237273	0.999997
AL132656.1	1.041268	0.999628
AC080023.2	4.042464	0.998653
AC007639.1	1.399405	0.998523
ABCA3	-0.19764	0.998492
AL122017.1	-4.00712	0.99823
NDUFS3	0.200317	0.998217
PIGZ	-0.74188	0.998008
NUCB2	0.305168	0.997773
AC005837.1	-0.84101	0.997007
HM13-AS1	1.354181	0.996173
ADAT1	-0.21557	0.995921
ITGA4	0.308969	0.995709
AC114760.2	3.461027	0.995672
AC009563.1	0.751764	0.995402
HCFC1	-0.23999	0.994987
NA	-1.31198	0.994917
SF3B4	-0.20219	0.994152
AC116609.1	1.996867	0.993663
FNIP2	-0.26122	0.993171
SETD1B	-0.36922	0.992631

AC015712.2	-0.42089	0.992212
AL139099.1	0.22001	0.992156
MDC1	-0.22344	0.9919
KRT18P4	-4.22215	0.991663
NUP188	-0.20657	0.991454
SFT2D3	0.243237	0.991025
RTEL1P1	4.237269	0.990924
AC074117.2	-3.51563	0.990526
TRMT5	-0.25574	0.990316
MARS	0.190387	0.989849
SHISA7	1.739503	0.989101
VMAC	-0.49776	0.988468
NA	2.089968	0.988369
SNRK	-0.22618	0.987834
SNORA47	-2.52471	0.987359
LAMC1-AS1	1.747279	0.987094
AJAP1	4.414037	0.986726
MAPKAPK5P1	4.414037	0.986726
AC104241.1	-3.97544	0.986574
RPL39	0.169682	0.986565
AL121983.1	4.413766	0.986378
CCNH	0.246801	0.986234
NELFE	0.181163	0.985981
AL353807.1	-2.57029	0.985336
AC008764.3	-0.38636	0.984859
PLAC8L1	1.223812	0.98484
AC022400.4	-1.71083	0.98467
AC012313.4	-1.30864	0.984151
AL137026.2	4.22618	0.98334
AL359918.2	-1.53828	0.983057
AC010333.2	-4.15121	0.982645
LTB	0.966762	0.982447
AMDHD1	0.891999	0.982193
AC066612.2	-3.57111	0.981928
RAB40A	-0.60131	0.981843
AC005954.2	2.260947	0.981062
AL158827.1	4.221825	0.981029
SUCLA2P3	4.221825	0.981029
ENOX1-AS1	4.221825	0.981029
RN7SL376P	4.221825	0.981029
AC024475.1	-2.91012	0.98069
IGHV3-47	1.150315	0.980603
AC087222.1	1.359826	0.9806
AL592146.1	0.498694	0.980586
AC131971.1	1.620672	0.980164
GNAI1	0.217325	0.979982
SNHG10	0.315636	0.979949
SLC38A10	-0.20441	0.979841
YARS	0.19441	0.97961
MRAP2	-0.3009	0.979393

ARHGAP29	-0.57717	0.978794
CHRNB2	-0.20652	0.978465
CMIP	-0.20683	0.978451
CORO1C	0.202818	0.977943
BRI3P1	-2.40728	0.977664
FAHD2P1	1.768343	0.977624
NLRX1	-0.39878	0.977539
RPL17P36	2.390512	0.977259
ING4	0.223446	0.977047
NDUFA7	-1.30623	0.97697
PGM2L1	0.363031	0.976957
AL133346.1	0.746884	0.976943
NA	0.522988	0.976767
AC021074.1	3.049726	0.976536
MEX3B	0.208888	0.975837
MKI67	-0.17438	0.975729
CPS1	-0.6041	0.975638
RPL8	0.160736	0.975041
NPY	0.68342	0.974496
SEMA3G	-1.10189	0.974469
TMEM185B	-0.32008	0.97429
LRRFIP1P1	-1.19285	0.974221
PLEKHA5	0.452072	0.973528
LSM4	0.173378	0.972596
AC026401.1	-0.80938	0.972468
RNF113A	0.303931	0.972365
RAD54L2	-0.2182	0.972134
DUSP5P1	-1.06668	0.971986
NOTCH1	-0.49617	0.971883
AC145285.1	3.567052	0.971474
AC007954.1	3.567052	0.971474
AL391839.1	-2.61834	0.970561
PCDHGB2	-0.71685	0.970247
AKR1E2	0.485347	0.969994
NA	3.070675	0.969953
ZHX3	-0.28663	0.969518
PCDHB15	-0.87678	0.969504
AF117829.1	0.464283	0.969277
KPNA1	-0.19697	0.968877
TMEM156	-2.19041	0.968513
ZNF836	-0.53924	0.968299
AC024267.4	1.630677	0.968102
AC005597.1	-2.71451	0.96797
NA	-2.06524	0.967678
IKZF1	1.307087	0.967666
ARFGAP3	0.310407	0.967416
EVPL	-1.84954	0.967271
AL121899.1	1.420877	0.967263
PAK1	0.353002	0.967146
MXRA5	-0.78113	0.96695

AC005838.1	-4.13471	0.96671
CEP164	-0.22249	0.966598
DCUN1D4	0.232706	0.966382
RPL10A	0.164677	0.96607
AC005740.3	0.554703	0.96605
PFKP	-0.18559	0.96542
CTSH	0.572556	0.965263
LMNTD2	0.810459	0.964531
NTHL1	0.258231	0.964376
RPL13A	0.172865	0.964218
ARHGEF38	0.416103	0.964207
ACTG1P3	-1.90776	0.963858
NA	0.922108	0.963645
FKBP15	0.210312	0.963115
LRIG1	-0.32473	0.962801
AC112503.1	-3.97114	0.962251
AP003064.2	2.05241	0.962077
LINC00511	-0.45712	0.961296
ADAM29	4.19158	0.961032
AMHR2	-2.88879	0.961018
PDIA3P1	0.508822	0.960854
SPATA9	0.963154	0.9607
MIR22HG	0.360715	0.960643
AP000915.1	-3.0219	0.959964
BCL2L14	2.181924	0.959805
IL1R1	-1.34581	0.959603
AC104109.2	1.708379	0.959546
TAOK3	0.214504	0.959388
AC107954.1	4.188374	0.958975
ASTN1	0.54596	0.958395
SPDYE3	0.408374	0.957566
AVEN	0.325186	0.957529
AL157400.4	1.991337	0.957454
AC012615.1	0.335565	0.957279
FAU	0.179023	0.956964
SYTL1	0.840541	0.956936
AC107959.1	-0.33947	0.956721
CDK2AP2P3	3.950468	0.956214
OGFOD1P1	3.950468	0.956214
AC025575.1	3.391657	0.95612
ECHDC2	-0.9707	0.956016
AC008105.1	-1.40152	0.955812
BTBD7	-0.20678	0.955812
RPL29	0.172522	0.955589
DOK4	-0.1552	0.955227
AL162431.1	-2.83668	0.955007
AC233723.1	3.050893	0.954967
AC211486.1	-1.39877	0.954705
CCDC93	0.191536	0.95457
COL6A2	-0.20585	0.95449

AC006466.1	-4.34588	0.95422
AC092123.1	-1.35064	0.954218
AC108463.2	-3.40251	0.953393
ATP6V1C2	0.155604	0.953373
USP53	0.329097	0.953189
AC129492.6	2.614586	0.952614
ZNF214	0.553137	0.952326
HDAC2-AS2	-0.76738	0.952124
MRPS18B	-0.21611	0.951011
SEMA6A-AS1	-0.65871	0.950623
NDUFAF8	0.200107	0.949393
AC104241.2	1.885557	0.949393
RNU6-762P	2.253232	0.949335
MRGBP	0.24282	0.948614
PTCHD1	2.936361	0.948606
KIAA1549	-0.19801	0.948475
BOK	0.275093	0.948375
FAT1	0.226822	0.948257
SNX19	-0.21618	0.948228
TMEM182	-0.4599	0.947843
AC007387.1	2.301202	0.947665
GLRA3	-2.89925	0.947249
AKAP5	0.269216	0.947142
WBP1LP2	-2.68165	0.947009
COASY	-0.22407	0.946992
ZBTB44	-0.23481	0.946262
HEATR5A	0.304111	0.944775
GSG1L	-2.34082	0.944431
NPTXR	-0.49349	0.94406
BEST4	3.405272	0.943872
NA	3.375266	0.943802
ALOX5AP	-2.82589	0.943708
SLITRK3	4.107531	0.942481
ZNF860	4.107531	0.942481
RF00019	-4.0901	0.942397
PTP4A1	-0.17173	0.942224
TBX18	-1.94161	0.942144
RASA4	-0.30068	0.942089
AC132192.1	-1.79633	0.942037
RPL34	0.188892	0.941693
PAK2	-0.16015	0.941299
MAFA	-4.08811	0.94111
COL4A1	-0.20011	0.940796
IL5RA	4.341307	0.94058
AC023389.2	4.341307	0.94058
DNAH17-AS1	4.341307	0.94058
OSBPL2	0.219977	0.940489
AL138831.1	1.387937	0.940324
NA	2.08961	0.939914
AC124016.2	-1.30182	0.939846

NA	-2.88195	0.939796
PLCD3	-0.33184	0.939385
NA	0.552367	0.939213
MAML2	-0.41733	0.938371
HIGD1B	2.395668	0.938181
ACOT13	0.288222	0.938144
NA	-1.11962	0.937441
NA	4.121102	0.937281
LINC00930	4.121102	0.937281
NA	-2.86962	0.93701
TSHR	3.080884	0.936762
S100A16	-0.72949	0.936306
DLX4	1.390877	0.936176
SELE	0.648524	0.935776
AP002768.1	-4.08707	0.935732
NA	-3.50455	0.935722
CYB561	-0.24391	0.935716
FAM167A	-0.2077	0.935156
AL353593.2	-1.28777	0.935127
AP005131.1	-2.26665	0.93496
PLA2G4C	-0.71383	0.934937
AC130689.1	-3.50905	0.934419
PPP4R3C	-3.50905	0.934419
DIAPH2-AS1	-2.25924	0.933988
NA	0.617434	0.933637
AL021707.5	0.857683	0.933434
PHLDA2	-0.62884	0.933351
ZDHHC7	-0.21333	0.9332
TOB2	-0.3471	0.933067
GNL3L	0.213873	0.932432
ZNRF2	0.276354	0.932342
RNU6-418P	-2.19435	0.932216
DRGX	-0.47336	0.932084
AC027228.2	-0.25737	0.932008
EHHADH-AS1	-1.62445	0.931925
MTHFD2	0.223625	0.931544
NOMO3	-0.30862	0.930945
TRIM23	0.273657	0.930814
ENOX1	-0.39806	0.930596
BMPR1A	0.192764	0.930492
TRMT10B	0.272754	0.930272
PMVK	-0.20897	0.929971
AC117498.2	1.882518	0.929719
SYNDIG1	-3.95267	0.9297
ZNF274	-0.40202	0.929433
LIMD1	-0.24959	0.929031
IFFO2	-0.82404	0.929027
FGD1	0.262794	0.929016
RBMS1P1	-3.35014	0.92894
ASPRV1	1.476513	0.928844

AC003005.2	2.704823	0.928654
TTC7A	-0.2619	0.9286
FAM66C	0.614	0.928548
LINC02657	-3.47297	0.928442
AC109446.3	-0.7457	0.927846
AC005702.2	-3.40866	0.927824
PDE10A	-0.30011	0.92781
LDLRAD3	-0.20744	0.927311
SPINT2	1.427582	0.926865
KIF27	0.494061	0.926679
CDHR1	-0.78557	0.926092
RN7SL181P	1.760691	0.925933
SOCS3	-0.27531	0.925793
LIPT2	0.668778	0.92578
AC068880.1	-4.086	0.92558
INCA1	0.686728	0.925339
HLF	0.863115	0.925276
PDLIM7	0.233837	0.925154
LRP4	0.351582	0.924981
SORCS3	-1.86915	0.924961
UGDH	0.229853	0.924944
STARD4	0.286514	0.924931
FTO	-0.23769	0.924786
AC009102.2	-0.87138	0.924747
PKP2	-0.36858	0.924447
MRPL34	0.185613	0.923961
NA	-0.96761	0.923706
AC096734.1	2.174302	0.923273
NA	-0.3233	0.923
NA	-0.34766	0.922934
LINC01586	3.040219	0.922328
HAUS2	0.242922	0.922142
CFI	1.21203	0.922061
GNG3	-0.70047	0.921967
IER3	0.428669	0.921427
AC027338.1	1.85603	0.921389
ACTN4	-0.15316	0.921132
PKP4-AS1	-0.58434	0.920918
MYNN	0.21313	0.920703
MMP2	-0.15149	0.920442
NEK6	-0.38676	0.920442
AL513548.1	0.53664	0.920274
NPIPB7	-3.40141	0.920146
AC098818.2	3.953616	0.91989
AC024145.1	-3.46042	0.919725
ACKR4	0.7865	0.919521
AC009407.1	2.957629	0.919249
RIMKLBP2	-1.65813	0.919203
MAGI2-AS3	0.168103	0.918988
ELF2	0.220537	0.91861

AC100861.1	1.452676	0.918181
CYP4F26P	-0.93834	0.91805
RPA4	-3.29601	0.91794
USF3	-0.25168	0.917938
BX005019.1	-1.32395	0.917678
COQ10A	0.241708	0.917638
DHRS4	-0.2998	0.917593
AP001972.2	-0.78157	0.917588
SRPRB	-0.20513	0.917543
RN7SL574P	3.341809	0.917447
RRAGD	0.41645	0.917351
BCHE	0.722049	0.917264
KANK4	0.884468	0.916632
AC090774.1	1.21857	0.916575
HNF4G	-0.5925	0.916425
ISOC1	0.169635	0.915797
SNRNP35	0.251235	0.915549
SNHG6	0.221312	0.9155
GEM	0.639902	0.915398
EFCAB6	-1.28796	0.915371
EXOC3-AS1	0.457331	0.915361
SMDT1	0.303329	0.915255
AP000866.3	-2.84872	0.914739
ZDHHC14	-0.3955	0.914726
AL358334.3	0.809011	0.914459
DENND2D	3.392649	0.913927
TMSB4X	-0.18636	0.913719
RING1	0.195384	0.913215
MTCYBP35	4.063613	0.913152
GATA4	-0.22376	0.913088
MMP11	-0.27907	0.913017
AC019080.1	0.773159	0.912806
PPP6R2	-0.197	0.912227
TMEM108	-0.25592	0.912171
NA	-0.43549	0.911967
AC022613.3	-3.07019	0.911207
COL19A1	1.691089	0.911165
HIST3H2A	0.261171	0.911088
AL161454.1	-4.06198	0.910465
ZNF845	0.424278	0.910263
MLNR	3.099269	0.910167
AL050331.1	0.859999	0.909924
ZNF155	0.34247	0.909793
RFPL1S	-0.41078	0.909229
MEST	0.701956	0.909136
RN7SL674P	-1.16379	0.908651
NANS	0.256302	0.907778
AL080317.2	-1.38614	0.907715
AC004383.1	3.316335	0.907542
HMGB1P47	-2.79309	0.906993

CDC37P1	2.926333	0.905865
MTND2P40	-2.50943	0.905787
LAMB4	-1.54004	0.905679
DANT2	1.700704	0.905623
AL136988.1	-0.22639	0.905382
AC107959.2	1.408521	0.905324
GJD3	-0.78893	0.905267
GAPDHP15	-2.96531	0.904981
AC018521.6	2.002967	0.904264
PLCH1	1.103591	0.902324
LRRC2	0.713035	0.9021
IFNA20P	-4.07861	0.901717
LRRN4CL	3.146154	0.901685
TEAD2	0.220979	0.901677
DEPDC1-AS1	-4.02861	0.901655
AC013726.1	-4.02861	0.901655
AC068987.2	-0.74532	0.901335
NPM1P37	-4.0776	0.901109
EIF3C	-0.28036	0.900983
AL592166.1	0.231799	0.90078
CNTFR	-0.20895	0.900559
ERICH6B	0.560817	0.900208
KIF26B	-0.23243	0.899806
PRR34	0.785597	0.899431
RHOBTB1	0.226331	0.899128
AL359263.1	-4.02445	0.899075
AL355990.2	-4.02445	0.899075
AL139352.1	3.479735	0.898945
AC103739.1	-1.49484	0.898883
FOXD4L1	-1.58696	0.898735
NA	0.379125	0.89867
PAPLN	0.54031	0.898652
ERMP1	-0.31745	0.898533
AC068196.1	1.546493	0.898154
AGL	-0.2132	0.897885
AC012181.1	2.284214	0.897851
KCNK15-AS1	1.226292	0.897217
RND3	0.218812	0.897149
NA	-0.3736	0.896957
FRZB	0.595726	0.896747
SNORA50A	-3.44146	0.896291
EPS8	-0.75004	0.896099
LINC01116	-0.30272	0.895939
S1PR5	-1.18681	0.895693
TCTEX1D4	1.19365	0.895321
AC104534.1	1.914001	0.895089
AC093151.2	-3.50186	0.895041
AC136777.1	-2.60285	0.895023
KLF11	-0.30264	0.894962
GAPDHP48	-3.86705	0.894907

AC073365.1	0.514715	0.894784
SYT5	0.421643	0.894484
GARS	0.159002	0.894251
POP4	0.249883	0.894171
RNA5SP217	2.003152	0.893978
TAOK2	-0.18427	0.893256
REXO5	0.30451	0.893122
GNG4	-0.15315	0.892892
AC009961.3	3.27591	0.892605
MOB2	0.200156	0.892471
OSBPL3	-0.34013	0.892406
MAP1A	-0.22037	0.892389
FO393419.3	2.794348	0.892141
ARHGAP42	4.080238	0.892126
OR6L2P	4.080238	0.892126
MYO1E	0.258526	0.892037
CNTD1	-0.2618	0.891825
AC003991.2	0.288261	0.891696
AL158801.2	2.285603	0.891377
STARD8	-0.6533	0.891372
AC012370.2	-4.01171	0.891163
GRIA2	0.493188	0.890911
IGF2	0.607054	0.890833
AC008808.1	0.692374	0.890832
LHFPL3-AS2	3.426869	0.890711
HMGN1P12	3.426869	0.890711
INPP5D	2.613682	0.890264
FILIP1	0.633084	0.890073
EEF1A1P10	4.076592	0.890038
AL645924.1	4.076592	0.890038
AC099343.2	-3.29843	0.889825
INTS3	-0.18641	0.889505
ALDH1A3	-0.34132	0.889488
AC004707.1	0.214199	0.889463
NA	0.896069	0.889458
SIRT5	-0.32764	0.889303
LRP2BP	0.483644	0.888636
RHOBTB3	-0.20974	0.888622
UVRAG-DT	-0.9118	0.888405
AC073063.1	1.465563	0.88802
AL355102.4	-1.71707	0.887194
AL358176.1	2.239644	0.887192
NA	-1.48056	0.887025
ZNF81	-0.22856	0.887016
DGKQ	-0.39586	0.886646
NCOA7	0.230709	0.885906
RPS3AP38	1.360238	0.885869
LRG1	4.039159	0.885839
AC019155.3	4.039159	0.885839
AL109917.1	4.039159	0.885839

AC008738.5	3.443147	0.885805
FERMT2	0.16597	0.885805
DCAF7	-0.1452	0.885725
DDX59	0.248426	0.885662
ATP5F1E	0.241637	0.884976
ARHGAP19	-0.21891	0.884962
PDE6A	1.446209	0.884688
EMC1	-0.19043	0.884408
AC026271.1	0.426613	0.884327
AC026704.1	-0.93872	0.884245
AC009533.2	1.670672	0.884127
NA	2.92417	0.884084
CRELD1	0.261355	0.883885
EFNA1	0.234797	0.883819
NA	4.064249	0.883352
HIGD2A	0.189652	0.883186
MPND	0.281857	0.882877
ARMC4P1	3.307352	0.882062
KLF2	-1.67563	0.881894
AC124276.2	-2.74218	0.881735
NA	-0.9006	0.88164
AL360270.3	-0.48141	0.881487
RPL5P32	3.851935	0.881395
AC099329.2	-2.91342	0.881029
RF00432	3.890477	0.880918
AC012464.3	3.890477	0.880918
USP14	-0.17275	0.880486
CYCSP34	1.260556	0.88045
EMILIN2	-0.56704	0.880297
HACD1	-0.30384	0.879884
RIPOR2	1.94713	0.879875
YARS2	0.237076	0.879874
AC079140.1	2.898604	0.879771
ISY1	-0.25342	0.879084
AF181450.1	1.821467	0.879083
TEX22	0.762244	0.878933
CTNNBL1	0.209355	0.878868
PAFAH1B3	0.18431	0.878664
DACT1	-0.32327	0.878571
DRAM1	0.245361	0.87834
IGHV3-43	-1.02102	0.877945
G2E3-AS1	3.413205	0.877936
MYO5B	-1.26786	0.877873
RPS5	0.175618	0.877863
AC134349.2	2.612237	0.877562
ZDBF2	0.227936	0.877497
RF00019	4.055904	0.877326
AGBL5-IT1	-1.16194	0.877105
ZNF687-AS1	-0.69774	0.876385
AC090587.2	1.411878	0.876355

SLC35F4	-3.82617	0.876115
SFI1	0.22628	0.876024
RNF112	-0.24916	0.875882
AC087301.1	-1.19932	0.875751
AC000095.1	-2.62015	0.875494
AL606537.1	-2.13356	0.875429
AC011899.2	-2.10205	0.875289
AP001604.1	-3.80715	0.874838
NFRKB	-0.24724	0.874323
S100B	1.001599	0.874282
DERL2	0.176698	0.874043
AC018523.1	-3.98823	0.873414
GDNF	-1.19101	0.873391
AC145207.2	-0.18102	0.873278
BICD2	-0.2107	0.87319
HLA-C	0.241234	0.872716
C11orf74	0.319249	0.872042
ZBTB40	-0.19071	0.872007
TTLL9	-1.0508	0.871698
NA	2.612642	0.871296
PCYOX1	-0.18545	0.870427
AC012254.3	-0.28667	0.870397
NA	-3.60709	0.869499
SNORA75	1.623491	0.869449
DBP	0.318331	0.86943
TBC1D8-AS1	0.952762	0.869374
INHBA-AS1	-3.38949	0.869364
PPP1R1C	3.832269	0.869296
SPATA21	-0.20342	0.869279
AL133215.2	0.735442	0.868631
CAPN6	0.995456	0.868366
DMXL1	0.195742	0.868327
CDC42EP5	-0.74102	0.867728
LSM12P1	2.205883	0.867639
AC015563.2	0.611249	0.867613
LMO7	-0.25581	0.867447
FAM193A	-0.23822	0.867442
AC127024.3	2.20933	0.867439
AC009095.1	-3.42204	0.867253
HNRNPUL2	-0.18099	0.867195
CRLS1	0.213769	0.86663
NA	-0.60243	0.8666
MYT1L-AS1	-0.90768	0.866592
NA	-0.69153	0.866581
CFAP73	-0.91389	0.866113
FLYWCH2	0.287484	0.865747
ST6GALNAC5	0.24458	0.8655
AC100786.2	1.006001	0.865194
AL139011.2	4.003666	0.86469
BIN3	0.231651	0.864668

AC135279.2	3.988549	0.864266
RGN	1.557402	0.864244
ELF3	-1.04665	0.863823
AL160291.1	-1.77096	0.863222
ASAP1	-0.17671	0.863049
AC069287.2	-0.28877	0.862846
SRCAP	-0.20991	0.86282
CCKAR	1.646413	0.862796
ZNF142	-0.27559	0.862614
MAT2B	0.173276	0.862556
CPD	-0.17079	0.8624
ARAP1-AS2	-0.53541	0.86221
AC004023.1	-1.77191	0.862097
DAB1	0.965107	0.860754
FLNC	-0.30178	0.860568
AC090197.1	-0.42824	0.860351
ZNF30-AS1	-3.39427	0.859746
TAGAP	-0.53605	0.859719
GAREM2	0.326143	0.859327
SAMHD1	-0.24779	0.859068
DYNLT3	-0.43474	0.859013
DNAJC5G	-3.97731	0.859006
BCAS1	4.20383	0.858672
AC009487.1	4.20383	0.858672
AC093166.2	4.20383	0.858672
AP000534.2	4.20383	0.858672
HMBOX1-IT1	4.20383	0.858672
AP002770.1	0.307988	0.858588
SLC2A1	-0.19208	0.858308
AL162386.2	-2.19365	0.857542
MTMR11	-0.62503	0.856777
SLC39A4	0.260843	0.85663
AC016877.3	-3.27742	0.856137
PCDHA1	-0.81283	0.856123
SNORA16B	2.130696	0.856026
SNORD63	-1.89853	0.855638
ACSL3	0.180587	0.855198
BEGAIN	0.214272	0.85512
TMEM158	-0.43431	0.855106
SULT2B1	2.736862	0.854838
MAPKAPK5-AS1	0.28242	0.854654
AL118558.2	-3.96975	0.85453
CAMLG	0.215711	0.854511
U47924.2	0.185809	0.854434
RNF145	-0.15474	0.854431
AC007688.1	1.994748	0.85407
AK4P1	1.001368	0.853976
NCK2	-0.17975	0.853512
FBXO25	0.228147	0.853176
LEF1-AS1	-0.37351	0.853149

PHBP4	-3.78784	0.85266
NEK9	-0.14798	0.852357
NA	-0.87044	0.851914
TMEM109	-0.18722	0.85155
GRID2IP	1.144398	0.851433
AC010487.1	-3.27737	0.851221
NA	1.146949	0.851197
PRKCG	-0.68254	0.851031
AC021739.2	0.359033	0.85089
AC107373.2	0.974145	0.850416
DLGAP3	0.381571	0.850262
RAB3B	-0.39177	0.850157
SMAD5-AS1	1.313842	0.850032
AC004408.1	0.847962	0.849893
METTL2A	0.216202	0.849477
PDXP	0.354919	0.849456
AL157932.1	2.39619	0.848756
AC080129.2	-3.79021	0.84817
XRCC4	-0.39514	0.84774
UBA5	0.190484	0.847576
AC007823.1	-1.8434	0.847337
GABARAPL1	0.317137	0.847025
AC009121.1	-1.00496	0.846882
ITPRIPL1	-0.34268	0.846766
RPL7	0.146766	0.846756
EPHB4	-0.37115	0.846729
SARS	0.149475	0.846349
AL138831.2	1.648552	0.846347
NA	-0.80305	0.846228
TOX2	-0.70454	0.84622
TMEM115	-0.20481	0.845978
HLX	-0.28094	0.845827
RHEBL1	0.414317	0.845489
HIPK4	3.959561	0.845357
SYF2	0.189311	0.84533
AC007613.1	0.678196	0.845258
AL590822.2	-0.83122	0.845173
CLDN2	-3.22111	0.844898
BVES-AS1	-0.46315	0.84472
AC007563.2	-0.35776	0.844449
HIST1H2APS5	3.367309	0.844442
CA5BP1	-0.32954	0.844407
NA	-0.28692	0.84378
JAZF1	0.24251	0.843234
CLEC16A	-0.22831	0.843006
AC006504.1	-1.1839	0.842051
TAF8	-0.21201	0.841899
GTF3C5	0.173556	0.841614
AC027130.1	-2.48952	0.841602
ZNF747	-0.302	0.841506

TEX19	1.221443	0.8413
AC008937.2	0.365242	0.84117
AL645924.2	1.930856	0.841145
AC087203.3	-2.45795	0.84081
MAGEC1	-0.38214	0.840398
TOB1	-0.24923	0.840074
ADCY1	-0.17656	0.840035
RPL23P8	1.64318	0.839734
AC079807.1	0.491755	0.83958
HTR1E	0.722237	0.839511
OSER1-DT	0.370662	0.839309
SNRPN	-0.8044	0.839277
BAG5	-0.19193	0.839246
EDNRA	0.257233	0.839085
CDKN1B	0.177844	0.838904
CATSPER3	-2.19578	0.838157
AL355075.3	0.310922	0.838144
AKAP7	2.952665	0.838066
EEF1AKNMT	-0.15515	0.838032
TAS2R8	-3.40747	0.837956
HERC2P4	0.354773	0.837681
AP000438.1	0.833909	0.837648
BTBD9	-0.2557	0.837368
ZNF385C	-0.69064	0.83702
AL138785.1	0.918571	0.836746
DKKL1	1.224835	0.836711
NCOA6	-0.26192	0.836214
AC020612.1	0.957254	0.835637
WIPI1	0.312365	0.835579
EBAG9	0.282415	0.835552
OGFOD2	-0.52754	0.835452
MYL5	0.257587	0.83488
YWHAZP3	1.919013	0.834665
COL27A1	-0.4358	0.834646
AC006130.1	1.494202	0.834634
AC026250.1	3.203599	0.834428
GNAI2	-0.14599	0.834306
AC099489.1	0.764089	0.834299
AC126118.1	1.505651	0.834084
ITGA7	0.634837	0.834044
AZIN1-AS1	0.508835	0.833234
ELN	-0.48263	0.832912
BOC	-0.39406	0.832812
RPS16	0.156003	0.83262
GTF2IP11	2.047338	0.832587
NA	-0.20252	0.832388
NFATC4	0.324297	0.832386
IFITM1	2.868848	0.832294
CCDC38	-1.0059	0.832167
NA	2.155464	0.831995

AC104458.1	-2.72424	0.831913
AC117395.1	1.53319	0.831807
SLC14A2	-3.9597	0.831712
PPP1R8P1	-3.9597	0.831712
AL591623.2	-1.02605	0.831449
AC091849.1	-0.94396	0.831329
TRAF6	-0.26647	0.831207
ST8SIA2	-0.27764	0.831054
NA	1.321069	0.830984
BCL2L1	-0.21411	0.830976
CPOX	-0.20644	0.830961
GP1BA	0.917193	0.830849
AC104115.2	3.344649	0.830809
GSE1	-0.17425	0.830683
AC008770.3	-1.63921	0.830255
FAM86GP	2.000481	0.830092
N4BP2L2	0.168323	0.829988
ZFAS1	0.211678	0.829589
ATP6V1E1	0.17028	0.829404
CHCHD3P3	-2.51279	0.829238
NA	1.537923	0.829038
ZNF555	0.291494	0.828805
MTND3P10	-3.78527	0.828713
NIFK-AS1	0.237152	0.828438
RPL15P3	0.449418	0.828419
SMAD6	-0.17945	0.828207
AL049840.3	-0.55956	0.828049
IMPG2	2.174504	0.828003
AP001469.3	0.480403	0.827906
MRPS36	0.270387	0.827808
FGFBP2	1.631581	0.827741
SMCR2	2.902928	0.827654
ARL4AP4	2.312965	0.82755
AL132838.1	-2.684	0.827162
KIZ-AS1	-2.06672	0.82695
CST6	-2.48185	0.826926
RPL31	0.15404	0.826867
BHLHB9	-0.29342	0.826731
RN7SL775P	-3.95093	0.826723
ZFR2	0.774795	0.826335
HUS1B	2.806906	0.825866
PDIK1L	0.276728	0.825587
ACMSD	1.189607	0.825402
COL7A1	-0.24376	0.825385
C9orf170	3.326853	0.825158
AC136475.1	1.160699	0.825043
PCDHGC5	-0.9186	0.824971
METTL21A	0.244809	0.82486
MIR432	-3.17868	0.824536
MIRLET7I	1.400284	0.824395

SNORA28	3.76735	0.82414
ZBTB26	0.200774	0.824056
ITGB1BP2	1.354783	0.823854
ZMIZ1-AS1	1.238231	0.823749
TSPAN17	-0.18154	0.823724
NA	0.720164	0.823686
SCG2	0.129009	0.823335
SGIP1	0.241968	0.823139
LINC01050	3.791684	0.822634
HOXC5	0.892191	0.822515
SMIM5	0.974274	0.82217
ATP1B1P1	-2.45461	0.822107
FUT11	-0.2294	0.821917
CTBP2	0.169548	0.821805
AC010327.3	2.170376	0.821704
TPT1P6	3.326913	0.821645
KCNJ4	3.914247	0.82073
GAPDHP42	3.914247	0.82073
AC234783.1	3.914247	0.82073
YWHAQP6	3.914247	0.82073
SPINT1-AS1	3.914247	0.82073
SV2B	1.114858	0.820506
LINC00896	-1.46466	0.820475
AL138759.1	3.37642	0.820168
MSTO1	0.254878	0.820159
AC119751.4	-1.35916	0.820096
IGSF9B	-0.33762	0.820081
GATA2	-0.18205	0.819042
SNAI1	-0.26181	0.818828
TCF7	-0.22287	0.818689
KLF5	0.637021	0.818429
AC023154.1	1.162245	0.81795
TIMM50	0.159558	0.817945
SLC19A2	0.234826	0.81784
TIMM44	0.199891	0.817699
SEC14L5	3.23271	0.817605
AC106895.2	0.251741	0.817387
AC025188.1	-0.5384	0.817301
NRTN	0.555187	0.817124
MRPL42	0.178876	0.816888
AC068896.1	-2.33377	0.81684
TWIST1	0.158387	0.816605
AC090409.2	-3.93304	0.816562
AC078880.3	-3.93304	0.816562
LGALS3BP	-0.14986	0.816535
DNAJC5	-0.17901	0.815565
GRPEL2	0.192418	0.81478
NA	-0.41907	0.81466
SUCLG2-AS1	0.824512	0.814632
PGRMC1	0.22553	0.814495

WDR44	0.217703	0.814341
GAS5	0.130823	0.814294
FAM20A	1.808907	0.813564
AC092070.1	3.166081	0.813247
SLIRP	0.217671	0.813154
MIS18A	0.237062	0.813023
EEF1AKMT1	-0.42223	0.812724
CCT6P3	0.453713	0.812437
ARHGEF19	0.343833	0.812437
HTR2B	0.438063	0.812376
NA	2.066533	0.811835
NA	-2.76119	0.81162
DHX37	0.258259	0.811501
RFLNB	-0.26528	0.811499
AC026310.2	-0.82202	0.810971
ADD2	-0.20333	0.810899
BOD1	0.177321	0.810849
AC233976.1	2.521614	0.81082
AC147067.1	3.365996	0.810689
AC137767.1	0.508265	0.810684
AMOTL1	-0.14851	0.810548
PRKAR2A-AS1	0.638302	0.809982
AL139289.2	-3.14572	0.809971
TTC30A	0.330661	0.809885
BICDL1	0.434624	0.80972
RNU6-1278P	3.166157	0.809621
SNORA79B	1.54906	0.809221
AC023355.1	0.265523	0.808906
FAM171B	0.202252	0.808562
MIR4754	-2.70999	0.807791
GIHCG	0.313522	0.807789
AC009509.1	-1.02549	0.807515
RNF149	0.222403	0.807343
IGHV3-33-2	-2.71251	0.807257
AL356235.1	1.39495	0.807199
LINC01565	3.933008	0.806895
AL021937.4	3.933008	0.806895
MCPH1-AS1	0.796859	0.806714
LINC00265	-0.33539	0.8067
AC004890.1	-1.89907	0.806574
LINC01823	-4.09314	0.806488
PIFO	2.069142	0.806286
ECEL1	-3.14568	0.806247
FBXO2	0.575383	0.806232
SHANK1	-0.3566	0.806075
AC079354.1	-3.88551	0.805898
AC069155.1	3.306369	0.805715
WDR45B	0.146569	0.805536
ARL6IP1	0.176339	0.805386
AXDND1	-2.78888	0.805347

PDE9A	-0.29672	0.805304
CCDC110	-1.81085	0.805117
CRHR2	2.179064	0.804845
ANKDD1B	0.925777	0.804502
CCNG2	0.199975	0.804442
AC104619.3	2.233122	0.804317
LYST	0.268443	0.804136
LINC01134	0.849319	0.804084
AC009097.1	-1.41191	0.803934
ASL	0.263859	0.80375
SNIP1	0.186786	0.803251
GPR22	0.721899	0.802885
PSPC1-AS2	0.610029	0.802509
NA	0.671231	0.802411
AL354694.1	2.2998	0.802252
UNC5C	-0.15189	0.802192
TMEM88B	3.707747	0.802134
GUCY1B2	3.729223	0.801789
TAS1R1	3.729223	0.801789
AC104984.5	-3.90533	0.801742
TENT4A	-0.1968	0.801388
AC092687.3	1.363206	0.801367
AC008738.3	2.173616	0.801266
CCDC149	-2.0913	0.801211
AL591848.3	1.134385	0.800993
CKM	-4.08299	0.800943
AL590365.1	-4.08299	0.800943
DEFB109A	-4.08299	0.800943
REPS2	0.892617	0.800883
ZNF397	-0.1907	0.800852
AL645608.2	-0.3722	0.800741
SNRNP27	0.225379	0.800325
CXXC4	-0.29451	0.799954
AC027682.4	-1.88479	0.799582
ZC3H7B	-0.18176	0.799485
LRRC8C	0.243422	0.799332
AKAP4	2.020221	0.7992
TRMT9B	-2.04612	0.799171
RPL12P14	-1.90751	0.799147
NA	1.329774	0.79883
TCF7L1	-0.50118	0.798374
POLA2	-0.25201	0.798355
AC044849.1	0.244717	0.797927
ASMTL-AS1	0.530273	0.797906
NA	2.908743	0.797706
LINC01271	0.407259	0.797693
MACROD1	0.195767	0.797649
FAT4	0.30418	0.797622
NA	4.09147	0.797141
MIR548A3	4.09147	0.797141

RPS2P44	4.09147	0.797141
AC073941.1	4.09147	0.797141
COX10	-0.29086	0.797029
CYGB	-0.19389	0.796406
AATBC	-1.92028	0.795698
ARPC3P1	2.770263	0.795442
F8	0.376142	0.794116
ATG10-IT1	-3.85022	0.793875
EHD3	-0.23037	0.793696
SEMA3E	-2.70735	0.7934
TUSC2	0.205717	0.79337
LINC00565	-1.12442	0.792623
HOXC11	-1.69305	0.792613
AC036111.1	-1.0297	0.792585
EZHIP	1.965875	0.792407
ENTPD3-AS1	0.838117	0.792263
RB1-DT	-3.1427	0.792176
CCL26	3.906079	0.791755
PCDHGB6	1.991987	0.791569
SH3RF2	-1.86544	0.791518
TPTE2	-3.84551	0.791239
SLC25A14P1	-3.84551	0.791239
TLK2P2	-3.84551	0.791239
RPS4XP14	-3.84551	0.791239
NA	-3.84551	0.791239
AC097639.1	-3.84551	0.791239
BCL9L	-0.28973	0.791187
C19orf73	-0.72962	0.791015
CD59	-0.18514	0.789849
BEND4	-0.27061	0.789743
NOS2P3	3.291017	0.789658
ATP5F1AP3	3.291017	0.789658
ITGB7	1.979568	0.789519
VEGFD	-1.587	0.789325
SLC30A3	0.920879	0.7891
NKIRAS1	-0.2427	0.788841
AC074050.3	0.534813	0.788582
AL162431.3	-1.1946	0.788513
PIGCP1	-0.35249	0.787972
ZNF492	-0.41478	0.787525
GLI2	-0.69389	0.787519
AC044860.1	-0.33776	0.787369
DPY19L2P3	0.348344	0.787134
FBLN1	-0.17082	0.78704
PPP2R3A	0.298438	0.786992
GAB2	0.213059	0.78672
CNTLN	-0.23781	0.786589
ZNF407	0.242695	0.786384
SMIM32	1.50681	0.785938
ALPK1	-0.34436	0.78585

CD226	0.838607	0.785328
AL358942.1	-3.14832	0.78528
HSPA1B	-0.25957	0.785272
CFAP57	0.221743	0.785226
FBLL1	0.243012	0.785199
AL772161.1	-1.82187	0.784942
CCDC126	-0.29736	0.784881
AL157871.1	0.240818	0.784605
SRM	-0.21545	0.784318
SUGT1P2	2.889026	0.783992
DNAJC27-AS1	0.994196	0.78359
RGL4	0.283983	0.783516
ANXA2P2	-0.78501	0.783213
ASB13	-0.17263	0.783209
FCF1P1	3.117708	0.783072
HNRNPA1P16	0.719627	0.78307
DIAPH1	-0.15858	0.783029
NA	1.056552	0.78295
KMT2E	-0.15351	0.782884
AC113189.1	0.662424	0.782618
FAM13A-AS1	0.444343	0.782555
AC022893.1	0.913348	0.782296
AC005064.1	-3.25416	0.782247
AL513523.3	-0.85164	0.782239
FAM107B	0.132582	0.7822
TENT5A	-0.33475	0.782117
NRIR	-3.25236	0.78132
PARD6A	0.398369	0.781194
JMJD6	-0.20137	0.78119
PPDPF	0.208358	0.78108
AC090152.1	0.437797	0.780234
AP003392.5	-2.2615	0.780208
UPK3B	2.726322	0.780199
CNPY2	-0.34676	0.780068
SNHG5	0.159781	0.779525
CD99	-0.87669	0.779273
RAB25	1.674719	0.779248
SNORD101	-1.75378	0.778876
NA	-0.5364	0.778823
RASA3	-0.20361	0.778596
CCDC28B	0.246084	0.778252
SLC22A15	0.422376	0.778187
AC046143.2	2.735815	0.778179
RAB11B-AS1	0.282397	0.778121
AC067817.2	-2.48902	0.777723
AC093724.1	-0.59561	0.777529
RNA5SP265	-3.22097	0.777428
IGFALS	-1.66931	0.777421
DNM1P51	1.280077	0.777326
PRKX	0.210289	0.777182

RNU6-638P	-3.25425	0.777173
AOC2	-0.74671	0.777017
AL513320.1	-1.56475	0.776831
PAIP1P1	-2.69361	0.77676
AC011825.2	2.295746	0.776644
ZBED4	-0.16243	0.776419
AL359504.2	0.467613	0.776398
ANPEP	-0.38331	0.776129
HOXC8	-0.85252	0.775984
AC091906.1	-3.27579	0.775847
AL589935.1	-0.95592	0.775747
AL136988.2	-2.4441	0.77526
PLPBP	0.202258	0.775215
AC008763.1	4.051854	0.774942
MINDY2	-0.28083	0.774517
HHIP-AS1	-2.58155	0.774431
MAP3K3	-0.1561	0.773721
NUDT10	0.406883	0.773582
TNRC6B	-0.20302	0.773559
FAM126B	0.232236	0.773478
MANEA	-0.25703	0.773123
RELB	0.269754	0.773107
MIR4515	-3.20977	0.772809
RNA5SP445	3.449584	0.772647
AL160191.2	3.449584	0.772647
AL031666.2	3.867715	0.772555
RPS3AP47	1.966641	0.772523
HIPK3	-0.18807	0.772198
AL357055.2	3.842836	0.772125
RNU7-75P	3.842836	0.772125
ERRFI1	0.257557	0.771865
FHOD1	-0.18645	0.771862
CYP2U1	-0.24243	0.771454
AL139021.2	1.83685	0.771128
AC005104.2	-3.20514	0.770645
AL357556.1	3.839125	0.770629
NA	3.839125	0.770629
RN7SL417P	-2.38354	0.77043
AP000942.2	-0.69475	0.770409
NA	0.361908	0.7704
CD44-AS1	-0.55867	0.770383
NA	1.100206	0.770338
AC246787.1	1.148867	0.769932
CPNE8	1.190291	0.769311
AC073283.1	-3.22106	0.769158
PRPH2	3.862616	0.768666
SF3A1	-0.19974	0.768368
ENTPD3	2.445873	0.768355
AL034380.1	-0.41692	0.768314
AC104002.3	3.139606	0.768046

NICN1	0.234321	0.767538
ARHGEF28	-0.29	0.767178
KCNK5	-0.8976	0.76712
ADPRHL2	0.205064	0.767104
CNTN4-AS2	3.822768	0.767068
AC013356.3	3.822768	0.767068
AC027243.2	3.822768	0.767068
PBX4	-0.83996	0.766902
AP000785.2	4.035862	0.766036
AC245008.1	4.035862	0.766036
BCL2L15	1.340356	0.765988
AC136604.2	0.414511	0.765715
FXYD6	-0.22169	0.765605
AC008667.3	-2.15966	0.765604
AC089984.1	1.084401	0.765059
AL445189.2	-3.81175	0.765005
LINC00595	2.623278	0.764732
MIXL1	-3.20984	0.764495
DAP	-0.18377	0.764435
ZNF576	-0.28462	0.764197
AC025917.1	0.46545	0.764024
RPL10	0.152646	0.763952
WDR24	-0.26467	0.763606
CCDC160	2.82858	0.763537
PRMT7	0.210812	0.763436
AP005432.2	3.654905	0.763271
PCSK7	-0.24591	0.763258
DISP3	-0.46089	0.762875
C6orf89	-0.14424	0.762722
COMMD4	0.165325	0.762608
HIST1H2AI	1.15617	0.762369
ERN1	0.288512	0.762328
MAN2C1	0.163761	0.762184
PRR12	-0.24845	0.762134
GYS2	3.847816	0.762017
TRIM36-IT1	3.847816	0.762017
NA	3.847816	0.762017
FGFR1OP	0.236016	0.761789
OPRD1	0.259008	0.761783
ARSG	0.468827	0.761218
AC234781.2	-3.80448	0.761081
BRCC3P1	-2.29922	0.761
NA	-3.18916	0.760998
SNORA80D	-3.18916	0.760998
TCAF2	-1.33235	0.760799
SERPINE3	0.702505	0.760395
NR1H2	0.199407	0.760178
AL022322.1	-1.38774	0.760058
LIMA1	0.253666	0.759883
ZNF720	0.263163	0.759789

ACVR2B-AS1	0.469494	0.759751
AC105020.1	-0.57741	0.759611
MORF4L1P4	3.810322	0.75961
UACA	-0.27797	0.759504
CCDC144NL	0.58313	0.759273
CD36	-1.37944	0.759066
ATP6V1B1	0.493642	0.75894
NDUFA9P1	-3.04786	0.758787
AL390195.2	-1.14183	0.758642
AL662844.4	1.171399	0.758488
AL022329.2	2.283089	0.758475
TSC22D3	0.224571	0.758435
MTOR	-0.15521	0.758416
PDCL3	0.193122	0.758293
CERCAM	-0.2607	0.758271
OSR2	2.914704	0.758168
TAS1R3	1.171583	0.758056
ATP2C2-AS1	1.518286	0.757548
PPIAP73	3.802388	0.757548
TPSB2	3.802388	0.757548
AP000533.2	3.802388	0.757548
RPL6P2	3.802388	0.757548
AC074134.1	3.802388	0.757548
AC005072.1	3.802388	0.757548
SFTPD-AS1	3.802388	0.757548
AC013400.1	-2.52165	0.757462
MKRN7P	1.843881	0.757142
MIR3661	0.227611	0.757048
MYH16	-2.18254	0.756816
EVI5	-0.25958	0.756794
TBC1D16	-0.15303	0.756549
RNPEPL1	-0.26789	0.75638
AC007163.1	0.303176	0.755907
TONSL	-0.23847	0.755779
DISC1	-0.32111	0.755511
C22orf24	1.564898	0.755481
RNU6-1016P	-1.94128	0.755081
SAP30	0.196373	0.754837
AL365226.1	3.206093	0.754777
NDUFA4L2	0.741372	0.754586
VLDLR	-0.19215	0.754531
MIR3918	1.836006	0.754521
ZNF772	0.326453	0.754065
SS18L2	0.234064	0.754037
TP73	-0.31493	0.753865
LINC01003	0.304485	0.753839
NA	3.257229	0.753803
ZNF350	0.361555	0.753702
AC027544.2	3.257237	0.753606
AC064805.2	3.257237	0.753606

CDK5	0.15375	0.753425
CES3	-0.90139	0.753204
CA5B	0.327676	0.75242
AC006064.1	-1.64422	0.752373
KRT18P31	-3.62333	0.752366
LCP1	-0.31996	0.752187
ERI3	-0.1686	0.752107
RAD51D	0.340843	0.752014
MIR23B	1.593665	0.751947
DTX1	0.315122	0.751789
COMTD1	0.450249	0.751768
SIAH2	0.235511	0.751551
AC036103.1	-0.27368	0.751528
TMEM108-AS1	0.587832	0.751512
CAMKK2	0.198768	0.751497
RF00019	-1.98822	0.751268
AK4P3	0.917298	0.751261
AC141557.1	-0.98456	0.75124
COMMD6	0.214096	0.750843
TDGP1	3.211734	0.750707
AL158166.2	1.286537	0.750645
RBFA	-0.31761	0.750514
ACTA1	-1.96991	0.750474
AC007848.1	-0.75409	0.750403
SLC17A7	-2.15523	0.750393
AC113346.1	-1.7931	0.75018
PON1	-3.61928	0.750168
C15orf41	-0.29911	0.750144
C3	0.305376	0.750142
NA	-0.74622	0.750042
AC009506.1	0.412553	0.749821
AC113191.1	-0.46008	0.74965
AL162171.1	-0.18972	0.749391
DGKB	-0.48226	0.749193
IGLV1-51	3.047366	0.749181
PIM2	0.303316	0.749004
NA	3.798363	0.74887
AP001528.1	3.798363	0.74887
TNRC18	-0.22785	0.748744
SMIM4	-0.26766	0.748721
NMNAT2	-0.16381	0.748422
RF00019	-3.04488	0.748338
AC127496.4	0.790765	0.748073
SYNM	-0.21462	0.747891
LINC00987	0.989708	0.74789
AC009237.3	0.604766	0.747799
AC020558.2	0.676331	0.747089
ZNF826P	-1.10201	0.747036
SMAD2	0.146728	0.746811
ZNF513	0.15768	0.746782

LINC01091	-2.08846	0.74645
VSTM2L	0.390124	0.746408
CLHC1	0.31095	0.746352
SLC25A20	-0.21832	0.746289
TRAPPC12-AS1	-0.46481	0.746249
NCAM1	-0.21686	0.746129
WARS2	0.325444	0.746107
HDGFL2	0.175842	0.746097
PPIC	0.289959	0.745874
SULT1C4	0.222741	0.745786
NA	3.047302	0.745438
KAZALD1	-0.41681	0.745355
FO393401.1	-0.88438	0.745256
RPL21P3	1.519265	0.745105
MARS2	-0.27523	0.745055
CDC26	0.305084	0.745017
TTC9B	0.492322	0.745015
MEF2A	0.161653	0.744835
KRTAP5-9	2.712941	0.744796
RF00072	3.781663	0.744731
HMGN2P4	1.906505	0.744325
LHCGR	-3.0119	0.744109
TMEM14DP	3.781081	0.743867
MECOM	-1.92104	0.743849
AL022069.1	0.909537	0.743822
AL589765.1	1.379603	0.743782
SNX22	0.141896	0.743673
AC087623.2	0.968998	0.743491
CEP250	-0.20968	0.743372
AC018761.3	3.180212	0.743175
MTND4LP30	3.183723	0.743173
PJA2	0.147726	0.743145
MRPL46	0.299123	0.742999
FKBP10	-0.14656	0.742955
HSD17B7P2	0.555026	0.742815
AL355512.1	2.267607	0.742404
ATP6V1E2	-0.26185	0.74227
SNORD46	1.804776	0.742161
STX5	0.202157	0.74215
ZNF382	-0.32623	0.741938
AC010761.1	-0.36282	0.741869
RRP1	0.172002	0.741863
MADCAM1	0.665439	0.741819
VWA5B2	-0.58238	0.741802
CLN8	-0.21393	0.741794
IFNAR1	0.177752	0.74164
AGGF1P1	-1.30847	0.7414
ENDOD1	-0.29371	0.741252
PDPR	-0.16087	0.741186
MIR100HG	0.175271	0.741081

GCNT4	1.698148	0.74097
RNU6-237P	-3.79204	0.740763
NA	-0.96009	0.740638
CALR	0.139145	0.740509
AC131953.1	-0.76123	0.74032
CPM	-0.3499	0.740273
AC074135.1	-0.81941	0.740163
MIR647	-1.29993	0.740094
ZEB2	0.143792	0.739962
TATDN1P1	2.620954	0.739836
SLURP1	-3.76474	0.739748
AC093702.1	-3.76474	0.739748
AC063979.2	-3.76474	0.739748
VDAC2P2	-3.76474	0.739748
PEX11G	0.693364	0.739728
AL049820.1	-3.60004	0.739726
NA	-3.60004	0.739726
TMEM169	-0.28669	0.7396
UBE2E1-AS1	0.999702	0.739434
SDHB	0.185977	0.739341
NA	2.423041	0.739209
SYT14	0.342695	0.739137
AC040168.1	-0.66799	0.739078
ROBO3	-0.27063	0.739053
SEC63P1	-2.63356	0.739034
GRK5	0.335077	0.738901
AC106881.1	0.26344	0.738749
RPL26P19	0.724202	0.738522
SNRPEP2	3.227174	0.738263
APOBEC4	-3.5973	0.738255
AC244035.1	-3.5973	0.738255
ZNF347	-0.32617	0.738207
IGHV1-45	1.74798	0.737971
NA	-0.46325	0.737927
PLEKHO1	0.168398	0.737775
NOS1AP	1.674186	0.737748
AC016065.1	0.412573	0.737747
NOP14	-0.16065	0.737653
ACTR6	0.166769	0.737565
AL138781.1	1.021242	0.737536
DPP4	-0.70753	0.73751
TMEM130	0.621512	0.737275
SYNGR4	-1.02417	0.737266
HID1	0.397392	0.737107
AL591848.4	0.61007	0.736834
TMEM251	0.320775	0.736679
ZNF511	0.179283	0.736544
RPS24P8	1.353384	0.736525
UBAP1L	-0.34194	0.736451
PPP1R42	-3.78343	0.736289

GMPSP1	-3.78343	0.736289
CHRNA3	-0.13347	0.735978
UBE2D4	-0.22649	0.735917
NA	2.80237	0.73587
CLMN	-0.55531	0.735844
AL031716.1	2.773249	0.735651
RBM4	-0.35853	0.735411
AC011503.1	0.648622	0.735297
INSYN1	0.567455	0.735281
BIRC3	0.288688	0.734985
ARHGAP32	-0.26514	0.734811
AC008013.3	-1.86806	0.734797
BOLA3-AS1	0.308073	0.734505
NA	3.030009	0.734419
EXOSC8	0.193867	0.734282
FBXL19-AS1	0.28055	0.73403
NOL11	0.15922	0.733932
CCDC146	0.493429	0.73369
EGFR	-0.20999	0.733546
IRAK3	-0.74409	0.73341
SYCE2	-0.49352	0.733283
SLC9A3R1	-0.19359	0.733238
AC137630.2	-0.30354	0.733209
ZNF649	-0.31261	0.732918
TMEM51	0.205213	0.732806
TMEM38A	-0.40133	0.732563
MTHFD1L	0.181954	0.732467
PGAM4	2.513651	0.732466
PSTPIP1	-0.57113	0.732451
RPN1	0.145352	0.732131
EEPD1	-0.27374	0.731994
RF00019	3.16102	0.731967
RF00019	3.16102	0.731967
STK24	-0.171	0.731772
GK	-0.38681	0.731626
AC018647.2	-0.21196	0.731197
KMT2A	-0.17178	0.731153
EPB41L4B	-0.68634	0.730765
CPSF7	-0.15569	0.730743
HRH1	-0.30199	0.73072
GCSH	0.304742	0.730685
CDH17	2.385034	0.73062
LAYN	-0.24595	0.730619
TMSB15A	0.216166	0.730583
AL162412.1	2.40013	0.730448
GSN	-0.20363	0.730162
RAB40B	-0.25381	0.730092
GABRP	-1.74007	0.730064
PAN3-AS1	-0.75138	0.729866
AC090204.1	0.811905	0.729603

AC092017.1	-2.27596	0.729568
NA	0.895249	0.729563
FAM87B	2.686263	0.729545
POLR2H	0.155693	0.72942
UBE2SP1	0.952427	0.729327
FLNB	0.242139	0.72905
GCNT7	-1.04251	0.728958
AP002840.2	-1.61019	0.728917
RNU7-20P	-1.56778	0.728575
ERVK3-1	-0.19399	0.728513
AF131215.2	1.490811	0.728117
HLA-T	3.76053	0.728109
MIR3117	3.76053	0.728109
ODC1	0.147953	0.728041
YDJC	0.246977	0.728016
MAGEA4	-3.60134	0.727893
AL355304.1	-3.60134	0.727893
DUX4L50	-0.24658	0.727756
ELP6	0.17237	0.727705
LINC00892	-2.67278	0.727553
OSBPL10	-1.22472	0.72753
LCOR	0.214031	0.727458
AL049779.2	3.054668	0.727404
AC090971.1	-0.30817	0.727276
NA	-1.16306	0.727274
ITFG1	-0.17086	0.727254
BTF3P10	-3.15661	0.727194
RPS26	0.172634	0.727166
FAR2P4	-3.9412	0.727006
SLCO2B1	-0.39523	0.726987
REXO2	-0.27551	0.726913
AC108673.3	1.721574	0.726808
ENDOG	0.190537	0.726741
AC018410.1	1.772701	0.726656
AC110285.2	-0.87034	0.726629
TJP2	0.517343	0.72653
RPS2	0.130886	0.726514
AC006065.4	3.747569	0.726482
AC090825.2	3.747569	0.726482
TMEM82	3.029882	0.726471
NA	-1.12086	0.72627
AC005544.1	1.251257	0.726027
TPRG1-AS1	-3.76335	0.725881
FAM234A	-0.19349	0.725677
METAP2	0.157897	0.725649
TMEM223	0.195542	0.725622
TMEM229A	-0.26843	0.725588
KLK14	3.141596	0.725555
BRD7	-0.14314	0.725521
ETV6	-0.21852	0.725118

AC137932.2	-3.12934	0.725115
BTF3P9	-3.59583	0.72502
AC103740.1	-0.3452	0.724862
THORLNC	-2.21804	0.724696
ELOA	-0.14457	0.724666
CDHR3	0.46201	0.724369
WARS	0.158666	0.724222
AIFM1	-0.17878	0.72405
PIK3C3	0.149113	0.723729
FAM120B	-0.15876	0.723722
GALNT2	-0.1517	0.723607
MIATNB	0.443585	0.723603
CHD8	-0.13937	0.723343
NA	1.533053	0.723338
NA	1.282816	0.723162
CARNMT1	-0.34217	0.723036
CPB1	3.609165	0.722963
AC080013.6	-1.61906	0.722888
TICAM1	-0.25166	0.722789
XG	-1.5687	0.722562
ATP5ME	0.262045	0.722407
IQCH-AS1	0.333675	0.722213
ARL5C	3.141649	0.722045
FXYD3	-3.93106	0.721907
RN7SKP269	-3.93106	0.721907
AL138962.1	-3.93106	0.721907
GFOD1	0.317261	0.721853
RNU6-584P	3.160908	0.721818
AC008743.1	1.419412	0.721635
AC008443.4	-3.12929	0.721542
NA	-0.38518	0.721296
RPL10AP6	0.746864	0.721195
PARD3B	0.484691	0.721157
TMEM176B	1.250892	0.720997
RNU6-105P	-2.99608	0.720965
NA	-2.50555	0.720904
ZNF609	-0.19032	0.7209
COL9A1	0.704224	0.720794
CRPPA	1.200416	0.720707
RSPH1	-0.93719	0.720705
AL135791.1	1.222678	0.720661
HUWE1	-0.16978	0.720659
ICAM3	-0.3269	0.720621
DRC1	-3.12734	0.720553
PSMB7	0.145989	0.720314
CTAG2	-3.92628	0.720253
TEX29	-3.92628	0.720253
C10orf53	-3.92628	0.720253
SETP8	-3.92628	0.720253
AL662890.1	-3.92628	0.720253

AC135977.1	-3.92628	0.720253
AL133368.1	-3.92628	0.720253
AL512310.8	-3.92628	0.720253
RPL13P5	-0.81093	0.720228
AP4S1	-0.38755	0.720202
SEMA3C	0.126846	0.720199
AC013731.1	1.111603	0.719933
ZNF157	-0.68975	0.719879
RPL23	0.154953	0.719825
LINC00334	3.768975	0.719814
RPS27P25	3.768975	0.719814
AC118465.1	3.768975	0.719814
ALDH1B1	-0.21176	0.719711
NA	-0.55646	0.71961
HMOX2	0.203134	0.719461
PHOSPHO1	-1.76382	0.71941
AC083873.1	-3.1312	0.719385
AC069503.1	1.791473	0.719136
TFG	0.138291	0.71912
C1orf122	0.176479	0.718933
SLC25A5-AS1	0.180412	0.718729
NFIX	-0.25175	0.718569
CDKN2B	2.255246	0.718555
KCNK3	0.22426	0.718339
KIAA1211L	-0.48179	0.718304
UBA1	-0.14204	0.71825
AC106038.1	-1.29453	0.718185
ZNF277	0.208377	0.717809
SNCA-AS1	1.552854	0.717748
NA	1.615787	0.717227
AC009950.1	-1.04089	0.717094
C19orf81	-0.48969	0.716866
LINC00653	0.708632	0.71685
AL035448.1	0.340022	0.716643
MED20	0.210192	0.716536
PCBP1	-0.12716	0.7165
TP53INP1	-0.15873	0.716457
SSBP3-AS1	1.097827	0.71626
HIST2H3DP1	1.153445	0.716189
AF038458.3	-3.1263	0.716141
LRRC6	0.55604	0.716112
PMFBP1	-0.22778	0.716065
AF001548.2	1.170871	0.715981
SLC25A29	0.185955	0.715965
ATP6V1A	-0.17274	0.715826
TMEM117	0.280742	0.715686
PTPRU	-0.15842	0.715575
AC012640.2	0.69324	0.715356
PLA2G12AP1	-2.00426	0.715319
SCN2A	-0.17709	0.715221

AC116616.1	1.55009	0.715218
DLGAP4	-0.19587	0.715146
AC018816.1	-0.35229	0.715066
LINC01341	-0.57089	0.715056
AFDN-DT	-1.15285	0.714976
REV3L	-0.17085	0.714769
MUTYH	0.206077	0.714605
ATG10	0.170129	0.714563
TMEM151A	-0.46815	0.7145
RPL32	0.15175	0.714453
RNU6-323P	3.131382	0.714369
AC131097.2	1.876304	0.714277
MAPRE1	0.147292	0.714241
RNF38	0.184533	0.714237
TUBB4A	-0.47061	0.71423
AC010442.1	-0.23027	0.714153
MAGEE2	-2.49588	0.714098
PDK1	-0.31152	0.713977
CD37	0.273111	0.713881
N4BP3	0.700268	0.713805
ASB7	-0.218	0.713687
AC021087.1	2.003368	0.713194
KIFC1	-0.17277	0.71309
EBPL	0.174139	0.712802
MIR1282	0.147634	0.712589
AL590762.4	2.612459	0.712529
AC006504.3	1.432085	0.712409
SKAP2	-0.59156	0.712266
ADRM1	0.128462	0.712172
SCRIB	-0.16883	0.712116
SMYD4	-0.20177	0.712042
NFASC	-0.33385	0.711896
AC145350.2	2.000291	0.711797
TMC5	1.513379	0.711796
ITSN1	-0.17008	0.711404
AC244517.1	-0.47407	0.711226
MYOZ2	0.910496	0.711168
IDH3B	0.173437	0.711119
RPL23AP88	-3.1311	0.710998
TMEM213	-0.44038	0.710906
STX3	0.248324	0.710807
AC016027.1	-0.27629	0.710381
LINC02241	1.366957	0.710177
BORCS8	-0.34072	0.710009
AC008429.1	0.852259	0.709867
WTIP	-0.38825	0.70981
AC011352.1	3.138087	0.709793
UXS1	0.184893	0.709715
PI3	-2.09934	0.709546
CYCSP40	3.581313	0.709486

ZC3H12B	-0.31655	0.709304
PCSK4	-0.49886	0.709287
ATP5MD	0.183364	0.709236
PCGF1	0.191667	0.709191
APP	-0.11211	0.708961
TCHH	-2.15279	0.708804
METTL25	0.472723	0.708702
FAM160A1	-1.26746	0.70857
AL023583.1	1.997773	0.708446
RPRD1B	-0.1833	0.708434
TYRO3P	1.797334	0.708238
AC006299.1	-2.6203	0.708171
TMEM231	-0.33471	0.708127
TAS2R19	1.424546	0.708118
SOHLH1	-1.61864	0.708059
SON	-0.11993	0.707951
TTC7B	-0.24765	0.707928
TMEM258	0.145284	0.707897
NA	-3.13994	0.707867
AC062037.2	1.395275	0.707735
FTLP12	-1.51985	0.707714
AFAP1L2	-0.76584	0.707693
AC090607.2	-0.78931	0.70752
NOP53	0.157093	0.707382
MRPS26	0.158426	0.707233
TAPBPL	0.378937	0.707163
FAM72B	-0.33726	0.707119
AP001830.1	-1.79907	0.707038
PROX1	-0.17916	0.706962
RPL5P30	2.37617	0.706951
MAX	0.155166	0.706811
SH3BP5	0.2399	0.706712
CRY1	0.200243	0.706361
MIR616	1.47321	0.706165
PLS1	0.268095	0.706146
GLYATL1	1.986754	0.706012
AC113382.2	-2.14248	0.705906
RECQL5	-0.19218	0.705814
CCDC22	-0.24236	0.705784
YWHAG	-0.11479	0.705758
RPL6P24	1.084551	0.705554
PLK2	-0.24944	0.705523
AC006213.1	-0.38979	0.705304
MIR635	1.329037	0.705282
AC026403.1	0.643056	0.705276
UGDH-AS1	0.500572	0.70518
TNFRSF1A	0.252026	0.704816
ASF1B	-0.25184	0.704765
BX322639.1	-0.25401	0.704186
FAM215B	1.292746	0.704168

AUNIP	-0.3628	0.704076
RF00017	0.768773	0.703763
VAMP7	0.157493	0.703722
NOMO1	-0.17302	0.703557
SPTSSB	0.396801	0.703506
SETX	0.140355	0.703485
LRRTM2	0.389551	0.703444
UBALD2	0.17689	0.70304
HS6ST1	-0.20666	0.702958
TSSC4	0.181786	0.702901
EFL1	0.178572	0.702824
VDR	1.176022	0.702759
GTF3C1	-0.1567	0.702709
PPP1R12B	0.179698	0.702658
NKAPP1	0.876697	0.702408
RPS20P33	-1.04118	0.702122
AL158847.1	0.38886	0.702048
NA	-0.57418	0.701739
GOLGA5	0.176628	0.701516
FAM102B	-0.23407	0.701402
F7	-3.68974	0.701391
NA	-3.68974	0.701391
ZSCAN5DP	-3.68974	0.701391
FAM162B	1.433758	0.701371
URB2	-0.15412	0.70121
AC027682.3	-0.69325	0.701178
LIG4	-0.22767	0.700987
NA	-0.74282	0.700905
ZNF71	-0.24142	0.700739
DPY19L2P4	0.561209	0.700493
ZNF792	-0.26883	0.700456
AC024451.4	-3.07929	0.70043
NXPH3	1.821774	0.700421
LDHAL6FP	-3.71101	0.70037
ELOCP21	-3.71101	0.70037
CACNA2D3-AS1	-3.71101	0.70037
AC005699.1	-3.71101	0.70037
AP001267.4	-3.71101	0.70037
AL590133.1	-1.66672	0.700304
ERFE	0.662217	0.700277
RF00156	2.943518	0.700245
AC087393.2	-0.18583	0.69992
RNU7-123P	-1.95369	0.699891
SIX3	0.168901	0.699811
EMCN	-3.70971	0.699728
EEF1GP7	-3.70971	0.699728
AL513302.1	-3.70971	0.699728
AC136624.3	-3.70971	0.699728
RNF146	0.18086	0.699661
PALLD	0.249444	0.699634

CACUL1	-0.15329	0.699585
KCNMA1	-0.27919	0.698903
THG1L	0.188201	0.698865
SMG1P7	-0.29346	0.698793
NA	-1.05529	0.69872
CBLN2	0.276575	0.698522
NA	2.221591	0.69844
IFT27	-0.18328	0.698404
SSH2	-0.1601	0.698381
MTND5P11	0.809052	0.698349
IRX6	-0.61115	0.698266
ANP32BP1	2.180674	0.698168
SLC22A8	2.718333	0.698156
HJURP	0.180323	0.698065
CCDC117	-0.18754	0.69804
GOLGA8A	-0.1528	0.697979
SIX4	0.754878	0.697844
CLSTN2	0.470294	0.697594
PIM3	0.243295	0.697482
RNA5SP216	1.200636	0.697465
BCOR	-0.16144	0.697423
AC008764.6	-0.45803	0.69734
CADM1	-0.20215	0.697301
GFI1	-3.09675	0.697201
AC009228.1	-0.71357	0.696967
SPATA45	-2.7272	0.696658
HOXD1	-1.28231	0.696577
AC007038.2	0.661469	0.696503
NA	3.720863	0.696307
H1FX	0.152031	0.69623
AC008608.2	-0.86706	0.696221
AP000593.1	-1.16118	0.696201
AC110597.1	1.072516	0.6962
AC092910.3	-0.42417	0.695998
IL32	0.240987	0.695965
SEMA5A	0.220838	0.695942
AL513523.1	-3.11508	0.695879
COL4A3BP	-0.18861	0.69579
SH2B2	0.257196	0.695695
UBE3C	-0.13738	0.695519
LNP1	-0.37439	0.695261
KCNA2	-1.35916	0.69518
DLGAP1-AS1	0.395593	0.695105
PRSS53	0.549025	0.695092
PHYHIP	-0.94534	0.695056
WASH2P	0.265222	0.694923
EFHD1	-1.2502	0.694882
TMEM31	-3.66832	0.694788
AC241585.1	-3.66832	0.694788
AL136528.2	-3.66832	0.694788

AC128657.1	-3.66832	0.694788
LRRC15	-0.50787	0.694606
WDR38	1.704348	0.69423
AL133338.1	-0.69219	0.694167
COQ8B	0.209847	0.693991
PIBF1	0.233844	0.693818
INPP4B	-0.67816	0.693686
RNU6-377P	-3.08245	0.693524
AC016866.1	2.696102	0.693436
ZIM2	1.635314	0.69334
NA	-2.32901	0.692988
AC106052.1	2.93622	0.692929
LCNL1	1.556642	0.692872
RPL4	0.126266	0.692793
LIVAR	-3.66432	0.692775
AC007663.4	-0.88824	0.692674
AC011511.2	0.235592	0.692633
BTN3A3	-0.2569	0.692558
RBM12	-0.16599	0.692555
AC124283.4	1.868225	0.692523
GLT8D2	-0.39863	0.692503
LARP1	-0.14714	0.692471
FHL2	0.274269	0.692174
BNC1	-3.12401	0.69212
TCEA2	0.21927	0.69196
C7orf31	-0.48225	0.691826
COPS9	0.172786	0.691495
LRRC4	-0.43937	0.691441
RPLP0	0.171881	0.691412
AFG3L2	-0.1471	0.6912
AC036108.2	-0.25809	0.691003
FSTL1	-0.18716	0.690466
MCFD2	0.128451	0.690349
NA	0.415622	0.690115
SMPD4	0.160487	0.689829
ISM2	3.884083	0.689753
C1orf105	3.884083	0.689753
PFN1P6	-0.47511	0.689548
MGST3	0.158593	0.689499
NACA	0.143151	0.689472
AL139327.1	3.881957	0.689394
OTP	3.881957	0.689394
RNU1-1	3.881957	0.689394
VTI1BP2	3.881957	0.689394
ACTN4P1	3.881957	0.689394
AL160162.1	3.881957	0.689394
RPL23AP43	3.881957	0.689394
AL022313.2	3.881957	0.689394
RPL34P1	3.881957	0.689394
RN7SL743P	3.881957	0.689394

CLSTN2-AS1	3.881957	0.689394
AC007848.2	3.881957	0.689394
MN1	-1.04144	0.689227
FRG1-DT	-1.26491	0.689196
NA	-0.3088	0.689094
SLC28A3	-3.26947	0.68907
GCHFR	0.431053	0.688951
RHOXF1-AS1	-2.409	0.688873
RF00019	2.954709	0.688757
AC009054.2	-1.85557	0.688705
TNC	-0.19594	0.688669
CCDC12	0.204569	0.688519
AP003392.3	0.198521	0.688349
AC024075.1	0.390572	0.688324
AC073912.2	-3.11426	0.687851
EDF1	0.13663	0.687826
LRRC23	-0.34423	0.687804
AC044802.1	1.020434	0.687578
SCLY	-0.35856	0.687485
GPSM3	-0.65306	0.687286
RPS4X	0.125787	0.687238
LINC02443	-2.73239	0.687208
AC244197.3	-0.91892	0.687139
TTC23	-0.38775	0.687085
ATF7	-0.18579	0.686706
MIB1	-0.14511	0.686691
ZMAT3	-0.17097	0.686626
AC145207.5	0.373112	0.686611
SLC25A25-AS1	-0.28377	0.686556
ZNF385B	-3.06192	0.686441
FAM162A	0.245024	0.686307
BTN3A2	0.191903	0.686255
CCDC69	1.47937	0.686051
TSNAX	-0.18065	0.685827
AL732372.2	-1.17976	0.685685
TMEM170A	0.216915	0.685557
AC005261.2	0.186409	0.685527
NA	-0.23848	0.685488
SLC6A10P	-1.89072	0.685339
NA	0.942037	0.685168
ZC2HC1C	0.588585	0.684968
MED18	-0.22968	0.684961
NQO2	-0.33804	0.68494
LSM2	-0.19368	0.684856
MIR193BHG	-2.1764	0.684787
NA	1.768173	0.684755
INSYN2A	-2.71659	0.684724
AC241584.1	3.513164	0.684714
AL031123.2	1.007338	0.68459
AL354920.1	0.283217	0.68407

EFEMP2	-0.19082	0.683916
RPL11P4	-3.64666	0.683892
AL353764.1	-3.64666	0.683892
NA	-0.15493	0.683808
CDCA8	-0.18367	0.683774
MOBP	1.446341	0.683713
PAQR6	0.256421	0.683499
PIGA	0.26188	0.683456
TK2	0.276535	0.683452
AL049840.1	0.344642	0.683314
AC096564.2	1.822673	0.683278
AC012186.2	2.925047	0.683212
RPS15	0.142431	0.68302
CTNS	-0.23161	0.682919
MIR4653	-1.47256	0.682829
C11orf24	-0.1748	0.682808
DCTN2	0.152987	0.682798
ASB1	-0.14892	0.682743
NRF1	0.234781	0.682689
UTP4	0.130085	0.682576
FRAT2	0.222871	0.682304
HES4	0.525685	0.682294
ABHD2	-0.1654	0.682188
AL133551.1	2.038728	0.681594
CBX8	0.176589	0.681594
RPL38	0.18113	0.681437
AC010809.1	0.77722	0.681242
AC119751.5	1.952721	0.681213
ISCA1P1	-3.64124	0.681202
PRRG2	1.605742	0.681136
CCDC3	0.275491	0.681094
PKD1P1	-1.88805	0.68094
CHKB-DT	0.79953	0.680934
ABCC4	-0.21576	0.680797
SRPK2	0.133107	0.680225
ITIH3	-2.06066	0.680163
SLC25A34	-0.63789	0.68015
ZNF182	-0.21559	0.679919
NA	0.743216	0.679834
AC003986.3	-1.16786	0.679649
SNHG29	0.125564	0.679625
AL512288.1	2.404793	0.679399
AC007308.1	-0.57476	0.679375
ZNF354A	0.229327	0.679171
NA	3.060306	0.679028
PDZD7	0.377816	0.67902
LPO	0.530201	0.678977
AC244669.1	0.694989	0.678622
ADM5	-0.81517	0.678542
ZNF107	-0.24752	0.678371

RPS20P14	0.929704	0.678258
BGN	-0.61148	0.678133
SLIT3	-0.13407	0.677896
SIRT3	-0.20697	0.677707
AC011005.4	-0.66832	0.677663
ERVH48-1	-2.31023	0.677444
DOC2GP	-1.54768	0.677305
MLLT10	-0.16348	0.677271
AC093627.5	-0.55076	0.677058
PRCC	-0.14793	0.676973
SAP30BP	0.131663	0.676671
RPL27A	0.130715	0.676421
ZBTB33	0.168413	0.676336
CCDC73	0.245985	0.67623
MCHR1	1.356177	0.676187
GPM6B	0.169342	0.676164
NUP153	-0.13296	0.67607
RNF32	-0.34022	0.675985
MIS12	0.163979	0.675978
NA	0.650398	0.675887
PICART1	-2.72644	0.675849
NA	0.236298	0.675722
TMEM19	-0.22102	0.675604
ZBTB14	0.22428	0.675515
LPAR3	-0.92174	0.67549
SLC20A1	-0.16954	0.675343
AC060780.3	-1.2669	0.675292
AC002519.1	3.242373	0.675203
TPM3P6	1.931272	0.675129
C17orf80	-0.14355	0.674994
PIP5K1C	-0.17896	0.67498
SUSD6	-0.23866	0.674959
AC013391.3	-1.05991	0.674913
AC073389.2	0.613237	0.674839
RGS4	0.132592	0.674833
AL031666.1	2.898816	0.674773
MB	1.313217	0.674653
AL358333.3	-0.32743	0.674379
PRDM15	-0.2058	0.674375
LAMB1	-0.11227	0.674243
MALT1	0.262648	0.673948
OSBPL8	0.1458	0.673947
TPT1	0.11499	0.673793
MICB	-0.75344	0.673704
Z82188.2	-0.37297	0.67361
NA	0.518378	0.673572
STAT4	-0.83596	0.673431
RBP5	0.778724	0.672998
AL359921.1	0.822348	0.672861
AC019080.4	2.424327	0.672814

GKAP1	0.363346	0.672641
HYKK	-0.785	0.672418
FAS	-0.30794	0.672353
RF00019	-2.62057	0.672138
OBP2B	3.24236	0.672096
LINC00487	3.24236	0.672096
CDH4	-1.99932	0.672055
AC012508.2	1.114795	0.67199
ITGAM	3.081887	0.671913
AC115837.1	0.995977	0.671836
RF00019	-1.64398	0.671765
FKBP9P1	1.541597	0.671698
SLC25A22	-0.20675	0.671615
ANKRD44-IT1	-2.88469	0.671578
AC025034.1	-1.73056	0.671513
EPB41L2	-1.03582	0.671435
SIK1	-0.45095	0.671112
AQP9	-2.36652	0.671098
WNT3	-0.28006	0.670839
AC068790.7	-1.04132	0.670839
AC079322.1	-2.39365	0.670693
RIPPLY3	2.910111	0.670655
GRIK3	0.36748	0.67056
FBXO45	-0.17122	0.670554
PLAG1	2.181852	0.670359
RIMKLA	-0.22999	0.670304
NRBF2P5	-1.63878	0.670106
DGCR6L	0.193195	0.670066
SLCO1B1	3.667219	0.669971
C1QL2	3.667219	0.669971
NEURL3	3.667219	0.669971
MIR1302-3	3.667219	0.669971
CDC42-IT1	3.667219	0.669971
AL031005.1	3.667219	0.669971
AL050327.1	3.667219	0.669971
SPINK1	3.644566	0.66997
NA	3.644566	0.66997
NA	-2.53408	0.66974
NA	-0.23967	0.669646
MX1	-2.22375	0.66957
IL17REL	2.418403	0.669482
LINC01089	0.25508	0.669344
COQ7	0.191566	0.669236
C1RL-AS1	0.4362	0.66912
HMGCLL1	1.227426	0.668964
NA	-0.93836	0.668896
ALKBH6	-0.59297	0.668771
ZSCAN16	0.261148	0.668726
AKR1C3	0.310356	0.668451
BLK	-0.30496	0.66841

ZNF226	0.183945	0.668342
HYI	-0.21135	0.668332
NDUFA6	0.239591	0.668297
ACTN3	3.038962	0.668283
AC005722.3	3.038962	0.668283
AC004832.4	-2.90409	0.668042
DOK6	-0.17454	0.668003
CSPG4	0.475247	0.667716
SMURF2	-0.16715	0.667689
AC130651.1	2.088261	0.667657
NA	3.073591	0.667607
KPNA3	-0.1616	0.667573
AC004846.1	2.618935	0.667497
PPIE	0.161734	0.667311
C9orf139	-2.02246	0.667128
MAP3K8	0.323473	0.667024
NKAIN1	0.219688	0.666956
SPZ1	2.91008	0.666841
AC068025.1	0.602141	0.666376
AC007389.1	1.643713	0.666166
AC003989.2	2.928821	0.666014
ANO10	-0.23046	0.665874
AC104407.1	0.749975	0.665854
TRIML2	-3.61779	0.665718
MIR431	2.256852	0.665681
SLC38A2	0.136864	0.66552
AC010336.2	-1.49732	0.665446
PANK2	0.169518	0.665415
SEPHS2	0.14793	0.665395
RLIM	-0.13717	0.665355
NDUFA2	0.192237	0.66502
RN7SL5P	1.000302	0.664842
MRPL21	0.1756	0.664643
TMEM222	-0.18341	0.664353
RF00012	1.424784	0.664196
NA	-0.99009	0.664193
AC103706.1	0.809996	0.66418
LRRC19	1.947408	0.663995
HHLA3	-0.53202	0.663646
CHCHD10	-0.24754	0.663482
SPATA5	-0.21398	0.663398
AP006216.1	-1.66317	0.663372
MAP4K3-DT	-0.545	0.663269
STXBP2	1.518383	0.663142
MAST3	-0.24617	0.663098
AC008440.2	0.271807	0.662727
AC093791.1	3.02133	0.66269
JUND	0.194348	0.662641
AC097523.1	2.928815	0.66257
ABCG8	3.039402	0.66224

S100G	3.621524	0.662229
RN7SKP64	3.621524	0.662229
NA	3.621524	0.662229
RNU6-353P	3.024718	0.662217
ACP5	-0.96473	0.662132
HSPE1P2	3.039409	0.662034
AL365205.2	-0.80557	0.661949
FAM92B	-1.13752	0.661945
AL356512.1	0.477972	0.661761
AC002470.1	1.890823	0.661693
IL1A	1.586153	0.661525
ITFG2-AS1	0.358941	0.661365
ZC3H15	0.150512	0.661282
TXNDC17	0.171253	0.661081
KCTD17	0.150271	0.66099
ADAMTSL1	-0.42471	0.660844
PPP2R5E	-0.15508	0.66052
LMO4	0.585311	0.660518
SLC18A3	0.619782	0.660498
CRMP1	-0.13204	0.660416
PLBD1	-2.34675	0.660333
RPL15P18	1.358432	0.660305
ZNF777	-0.23665	0.660074
DNAJC14	-0.1494	0.660024
AC078778.1	1.507381	0.659844
MYLK	-0.493	0.659766
FAM129C	2.703239	0.659657
BCAS4	-0.40127	0.659475
TSHZ2	-0.1822	0.659215
CD3EAP	-0.17122	0.65917
ITGA5	-0.27622	0.659068
ZNF587B	-0.26617	0.659067
NA	0.605825	0.659032
AC025370.1	-0.74402	0.659023
MRPL11	0.196911	0.658987
AC021242.3	0.450993	0.658928
PRR4	0.412516	0.658811
TPP1	-0.19599	0.658769
MAPT	-0.27908	0.65876
LINC00472	1.345998	0.658729
AC097505.1	2.389733	0.65862
LINC00314	3.640251	0.658561
RAMP3	-0.4487	0.658417
AL390334.1	-2.29488	0.658278
ZNF841	0.237172	0.658187
PABPC5	-2.01928	0.657958
TRIP13	-0.16687	0.657867
AC083949.1	0.261	0.657849
AC131182.1	3.003545	0.657847
AC015813.1	-0.31185	0.6578

AL445231.1	-1.93464	0.657775
TCEAL2	3.478045	0.657753
AC026992.2	3.478045	0.657753
KCNK1	0.282786	0.657713
AC046130.2	2.253842	0.657688
NA	1.431132	0.657639
AP002530.1	-2.86415	0.657367
NFIL3	0.267207	0.657264
PSMD8	0.123702	0.657254
Z98884.2	1.68024	0.657196
IFT20	0.157414	0.65698
GUCA1B	-0.7219	0.656977
CATSPER2P1	0.196654	0.656972
BMF	0.240624	0.656951
AP005018.2	-0.65781	0.656836
SMC4	-0.16803	0.656832
SDCBP	0.129933	0.656762
CPEB2	-0.23253	0.656693
UGCG	-0.16963	0.656654
LRRN2	0.530149	0.656646
AC099792.1	0.972307	0.656631
CDH13	-1.17888	0.65663
NA	-1.60185	0.656561
FAM117A	0.266101	0.656532
RPSAP33	-3.45766	0.656491
POLR2J2	-3.45766	0.656491
WDR45BP1	-3.45766	0.656491
SLC4A8	0.25939	0.656486
RPL24	0.130105	0.656187
RPL22P2	-2.34554	0.656127
SEMA3F	-0.64927	0.656075
LIPH	2.532056	0.656001
LINGO2	-0.28779	0.655988
NA	-1.60349	0.655974
CTC1	0.229566	0.655932
LIF	0.207194	0.655873
ULK4P3	-2.0409	0.655775
ZNF672	-0.18951	0.655707
RPIA	0.160364	0.655512
BCL2L11	-0.16795	0.655388
TGM2	-0.4379	0.655311
MAP11	-0.15802	0.655241
ADAMTS9	-0.11333	0.655208
PLEKHH1	0.236661	0.655121
RTL1	-0.54263	0.655055
AC126177.6	1.914489	0.654999
RP1L1	2.42583	0.654939
TTC25	-0.4739	0.65484
TFIP11	-0.15078	0.65483
RIT1	0.154186	0.65474

FNDC3A	0.134226	0.654628
ICAM4	0.992305	0.654608
DIP2A	-0.17389	0.654359
AL139241.1	1.663527	0.654339
TLE3	-0.21874	0.654238
NA	0.837593	0.654232
AC023790.2	-1.64688	0.654105
NNAT	0.157632	0.65405
BMX	3.003431	0.65402
RPS3	0.129855	0.653995
YPEL3	0.197483	0.653908
AL139811.1	2.396191	0.6539
AC022868.1	2.871982	0.653753
AP003064.1	0.514219	0.653673
CCDC184	0.331409	0.653592
PCLO	-0.21767	0.653563
IL17RD	-0.24714	0.653292
CYB5R3	-0.15741	0.65308
LRRC34	0.274056	0.652661
AMER1	0.186437	0.652621
CFAP44	-0.19665	0.652619
AL161729.1	0.437396	0.652618
AL606834.2	-1.24179	0.652504
AC115220.1	3.606951	0.652446
AC073575.1	3.606951	0.652446
AL157911.1	3.606951	0.652446
L1CAM	-0.1421	0.652413
TBCEL	-0.19835	0.651856
OR7E126P	1.472843	0.651555
CTSZ	-1.31114	0.651395
RPL17P50	-1.93374	0.651262
LRRC49	-0.19173	0.651168
RF00019	3.598068	0.651017
AL139317.2	3.598068	0.651017
RASGRF1	-2.37962	0.650933
KLC3	-0.31392	0.650917
VAMP3	-0.19114	0.650677
FBXL21P	0.347815	0.650437
WDFY3-AS2	0.817004	0.650321
Z98752.1	-1.02678	0.650255
RTN4RL1	2.207636	0.650142
SNORA66	-1.44192	0.649935
AC010907.2	-1.20454	0.649765
IBTK	0.175593	0.649686
ZNF542P	0.206428	0.649538
NR2F1	0.184415	0.649534
RPS12	0.12822	0.649435
MPST	0.171127	0.64941
N4BP2L1	0.270069	0.649365
FBN2	-1.14338	0.649355

AC114956.3	0.537325	0.649154
RF00072	-3.57543	0.648956
AC093423.1	-3.57543	0.648956
AC093249.3	-3.57543	0.648956
COPG2	0.192782	0.648787
CAMK1G	-0.63036	0.648733
AL603839.3	1.030586	0.648695
AC009570.1	1.408051	0.648634
QDPR	0.186266	0.648591
AC012510.1	-0.4272	0.648535
CH25H	-3.57401	0.648277
RPS10P3	1.491691	0.648078
AP000769.1	-1.0718	0.64806
FAM86C2P	-0.44469	0.647948
DENND5B-AS1	-0.70193	0.64784
ACY3	-3.60227	0.647725
SPTLC3	-3.60227	0.647725
AC116651.1	-3.60227	0.647725
AC040169.2	3.616983	0.647707
DHPS	0.127037	0.647698
WDR78	-0.55974	0.64749
RNF111	-0.16019	0.647453
ATP5MC3	0.155827	0.647361
DDTP1	2.996074	0.647346
AL034370.1	-3.40636	0.647337
NA	0.47654	0.646936
KIF21B	-0.41901	0.64686
PHF13	-0.21131	0.64663
AC006146.1	-0.46543	0.646625
ERMAP	-0.22904	0.646527
CHST10	0.21026	0.646524
DOCK4-AS1	1.499323	0.646214
AC022558.2	-3.57681	0.646101
AC004086.1	0.120832	0.646078
SNX32	0.119429	0.646034
RABGGTA	-0.21737	0.645959
ZNF70	-0.36386	0.645924
ITLN2	-3.77134	0.645911
DMBT1	-3.77134	0.645911
ZNF812P	-3.77134	0.645911
AL359091.2	-3.77134	0.645911
AC016822.1	-3.77134	0.645911
HNRNPA1P70	-3.77134	0.645911
SCARNA21	-3.77134	0.645911
AC093627.7	-3.77134	0.645911
MINAR1	-0.3421	0.64569
PNKP	0.218108	0.64567
FUCA1	-0.28171	0.645453
UPP1	0.449566	0.645248
HS3ST3A1	-0.70678	0.644973

AC103925.1	-2.82692	0.644912
CTPS2	0.174996	0.644825
AC074143.1	-0.19083	0.644823
BAG2	0.188773	0.644731
RNU2-51P	2.476599	0.644731
AC093734.1	2.476599	0.644731
P4HTM	-1.30121	0.64471
LAMTOR5	0.147919	0.644539
HIVEP2	0.180661	0.644427
ZNF806	-0.74261	0.644413
RPL11	0.134529	0.644385
NA	-1.04286	0.644381
YPEL2	0.174958	0.644148
ACKR2	-3.57255	0.644079
AP005131.7	-3.57255	0.644079
ELOB	0.140768	0.64407
SMIM22	2.48675	0.643986
ARL14EP	0.188319	0.643953
KCNC4	-0.424	0.643935
AC005076.1	0.718116	0.643889
ACP7	-2.97804	0.643827
RPS20P2	-3.59387	0.6438
PPIAP91	-3.59387	0.6438
ARLNC1	-3.59387	0.6438
H3F3AP2	-3.59387	0.6438
OTUD6B-AS1	0.2232	0.643743
NA	-1.17448	0.643689
DPH6	-0.32054	0.643548
LINC00475	-1.77918	0.643421
CLCN5	0.18661	0.643369
SMC3	0.151023	0.642993
RPL13	0.139676	0.642974
DPP3	-0.15991	0.642788
SPTBN4	0.286525	0.642757
HMGB1P8	-1.23544	0.642749
FRY-AS1	0.923656	0.642702
TFDP2	0.150912	0.642691
AC083798.2	-0.1723	0.642678
CACNG8	-0.2096	0.642519
ACTL10	0.485486	0.642289
OCIAD2	0.161951	0.642268
PCOTH	-1.22238	0.642262
PTPN13	0.149921	0.642115
RSKR	0.751622	0.642105
C1GALT1C1	0.269581	0.642097
SRP19	0.267617	0.642061
MGAT4A	-0.33893	0.641994
TPH1	1.878876	0.641667
AC005009.2	-1.18904	0.641656
NA	1.501074	0.641607

UBASH3B	-0.64132	0.641495
VCAM1	0.311927	0.641422
MOCS2	-0.1959	0.641378
UQCRQ	0.14453	0.641348
DAPP1	-3.76122	0.641282
PIP5K1P1	-3.76122	0.641282
ADH5P3	-3.76122	0.641282
CROCCP1	-3.76122	0.641282
CAP1P2	-3.76122	0.641282
Z98752.2	-3.76122	0.641282
LINC01524	-3.76122	0.641282
BX284668.4	-3.76122	0.641282
RN7SL390P	-3.76122	0.641282
AC005920.1	-3.76122	0.641282
AC021491.1	-3.76122	0.641282
AC008871.1	-3.76122	0.641282
AC126177.2	-3.76122	0.641282
AC134312.3	-3.76122	0.641282
CDC14A	-0.34297	0.641271
AL109615.3	1.084784	0.64114
NA	0.741638	0.64112
SHMT2	0.15493	0.641087
PAM16	0.535315	0.641063
WNK3	0.245956	0.640899
ADAMTS6	0.591367	0.640818
AC005154.4	-1.56645	0.640643
ZBTB32	1.282116	0.640548
SERPINF1	0.234768	0.640428
AL445433.2	0.259601	0.640345
SLC10A4	-0.20119	0.640337
TUBE1	0.194154	0.640309
PRPSAP1	-0.13934	0.64024
NA	-3.4048	0.640101
RF00019	-3.4048	0.640101
AC095350.1	-3.4048	0.640101
MICOS13	0.167367	0.640043
TMEM203	-0.16661	0.639929
IGFBP5	-0.33876	0.639923
RBM20	-0.17072	0.639895
AC073657.1	0.76554	0.639876
DPH7	0.164155	0.639858
DUSP10	0.313641	0.639756
LINC01694	0.991	0.639651
NUP214	-0.13815	0.639648
CAMTA1	0.200629	0.63957
ERG	1.144759	0.639533
TARS	0.125495	0.639488
REM2	0.623163	0.639346
NA	1.827382	0.639318
ANKRD40	-0.13472	0.639254

MTMR1	-0.18073	0.639069
NCAM1-AS1	-0.31142	0.639003
TRIT1	0.209315	0.638856
TF	-2.44166	0.638802
AIFM2	-0.23278	0.638758
AC022748.1	-1.81039	0.638511
RPL36	0.145757	0.638437
NFS1	-0.23437	0.638376
SH2B3	-0.22913	0.638254
MTMR4	0.134086	0.638231
LINC01424	-1.14134	0.638217
LMBR1	-0.13523	0.63813
RF00019	-2.98992	0.638122
HFM1	0.338627	0.638063
VPS8	0.178743	0.63795
PLK4	0.201318	0.637948
UPRT	0.230768	0.637846
NA	0.453629	0.63779
ST13P6	2.293114	0.637742
NA	-0.38787	0.637678
CCDC112	0.225582	0.637667
RPL12P1	-1.09514	0.637317
NA	0.461836	0.637178
XPC	-0.13872	0.637146
MEGF11	0.807271	0.637032
AC005046.1	-0.39889	0.637008
ZFPM1	-0.19332	0.636877
AC116533.1	0.333637	0.636868
AL139260.1	-0.9074	0.636828
SNORD6	0.901323	0.636798
PHB2	0.142354	0.636745
RETREG1	0.409095	0.63667
RSPO3	-3.57811	0.636434
ABCA4	-3.57811	0.636434
AC009271.1	-3.57811	0.636434
C3orf14	0.232646	0.636331
ZNF132	-0.28441	0.636329
PCSK1N	0.223039	0.635967
ITGB8	0.29731	0.635851
IMPDH1P10	0.561175	0.635692
Z97055.2	-0.9383	0.635639
AC015802.5	-0.65103	0.635504
MYOF	-0.58647	0.635346
RPL15	0.126431	0.635329
PELI1	0.165492	0.635179
IGHV3-36	2.973773	0.635129
SOAT2	2.048795	0.635049
CAVIN3	-1.00052	0.63501
LINC01004	-0.46189	0.634979
AP1S3	-0.50891	0.634936

C17orf49	-0.59317	0.634906
C5orf66	0.137381	0.634882
FDFT1	0.179847	0.634834
AC002401.1	-0.23421	0.634748
NAIF1	-0.258	0.634256
NA	1.176681	0.634155
SPATA17-AS1	-1.04874	0.634031
MAP4	-0.13522	0.634028
APOBEC3D	-2.98005	0.633686
NA	1.267462	0.633629
RPS2P7	-2.02694	0.633386
LRP8	-0.17582	0.633301
AC004877.1	-0.32896	0.63325
AC025442.1	0.392882	0.633204
TKTL1	-3.571	0.633152
KCNK4	-3.571	0.633152
SATB2-AS1	-3.571	0.633152
BNIP3P41	-3.571	0.633152
NA	-3.571	0.633152
AL513550.1	0.239076	0.633124
LRRC75B	0.291014	0.632955
SPTBN1	-0.11878	0.632877
VAV3	-0.47223	0.632868
IFIH1	0.603634	0.632849
PGAP2	-0.21447	0.632835
GMDS-DT	0.732024	0.632792
AC135178.3	0.443029	0.632711
AL360091.3	2.951639	0.632305
MMS22L	-0.20162	0.632245
AL049780.2	1.588923	0.63223
AL035456.1	-1.79491	0.632161
KIF24	-0.23397	0.632088
LINC01695	0.768025	0.632051
AL359955.1	2.341593	0.631993
AATK	0.332973	0.63179
AC006252.1	0.978074	0.63175
TWNK	-0.13131	0.631628
COX17P1	2.973818	0.63153
MT-TT	-0.6942	0.631526
PABPC4L	-1.42248	0.631443
SPG21	0.149433	0.631375
AC233266.1	0.57747	0.631294
NA	1.970194	0.631282
PLPPR4	-0.13319	0.631246
CCNYL1	0.224141	0.631244
SCARNA17	1.344447	0.631237
EDRF1	0.146679	0.631226
GPI	-0.13244	0.63114
TMEM160	0.225239	0.631112
AC117382.2	-2.12928	0.631028

AC006059.1	-0.56072	0.630922
AC011491.1	3.583796	0.630917
AL592148.1	3.583796	0.630917
LINC00167	3.583796	0.630917
ALG9-IT1	3.583796	0.630917
AC084824.2	3.583796	0.630917
C1QBPP2	-1.42245	0.630911
RN7SL146P	-1.87369	0.630903
H3F3A	0.110476	0.630878
LINC02249	0.632576	0.630782
FOPNL	0.190378	0.630575
GSTO1	0.172336	0.630553
RLN1	-3.13291	0.630492
MIR3665	0.861408	0.630477
SDAD1P1	-0.4077	0.630353
PPIB	0.11421	0.630335
REG3G	3.559054	0.630251
PGM5	3.559054	0.630251
METTL21AP1	3.559054	0.630251
HMGB1P49	3.559054	0.630251
NA	3.559054	0.630251
WDR86-AS1	0.602497	0.630187
AC132938.1	-2.36975	0.630005
SRSF10	-0.2178	0.629909
CD74	-0.89762	0.629775
PRPF8	-0.14194	0.629671
PCDHGA11	-2.34134	0.629359
SORL1	-0.34131	0.629319
AP000346.1	-2.05563	0.629252
AL512343.2	0.969016	0.629008
AC097359.3	-2.97998	0.628976
RAB32	0.177239	0.628893
MEGF8	-0.1423	0.628748
AC090912.2	2.95168	0.628618
ARL2	-0.21013	0.628379
PPP1R16B	-0.15744	0.628348
CASZ1	-0.30916	0.628333
SEPT1	0.416558	0.628315
AC104564.2	2.042264	0.628058
MRPL15	0.188169	0.628035
ESCO1	0.183558	0.628025
AC078899.1	2.456764	0.627967
MYO5BP2	-3.37933	0.627951
VLDLR-AS1	0.304881	0.62772
AC145207.6	0.4733	0.627439
CA11	0.228585	0.627343
NA	1.979711	0.627342
FAM217B	-0.1735	0.627305
STPG4	1.745611	0.627237
ANGPT2	-0.67027	0.627077

SGMS1	0.233507	0.627011
MIR4740	2.852068	0.62701
IRS3P	-3.13208	0.626844
SIGIRR	0.309516	0.626751
AC022613.2	-1.12513	0.626503
COL13A1	-0.91558	0.626497
NEXN	-0.32058	0.626267
AC004217.1	0.773978	0.626253
VAC14	-0.16344	0.626167
TM6SF1	0.292025	0.626109
IGF2-AS	2.13003	0.626026
FLOT1	0.144613	0.626025
ERCC2	-0.16107	0.625809
RN7SL508P	2.823717	0.625662
AL157392.1	-2.97236	0.625593
LINC01583	1.025652	0.625363
PLIN1	-1.73131	0.625256
JMJD1C	0.13118	0.625215
BTBD10	0.184696	0.625106
GALNT5	-0.80743	0.625091
AGAP1	-0.15404	0.625039
LINC01901	2.563658	0.624596
HINT1	0.139036	0.624509
JMJD7-PLA2G4B	-0.48831	0.624486
PPT2	0.217783	0.624332
MTBP	-0.25592	0.624213
ZNF137P	-0.62432	0.624064
PRB3	3.126321	0.624039
NA	3.126321	0.624039
MRAS	-0.16109	0.624
ZP1	-2.50265	0.623916
MIR4767	1.294316	0.623864
SF3B1	0.111204	0.623737
AC019205.1	-0.45876	0.623535
NA	-2.61988	0.623486
TSGA10	0.387052	0.623025
FBXL16	-0.21946	0.622897
PMEPA1	-0.17781	0.622882
ADCY7	-0.16695	0.622834
MIF-AS1	0.16396	0.622699
ST7-OT4	1.063924	0.622356
GCLM	-0.21057	0.622354
GRB10	-2.97231	0.622299
AC046134.1	-1.44784	0.622271
ITPRID2	0.179024	0.622195
AC022126.1	0.58517	0.621992
COL16A1	-0.24371	0.621938
TAS2R3	-1.24335	0.621905
AC010642.1	-1.02705	0.621812
NA	2.832391	0.621769

AC099811.3	-2.92441	0.621615
MESP1	-1.2257	0.621454
ENKUR	0.861897	0.621364
ZIC4-AS1	-1.1532	0.621335
AC007114.1	1.047284	0.621252
SVBP	0.24541	0.621163
PIANP	-0.2514	0.621146
PNPLA8	0.163547	0.621137
CXorf58	0.670054	0.621132
AC010320.1	3.400048	0.621121
AC091133.5	3.400048	0.621121
GORASP2	0.133549	0.621084
FBXL2	-0.31686	0.621053
KIAA1614-AS1	-0.43429	0.62105
EEF1DP2	1.08727	0.621047
RF00019	3.126465	0.621032
PXMP2	0.536256	0.621031
AMMECR1L	-0.14828	0.620983
CLTCL1	-0.38659	0.620956
AC010531.5	-1.3643	0.620931
SLC6A1	-3.38246	0.620921
PPIAP20	-3.38246	0.620921
AL035071.2	-3.38246	0.620921
TAS2R20	-0.8971	0.620917
ETS2	-0.13713	0.620873
BCAN	-0.39856	0.620782
NSG1	0.21305	0.620782
NA	1.689258	0.620777
XXYLT1-AS1	-2.83016	0.620772
RF00019	3.538801	0.620568
RN7SL68P	3.538801	0.620568
RF00019	3.538801	0.620568
PPIAP50	3.538801	0.620568
C19orf66	-0.26089	0.620462
GRK1	1.101357	0.620372
MYO15A	2.040029	0.620358
BDNF	-0.40866	0.620093
CTAGE7P	-0.64478	0.619976
GRASP	0.34062	0.619853
PPRC1	-0.16347	0.619825
COA5	0.155465	0.619676
AL138767.1	1.101413	0.619573
AC117382.1	2.27563	0.619564
PLCD1	0.315916	0.619459
TMC3	1.023126	0.619422
OTOF	2.973693	0.619278
MIR136	-2.41199	0.619137
MTCO2P27	2.973686	0.619071
CLK2P1	2.973686	0.619071
AC040160.1	-0.54916	0.619026

ACTL6B	0.202487	0.619022
CHPF	-0.16181	0.61901
LONRF2	0.210318	0.618968
UQCRBP1	-3.37767	0.618714
MARVELD1	-0.17664	0.618423
BMF-AS1	0.378909	0.618322
RBFOX3	-0.53355	0.618182
RHBDL3	-0.37967	0.618127
THAP9	0.307541	0.618036
PDCD2L	0.372268	0.617994
CHAT	-2.21544	0.617774
AL513534.2	-0.31372	0.617748
DNAH10	0.308769	0.617712
FAM111A	-0.28488	0.617668
MIIP	0.186724	0.617569
SKP1P1	-1.13424	0.617503
TRIM71	-1.97179	0.617442
KYAT1	0.230704	0.617306
TP73-AS1	-0.16944	0.617237
AL136531.1	-1.07224	0.617211
SPAG6	0.642931	0.617172
GBF1	-0.18413	0.617126
E2F5	0.184321	0.617098
AC107871.2	0.740691	0.616938
ZNF560	-0.61965	0.616842
AC107398.3	-2.92452	0.616833
NA	0.325266	0.616703
RPL26P6	-1.43321	0.616594
RASEF	-0.37615	0.616563
CNEP1R1	-0.23538	0.616501
NA	0.590765	0.616433
SLC35E2B	-0.1645	0.61631
NONOP2	1.104804	0.616224
VWDE	0.575138	0.616145
AC116366.1	0.694061	0.616127
MIR1184-1	-0.64006	0.615996
TBC1D9B	-0.12832	0.615869
PLK5	-2.92226	0.615838
HESX1	0.648664	0.615809
TBC1D22A	-0.20644	0.615802
XK	2.433558	0.615683
MT-ND5	-0.11067	0.615496
AC092422.1	-2.49436	0.615194
KRT86	3.549537	0.615124
TRBV20OR9-2	3.549537	0.615124
BNIP3P40	3.549537	0.615124
AL139246.5	3.549537	0.615124
SLC35B1	0.137218	0.615124
NA	0.437571	0.615006
TEAD4	0.244165	0.614969

AC024361.3	-0.99661	0.61493
C2orf15	-0.77186	0.614804
RPL9	0.132971	0.614753
AC009518.1	-1.24748	0.614689
NA	-0.2338	0.614679
AC073072.2	-2.11996	0.614616
CSF1R	1.358377	0.614424
BAIAP2L2	-0.95754	0.614175
KBTBD2	-0.13072	0.614061
NA	0.238442	0.613994
AC147651.1	1.590158	0.613956
SLC35A5	0.148643	0.613767
SPTB	0.373058	0.613741
PIGP	-0.23621	0.613738
SMC1B	1.757156	0.613714
C2orf16	0.454654	0.61363
GRAMD2A	2.238344	0.613558
AC008667.4	-2.92459	0.613491
MCM3AP	-0.1312	0.613388
C5orf47	-0.99641	0.613375
SLC47A1	-0.64277	0.613307
APOPT1	0.261569	0.613104
SHB	-0.34245	0.613013
MROH1	0.390869	0.613005
EXOSC5	0.147211	0.613001
NA	2.484185	0.612984
SPRYD4	-0.24251	0.612938
DOCK5	-0.19548	0.612928
CCDC181	0.290116	0.612894
SERTAD4	0.20455	0.612863
DZIP1L	0.295831	0.612796
BCYRN1	0.195525	0.612782
AC027020.1	-1.33406	0.612292
KANSL3	-0.14381	0.612285
CFAP410	0.200503	0.612226
HMGB3P24	1.616275	0.612106
FAM71F1	3.714394	0.612075
AQP2	3.714394	0.612075
MUC3A	3.714394	0.612075
HIST1H2BL	3.714394	0.612075
AC068051.1	3.714394	0.612075
MIR760	3.714394	0.612075
TCEA1P3	3.714394	0.612075
NA	3.714394	0.612075
AC006947.1	3.714394	0.612075
AL133260.2	3.714394	0.612075
RN7SL265P	3.714394	0.612075
RNU6-539P	3.714394	0.612075
AC025431.1	3.714394	0.612075
NA	3.714394	0.612075

HERC2P8	0.57515	0.612051
TAP1	-0.24528	0.612038
AL360181.3	-2.88763	0.611981
DDRGK1	0.19902	0.611676
MTSS1	0.258236	0.611643
AC006148.1	-0.47766	0.611613
ARHGEF9	0.202264	0.611607
KIF13B	-0.17846	0.611475
SCUBE3	-0.42312	0.61135
REEP6	0.276161	0.611294
C11orf71	-0.53601	0.611271
LRRC4B	0.241459	0.611042
AC015727.1	2.162278	0.611027
FGF7	0.536334	0.610758
EIF3D	0.112846	0.610617
BBS7	0.239118	0.610312
ATG5	0.199579	0.61018
MIR4746	-0.98773	0.610159
FOXN3-AS1	0.637906	0.610116
AC147651.2	1.751582	0.610035
ALG8	0.144505	0.609969
CLIP4	0.18609	0.609952
LINC02482	0.531241	0.609904
CABP7	0.289432	0.609774
DPH3	-0.18289	0.609684
MTFMT	0.23425	0.609214
MAPRE2	-0.16877	0.609108
CACHD1	-0.18484	0.609108
ADAM9	0.143126	0.609008
MIR3190	2.427628	0.608912
SPPL3	0.191252	0.608856
EMC3	-0.16005	0.608763
RNF224	0.834985	0.608703
TRIM16L	0.177801	0.608655
SNX9	0.147008	0.608641
CTF1	0.424132	0.608563
CDK12	-0.14125	0.608293
MED21	0.171297	0.608222
ARHGAP26	0.211092	0.608199
STXBP5L	-0.32545	0.608189
MFSD13B	-1.83209	0.608177
RPL26P29	3.090427	0.608148
ADAMTS9-AS1	-0.23064	0.607894
PLLP	-0.6307	0.607783
HES6	0.188155	0.607751
TAF6	-0.14749	0.607747
ARHGEF16	-0.69095	0.607711
NA	-1.93703	0.607597
HAGHL	0.14603	0.607557
TRIP10	0.172461	0.607533

RWDD4	0.190524	0.607517
SNORD7	2.947461	0.607436
YPEL5	0.150299	0.607426
AL020995.1	0.571441	0.607377
ZNF827	-0.19734	0.607278
CNNM3	-0.2215	0.607261
AP001010.1	0.568516	0.607158
SH3PXD2A	-0.17683	0.607109
IZUMO1	0.696631	0.607051
NA	-0.66758	0.60686
LINC02037	-1.08905	0.606843
MSANTD1	-1.74265	0.606841
GPR1	1.56069	0.606811
AC099811.1	-2.44184	0.606756
TTYH3	-0.14198	0.606464
RMDN2-AS1	-2.00996	0.606382
EID2	0.208187	0.606299
PGAM5	-0.15057	0.606148
AL021368.2	0.792451	0.60606
VAC14-AS1	0.502098	0.606059
IGSF5	1.910216	0.605975
CTTNBP2NL	-0.15499	0.60597
CCDC80	-0.59042	0.605934
EEF1AKMT2	-0.36433	0.605918
ALG10	-0.33203	0.605855
RPS7P11	0.751673	0.60575
CICP27	1.71003	0.605582
AC092447.7	-1.24643	0.605535
GLCE	-0.20197	0.605523
SAMD11	-0.13568	0.605407
TMEM150C	-0.45708	0.605349
AC092574.1	1.327074	0.605316
CMTM6	-0.14048	0.605243
KLHL20	0.147679	0.605188
RMC1	-0.16947	0.605096
LINC01585	3.090412	0.605046
PAEP	-2.88307	0.605033
BTG4	-2.88307	0.605033
AL078604.2	-2.88307	0.605033
GPC3	0.388487	0.604991
GARS-DT	0.182392	0.604892
PNMA2	-0.14721	0.604823
RPL18A	0.131319	0.604709
NSMCE4A	0.217605	0.604481
ITGB5	-0.72339	0.604286
TULP2	0.862496	0.604284
USP6	-0.54082	0.604279
RF00019	-2.3724	0.604255
ACSM4	-0.5761	0.604185
AAMDC	-0.24472	0.60392

PISD	0.178794	0.60381
AC098484.1	-0.66913	0.603706
AFF1	-0.15008	0.603454
SERPING1	0.396069	0.603413
PTTG4P	2.766567	0.603326
GRIN3A	-0.60804	0.603314
HMG20B	0.132027	0.603282
TOMM34	-0.14008	0.60323
AC010343.1	0.81835	0.603221
AC105285.1	0.39496	0.603093
DDB2	-0.21924	0.60297
CCDC154	0.826749	0.602915
AC010185.1	0.476093	0.602795
UOX	-1.61149	0.602619
PRR5	-0.37228	0.602387
OR13E1P	3.077132	0.602361
RN7SL4P	1.066248	0.602356
RIPK2	0.198778	0.60226
PSG5	-1.73606	0.602197
NA	0.232122	0.602196
AC008105.2	1.114903	0.60216
SETD2	-0.13199	0.602154
AC022148.2	3.689342	0.60205
MIR181A2HG	0.723809	0.602026
CLTB	0.158498	0.601939
AL139021.1	0.423892	0.601931
CD163L1	-0.35703	0.601918
DDR2	0.132653	0.60169
LINC00431	-0.98013	0.601678
PLRG1	0.152735	0.601628
KCNH3	0.935599	0.601576
YJEFN3	-0.28432	0.601285
DCLK1	-0.29588	0.6012
KLHL32	1.060633	0.60119
TRIB3	0.60679	0.601182
GAS2	-0.34711	0.601084
ZNF738	-0.2573	0.601021
ZBTB8B	0.304453	0.600908
SNORA26	0.812756	0.600855
SLC2A8	0.242663	0.600414
PPP1R37	-0.18945	0.600389
DPY19L2P2	0.301888	0.600125
GPR61	-1.07167	0.600092
SAMM50	0.15757	0.600036
AL133284.1	2.143672	0.59998
ACPP	0.645064	0.599943
SZT2	-0.1726	0.599893
RSPH9	0.221064	0.599515
AL606534.2	0.210478	0.59947
DOK3	-0.26998	0.599402

NSUN7	0.47523	0.599391
AL078459.1	-2.00871	0.599233
TTC3P1	0.33305	0.599225
NA	3.076998	0.599188
GMPR2	0.155053	0.59903
LINC00476	0.36518	0.599021
NA	0.603928	0.598915
MHENCR	-0.48696	0.598889
STAT5B	0.152887	0.598835
DAND5	-0.39684	0.598239
AC004771.3	-0.83567	0.598235
MZF1-AS1	0.210321	0.598125
ZNRF2P1	0.508558	0.598026
BZW1P2	0.738916	0.597871
SNHG8	0.169369	0.597819
COL6A3	-0.92406	0.597766
CARS-AS1	0.230209	0.597738
RPS9	0.122249	0.597722
TVP23A	0.179904	0.597698
MORC1	3.675701	0.59765
AKR1C6P	3.675701	0.59765
SERPINA9	3.675701	0.59765
RNU6-926P	3.675701	0.59765
EIF4A1P8	3.675701	0.59765
LINC01492	3.675701	0.59765
AL021393.1	3.675701	0.59765
FNTAP2	3.675701	0.59765
AL357373.1	3.675701	0.59765
AC093732.1	3.675701	0.59765
AC004231.1	3.675701	0.59765
LCE1F	3.675701	0.59765
AC004223.1	3.675701	0.59765
RPL32P33	3.675701	0.59765
NA	3.675701	0.59765
AP000893.2	3.675701	0.59765
AC009135.1	3.675701	0.59765
AC007687.1	3.675701	0.59765
AL137843.1	3.675701	0.59765
RAP2CP1	3.675701	0.59765
AL683887.1	3.675701	0.59765
AC017033.1	3.675701	0.59765
JPT2	-0.14186	0.597597
CCDC9B	-0.21006	0.597587
ALG3	-0.16159	0.597479
SAPCD2	-0.17885	0.597393
TCEAL5	0.244625	0.59739
AL121672.2	1.395385	0.597364
LRRC37B	0.271874	0.597269
GPR19	-0.34738	0.597201
AC091180.2	-1.14778	0.597171

Z83838.1	-2.73407	0.59704
KLHL22	-0.20836	0.596724
SFXN2	0.220655	0.596712
AC017101.1	0.31338	0.596679
AC067747.1	-2.04519	0.596645
PITRM1-AS1	0.23333	0.59657
HTRA2	0.133363	0.596535
ALMS1	0.175031	0.596501
AC113189.3	-0.77499	0.596383
AC087294.1	1.050472	0.596337
EBF4	0.228829	0.596329
GNG13	-0.77416	0.596181
GPR37	0.54985	0.596156
NDUFB2-AS1	0.147815	0.596093
GPX8	-0.43148	0.596039
MAGEE1	-0.49042	0.596023
POLR2J4	0.287628	0.59602
COQ5	0.194259	0.595988
GAPDHP60	3.503557	0.595953
AC027702.1	-1.28091	0.595644
TMEM159	0.450048	0.595441
NA	1.472753	0.595405
SCIN	-3.48342	0.59512
FMO4	-3.48342	0.59512
RPS4XP8	-3.48342	0.59512
AC104964.3	2.131754	0.594882
PMS2P3	-0.22065	0.594707
MATN1	1.162446	0.594605
CLK1	0.142548	0.59452
TACC1	0.302498	0.594403
SLC38A3	-0.93573	0.594239
RAB27B	-0.41716	0.594142
YAF2	0.181653	0.593986
IL33	1.078606	0.593933
TPI1P2	0.936528	0.593913
AMBRA1	-0.19483	0.593787
AC079766.1	-2.09224	0.593752
BX119917.1	-0.78428	0.593724
TIMM17A	0.136841	0.593711
LINC00702	1.038851	0.593699
DLG3	0.326543	0.593657
KLHL5	0.129834	0.593639
GPRASP1	-0.29756	0.593637
AC040934.1	-1.25412	0.593594
COLEC11	-0.12851	0.593573
KCNQ4	-1.52847	0.593522
C2CD3	0.139286	0.593502
IKZF2	0.695312	0.593412
IL12A	-0.63835	0.593329
NA	-1.99273	0.593326

MIR1276	-3.46014	0.593322
AC104162.1	-2.27218	0.593319
AC092115.2	-0.42582	0.593308
BICDL2	0.95574	0.59305
IFI44	-0.59683	0.593049
TECTA	-0.42847	0.593011
PCDHA9	1.785059	0.592986
AC003102.1	-0.47099	0.592971
PANX2	-0.59369	0.592907
CABYR	0.279711	0.592824
PLCE1-AS1	-1.22385	0.592712
PSPH	0.169798	0.592568
RSAD2	-0.56996	0.592494
ATXN1L	-0.14174	0.592156
S1PR4	2.902171	0.592152
AP002761.3	-0.39339	0.592075
GLUD1P3	0.423197	0.592071
CHD2	0.131139	0.592023
NDUFS2	0.11239	0.591987
CDK15	-3.45706	0.591967
SMOC1	-3.45706	0.591967
AC022973.3	-3.45706	0.591967
AC008740.1	-3.45706	0.591967
SNHG11	0.226975	0.591945
NA	2.172616	0.591817
AC026191.1	0.301649	0.591668
GRIK2	-0.22191	0.591661
HPSE2	0.483193	0.591647
SERINC5	0.360916	0.591585
Z98048.1	-2.86319	0.591479
AC100827.4	-2.86319	0.591479
KLHL8	-0.20893	0.591402
GP5	2.587815	0.591387
NA	-1.99075	0.59114
ETV2	0.764142	0.591134
INPP1	0.269588	0.590884
BMS1P22	0.336013	0.590842
AL049779.1	2.724464	0.590746
NA	-0.22807	0.590662
AC013451.1	-0.67238	0.590619
BMPER	0.368883	0.590574
GPNMB	0.422955	0.590534
AL031705.1	-2.30701	0.590032
AC010978.1	0.273421	0.589936
PPIAP55	2.522494	0.589826
LINC01686	0.907539	0.589779
AC107886.1	-1.42557	0.58971
AP001189.1	-0.9463	0.589669
CD27	0.434509	0.58966
MRPL37	0.147307	0.589578

RPS8	0.123761	0.589538
CFH	0.866879	0.589517
RPS6KA2-IT1	1.037372	0.589419
ZNF66	-0.61496	0.58927
RNA5SP373	-2.85011	0.589239
RPS27A	0.117364	0.589126
ZNF12	0.13669	0.589104
HCG14	2.902349	0.588988
AC003092.1	2.902349	0.588988
C1orf131	-0.18744	0.588841
LDLRAP1	-0.30046	0.588797
GBP7	2.90236	0.588793
ATP7B	-0.26699	0.588678
ASIC1	0.299814	0.588675
CYS1	0.956037	0.588606
C14orf180	-0.28824	0.588461
MIR1-1HG-AS1	-1.24839	0.588461
RPL7AP6	0.432944	0.588458
EIF4EBP1P1	-1.85024	0.588375
AC073254.1	-0.22695	0.588351
SLC49A4	0.308743	0.5883
PLXNA1	-0.17795	0.588257
BLVRA	-0.15716	0.58814
NA	0.381862	0.587841
RNU6-343P	2.874168	0.587711
LINGO4	-0.22626	0.587561
CPB2	2.874178	0.587508
MT1M	-2.40476	0.587497
NA	0.500136	0.587457
NUDT13	0.28302	0.587415
TLR7	-0.55336	0.587378
SLC25A19	-0.19273	0.587373
AC026471.4	-0.55381	0.587343
AC010530.1	-1.35693	0.586995
SEMA4A	-0.75222	0.586954
MAPK6	0.14115	0.586852
DOCK1	-0.14109	0.586847
DNASE1L1	-0.20858	0.586847
FOXP4	-0.22941	0.586744
NT5C3AP1	-2.37789	0.58672
NPIPB2	-2.37789	0.58672
SOGA1	-0.13593	0.586596
HBQ1	0.709782	0.586542
AC092159.2	0.352897	0.586493
ZNF717	-0.37803	0.586444
BTN3A1	-0.19204	0.586411
ORC1	-0.20958	0.58639
IGHV7-40	-0.67421	0.586308
NA	-2.84301	0.58622
PIGL	0.176647	0.5862

MICA	-0.66368	0.586059
AGGF1P2	1.426413	0.585949
FAM110D	-0.90672	0.585875
PIK3R4	-0.17816	0.585774
PMS1	-0.16229	0.58573
HIST1H4C	2.544018	0.585569
BBS10	0.171305	0.585528
NA	-0.81359	0.58542
FAM24B	1.042156	0.585419
RAD51	-0.24798	0.585316
KCNQ5	-0.28272	0.585227
GRB14	-0.65717	0.58512
ZNF784	0.25058	0.584859
HERC2P5	0.363123	0.584775
RAPGEFL1	-0.28659	0.584497
C6orf226	0.344885	0.584425
UCP2	-0.21221	0.584416
DAPK2	0.506729	0.584385
DYNC1H1	-0.12395	0.584342
AC090625.2	-3.43508	0.584247
RPL19P16	2.234769	0.584185
ZNF620	0.197995	0.584081
RN7SL221P	2.882563	0.583972
SLC30A5	-0.13171	0.583911
RNU6-890P	-2.70319	0.583907
C8orf74	2.842885	0.583815
AC093835.1	-2.2626	0.583717
AC139530.1	1.481577	0.583672
LINC00665	-0.21299	0.58323
AC067852.3	0.597729	0.583214
AC034102.7	0.235503	0.583164
AMZ2P1	0.242245	0.582908
AL031768.1	2.163239	0.582809
DCAKD	-0.20408	0.582765
AC119428.2	-1.6349	0.582741
LATS1	-0.14441	0.582733
LHB	-1.02109	0.582703
AC245884.8	1.636328	0.582626
RNF125	-0.52824	0.582509
COQ10B	0.220876	0.582372
AL590652.1	-2.72386	0.582364
NPEPL1	-0.50353	0.582302
GPR39	0.341795	0.582134
FAM120A	-0.12206	0.582132
ZXDC	-0.21254	0.581992
ING3	0.175587	0.581624
RAB43	0.556423	0.581607
HK2	-0.12845	0.581578
NBPF13P	2.141577	0.581529
PGBD4P3	-1.74682	0.581349

HAND1	-0.33498	0.581283
PLEKHH2	0.397198	0.581168
WBP2NL	-1.01598	0.581166
AC092718.2	-1.18983	0.581058
AL512326.1	-2.85009	0.58103
MYO1H	-1.00212	0.580989
DROSHA	-0.13423	0.580892
AC034198.2	-1.21041	0.580853
AC104695.3	2.882728	0.580802
NA	0.828504	0.580703
NA	0.376314	0.580653
METTL7A	0.181719	0.580621
AC009119.2	2.882738	0.580607
ADAMTS19	0.415438	0.580591
Z92544.2	-0.27864	0.580567
CDH5	-1.09456	0.580498
AC010359.1	2.380414	0.58045
VAX2	0.4914	0.580433
PNPO	0.216774	0.580395
AC004453.1	0.723356	0.580312
COX4I1	0.118392	0.580303
AL023754.1	2.842774	0.580281
AL162311.1	2.842767	0.580063
SLC46A1	-0.1567	0.580042
HAND2-AS1	0.102085	0.579983
SEMA6D	0.192092	0.579958
AL132640.2	2.737261	0.579942
CDH7	-1.37022	0.579879
HLA-J	-0.53792	0.579858
TRAK2	0.162458	0.57985
PIWIL1	0.421587	0.579797
NA	-1.45124	0.579712
PLEKHA4	0.234628	0.579656
GPR27	0.353893	0.57961
NA	0.376994	0.579523
AC015912.1	-0.2989	0.579494
AC008676.1	-1.42535	0.579457
KDM3A	0.132091	0.579453
AC073475.1	-1.57535	0.579287
EIF3G	0.122098	0.579129
TRMT11	0.167608	0.579039
PIGH	0.20553	0.579006
KMT2D	-0.18733	0.578974
COL11A1	0.230716	0.57871
GABRR2	1.624365	0.578585
PPIF	-0.13277	0.578458
AC090409.1	-1.21186	0.578312
IGF2R	-0.1488	0.578223
C12orf29	0.170098	0.578222
AC053513.2	0.623282	0.578214

TMEM145	-0.30616	0.578061
TCAF1	-0.1192	0.578048
TPM1	-0.16858	0.577956
ACRBP	0.646647	0.577952
NPY2R	-0.30412	0.577888
SUSD5	-0.45185	0.577887
EIF2AK3-DT	-0.56401	0.577794
SPOCK1	-0.20463	0.577473
LINC01001	0.709518	0.577284
AF129408.1	-0.40119	0.577254
RN7SL589P	2.110807	0.577165
SIPA1L2	0.170804	0.57706
SGMS1-AS1	0.311726	0.576968
EIF3L	0.107572	0.576898
TMC4	-0.79865	0.576601
COMMD7	0.173716	0.576591
CERK	-0.14739	0.576572
MAPK14	-0.13456	0.576494
IL17B	2.46696	0.576469
GGTA1P	-1.05413	0.576402
AC092611.1	-0.64374	0.57639
EMB	0.233241	0.576353
TNR	0.443182	0.576207
NEIL2	-0.19861	0.576178
CILP	0.409423	0.576159
ALDH1L1	-1.23827	0.576114
AC244102.1	2.441775	0.576074
AC126755.1	0.458831	0.576056
FAM111A-DT	0.641558	0.575929
AL606760.1	-0.49951	0.575846
NA	1.129459	0.575817
SH2D6	-0.91551	0.575772
EXOC2	0.153627	0.575654
AC016650.1	1.19527	0.575627
RBP7	2.338659	0.575575
NA	-2.70334	0.575528
TM6SF2	0.571855	0.57549
GPX1	0.113782	0.575317
RNU6-553P	3.436647	0.575296
PHBP3	3.436647	0.575296
AC020928.1	0.337422	0.575234
AC008467.1	0.948342	0.575174
TAGLN2P1	-2.09108	0.575113
AC106037.1	-2.70205	0.574978
AC138028.2	0.446377	0.574871
TMPRSS11CP	-1.47406	0.57487
HS3ST3B1	-1.8917	0.574828
ZNF436-AS1	0.323526	0.574818
RGPD3	2.215781	0.574776
SSBP4	0.153521	0.574707

DNAJC16	-0.17388	0.574514
SCGB1A1	-2.24599	0.574495
RPL5	0.126237	0.574494
CLK4	0.179367	0.574459
LIMD1-AS1	-0.32754	0.574322
MOV10	-0.16432	0.57431
AP000662.1	0.80391	0.574245
ZNF23	0.398996	0.57413
AC021106.1	1.566898	0.574121
HUS1	-0.19202	0.573892
GALNT18	0.344252	0.573845
RHBDF1	-0.33913	0.573813
CASQ1	-1.76075	0.573791
AL137003.2	0.797849	0.573688
CDON	-0.17041	0.57367
DUS4L	0.189177	0.573523
NME1	0.174872	0.573488
SPAG7	0.142307	0.573479
NDUFV2	-0.59719	0.573406
PAQR5	-0.33009	0.573351
AC132825.4	-2.08554	0.573341
PAX8-AS1	-0.98439	0.57328
AL358472.3	-0.68706	0.573252
MAP1LC3A	0.216999	0.573237
ENG	-0.16068	0.57321
CELA2B	-1.41402	0.573063
TNRC6C	-0.15865	0.573018
AC087292.1	-0.23039	0.573003
VASN	0.215199	0.572986
RNA5SP317	1.02832	0.572973
MIR99AHG	0.383973	0.572966
NAA30	-0.17076	0.572771
USP51	0.401851	0.572711
CAPN5	-0.17483	0.572521
ANKRD54	-0.17592	0.572492
TRAF3	-0.17046	0.572369
SF3A2	-0.16506	0.572274
MPPED2	-0.65272	0.57227
ZRANB3	0.209856	0.572245
BOLA2P2	0.995756	0.572236
ADGRG6	-2.80675	0.571942
AC067945.1	-2.80675	0.571942
AMY2B	0.7736	0.571888
TSPAN2	0.278209	0.571787
ATF7IP2	0.45496	0.57175
DUSP6	0.23416	0.571739
AC097468.2	0.879288	0.571533
SLC25A10	-0.27041	0.571458
RPS19	0.124565	0.57134
GGN	0.819789	0.571335

NLRP6	-3.40963	0.57111
AL355607.1	-3.40963	0.57111
AC073316.2	-3.40963	0.57111
NA	-3.40963	0.57111
LINC01544	-3.40963	0.57111
FMO5	-0.29903	0.570962
GGTA2P	-1.35004	0.570927
RAX	1.331651	0.570906
AC006504.5	-0.29402	0.570686
PRSS42P	2.834071	0.570664
SPEN	-0.15526	0.57062
THOC5	0.128401	0.570534
AL023806.1	-1.19054	0.570401
FASTKD2	0.161502	0.570354
ACSS2	0.180661	0.570158
AC022400.6	-0.64905	0.570086
PIMREG	0.188296	0.569997
CEBPG	0.172443	0.569984
AC025162.1	0.204531	0.569983
HMGA2-AS1	1.622917	0.569961
RPS3A	0.11754	0.569955
NXPH2	0.628577	0.569796
CNOT6	-0.14861	0.569644
RPS28P7	0.552813	0.569506
LINC00618	-1.63269	0.569254
TMEM101	0.196261	0.569167
RBM24	0.536842	0.569128
CHST8	-0.16955	0.569087
RN7SL394P	3.441143	0.569
RNASEH2A	0.184443	0.568921
DNAJC15	0.226323	0.568844
FTH1P3	1.272847	0.568801
ATP6V0D1	-0.15582	0.568677
NTNG2	0.611307	0.568664
A3GALT2	-3.40326	0.568553
OVOL3	-1.6733	0.56853
AC090220.1	-2.80669	0.568466
AC099518.1	2.168412	0.568398
SPATC1L	-0.26736	0.568391
CDK20	-0.24711	0.568277
FRMD8	-0.21407	0.568239
CSAD	0.202462	0.568229
AC233992.1	0.332289	0.568083
CLIP1	0.144202	0.568077
SEPT4-AS1	0.865991	0.568014
KRTAP5-AS1	0.513645	0.567889
CD82	0.233874	0.567863
AC124319.1	-0.13557	0.567831
ZFC3H1	0.123247	0.567723
TAGLN2	-0.13716	0.567668

RPL12	0.122966	0.567552
BIK	2.587624	0.567511
UCHL3	0.466311	0.567497
AC090004.1	-0.75913	0.567471
AC103770.1	2.817386	0.567433
AP000350.5	-1.25881	0.567397
DOCK10	-0.15222	0.567389
AC096915.1	2.817392	0.567225
SLC39A5	-1.08534	0.567203
HEXB	-0.15306	0.567178
TMEM116	0.281656	0.567017
RPL35P5	2.403707	0.566919
LIPE	-0.29922	0.56674
AC005775.1	0.174166	0.566571
NA	-2.82245	0.566549
ANKRD17	-0.10995	0.566532
NRG2	0.271184	0.566465
ASPDH	0.334843	0.566397
LINC00467	0.311605	0.56604
NA	-2.66028	0.565912
RPS19BP1	-0.14148	0.565873
EGFR-AS1	-0.38576	0.565859
LEAP2	-0.37068	0.565758
ZBTB43	0.184168	0.565756
AC090907.2	-0.93311	0.565749
XRRA1	-0.18038	0.565559
NAT9	0.127517	0.565385
ANKRD13A	0.129526	0.565148
ACOT1	-0.35644	0.565147
SELENOT	0.147428	0.565124
FAM167B	1.325517	0.565059
NA	1.06841	0.56493
AL592490.1	-0.72906	0.564769
GGPS1	-0.12891	0.564741
ZNF239	-0.18629	0.564688
ADAM3B	-2.31695	0.564623
ILF3-DT	0.188973	0.564523
BTD	0.243392	0.564356
AC009220.2	-1.50112	0.56422
TAS2R4	-0.79731	0.563962
ADAM19	-0.20539	0.563444
FAM122B	-0.15031	0.56344
MYH8	-0.66718	0.563402
SLC25A23	0.150128	0.563328
USP2-AS1	-0.75963	0.563232
TMEM211	-3.57877	0.563229
FDPSP7	-3.57877	0.563229
AL160262.1	-3.57877	0.563229
NA	-2.98021	0.563208
AP000880.1	1.161298	0.563124

TMEM94	-0.16938	0.563064
FOSL2	0.236788	0.563063
CBX2	0.136365	0.562928
LINC00599	0.227536	0.562887
EFHD2	0.191968	0.562839
REEP1	0.398583	0.5628
SERP1	0.113587	0.562587
C6orf132	-2.97028	0.562278
RPL7P60	-2.97028	0.562278
PAM	-0.13682	0.56226
AC090971.2	0.388587	0.562246
BRCC3	0.205859	0.562125
SOX2	-0.40216	0.562116
NA	-2.23686	0.562052
AL031118.1	-2.82239	0.561892
KCNJ14	-0.43109	0.561833
ROBO4	-1.65342	0.561787
YBX2	-0.5708	0.561778
RNLS	-1.8805	0.561532
AC104521.1	-1.2094	0.561466
TOR4A	-0.56396	0.561413
MAN1B1-DT	-0.93407	0.5614
UBXN6	0.124013	0.561378
KLHL24	0.123739	0.561264
ATP6V1H	-0.16516	0.561245
AC009139.2	-0.36368	0.560934
LRRC37A4P	-0.22843	0.560881
ZNF154	0.812472	0.560753
BCL11A	0.301909	0.560711
AL355816.1	-1.28744	0.560659
ARL4D	-0.37033	0.560628
EFTUD2	-0.12094	0.560613
AP000866.4	1.108065	0.560555
ERC1	-0.1418	0.560521
NA	-0.32032	0.560385
USP38	-0.15312	0.560314
PP2D1	0.25209	0.560285
SORBS2	0.455806	0.560199
RTN1	0.117666	0.560174
AC011442.1	1.268605	0.560154
TET2	0.174975	0.559832
ZNF204P	0.607669	0.559831
AC087623.1	0.384638	0.559821
RPL3P7	2.193358	0.55959
HADHB	0.117675	0.559421
AC092658.1	-1.96033	0.559311
AC011468.1	0.738097	0.55927
ZSCAN23	0.281274	0.559207
PIGU	-0.17745	0.559182
SOX21	-3.56866	0.559096

LINC02656	-3.56866	0.559096
SMCO1	-3.56866	0.559096
RPSAP36	-3.56866	0.559096
RPL3P3	-3.56866	0.559096
ENPP7P12	-3.56866	0.559096
CYB5R1	-0.1874	0.559033
SEMA6A	-0.27798	0.558962
PXMP4	0.297309	0.558922
CLTC	-0.10227	0.558889
AL355816.2	0.280074	0.558815
TADA2B	-0.18358	0.558474
TRGV7	-2.50522	0.558333
NA	0.188116	0.558303
LDLRAD4	0.152734	0.558221
ZNF563	0.647184	0.558079
OSMR	-0.25738	0.557893
WIPF3	0.535222	0.557644
DSN1	0.194245	0.557536
NCOR1	-0.11236	0.557493
POLR2I	0.162607	0.557482
GCNT2P1	-1.93677	0.557466
CMAS	0.148012	0.557459
AC005261.4	-0.46985	0.557214
CD63	0.120705	0.557185
ILVBL	0.15211	0.557087
LINC02198	2.801821	0.556981
NA	2.801821	0.556981
AL031282.2	-0.46251	0.556888
FNBP4	-0.12861	0.556488
MID2	-1.99807	0.556425
LINC01389	1.492463	0.556359
MAFB	-0.18163	0.556356
AC105020.2	0.250148	0.556356
DKFZP434K028	-0.57361	0.556325
AL359715.1	-0.63534	0.556064
GPR150	1.645618	0.555955
HCG4B	3.387581	0.555776
LRP1B	0.381595	0.555755
SWSAP1	0.39499	0.555722
ATP6AP1L	-0.35486	0.555668
ABHD14A-ACY1	2.416356	0.555611
TLE1P1	1.293631	0.555584
LARP1P1	-2.63639	0.555525
AC040169.1	-0.87886	0.555493
CDK14	-0.15919	0.555464
PPP1R10	-0.15639	0.555427
GSTT2	0.282234	0.555355
TCF7L2	0.274184	0.555205
KCNJ13	-1.08979	0.555131
NAA80	-0.36784	0.554855

ETFA	0.127505	0.554691
AC110619.1	1.771224	0.554642
CLCN3P1	0.625133	0.554614
GPC1	-0.18112	0.554509
SLC35C2	0.144488	0.554454
AC009088.1	-2.335	0.554443
GAPDHP1	0.659891	0.554404
DDX19B	0.217922	0.554377
AL110504.1	-0.2623	0.554339
C12orf4	0.162816	0.554329
TRNT1	0.162779	0.554266
AC106795.1	-0.20723	0.554147
SFSWAP	0.143625	0.554098
RAP2A	0.160437	0.553988
TLDC2	-0.46874	0.553937
ADAM1A	-0.27082	0.55392
AC011603.3	0.820836	0.553919
ZNF711	0.129459	0.553808
AL357093.2	2.215137	0.553795
SNAP29	0.15786	0.553758
TLX2	0.148804	0.553692
CLDN14	-2.81006	0.553586
MRPS15	0.148789	0.553569
MARCH11	0.117185	0.553515
KPNA5	0.19494	0.553501
C19orf57	-0.2497	0.553364
IMPAD1	-0.12581	0.553307
FBXO21	0.156473	0.553113
COL21A1	-2.21109	0.553047
AP001992.1	1.279309	0.552897
RBM8B	-0.87876	0.552885
KANK1	-1.4779	0.552712
BHLHE22	-3.38367	0.552664
CROCCP5	-2.41196	0.552482
OR1F1	-2.49106	0.552346
NFX1	0.140765	0.552123
AL583722.2	-0.13714	0.552114
C9orf116	-0.33806	0.552085
RIIAD1	3.229257	0.552081
AC096669.1	3.229257	0.552081
AC002400.1	3.229257	0.552081
TMEM106C	0.12458	0.552039
YBX1P1	0.470334	0.551878
NOP58	0.129675	0.551673
NA	-2.639	0.551654
DUSP18	0.36335	0.551627
AC130324.1	2.788365	0.5516
MINPP1	-0.13992	0.551573
AC018766.1	0.239394	0.551397
FAM161A	0.202528	0.551387

DOK1	-0.18884	0.55114
ENTPD2	1.248386	0.551129
F2RL1	-0.37427	0.551038
LMO7-AS1	0.389738	0.550927
PLGRKT	0.223536	0.550615
OXCT2P1	-1.62614	0.550598
UBE2SP2	-2.63633	0.550544
MRTFA-AS1	-2.63633	0.550544
SNX25P1	1.961879	0.550438
RAMACL	-2.26384	0.550421
PCDH8	-0.76865	0.550397
CCDC71L	0.158089	0.550312
AL133297.2	2.59385	0.550293
SPINK9	-1.63517	0.550291
AJUBA	0.213225	0.550264
LINC01315	0.622753	0.55017
UBXN7	-0.12145	0.550038
NA	1.461735	0.550016
AZI2	0.128648	0.549943
AL138799.1	1.904324	0.549525
AC010327.5	-0.73708	0.549394
RPL7P9	0.414756	0.549379
ISYNA1	0.137379	0.549352
RRM2B	-0.18098	0.549342
AC006042.1	0.212541	0.549233
NPAS2	0.208043	0.549141
VPS25	0.132213	0.549101
C1S	0.475737	0.549066
RN7SL362P	-2.62062	0.549015
DLG5-AS1	-0.7581	0.548881
XPO6	-0.13358	0.548876
IPO5P1	0.273154	0.548796
LGALSL-DT	-3.3524	0.548756
NA	-0.59636	0.548742
GIGYF2	-0.12126	0.548682
AC040162.3	-1.54486	0.548646
C9orf50	-1.07848	0.548575
DNAJC25	-0.23152	0.548371
ZNF561	0.134675	0.548346
AC080075.1	0.420564	0.548331
AL078587.1	-2.3753	0.548321
DAG1	-0.15403	0.548225
TARSL2	0.198463	0.54822
RNF139-AS1	-0.37193	0.548102
CDCA2	-0.21285	0.548076
BEX4	0.139283	0.548032
SDE2	0.125773	0.547997
HBP1	0.13358	0.547943
FAM83G	0.2748	0.547933
LCTL	-0.24907	0.547816

ANKRD30A	2.288324	0.547769
FCRLB	-1.00702	0.547696
MARCH10	-3.35403	0.547596
AC009477.1	-3.35403	0.547596
AL590787.1	-3.35403	0.547596
AL031432.2	-1.22872	0.547552
NA	-0.42804	0.547481
ITPR1	-0.13897	0.54735
ACBD4	0.293772	0.547308
AL450405.1	0.38422	0.547288
AC007881.3	-2.63628	0.547054
CGRRF1	0.221984	0.547015
HKDC1	2.543705	0.546995
PDLIM5	0.16271	0.546912
RAB30	-0.17752	0.546861
HLA-L	0.238843	0.546782
TMC3-AS1	-0.83796	0.54674
AC034231.1	0.928398	0.546719
IQSEC1	-0.20865	0.546666
KATNAL2	0.276269	0.546407
ARHGEF25	0.147699	0.546277
AC022167.3	-3.35072	0.54623
NUFIP2	-0.13507	0.546142
ZFP1	0.199467	0.546041
HMMR-AS1	0.400134	0.546015
MFSD3	0.179487	0.545861
LINC00337	-1.54886	0.545821
AC074212.1	-0.21523	0.545728
SCAND2P	-0.19677	0.545713
NACC2	-0.22139	0.545648
CYP4F3	2.018201	0.54564
SRRM2	-0.13527	0.545623
AC079203.2	-0.74615	0.545558
PRLHR	-0.51029	0.545531
SERGEF	-0.3644	0.545334
NA	-2.63493	0.54531
RB1CC1	0.135148	0.545249
TDRD7	-0.23803	0.545105
EIF4EP2	-0.75833	0.545092
BMS1P10	0.524929	0.545076
ZNF320	0.158217	0.545043
LINC01397	2.932923	0.544975
AC092045.1	-2.17045	0.544948
HINT2	0.178985	0.544944
NUPL2	0.141814	0.544906
PDIA6	0.099863	0.54486
AP000808.2	1.95404	0.544465
TMEM134	0.178238	0.544418
CACNA1A	-1.11072	0.544418
AL590705.3	2.235857	0.544409

PRKACA	-0.15596	0.544324
NRP2	-0.1801	0.544322
ARF4P2	2.284906	0.54427
ATAD2B	-0.14415	0.544251
IGFBP3	0.548873	0.544249
MYRF	0.351102	0.54415
ICAM1	0.198419	0.544147
AC067751.1	0.273328	0.543991
MGAT5	-0.14048	0.543967
AL391069.1	0.249466	0.543766
HIVEP1	0.179492	0.543695
SNX29	-0.18734	0.543676
ERICH5	-2.27619	0.54365
AC114980.1	2.296799	0.543643
AC112493.1	2.649859	0.543623
VIPAS39	0.158416	0.543585
NA	0.447832	0.543563
AADAC	-1.01277	0.543522
ERLIN2	-0.16487	0.543506
AP001178.1	1.922149	0.543268
CTSC	-0.13303	0.543265
TACO1	0.156372	0.5432
AL022341.1	-0.73836	0.542848
AC005165.1	-1.20074	0.542783
CILP2	0.465103	0.542642
ACTBP7	3.359936	0.542619
AL445985.1	1.003028	0.542457
LRRC9	-1.34855	0.542421
BBS2	-0.13782	0.542401
NMI	0.443931	0.542118
FKBP7	-0.16233	0.542091
AC022973.4	0.496887	0.542054
SNCA	0.236313	0.542043
CSRP3-AS1	-0.87691	0.542029
RACK1	0.117362	0.541902
KLHDC2	0.119408	0.541755
AC022146.2	3.217059	0.541747
AC021683.1	2.780665	0.541694
ADAMTS5	0.422918	0.541507
PYCARD	3.37025	0.541478
PLOD2	3.37025	0.541478
HNRNPA1P40	3.37025	0.541478
RPL19P20	3.37025	0.541478
LINC01204	3.37025	0.541478
LSAMP-AS1	3.37025	0.541478
MIR548AT	3.37025	0.541478
AC006270.3	3.37025	0.541478
EXOC3L4	0.564507	0.541456
AC010615.2	-0.81582	0.541266
RF00100	0.521282	0.541256

ABITRAM	0.185778	0.541235
AL355490.1	-0.5659	0.541195
AC090543.1	-3.35558	0.541149
MTND3P25	-3.35558	0.541149
AL356215.1	-3.35558	0.541149
ROBO1	-0.12325	0.541143
TRPM8	2.925629	0.541093
AC007681.1	2.925629	0.541093
STOML1	0.223131	0.541028
HDAC1	-0.12189	0.540856
RDH5	0.727345	0.540815
FAM149A	-0.50573	0.540602
RCAN3	-0.28743	0.540495
NDUFB9	0.133017	0.540361
AC021016.3	1.176926	0.540355
AC245052.4	-0.17445	0.540299
PDGFRL	0.332436	0.540296
RAMP2	0.321526	0.540284
SRRM2-AS1	0.619825	0.540218
POLR2L	-0.14233	0.540179
CCDC13	-1.52256	0.540118
PHETA2	-0.21485	0.539862
AJ003147.2	0.627649	0.539826
SOHLH2	0.623168	0.539529
DET1	-0.288	0.539476
CFAP300	2.920567	0.539453
PTPN2P2	2.920567	0.539453
AC011503.3	-2.26592	0.539417
AC022211.2	0.22985	0.539408
SHISAL1	2.087569	0.539308
GAPDHS	0.265403	0.539178
SLC5A10	0.578278	0.539133
B3GALT4	-0.78035	0.539118
AC092171.5	0.839027	0.538979
COLEC12	-0.4856	0.538876
AC007292.2	-0.31163	0.538845
NDUFB3	0.204824	0.538591
TARBP1	-0.1423	0.538581
STMN4	0.305355	0.538572
RPS20	0.116878	0.538492
SKP1	0.122166	0.538476
AP000238.1	-1.72767	0.538428
PLCL1	0.35861	0.538379
AARSD1	0.506824	0.538295
AC012313.2	0.479051	0.53819
UBE2B	-0.12681	0.538177
PAQR4	-0.18262	0.538162
PDZD9	2.780476	0.538152
GMCL1	-0.12693	0.53809
ZNF264	0.247646	0.537933

AP006333.1	-0.89856	0.537933
ANO9	0.23556	0.537908
CFAP99	2.925467	0.537749
SLC19A1	-0.14145	0.537684
AC025165.5	-0.88004	0.537476
GJC2	0.346264	0.537391
AC004490.1	-0.3591	0.537365
GATA3-AS1	0.145868	0.537221
SLC22A16	-3.51088	0.537186
APOA1	-3.51088	0.537186
TMPRSS13	-3.51088	0.537186
CNTN5	-3.51088	0.537186
CDA	-3.51088	0.537186
NA	-3.51088	0.537186
MIR125B1	-3.51088	0.537186
NA	-3.51088	0.537186
RNA5SP294	-3.51088	0.537186
SNAP23P1	-3.51088	0.537186
AL365204.1	-3.51088	0.537186
NA	-3.51088	0.537186
MT1XP1	-3.51088	0.537186
AL162727.2	-3.51088	0.537186
AC007879.4	-3.51088	0.537186
AC012625.1	-3.51088	0.537186
AC079054.1	-3.51088	0.537186
RNU5A-3P	-3.51088	0.537186
LINC00678	-3.51088	0.537186
ACTN1-AS1	-3.51088	0.537186
AP005131.4	-3.51088	0.537186
AC005332.2	-3.51088	0.537186
AL139041.1	-3.51088	0.537186
RPS13	0.121497	0.53714
PSMD7	0.116984	0.537136
AC004158.1	0.58941	0.537007
GPAT2	0.453061	0.536894
MIR497HG	0.190424	0.536842
MZT2A	0.162716	0.536797
AL359711.2	1.275882	0.536729
AL606834.1	1.056487	0.536574
SPOPL	0.164066	0.53651
FP325335.1	2.920549	0.536377
PDIA3	0.11318	0.536242
AP002892.2	2.920548	0.536188
NA	2.920548	0.536188
FAM106A	2.920548	0.536188
AC013452.2	1.680828	0.536095
BMPR2	-0.12477	0.536088
VRK2	-0.23805	0.53608
ZMAT4	-0.3629	0.536034
RET	-0.17749	0.535933

MSANTD2	-0.19663	0.535893
RNU6-885P	1.386562	0.53588
MMAA	0.378397	0.535845
LAX1	-3.18437	0.535664
TICAM2	-3.18437	0.535664
FKBP2	0.147769	0.535543
AL139220.2	-1.49619	0.53553
RDH12	1.435839	0.535517
NA	-1.51396	0.535407
AC007637.1	0.770938	0.535254
AC073641.1	-0.54516	0.535228
WNK1	-0.14136	0.535084
AL136454.1	0.629203	0.534795
COX5B	0.117403	0.534787
HMGN5	0.545654	0.534593
TCEA1P4	1.136151	0.534548
COPZ2	-0.33758	0.534538
HAUS6P1	1.955108	0.534416
RHNO1	-0.15156	0.534386
ZFHX4-AS1	0.785869	0.534243
HILPDA	0.174093	0.534183
AL513218.1	0.370841	0.5341
NFAM1	2.428001	0.534076
MSRB2	0.162972	0.533851
ISG20	0.622236	0.533848
AP005205.2	1.259186	0.533755
NA	0.598783	0.533596
DBI	0.13291	0.533457
ARL6IP6	0.16965	0.533444
AL356317.1	2.758712	0.533415
SNORA58	2.758712	0.533415
HOMER3-AS1	-0.98471	0.533399
LINC01833	0.166297	0.533339
NECAB1	0.2154	0.533286
PDK3	-0.33548	0.533195
AL591721.1	0.333054	0.533152
UPK1A	3.522002	0.533035
AP001793.1	0.276479	0.532956
AL049646.2	-0.45856	0.53292
ZFAND2B	-0.17675	0.532856
MIR367	2.733076	0.532836
NMNAT3	-2.35226	0.532768
AC124068.2	1.281259	0.532659
SP4	-0.18806	0.532557
DIRAS1	-0.14544	0.532546
PRRX1	-0.77392	0.532529
LFNG	-0.20787	0.532519
MFAP4	-0.1386	0.532411
PCED1A	0.150334	0.532283
DZIP1	-0.11802	0.532256

AZIN1	-0.12017	0.5322
ARMC12	0.568323	0.532101
RNA5SP283	-0.34991	0.531937
TSSK1B	2.052685	0.53191
SAYSD1	-0.21962	0.531747
RPS4XP6	1.286379	0.531675
RILP	-0.60328	0.531633
MAGOH2P	-0.83169	0.531376
CACNA1G	0.516129	0.531356
CDKAL1	-0.14746	0.53132
GSN-AS1	-0.43157	0.531303
PGAM2	0.243847	0.531187
NEK1	0.15781	0.53116
AC000120.1	0.748658	0.53116
AC104590.1	1.848901	0.531048
MEOX1	2.020864	0.531024
AC110792.3	1.981714	0.531002
FOCAD-AS1	2.175605	0.53098
C1GALT1C1L	0.399777	0.530807
RAPGEF3	-0.2921	0.530664
ANKAR	0.366414	0.530628
AC087749.2	1.508418	0.530604
AC007683.1	-1.83759	0.530477
UNC5D	0.581349	0.530472
MIR3124	-1.79427	0.530364
GBX1	3.34222	0.530345
FER1L6	3.34222	0.530345
AC093422.2	3.34222	0.530345
CEACAM22P	3.34222	0.530345
AL135786.1	3.34222	0.530345
AC096888.1	3.34222	0.530345
GDNF-AS1	3.34222	0.530345
NA	3.34222	0.530345
AC011933.3	3.34222	0.530345
TTC14	0.146965	0.530246
CELSR3	-0.1483	0.530216
DUSP23	0.167672	0.530211
AC012645.2	-2.11021	0.530197
HLTF-AS1	0.521711	0.53015
ABHD17B	-0.17699	0.530105
CASP12	-2.38899	0.530095
PCDHA5	-2.15092	0.530065
LRP5	-0.15999	0.530016
AL033380.1	2.758732	0.529995
PHF24	1.411235	0.529992
PRKDC	-0.10276	0.529876
DDN-AS1	0.197298	0.529834
AL353705.4	-1.7373	0.529767
AC092881.1	0.119735	0.529723
CCDC82	0.181628	0.529675

BORCS7	-0.19887	0.529646
TMEM107	-0.3809	0.529603
ZSCAN29	-0.13769	0.529574
MYL6B	0.142967	0.529504
NA	1.436739	0.529482
DNAJC11	-0.14075	0.529482
FDPS	0.116804	0.529461
NPB	0.307225	0.529436
NUDT17	0.226462	0.529385
ZNF48	0.152413	0.529254
HIST2H2BC	-0.16528	0.52919
STOML2	0.123732	0.529171
MFSD13A	-0.21854	0.528948
NA	1.624731	0.528876
SEC23A	-0.14049	0.528855
AC016526.2	-1.9055	0.528848
AP003108.2	0.265394	0.528807
FAM102A	-0.149	0.528798
TTBK2	-0.15967	0.528715
AC010285.1	2.134134	0.528664
SCART1	0.542919	0.528635
BRK1	0.119881	0.528599
PTPRN2	0.158378	0.528557
AC005868.1	-2.74611	0.528504
AC090772.2	-2.74611	0.528504
AC007192.2	0.570154	0.528386
GPRIN1	-0.123	0.528286
RPS26P15	-3.15461	0.528273
TRAM2	-0.13403	0.528137
AC073869.3	-0.74169	0.528072
SNAPIN	0.139905	0.528028
RBM47	0.708589	0.527867
TENM3	-0.13915	0.527782
ZKSCAN1	-0.10627	0.527737
CLEC4A	1.910509	0.527603
UNC13D	-0.24398	0.527599
AP002906.1	-3.15274	0.527514
SLC2A1-AS1	-0.91415	0.527481
NDUFC2	0.193604	0.527453
FBXO32	0.311322	0.527379
NA	0.581542	0.527309
ABCC10	0.162146	0.52724
NA	-0.28951	0.527224
ATRAID	0.126729	0.527186
APOL2	-0.29265	0.527166
HLA-E	0.183189	0.52699
VIPR2	0.375952	0.526922
AC012313.7	-0.6215	0.526876
AC092171.2	-0.16839	0.526867
ATP2B1	0.176823	0.526851

SLC25A6	0.121299	0.526845
AP000811.1	1.226215	0.526836
U62317.2	0.195093	0.526754
LINC00847	0.18559	0.526597
AC104564.1	-1.61765	0.526552
ALG6	-0.23694	0.526535
PEAR1	-0.26918	0.526512
TMED2	0.100552	0.52612
FOXO6	0.314709	0.526082
LYSMD3	0.17907	0.526059
HMGN1P8	1.559173	0.525938
MAPK4	0.439109	0.525872
C1RL	0.283789	0.525817
TEPP	-1.60795	0.525776
KCNG3	-0.49355	0.525772
USP22	-0.12292	0.525752
SCARNA9	0.906536	0.525691
RMI1	-0.21915	0.525606
C16orf92	0.306524	0.525569
OLA1P1	1.548239	0.525498
ZNF524	0.263668	0.525494
LINC00639	-0.69151	0.525447
PMM1	0.268377	0.525421
AC009264.1	-0.9716	0.525265
WTAP	0.111489	0.525256
UTP3	-0.15268	0.525224
ABCB4	0.508203	0.525221
AL592528.1	-1.72791	0.525195
EIPR1-IT1	-0.8132	0.525132
CDRT15P1	0.666115	0.525095
NA	-2.23283	0.525064
CYP2A6	-1.01146	0.52506
CEMIP2	-1.0626	0.524976
AC139491.2	-0.72273	0.524843
FAM86B3P	-0.37034	0.524828
LGR6	-2.68428	0.524788
KLHL9	-0.14124	0.524654
TMEM102	-0.26304	0.524565
PAXIP1	-0.1488	0.524556
AC018645.2	0.540595	0.524543
RPL29P24	1.457923	0.524486
AC009403.1	-0.40374	0.524473
NLGN3	-0.25873	0.524448
RPL13AP20	1.161902	0.524284
PRAM1	3.331522	0.524273
AC079601.2	3.331522	0.524273
ZNF646	-0.15912	0.52427
GLIS2-AS1	1.606787	0.524114
CHCHD1	0.150767	0.524097
WDR19	0.126156	0.524053

AC140725.1	-1.89246	0.52402
ZC2HC1A	0.184459	0.523973
DNAH5	-1.00976	0.523948
MRPS24	-1.00054	0.523931
TMEM220	-0.23672	0.523895
AL137013.1	-1.38407	0.523805
STAM2	-0.16721	0.523771
C14orf39	1.061717	0.523704
ZNF594	-0.16677	0.523673
TIMM29	0.222534	0.523599
LINC02475	2.249934	0.52357
RC3H1-IT1	-1.56105	0.523533
FBXL12	0.174243	0.523389
TEX30	0.195852	0.523306
PRKCD	0.328749	0.523221
AC112907.3	0.226998	0.523216
ANKRD52	-0.14872	0.523208
PSMB5	0.107244	0.523177
DHRS1	-0.23875	0.523166
KIAA1257	2.392512	0.523045
AP000787.1	0.699625	0.522996
FARP1-AS1	-0.48974	0.52288
AC079779.2	2.162465	0.522849
GTF2H3	-0.12663	0.522811
SNRPE	0.152829	0.522804
ASB16	0.324893	0.522783
ASS1P2	-1.46725	0.522761
AL161787.1	1.848706	0.52273
MSN	-0.16803	0.522679
MIR659	1.826937	0.522608
AC092718.5	1.156353	0.5226
PPFIA3	0.160302	0.522487
HOXD3	-0.1717	0.522413
BCDIN3D	0.217366	0.522377
HNRNPA1P9	-1.14262	0.522208
AC026150.3	-1.35968	0.522099
YEATS2	-0.11083	0.522041
NA	-1.37808	0.521991
HAX1	0.131956	0.521961
CNBP	0.113113	0.521924
AC117498.3	1.517554	0.521594
TSC1	-0.12891	0.521531
AL589880.1	0.506992	0.521292
AC005776.1	1.952623	0.521261
AL592114.1	2.58343	0.52126
SPICE1	-0.19284	0.521205
MRPL23-AS1	0.968165	0.521145
NA	-0.35683	0.520841
NT5C1A	0.370195	0.520823
MRPL17	0.150417	0.520797

HMGCR	0.164746	0.520792
ZFYVE26	0.176573	0.520644
GNG7	-0.25172	0.520634
ARHGDIA	-0.11758	0.520607
KRR1P1	-1.40203	0.5206
C11orf94	1.078037	0.52055
PNP	0.148463	0.520548
NLK	-0.19268	0.520536
BEX1	0.122467	0.520502
SLC37A3	0.119746	0.520498
BX284668.6	1.287391	0.520394
LGALS9	1.012014	0.520357
EMC9	0.210163	0.520204
CITED2	-0.28186	0.520191
USP16	0.175781	0.520172
ARGLU1	0.127346	0.520026
ANXA1	-0.67892	0.520004
FBXO16	0.365707	0.520004
NFKBID	-0.41855	0.519706
ZC3H11A	0.104159	0.519693
NA	-0.47138	0.519686
AC005746.1	0.578443	0.519497
SCML4	-1.43147	0.519413
NA	-0.68077	0.519318
COQ2	0.244246	0.519182
MCF2L2	-0.21258	0.519172
HMGN2P3	-0.35264	0.51914
LINC01881	-0.5232	0.519085
AC016596.1	1.802154	0.518976
AC022784.1	2.140561	0.518975
MST1R	-0.64587	0.518846
TRIM38	-0.85649	0.518727
AC093690.1	-0.78094	0.518593
AK5	0.28413	0.51847
AC116158.2	2.044465	0.518233
AP001029.1	-1.29721	0.51823
CKMT2	0.512891	0.518071
CENPE	-0.19913	0.518038
AC034102.6	0.16141	0.517916
MFSD1	-0.12334	0.517599
MFSD11	0.100656	0.517537
KIAA1211	-0.13466	0.517482
NA	2.221416	0.517419
MTHFR	0.192422	0.517404
TMEM150A	0.277494	0.517294
BCAR1	-0.1421	0.517204
CYP1B1	-0.39745	0.517135
CUTALP	-0.16106	0.517018
AL604028.2	1.457339	0.516844
GOLGA8UP	3.155235	0.516829

MIR298	3.155235	0.516829
NCOA7-AS1	3.155235	0.516829
YJU2	0.197502	0.516817
AC004039.1	-1.94982	0.516664
AC011379.2	-1.21236	0.516543
WASF3	-0.16035	0.516472
RNU4ATAC18P	1.242863	0.51647
ZNF557	0.22519	0.516258
RFPL1	-0.41843	0.516217
MICAL1	-0.13741	0.516132
ANK1	-0.31645	0.516093
FAM220A	0.16483	0.515879
TTC27	-0.15098	0.515804
POLH	0.196649	0.515784
SLC12A3	-1.28979	0.515694
SMIM18	-0.46121	0.51553
NUMB	0.120809	0.515465
CSNK1A1	0.117974	0.515412
AC110813.1	1.295774	0.515391
STAG3L3	0.227866	0.51521
RN7SL233P	3.289492	0.515129
AC005586.2	1.720521	0.515116
MAPK1IP1L	-0.11508	0.515036
AP001160.1	0.15971	0.514902
AC004865.2	1.163287	0.514863
RPL5P23	1.31612	0.514833
RNU6-705P	1.193438	0.514797
TTLL11	0.262562	0.514709
AC010186.3	0.355255	0.514686
RAD21L1	1.260598	0.51461
MIR155HG	-2.23286	0.514558
PPFIBP2	-0.2406	0.514545
RBMS3	0.107536	0.514514
KIF1BP	-0.15166	0.514494
TMF1	-0.15224	0.514411
NA	-0.1913	0.514326
SLC12A1	1.67189	0.514314
ZFYVE19	-0.16077	0.514313
PCDHA7	1.370197	0.514278
POTEG	-2.19921	0.513951
RPS20P4	1.984691	0.513894
ATOX1	0.164835	0.513889
SLC6A8	-0.1347	0.51383
RF00019	-1.96202	0.51372
ZFAND3	0.129056	0.513601
MECP2	-0.1295	0.513572
ANAPC11	0.117485	0.513503
CLCNKA	-0.48555	0.51349
NA	0.911299	0.513423
TNIP3	-0.89489	0.513393

NAV2-AS2	-0.27172	0.513236
LINC01194	-0.58695	0.513164
KIF17	0.625242	0.513014
TBX21	3.466981	0.512956
ENPP3	3.466981	0.512956
SLC6A19	3.466981	0.512956
LEP	3.466981	0.512956
PAWR	3.466981	0.512956
DRD1	3.466981	0.512956
C2orf78	3.466981	0.512956
CR1L	3.466981	0.512956
AKR1B10	3.466981	0.512956
RF00092	3.466981	0.512956
EEF1DP4	3.466981	0.512956
MDP1	3.466981	0.512956
RPL17P26	3.466981	0.512956
NA	3.466981	0.512956
RPS27P16	3.466981	0.512956
AC114760.1	3.466981	0.512956
AC009229.2	3.466981	0.512956
AL008627.1	3.466981	0.512956
DYNLL1P7	3.466981	0.512956
RPL23AP35	3.466981	0.512956
RPS26P31	3.466981	0.512956
AC000099.1	3.466981	0.512956
AL441964.1	3.466981	0.512956
RNU7-140P	3.466981	0.512956
AL442663.1	3.466981	0.512956
AC010469.2	3.466981	0.512956
RPL12P32	3.466981	0.512956
AC007537.1	3.466981	0.512956
AC093627.6	3.466981	0.512956
LINC02234	3.466981	0.512956
AC138832.1	3.466981	0.512956
AC010182.1	3.466981	0.512956
AC093283.1	3.466981	0.512956
SETP3	3.466981	0.512956
AC073413.1	3.466981	0.512956
LAPTM4BP2	3.466981	0.512956
TMTC2	0.173703	0.512888
RNU6-195P	2.217113	0.512877
AC015688.6	0.352605	0.512852
SRRT	-0.10843	0.512839
MACF1	-0.11076	0.512815
AC004076.1	2.680079	0.512273
GBP4	0.674034	0.512233
LINC02145	1.220218	0.512144
AOX1	-0.40125	0.511892
XPOT	0.101517	0.511725
RABL3	0.186327	0.511662

NUMBL	0.186949	0.511627
RN7SL521P	0.881075	0.511609
NA	0.194601	0.511507
AC135983.2	0.327451	0.511499
PSME1	0.121345	0.511488
TMEM88	-0.78358	0.511442
RPL17P10	-3.12428	0.511391
IBA57-DT	-1.19412	0.51129
HPF1	0.15688	0.511218
MED22	-0.12641	0.51118
AP003072.2	-1.14881	0.511068
ANGPTL6	-0.36431	0.510996
CD79A	2.136695	0.510864
NAPB	-0.14627	0.510738
TMED1	-0.14673	0.510543
EDEM2	0.201275	0.510474
AC090114.1	-1.75516	0.510233
MORC2	-0.13975	0.51013
MAGEC2	-0.49397	0.510087
AP001160.3	-0.23385	0.510086
SDHC	0.108821	0.510035
COL6A5	2.684276	0.509933
NA	2.684276	0.509933
NA	2.684276	0.509933
PIH1D1	0.105692	0.509933
EGR1	0.427276	0.509932
DNM1P46	-1.27042	0.509899
NA	-3.12047	0.509888
KCNV2	-0.7504	0.509849
U91328.3	-1.65628	0.509712
SELENON	-0.1599	0.509673
PTGES3P3	2.706366	0.509665
AC079336.4	2.706366	0.509665
AFTPH	0.141348	0.509644
MRVI1	-0.19429	0.509464
ZNF876P	0.473803	0.509448
EFL1P1	0.78424	0.509407
NA	-2.23342	0.509291
AC105105.1	-0.49477	0.509241
B3GLCT	-0.17085	0.509185
HERC2	-0.12698	0.509171
RN7SL23P	1.090909	0.509154
COL3A1	-0.15821	0.509072
AC020661.3	2.151655	0.50907
MST1	0.215735	0.509032
NA	-0.85485	0.50884
SNORD51	-1.54281	0.508831
ALKBH2	0.173859	0.508803
HTATSF1	-0.11679	0.508583
RGPD6	3.27219	0.508572

MMRN2	-2.66289	0.508514
SORBS1	-0.29023	0.508435
AL157713.1	2.234206	0.508345
AC005520.2	-1.48368	0.508344
TAF7	-0.11632	0.508194
AC008752.3	-0.97786	0.508111
AC005520.3	2.661946	0.508096
ZNF599	0.215636	0.508066
RER1	-0.13579	0.508027
NA	1.692948	0.507972
AC022966.2	-0.15995	0.50792
IFFO1	-0.15153	0.507879
AC011451.2	-1.94468	0.507762
RF00426	2.525885	0.507609
AL138976.2	1.039282	0.5075
H19	0.660593	0.507406
PWWP2A	-0.14899	0.507391
CLIC2	-0.61113	0.507289
SASH1	-1.3737	0.507278
TSNAXIP1	-0.20026	0.507236
PRDM6	-0.6739	0.507056
GCDH	0.184113	0.506926
MRM2	-0.11506	0.50686
MED4	0.151862	0.506624
GPS2	-0.10845	0.506607
TRO	0.114832	0.506472
NA	-1.95918	0.506324
CACFD1	-0.19276	0.506298
GALNT6	0.28076	0.506138
HELQ	-0.21092	0.50611
ACAT1	-0.17674	0.506027
AC073573.1	0.346979	0.505792
PCGF3	-0.10519	0.505634
SAR1A	0.117734	0.505578
RBBP4P2	2.08833	0.505564
SHISA5	-0.16531	0.505477
SLC5A9	-0.66843	0.505392
NR2F1-AS1	-0.24873	0.505375
AL390835.1	1.21022	0.505315
CCNY	0.110196	0.505302
UST	-0.76826	0.505301
AC079922.1	2.074673	0.505041
ZNF630	0.420931	0.505024
AL451085.2	-0.14646	0.504967
AC067838.1	1.104682	0.504771
COMMD3	0.264591	0.504711
GOT1	0.145614	0.50467
AC018635.1	-0.24047	0.504645
LZTFL1	0.16104	0.504608
AL133230.1	-0.30735	0.504515

CD81	-0.13666	0.504334
EEF1A1P13	0.566292	0.504251
AL121895.1	1.601944	0.504154
RASGRP2	0.475997	0.504144
AC103810.5	1.470765	0.504117
BX470102.1	1.960408	0.504059
PIK3IP1-AS1	0.785365	0.504022
FOXO3	-0.11348	0.503883
AC016723.1	-1.72326	0.503867
H1F0	0.099336	0.503743
ZC3H6	0.188307	0.503718
NA	-1.95846	0.503712
PLXND1	-0.15131	0.50368
FZD6	-0.22714	0.503657
MET	-0.26878	0.503553
AF111167.2	0.907869	0.503549
EDN1	0.602628	0.503415
PPP2R5B	-0.16598	0.503243
VN1R83P	0.749537	0.503227
KAT2B	-0.2383	0.503215
SNORD14E	1.764163	0.503179
ARHGEF12	-0.13865	0.503155
AC024575.1	-0.55519	0.503147
C9orf147	-0.46647	0.503106
WDR1	0.114004	0.503106
NUAK1	0.262489	0.503077
AC108134.4	-1.94248	0.503071
AC078883.2	1.273918	0.503037
AC024558.1	-1.87582	0.503031
MIR25	0.286337	0.503025
OSER1	0.143632	0.502975
KCNMB3	-0.43129	0.502872
THAP7	0.153781	0.502826
NA	-0.52125	0.502752
NA	-2.15743	0.502722
MEA1	0.107103	0.50272
RNF222	-2.22097	0.502708
GPRASP2	-0.18902	0.502514
FKBP8	0.11914	0.502479
AC068790.5	1.155857	0.502442
ARHGEF39	-0.16729	0.502244
CUL3	-0.11336	0.502126
ZNF502	0.245324	0.502056
AC078842.1	-0.60455	0.502025
AL035409.1	0.879424	0.502017
GTF2IP5	-0.84471	0.50201
LETM2	-0.30226	0.501993
ISM1-AS1	-2.80061	0.501887
ZNF786	-0.1871	0.501872
LRIG2	-0.15605	0.50176

ARSA	-0.2158	0.501728
ZSWIM6	0.135804	0.501722
XYLT1	-0.19568	0.501714
SPRY1	0.249195	0.501625
CYP4V2	-0.14872	0.501379
LINC00313	2.292835	0.501283
LUC7L3	0.104713	0.501277
AC099518.5	-0.26908	0.501271
C12orf45	0.279335	0.50118
TMEM9B	0.143325	0.501125
REP15	0.682702	0.501115
AC019163.1	0.235981	0.5009
FAM86HP	0.648152	0.500884
HTR4	0.912952	0.500837
MIR4312	1.705251	0.500662
OCLNP1	-0.64229	0.50052
RPLP0P2	-1.24233	0.500485
ZNF230	0.199777	0.500455
CAPRIN2	0.135024	0.500394
CITED4	0.535119	0.500314
TMEM179B	-0.15554	0.500074
AP002840.1	-2.0422	0.500041
NDUFAF6	0.204287	0.500039
ACTR1A	-0.09895	0.499928
PCDHA14	0.63335	0.499839
UQCRB	0.12637	0.499772
AC013356.4	-0.66755	0.499758
RPS23	0.128111	0.499715
AC073352.1	-1.03651	0.499671
AL445931.1	0.815431	0.499657
HSPA4L	-0.23	0.499594
RAB6C-AS1	0.660115	0.499548
Z97652.1	-0.46449	0.499481
ZNF433-AS1	0.348965	0.499411
HHIPL1	-0.64765	0.499189
AC146944.4	0.633366	0.499144
CEP290	0.157823	0.498979
TAGLN3	0.215048	0.498955
AC009065.4	0.370058	0.498943
TNFSF12	-0.34774	0.498675
COG2	0.141702	0.498622
CYB561D2	-0.15247	0.498502
P4HB	0.097649	0.498441
NA	-0.28585	0.498396
AC004475.1	-0.16196	0.498357
POFUT1	-0.1196	0.498337
WSB1	-0.11404	0.498287
AC026785.2	1.361976	0.498248
HEG1	-0.1977	0.498197
GCC2-AS1	0.423106	0.498157

RADX	-2.81043	0.498124
AC134312.2	-2.81043	0.498124
PPP1R14A	0.69634	0.497676
AC091390.4	0.690748	0.497634
AC078819.1	1.216779	0.497633
TEX43	-2.80053	0.497525
JAG1	-0.2601	0.497511
AC061975.1	-2.61371	0.497481
AC019118.2	2.641622	0.497477
EGLN3P1	2.641622	0.497477
AC010336.6	-0.2309	0.497413
AC092375.2	0.459853	0.49726
LCLAT1	-0.16492	0.497258
CEP63	0.163386	0.497232
GBAP1	0.169144	0.497007
POM121L10P	1.33204	0.496918
ATXN7L2	0.185094	0.496785
AC037487.1	1.672552	0.49677
NA	-0.25001	0.496763
UBE3B	-0.143	0.496711
GPR62	-0.5336	0.496702
EID3	1.804821	0.496586
FAM160B1	0.157976	0.496522
ITGA2	-0.15841	0.496518
SLC1A7	1.415269	0.496371
SSUH2	-0.45799	0.496283
NCAPG	0.14054	0.496263
CFLAR-AS1	0.432059	0.496261
RPS2P1	1.01199	0.496206
TESK1	-0.18895	0.496189
KRT8P29	-2.6411	0.496111
HDX	0.271751	0.496052
AC010719.1	1.892691	0.49604
TDRKH-AS1	0.281486	0.495967
E4F1	0.167439	0.49593
MIR4263	1.861851	0.495926
NA	-2.31216	0.495896
PDE12	-0.12591	0.49585
AL928654.2	0.16567	0.495784
Z93930.2	1.099891	0.495776
AL445305.1	2.638964	0.495716
AL021392.1	2.638964	0.495716
SERPINB7	-0.72891	0.495711
HCG17	-0.69247	0.49567
PLEC	-0.16973	0.495663
EEF1DP7	-2.08179	0.495571
AC091152.1	-2.6164	0.495495
GPD2	0.169836	0.495343
HSD3B7	-0.32401	0.495323
ZNRD2	-0.16799	0.495319

KCNH1-IT1	-1.90624	0.495281
WDR88	0.499637	0.495259
CHKB	0.784437	0.49515
ID4	0.155773	0.495015
ATP5PDP4	-1.65568	0.494955
CLPX	0.131467	0.494901
EEF1B2	0.100474	0.494841
PBK	0.166055	0.494793
AC009121.2	1.405395	0.494721
NA	0.814839	0.49472
AL391834.2	-0.40781	0.494621
AL121906.1	-1.45389	0.494536
ZIC5	-0.56732	0.494517
MZB1	-2.80048	0.494476
NT5C1B	-2.80048	0.494476
AP000593.2	-2.80048	0.494476
AL360219.1	-2.80048	0.494476
PLN	1.19494	0.494468
USP32P3	-0.48334	0.494383
CNTNAP2	0.240208	0.494293
FUT6	3.412243	0.494271
SLAMF9	3.412243	0.494271
IFIT1B	3.412243	0.494271
AC114801.1	3.412243	0.494271
FRG2JP	3.412243	0.494271
AC009088.3	3.412243	0.494271
POU2F2	-0.5648	0.494139
POLR2A	-0.14525	0.494126
NA	-0.26894	0.494115
ZNF776	0.166725	0.494036
CPEB2-DT	-2.61226	0.493941
ZNF114-AS1	-2.61226	0.493941
IL15RA	1.836726	0.493941
MYLK-AS1	-0.38112	0.493924
AC174071.1	-0.7795	0.493885
PDCD5	0.180952	0.493857
RASL11A	0.634143	0.493852
AC009902.3	-0.69096	0.493805
AL596087.1	1.037054	0.493784
TNFAIP2	0.263404	0.493677
BCKDHB	-0.15237	0.493573
RUFY4	3.247589	0.493476
MIR571	3.247589	0.493476
TUBB8P2	3.247589	0.493476
HNRNPA1P12	3.247589	0.493476
RF00019	3.247589	0.493476
UFL1-AS1	3.247589	0.493476
COX7CP1	3.247589	0.493476
AP001646.3	3.247589	0.493476
AC027228.1	3.247589	0.493476

PLS3-AS1	3.247589	0.493476
AL008582.1	3.247589	0.493476
FAM43B	0.316944	0.493171
CCM2L	0.521552	0.493017
CDHR5	0.529295	0.493009
AL133453.1	-0.26671	0.492849
GPD1	-1.70533	0.492746
FAM201B	0.878513	0.4927
ZNF568	0.221271	0.492656
YEATS4	0.158084	0.49263
C9orf40	0.206697	0.49255
RBL2	0.109079	0.49247
AC005614.1	0.810616	0.492394
NA	1.66006	0.492378
AC006538.1	0.113977	0.492244
DGKH	0.176227	0.492183
NUP43	0.125476	0.492163
AC124242.2	-1.30881	0.492127
AL137127.1	-0.85502	0.492035
POTEM	1.007748	0.492028
FUT1	1.164586	0.491981
RABGEF1	0.248101	0.491937
SKI	-0.16093	0.491867
CCDC200	1.10719	0.491828
CPNE9	1.416782	0.491671
AHRR	0.453724	0.491637
POC1A	-0.19684	0.491577
SEMA4B	-0.15953	0.491564
MTDHP1	1.187051	0.491555
BOLA2B	0.48383	0.49143
AP003498.1	-0.4776	0.491385
ALKBH1	0.207206	0.491317
SHROOM1	-0.28127	0.4912
LAMP2	-0.13717	0.491186
NOP16	0.115295	0.491081
KCTD9P2	2.198007	0.491023
RSPH4A	0.667128	0.491022
SPCS1	0.123539	0.490967
NA	-0.17131	0.490919
ZNF887P	-0.58626	0.490868
ARVCF	-0.12207	0.490515
FNDC10	0.238912	0.490514
ARSD-AS1	0.424994	0.490419
TMEM63C	0.418504	0.490349
AC008514.2	1.871967	0.490286
RNASEL	-2.49407	0.490274
NUDT9P1	-2.49407	0.490274
ELMSAN1	0.181087	0.490262
AC011500.3	0.451674	0.490243
TNRC6A	-0.1021	0.490236

ELOVL2-AS1	2.651607	0.490174
AL356740.3	2.651607	0.490174
IL24	0.874665	0.490149
OSBPL9	0.104496	0.490026
COPG1	-0.10488	0.490011
AC007250.1	2.651618	0.489983
TXNL1	0.127966	0.489929
GCAT	0.194104	0.489904
NGLY1	0.17743	0.489897
CHCHD5	0.213809	0.489855
SPOCK2	0.279392	0.489852
SFXN1	-0.1099	0.489851
NA	-2.61491	0.489828
AC124944.2	0.368929	0.489794
ZNF585B	-0.18017	0.48948
CTU2	0.198185	0.48946
EXOSC7	-0.15082	0.489442
METTL17	0.110205	0.489393
RF00012	-1.76915	0.489375
SIAH1	0.133325	0.489346
TNK1	0.351283	0.489262
SAMD1	-0.12207	0.48926
NA	2.501337	0.489205
RSL24D1P11	-2.1658	0.489037
COL15A1	-0.39026	0.48891
SMG7-AS1	0.680434	0.488845
C2orf40	-3.2168	0.48882
TSPAN16	-3.2168	0.48882
TEX36	-3.2168	0.48882
OR51B4	-3.2168	0.48882
SNORA1B	-3.2168	0.48882
TRDC	-3.2168	0.48882
HNRNPA1P68	-3.2168	0.48882
AC016065.2	-3.2168	0.48882
AC090572.2	-3.2168	0.48882
AC006504.4	-3.2168	0.48882
NA	-3.2168	0.48882
NA	-0.30811	0.488797
AP002360.1	0.411735	0.488792
TMEFF1	0.309793	0.488501
NA	-0.13202	0.488498
IRF2BPL	-0.16206	0.488454
SH3RF3	0.326212	0.488438
PTRHD1	0.168529	0.488391
SLC5A4	-2.64102	0.488357
AL352979.2	0.463083	0.488278
RPS12P28	-1.03357	0.488181
ADGRL4	-3.21498	0.488143
ANGPTL7	-3.21498	0.488143
FAM180B	-3.21498	0.488143

RNA5SP263	-3.21498	0.488143
KRT81	-3.21498	0.488143
LINC00601	-3.21498	0.488143
AC006387.1	-3.21498	0.488143
AP005018.1	-3.21498	0.488143
NA	-3.21498	0.488143
AL353803.5	-3.21498	0.488143
DGCR10	-3.21498	0.488143
NA	-0.10532	0.487935
MIR374B	1.678242	0.487929
USB1	0.132656	0.487806
STAMBPL1	0.157843	0.487768
AL365436.2	2.51628	0.487698
TMEM234	-0.23315	0.487513
CGB5	-2.61624	0.487436
AL162171.2	0.709045	0.487271
CABIN1	-0.13422	0.487265
INO80-AS1	0.488408	0.487147
CYP3A5	-0.61689	0.487112
PHTF1	0.134219	0.486985
DAGLB	-0.1847	0.486905
OSR1	-0.89354	0.486905
AL035530.1	-2.76961	0.486883
AL133297.1	2.14816	0.486867
RHBDL1	-0.35635	0.486796
CMTM3	-0.14674	0.486747
KLHL7	0.115928	0.486559
NA	-1.00286	0.486533
MMP3	-0.98247	0.486249
STAG3L5P	0.257922	0.486008
LACTB2-AS1	0.346187	0.48598
ZNF680	-0.18389	0.485949
PVALEF	-1.18032	0.485879
LRCH3	-0.11565	0.485855
GRTP1-AS1	0.5939	0.485777
TGFBR2	-0.18809	0.485674
AC078923.1	-2.4942	0.485665
CNKSR2	0.383423	0.485643
LAGE3P1	2.1836	0.485583
TIMM23B	0.577936	0.485571
TRIM14	0.159982	0.485462
LINC00663	-0.41459	0.485346
AC008154.2	-2.6328	0.485321
ARFIP1	0.134293	0.485189
EEF1A1	0.09386	0.485161
TRIR	0.099647	0.485069
LRRC75A	0.097033	0.484979
NA	-2.61068	0.484882
AC007540.1	-0.81155	0.484834
CSF3	1.073423	0.484736

CHSY1	-0.12136	0.484691
NA	1.121744	0.484674
CARD11	-0.9024	0.484627
CENPV	0.10571	0.48462
AC091390.3	0.529165	0.484607
H2BFS	2.182632	0.484474
FOXA3	-0.44244	0.484422
MCUR1	0.146699	0.484243
ZBED3-AS1	-0.29939	0.484224
PXDN	-0.11168	0.484071
WNT11	1.101453	0.484032
AC009302.1	2.615602	0.484031
PIGN	0.162895	0.483996
AL354733.2	0.704981	0.483865
INTS4	0.12282	0.483801
GANAB	-0.09634	0.4838
L3MBTL1	0.224562	0.483772
RPL36AP26	-2.44443	0.483734
PLP1	0.673781	0.483726
DCTN1	-0.10914	0.483668
DHX33	-0.1159	0.483661
MBOAT2	-0.11527	0.483328
RHOC	-0.15035	0.483321
CALCOCO1	0.128822	0.483261
TSPAN18	-0.11976	0.483215
CSGALNACT1	1.88545	0.483137
ANK2	-0.15285	0.48303
UBE2V1P2	-2.46867	0.482989
TNFRSF12A	0.14826	0.482915
OR7E38P	0.343812	0.482886
SMYD3-IT1	-2.61752	0.482846
NSUN2	-0.11303	0.482815
KDELR3	-0.30785	0.48281
AL136115.1	2.624825	0.482796
MIDN	-0.16003	0.48258
C12orf49	0.150187	0.482534
ICA1	0.148339	0.482514
PHBP12	-2.4943	0.482432
DDX50	0.13001	0.482304
AC034236.2	0.346611	0.482176
AC090360.1	0.959495	0.482071
AC005828.3	-1.58749	0.482051
CAMKK1	0.314029	0.482016
ARL10	0.099671	0.481997
AC117402.1	1.627204	0.481921
PDE8A	-0.15872	0.481875
AC004943.2	0.4988	0.481827
TMED5	0.12978	0.48178
RNF157-AS1	-0.27877	0.481771
SLC22A18AS	0.806006	0.481689

AC032044.1	2.246233	0.481581
EGFEM1P	-0.44433	0.481577
AL035413.1	-0.50513	0.48152
HPDL	-0.69494	0.481496
AL139393.3	-0.33621	0.481484
NUDT19	-0.18188	0.481463
RNU5E-8P	-3.18333	0.481453
RNU6-1250P	-3.18333	0.481453
GTF2IP9	-3.18333	0.481453
PCDHGA10	-1.4471	0.481423
ZNF778	-0.19102	0.481389
HOXD4	-0.77754	0.481247
HNRNPA3P1	1.754528	0.481224
SURF2	-0.14619	0.481223
EPS8L2	0.519026	0.481144
AC019131.2	-0.94079	0.480969
TRAPPC2L	0.160581	0.480939
RAB11FIP2	-0.13913	0.480902
AL590867.2	0.317408	0.480875
SLFN12	-0.82611	0.480869
HMGB1P10	-0.47885	0.480825
RPL35A	0.120153	0.480824
CRTC2	-0.11693	0.480728
OLFM4	3.198921	0.480716
HSPE1P26	3.198921	0.480716
ZBTB20-AS1	3.198921	0.480716
AL031600.2	3.198921	0.480716
HERC6	-0.3548	0.480507
AADAT	0.16061	0.480436
AP001001.1	-0.23582	0.480414
RPL3	0.102379	0.480375
ZNF639	0.128205	0.480324
MYL9	-2.61057	0.48031
AC105129.1	-2.61057	0.48031
RPL6P27	0.185103	0.480289
IFI27L2	0.159089	0.480288
SMC5	0.140995	0.480273
SERP2	-0.21117	0.480255
NA	-2.47022	0.480216
TXNDC12-AS1	2.144504	0.480196
AC073896.2	0.185691	0.480172
XPO4	-0.12078	0.480102
ELAVL1	-0.12075	0.479894
NA	0.089648	0.47987
AGMAT	-0.34471	0.47974
SNAPC2	0.162507	0.479724
AC034228.2	-2.61745	0.479646
CLIC4P1	-2.61745	0.479646
HNRNPA1	0.100796	0.479595
DOLPP1	0.140764	0.479558

SUN3	2.624681	0.479543
FTCD-AS1	2.466417	0.479448
AC079610.1	1.95249	0.479445
ITGA8	2.145453	0.479385
NA	-0.6255	0.479377
BACH1-IT2	1.175478	0.479346
FLAD1	0.121459	0.479275
MTREX	0.140618	0.479262
AC012213.3	-2.44596	0.479211
AC012184.1	0.234833	0.479059
LRP2	-3.35645	0.479025
SLC7A8	-3.35645	0.479025
APOA5	-3.35645	0.479025
ADAM20	-3.35645	0.479025
TRAJ39	-3.35645	0.479025
AC090071.1	-3.35645	0.479025
RPS10P18	-3.35645	0.479025
PRB4	-3.35645	0.479025
SLFN14	-3.35645	0.479025
RN7SL385P	-3.35645	0.479025
AC026726.1	-3.35645	0.479025
LINC02373	-3.35645	0.479025
LINC02176	-3.35645	0.479025
AC010287.1	-3.35645	0.479025
AC010976.2	-3.35645	0.479025
MRTFA	-0.19545	0.479024
DYRK3	-0.15411	0.478973
POLR2KP1	-0.86965	0.478962
CCDC134	0.509674	0.478931
EYS	0.721751	0.478916
NA	0.503197	0.47881
C2orf69	-0.19129	0.478809
TSSK3	-0.23066	0.478751
ABT1	-0.14448	0.478614
ARHGAP1	-0.13118	0.478514
HYDIN	1.253003	0.478461
RNF215	0.167512	0.478459
WFDC2	1.977725	0.478151
ZCRB1	0.136126	0.478132
C20orf144	-0.60542	0.47811
ZFHX2-AS1	0.45695	0.47807
ZFAND5	-0.0974	0.477761
NA	-0.51499	0.477751
STARD7-AS1	0.159549	0.477682
AC018904.1	0.474546	0.477679
RF00283	1.64449	0.477654
TCTEX1D2	0.438852	0.477635
AC015917.2	-0.53984	0.477619
JADE3	0.136661	0.477608
FCER1G	0.997373	0.477581

AC025918.1	-0.31232	0.47745
AC137630.3	0.208042	0.477433
PCAT18	-1.45591	0.477427
NA	1.39369	0.477387
CKLF	-0.35861	0.477333
MMP15	-0.35626	0.477263
RF01210	-2.6105	0.477108
AC009113.2	-0.33408	0.476949
KCNK2	-1.47398	0.476841
NPC2	0.138529	0.47683
NA	2.594823	0.47682
JAM3	-0.15124	0.476799
DEF6	1.737761	0.476735
NA	2.594831	0.476622
AC005921.2	0.154819	0.476465
DNAJC17	0.208696	0.476419
FN1	-0.28906	0.476357
PGAM1	-0.09982	0.476333
AC007391.1	-2.05064	0.476205
RILPL2	0.225139	0.476175
CFAP46	-0.41224	0.47617
MTCO1P2	2.161037	0.476115
SCARNA6	1.298713	0.475983
FSD2	0.221679	0.475803
AC004951.2	-1.21094	0.475759
EIF4G1	-0.10645	0.475733
UHRF2	0.139014	0.475733
AC134772.1	0.146092	0.475696
DUSP19	-0.31058	0.475604
HSBP1L1	-0.2626	0.47549
CREB3L4	0.160293	0.475474
RNU6-97P	-2.44445	0.475454
AC008073.2	-2.44445	0.475454
PIK3CG	-3.34636	0.475414
WFDC12	-3.34636	0.475414
AC008267.1	-3.34636	0.475414
HTR1D	-3.34636	0.475414
NA	-3.34636	0.475414
AL096700.1	-3.34636	0.475414
MIR26B	-3.34636	0.475414
RNU6-670P	-3.34636	0.475414
RNU6-460P	-3.34636	0.475414
KRT42P	-3.34636	0.475414
AC006015.1	-3.34636	0.475414
USP17L7	-3.34636	0.475414
NA	-3.34636	0.475414
STMND1	-3.34636	0.475414
LINC01647	-3.34636	0.475414
AC073130.1	-3.34636	0.475414
RPS2P39	-3.34636	0.475414

RN7SL815P	-3.34636	0.475414
AC106882.1	-3.34636	0.475414
AC113352.1	-3.34636	0.475414
AC022613.1	-3.34636	0.475414
OR11H12	-3.34636	0.475414
LINC02319	-3.34636	0.475414
LINC00165	-3.34636	0.475414
RN7SL744P	-3.34636	0.475414
NA	-3.34636	0.475414
AC015813.3	-3.34636	0.475414
NA	-3.34636	0.475414
STX8P1	-3.34636	0.475414
NPAS1	0.507456	0.475393
ZNF527	0.27696	0.475383
SIPA1L3	-0.15602	0.47533
ARPIN	-0.20368	0.475264
RRP1B	-0.11778	0.475227
RPL22P1	0.509007	0.475213
IL10RA	2.756088	0.4752
AL357078.1	2.756088	0.4752
IL21R-AS1	2.756088	0.4752
TSPAN1	-1.45671	0.475038
DTYMK	0.122336	0.475036
SLC2A11	0.183199	0.474953
FAM166B	1.208671	0.474912
NA	0.320976	0.474867
CCDC127	-0.14246	0.474789
MAP2K5	-0.18402	0.474762
MTND6P4	1.09365	0.474438
EIF3F	-0.11166	0.474407
AC027601.3	-0.41317	0.474395
MAGEA10-MAGEA5	1.453814	0.474379
HSD11B1L	0.190421	0.474347
LINC01102	-0.46772	0.474248
NA	0.216819	0.474245
SERPINA6	-1.05458	0.47423
NA	-0.20444	0.474194
AC092802.1	-2.03279	0.474162
SUMF2	0.107804	0.474154
POLR2F	0.137172	0.474136
AC025176.1	-1.13566	0.474069
ACTRT3	0.33037	0.474027
FBXL15	0.176742	0.473898
FRRS1	0.836325	0.47387
RPS6KB2	0.131739	0.473863
UBXN2A	-0.13573	0.473827
LSM1	0.114431	0.473782
SEMA3F-AS1	0.154137	0.473707
TBC1D22B	0.186747	0.473702
FANCC	-0.15513	0.4736

SYBU	0.193232	0.473537
BECN1	-0.11509	0.473498
TAF4B	0.18246	0.473418
MELTF-AS1	0.30391	0.473366
KCNN2	2.030215	0.473308
ZW10	-0.19858	0.473167
PPA1	0.112152	0.473162
NCLP1	-0.76616	0.47313
NA	2.575351	0.472879
AC073957.2	-0.40794	0.472758
RPL18	0.116404	0.472733
COLGALT1	-0.14207	0.472726
ADSL	0.120269	0.472682
PLEKHG3	-0.20873	0.472673
AC008063.1	1.884453	0.472595
CALM2P2	0.55036	0.472584
PSMC1	0.108614	0.47258
AP000523.1	0.341223	0.472574
APH1B	-0.20345	0.472316
AC024560.2	-0.49003	0.472272
ZNF665	-0.53126	0.472244
AC020909.4	0.118369	0.472191
GAMT	-0.12922	0.47209
AL356801.1	-0.92202	0.472083
AC012615.2	0.272478	0.472017
PMS2P5	0.330214	0.471989
NA	1.076285	0.471868
MYO3B	-1.76218	0.471851
DCTPP1	0.124	0.471848
SPIN1	0.099126	0.47175
KARS	0.100506	0.471701
RF00432	-3.1568	0.47168
AP002490.2	-3.1568	0.47168
AC011603.1	-3.1568	0.47168
TMEM268	-0.16765	0.47146
RRNAD1	0.148156	0.471442
AC022395.1	-0.47802	0.471437
GMNN	-0.13834	0.471423
CCDC180	-0.55986	0.471416
LINC00640	2.189834	0.471313
RBBP4P1	1.095491	0.471306
AC012629.2	-0.2867	0.471254
MBL2	-1.75077	0.471241
CDNF	0.323613	0.471227
AC131097.1	1.422354	0.471207
EHD4	0.172269	0.471194
STMN3	0.101688	0.471058
NA	1.320459	0.471042
SEPT7P9	-1.35573	0.471008
DGCR2	-0.11635	0.470981

FTSJ1	-0.14182	0.47089
AC109460.2	0.261326	0.470771
NA	2.591716	0.47067
AC027277.2	2.591716	0.47067
PLD2	-0.20511	0.470667
RPAP1	-0.11973	0.470577
TCTE3	0.689314	0.470535
ZNF695	-0.41286	0.470534
NA	0.784701	0.470533
VEGFC	-0.65538	0.470473
CCL7	-0.42803	0.470378
GAS1	-0.21651	0.470317
UPF1	-0.13801	0.470258
NA	-0.17428	0.470222
HEMK1	0.157401	0.470149
AL109761.1	-0.82311	0.470132
ATP1A3	-0.133	0.470038
PRR7	0.204844	0.47001
IL12A-AS1	-1.10591	0.469972
AP001020.2	0.955586	0.469905
AHDC1	-0.20365	0.46977
SLC22A7	2.260312	0.469757
AP001453.1	0.205595	0.469733
NA	-0.91753	0.469693
PYGO2	-0.11428	0.469679
AP003396.5	-0.18486	0.469674
RNU6-2	-3.15112	0.469609
PIP5K1A	-0.10397	0.469595
LINC02356	2.390037	0.469578
SNORA54	-2.41992	0.469521
RPL26P30	-2.41992	0.469521
TH2LCRR	0.173155	0.469487
RF01241	1.006505	0.469379
TRIM67	-0.21827	0.469216
PHEX	1.155781	0.469188
AC011511.5	0.263488	0.469126
ARL6IP4	-0.58691	0.469119
ASIC2	2.727997	0.46906
AL358075.1	2.727997	0.46906
DLL1	0.64916	0.468987
AC016745.1	-2.41838	0.468955
ABCB7	0.160049	0.468929
DDX28	-0.19346	0.468913
NA	0.31375	0.468814
NA	-0.13564	0.468809
FAM234B	-0.2253	0.46872
HIST2H2BA	-0.48743	0.468716
ARHGDIB	-0.72398	0.468709
PGAM1P7	-2.07407	0.468431
MAP3K6	-0.29915	0.468263

FGD3	1.193825	0.468062
EIF2S1	0.112134	0.468021
MPZL1	-0.1068	0.467962
RAG1	-0.92065	0.467914
RGS10	0.31614	0.467792
CD160	0.396972	0.467641
NA	0.141161	0.467557
LINC00222	2.591511	0.467405
COX7A2P1	2.591511	0.467405
CEACAM1	1.357856	0.467323
AC106760.1	1.632077	0.467287
DAB2	-1.71789	0.46721
PTMAP2	0.450522	0.467126
PPP1R36	1.04254	0.466996
UBE2Z	-0.09388	0.466972
SLC39A13	-0.13077	0.466965
BX537318.1	0.774288	0.46684
AL442071.1	-0.89415	0.466806
BEX3	0.110051	0.466802
UBC	0.103351	0.466754
SERAC1	-0.21045	0.466649
ATL3	-0.16556	0.466498
ZNF668	0.171519	0.466443
N4BP2	0.154694	0.466361
AC067945.3	-2.22714	0.466321
MILR1	-1.86924	0.466308
C9orf72	0.159822	0.466294
SNORA9B	-1.33931	0.466272
A2M	0.802949	0.466124
CBLL1	0.116568	0.466109
NA	2.727976	0.466038
PITX1	3.172927	0.465936
KCTD4	3.172927	0.465936
SLC2A3P2	3.172927	0.465936
SNORA14A	3.172927	0.465936
OR7E122P	3.172927	0.465936
GLRX3P2	3.172927	0.465936
HIGD1AP16	3.172927	0.465936
ABCC5-AS1	3.172927	0.465936
AC007036.1	3.172927	0.465936
AL445928.1	3.172927	0.465936
RPS27AP2	3.172927	0.465936
DDX3P1	3.172927	0.465936
RPL7P13	3.172927	0.465936
AC007731.2	3.172927	0.465936
AC008494.2	3.172927	0.465936
CDC42P4	3.172927	0.465936
AP003059.2	3.172927	0.465936
TMEM238L	3.172927	0.465936
AC104982.1	3.172927	0.465936

AP001198.1	3.172927	0.465936
TDGF1P7	3.172927	0.465936
AC006483.2	3.172927	0.465936
AC073316.3	1.500852	0.465915
AC012254.2	1.020267	0.465899
TMPRSS5	-1.06645	0.465877
SPTLC1P1	2.727975	0.465852
AC087500.2	2.727975	0.465852
TOP1	-0.09748	0.465784
GAPDHP63	1.920516	0.465718
NAGK	-0.13451	0.465557
AC100778.3	0.246818	0.465554
CCDC183	0.514543	0.465428
NEIL3	0.193627	0.465339
ZBTB3	-0.29883	0.465303
NT5C2	0.117619	0.465212
AZIN2	0.318773	0.465208
AC138811.1	0.334452	0.465173
SDCBP2-AS1	0.269178	0.465131
ROCK1P1	-0.69268	0.465122
OASL	1.336479	0.464975
RILPL1	0.205145	0.464911
C1orf198	-0.12854	0.464822
LSMEM1	-0.35495	0.464775
CBWD4P	-0.64792	0.464629
AL139412.1	0.790942	0.464572
AC073127.1	-2.14895	0.464466
CDKL5	-0.25326	0.464422
UBA52	0.099808	0.464351
NEIL1	0.193195	0.464336
AC097376.2	0.289453	0.464302
C9orf24	0.865734	0.464265
EIF1AX-AS1	-2.41831	0.464174
PPP1R13L	-0.26964	0.464149
AC005387.2	-1.23228	0.464131
NECTIN4	1.052118	0.464095
C2orf50	-1.56239	0.464059
ACAP2	-0.13384	0.463974
ZNF385D	0.470995	0.463901
OCLN	-0.28896	0.463648
SFTPA2	2.709642	0.463614
RPL7AP15	2.709642	0.463614
APOBEC2	-2.24044	0.463391
KIF4A	-0.13265	0.463288
HCLS1	2.440616	0.463277
AL157384.1	2.440616	0.463277
SOX9	-0.24341	0.46326
AC027682.2	-0.37733	0.463252
MAPK7	-0.14952	0.463226
PPARGC1B	0.402351	0.46313

TTC34	-1.30584	0.463085
KCND3	-1.22696	0.463081
BCAP29	0.137481	0.463019
PRKAR2A	-0.13821	0.462821
CAMK1D	0.167355	0.4628
AP000349.1	-0.20887	0.462795
VAMP4	0.135882	0.46276
BAG1	0.106019	0.462698
GCSHP5	1.377189	0.46268
RRP36	-0.11163	0.462607
NA	-0.26396	0.462563
IRF9	0.357942	0.462536
ZMYM4-AS1	-0.32018	0.462492
RPL26	0.161574	0.462489
ATP8B1	-0.31826	0.46242
GYG2	-0.84986	0.462368
NA	0.156856	0.462294
AC092143.2	-0.39541	0.462276
CEP164P1	0.167895	0.462241
GNL1	0.105819	0.462119
AC009163.1	2.01669	0.462106
TRPS1	-0.43591	0.462079
AC102953.2	-0.21152	0.462075
BBS9	0.195859	0.462069
SNX7	0.137415	0.462012
RAB33A	0.252793	0.461686
RAB3GAP2	0.104949	0.461653
ADAMTS15	-1.02516	0.461647
FRG1BP	0.137276	0.461585
STK32C	0.155844	0.461508
HNRNPA1P36	-2.41982	0.461385
AC018755.1	-2.14361	0.461341
NEU1	-0.13765	0.46131
GASK1A	2.091006	0.461294
ANKRD6	0.211152	0.461292
SP2	-0.17197	0.461198
SOD2	0.099384	0.461154
AP001469.1	0.31769	0.461088
AC016355.1	0.589501	0.460992
LEKR1	-0.80568	0.460912
ATP6V0E1	-0.12402	0.46087
FICD	0.224696	0.460848
ZNF259P1	-2.41827	0.460818
LINC01433	2.70985	0.4607
AC079089.1	2.70985	0.4607
AC010468.2	2.70985	0.4607
AP000347.1	0.445659	0.460699
SUCLA2-AS1	-1.55129	0.460587
ATG2B	0.119332	0.460551
RNF6P1	2.709863	0.46052

AC110741.1	2.709863	0.46052
NA	2.709863	0.46052
AC060766.7	2.709863	0.46052
CALHM2	-0.22497	0.460519
ZNF529-AS1	-0.30854	0.460458
CAAP1	0.157323	0.460294
TBRG4	0.10253	0.460279
AC145343.1	1.67353	0.460199
SMAD7	-0.13641	0.460133
ORAI2	-0.11468	0.460125
FBLIM1	0.181741	0.460044
ABI3BP	0.26655	0.459958
AC135803.1	2.440487	0.459958
THSD7A	-0.13086	0.459946
GPR68	-0.26817	0.459862
DUSP2	0.240588	0.459728
TIMP2	-0.12225	0.459641
HIST1H2BC	-0.37309	0.459405
OTUD5	0.121764	0.459348
AC139795.2	-0.29051	0.459261
CROT	-0.17274	0.459259
LINC01415	-0.53912	0.459253
AP003043.1	3.140958	0.459243
SPTY2D1OS	0.26062	0.459224
AC020934.2	0.33945	0.459223
TMEM243	0.133358	0.45919
AC131235.3	1.864513	0.459071
AC004832.5	-1.69173	0.45895
RPL21P89	-1.61288	0.458912
HSPA13	0.114706	0.4589
OLFM2	0.171901	0.458846
HOPX	-2.42133	0.458777
RPL12P12	1.827639	0.458733
ZNRF2P2	0.401447	0.458719
RHBDL2	-1.16321	0.458655
CCDC74A	0.185363	0.458567
B4GALT1-AS1	-0.48133	0.458523
AC011455.1	1.869372	0.458481
NA	-0.37692	0.458462
MED15	-0.12576	0.458233
DUSP7	-0.15858	0.458224
NA	-0.29438	0.458192
AL391097.1	2.074964	0.457982
NA	-0.44258	0.457925
ANKS6	-0.14517	0.457835
CMA1	2.202024	0.457799
NA	0.245986	0.457671
MBTPS2	-0.11267	0.457498
MCTP1	0.319225	0.457232
KANK3	0.320404	0.457215

ELAC1	0.243831	0.457204
MFSD14C	0.205082	0.457183
AC091180.5	-0.94194	0.457178
TMC2	-1.43111	0.457118
LINC00852	-2.41668	0.457104
ATP6V1G1	0.119279	0.457007
GEMIN4	-0.13531	0.457
MT-CO2	0.095485	0.456837
NGRN	0.108095	0.456764
AL513366.1	2.090967	0.456749
S100A3	0.413223	0.456685
CDH20	-0.89688	0.456661
THSD4	-0.30208	0.45664
WWC1	0.180009	0.456622
GOLPH3	0.112453	0.456603
AC005041.3	-0.90678	0.456599
AC103739.2	-0.58134	0.456367
RNF169	0.149207	0.456245
USP2	0.484528	0.456222
AC022762.2	-0.81911	0.456168
AC008569.1	-0.49045	0.45609
WBP2	0.135053	0.45594
AGT	-0.64418	0.455879
FKBP4P1	2.219934	0.45587
Z99755.1	1.845868	0.455862
ZNF862	-0.1618	0.455837
FAM104B	0.167176	0.45578
ADCK1	-0.27538	0.455779
UTP11	0.107406	0.455769
STXBP6	-3.1259	0.455655
SERPINB9	-3.1259	0.455655
MAGEA1	-3.1259	0.455655
UBE2V2P4	-3.1259	0.455655
BX679664.2	-3.1259	0.455655
AC108667.1	-3.1259	0.455655
KCNMB2-AS1	-3.1259	0.455655
AC122129.2	-3.1259	0.455655
ZNF213	-0.2001	0.455652
EDN2	0.692366	0.455554
AC091180.4	-1.00005	0.455347
AC016738.2	0.754828	0.455296
AL157832.1	-2.09365	0.455274
CAMK2N2	-0.21876	0.455236
GABRG3	0.667159	0.455224
DBNDD1	-0.15918	0.45508
NA	-0.23285	0.455007
ENO1P2	1.003938	0.454951
C19orf38	-1.31206	0.454939
NA	-0.1345	0.454927
TEX45	-1.05261	0.454704

ASXL3	0.302021	0.45463
AC091053.2	-2.07064	0.454628
AC005291.1	-0.33154	0.454448
LINC00622	0.775535	0.454344
TMEM232	0.571344	0.454341
CDK7	0.141568	0.454335
AC079848.1	1.181177	0.454314
INHBA	-0.33674	0.454261
NUP62CL	-0.4734	0.454098
RPLP1	0.105439	0.454039
RPL17	0.237443	0.454017
RAB15	-0.17577	0.453911
NEDD4L	0.17835	0.453892
AC011899.1	-2.41663	0.453843
UNC93B1	0.262974	0.453791
AL023584.1	-0.45107	0.453699
UBQLN2	-0.12445	0.453635
AP001432.1	0.753639	0.453603
AC067863.1	-0.42481	0.453468
PPP1R7	-0.12762	0.453345
SLC25A18	-0.78831	0.453245
PLK1	-0.15816	0.453179
LDHB	0.094945	0.453168
AL079301.1	1.966605	0.453154
CCBE1	-0.8958	0.453119
TPM4	0.0877	0.453109
HAUS8	0.199277	0.453107
MRGPRD	-3.11814	0.452939
LINC00303	-3.11814	0.452939
C10orf67	-3.11814	0.452939
FAM71C	-3.11814	0.452939
AL354993.1	-3.11814	0.452939
AC140479.3	-3.11814	0.452939
LINC01589	-3.11814	0.452939
AC022001.2	-3.11814	0.452939
AC098479.1	-3.11814	0.452939
AC083805.1	-1.80354	0.452904
FCF1	0.128204	0.45284
BTBD16	3.299879	0.452789
CD200R1	3.299879	0.452789
PCSK1	3.299879	0.452789
GDPD4	3.299879	0.452789
PPP3R2	3.299879	0.452789
NPIPB9	3.299879	0.452789
NA	3.299879	0.452789
AC011999.1	3.299879	0.452789
AC025750.1	3.299879	0.452789
BCRP7	3.299879	0.452789
AL513412.1	3.299879	0.452789
SAPCD2P3	3.299879	0.452789

LINC01725	3.299879	0.452789
AC084809.2	3.299879	0.452789
RPL23AP23	3.299879	0.452789
NA	3.299879	0.452789
AC108751.5	3.299879	0.452789
AC024588.1	3.299879	0.452789
CCNL2P1	3.299879	0.452789
HMGB3P15	3.299879	0.452789
NA	3.299879	0.452789
AC026979.1	3.299879	0.452789
AC008438.2	3.299879	0.452789
AC087439.2	3.299879	0.452789
FDPSP4	3.299879	0.452789
AC051619.3	3.299879	0.452789
FBXO36P1	3.299879	0.452789
AC090241.1	3.299879	0.452789
Z83818.1	3.299879	0.452789
AL391069.3	3.299879	0.452789
MARK3	-0.10401	0.452769
RNU6-840P	1.32732	0.452534
C10orf55	-0.38824	0.452398
APOC1	-0.33109	0.452371
TMEM168	-0.12807	0.452306
FZD8	-0.3037	0.452147
UBA6-AS1	0.174827	0.452146
SAMD4A	0.174976	0.452088
AL445435.1	-1.16412	0.452045
AL163051.1	0.610009	0.452027
MARVELD3	1.126192	0.452016
C5orf24	-0.11092	0.451989
NA	0.564733	0.451915
AC007128.1	0.800837	0.451896
STXBP5	0.161775	0.451705
LDLRAD2	-0.36377	0.451658
ARHGAP35	-0.09781	0.451569
ARHGEF37	0.233557	0.451561
ZNF189	0.139337	0.451557
RAC1P2	-0.5962	0.451515
UBXN10	0.56616	0.451475
ZNF624	0.280508	0.451424
COG3	0.119393	0.451413
AC135050.3	1.27195	0.451378
CHCHD2	0.094775	0.451351
LDB3	0.589098	0.451308
AC138409.2	0.240144	0.451205
AC005326.1	-0.74593	0.451176
SH3RF1	0.166299	0.451171
MIPEPP3	0.520038	0.451131
AC099518.4	-0.569	0.451093
WDR25	0.200114	0.45099

FAM205A	2.166995	0.450975
TMPRSS6	-0.7722	0.450856
SULT1A3	1.065736	0.450854
TXNDC9	0.148545	0.45082
POM121B	-0.31916	0.450811
AC008906.1	-0.4062	0.450805
GAP43	-0.12759	0.450724
TMSB15B	0.169104	0.450669
MGAT1	0.10377	0.450607
NA	-0.8609	0.450455
CHD4	-0.0979	0.450436
LRFN1	0.350398	0.450273
DNAJC27	-0.18183	0.450164
NA	2.541233	0.450125
SPAG4	0.291815	0.450071
NPIPB6	-1.43272	0.450011
IGF2BP2	-0.84658	0.449906
CHST11	0.237655	0.449816
AC023794.5	-0.97893	0.44969
PHF19	-0.1181	0.449682
LINC01798	1.273741	0.449672
MYCBP2	-0.11668	0.449666
AC004542.1	0.144269	0.449645
CDC37	0.089712	0.449596
C1orf162	-0.87535	0.44947
FGF19	-2.11148	0.449355
AC087289.1	0.555512	0.449354
AC073389.3	-0.84507	0.449239
NA	-0.28128	0.449206
ETHE1	0.214781	0.449187
AC011495.2	-0.39906	0.449177
SLC24A2	-2.52103	0.449115
OCLM	0.602275	0.44895
RPS26P6	2.035208	0.448914
TMEM230	0.10583	0.44891
MBOAT1	-0.27567	0.448797
ARL5B	0.118311	0.448667
AL162742.1	-0.4553	0.448609
MCCC2	-0.10249	0.448601
PRAF2	-0.1567	0.448594
BRSK2	-0.19465	0.448538
IQCE	-0.11769	0.448359
DMAP1	0.128783	0.448254
AL353622.1	0.243348	0.448248
AL122035.2	0.172968	0.448242
DARS-AS1	0.231105	0.448238
RPL34P18	0.760822	0.448238
C20orf27	-0.12715	0.44823
MYSM1	0.11694	0.448206
HNRNPA3P14	1.841857	0.448202

SCPEP1	0.129082	0.44809
DHRS3	0.476845	0.448081
C1orf94	3.121671	0.448019
GBX2	3.121671	0.448019
RNU6-1010P	3.121671	0.448019
RF00019	3.121671	0.448019
RF00019	3.121671	0.448019
AL591719.1	3.121671	0.448019
PPIAP35	3.121671	0.448019
AP000563.1	3.121671	0.448019
AC058791.1	3.121671	0.448019
VAMP1	0.184538	0.448002
SMAD3	0.150255	0.447944
IL12RB2	-1.05767	0.447898
RF00019	-3.09043	0.447828
NA	-3.09043	0.447828
NPIPB8	-3.09043	0.447828
CCT6B	0.528053	0.447746
MPRIP-AS1	1.942418	0.447655
AC022167.2	0.185507	0.44765
AL663058.1	3.108198	0.447643
AC021086.1	3.108198	0.447643
AL022722.1	-2.49677	0.447584
UBXN10-AS1	-2.49677	0.447584
AC092667.1	-2.49677	0.447584
TIPARP-AS1	0.656298	0.447509
AC006206.1	-1.67233	0.447481
GPR85	-0.24395	0.44746
MORN2	0.181174	0.447402
MMP25	0.462002	0.447318
C2orf91	-0.55748	0.447291
AC018809.1	1.202986	0.44723
TSPAN11	-0.13913	0.447169
RPS15AP16	1.731748	0.447143
RNY1P13	-3.08845	0.447134
LINC00607	-3.08845	0.447134
MUSTN1	-3.08845	0.447134
GUSBP1	-0.23354	0.447124
HNF1A	0.438059	0.447115
ZNF707	0.195245	0.447103
MYO7B	-1.46799	0.447084
AC022211.3	0.385448	0.447003
AC009237.8	-1.94617	0.446996
ACTR8	0.119176	0.446946
GPR88	-1.72367	0.446897
BFSP1	-0.5272	0.446882
CRYZL1	0.143838	0.446792
NME2	0.239784	0.446747
NA	0.264685	0.446656
GNL2	0.107698	0.446653

TMEM167B	0.131042	0.44661
RNU6-398P	-0.68014	0.446599
AXIN2	0.200325	0.446594
SLC36A4	0.126794	0.44657
CALM3	-0.09145	0.446544
NA	-2.49377	0.446538
NA	-0.63259	0.446533
ACTR3B	-0.1342	0.44652
RALA	0.101705	0.446472
STX7	-0.16396	0.446445
ZNF346-IT1	-1.05287	0.446444
CACNA1B	-0.16328	0.446439
FOXP1	0.137145	0.446311
AC011767.1	-0.3646	0.446279
TENT5C	0.852931	0.446067
MTND4P15	-2.52114	0.44604
AP001020.1	1.812387	0.445876
CUL1	0.098927	0.445815
ARHGDIG	0.485412	0.445749
SCAMP4	-0.16118	0.445716
MYDGF	0.131764	0.445654
ELOVL2	0.186152	0.445586
TXNRD3	0.27616	0.445568
GCNT3	-2.51966	0.445528
AC138627.1	-2.51966	0.445528
STK3	-0.19495	0.445478
AC007566.1	-0.26809	0.44531
AC007193.2	1.850136	0.445301
AL136298.1	-1.94163	0.445256
AP003096.1	0.225762	0.445235
AC005255.2	1.615906	0.445201
AC093909.1	-0.31042	0.445134
SCAND1	0.127875	0.445099
AC129492.1	0.205447	0.445059
AC099560.2	0.46011	0.445056
HHIPL2	-0.38359	0.444949
MAGEA12	-0.11909	0.444915
HMGB1P6	-0.15231	0.444889
AP001626.1	0.991891	0.444829
SLC25A30	0.174756	0.444716
NXT2	-0.167	0.444701
NHLRC3	0.150988	0.444444
TUBA3D	0.525621	0.444437
HSD11B2	0.635658	0.444403
EPHB6	2.037803	0.444393
RGPD5	2.149301	0.444392
RNPS1P1	1.133633	0.444345
NA	-2.07104	0.444338
CNTF	-0.50779	0.44427
GKN1	-3.09233	0.443928

TCERG1L	-3.09233	0.443928
SEPHS1P1	-3.09233	0.443928
VN2R17P	-3.09233	0.443928
MMP23A	-3.09233	0.443928
OSTCP4	-3.09233	0.443928
RPL23AP95	-3.09233	0.443928
PSMC1P4	-3.09233	0.443928
NA	-3.09233	0.443928
NA	-3.09233	0.443928
AC009803.1	-3.09233	0.443928
AC015911.4	-3.09233	0.443928
NA	-3.09233	0.443928
AC005828.7	-3.09233	0.443928
NR3C2	0.624253	0.443541
AL162595.1	0.417117	0.443383
CHRNA2	-2.49385	0.44336
AC009078.2	-2.49385	0.44336
PCDHGA2	0.833345	0.443305
EMC2	0.200005	0.443245
YIPF5	0.106586	0.443162
SLC4A2	-0.10216	0.443144
DLK1	0.559289	0.442949
NA	-0.19837	0.442884
NFATC1	-3.24763	0.442849
GSDMA	-3.24763	0.442849
GJA4	-3.24763	0.442849
NA	-3.24763	0.442849
AC012066.1	-3.24763	0.442849
FGF7P8	-3.24763	0.442849
AL357500.1	-3.24763	0.442849
NA	-3.24763	0.442849
RN7SKP198	-3.24763	0.442849
RPS10P28	-3.24763	0.442849
NA	-3.24763	0.442849
AC004847.1	-3.24763	0.442849
TACR1	-2.15914	0.442766
LINC01118	-2.15914	0.442766
AC008750.5	1.152683	0.442709
NBEAL1	0.262343	0.44263
TMEM14A	0.141752	0.442595
AC020907.4	0.541534	0.442492
KNOP1P2	2.065899	0.442425
THY1	-0.12995	0.442398
LINC00449	-0.59542	0.44228
BRD8	-0.11242	0.442018
NA	-1.49092	0.441992
RINT1	0.122026	0.441935
CHST4	-3.08638	0.441872
LINC00208	-3.08638	0.441872
AC003035.1	-3.08638	0.441872

MEF2B	-3.08638	0.441872
AC007422.1	-3.08638	0.441872
SLC25A5P5	-3.08638	0.441872
RPL35AP31	-3.08638	0.441872
RNU7-171P	-3.08638	0.441872
AC019270.1	-3.08638	0.441872
AP000640.1	-3.08638	0.441872
MIR3192	-3.08638	0.441872
ITM2BP1	-3.08638	0.441872
AC005070.1	-3.08638	0.441872
PSMA1	-0.15848	0.441852
MKNK2	-0.13983	0.441822
AC027801.4	-1.10049	0.441782
LRFN2	-0.27985	0.441686
HSPA8	-0.13841	0.441569
AC007040.1	0.352685	0.441566
AC073111.1	0.973128	0.441497
MDK	0.13888	0.441482
LMTK3	0.320075	0.441445
PLPPR2	-0.16644	0.441417
AC105390.1	1.826409	0.441313
PQLC1	-0.16005	0.441255
TRMT112P6	-1.05598	0.441216
AC110285.1	-1.56709	0.441186
AC132872.2	0.933098	0.44111
LINC02520	2.950155	0.441105
SRRM1P3	2.950155	0.441105
USH2A	-1.05428	0.441086
STAB1	0.233173	0.441077
IL27RA	-0.23388	0.441074
UBXN1	0.097841	0.44106
AL450998.2	0.189701	0.440954
PLEKHA1	0.122927	0.440948
ZDHHC2	-0.18348	0.44089
TERF2IP	-0.0992	0.440842
AC103974.1	-0.64229	0.440804
RND1	0.515239	0.440768
FLOT2	-0.10507	0.440724
L3MBTL3	-0.3069	0.440695
DOCK2	-0.45556	0.440686
RGS14	-0.2033	0.440681
AKR7A3	0.649139	0.440569
LETM1	-0.11364	0.440539
ZNF235	0.1239	0.440445
AC114730.2	-0.42358	0.440436
MT-CO3	0.097365	0.440413
CCDC32	0.159352	0.440399
ZCCHC17	0.133388	0.440298
CHMP6	-0.14991	0.440236
LMTK2	-0.1428	0.440225

INSIG2	0.145277	0.440197
NMD3	0.110294	0.440185
SOX2-OT	-1.65833	0.440134
BANK1	1.579253	0.440029
AC096887.1	1.272227	0.440027
NA	0.152904	0.439879
HCG11	-2.12908	0.439848
HMGB2	0.114964	0.43984
MEIS3P2	-0.67432	0.439805
MAMDC4	-0.21965	0.439735
AC051619.4	0.960513	0.439596
PHYHIPL	0.095905	0.439556
COL4A2-AS2	-0.36916	0.439436
AL133330.1	1.4774	0.439341
SPIDR	0.141583	0.439296
PINLYP	0.341267	0.43923
AC010680.1	1.257019	0.439228
AC012358.3	-0.30684	0.439182
PSD4	-0.62664	0.438894
LOXL3	0.120061	0.438893
NPTX1	-2.61808	0.438798
AL158068.2	-2.05611	0.438729
MLST8	0.12831	0.438659
Z73417.1	2.059384	0.438613
AC034228.3	1.797813	0.438541
TENM4	-0.15657	0.438395
RPLP2	0.100271	0.438391
AL136418.1	0.31582	0.438376
MAML3	-0.13664	0.438281
PRC1-AS1	0.1308	0.438273
RAB1B	-0.09283	0.438145
AC112777.1	-0.24793	0.438082
AVPI1	0.367457	0.438059
NAT8	-1.8185	0.438041
SCAF4	-0.11694	0.438032
SLAMF8	-0.90201	0.437946
AC009065.8	0.497259	0.437904
KIF3C	-0.11046	0.437826
TMEM56	-0.30508	0.437739
DECR1	-0.17585	0.437718
SPINDOC	0.112076	0.437668
ARF6	0.113929	0.437621
AL645998.1	1.671531	0.437517
DGKI	-0.15332	0.437453
AC078950.1	-1.55278	0.437436
AC079880.2	1.212368	0.437424
MFSD9	0.190558	0.437354
BCL2	-0.14699	0.437189
AC125437.1	0.97372	0.437078
AL109930.1	0.411221	0.43704

ANO3	0.823847	0.437032
IGHVII-44-2	-0.19832	0.43701
AC008667.2	0.645597	0.436982
GLRX2	-0.1983	0.436763
AL358072.1	-1.77653	0.436649
IGFBP4	-0.11548	0.436574
E2F4	0.097199	0.436562
AC011477.2	-0.36709	0.436524
TOMM20L	0.587622	0.436502
SLC8A2	-0.262	0.43649
INTS2	-0.11977	0.436449
C2orf49	-0.12343	0.43642
AC073861.1	0.268033	0.436408
BTBD18	0.599401	0.436283
SH3PXD2B	-0.11479	0.436255
AC007731.1	-0.56447	0.43622
AC084337.1	-0.44207	0.436182
CCDC18-AS1	0.167849	0.43618
AC090515.2	0.744041	0.436172
TCP1	-0.08855	0.436096
GLIS3-AS1	-1.36917	0.43604
AC092718.4	0.280616	0.435978
RPS10	0.295886	0.43596
PDCL	0.12394	0.435903
NA	0.471078	0.435886
MCMDC2	0.143138	0.435713
AC104794.4	0.439849	0.435672
NDUFA3	0.147601	0.435572
NA	-2.60819	0.435537
GFM2	0.119347	0.43546
TNNC2	1.631648	0.435455
MOK	0.259175	0.435437
AC116535.1	-1.05251	0.435331
ARHGEF26	-0.36639	0.435329
POSTN	-0.58534	0.435302
ZNF710-AS1	-0.24443	0.435215
NA	1.517807	0.435202
FBXO17	-0.16474	0.435103
IGF2BP3	-0.09821	0.43507
PCDHB14	-0.2999	0.435035
SYK	-0.41533	0.435029
RELN	0.496731	0.43501
STEAP2	-0.21981	0.434986
RCOR2	0.175994	0.434983
TFAP2A	-1.27208	0.434847
CLBA1	0.145627	0.434752
TTC16	0.660927	0.434694
NA	0.096986	0.434623
KLRB1	2.475657	0.434621
NA	-0.72769	0.434571

GPHN	-0.18367	0.434546
NA	-2.618	0.434534
NA	-2.618	0.434534
INAVA	-0.38903	0.434484
TPI1	0.113264	0.434317
TOP3A	-0.12152	0.434273
KRT8P12	0.225111	0.434254
FRAS1	-0.13927	0.43414
MSX1	-0.70252	0.4341
NA	0.098771	0.434072
AC002064.2	1.613032	0.434056
MXD3	0.092886	0.43405
AL109918.1	-0.17506	0.433939
PLSCR3	-0.83306	0.433865
CSNK1G3	0.120126	0.433774
PHRF1	-0.11524	0.433735
AP003392.6	1.255835	0.433718
AC132938.3	-0.4783	0.433638
NA	-0.12201	0.433625
DHX34	-0.15075	0.433539
TKT	0.108426	0.433537
YTHDF1	-0.12457	0.433503
AC093227.1	-0.31871	0.43349
SEMA7A	-0.36588	0.433461
AC233992.3	0.583406	0.433447
AC003665.1	-0.15263	0.433399
AC004918.1	-0.23003	0.433379
CCDC8	2.016635	0.43325
NA	1.048398	0.433249
KLC2	-0.14851	0.43314
AC145207.4	0.11961	0.433107
NT5E	-0.48766	0.433102
PHLDA1	0.150769	0.433097
E2F6P1	-2.44144	0.433057
RDM1P3	-2.44144	0.433057
CIPC	0.144837	0.433053
S1PR3	-0.14259	0.433007
RAD9B	-0.5786	0.432711
PRRX2-AS1	2.480574	0.432632
BRMS1L	0.167904	0.432561
RASGRP4	1.11897	0.432531
RGMA	-0.54976	0.432506
ZNF350-AS1	1.752291	0.432501
GLB1L3	-0.49389	0.432414
AL596325.2	1.001057	0.432411
PAFAH2	0.165844	0.432314
SLC30A2	1.049118	0.432272
MTERF3	-0.18328	0.432189
CBX3P9	0.823586	0.43216
AL031587.2	0.398979	0.431954

VARS2	0.128211	0.431823
ALG9	-0.28202	0.431813
TIMM8A	0.147149	0.431802
NR4A3	0.425968	0.431801
CCNB1IP1	0.106995	0.431795
ITGAL	3.074475	0.431705
RF00019	3.074475	0.431705
AL049646.1	3.074475	0.431705
ATP6V0CP1	3.074475	0.431705
H3F3BP1	3.074475	0.431705
AC016954.1	3.074475	0.431705
AL355102.5	3.074475	0.431705
MIR4677	3.074475	0.431705
RN7SL652P	3.074475	0.431705
TAF5L	0.119256	0.431665
RC3H1	-0.1125	0.431655
AC128687.2	-2.61795	0.431551
TMEM62	0.185275	0.431454
DCBLD1	-0.27314	0.431376
ABHD18	-0.1896	0.431362
MTMR12	-0.16704	0.431283
AC114284.1	0.277332	0.431283
AL390237.1	-2.6081	0.431273
NA	1.681085	0.431248
SLC8B1	-0.20367	0.431232
ZFPM2	0.90903	0.431199
HOXC9	0.287758	0.431153
P2RX6P	1.638813	0.431113
DIRAS2	0.621947	0.431067
UGT8	1.756815	0.431014
PSMA5	0.107492	0.430973
JDP2	0.225109	0.430951
MSL2	-0.13354	0.430908
KCNS1	-2.05889	0.430866
ZNF566	0.159176	0.430802
AL021878.2	1.331775	0.430745
CBR3-AS1	-0.37351	0.43072
AC027682.1	1.196446	0.4307
SMG1P1	-0.3242	0.430678
NIPAL1	0.430804	0.430657
AC007114.2	1.541019	0.430634
GALNT3	-2.44765	0.430617
ZNHIT2	0.181853	0.430572
APIP	-0.16295	0.430533
RPS2P41	2.454851	0.430473
AC008663.3	2.454851	0.430473
AREL1	-0.09869	0.430391
KIF3A	0.10741	0.430383
UBAP1	-0.1298	0.430362
SNX25	0.133193	0.430324

ATE1-AS1	-0.3683	0.43029
EEF1B2P3	0.399606	0.430281
SNRPCP3	0.964876	0.43025
AL049795.1	0.227139	0.430214
TRAPPC6A	0.204634	0.430174
POU4F1	1.662905	0.430163
USP3-AS1	0.191937	0.430136
AC009948.3	0.514384	0.429982
KLHL13	0.107569	0.429945
AC091736.1	-0.48783	0.429884
KCNF1	-0.51599	0.429864
ZNF134	-0.14333	0.429853
NA	-0.39548	0.42985
KIF5B	-0.0991	0.429831
RCE1	-0.16919	0.429811
AC079921.2	-0.54223	0.429785
TYSND1	-0.17779	0.42972
TWF1P1	-1.0546	0.429695
HSPB8	0.409477	0.42966
NA	-0.44572	0.429527
AC131235.1	0.578271	0.429512
RNH1	-0.12959	0.429356
INPP5A	-0.14276	0.429329
AC100821.1	0.60292	0.429321
AL049870.2	2.480506	0.4293
AC009093.2	2.480506	0.4293
NA	-1.66484	0.429196
SMC1A	-0.09818	0.429098
KBTBD4	-0.13095	0.429068
PLEKHA7	-0.44497	0.42901
AL136038.2	-0.26777	0.428925
AC105339.1	-1.8323	0.428907
LINC01128	-0.20984	0.428887
DDX47	0.379412	0.428755
LINC00942	-1.12916	0.428699
FKBP5	-0.13092	0.4286
AC005329.2	0.175683	0.428589
AL626787.1	-2.44144	0.428513
TPTE2P5	-0.6962	0.428485
FAM133A	0.755023	0.428481
AC008897.3	-1.82407	0.42846
AC025048.2	0.380909	0.428426
PKIA-AS1	0.977115	0.428422
SREK1IP1	-0.13352	0.42842
RPL21P121	1.905669	0.428381
AC048382.1	1.096008	0.428325
NA	-0.98559	0.428196
RNF138	-0.14266	0.428187
DIAPH3	-0.18264	0.428182
AC090985.1	-1.67159	0.428157

ERH	0.096317	0.428128
AL356356.1	0.72711	0.428081
AC010973.2	-0.11885	0.427893
NOX3	-1.39814	0.427583
ADAT2	-0.16945	0.427565
AC018635.2	-2.44766	0.427432
KAT6A	-0.10137	0.42731
LGSN	2.454743	0.427229
FAM198B-AS1	-0.57309	0.427166
RPL14	0.084263	0.427137
AC093732.2	-0.7557	0.427134
LAG3	0.392529	0.427097
SMAGP	-0.25147	0.42703
FAM220CP	2.454737	0.427028
NANOS1	0.143647	0.427001
EXO5	-0.18029	0.426964
PGM3	0.108517	0.426952
ZSWIM7	0.148484	0.426898
ZNF692	0.132868	0.426888
ZNF396	-0.35113	0.426782
UBE2J1	0.103814	0.42675
WFIKKN1	0.651551	0.426732
AL132655.2	-0.44011	0.426704
UBALD1	0.132815	0.426672
AC004691.1	2.024293	0.426627
OGFRL1	-0.26873	0.42653
MAD2L1BP	-0.15723	0.426502
OAF	-0.22122	0.426474
WHRN	-0.4328	0.426352
CDKL2	-0.87465	0.426199
PRSS12	-0.26712	0.426063
NA	0.114338	0.426018
ZNF799	-0.32285	0.425802
NA	-1.25809	0.425661
GBP3	-0.40129	0.425583
AKR1C1	-0.2759	0.425457
RTCB	0.110459	0.425433
CNGA4	-2.44144	0.425328
PACS2	-0.13203	0.425287
AC091212.1	-1.4409	0.425225
ADH5	0.10349	0.425203
PAQR8	-0.14853	0.425116
ABCF2	-0.09721	0.424977
EMC4	0.10985	0.424944
RAPH1	-0.15403	0.424929
ANKFY1	-0.1154	0.424913
AC024940.2	-0.50927	0.424863
AC007619.1	1.642196	0.424747
NA	-0.84415	0.424694
TTLL12	-0.10638	0.42466

AC068888.1	0.111372	0.424599
MIR34AHG	-1.34952	0.424519
KCTD14	-1.41393	0.424451
NPM1P26	-0.94521	0.424342
IFT43	0.164975	0.424299
AC006116.5	2.097186	0.424164
Z84492.1	0.227355	0.424124
TPST1	0.153868	0.424095
SRSF4	0.09856	0.424072
RNU6ATAC16P	1.921502	0.423872
AC013275.1	2.106025	0.423852
LHFPL2	-0.09544	0.423776
TCEAL3	0.187109	0.423758
IDH2	0.090279	0.42374
NA	-0.25217	0.423715
AC011247.1	-1.17731	0.423602
AL928970.1	0.758818	0.423565
SLC25A28	0.16755	0.423539
TCFL5	0.162393	0.423498
AP002884.2	2.005044	0.423338
CD207	0.686993	0.423292
AC016738.1	0.352519	0.423202
ERICH6	0.838857	0.423143
CMKLR1	-0.54362	0.423102
TTLL13P	2.898171	0.42306
TERF1P4	2.898171	0.42306
AC099535.1	2.898171	0.42306
BDH2	0.174784	0.423025
KIFC3	-0.14999	0.422996
BCL9	-0.12119	0.422896
BUD31	0.101696	0.422787
NAA60	-0.35858	0.422778
MGST2	-0.26819	0.42275
SPSB3	0.141424	0.42271
PCNX1	0.099582	0.42254
SORT1	-0.11298	0.422429
ADSS	0.108888	0.422416
GJA9	1.723359	0.42239
PLK3	-0.1764	0.422308
NA	3.203981	0.42226
CNGB1	3.203981	0.42226
AC069218.1	3.203981	0.42226
AC025262.1	3.203981	0.42226
NA	3.203981	0.42226
LINC01201	3.203981	0.42226
MTND5P8	3.203981	0.42226
RPL31P50	3.203981	0.42226
AL731563.1	3.203981	0.42226
NA	3.203981	0.42226
AC120036.1	3.203981	0.42226

AL096869.1	3.203981	0.42226
NA	-0.85398	0.422116
CCR10	0.379325	0.422046
AP005482.3	-0.5399	0.421983
NA	0.474288	0.421981
PPIL4	-0.12884	0.421831
RF00019	1.495497	0.421784
PPAT	0.118382	0.421592
NPTX2	-0.16593	0.421584
RHBG	0.503677	0.42153
OSBP	0.123691	0.42151
RF00432	3.038049	0.42147
GLIPR2	0.149584	0.421419
MAN1C1	-0.28466	0.421399
GTF3C6	0.113614	0.421313
CABP1	0.619835	0.421308
MRPS11	0.126184	0.421253
TUBD1	0.195069	0.421162
TINAGL1	1.885853	0.42107
NPIPB11	-0.41433	0.421049
TOLLIP	0.115308	0.421006
AC074044.1	0.271685	0.420903
MAMDC2-AS1	1.36436	0.420708
CCDC78	-0.20001	0.420661
GDF11	0.147153	0.420649
ARHGEF7	-0.09445	0.420563
DUOXA1	-0.65242	0.420486
C17orf51	-0.32002	0.420473
PURG	0.211578	0.420446
NUP50-DT	0.250601	0.420366
NDP-AS1	-1.39086	0.420362
HSCB	0.20293	0.42032
TXNL4A	0.104344	0.420299
AC130469.1	-2.42247	0.42022
AL356019.2	0.4829	0.420032
MYPOP	-0.20253	0.419987
PEX26	0.101679	0.419978
PPP1R3D	-0.26576	0.419954
COPS8P2	1.385108	0.419885
NLGN4X	-0.15184	0.419834
LINC01405	1.332476	0.419811
CIC	-0.14877	0.419686
HMBS	-0.12936	0.419649
MRPL51	0.117	0.419641
MOV10L1	0.804168	0.419632
KIF6	-0.55831	0.419502
HPS5	-0.14632	0.419447
AC093849.1	1.162938	0.419303
COX7C	0.121273	0.419217
ESRRA	0.112438	0.419177

OGG1	0.122255	0.419005
CORO1A	0.220411	0.418927
PASK	-0.14852	0.418905
TMEM241	-0.17069	0.418859
PEBP1	0.111251	0.4188
NOTCH3	-0.18317	0.418792
GOLGA6L4	0.904169	0.418736
NFU1	0.176763	0.418706
RERE	-0.17887	0.418655
TOP3B	0.122411	0.418538
NA	-0.21804	0.418519
TATDN3	-0.14939	0.41851
ZFP90	0.112003	0.41849
SNX18P7	-0.64926	0.41832
NA	-0.28691	0.418221
AC004816.1	0.431984	0.418214
TIAF1	-0.59459	0.418174
CNIH2	0.154301	0.418158
LINC01473	0.908369	0.418144
IGHV1-46	0.843808	0.418081
METTL26	0.136759	0.41806
SAMD10	-0.20424	0.418006
RNF122	0.208567	0.417926
AC004057.1	0.328168	0.417801
ZBED6	-0.15692	0.417689
GPRACR	-2.41457	0.417632
AC008750.7	-2.41457	0.417632
IGFN1	-0.38621	0.417609
HIST4H4	-0.43735	0.417575
PCDHGA7	0.231614	0.417527
ARHGEF17	-0.13971	0.417512
AL136366.1	-0.6062	0.417352
GLRX3	0.115958	0.417347
AL645568.1	0.494866	0.417324
CASC4	-0.10711	0.417262
SLC34A3	0.411841	0.417241
AC112211.1	0.208914	0.417196
PCDHGA5	-0.95191	0.417169
TGFB1I1	0.170413	0.417149
MIR3186	-2.39383	0.41705
AL158050.1	2.295332	0.416986
RPS27AP13	2.295332	0.416986
SMOX	0.30801	0.416913
DNAH1	0.295672	0.416908
NRCAM	-0.14581	0.41684
TRIM26BP	0.88878	0.416834
RUSC1	-0.11263	0.416789
RNU6-415P	-1.37738	0.416712
CACNA1C-AS2	1.105774	0.416673
RF00190	-1.87822	0.416612

FTH1P23	2.238879	0.416606
CHPF2	-0.11252	0.416459
LYAR	-0.13537	0.416357
TAT	-2.54658	0.416353
RNU2-33P	-2.54658	0.416353
ZSCAN25	0.125022	0.416279
ACTG1P14	-2.13498	0.416205
ZNF93	0.191549	0.416198
AC021321.1	0.847165	0.416134
AC245052.3	1.639168	0.416129
PRDX5	0.092092	0.416076
DDX27	0.093803	0.415969
ARNTL2-AS1	-0.56626	0.415951
AKAP8L	0.119718	0.415914
NA	-0.77432	0.415894
TMEM37	0.975638	0.415818
CADM4	0.173685	0.415761
MT-TS1	-0.12099	0.415752
SNORD14A	0.597067	0.415708
ZFP69B	0.195272	0.415687
CYP27C1	-0.62392	0.415665
C7orf25	-0.48003	0.415641
SLC35E1	-0.10407	0.415631
SLC28A2	1.271815	0.415585
PCF11-AS1	-0.32344	0.415553
LINC01266	-1.10558	0.415485
RBFADN	-0.41143	0.415481
PRCP	-0.10619	0.415443
NA	-0.55151	0.41544
RPS3AP6	0.264723	0.415345
ARMCX4	-0.15127	0.415191
GREM1	-0.14665	0.41506
AL356475.1	-0.32566	0.415023
TTN-AS1	0.143125	0.414937
SNRPFP1	1.775999	0.414916
AC097534.2	0.326588	0.414893
CLSTN1	-0.11534	0.414875
NA	-0.33108	0.414782
STAP2	-0.27568	0.414777
AC243562.2	-0.3736	0.41463
ROM1	-0.21994	0.414616
AC000123.1	0.389879	0.414536
AC015961.1	-1.04918	0.414485
PCDHA11	-0.38984	0.414463
GLG1	-0.09466	0.414462
AC009133.2	0.130733	0.414458
SLC7A2	-0.13478	0.414434
NA	-0.47051	0.414414
VSTM5	0.830725	0.414296
AC016596.2	0.348032	0.414235

OSBPL11	-0.13853	0.414172
PGBD5	-0.15871	0.414035
TRAF5	0.125729	0.414029
RNU7-3P	2.026324	0.413994
AC093591.2	-1.0172	0.41399
LRRC7	-0.42151	0.413935
NCAPD3	-0.13277	0.413861
AP000866.5	-0.43742	0.413766
B4GALT4-AS1	0.345604	0.413733
TTPAL	-0.12041	0.413725
ACO1	-0.12968	0.413724
AC010649.1	0.733746	0.413707
TMEM154	-0.49171	0.413679
RTN4IP1	-0.24455	0.413618
DHH	-1.76592	0.413535
RNF151	-1.17802	0.413494
TMEM191C	0.697391	0.413489
AL391811.1	1.75724	0.413327
PBRM1	-0.09228	0.41332
SPATA6L	0.252938	0.413316
TRAF6P1	1.967082	0.413153
CD9	0.141313	0.413134
SYNPO2L	0.473252	0.413073
CTSF	-0.12886	0.413055
CNPPD1	0.115659	0.413049
TRIM11	-0.12844	0.413038
C11orf96	0.31322	0.413036
AC093157.1	0.19329	0.413032
RSBN1	-0.13483	0.413021
MIR4458HG	0.227629	0.412987
AC012618.1	1.784887	0.412979
TBCK	0.138057	0.412963
LINS1	0.170813	0.412926
GABRB3	-0.1155	0.412859
HDDC3	0.223294	0.412845
PTPRCAP	0.46722	0.412841
AC006518.1	-0.96324	0.412755
AC098847.1	-2.4224	0.412728
ACAD10	-0.15452	0.412717
NA	0.544317	0.412679
NA	-0.17632	0.412582
AL133523.1	0.764548	0.412558
HSPA8P9	-2.39371	0.412498
AC100826.1	-2.39371	0.412498
PTMAP5	0.367538	0.41249
VWCE	-0.28915	0.41242
AC079880.1	0.228696	0.412392
AC116914.2	-0.47979	0.412371
RWDD2A	0.188839	0.412364
TMEM233	1.602765	0.412311

KHDRBS1	-0.09668	0.412298
GALNT15	-2.54676	0.412237
NA	-2.54676	0.412237
IFIT1	-0.4799	0.412193
ZNF10	-0.16486	0.412189
AC010605.1	-0.19429	0.412089
C1QL4	0.488603	0.412072
RNASEH1	0.101742	0.411961
C11orf21	-1.05209	0.411943
FAM20B	-0.10945	0.411888
ABCB6	-0.30098	0.411877
CCL8	-2.84709	0.41187
TNNC1	-2.84709	0.41187
BMPR1AP2	-2.84709	0.41187
DUSP16	0.104664	0.411844
AC087362.1	-0.32596	0.411843
IL17RA	-1.27749	0.411768
NEFH	-0.15192	0.411757
AC026740.1	-0.86968	0.411689
AC106897.1	-1.63838	0.411675
TMEM86B	-0.26039	0.411653
AC011997.1	1.960135	0.411608
RANBP9	-0.13226	0.411543
CAV1	-0.841	0.411517
LCK	2.564091	0.411494
EDNRB-AS1	2.564091	0.411494
TYMP	0.369139	0.411445
AC073114.1	-2.3905	0.41144
GCGR	-2.39527	0.41132
AL031710.1	-2.39527	0.41132
LINC01864	-2.39527	0.41132
AC024075.3	0.285141	0.411308
GJB3	2.564079	0.411307
AC116609.3	2.564079	0.411307
FAM177A1	-0.16262	0.411288
PLXDC2	0.18354	0.41127
CA12	-0.28903	0.411194
RN7SL832P	-0.86055	0.411131
TTC13	0.127916	0.411092
AL353588.1	-0.80605	0.411053
LAT	1.220246	0.410979
RPS3AP34	1.506376	0.410967
ADH6	-0.6966	0.410954
C17orf97	-0.40074	0.410939
AC079385.1	0.296626	0.410928
AC002558.1	1.616599	0.410921
ERICH3	-0.21703	0.410881
EIPR1	0.10456	0.410839
SINHCAF	0.094337	0.410772
COX7B	0.125888	0.410591

RPL3P4	0.35096	0.410544
TSPO2	1.947703	0.410532
RNU2-11P	1.317279	0.410531
AC010536.2	0.930546	0.410499
BCRP3	1.061769	0.410454
DYNLRB2	2.86002	0.41044
LINC01107	2.86002	0.41044
Z69666.1	2.86002	0.41044
STIM2-AS1	2.86002	0.41044
AC073352.2	2.86002	0.41044
XKR6	0.232408	0.410427
AC133550.2	-0.67135	0.410419
AC004540.2	0.546451	0.410413
SCGB1B2P	-1.40789	0.410367
ETNK2	0.183772	0.410361
PCNPP5	2.412297	0.410256
FAM83H	2.412297	0.410256
KCNT1	-0.20254	0.410256
INAFM1	0.209603	0.410208
ZDHHC20	0.16565	0.410166
AP006259.1	3.002772	0.410158
MIR4502	3.002772	0.410158
TUBG2	-0.19633	0.410152
LGALS2	-2.41447	0.410139
AC004690.2	-2.41447	0.410139
SVIL	-0.13789	0.410098
MOB1A	-0.1011	0.410098
AC025259.3	0.326371	0.41007
AGFG2	-0.1737	0.410028
ADA	0.212084	0.409919
CHFR	0.115076	0.40988
AC010210.1	1.694434	0.409837
AL356968.2	1.957334	0.409734
C8orf49	-0.45779	0.409639
UBE2L3	-0.10436	0.409622
UNC50	0.126478	0.40957
AC018410.2	-1.01998	0.409563
LINC01104	-2.54688	0.409355
ZNF101P2	2.401581	0.409317
LINC01291	0.329989	0.409232
SMG7	-0.08978	0.409177
FCHO2	-0.17502	0.409167
API5	-0.10028	0.409058
GHRL	1.092698	0.409041
ZNF140	-0.14044	0.409025
DDX12P	0.133505	0.409
AL807752.5	-1.2258	0.408921
NA	-1.85604	0.408706
TLN1	-0.0921	0.408706
TAF1C	-0.12418	0.408697

KMT2E-AS1	0.298242	0.408681
CIP2A	0.151394	0.408591
AC005070.3	-0.30479	0.408538
TUFMP1	0.568988	0.408426
MRPL13	-0.13865	0.408424
RALY	0.090739	0.40837
APOBEC3C	-0.1752	0.408354
CYREN	0.106829	0.408334
AC093014.1	2.403771	0.408328
AL160270.1	0.391438	0.408236
DENND4C	0.108863	0.408234
EFCAB13	0.553278	0.408232
AC018638.1	0.38602	0.408173
PEAK1	-0.12037	0.408159
FURIN	-0.13156	0.408118
AC016590.1	-1.04636	0.408108
CPT1B	0.545351	0.408062
SUDS3	0.101618	0.40804
PRSS8	-1.62585	0.408034
AF064860.2	1.972421	0.408031
PXDNL	-0.65236	0.408012
AC010327.1	0.405787	0.407982
GPR37L1	0.277914	0.407961
ADAM17	0.086829	0.407858
NLGN2	-0.12657	0.407854
ZNF521	0.215363	0.407845
AMN	-0.17134	0.40782
TMA7	0.11847	0.407792
MOB3C	0.185713	0.407754
AC012378.1	0.841786	0.407653
PLAGL1	0.291318	0.407651
RNU6-930P	-1.8904	0.407505
AC103740.2	-1.8904	0.407505
CNKSR1	0.571472	0.407472
CD8A	-0.41126	0.407425
PPP1R14BP3	-0.52391	0.407356
MASP2	0.315348	0.407272
TRAPPC1	-0.13075	0.40725
EIF5A2	0.206402	0.407208
LUZP1	-0.12273	0.407179
SNRPGP10	0.380771	0.407164
AC093567.1	-1.4149	0.407117
TNPO2	-0.10612	0.407082
PNISR	0.09724	0.407064
CYP17A1	2.412132	0.406924
AC025271.2	2.412132	0.406924
PPP1R2	-0.13335	0.406911
RIPOR1	-0.1416	0.406861
SMIM13	-0.12615	0.40685
AC010761.5	-0.19995	0.4068

CDK4	0.088423	0.406635
KLHDC8B	0.158503	0.406598
MED30	0.180801	0.406598
SEC23B	0.088221	0.406583
H2AFVP1	0.987781	0.406572
AMDHD2	-0.16543	0.406512
FAM71D	1.946874	0.406496
CRIM1	-0.60791	0.406396
SLC44A3	-0.3259	0.406314
KCTD10	-0.10519	0.406278
UGGT2	0.124807	0.406175
FAM126A	0.633196	0.406171
AC027020.2	-0.49287	0.406122
MFSD4B	0.1525	0.406111
AC010834.3	-0.3318	0.406063
SLC46A3	0.59856	0.406052
SLC27A1	-0.19118	0.405935
ACTG1P20	0.603423	0.405933
RTEL1	-0.67778	0.405932
SNX27	-0.11187	0.405912
AC084262.1	-1.06871	0.405891
WDR54	0.134981	0.405852
ACSM3	0.223771	0.405797
NA	-0.4953	0.405731
INTS1	-0.11262	0.405607
USE1	0.144458	0.405604
RAB3A	-0.23098	0.405569
DAAM2	0.317339	0.40555
RPL10P16	0.431157	0.405436
AL049873.2	1.931719	0.405435
UBAC1	-0.09658	0.405404
BRINP2	2.404024	0.405364
RPL34P33	2.404024	0.405364
SMARCA5-AS1	0.163211	0.405357
RNU2-59P	-1.94344	0.405347
ASB14	0.262467	0.405281
AC087289.3	0.223733	0.405277
KRT18P7	-1.74207	0.405223
CYP19A1	2.40404	0.405181
AC078817.1	2.40404	0.405181
C2orf69P1	2.40404	0.405181
AC004771.2	-0.35196	0.405034
NAMPTP1	0.889759	0.40488
NA	1.965508	0.404858
AP006621.4	1.965508	0.404858
NME9	0.590132	0.404854
LYN	-0.09534	0.404836
SF3B2	-0.09224	0.404788
HACE1	0.147779	0.404758
LRRC42	-0.11641	0.404749

IL18R1	-1.05713	0.404745
PRMT5-AS1	-0.10928	0.404697
RNU6-431P	1.41774	0.404673
TUBA3E	-0.98961	0.404669
AC123768.1	1.928289	0.404667
RAB17	-2.0779	0.404629
AC008163.1	-2.0779	0.404629
AC107223.1	1.736792	0.404629
ZNF408	0.18418	0.404606
RPS15A	0.127346	0.404344
AC009093.4	-1.28476	0.404291
NGDN	0.118521	0.404266
RN7SL396P	2.266626	0.40424
ASNSP1	2.266626	0.40424
FAM185A	-0.18459	0.404186
AL359399.1	0.463957	0.404143
AC084809.1	0.504677	0.404137
IL21R	1.298444	0.404071
NA	2.266629	0.404044
STX4	0.111462	0.40402
PCDHB9	-0.25386	0.403969
CACNA1C-AS1	-0.62173	0.403938
SLC12A4	-0.14104	0.403925
AC025159.1	0.291599	0.403915
RPS6	0.091494	0.403823
AC107982.3	-2.39508	0.403821
ANKRD23	-0.25186	0.403815
PRX	0.29756	0.403785
SMIM12	0.135824	0.40371
CTSE	0.630679	0.403687
FIGNL2	0.423702	0.403607
ZFP64	-0.15908	0.403544
WASF2	-0.10475	0.403476
GNRH1	-0.38202	0.403467
TLK2P1	-1.02402	0.403448
YIF1B	0.11745	0.403434
AC008982.2	-0.46796	0.403419
METAP1D	-0.17098	0.403408
MIR4479	1.658534	0.403383
MEF2C	-0.20154	0.403334
GPRC5D-AS1	0.35952	0.403334
ZNF225	0.234141	0.403301
AP1G2	0.118615	0.403275
FAM222B	-0.15848	0.403162
ARMCX3	0.118309	0.403092
AP000223.1	-1.8537	0.403073
NA	-1.61776	0.402969
RPL41P1	0.293429	0.402944
CLCNKB	1.482943	0.402917
AC024243.1	-0.97309	0.402898

INTU	-0.16735	0.40285
ST7	0.161878	0.402714
EPS8L1	-0.24795	0.402622
CAMK2N1	-0.13826	0.402591
EAF1-AS1	0.190477	0.402571
FOSB	-1.1681	0.402547
AC126696.2	0.309181	0.402547
EMC3-AS1	0.241106	0.402484
CEP112	0.230298	0.402348
AC007743.1	-0.4933	0.402316
IRF2	0.141216	0.402229
ENO3	0.088574	0.402189
MBTPS1	-0.09877	0.402144
AC010226.1	0.170532	0.402117
NA	-1.41397	0.402098
GABARAP	0.233325	0.402078
FAM71F2	-0.8506	0.402046
AC005828.4	-0.48642	0.401739
PRPS1	0.106435	0.401673
MAPK6-DT	-1.75347	0.401655
PHKB	0.121987	0.401581
PPP3CC	0.233284	0.401428
SPOUT1	-0.10473	0.40141
NA	0.238409	0.401408
AC021660.2	0.478862	0.401247
ECPAS	0.090726	0.401215
RERG	1.054455	0.40121
PDZD8	-0.13561	0.40115
FAM83E	-1.37388	0.401112
GBP5	-0.77827	0.401013
AC040970.1	-0.72049	0.401005
AC115989.1	-1.559	0.400973
PAPPA-AS2	-1.23721	0.400954
IER3IP1	-0.17298	0.400922
ACCSL	2.364812	0.400898
AL590002.1	2.364812	0.400898
RIPPLY2	0.24569	0.400876
AC134349.1	0.390147	0.400851
MIR124-2HG	0.670598	0.400704
PJVK	-0.44653	0.400662
FAM155A-IT1	-1.34517	0.400646
GEMIN5	-0.11849	0.400593
MAPT-AS1	0.554313	0.400536
CDH15	0.769734	0.400526
FBXO11	-0.10024	0.4005
EEF2KMT	0.131941	0.400472
FUOM	0.177806	0.400439
AC096720.1	1.189933	0.40038
EPHA1-AS1	-1.26325	0.400263
TMEM253	0.540112	0.400257

NOP9	-0.10217	0.400229
MRPL20	0.121722	0.400225
NA	1.14237	0.400225
PATJ	0.216908	0.400206
GGA2	0.094303	0.400155
PCIF1	-0.13855	0.40012
AC104187.1	0.45677	0.400118
ATXN2	-0.1249	0.400012
SNHG14	-0.10873	0.399971
ZBTB8A	0.208457	0.399944
CHRNG	1.363978	0.399941
MTFR1L	-0.11369	0.39985
RF00019	0.539702	0.399758
NA	1.809206	0.399718
WNT6	0.33204	0.399717
MIR4665	0.840561	0.399668
EIF4A1P1	2.376316	0.399636
RPL10P19	2.376316	0.399636
PLAAT4	0.583264	0.399614
RUNX3	-2.80955	0.399523
RN7SL566P	-2.80955	0.399523
SEH1L	0.131519	0.399444
SCYL2	0.101359	0.39926
ATP5F1C	0.112373	0.399224
AC073896.5	-0.25504	0.399177
EPM2A	-0.18464	0.399172
TMEM64	-0.12682	0.399112
A2ML1-AS1	0.375148	0.39909
DAPK3	0.133523	0.399018
AC027559.1	0.512895	0.398969
NA	1.946167	0.398942
ZNF556	0.673019	0.398909
TRMT1	0.110733	0.398784
GCM2	-2.80719	0.39876
KRT17P8	-2.80719	0.39876
RS1	-1.31077	0.398747
GBP2	0.178875	0.398714
TMEM189	-0.19635	0.398709
AGAP9	-1.22114	0.398672
SLC7A11-AS1	1.038924	0.398667
CREB5	0.148076	0.398619
NA	-1.48556	0.398617
ENKD1	0.144888	0.398565
PIK3CD-AS2	-0.2904	0.398547
FMN1	0.165757	0.398516
VSIR	-0.55045	0.398514
MFSD4A	0.507616	0.398482
AC092849.2	0.209432	0.398466
MYH9	0.120742	0.398465
C12orf42	1.978388	0.398463

AC124068.1	-0.17595	0.39843
RRM2P3	-1.59315	0.398314
BFAR	0.091276	0.398295
TRAPPC12	-0.10228	0.398279
RNU6-100P	1.59644	0.398252
AC013553.1	1.842879	0.398166
GNB5	-0.14289	0.398166
MID1IP1-AS1	-0.55618	0.398163
RNU6-510P	-1.01532	0.398134
AC036176.2	-1.93918	0.398077
IMPDH1P5	0.628586	0.398068
NRAS	-0.09644	0.398067
SH3BGRL	-0.10511	0.397984
MPPED1	-1.31628	0.39793
NA	2.374103	0.397885
AC078852.1	2.374103	0.397885
RIT2	0.720654	0.397883
AL158198.1	-1.26227	0.397781
PPP4C	0.090353	0.397706
SF3B3	-0.07868	0.397696
AL031847.1	-0.66963	0.397684
SNORD94	0.826763	0.397684
EXOSC2	0.098108	0.397649
AC012170.3	-0.27679	0.397551
AC111182.1	0.658111	0.39754
FAM124A	-0.21036	0.397527
RFXAP	-0.1727	0.397448
RPL19	0.094733	0.3974
WDR73	0.103267	0.397343
WDR20	-0.12528	0.397249
TACR2	0.884117	0.397171
AC003112.1	0.163894	0.397149
AL353622.2	-1.81835	0.397088
NA	-0.83974	0.397019
AC012404.1	2.505693	0.396895
AL121758.1	2.505693	0.396895
MRPL33	0.135043	0.396885
WNT7B	-1.21588	0.396775
QPRT	0.100757	0.396744
UBA7	-0.24181	0.396723
AP003071.1	1.287973	0.396691
PLA2G10	2.376541	0.396673
AP001372.1	2.376541	0.396673
AP002373.2	2.376541	0.396673
AC016876.2	2.376541	0.396673
AC079610.2	2.376541	0.396673
CT66	-0.56647	0.396605
ACADSB	-0.13803	0.396587
ZNF654	-0.16188	0.396546
AC011611.3	0.20833	0.396537

ACAT2	0.103336	0.396528
LPP	-0.69506	0.396523
VSIG2	1.301219	0.396511
NCF4	2.376554	0.396491
RPL21P65	2.376554	0.396491
AL592182.1	2.376554	0.396491
SCARNA5	2.376554	0.396491
AC090844.2	2.376554	0.396491
CNDP2	-0.11232	0.396354
AC009065.2	0.205852	0.396308
MYLK2	-0.81481	0.396154
CADM3-AS1	0.289264	0.396137
HMGXB3	-0.10648	0.396085
MIR4656	-2.043	0.395956
AL450998.1	1.552373	0.395944
RYR3	-0.27491	0.395938
NR6A1	-0.30377	0.395825
MINAR2	0.562155	0.395816
AL158163.1	0.530869	0.395801
AC083799.1	0.384202	0.395711
AC012645.1	-0.62702	0.395532
MPRIPP1	0.424022	0.395511
SERPINB9P1	-1.18987	0.395375
TMEM132E	1.518146	0.395356
HOGA1	0.756879	0.395341
TUSC1	0.148444	0.395321
NA	0.234179	0.395317
AC026904.3	1.914367	0.395301
NA	1.095558	0.395165
AC068870.1	-1.60197	0.395159
ZNF32	0.162195	0.395155
RSL24D1P3	-1.58185	0.395153
IL10RB	-0.23985	0.395083
CCDC141	1.761175	0.395078
ARID4A	0.13494	0.394921
NANP	-0.18847	0.394907
AP001972.3	-1.25135	0.394816
MST1P2	0.30376	0.394792
DDX41	0.098076	0.394783
KCNN3	-0.74265	0.394744
UBA6	-0.10629	0.39457
TPTE2P6	2.373849	0.394546
AC245452.2	2.373849	0.394546
AC139103.1	2.373849	0.394546
RPL18AP7	-1.81663	0.394531
ZNF300	0.113043	0.394516
LZTS2	0.100837	0.39445
FAHD2A	0.18678	0.394441
AC020898.1	0.986975	0.394441
MYRIP	-0.15735	0.394435

MRPS34	0.09051	0.39443
CAPN13	-0.95274	0.394372
LINC01560	-0.34519	0.3943
MEX3A	-0.09268	0.39411
UQCRC2	0.086959	0.394096
AP001931.1	-1.59902	0.394027
HAND2	0.090597	0.394014
RASGRP3	1.45261	0.393996
DOK5	-0.68484	0.393984
ZG16B	1.25042	0.393975
NA	2.505668	0.393968
RPL23AP14	2.505668	0.393968
PCBP3	-0.13339	0.393958
ARL17B	0.93721	0.393853
DAZAP2	0.089774	0.393803
LIPT1P1	2.505666	0.393788
AC080037.1	2.505666	0.393788
ZNF782	-0.1995	0.393741
AC010900.1	-1.41873	0.393734
TMEM229B	0.710595	0.393661
ENOPH1	0.109042	0.39354
NA	0.335975	0.393498
TIMM10	0.115096	0.393478
NA	-0.26111	0.393471
MYMK	-1.68563	0.393468
RAC3	0.121228	0.393464
MISP	-3.09351	0.393365
HHLA2	-3.09351	0.393365
CER1	-3.09351	0.393365
HIST2H2AB	-3.09351	0.393365
CGB7	-3.09351	0.393365
AC116351.1	-3.09351	0.393365
ARMC8P1	-3.09351	0.393365
AL020997.1	-3.09351	0.393365
LINC02005	-3.09351	0.393365
AL445430.2	-3.09351	0.393365
AL512604.3	-3.09351	0.393365
WDR17	-0.18612	0.393337
SEMA3B-AS1	0.763936	0.393264
AL031714.1	-0.11634	0.39319
CLEC18C	-1.35866	0.393142
POU5F1P6	1.901847	0.393125
C6orf163	-0.93889	0.393124
HAGH	0.1185	0.392999
C12orf66	0.234849	0.392938
ZHX1-C8orf76	-1.15763	0.392933
PLEKHG6	-1.32161	0.392904
AC012613.2	-1.27633	0.392901
SIGLEC10	0.946027	0.392875
AL360270.1	0.271627	0.392823

HSD17B7	0.176135	0.392813
AC073934.1	2.064378	0.392742
ZNF641	-0.15038	0.392691
BIRC6-AS1	0.565891	0.392685
CFAP20	0.1229	0.39265
CEP19	0.238768	0.392565
DIRAS3	0.979246	0.392563
CEP76	-0.15107	0.392497
RFX7	-0.10272	0.392444
ART4	0.908422	0.39235
AL157394.1	1.381375	0.39233
MANBA	-0.17698	0.392311
SHC1	-0.07582	0.392301
SPTSSA	0.122222	0.392212
KIAA0408	0.477374	0.392188
POLR2J3	-0.18528	0.392132
TRIM62	-0.26298	0.392125
AC007182.1	-1.04218	0.392036
PPP1R1B	-0.82208	0.392003
OAT	-0.10276	0.391891
NHS	0.488538	0.391857
AC005822.1	1.129256	0.391847
AL391863.1	0.228867	0.391799
NUDT8	-0.35472	0.391795
NEURL2	0.284621	0.391775
AC008734.1	-0.36411	0.391722
CYP2J2	0.30755	0.391704
NHSL2	-0.2524	0.391701
RTL10	-0.15194	0.391673
NR2C1	0.110264	0.391589
AL050403.2	-1.17839	0.391579
APLF	-0.25299	0.391557
RAPGEF1	-0.12983	0.391542
AL354707.3	-1.81851	0.391372
IGFBPL1	0.112627	0.391363
DCN	0.491246	0.391344
GDAP2	0.125005	0.391332
MGAT2	0.115789	0.391288
ZNF254	-0.13599	0.391269
GPN2	0.104896	0.391235
PJA1	0.114072	0.391232
GATAD2B	-0.11377	0.391196
HSPB6	-1.28332	0.391104
NA	0.197655	0.391082
LINC01399	0.633102	0.391023
RPL14P1	0.395611	0.391021
UNC80	0.154889	0.390981
MTCO3P12	0.423002	0.390959
SFTPB	2.012407	0.390949
NDUFAF1	-0.16578	0.390943

KLHL31	-0.56936	0.390787
RABL2A	0.183163	0.390772
LMAN1	0.107203	0.390673
SCN1A	0.503892	0.39066
ARMC6	0.113797	0.390595
MT-ND4L	0.101842	0.390591
OSTM1	0.139521	0.390525
YIPF3	0.09473	0.390495
ALG14	0.273853	0.390492
AL603756.1	0.178925	0.390485
ETV3	-0.0963	0.390436
MXRA7	-0.09333	0.3904
FBXL14	0.169384	0.390399
LRIG3	-3.08343	0.3903
RNU6-1123P	-3.08343	0.3903
RF00019	-3.08343	0.3903
IFI30	-3.08343	0.3903
VPS26BP1	-3.08343	0.3903
AC131571.1	-3.08343	0.3903
AL691426.1	-3.08343	0.3903
AC107081.1	-3.08343	0.3903
AL591438.2	-3.08343	0.3903
NA	-3.08343	0.3903
AC010894.3	-3.08343	0.3903
PPM1AP1	-3.08343	0.3903
LINC02287	-3.08343	0.3903
NA	-3.08343	0.3903
AC005696.2	-0.1344	0.390277
EVI5L	0.204711	0.390276
ABI1	0.134048	0.390256
PTPN23	-0.15973	0.390238
KRT7-AS	1.194548	0.390234
SOS1-IT1	-0.29436	0.390182
PHF3	0.102292	0.390137
GPATCH2L	0.095592	0.390131
ATP6V1F	0.103907	0.390116
GUSB	-0.12762	0.390028
NA	1.654595	0.39001
YRDC	0.112773	0.389766
AC025857.2	-1.61753	0.389742
TPD52	-0.19476	0.38973
ITGB3	-0.44899	0.389715
C10orf95	0.337039	0.389683
AC008781.1	-0.18856	0.389597
P3H4	-0.12562	0.389592
AC012442.2	0.995204	0.389554
BICRA	-0.17767	0.389517
AC003072.1	0.550308	0.389486
AL359198.1	-0.5195	0.389477
RAB13	0.087312	0.389474

XRN2	-0.08018	0.389426
RSRC2	0.093954	0.389419
AC012513.1	-0.46401	0.389349
AC009065.7	0.203959	0.389258
MRVI1-AS1	0.625337	0.389173
PDE3B	-0.13471	0.389164
TGM1	-0.41818	0.389144
LIMK1	-0.12604	0.389015
TMEM175	-0.17521	0.389004
CYCSP10	-0.65922	0.38894
NA	-1.29907	0.38885
DTWD2	0.206182	0.38876
AP001439.1	2.93617	0.388758
THAP12P1	2.93617	0.388758
FTLP2	2.93617	0.388758
AC098859.1	2.93617	0.388758
AC005828.2	2.93617	0.388758
HGD	1.575907	0.388757
GABRE	3.089845	0.388653
CCDC144B	3.089845	0.388653
TDRD10	3.089845	0.388653
PRKCB	3.089845	0.388653
SLC38A11	3.089845	0.388653
RPRM	3.089845	0.388653
NA	3.089845	0.388653
BEX5	3.089845	0.388653
ZNF679	3.089845	0.388653
AP003027.1	3.089845	0.388653
RF00017	3.089845	0.388653
LINC01602	3.089845	0.388653
RF00019	3.089845	0.388653
MIR558	3.089845	0.388653
GAPDHP44	3.089845	0.388653
KRT8P41	3.089845	0.388653
AC073310.1	3.089845	0.388653
NEPNP	3.089845	0.388653
AL357054.1	3.089845	0.388653
NA	3.089845	0.388653
MIR1260A	3.089845	0.388653
AP001341.1	3.089845	0.388653
NA	3.089845	0.388653
AL391244.1	3.089845	0.388653
HAR1A	3.089845	0.388653
MTND4P32	3.089845	0.388653
KIF4B	3.089845	0.388653
RPL35AP3	3.089845	0.388653
AL451074.4	3.089845	0.388653
NA	3.089845	0.388653
LINC01273	3.089845	0.388653
LINC00708	3.089845	0.388653

POLHP1	3.089845	0.388653
LINC01135	3.089845	0.388653
RPL7L1P3	3.089845	0.388653
COX5BP3	3.089845	0.388653
RF00019	3.089845	0.388653
SCARNA7	3.089845	0.388653
SNORD13D	3.089845	0.388653
NA	3.089845	0.388653
RN7SL745P	3.089845	0.388653
RPS27AP9	3.089845	0.388653
ST13P15	3.089845	0.388653
CRYZP2	3.089845	0.388653
AC017007.5	3.089845	0.388653
NA	3.089845	0.388653
ZNF317P1	3.089845	0.388653
AP001783.1	3.089845	0.388653
AC090673.1	3.089845	0.388653
NA	3.089845	0.388653
AC027544.3	3.089845	0.388653
AP000851.1	3.089845	0.388653
AC125611.1	3.089845	0.388653
AC091544.2	3.089845	0.388653
BMS1P16	3.089845	0.388653
AC114546.1	3.089845	0.388653
AC022960.1	3.089845	0.388653
AC079336.2	3.089845	0.388653
AC090844.3	3.089845	0.388653
AC011591.1	3.089845	0.388653
CHCHD2P11	3.089845	0.388653
AC091488.1	3.089845	0.388653
AC010240.3	3.089845	0.388653
AC034236.3	3.089845	0.388653
NHLH2	-1.94994	0.38854
DCAF4L1	-0.95533	0.388536
RPS23P8	0.457666	0.388497
SASS6	0.148875	0.388482
DPF1	-0.17919	0.388469
MT-TY	0.122341	0.388429
RIOK1	0.118898	0.388369
AC211476.1	-0.59798	0.388295
DGKZ	-0.12172	0.388292
AP001020.3	-0.38156	0.388165
NDUFA8	0.121023	0.388099
KCNJ11	1.405613	0.388095
C9orf16	0.126916	0.388022
EPHX1	0.184833	0.387982
ZNF174	0.190524	0.387949
NA	-0.11838	0.387933
SIK2	-0.14767	0.387879
ZNF562	0.106263	0.387841

POFUT2	0.112534	0.387787
GEMIN2	-0.225	0.387769
RNU7-111P	-0.60209	0.38758
DDX60	-0.19227	0.387578
SYPL1	-0.11278	0.387498
AC008870.4	0.357855	0.387483
DDX11	-0.10957	0.387483
AP002807.1	0.206469	0.387479
RAB23	0.132862	0.387473
NA	-0.22344	0.387468
SENP5	0.106181	0.387444
ATP5MC2	0.124039	0.387362
RNF170	-0.13461	0.387344
AC009126.1	-0.45522	0.387331
NA	-1.33029	0.387308
GPRC5A	0.612145	0.387276
AC005740.4	-0.99166	0.387207
PTPN6	1.051468	0.387188
FOXRED1	-0.13001	0.387138
FCHSD1	-0.13299	0.38705
MINCR	0.243679	0.387007
PRSS45P	2.099496	0.386976
AC025754.1	0.414755	0.386969
MYO1G	0.712324	0.386877
MRFAP1L1	-0.10117	0.386778
AP002884.1	0.434088	0.38676
Z99572.1	0.706331	0.386754
AC112503.2	1.989668	0.386644
UNC5A	-0.1423	0.386624
STON2	-0.34902	0.386588
PIPSL	0.513071	0.386585
PMCH	0.223916	0.386555
FBXO43	1.195979	0.386541
HNRNPCP7	0.648465	0.386451
OLFM1	-0.15117	0.386395
SUPV3L1	0.143735	0.386333
IFNAR2	0.177827	0.386262
SPAG5	0.098572	0.386211
RNF121	0.116958	0.386202
HSPD1P1	0.507371	0.386194
AC127164.1	0.298118	0.38616
TTC9	-0.17656	0.386142
SEMA3A	0.222329	0.386082
C2	-0.219	0.386079
NA	0.163279	0.386026
LY6G5C	0.523678	0.386022
GNPNAT1	0.103397	0.385892
TAGLN	-0.40065	0.38581
NA	-0.21734	0.3858
NA	1.22131	0.385799

AC107294.2	-1.00712	0.38577
RAB22A	0.125803	0.385744
PRRC2A	-0.1143	0.385735
DMC1	1.439653	0.385733
GJD2	-1.84754	0.385715
TPP2	0.091764	0.385692
CCDC88C	-0.19827	0.38569
UBR7	-0.10359	0.385655
AC108693.1	1.991663	0.385633
PHLDB1	-0.09934	0.385552
IFT46	-0.15865	0.385531
MIR5692C2	-1.80757	0.385526
RORC	-0.41116	0.385467
ANTXR2	-0.11648	0.385464
RAB21	0.111914	0.385416
HMOX1	0.268102	0.385392
SRRM1	-0.09713	0.385367
MVP	-0.16568	0.385364
FAM229B	-0.18919	0.385362
NA	2.325456	0.385284
DHX57	0.100558	0.385278
RPL13P12	-0.31213	0.385201
RPL21P119	1.982333	0.385133
LINC01659	1.982333	0.385133
PRRT2	-0.14098	0.385082
FTX	0.235152	0.385058
AL136084.2	-0.74249	0.385054
PSMD10	0.143282	0.384971
HFE	-0.90371	0.384965
MKNK1-AS1	0.348109	0.384888
RNF216	0.096723	0.384881
SIVA1	0.107115	0.384842
AL096803.3	2.914004	0.38483
AC108448.2	2.914004	0.38483
KLF3P1	2.914004	0.38483
AP000317.2	2.914004	0.38483
AC027796.4	-0.34111	0.384619
RF00019	-2.19045	0.384593
AC091132.2	-1.88869	0.384584
LINC00907	0.699675	0.384513
LSM3	0.135448	0.384512
AC135178.5	-1.07889	0.384467
PHF2	0.121	0.384446
AC008736.3	-1.84618	0.384303
ADAT3	-0.23784	0.384302
TRMT44	-0.1659	0.38428
MCM3AP-AS1	-0.11724	0.38426
ZNF600	-0.28048	0.384187
RHCE	0.440503	0.384118
CERS6-AS1	-0.13137	0.384115

STOX2	-0.11261	0.383995
TRAF3IP3	0.175064	0.383955
AC048351.1	2.014302	0.383911
AL607028.1	1.063624	0.383911
GSPT1	-0.08856	0.383744
BCL2L13	0.108545	0.383678
MRPL47	0.112856	0.383658
TMEM126B	0.119835	0.38342
DGUOK	0.092431	0.383419
CMTM8	0.531388	0.383397
AL080317.3	0.242171	0.383319
DLEU7	2.308122	0.383143
MFSD2B	1.214472	0.383141
FSCN1P1	-1.757	0.38314
RPSA	0.089942	0.383094
AP2B1	-0.08123	0.38309
DPY19L4	-0.12111	0.383053
HELLS	-0.12809	0.383051
AC114730.3	-0.23546	0.382814
EPB42	-0.70583	0.382776
DBR1	-0.1143	0.382625
EPN2	0.104123	0.382607
NA	-0.16335	0.382587
AL445685.1	0.268643	0.382581
RNU6-1053P	-0.90771	0.382446
AL033519.2	0.977918	0.382338
NA	0.422506	0.382278
TRAF3IP2-AS1	0.240779	0.382267
ZNF391	0.206529	0.382263
ZFP30	-0.14337	0.382258
AP000894.4	-1.78966	0.382188
ZBTB7A	-0.13138	0.382179
AC012146.1	-0.27069	0.382138
SGF29	-0.17335	0.382136
ERG28	0.120214	0.38211
ARHGAP20	-1.63561	0.382004
RF00019	-1.0375	0.381888
AC092597.1	1.117039	0.381885
AL359317.2	0.543704	0.381881
AC064807.4	-1.22202	0.381861
NTN5	-1.61817	0.381852
PRPS2	0.131272	0.38185
CAPN2	-0.10362	0.381833
NPM1P46	1.088744	0.381832
AC131934.1	1.076199	0.381723
AL021707.3	0.621031	0.381717
C10orf143	0.5965	0.381537
NA	-2.19048	0.381465
BOLA1	0.148702	0.381443
AC090907.1	-1.86954	0.381393

PON2	-0.11583	0.381381
TRAF2	0.118801	0.381374
DCC	-0.4524	0.381315
NEK11	0.253843	0.381301
AC012055.1	0.565429	0.381234
AL513217.1	0.538379	0.381172
CRACR2A	-0.22798	0.381145
SAMD13	0.331447	0.381119
MED26	-0.23601	0.381114
PIGF	0.103031	0.381052
C16orf96	0.972728	0.381017
RPL28	0.093022	0.380985
TIAM1	-0.15286	0.380957
AC139272.1	1.372792	0.380914
SNORD12B	-1.17313	0.380906
NA	0.487375	0.380826
DNM3OS	0.147276	0.380747
OPN3	-0.12118	0.380711
FAM171A1	-0.1192	0.380658
AC005005.3	0.347747	0.380652
RASGEF1A	1.325065	0.380652
SIRPAP1	2.195459	0.380613
AC006480.1	2.195459	0.380613
PEBP1P2	2.195459	0.380613
NA	-0.27629	0.380555
TRIM66	-0.15644	0.380532
AC078929.1	0.352515	0.380529
ZNF112	0.172444	0.380445
ASAH2B	0.161634	0.380443
VWA5B1	1.125756	0.380442
CYP21A2	-0.74782	0.380427
AC013244.1	-1.66636	0.380303
LIPG	0.495611	0.380254
KIF13A	-0.12146	0.380243
NA	0.194342	0.380194
AL355075.1	-0.31766	0.38016
UQCR10	0.128718	0.380155
PPM1A	-0.09465	0.380013
ANKRD10-IT1	-0.19279	0.38001
STMN2	0.103485	0.379999
LPIN1	0.116025	0.379944
ZNF608	0.150466	0.37988
CCDC77	0.115945	0.379848
AL031055.1	1.824936	0.379847
TRPM5	2.445493	0.379833
TNFAIP6	0.219963	0.379829
TGIF2LX	0.250144	0.379797
P2RY6	-0.5006	0.379702
FAM81A	0.34798	0.379597
GLI1	0.559598	0.379566

MLF2	0.10846	0.379561
DUOX2	1.148475	0.379502
NSFL1C	0.132463	0.379447
ZPR1	-0.11141	0.379445
AC114490.1	0.286573	0.379411
C6orf201	-0.16869	0.3794
AL358176.2	-2.88732	0.379325
AC006270.2	-2.88732	0.379325
NA	-2.88732	0.379325
CCDC58P3	-2.88732	0.379325
AC012360.2	-2.88732	0.379325
AC092653.1	-2.88732	0.379325
PIPOX	0.39024	0.379226
AF131215.5	-0.14278	0.379208
WNT16	1.560852	0.379153
SPC24	0.149255	0.379121
NA	0.704586	0.379119
ARHGAP11A	-0.1176	0.379103
CRNDE	0.154286	0.379003
NRDC	-0.08866	0.378965
NA	-0.964	0.378963
SNORD83A	-1.09349	0.37887
HIST2H2AC	0.555933	0.378746
TUBA1A	0.077396	0.378733
HEBP2	-1.3058	0.378669
ALDH8A1	-2.88504	0.378636
SRGN	-2.88504	0.378636
NA	-2.88504	0.378636
NA	-2.88504	0.378636
AC137761.1	-2.88504	0.378636
AC118757.1	-2.88504	0.378636
AL033528.1	-1.74347	0.378635
GREB1	-0.1146	0.37857
ZNF324	-0.13417	0.37853
ATXN3	0.13561	0.37852
ANTXR1	-0.09478	0.378511
RABGAP1	0.092002	0.378462
RALB	-0.12463	0.378358
BX005266.2	-0.80791	0.378326
TRPM6	-0.72907	0.378313
LGR5	0.136235	0.378293
AC009318.1	0.338527	0.378231
VANGL1	-0.15833	0.378193
GREM2	-0.13016	0.378131
AL132994.1	1.42953	0.378125
LIN7B	0.209611	0.378097
AC105219.2	0.61988	0.378077
GRK3	-0.07928	0.378075
CBX3P4	1.241707	0.377961
NA	-0.92621	0.377946

AC087362.2	0.332584	0.377918
DIDO1	-0.09209	0.377846
ANAPC7	0.101419	0.377835
AL138828.1	0.667504	0.377829
ARL4A	-0.19697	0.377826
NPFFR2	-0.21752	0.377804
AC002454.1	0.326319	0.377772
RPH3AL	0.419384	0.377678
AP003721.3	-0.99332	0.377653
YPEL4	0.357646	0.377631
CCDC162P	1.579945	0.377562
ZNF205	-0.18849	0.377559
PRKAB2	-0.1231	0.377448
MRPS33	0.114527	0.377281
TNKS1BP1	0.14076	0.377215
TOMM7	0.115724	0.377177
MRNIP	0.132006	0.377173
FLCN	0.15247	0.377067
NA	2.445754	0.377039
AC073288.2	2.445754	0.377039
AC017071.1	2.445754	0.377039
MIR98	2.445754	0.377039
AC007279.1	1.874145	0.377
RPS29P5	0.858687	0.376985
SOBP	-0.17797	0.376967
RBM10	0.102531	0.376945
SDHAF4	0.226384	0.376914
HMGN1P17	2.44577	0.376867
NRG1	-0.16525	0.376824
AC016747.3	-1.05169	0.376804
DDX39B-AS1	0.822359	0.376652
ANXA11	-0.10867	0.376588
AC069234.3	-0.1799	0.376558
ZNF768	-0.10873	0.376479
BASP1	-0.14932	0.376434
RBKS	0.246177	0.376353
ISCU	0.086021	0.376305
AC092118.1	-0.54995	0.376302
MFSD14A	0.090023	0.37627
EIF4EBP1	0.088109	0.376268
OBSCN-AS1	0.355085	0.376186
MIR137HG	-0.3127	0.376177
AC010542.4	0.4084	0.37613
AL136295.5	-0.61146	0.376127
PGBD1	-0.15258	0.376035
VHL	0.09257	0.375934
STC2	0.285772	0.375879
PLAC1	1.900188	0.375846
AC135050.1	-0.19558	0.375807
MIR503	-1.452	0.375798

MAN2B2	-0.15304	0.375772
SPIRE1	0.102091	0.375767
CYB5R2	-0.5344	0.375754
SMAD1-AS1	0.230307	0.37572
AC024940.3	-1.32436	0.375655
USP28	-0.16502	0.37559
SUV39H1	-0.15366	0.375553
MIR4453HG	0.186477	0.375523
LINC02102	1.382004	0.375522
NA	-0.91508	0.375517
LRATD1	-0.35695	0.375509
IARS	0.082604	0.375467
GLIS1	-1.19039	0.375447
FAM110B	0.180912	0.375412
IQCD	0.569328	0.37536
FITM2	0.200455	0.375328
NA	-2.16128	0.375309
AC007378.1	-0.34726	0.375266
AC020913.1	-0.28433	0.375233
NA	-0.51432	0.375168
B4GALNT1	-0.12615	0.375149
S100A11	-0.3585	0.375099
PRMT3	0.148254	0.375067
RRP9	0.111283	0.37499
NA	-1.23799	0.374975
AC073912.1	-1.32789	0.374941
SPRED2	-0.12831	0.374847
YES1	0.110886	0.374842
PIP4K2A	-0.16719	0.374637
AC100793.1	1.853678	0.374568
AL390294.1	0.156261	0.374503
AC079447.1	-0.24765	0.374475
AC010320.2	-0.15663	0.37445
BARD1	0.176624	0.37443
TAB3-AS1	-1.24259	0.374399
PABPN1L	-0.43599	0.37434
TGM4	1.232439	0.374319
SYNE4	0.702427	0.374295
NA	-0.11239	0.374293
NODAL	0.457654	0.374201
RORA-AS1	0.47141	0.374185
BABAM1	0.117344	0.37414
PPFIA4	0.274261	0.374103
ECE1	-0.10101	0.374088
VAPB	-0.09602	0.374008
TUT4	-0.09506	0.373989
SCN11A	0.63026	0.373987
DLG4	0.127256	0.373939
LCMT1-AS2	-0.65215	0.373913
NUDT11	0.19303	0.373875

AC064836.3	0.758144	0.37387
PAPOLB	1.481709	0.373853
OGN	-1.68357	0.373842
AC078909.1	0.119543	0.373753
AC010300.1	1.359994	0.373732
MBD1	-0.09651	0.373575
ZNF330	0.115819	0.373575
AC008147.2	-1.44503	0.373545
HCN3	0.125123	0.373489
MROH2B	0.768107	0.373474
FNDC9	-1.46167	0.373467
AC005697.2	-0.54678	0.373457
EXOSC6	-0.1302	0.373403
CENPC	0.139229	0.373389
NA	0.199082	0.373349
RAPGEF4	-0.08967	0.373289
AC144652.1	-0.42742	0.373202
AC087521.1	0.206005	0.373186
AC010247.1	-1.09172	0.373131
SNHG32	0.084357	0.373128
UGP2	0.105749	0.373122
LINC00910	-0.3145	0.373111
FGFR3	-0.31642	0.373095
CNIH4	-0.09529	0.373068
UBA52P8	1.560726	0.372958
AC109992.1	0.402868	0.372806
USP44	-0.22617	0.372699
HNRNPCL1	1.117644	0.372697
DCAF12	-0.09116	0.372697
TMEM53	0.127935	0.372691
NOTCH2	-0.11507	0.372619
FAM151B	-0.32041	0.372569
AP001453.4	0.31451	0.372559
SLC8A1	-0.17652	0.372534
ARF1	-0.07917	0.372517
AQP6	-1.03113	0.372489
AC245297.2	-0.68879	0.372448
RPSAP61	-2.16307	0.372396
ARPC5	-0.08441	0.37236
RWDD3	0.217776	0.372358
AC015802.4	-0.30017	0.372235
THBS1	-0.50499	0.372214
ANKRD49	0.15445	0.372126
PPIEL	0.464729	0.372117
AC132192.2	0.381436	0.372101
MRPL10	0.102699	0.371996
ERGIC3	0.09055	0.371926
AC096564.1	0.571995	0.371918
HARBI1	-0.14903	0.371894
AC008635.1	0.14819	0.37184

NDUFB2	0.079368	0.371765
AL592435.1	-0.49655	0.371747
AC137590.1	-0.23658	0.371713
SEC24D	0.085356	0.371618
AC100814.1	-1.10341	0.371599
CRNKL1	0.110035	0.37158
AP003086.1	-1.14157	0.371579
LINC01137	0.205887	0.371568
AP000919.4	0.427214	0.371542
HES1	-0.19985	0.371501
LRBA	-0.10727	0.371476
MYO1F	0.455635	0.371435
TRMT61A	0.118316	0.37131
AC105129.3	-2.15939	0.371256
AC105219.1	0.751338	0.371208
NA	2.284893	0.371199
MIR4666A	2.284893	0.371199
HADHAP2	-1.92173	0.371157
LINC00592	1.73676	0.371105
MOXD1	-0.67876	0.371068
BRS3	3.03714	0.371036
NA	3.03714	0.371036
KRT12	3.03714	0.371036
KRT8P45	3.03714	0.371036
AP001610.1	3.03714	0.371036
NA	3.03714	0.371036
RHBDF1P1	3.03714	0.371036
AC010729.1	3.03714	0.371036
RPL23AP24	3.03714	0.371036
AL139130.1	3.03714	0.371036
RF01225	3.03714	0.371036
AC008953.1	3.03714	0.371036
AC027104.1	3.03714	0.371036
AC069366.1	3.03714	0.371036
NA	3.03714	0.371036
EFR3A	-0.13643	0.371027
NID2	-0.23318	0.371024
TBC1D13	-0.13567	0.370887
ACSL4	-0.1105	0.37085
TNFAIP8	0.18726	0.370779
GNG2	-0.12123	0.370776
ZNF706	0.116523	0.370754
AC073611.1	1.90832	0.37075
AL157938.3	-0.39189	0.370731
OFD1	0.11121	0.370682
DEFB1	-2.85	0.370649
LINC01562	-2.85	0.370649
NA	-2.85	0.370649
NA	-2.85	0.370649
AC004994.1	-2.85	0.370649

FAM242A	-2.85	0.370649
GOLGA8K	-2.85	0.370649
AC129507.2	-2.85	0.370649
NA	-2.85	0.370649
FAM133B	0.11341	0.370494
GALNT12	-0.40367	0.370494
NA	-0.5023	0.370492
PAQR9-AS1	-0.88905	0.370461
LRP11	-0.15854	0.370441
RF00019	-2.39591	0.370424
RNU6-233P	-2.39591	0.370424
AC092042.1	-2.39591	0.370424
AL032819.1	-2.39591	0.370424
CDS2	0.096315	0.370356
AC113139.1	1.840752	0.370352
TTC5	-0.12246	0.370329
SPINK13	1.167266	0.370292
MIS18BP1	-0.13772	0.370213
OR7E7P	-0.57448	0.370156
WBP1	0.147663	0.370139
OR7E12P	-1.16133	0.370085
CD2BP2	0.079678	0.37008
TLX3	0.184122	0.370017
RPS29	0.335128	0.369972
NA	0.4697	0.369957
HSD17B1P1	-0.23269	0.369813
SETDB2	0.152777	0.369753
C1orf50	-0.20055	0.369694
DUSP12	0.110521	0.369664
HSPA1L	0.230043	0.369633
TEK	1.926212	0.369538
HHIP	-1.29083	0.369523
RHPN2	-0.23622	0.369471
PLGLB2	0.453491	0.369418
ALDH1L1-AS2	-2.16304	0.369322
MRS2	0.117494	0.36926
RF00340	-2.84532	0.369253
AC005000.1	-2.84532	0.369253
RF01233	-2.84532	0.369253
AC092902.1	-2.84532	0.369253
AC026407.1	-2.84532	0.369253
HERC2P11	-2.84532	0.369253
ZC3H13	-0.08386	0.369237
AC090825.1	0.438583	0.369232
PBDC1	0.134564	0.369217
AC091132.4	-0.81038	0.369183
FARP2	-0.11031	0.36918
ROBO2	0.513033	0.369084
C3orf38	0.127342	0.369006
BDH1	-0.14384	0.369002

AL670729.1	-0.17947	0.368988
AMER3	0.244962	0.36896
MRPS6	-0.14148	0.368926
LINC00898	-1.73344	0.368896
RN7SL388P	1.675313	0.368891
PEF1	0.101326	0.368847
HSPA8P5	1.237485	0.368835
PUF60	0.097439	0.368801
AL391095.1	0.510503	0.36874
AC079145.1	0.605595	0.368693
NACAD	-0.15678	0.368591
MTRF1	-0.15942	0.36859
PMP22	-0.14886	0.368576
PHC2	-0.10033	0.368453
RNA5SP118	0.917941	0.368435
SGSM1	-0.17533	0.36833
ESPL1	-0.09673	0.368314
RP9P	0.148319	0.368306
HOXC4	-0.58183	0.368288
AC092691.2	1.672747	0.368262
LINC02572	1.6415	0.368259
GPR152	2.284945	0.368228
MIR199A1	2.284945	0.368228
MYL7	-1.37219	0.368209
BNIP3P39	-2.15935	0.368182
C16orf95	-0.61454	0.368051
SRSF3	0.086885	0.368045
AC007622.1	2.284948	0.368045
MTCO1P30	2.284948	0.368045
AC025164.1	0.172438	0.368026
ZNF626	-0.40403	0.368024
CDK9	0.10987	0.368015
EFNA5	-0.16748	0.36801
DNM1P35	0.776231	0.367948
PTPRS	-0.11384	0.36792
FKBP1A	0.084624	0.367862
AC025871.1	-0.72519	0.367856
AL024507.2	0.188189	0.367846
NA	-2.2525	0.36778
DIXDC1	-0.15917	0.36774
AC091153.1	-1.10304	0.367667
AC079416.1	-0.79731	0.367633
TTC28	-0.09862	0.367585
MYL2	-2.38607	0.367565
ANKRD40CL	-2.38607	0.367565
RNU1-134P	-2.38607	0.367565
AP000357.1	-2.38607	0.367565
RPL36AP13	-2.38607	0.367565
PPP6R3	0.087191	0.367544
B3GALT6	-0.1321	0.367543

FAM3C2	-0.32651	0.367501
RSBN1L	-0.09518	0.367483
MAP1LC3B	-0.09251	0.367469
FAM166A	0.178025	0.367373
MCUB	-0.21262	0.367259
MIR219A1	-2.25073	0.367255
NA	-2.25073	0.367255
CYP51A1P2	-2.25073	0.367255
CDH2	0.104025	0.367247
AL136038.3	-1.15287	0.367218
NUP107	0.091251	0.367192
AL354919.2	0.940755	0.367153
RNU6-850P	0.639823	0.367056
SYNGR3	-0.36619	0.367038
LAMA1	-0.16197	0.36699
PIWIL4	-0.56092	0.366968
NA	1.596037	0.366962
NA	-1.11552	0.366864
IGSF6	-0.61521	0.366803
NA	-0.25515	0.366782
MAGEB6	-1.02371	0.366636
ABCF1	-0.08243	0.366597
PNPT1	-0.1037	0.366471
HEBP1	0.225425	0.366453
AL133163.3	-0.5351	0.36643
RBM14-RBM4	-2.39582	0.366317
AC019257.2	-2.39582	0.366317
LINC02575	0.264463	0.366262
NRSN2	0.110522	0.366237
SPSB4	-0.22738	0.36622
C2CD4D	-1.98347	0.366207
MSRB3	-0.26759	0.3662
ABCB8	-0.1232	0.366194
PTMS	0.12788	0.366193
LINC00342	-0.2168	0.366186
ATP8A1	0.134601	0.366173
COL24A1	1.633191	0.366113
RNF181	0.118919	0.366065
ZNF236-DT	0.391593	0.366016
CT75	0.161007	0.365983
COTL1	-0.15254	0.365969
SNORA12	1.783467	0.365906
PPP3CB	-0.11505	0.36584
HTT	-0.10982	0.365821
FGD4	0.156558	0.365793
ZNF605	-0.13186	0.365745
ECI2	0.129685	0.365719
BAD	0.114562	0.365703
SMAD5	0.093731	0.365653
FAM43A	-0.22834	0.365573

YIPF1	0.1416	0.36556
AC008393.1	0.349537	0.365377
CRHR1	1.957734	0.365243
CERKL	0.214539	0.365241
DOLK	-0.13866	0.365104
AC020978.1	-1.70393	0.365042
NA	-0.16603	0.365041
AL160191.1	1.014293	0.365011
NA	-1.21746	0.364998
TEDC1	-0.15222	0.364918
UBIAD1	0.124278	0.364913
SNORA7B	-1.49301	0.364905
YWHAB	-0.07618	0.364844
PLD4	1.406397	0.36482
BNIP3P15	-1.84374	0.364756
SH3TC2	-1.08821	0.364716
MTFP1	-0.55983	0.364709
AL162741.1	-1.97717	0.364709
RPL23AP86	-1.97717	0.364709
PBX1	-0.11891	0.364709
LIFR	0.150446	0.364682
PKIG	0.114183	0.364675
VTN	0.404221	0.364635
ADAMTSL5	-1.07806	0.364583
SPINT1	1.088686	0.364538
SRRM5	0.114089	0.364527
SHQ1	0.121756	0.364519
PARP3	-0.14285	0.364449
AC017100.1	-0.41391	0.364421
MRPL2	-0.13719	0.364407
AC008966.1	-0.29261	0.364331
DCAF4	-0.18255	0.364327
CASC9	-0.31344	0.364281
HASPIN	0.209401	0.364279
EEF1AKMT3	0.194559	0.364241
PLPP1	-0.13976	0.364184
TRAFD1	-0.10866	0.364154
AL928654.1	0.250261	0.364075
DRAM2	0.123333	0.364056
TMEM206	-0.09985	0.363962
AL024508.2	-0.30048	0.363961
CEP70	0.190392	0.363913
LHFPL3	0.736677	0.363871
ARF4-AS1	0.607902	0.363723
RF00019	-1.59975	0.363705
SMARCA5	-0.07887	0.363687
AC005625.1	-1.4968	0.363678
SPECC1	-0.11273	0.363588
LINC00632	-0.28609	0.363515
AC011290.1	-2.38596	0.36346

AC009365.3	-2.38596	0.36346
AC103724.3	-2.38596	0.36346
MIR4733	-2.38596	0.36346
TRPV3	-2.39576	0.363439
NPIPB13	-2.39576	0.363439
PRSS44P	-2.39576	0.363439
CLEC2L	-2.39576	0.363439
AC008897.2	-2.39576	0.363439
ZNF227	0.127789	0.363356
LINC00649	0.1543	0.363347
AC005842.1	1.906481	0.363305
AC243964.2	1.884735	0.363289
AC092070.3	-0.70927	0.363275
PIGHP1	-0.85279	0.36322
CD99P1	1.046158	0.363205
FMNL3	0.115891	0.363153
C1DP1	0.736905	0.363127
AC131281.1	-2.25092	0.363099
AL355922.2	-2.25092	0.363099
NA	-2.25092	0.363099
MAP7D3	0.131779	0.36307
BCS1L	-0.11052	0.363026
CDC42BPG	0.361512	0.363021
MAPKAP1	-0.09555	0.362993
CELF4	-0.17093	0.362984
HDAC1P2	-0.76551	0.362967
ARHGEF11	-0.0955	0.362951
LINC02044	-0.77405	0.362944
NA	-0.2259	0.36285
B3GNT2	-0.13357	0.362845
OTULIN	0.109485	0.362828
SH3D19	0.081663	0.362824
LINC01238	-0.24021	0.362811
NUTF2P2	2.067683	0.362779
AP001922.6	-1.11427	0.362718
AC138932.3	0.991474	0.362645
TCTE1	0.244351	0.362641
ATP10B	-2.22002	0.362561
RHOQP3	-1.7929	0.362476
FBXW2	-0.09134	0.362337
AF131216.1	0.197479	0.362325
LYG2	0.717621	0.362321
AL445524.1	0.294328	0.362244
NA	1.618793	0.362104
ZFAND6	0.098593	0.36209
CCDC30	0.312029	0.362086
TK1	-0.11761	0.362086
BACH1	0.101092	0.362081
PCDHA4	-0.28922	0.362076
TMEM270	-1.94181	0.361976

AC025165.1	0.113431	0.361872
PARP8	0.132899	0.361872
AC093890.1	1.869255	0.361864
C11orf68	-0.12454	0.361819
GPR157	-0.32231	0.361779
NA	0.593086	0.361763
AC123768.3	0.534168	0.361748
AC025569.1	-0.54946	0.361703
CACYBPP2	0.488326	0.361698
AC012618.2	0.890505	0.361691
USP6NL	-0.11461	0.361641
SLC25A32	0.10753	0.361612
AC040977.1	-0.68694	0.361611
AL158824.1	2.237219	0.361559
SMIM30	-0.13103	0.361544
PAFAH1B2	-0.11935	0.361535
NUDT6	-0.19968	0.361518
COL5A2	-0.52242	0.361517
AC127540.1	1.630904	0.36148
TATDN1	0.103814	0.361456
CALB1	-1.13078	0.361406
CFAP97	0.102936	0.361362
AC092724.1	0.370829	0.361333
AC011092.2	1.483662	0.361329
CMBL	-0.11437	0.361288
P4HA2	-0.1597	0.361287
AC113935.1	0.391491	0.361264
NA	2.062034	0.361165
P2RX2	2.062034	0.361165
AC072062.1	2.062034	0.361165
RNU7-59P	2.062034	0.361165
C1orf43	0.087047	0.361098
CWC27	-0.13089	0.361086
TENM2	-0.61799	0.361086
AC138230.1	1.205067	0.361035
RNU5E-10P	-1.72544	0.360946
MAP6D1	-0.29037	0.360808
C1QTNF3	-0.38622	0.360807
RCC2P6	-0.97596	0.360802
AL451054.1	2.843174	0.360751
NA	2.843174	0.360751
SLC25A1P1	2.843174	0.360751
SPEM2	-2.25283	0.360704
KLRG2	-2.25283	0.360704
MAP1LC3C	-2.25283	0.360704
MTHFD1P1	-2.25283	0.360704
AKAP8P1	-2.25283	0.360704
VTCN1	1.570507	0.360686
THAP5P1	1.570507	0.360686
MTRF1L	-0.12379	0.360686

C15orf39	-0.25241	0.360662
PHACTR2P1	-1.55884	0.360631
C4orf50	-0.63389	0.360614
AC005077.4	-2.38588	0.360582
AC006042.3	-0.98376	0.360544
ABCC8	0.536777	0.360518
EXD2	0.12483	0.36045
AC034102.5	0.100775	0.360359
HLA-K	1.181218	0.360331
MTTP	-0.83784	0.360276
AL353795.2	1.472454	0.360239
RHEBP2	0.864443	0.360218
MPV17L	0.301831	0.360197
NA	-2.25105	0.360179
AC022424.1	-2.25105	0.360179
RNU6-1095P	-2.25105	0.360179
AC119751.6	-0.86885	0.360169
SLC2A13	-0.17875	0.360136
AC091152.2	0.443536	0.360131
NA	0.737008	0.360097
IL13RA1	-0.14719	0.360082
PTGER1	0.404325	0.36008
MORC4	-0.20682	0.360076
AC109460.4	0.684112	0.360062
TOX	0.15435	0.360035
XAGE3	-1.14658	0.359971
HACD4	0.268999	0.359919
TMCC3	0.524541	0.359855
KCNN1	-0.27826	0.359802
MMP17	0.2296	0.359658
FAM86B1	0.400033	0.35964
NA	-0.83269	0.359636
CARNS1	0.521676	0.359598
AP001893.1	-0.70584	0.359524
ABL2	-0.09641	0.35951
KDM7A	0.111077	0.359346
FAM184B	0.16397	0.359331
H2AFY2	-0.09886	0.359274
NFKBIL1	0.134119	0.359239
GPATCH2	0.103319	0.359208
AL138733.1	-1.82815	0.359134
VN1R51P	1.43996	0.359114
ARGFXP2	1.025662	0.359053
AC139713.1	-0.47924	0.358997
AC007780.1	1.244879	0.358963
AC073342.1	1.133903	0.358963
APLP1	-0.13944	0.358959
AC008067.1	-0.32092	0.358904
ANKRD36	0.233913	0.358851
CDK5RAP1	-0.12353	0.358827

NEK4	0.14491	0.358736
IGHVII-46-1	-1.31052	0.358691
SLX4	-0.14016	0.35869
AC133552.2	0.627558	0.358686
ATP6V0C	-0.76072	0.358639
AC115099.1	2.237214	0.358518
WBP4	0.138194	0.358468
SLA2	-0.18776	0.358466
EPN3	-0.86102	0.358458
GAPDH	0.097271	0.358398
IFNGR2	0.10333	0.358377
RBP1	0.109682	0.358355
SYTL2	-0.46093	0.358346
ATG4A	-0.18255	0.358294
ERLNC1	0.407154	0.358265
TMEM129	-0.10457	0.358256
CXXC5	-0.08958	0.358237
AL158151.1	0.205076	0.358217
AL451007.1	1.1492	0.358208
SLC16A13	-0.45083	0.358196
DGCR6	-0.22411	0.358154
AL671277.1	0.11039	0.358053
AC010245.1	-0.21744	0.358003
SCN8A	0.150019	0.357995
ADRA2A	-0.18887	0.357994
AC109347.1	0.430561	0.357971
DCAF15	-0.10916	0.357925
AC021078.1	0.127056	0.357847
EIF4A2P2	-1.281	0.357787
IFNGR1	0.108713	0.357722
FSIP2-AS2	1.505243	0.357688
RRN3P3	0.200777	0.357671
SPARCL1	-0.45133	0.35761
CDC37L1-DT	-0.51841	0.357552
EEF1E1	0.2132	0.357445
SARAF	0.077907	0.357355
RNA5SP82	-0.66055	0.35729
ATG4C	-0.15491	0.357244
NIPBL	-0.08358	0.35721
AC025043.1	0.14524	0.357203
ZNF329	0.188291	0.357165
SEC11C	0.119635	0.357113
AC010501.1	-0.5925	0.357065
SNHG12	-0.11679	0.356935
AP003774.1	-2.81178	0.356908
MAGEB16	-2.81178	0.356908
AL031770.1	-2.81178	0.356908
NA	-2.81178	0.356908
MTRF1LP1	-2.81178	0.356908
AC061975.4	-2.81178	0.356908

LIN54	0.126467	0.35688
LINC02043	-1.75032	0.356879
TOLLIP-AS1	-0.78691	0.356858
DCAF8	-0.08734	0.356757
ESPNL	0.47578	0.356752
CCNC	0.112095	0.356659
TMEM238	0.362158	0.356631
MAGED4	-2.22383	0.356367
METTL5	0.107296	0.35636
CDO1	-0.21935	0.356334
AL049834.1	0.217657	0.356169
CXCL1	-0.62866	0.356138
KLHDC10	-0.09465	0.3561
NECAP1	0.107557	0.356001
SH3GL1P2	-0.89051	0.355996
AC024933.1	-0.95759	0.355992
MORF4L2	0.088937	0.355893
ARL4C	0.164294	0.355884
BCL7B	-0.10323	0.355847
DONSON	-0.09869	0.355835
CAV2	-0.7184	0.355766
SWT1	0.177123	0.355601
RAB24	0.214902	0.355495
FRS2	0.097373	0.355488
MIR1915HG	-0.17505	0.355453
MYL3	-1.19434	0.355452
INPP4A	-0.103	0.355435
CASKIN1	-0.10447	0.355389
AL590705.1	0.613503	0.355387
AC064852.1	-0.31611	0.355309
DLX5	0.363825	0.35529
BCL7C	-0.10014	0.355276
AC010894.4	-0.35198	0.355183
USF2	0.094383	0.355137
CNTN6	-1.11694	0.35512
METTL1	-0.15719	0.355116
RNF212	-1.18396	0.355111
MFHAS1	0.17115	0.355103
RDH13	0.164817	0.355076
AC022121.1	1.720952	0.355036
AC104333.1	-2.19441	0.35498
NA	0.131302	0.354906
PHF12	-0.1214	0.354891
NA	0.868787	0.354885
MRPS30-DT	-0.37985	0.354849
RF00019	-2.80456	0.354831
BDH2P1	-2.80456	0.354831
PHF5CP	-2.80456	0.354831
AC011471.2	-2.80456	0.354831
PLTP	-0.12815	0.35481

UPF3B	0.105511	0.354789
AC004801.2	0.396226	0.354705
AC131009.1	0.320992	0.354683
IGSF10	-0.69721	0.354675
ZNF878	-0.92839	0.354636
NDST1	-0.10913	0.354616
AC019130.1	0.394588	0.354613
RF00019	-1.24301	0.354598
NLGN1	0.243104	0.354586
CETN4P	-1.7788	0.354565
AC068987.3	-0.66859	0.354414
CHST9	1.155294	0.354412
BMP7	-0.44016	0.354369
SOCS2	0.084441	0.35428
RAX2	2.819591	0.354229
MIR138-1	2.819591	0.354229
SUPT20HL1	2.819591	0.354229
LINC01513	2.819591	0.354229
AC138123.1	2.819591	0.354229
NA	2.819591	0.354229
TACC2	-0.17948	0.354123
AC084866.1	0.699348	0.354063
MCM2	-0.08333	0.354029
ADARB1	-0.16812	0.35402
RDH16	1.459343	0.353861
AC024940.1	0.395555	0.353788
SNRPF	0.097512	0.353738
RMST	-0.52804	0.353674
AP003692.1	-0.14544	0.353648
AL359922.2	0.18037	0.353608
MAN2B1	0.131242	0.353581
NA	1.616622	0.353518
BEST1	0.098429	0.353368
NA	-0.95162	0.35334
ATRIP	-0.13859	0.353281
RAB9B	0.220218	0.353254
MUC20	-0.49944	0.353247
AL034374.1	-1.13768	0.353221
GYS1	-0.11585	0.353217
HCRTR1	0.215326	0.353137
CD40	-1.78261	0.35312
MRI1	-0.11361	0.353058
AL513534.1	-0.47647	0.35303
SLC25A33	-0.14455	0.352938
TECPR2	-0.14266	0.352934
DDX58	-0.19129	0.352899
COX6C	0.108591	0.352898
GUSBP5	0.379643	0.352891
UBN2	-0.08457	0.352834
BX649632.1	0.809983	0.352769

NA	0.16459	0.352757
NCK1-DT	-0.39769	0.352745
RAB28P5	-0.90545	0.352729
CCNT1	0.093674	0.352669
PTPN18	-0.13908	0.352668
AC122718.2	-1.65692	0.352502
ZNFX1	-0.10365	0.352486
RNU6-925P	-1.32029	0.352482
HPCA	-0.33974	0.352392
UNC5CL	0.646486	0.352345
ANGPT1	0.229106	0.35233
SYTL5	-0.85303	0.352198
SYMPK	-0.10157	0.352192
PLEKHD1	0.621207	0.352102
AC073333.1	0.140093	0.351993
NUTM2A-AS1	-0.14158	0.351931
RGS2	0.155412	0.351897
UNC5B	-0.09686	0.35185
WWTR1	0.194893	0.351818
MPHOSPH6	0.16151	0.351815
LNX1	-0.15486	0.351791
DEAF1	0.097217	0.351736
NA	-1.37377	0.351681
SLC7A6OS	-0.09162	0.351681
AL391825.1	0.832552	0.351674
TMX2P1	-0.25451	0.351625
KRBOX4	0.126122	0.3516
BAZ2B	0.094898	0.351574
RPL31P49	1.488006	0.351541
XIAP-AS1	1.745201	0.351539
MORC2-AS1	-0.20736	0.351522
AC133561.1	2.80292	0.351491
RF00093	2.80292	0.351491
RF00493	2.80292	0.351491
PRSS3P4	2.80292	0.351491
AC087343.1	2.80292	0.351491
AC006445.2	2.80292	0.351491
SNORD3A	2.80292	0.351491
AC025265.1	-1.05818	0.351387
NA	-0.20309	0.351359
LRRC37A	-0.46066	0.351349
SMYD3	0.132617	0.351345
SLC3A1	0.113097	0.351298
RIOK2	-0.13587	0.351233
ATF4P3	-1.49425	0.35123
PTP4A2	0.084624	0.351183
KLHDC8A	0.285672	0.351158
RNF44	-0.10662	0.351121
UBE4A	-0.12936	0.351107
NA	0.181641	0.351077

TGFB3	0.345031	0.351048
FBXO33	-0.1534	0.351047
AL158835.2	0.684231	0.350917
AL353796.1	-0.2827	0.35087
IGSF22	-0.36886	0.350835
AC018638.6	-1.5893	0.350791
NA	1.518	0.350781
PRG4	0.172737	0.350765
SECISBP2	0.100319	0.350759
AGAP6	0.126738	0.350701
CSGALNACT2	-0.1065	0.350644
LINC00513	1.352813	0.350591
SNORA5A	-0.73708	0.350551
ADI1	0.083522	0.350484
ZBTB7C	0.288691	0.350334
TRIM55	-0.32816	0.350129
NDUFS7	0.108973	0.350044
PPM1D	0.107198	0.350025
HEPH	0.536534	0.349944
FIS1	0.094808	0.349938
NRG3	0.330945	0.349934
SERPINE1	-0.37898	0.349898
AC064874.1	2.212712	0.349894
SOX18	-0.67451	0.349894
AC010343.3	-1.16082	0.349839
RFPL3S	-1.11629	0.349816
APEX2	-0.11067	0.349761
NA	0.820627	0.34975
RPL10P6	1.880015	0.349747
NA	1.880015	0.349747
NA	0.781425	0.349689
ATP5PD	-0.09646	0.349636
HSPA8P3	1.646912	0.349607
CERNA1	-0.90234	0.349567
ARHGAP39	0.146211	0.349493
AC114689.3	0.450917	0.349465
AC005670.1	0.879935	0.349443
RCOR3	-0.10903	0.349425
LRRC56	0.315197	0.349408
KRT18	-0.31705	0.349374
LINC01828	-1.05537	0.349309
ATP6V1G1P2	1.342093	0.349269
ACER2	0.445759	0.349246
RPS2P55	0.5462	0.349225
AC007319.1	1.906145	0.349158
SVIL2P	1.906145	0.349158
USHBP1	-0.29147	0.349138
AL161740.1	-2.18894	0.349109
AC007666.1	2.342706	0.349108
RPL12P35	2.342706	0.349108

SNX18P24	2.342706	0.349108
NA	2.342706	0.349108
AC109449.1	-0.57713	0.349104
ASH1L	-0.08186	0.34905
POLE4	0.180021	0.349022
MAP3K13	-0.10725	0.348967
ENDOV	0.12617	0.348965
NA	0.273654	0.348941
AC079328.2	-0.13671	0.34889
PXN	0.146688	0.348846
EIF1AD	-0.11401	0.348837
DSCAM	-1.89523	0.348798
WDHD1	0.151798	0.348723
AL050331.2	-0.36724	0.348648
MIR4435-2HG	-0.2455	0.348607
COL4A6	-1.52968	0.34854
DHFR2	0.169479	0.348525
PDGFRB	0.145447	0.348504
GDF5	1.795901	0.348477
DCUN1D3	0.133798	0.348466
ZNF510	-0.11955	0.348452
AC026471.1	0.174465	0.348403
FAM189A1	1.330163	0.348398
STARD3	-0.10384	0.348385
ANKRD26	-0.14612	0.348367
ELP1	-0.08288	0.348351
ORC3	-0.12997	0.348303
NA	1.872554	0.348284
RPUSD4	-0.12356	0.34821
AC231533.2	-1.78685	0.348188
RPP38	-0.14417	0.348171
AC079781.1	1.590489	0.348167
TUT7	-0.11667	0.348149
AC105339.3	0.913255	0.348144
NFKB2	0.133938	0.348137
SLC43A2	0.169146	0.348132
GNG12	-0.10596	0.348122
PPP2R2A	-0.10142	0.348078
CEP170B	-0.15531	0.348075
TSPAN33	0.159728	0.348068
SNX30	0.12509	0.348042
KCNH4	-0.26723	0.348014
NIFK	0.096025	0.348014
AL606469.1	-2.0456	0.34799
AC109631.1	-2.0456	0.34799
NA	-2.0456	0.34799
AC004241.1	-0.46151	0.347967
CPSF4L	-0.17871	0.347878
RPL23AP66	1.793203	0.347862
NA	1.793203	0.347862

ING2	-0.17269	0.347809
AL118558.1	-0.11321	0.34779
PITPNB	0.094172	0.347743
NPLOC4	-0.08218	0.347719
AC010200.1	1.898594	0.347695
ZNF252P	-0.09508	0.347669
PTPN2	0.11644	0.347619
ZBTB11	-0.11437	0.347588
AC008741.1	-0.28716	0.347563
AC005944.1	0.084418	0.347507
TXLNG	-0.08918	0.347474
CSTF3	-0.10801	0.347448
NA	-0.38141	0.347411
APOL1	0.921411	0.347377
VAMP2	-0.14245	0.347347
SNORA11	-0.98105	0.347309
PLIN3	-0.12446	0.347296
ATP8B2	-0.09619	0.347291
CDC42SE2	0.108098	0.347191
AC012313.1	-0.20989	0.347153
RNU6-583P	1.367057	0.347109
AL669841.1	-0.57945	0.346966
PNMA1	-0.08291	0.346953
OSCP1	0.236946	0.34695
HOXD13	2.212624	0.346924
RF00560	2.212624	0.346924
RPS2P45	2.212624	0.346924
AC011389.1	2.212624	0.346924
AP001094.2	-1.70606	0.346876
RPA1	-0.08083	0.346827
AC093726.2	0.672215	0.346826
HSP90AB2P	1.444205	0.34681
CTXN2	1.083107	0.346787
AP005202.1	1.557996	0.346782
AL591043.2	-0.64569	0.346765
NEUROD2	2.212618	0.346741
NA	2.212618	0.346741
RPS8P10	2.212618	0.346741
RERE-AS1	2.212618	0.346741
AC069200.1	2.212618	0.346741
GPR107	-0.07877	0.346731
MYO16	0.228731	0.346728
NXPE3	0.096945	0.346696
AP003068.2	-0.38047	0.34662
FAM92A1P1	-2.76761	0.346555
AC002056.1	-2.76761	0.346555
AL136322.1	-2.76761	0.346555
AC040975.1	-2.76761	0.346555
AC145207.7	-2.76761	0.346555
AP000654.1	-2.76761	0.346555

CCDC15	-0.25915	0.346446
SIGLEC1	-2.92547	0.346418
NA	-2.92547	0.346418
CFHR3	-2.92547	0.346418
F13B	-2.92547	0.346418
SFRP2	-2.92547	0.346418
HIST1H2BA	-2.92547	0.346418
GLYAT	-2.92547	0.346418
ODF1	-2.92547	0.346418
CLDN19	-2.92547	0.346418
PCDH19	-2.92547	0.346418
MIR1-1HG	-2.92547	0.346418
OR2B7P	-2.92547	0.346418
C17orf102	-2.92547	0.346418
KCNMB2	-2.92547	0.346418
RNU1-94P	-2.92547	0.346418
RNU6-37P	-2.92547	0.346418
RF00416	-2.92547	0.346418
RF00019	-2.92547	0.346418
NA	-2.92547	0.346418
NA	-2.92547	0.346418
NA	-2.92547	0.346418
NA	-2.92547	0.346418
ELOA2	-2.92547	0.346418
RNU6-571P	-2.92547	0.346418
RPL7P29	-2.92547	0.346418
BASP1-AS1	-2.92547	0.346418
AL031119.1	-2.92547	0.346418
AL157777.1	-2.92547	0.346418
RN7SKP213	-2.92547	0.346418
AL080273.1	-2.92547	0.346418
AC006965.1	-2.92547	0.346418
LINC02671	-2.92547	0.346418
Z82186.1	-2.92547	0.346418
MEG8	-2.92547	0.346418
CLYBL-AS2	-2.92547	0.346418
PNKDP1	-2.92547	0.346418
AL356310.1	-2.92547	0.346418
MTND1P9	-2.92547	0.346418
AL450327.1	-2.92547	0.346418
FTH1P1	-2.92547	0.346418
CHRM3-AS2	-2.92547	0.346418
AC013476.1	-2.92547	0.346418
NA	-2.92547	0.346418
AL138799.3	-2.92547	0.346418
AC019117.1	-2.92547	0.346418
LINC01522	-2.92547	0.346418
COX6CP10	-2.92547	0.346418
NA	-2.92547	0.346418
NA	-2.92547	0.346418

NA	-2.92547	0.346418
RN7SL258P	-2.92547	0.346418
LINC00939	-2.92547	0.346418
TERB1	-2.92547	0.346418
AC010638.1	-2.92547	0.346418
AC090015.1	-2.92547	0.346418
AC011632.2	-2.92547	0.346418
MRPL9P1	-2.92547	0.346418
ENPP7P8	-2.92547	0.346418
AC121334.1	-2.92547	0.346418
ENTR1P1	-2.92547	0.346418
CHMP4BP1	-2.92547	0.346418
AF111169.2	-2.92547	0.346418
AL512347.1	-2.92547	0.346418
NA	-2.92547	0.346418
BNIP3P24	-2.92547	0.346418
AC008687.6	-2.92547	0.346418
AC008758.6	-2.92547	0.346418
AL021997.1	-2.92547	0.346418
AC013467.2	-2.92547	0.346418
NA	-2.92547	0.346418
NBL1	-0.32626	0.346417
NME6	-0.15811	0.346359
EXOSC3	0.106952	0.346252
SLC25A30-AS1	0.865737	0.346226
AP006222.1	-0.8088	0.346216
AL157700.1	-0.81107	0.346203
DNAJA1P3	2.342456	0.346196
RPL9P2	2.342456	0.346196
OR7E158P	2.342456	0.346196
AC091062.1	2.342456	0.346196
MIR3681HG	-1.45872	0.34619
SSNA1	0.109542	0.346165
AC009053.3	-2.18896	0.346115
TRIM27	0.078956	0.346103
TOGARAM1	-0.1551	0.346018
AC241520.1	2.342441	0.346017
CCT4P1	2.342441	0.346017
AMIGO2	-0.2506	0.346
TERF1	0.102945	0.345949
ZNF579	0.150213	0.345903
POC1B-AS1	0.553213	0.345873
WASH4P	0.209217	0.345793
NUDT16P1	-1.80641	0.345756
AHCTF1	-0.08732	0.345724
MPZ	-0.33452	0.34567
C11orf95	-0.10165	0.345608
SLC9A7	-0.4636	0.345568
ENSA	0.080683	0.345565
C5AR1	-1.78572	0.34554

LINC01234	0.220383	0.345519
UBE2D3-AS1	0.365505	0.345427
AP000873.2	0.183009	0.345357
ZFHX3	-0.09538	0.345292
BSX	-1.05537	0.345266
AC010325.2	2.18184	0.345192
TBC1D30	0.392711	0.345172
CBY1	-0.18423	0.345165
ZMYND10-AS1	-0.6674	0.345149
SCN3B	-0.16578	0.345112
MPP1	-0.18816	0.345111
INTS11	-0.11227	0.345029
ARMC7	-0.15499	0.344991
MAGEA11	-2.77003	0.344964
SUCLG2P2	-2.77003	0.344964
AP002770.2	-2.77003	0.344964
AC004130.1	0.567289	0.344959
HECTD2	0.116951	0.344948
CPEB1-AS1	-0.69137	0.344947
FAM169A	0.141028	0.344947
AC025283.2	0.28073	0.344878
UBAP2	0.108478	0.344812
CHD9	0.092723	0.344761
MIR106B	0.71403	0.344753
HDHD3	-0.20882	0.344744
SUPT6H	-0.0786	0.344641
AC004148.2	0.12667	0.344579
TM9SF4	-0.09042	0.344564
DEC1	1.68901	0.344515
AL110292.1	0.713362	0.344481
PALB2	0.11852	0.344476
STARD6	-1.00611	0.34442
LINC02051	1.415968	0.344388
TBK1	0.095346	0.344366
ORMDL3	0.110852	0.344251
PRKCE	-0.15264	0.344237
EAF2	-0.7674	0.344227
AC107926.1	0.666503	0.344194
LINC01886	1.577969	0.344149
AC010326.3	0.214068	0.344111
AC016026.1	-0.99803	0.344049
MED14	-0.08681	0.344029
DPP9	-0.11242	0.343956
MAP3K11	-0.09456	0.343944
AC069222.1	-0.70616	0.343931
CATSPER1	0.81715	0.343916
ANO5	0.158456	0.343884
AL117350.1	-0.32502	0.343754
NA	0.831247	0.343753
FAM76B	0.105385	0.343692

AC074032.1	0.692277	0.343691
AC091132.1	-0.84728	0.343683
BCO2	-0.74392	0.343666
NA	-1.26421	0.343653
DBF4B	-0.12958	0.343637
MAN2A2	-0.0969	0.343625
CACNA1I	-2.76508	0.343576
RF00019	-2.76508	0.343576
CGB8	-2.76508	0.343576
AC005703.2	-2.76508	0.343576
RPS26P52	-2.76508	0.343576
HMGN1P10	-2.76508	0.343576
AC106800.2	-2.76508	0.343576
AC009093.1	-2.76508	0.343576
TCTN1	0.102037	0.343565
AL360091.1	0.973832	0.343465
STK25	0.087685	0.343434
SCARNA3	1.251592	0.343381
AC117453.1	0.820032	0.343381
ZNF253	0.16154	0.34336
FRK	0.669448	0.343321
FOXO3B	-0.24059	0.343258
NOX1	0.501746	0.343187
GGCX	0.085962	0.343183
HGFAC	0.562769	0.343137
IBA57	0.138682	0.343102
AL132777.1	-1.43428	0.34307
CMPK2	-0.24359	0.343059
DEFB131B	-0.87208	0.342998
AL359921.2	0.494581	0.342958
AC023908.3	0.276872	0.342899
GNE	-0.11754	0.342858
SIGMAR1	-0.10117	0.342851
BEND6	0.166467	0.342844
NDRG1	0.177153	0.342816
ELMO1-AS1	-0.30043	0.342735
DNAJC9	-0.10645	0.34271
THEGL	-1.60452	0.342655
STX12	-0.10233	0.342626
AL035658.1	0.522402	0.342611
AP002784.1	-0.97774	0.342605
C4orf36	0.37082	0.342538
AC112721.1	-1.72186	0.342512
AC073476.1	-0.47166	0.342478
OGFR-AS1	-0.61901	0.342474
NA	-1.05364	0.342414
METTL22	0.180531	0.342408
AL645504.1	-1.06851	0.342405
SNRPD2	0.085097	0.342379
PARP10	0.157693	0.342359

FAM210B	-0.0984	0.342332
CCDC106	-0.13623	0.342319
EIF4EBP2	-0.07839	0.342317
EIF4B	0.085245	0.342303
PLXNA3	-0.13208	0.342227
AC024267.5	-2.16461	0.342208
AC011306.1	-2.16461	0.342208
CASP7	0.133418	0.342169
TRIM4	0.127636	0.342125
HLTF	-0.10292	0.342124
TWIST2	-0.7218	0.342094
C22orf34	1.163352	0.342093
COX6B1	0.0861	0.342063
IPO9	-0.07797	0.342036
CXADR	0.149177	0.342029
SNTG1	1.118982	0.342017
C11orf42	0.89863	0.342001
H6PD	-0.13749	0.341974
RCN3	0.169702	0.341946
SLC6A15	0.105702	0.341926
BCAT1	0.074539	0.341916
PRUNE1	-0.10003	0.341891
SDK1	-0.13732	0.341878
NA	2.181674	0.341871
AC002546.1	2.181674	0.341871
HMGB1P5	-0.21457	0.341787
AC087289.5	0.306441	0.341782
FAM172A	-0.13591	0.34176
REXO1	-0.15132	0.341755
ZNF165	0.511049	0.341717
AC007611.1	-0.70528	0.341664
SLC25A15	0.114192	0.341582
CCDC189	-0.12691	0.34158
MYCNOS	0.517834	0.341571
IFT140	-0.12449	0.341425
DLAT	-0.13941	0.34142
AC108463.1	-1.3081	0.34129
DHCR24-DT	1.432232	0.341218
LINC00672	-0.22065	0.341155
MIR641	1.334129	0.341123
KCNQ1	-0.31565	0.341093
UNC5B-AS1	0.62745	0.341051
ARL1	0.106021	0.341047
QRICH1	-0.08352	0.340995
AL773545.3	0.38095	0.34098
AP001486.2	-0.2451	0.340946
CREB3L3	0.892895	0.34091
NA	1.834627	0.340893
ZNF22	0.104229	0.340807
MSANTD4	-0.13595	0.340751

HOTAIR	-0.30885	0.340722
USP46	-0.1209	0.340671
PELO	-0.14232	0.340669
AC133550.1	-0.19025	0.340608
AL929472.3	-0.13226	0.340596
AL031600.3	0.144137	0.340556
BHMT	-1.07016	0.340553
CTSA	-0.10632	0.340551
AC008481.2	0.86932	0.340538
AL606489.1	1.477725	0.340513
PDCD1LG2	0.479249	0.340391
CA13	0.934869	0.340359
ADD1	-0.09006	0.340315
NUS1	0.09269	0.340262
AL596244.1	-0.20378	0.340262
ROCK1	-0.10784	0.340229
CXorf40A	-0.20927	0.34018
SECTM1	0.631022	0.340172
RUNDC3A	0.124165	0.34015
RF00019	1.00794	0.340124
AC087273.1	0.41711	0.340098
CATIP	-2.1571	0.34009
AC084754.1	-2.1571	0.34009
LINC00662	-0.14182	0.340056
SLAIN1	0.138296	0.340047
MSTO2P	-0.17477	0.340026
ARHGAP9	-0.34625	0.339994
RASA4CP	0.279021	0.339994
ANKRD44	-0.12146	0.339953
ATXN7L3	-0.09045	0.33992
AL354892.1	0.529717	0.339907
C1QTNF1	0.151858	0.339846
PARG	0.094858	0.339799
PLSCR1	-0.59075	0.33976
DMD	0.324278	0.339724
RAF1	-0.08995	0.339723
USP40	-0.1151	0.339656
AK8	-0.55344	0.339586
AC116025.2	-1.46258	0.339518
SLC26A5	0.777014	0.339443
JRK	-0.0889	0.339406
FAM182B	-0.48787	0.339226
NA	-1.78492	0.339077
RLF	0.120826	0.339073
AP000526.1	0.33963	0.338993
AP000346.2	-0.84946	0.338946
ADCY3	-0.09294	0.338943
AC091564.4	-0.18114	0.338907
RPL7P26	-1.49754	0.338906
THEMIS2	-0.24775	0.338891

ATAD3C	-1.29865	0.338862
AC015909.1	-0.76467	0.338847
RAB11FIP3	-0.09729	0.338794
AC133485.3	1.262175	0.338783
DCBLD2	-0.1006	0.338775
SLC26A7	-1.86084	0.338713
AC106779.1	-1.86084	0.338713
LINC00339	-0.16356	0.3386
MEIS3P1	0.250044	0.338563
ABI2	-0.07716	0.338444
SLC4A11	-0.25927	0.338399
ZNF215	1.130092	0.338398
WEE1	0.089177	0.338311
AC080162.1	-0.60828	0.338268
ARMC9	0.099577	0.338257
ABCA8	1.537591	0.338203
AC092338.3	-1.51099	0.33819
PPP4R4	-0.25233	0.338172
AC084855.2	-0.96019	0.338093
AL049637.1	1.551767	0.338091
ZYG11B	-0.09215	0.338066
AC104809.2	-2.16457	0.338059
BX679664.3	0.304868	0.337999
MYLK4	0.419796	0.337995
ST8SIA5	1.672012	0.33798
AC022201.2	0.197155	0.337948
PRCD	-0.11707	0.337861
E2F6	0.096701	0.337848
QSER1	-0.08284	0.337817
ATP10A	2.761326	0.337657
RF00019	2.761326	0.337657
RNU6-125P	2.761326	0.337657
RBBP4P4	2.761326	0.337657
LINC00578	2.761326	0.337657
RN7SL364P	2.761326	0.337657
NA	1.682377	0.337513
GIT1	-0.09574	0.337489
PRLR	0.182369	0.337456
NA	0.724333	0.337308
BSN	-0.1222	0.337305
AC096637.2	-1.14507	0.33725
DDX25	0.301751	0.337236
AL035411.1	1.469634	0.337213
NGF	-0.45841	0.337139
NA	-0.67141	0.337091
MRAP	-0.2216	0.337071
AL590708.1	0.555133	0.337069
PCP4L1	-1.78815	0.337029
STK38	-0.10757	0.336973
FAM210A	0.124157	0.336858

F12	0.147705	0.3367
ST3GAL3	-0.17177	0.336668
RASSF7	-0.19769	0.336619
EIF1P6	0.80913	0.336599
AC015726.1	-0.5594	0.336468
TPK1	-1.35467	0.336455
NA	-0.11457	0.336369
AC115618.2	0.243688	0.336299
AL592293.2	1.644955	0.336251
CDK2AP1	0.081334	0.336187
REEP4	-0.13188	0.336134
ZNF444	0.106894	0.336095
E2F7	-0.17907	0.336082
NCAPD2	-0.08295	0.33604
MTHFSD	0.173612	0.336035
ZNF34	0.237952	0.335994
AC138207.2	0.302951	0.335983
AL135925.1	-0.25968	0.335976
CGNL1	-0.38738	0.335975
CCDC59	0.121638	0.335956
PPEF2	-2.15704	0.335942
NA	-2.15704	0.335942
LRCH1	0.09205	0.33594
AL357033.1	1.752414	0.335882
SEPT14P4	-0.99219	0.335871
GAL3ST1	0.454469	0.335853
BTBD9-AS1	2.162714	0.335848
AC087385.1	2.162714	0.335848
AP002812.4	2.162714	0.335848
LINC02427	2.162714	0.335848
HPS4	-0.0969	0.335828
AK1	-0.19504	0.335778
AC092614.1	-0.39483	0.335757
ARSK	-0.15409	0.335725
CCNT2-AS1	0.384847	0.335694
AL449106.1	0.715432	0.335676
NA	-1.53277	0.335614
RFC5	0.100463	0.335585
LINC01547	-0.28013	0.335581
FDX1P1	-2.28315	0.33557
AL592310.1	-2.28315	0.33557
LINC02028	-2.28315	0.33557
NA	-2.28315	0.33557
SLC35A3	-0.15024	0.335552
AL031600.1	0.378652	0.335478
TAF1B	0.115144	0.335442
AL354824.1	1.026357	0.335439
WNT4	-1.30458	0.335405
AL049840.2	-0.25603	0.335377
AC104779.1	-1.0963	0.335374

AL358781.1	-0.1381	0.335369
FAM53C	-0.09082	0.335357
PRRC2B	-0.10253	0.335344
PPAN	-0.20103	0.335336
LPGAT1	-0.08084	0.335308
FBXO24	-0.34867	0.335251
OCEL1	0.220347	0.335225
AP002892.1	0.743051	0.335225
TLNRD1	0.110042	0.335145
DNAH9	-2.16454	0.335143
IL6	-2.16454	0.335143
TWISTNB	0.127204	0.335126
CCNK	-0.10278	0.335091
RHOQ	0.081214	0.334916
PHKA1	0.108582	0.334909
HNRNPF	0.078396	0.334874
SLC35A4	-0.0878	0.334867
AKAP6	0.100827	0.334863
PDPK1	-0.10023	0.334838
ZCCHC4	-0.18803	0.334758
AC024270.3	0.391047	0.3347
OCRL	-0.08366	0.33469
AL583856.2	-1.88076	0.334688
AP1M1	-0.08277	0.334687
FHAD1	-0.3628	0.334625
KLHL36	-0.13104	0.334606
PCDHB18P	0.520685	0.33458
SERPINB6	0.096266	0.334505
ZNF3	0.084265	0.334501
TCAF2P1	-1.52205	0.33448
TMEM187	-0.2899	0.334303
MAP4K5	-0.08327	0.334256
LRP1-AS	0.488341	0.334165
TAF9B	-0.13837	0.334154
C1QTNF6	-0.11178	0.334147
AC099489.3	-1.28266	0.334111
CROCC	-0.11772	0.333981
AL512422.1	0.92369	0.33394
THUMPD1	0.093932	0.333925
AL355388.2	0.45351	0.333872
FAIM	-0.2185	0.333765
SH3BP5-AS1	-0.1141	0.333743
SENP7	0.129541	0.333742
NA	-0.22175	0.333674
SNORD69	0.761533	0.333665
HIST1H4H	-0.23544	0.333629
TNNT2	0.448016	0.333628
NPHP1	0.238256	0.333624
AL137009.1	-0.36394	0.333616
ZNF281	-0.09043	0.333583

CAMK2A	1.077566	0.333549
PIM1	0.130584	0.33353
AL445470.1	1.854422	0.333481
TLE6	0.376984	0.33347
TMPOP2	0.916835	0.333466
CNOT10	0.093255	0.333444
GPBAR1	-0.768	0.33341
ZNF724	0.24022	0.333365
C12orf65	-0.10352	0.333278
CAMK1	0.121442	0.333274
HK1	-0.07693	0.333269
AC027271.1	-0.92799	0.333215
NA	0.253099	0.333211
BST2	0.307767	0.333203
AL139288.1	0.492554	0.333148
ARHGAP22	0.242137	0.333112
RPS6P25	0.678985	0.333097
CTHRC1	-0.27155	0.333073
TMPRSS11A	-2.157	0.333027
RNU4-8P	-2.157	0.333027
SH3D21	0.148054	0.333015
OBP2A	-0.86115	0.333014
AC006435.2	0.132535	0.333001
TRIM9	0.243387	0.332904
MIR645	2.162551	0.33289
AC069307.1	2.162551	0.33289
NA	-0.1934	0.332862
ELOCP19	-0.76629	0.33283
FAR2P1	-2.2833	0.33283
RN7SL274P	-2.2833	0.33283
AL133338.2	-2.2833	0.33283
MTA2	-0.08093	0.332824
AC120024.1	-1.00938	0.332754
AL160314.2	1.15295	0.332739
PCCA-AS1	2.162541	0.332708
ABCA2	-0.10225	0.332694
WFDC1	-0.69314	0.33269
SEMA5A-AS1	-1.56187	0.332641
AC078795.1	-0.13259	0.33259
NOP56P1	1.632596	0.332561
KANK2	-0.11768	0.332449
AIFM3	-0.47736	0.332383
C22orf46	-0.15135	0.332373
AC007000.2	-1.87273	0.332307
IDI2	-0.23429	0.332301
NPM1	0.080872	0.33229
XIAP	0.107368	0.332274
DNAJC22	0.453622	0.332271
STIMATE-MUSTN1	-1.43791	0.332245
ISCA1P4	-1.38095	0.332061

CRYBB2P1	0.141042	0.332041
PRADC1	-0.17015	0.331977
RACGAP1	0.101976	0.331973
AC024940.4	1.80535	0.331931
AL590762.1	0.887316	0.331926
NA	-0.29649	0.331834
AL050320.1	1.384819	0.33183
RPS3AP26	0.292747	0.331743
NA	-2.12843	0.331741
SNORD116-24	-1.30773	0.331733
CAB39	0.0867	0.331722
AC010809.2	0.420542	0.331685
CCDC87	-0.84592	0.331664
Z95152.1	-0.71681	0.331643
SLC25A39P1	1.197171	0.331615
RNU6ATAC9P	-2.12653	0.331515
PHBP9	0.56585	0.331435
AL022068.1	1.009981	0.331367
GLIS3	-0.28179	0.331361
AC131097.3	-1.35247	0.33135
PHF23	-0.07582	0.331283
LINC01355	0.170729	0.33128
SUMF1	-0.13483	0.331155
CXCL6	-0.63196	0.331144
GPATCH1	0.131418	0.331127
RAD23A	-0.08406	0.331119
AC022905.1	-0.58618	0.331043
DESI1	-0.12007	0.331035
NA	-1.03398	0.330988
AC068790.3	0.601465	0.330944
PQBP1	0.100502	0.330846
AXL	-0.37594	0.330835
SH3KBP1	-0.12428	0.330812
TIGD3	-0.49797	0.330791
FCGRT	0.125816	0.33078
NOCT	0.242564	0.33078
LIG1	-0.09987	0.330719
AC104938.1	-0.25565	0.330706
GADD45B	0.254587	0.330613
AL162726.3	1.133461	0.330604
MRPL43	-0.09186	0.3306
RETREG2	-0.08265	0.330541
AC010619.2	-1.42001	0.330513
RPS12P17	1.840855	0.330476
C1orf167	0.241228	0.330456
CCN1	-0.13833	0.330376
SCAP	-0.10483	0.330368
PDLIM1	-0.14264	0.330353
AC104699.1	-0.38246	0.330244
ABCA17P	0.597014	0.330201

AC104073.1	-1.65372	0.330197
LINC01184	0.147001	0.330195
NA	-0.5045	0.33016
SERBP1	0.075136	0.330016
ZNF740	0.098252	0.329938
AAAS	0.097238	0.329896
H3F3B	0.069266	0.329847
PREPL	0.089186	0.329831
TFAP2D	2.882089	0.329819
ETV7	2.882089	0.329819
ZC2HC1B	2.882089	0.329819
IL17C	2.882089	0.329819
CYP2G1P	2.882089	0.329819
GRID2	2.882089	0.329819
CD96	2.882089	0.329819
OTOA	2.882089	0.329819
HTRA3	2.882089	0.329819
LINC01869	2.882089	0.329819
LHFPL1	2.882089	0.329819
NA	2.882089	0.329819
DUSP27	2.882089	0.329819
RNU6-1020P	2.882089	0.329819
LINC02487	2.882089	0.329819
C1orf146	2.882089	0.329819
CARD16	2.882089	0.329819
VGLL3	2.882089	0.329819
MIR149	2.882089	0.329819
MIR580	2.882089	0.329819
RNA5SP219	2.882089	0.329819
HNRNPA1P64	2.882089	0.329819
CTAGE11P	2.882089	0.329819
RPL31P1	2.882089	0.329819
CCNB2P1	2.882089	0.329819
XRCC6P5	2.882089	0.329819
NA	2.882089	0.329819
RPS16P5	2.882089	0.329819
CACYBPP3	2.882089	0.329819
BTBD10P2	2.882089	0.329819
MIR1302-5	2.882089	0.329819
RNU4-53P	2.882089	0.329819
AL683807.1	2.882089	0.329819
AC093107.1	2.882089	0.329819
MTND2P5	2.882089	0.329819
MTCYBP21	2.882089	0.329819
NA	2.882089	0.329819
NA	2.882089	0.329819
AC091492.1	2.882089	0.329819
AL513314.1	2.882089	0.329819
TLK1P1	2.882089	0.329819
LINC01630	2.882089	0.329819

NA	2.882089	0.329819
AC010240.1	2.882089	0.329819
MAGEA4-AS1	2.882089	0.329819
LINC01098	2.882089	0.329819
KLF2P1	2.882089	0.329819
OR55B1P	2.882089	0.329819
CT69	2.882089	0.329819
LINC01880	2.882089	0.329819
RPL22P23	2.882089	0.329819
AL357134.1	2.882089	0.329819
LINC01432	2.882089	0.329819
NA	2.882089	0.329819
AL031275.1	2.882089	0.329819
AC025750.2	2.882089	0.329819
AL163193.1	2.882089	0.329819
AC090192.1	2.882089	0.329819
LINC01471	2.882089	0.329819
RN7SL368P	2.882089	0.329819
AC104763.1	2.882089	0.329819
AC092691.3	2.882089	0.329819
RN7SL269P	2.882089	0.329819
LINC02071	2.882089	0.329819
AC092490.1	2.882089	0.329819
NA	2.882089	0.329819
RNA5SP330	2.882089	0.329819
AC011008.1	2.882089	0.329819
AC136475.4	2.882089	0.329819
AC084032.1	2.882089	0.329819
AC092375.1	2.882089	0.329819
AC012038.2	2.882089	0.329819
AL355102.1	2.882089	0.329819
SPECC1L-ADORA2A	2.882089	0.329819
AC087641.1	2.882089	0.329819
AC012291.1	2.882089	0.329819
AC104316.2	2.882089	0.329819
AC013355.1	2.882089	0.329819
LINC02673	2.882089	0.329819
ARHGAP23P1	2.882089	0.329819
MIR4436B1	2.882089	0.329819
AC011933.4	2.882089	0.329819
RN7SL220P	2.882089	0.329819
AC011990.1	2.882089	0.329819
WDR7-OT1	2.882089	0.329819
IGFL2-AS1	2.882089	0.329819
AC022432.1	2.882089	0.329819
BX546450.2	2.882089	0.329819
AC005520.4	2.882089	0.329819
AC106791.2	2.882089	0.329819
FXYD6P1	2.882089	0.329819
AC091946.2	2.882089	0.329819

AC103810.8	2.882089	0.329819
ITGA1	0.084051	0.329811
NA	-0.31933	0.329807
FBXW4	0.13947	0.32978
RAD51AP2	0.589244	0.329772
NEO1	-0.09016	0.329751
DCTN5	-0.07567	0.329718
ZNF587	0.096559	0.329703
NDST1-AS1	0.352312	0.329686
ASCL5	0.63632	0.329641
ZNF621	0.093426	0.329626
HIF1A-AS2	1.058241	0.329604
NTAN1P2	-0.59322	0.329603
LRRC37A6P	0.528675	0.329584
BROX	-0.09303	0.329505
AC011479.2	1.01507	0.329445
SRP14	0.072201	0.329426
AC104806.1	-1.32011	0.32942
PLAAT1	0.478792	0.329348
IRS2	-0.10089	0.329336
KATNBL1P4	1.444288	0.329319
RPSAP16	-2.13024	0.329288
AC011405.1	-0.33198	0.329214
NA	-0.77112	0.329198
LINC01503	1.514425	0.329171
ETAA1	-0.12534	0.32917
FAM120AOS	0.095005	0.329145
NA	1.213997	0.329082
RNA5SP37	1.729873	0.329064
PHF1	0.106974	0.329049
CACNB1	0.144637	0.329046
BRI3	0.118198	0.329008
ELOVL7	-0.46709	0.329002
SUCNR1	-0.55859	0.328967
FOXI3	-2.13214	0.328963
FNTB	0.191624	0.328879
COL9A2	0.257365	0.328855
AC096677.2	-0.3722	0.328772
GAPDHP62	-2.12832	0.32875
MIR544B	-2.12832	0.32875
NME2P1	-0.87596	0.328746
SLC25A1P5	0.338264	0.328669
AC109635.2	0.691419	0.328659
FGF17	0.753757	0.328643
ANKRD19P	-0.54131	0.32863
RBM44	0.653695	0.328597
AC020779.2	0.782594	0.32857
USP11	-0.09028	0.328534
RDH10	0.176703	0.328491
AL139022.1	0.168757	0.328489

ILKAP	0.120256	0.328484
NA	1.147885	0.32846
SGMS2	1.621959	0.328455
AC132942.1	0.781714	0.328433
CC2D2A	-0.1464	0.328424
ADGRG2	0.577995	0.328422
RPS15AP12	-1.12637	0.328412
RGL2	-0.09742	0.328318
SEPT7-AS1	-0.3799	0.328281
NA	0.191871	0.328254
FNDC3B	-0.10058	0.32816
TAFA4	0.499815	0.328142
RF00019	1.809855	0.327987
FAM98A	-0.09594	0.32798
RHOA	-0.07002	0.327894
AC005786.3	0.227577	0.327682
PRKG1-AS1	-0.33207	0.327546
VGF	0.094994	0.327512
CSF2	-0.80576	0.327475
PPIAP11	0.921945	0.327446
ITPR3	-0.15095	0.327439
AC092072.1	-0.27744	0.327409
STK40	-0.11942	0.327395
TMEM144	-2.12637	0.327371
RPS7P14	-2.12637	0.327371
TMEM14EP	-2.12637	0.327371
NA	-0.41075	0.327334
NA	0.191226	0.3273
LARP4B	-0.08281	0.327197
AC011603.2	0.064803	0.327188
AL078644.1	1.177676	0.327183
C2orf66	-1.64383	0.327114
NEDD9	0.153187	0.327111
SLC18A2	-0.21051	0.327065
AL512625.3	0.302736	0.327036
DPY19L2P1	0.329988	0.326896
AC130324.2	-0.53486	0.326863
AC005759.2	-0.57444	0.326818
NFATC3	0.089438	0.326779
TANK	-0.10035	0.326732
PIR	0.133372	0.326703
CLDN1	0.874303	0.326698
SPATA2	-0.14478	0.326678
GNAQ	-0.09259	0.326617
FAM66B	-0.53695	0.326559
PANK1	0.126557	0.326481
UAP1	0.107942	0.326443
NA	1.632536	0.326422
AC008764.7	-1.12157	0.326411
ANKRD26P4	-1.25565	0.326404

TUT1	-0.13847	0.326319
MEMO1P1	0.909322	0.326315
SLC38A4	-0.40795	0.326148
LIN7C	0.117399	0.32608
ADGRD1	-2.13204	0.326049
PTMAP8	-2.13204	0.326049
AC055811.3	-2.13204	0.326049
VGLL4	0.073163	0.32604
IL11RA	0.160316	0.325934
EML2	0.173572	0.325893
ZNF442	0.676937	0.325873
AP000320.1	0.983086	0.325863
FAM95B1	-1.45502	0.325726
UBE2M	0.08817	0.325719
SYCP2L	0.215754	0.325681
CEP85	-0.10228	0.325665
TRIM44	0.095244	0.325663
CSPP1	-0.12737	0.325658
HGH1	-0.30138	0.325646
ECM1	-0.24513	0.32562
SCD5	0.117709	0.325567
DHX30	-0.07742	0.325543
AC125807.2	-0.18377	0.325515
SPG11	-0.08773	0.325494
PIK3AP1	-0.19632	0.325484
NA	0.674313	0.325478
SNX24	-0.14746	0.325439
LRRC74B	1.100464	0.325375
IK	0.078695	0.325338
CCM2	-0.09929	0.325317
NA	-0.52957	0.325144
NA	1.782179	0.325143
AC064836.2	1.782179	0.325143
SNX10	0.220759	0.325077
ADNP	-0.07469	0.325062
AC125611.3	0.342953	0.325022
AIP	0.098541	0.324995
HOXC-AS1	0.589727	0.324923
OAS3	-0.10896	0.32485
AC002398.2	1.478691	0.32485
AASS	0.407466	0.324821
AP003469.4	-0.44768	0.324817
GOLIM4	-0.09225	0.324806
EIF5	0.070271	0.324729
AC005523.1	1.508721	0.324668
GUSBP3	-0.17823	0.324601
SOCS5P4	-0.89736	0.32458
NR5A2	-1.91504	0.324507
FAM185BP	0.316553	0.324488
PANK4	-0.14061	0.324458

NA	-2.12627	0.324458
RNU6-1008P	-2.12627	0.324458
C18orf21	0.126902	0.32443
ATP5PB	0.089174	0.324348
ADTRP	0.876627	0.324343
ZNF25	0.122301	0.324333
SACS-AS1	-1.08327	0.324318
ADORA2B	-0.40068	0.324292
CD6	-0.77339	0.324234
GNGT1	0.16096	0.324234
EEFSEC	-0.12583	0.324109
SLC25A17	0.108978	0.324105
CHCHD6	0.137057	0.324034
HLA-W	-1.44996	0.32403
ZBTB25	0.092587	0.324028
RGMB-AS1	0.334158	0.32391
SAPCD1-AS1	-0.34074	0.323908
NDUFS5	0.085585	0.323888
EIF5AP4	-0.76145	0.323834
PLOD3	-0.08632	0.323808
B3GNT4	0.101723	0.323745
RN7SL608P	-1.12022	0.323741
UPF3A	-0.08252	0.32374
FAM155B	-0.29827	0.323733
HOXD8	-0.1338	0.323699
CBY3	0.866064	0.323533
TTF2	-0.11779	0.323524
BTF3L4P2	-0.3753	0.323506
C17orf75	0.106117	0.323426
AC012513.2	-0.956	0.323397
SRP68P3	-0.676	0.323393
ADGRB1	0.160726	0.323309
GDPD2	-0.81535	0.323289
AC011487.1	2.117547	0.323243
AC010627.1	2.117547	0.323243
THRA	0.093667	0.323202
RPP40	0.180829	0.323095
RBM4B	-0.10065	0.32301
MDN1	-0.08078	0.323006
FGFR2	0.225943	0.322988
DENND6A	-0.10696	0.32293
MARK2	0.103565	0.322906
AF233439.1	-0.43054	0.322877
AC104772.1	2.242769	0.322852
AC005014.2	2.242769	0.322852
CYFIP2	-0.12877	0.32284
C3orf18	0.148167	0.322798
REM1	-0.88611	0.322787
THAP5	0.100385	0.322772
MMGT1	0.110855	0.322673

MGARP	1.279725	0.322672
MIR210HG	-0.46973	0.322629
C15orf61	-0.17599	0.322623
FAR2	0.122642	0.322594
ZSCAN21	0.113878	0.322552
GINS1	0.126713	0.322547
BNC2	-0.20693	0.322477
RPL11P3	0.762532	0.322343
ZNF114	-0.34726	0.322322
PIP4P2	-0.13175	0.322158
MIGA1	-0.10943	0.322154
AC026771.1	0.180423	0.32212
ALDOA	-0.06902	0.322075
KLHDC9	0.586814	0.322062
NORAD	-0.0725	0.322052
FMOD	1.461089	0.322035
AC108479.1	1.695412	0.322024
VPS37B	-0.11794	0.32202
UBE4B	-0.09213	0.322004
CCT6P1	0.137086	0.321992
UHRF1BP1	0.11428	0.321966
AC010680.5	0.677545	0.321886
TSN	-0.08144	0.321773
RWDD1	0.096623	0.321758
NA	-0.92121	0.321755
TTL	-0.08356	0.321751
PPP1R3C	-0.2171	0.321665
AC009303.2	0.33784	0.321626
HVCN1	0.343777	0.32153
AC018797.2	1.809286	0.32152
NA	1.809286	0.32152
RNF41	0.086015	0.321505
GABBR1	0.132204	0.321492
SNRPD1	0.087519	0.321457
AL122010.1	-0.40228	0.321363
AC091729.3	0.220381	0.321325
AC093495.1	1.180516	0.321297
AL365181.3	-0.39919	0.321282
AC000068.2	-0.14984	0.32126
FECH	-0.10509	0.321231
AL138762.1	0.392941	0.321135
AL512310.10	-1.76991	0.321078
GATD3A	0.0795	0.321078
AC011472.1	1.032844	0.320941
SKA3	-0.13866	0.320891
AC020915.1	0.555533	0.320888
SHOX2	-0.11229	0.320882
C6orf118	-0.66304	0.320866
MESP2	-1.67816	0.320843
PPM1M	-0.16161	0.320808

TMC7	0.457521	0.320792
MAP7	-0.12057	0.320651
MMP23B	1.060867	0.320627
CDCA4P1	1.071541	0.320584
NA	-0.17207	0.320579
MAGT1	0.102918	0.320512
MCPH1	0.127034	0.320491
AC116407.1	-0.26008	0.320444
FHOD3	-0.118	0.320409
AC012063.1	0.390765	0.320404
SNORA5C	-0.4625	0.320358
CNN2P1	2.117265	0.320285
AL137779.1	2.117265	0.320285
CAPN12	-0.12536	0.320252
ZNF749	0.170457	0.320222
AC011330.1	1.055927	0.320166
FAM86DP	0.178703	0.320163
RF00019	1.01169	0.320146
AC011477.3	0.348519	0.320123
TMEM65	0.134298	0.320116
LARGE1	0.30688	0.320112
DLG3-AS1	2.117248	0.320102
NA	2.117248	0.320102
PABPC1P7	2.117248	0.320102
LINC01539	2.117248	0.320102
AL356019.1	-0.3006	0.320063
NCAM2	0.316697	0.320061
TUBGCP3	-0.10167	0.320023
NELFB	-0.08941	0.319986
LHX8	-0.47696	0.319979
KDM4D	0.294425	0.319938
LDHC	2.242737	0.319909
AP006296.1	2.242737	0.319909
USP25	-0.11981	0.319905
S100A10	-0.31094	0.319902
GUSBP9	0.330432	0.319895
CRKL	-0.08507	0.319872
LRRC37A3	0.133132	0.319794
TTC3-AS1	-0.18003	0.319774
ERLIN1	-0.0957	0.31976
PLOD1	0.086144	0.319735
LTK	-0.37729	0.31967
HAP1	1.163604	0.319628
POGLUT3	-0.28641	0.319503
AGPAT1	-0.08232	0.319487
ERN2	-0.71982	0.319435
LINC01844	-1.13209	0.319423
ATP2B2	-0.23856	0.319321
APPL2	0.080521	0.319311
FSIP1	-0.47831	0.31929

KLHDC1	-0.37503	0.319275
SYT3	-0.2516	0.319272
DHX29	0.110617	0.319195
ZNF32-AS1	0.162025	0.31914
DDX10	-0.12963	0.31911
SMC5-AS1	-0.74717	0.319064
MPP2	0.156412	0.318924
RAB42	0.373977	0.318896
PRR22	-0.23876	0.318836
OGT	0.08187	0.318833
AHI1	0.104113	0.318811
AC012435.2	-1.09226	0.318782
SSR3	0.085961	0.318764
TMEM200C	0.344868	0.318747
BEST2	1.985563	0.318734
NIM1K	1.985563	0.318734
KATNBL1P6	1.985563	0.318734
AL137798.1	1.985563	0.318734
PCOLCE2	0.187517	0.318634
VEZT	0.094529	0.318603
SLC30A1	-0.09268	0.318518
VPS51	0.107282	0.318487
SPTY2D1	0.097298	0.31842
INKA1	0.274404	0.318382
RNF144B	-0.3724	0.318337
YIPF2	0.116995	0.318337
DNAAF3	-0.49724	0.31833
LINC01816	0.401818	0.318318
AL080243.2	0.520904	0.318288
NPIPB5	0.188212	0.318259
KIAA0513	0.229217	0.31825
MTMR9LP	-0.33016	0.318216
NA	0.736455	0.318159
ANXA2	-0.21907	0.318149
NA	-0.19383	0.318088
CYB561A3	-0.09316	0.318075
C14orf178	0.287366	0.318072
ZNF614	-0.11767	0.318067
UBP1	-0.07608	0.318067
LOXL1	1.593921	0.318055
AC017076.1	-0.4595	0.318048
AC121761.1	-1.01879	0.318028
AC115223.1	-0.20305	0.318017
LINC002481	1.602966	0.318011
AP000525.1	-0.15455	0.317972
AP000866.1	-0.37131	0.317971
LINC01011	0.337965	0.317957
LYG1	-0.79499	0.31782
DNMT3L	-1.70158	0.317748
ADGRB3	-0.11465	0.317667

DUSP8	-0.20668	0.317646
AP000944.1	1.257456	0.317642
RAP1GAP2	-0.10524	0.317589
KIFAP3	-0.09531	0.317588
GSPT2	-0.12606	0.317543
RAP2C-AS1	0.255383	0.317496
SETD6	0.097393	0.317485
EGLN3	-0.13056	0.31747
ATP13A5	1.798561	0.317454
NA	1.798561	0.317454
AL139100.1	0.698002	0.317428
PURB	-0.06918	0.317425
OR7E136P	0.786397	0.317421
NBPF3	-0.13471	0.317353
LTBP3	-0.08575	0.317346
VAPA	0.075708	0.317332
ALDH18A1	-0.08041	0.317309
BAIAP3	-0.19331	0.317142
PIAS4	0.106424	0.317108
SUFU	-0.11357	0.317001
PHF8	-0.10757	0.316982
PRKACB	0.099896	0.316961
SPDYE16	1.075942	0.316954
CHST13	0.714794	0.31695
COPRS	-0.11573	0.316885
GOLGA2	0.098831	0.316868
AP001922.5	1.139121	0.316751
ARMH3	0.108234	0.316719
NA	1.66395	0.316705
FEM1B	0.07532	0.316696
LINC00106	0.454909	0.316656
VSTM2A	-0.28419	0.316646
ZDHHC3	-0.08311	0.316612
APBB1	-0.11364	0.316576
AC036214.2	-1.14047	0.316569
CSRP1	0.103261	0.316536
AL135818.2	-0.97627	0.316499
AC078795.3	0.447375	0.316363
TCF3	-0.08326	0.316321
RN7SL481P	0.811734	0.316285
SDHD	-0.15051	0.316212
PAK1IP1	0.106513	0.316147
SERTAD3	-0.13942	0.316145
POMGNT1	0.080677	0.316105
ELOVL4	-0.16943	0.316049
G6PC2	1.790912	0.316035
ZFAT-AS1	-0.4407	0.315933
AC005481.1	0.781325	0.315901
ATP1B1	0.063166	0.315837
SCN1B	0.4896	0.315805

AC023796.2	0.225785	0.31576
GCSAM	0.962476	0.315743
RSPH14	0.294358	0.315739
AC107993.1	1.985743	0.315734
NA	-0.62737	0.31573
SLC39A10	0.083143	0.315728
MMP19	-0.18803	0.31569
AC007098.1	-0.1523	0.315687
AC026801.2	0.610952	0.315671
LMNB1-DT	-1.58805	0.315668
HDHD2	-0.08915	0.315663
SLC38A9	0.116681	0.315628
MT1X	-0.33048	0.315627
SNORA71A	0.774022	0.315616
AC092718.3	1.035557	0.315597
AC009022.1	0.376689	0.315529
AP001062.1	-0.18373	0.315387
NRG3-AS1	0.72114	0.315325
AL391121.1	0.185236	0.315289
GART	0.073382	0.3152
STAU2	0.105906	0.315181
SMAD1	-0.11357	0.315163
NR2C2	-0.09892	0.315129
TRIM13	-0.09924	0.314993
MTOR-AS1	0.27886	0.314948
MAFG	0.094681	0.314925
FZD4	-0.21559	0.314915
GSAP	-0.17475	0.314838
AC244090.1	0.468376	0.31483
AC007493.2	-0.29563	0.314796
MT2A	-0.13417	0.314772
AC011933.1	1.080099	0.314763
HGSNAT	-0.10454	0.314725
PGAM1P8	-0.95499	0.314698
ARID1A	-0.0999	0.314668
UBE2K	0.085227	0.314665
BRINP1	-0.33026	0.314662
AC018362.2	-0.21407	0.314659
AL355297.4	-0.49106	0.314638
DHX40	0.08323	0.314613
SMPD1	-0.1115	0.314566
AC120053.1	0.278757	0.314464
UNK	-0.08098	0.31441
SLC48A1	-0.15645	0.314389
IL17D	0.245493	0.314309
CLDN18	0.415843	0.314212
ADNP2	-0.09012	0.314199
NA	0.344218	0.314111
SUPT5H	-0.08203	0.314092
RNF144A	-0.07322	0.314054

TMEM74B	-0.34254	0.314054
RANBP6	-0.09683	0.314043
ZNF785	0.131419	0.313997
AC141586.2	0.472944	0.31397
NA	1.89917	0.313943
TANGO2	-0.16333	0.313855
EFNB2	-0.12013	0.313812
KLHL7-DT	0.539863	0.313757
FMO3	-2.77182	0.313707
RGS1	-2.77182	0.313707
SLC17A1	-2.77182	0.313707
EPO	-2.77182	0.313707
TNNT3	-2.77182	0.313707
MDFIC	-2.77182	0.313707
LAD1	-2.77182	0.313707
EN1	-2.77182	0.313707
LRATD2	-2.77182	0.313707
TNFRSF10D	-2.77182	0.313707
NA	-2.77182	0.313707
FAM230E	-2.77182	0.313707
OTOG	-2.77182	0.313707
RN7SKP243	-2.77182	0.313707
RF00019	-2.77182	0.313707
MIR613	-2.77182	0.313707
TRAV18	-2.77182	0.313707
RNU6-918P	-2.77182	0.313707
NA	-2.77182	0.313707
NA	-2.77182	0.313707
DCAF8L1	-2.77182	0.313707
UBE2V1P1	-2.77182	0.313707
AL450063.1	-2.77182	0.313707
RPL12P49	-2.77182	0.313707
SPATA31C1	-2.77182	0.313707
HHATL-AS1	-2.77182	0.313707
NA	-2.77182	0.313707
AL353743.3	-2.77182	0.313707
AC234782.2	-2.77182	0.313707
AL050344.1	-2.77182	0.313707
RPSAP31	-2.77182	0.313707
NA	-2.77182	0.313707
CTSLP8	-2.77182	0.313707
RPL21P135	-2.77182	0.313707
POU5F1P5	-2.77182	0.313707
HNRNPA1P22	-2.77182	0.313707
LINC00427	-2.77182	0.313707
AL449403.1	-2.77182	0.313707
AC022173.1	-2.77182	0.313707
HOXA11-AS	-2.77182	0.313707
AC092910.2	-2.77182	0.313707
RN7SL517P	-2.77182	0.313707

AC107027.1	-2.77182	0.313707
AC098799.1	-2.77182	0.313707
NCOA4P4	-2.77182	0.313707
ALG1L14P	-2.77182	0.313707
AC026124.1	-2.77182	0.313707
AC005355.1	-2.77182	0.313707
AC008958.1	-2.77182	0.313707
AC092828.1	-2.77182	0.313707
AC018620.1	-2.77182	0.313707
AC022364.2	-2.77182	0.313707
MRPL40P1	-2.77182	0.313707
OR8R1P	-2.77182	0.313707
AC063947.1	-2.77182	0.313707
AL133467.1	-2.77182	0.313707
AC005096.1	-2.77182	0.313707
AC008915.1	-2.77182	0.313707
AC051619.8	-2.77182	0.313707
AC007218.1	-2.77182	0.313707
AC142381.3	-2.77182	0.313707
AC055876.4	-2.77182	0.313707
TCF24	-2.77182	0.313707
MYMX	-2.77182	0.313707
NA	-2.77182	0.313707
AC006116.6	-2.77182	0.313707
NA	-2.77182	0.313707
AC022144.1	-2.77182	0.313707
LINC01235	-2.77182	0.313707
NTAN1P1	-2.77182	0.313707
Z99916.2	-2.77182	0.313707
AC016831.5	-2.77182	0.313707
CFAP206	-2.77182	0.313707
AC009974.1	-2.77182	0.313707
ANKRD27	0.112487	0.313666
NSMCE3	-0.11448	0.313597
AL359918.1	-1.38335	0.313588
AC069544.1	-0.40101	0.313559
NIPSNAP1	0.090299	0.313552
NA	0.124227	0.313536
RCN2	0.074219	0.313532
AL627389.1	-1.25751	0.313529
NF2	-0.11892	0.313479
DENND1A	-0.11764	0.313455
B4GALNT4	0.105878	0.313428
APH1A	0.064713	0.313418
SEC22A	-0.21503	0.313414
CASTOR1	0.940748	0.313236
RP2	-0.12637	0.313206
HYI-AS1	-0.47475	0.313188
AC093535.1	0.496429	0.313148
PIK3C2B	0.136934	0.313135

AC006064.4	0.090185	0.313124
AC005387.1	0.733627	0.31311
MTND1P36	-1.19064	0.313085
XKR8	1.677518	0.313064
ANKRD20A7P	1.677518	0.313064
AC135782.1	1.677518	0.313064
PMEL	-0.12425	0.31305
GFPT1	0.086506	0.312966
PXN-AS1	0.140321	0.312965
AC021237.1	1.506215	0.312933
NA	-1.58883	0.312884
ZBTB39	-0.11748	0.312843
C17orf67	0.28839	0.312812
AL138756.1	-0.20697	0.312776
PCNX3	-0.11315	0.312743
IFT88	-0.13718	0.312712
WFIKKN2	1.492618	0.312669
AL021396.1	1.492618	0.312669
NA	0.489404	0.312559
KRTCAP3	0.272174	0.312559
NA	0.447848	0.312495
AC115618.1	0.15562	0.312489
SYT9	-0.20795	0.31247
HIPK2	-0.10726	0.312451
SCN5A	-0.15127	0.312373
DNM3	-0.20185	0.31232
SOCS4	-0.10108	0.312295
ZAP70	-1.02538	0.312273
CYP24A1	-2.76182	0.312254
HYAL4	-2.76182	0.312254
EFCC1	-2.76182	0.312254
FBP2	-2.76182	0.312254
SLC5A12	-2.76182	0.312254
RTP3	-2.76182	0.312254
SCGB3A2	-2.76182	0.312254
ADGRF2	-2.76182	0.312254
ASB11	-2.76182	0.312254
PKD1L2	-2.76182	0.312254
RBMXL3	-2.76182	0.312254
ZNF804B	-2.76182	0.312254
FUNDC2P2	-2.76182	0.312254
OR1J2	-2.76182	0.312254
RNA5SP379	-2.76182	0.312254
RNU6-921P	-2.76182	0.312254
RNVU1-14	-2.76182	0.312254
MIR491	-2.76182	0.312254
IGHG3	-2.76182	0.312254
AC087491.1	-2.76182	0.312254
CFL1P6	-2.76182	0.312254
RPL10P1	-2.76182	0.312254

NA	-2.76182	0.312254
GSTA8P	-2.76182	0.312254
OR1F12	-2.76182	0.312254
RNA5SP345	-2.76182	0.312254
MTCO1P17	-2.76182	0.312254
RPL7P7	-2.76182	0.312254
LINC01907	-2.76182	0.312254
LINC02608	-2.76182	0.312254
RPS26P56	-2.76182	0.312254
TAS2R46	-2.76182	0.312254
AC119428.1	-2.76182	0.312254
AC010998.1	-2.76182	0.312254
LINC01724	-2.76182	0.312254
AKR1D1P1	-2.76182	0.312254
AL449043.1	-2.76182	0.312254
MACROD2-IT1	-2.76182	0.312254
THAP12P3	-2.76182	0.312254
MTATP6P11	-2.76182	0.312254
AC024937.1	-2.76182	0.312254
AL136097.1	-2.76182	0.312254
ANKRD36BP2	-2.76182	0.312254
ERVMER61-1	-2.76182	0.312254
AP000532.2	-2.76182	0.312254
AL132765.1	-2.76182	0.312254
AP000281.2	-2.76182	0.312254
GXYLT1P3	-2.76182	0.312254
AP001596.1	-2.76182	0.312254
AC016700.3	-2.76182	0.312254
RPS3AP44	-2.76182	0.312254
AL161785.2	-2.76182	0.312254
MRPL53P1	-2.76182	0.312254
AC099796.1	-2.76182	0.312254
ITCH-AS1	-2.76182	0.312254
HMGA1P1	-2.76182	0.312254
YRDCP2	-2.76182	0.312254
AL133244.2	-2.76182	0.312254
NA	-2.76182	0.312254
AL513185.3	-2.76182	0.312254
ATP6V1B1-AS1	-2.76182	0.312254
RN7SL552P	-2.76182	0.312254
AL355864.2	-2.76182	0.312254
AC084024.1	-2.76182	0.312254
TM4SF1-AS1	-2.76182	0.312254
RPL23AP70	-2.76182	0.312254
RPL23AP81	-2.76182	0.312254
ZNF702P	-2.76182	0.312254
RPL23AP55	-2.76182	0.312254
AC010230.1	-2.76182	0.312254
AC097103.1	-2.76182	0.312254
AC034223.1	-2.76182	0.312254

SLED1	-2.76182	0.312254
AC011411.1	-2.76182	0.312254
AC027335.1	-2.76182	0.312254
AC105383.1	-2.76182	0.312254
AC096751.1	-2.76182	0.312254
RNU6ATAC32P	-2.76182	0.312254
IGHEP2	-2.76182	0.312254
BRIX1P1	-2.76182	0.312254
AC044839.4	-2.76182	0.312254
PABPC1P4	-2.76182	0.312254
AL357673.1	-2.76182	0.312254
HP	-2.76182	0.312254
AL928654.3	-2.76182	0.312254
NA	-2.76182	0.312254
AC107241.1	-2.76182	0.312254
AC027139.1	-2.76182	0.312254
AL512274.1	-2.76182	0.312254
AC092120.1	-2.76182	0.312254
NA	-2.76182	0.312254
AC008670.1	-2.76182	0.312254
NA	-2.76182	0.312254
NA	-2.76182	0.312254
AC092316.1	-2.76182	0.312254
AC010323.2	-2.76182	0.312254
AC025278.2	-2.76182	0.312254
AC020910.2	-2.76182	0.312254
AC004987.4	-2.76182	0.312254
AL606495.2	-2.76182	0.312254
AC087501.4	-0.43146	0.312199
CENPVL3	1.078946	0.312143
WHAMM	0.176543	0.312091
UROD	-0.08323	0.312039
YIPF7	-0.51514	0.312038
NA	-0.08446	0.312037
TERF1P5	1.655914	0.312035
AC103810.3	-0.46009	0.311958
SOX7	-0.30411	0.311937
ANKRD36BP1	-0.13468	0.311873
FOXD2-AS1	-1.24795	0.311859
NUP50	-0.08374	0.311851
HS3ST5	-0.2172	0.311842
AC009060.1	-0.10137	0.311779
THOC1	0.095867	0.3117
CDR2L	-0.10417	0.31167
MIR5581	0.662119	0.311603
MALL	0.407383	0.311597
RREB1	-0.19452	0.311559
AC005606.1	0.119099	0.311538
GATB	0.101405	0.311518
SCARB2	-0.07959	0.311483

AC068134.3	-0.57272	0.311432
ZFP69	-0.15787	0.311366
STK4-AS1	-1.60356	0.311341
AC004980.2	-0.65117	0.311277
AC007610.1	0.411646	0.311233
AC106820.2	0.434185	0.311229
FZR1	-0.1152	0.311226
RHCG	0.670892	0.311221
SOCS1	-0.41865	0.311141
AC020892.1	-1.67946	0.311124
POLE	-0.09029	0.311105
APTX	0.125074	0.311004
P2RX5	0.563648	0.310987
RAB3C	-0.11914	0.310983
TFDP1	-0.09276	0.310859
AL671710.1	0.152616	0.310833
AC008649.1	-0.9188	0.310798
AC011495.1	0.488079	0.31079
RPL23A	0.073288	0.310771
RASA4B	-0.38315	0.310753
LGALSL	0.125262	0.310732
ARMC8	0.100271	0.310713
C5orf34	-0.14753	0.310477
GNPAT	-0.08555	0.310449
BTG1	0.095736	0.310428
ACBD3	0.082563	0.310414
B3GNT8	-0.6886	0.310352
KCNC1	0.168335	0.310347
ZKSCAN7	0.115102	0.310312
AC073487.1	-0.81264	0.310288
CRY2	-0.18261	0.310171
SLC30A6	0.079915	0.310166
ENGASE	-0.09819	0.310123
RPL21P28	0.330337	0.310118
EPAS1	-0.1254	0.310048
MIR3187	-1.19833	0.30994
FBXL18	-0.1102	0.309922
HDGFL3	0.071769	0.309782
AC034102.8	0.382718	0.309749
BCKDK	0.099532	0.309723
CA14	0.318971	0.309698
RPL18AP3	0.178671	0.309695
LINC01410	-0.13559	0.309691
SNRPG	0.122268	0.309604
GORAB-AS1	-0.63371	0.30959
ASIC4	0.168082	0.30958
RPL23AP7	-0.25767	0.30951
NA	0.081558	0.309493
AC083867.1	-1.45914	0.309461
UQCC2	0.099414	0.309391

RN7SL403P	-1.5924	0.309356
RPE	0.098673	0.309342
RCOR1	0.100859	0.309324
FAAP20	-0.1022	0.309303
PSMD6-AS2	-0.33827	0.309302
FOXM1	-0.08383	0.309292
MIAT	0.107425	0.309287
FAM120C	-0.13966	0.309283
TACC3	-0.10305	0.309277
AMMECR1	-0.12163	0.309271
STAG2	0.086547	0.309246
RAD51-AS1	-0.16964	0.309217
ZSWIM4	0.133775	0.309213
CREB3	0.085942	0.309154
AC090061.1	0.234133	0.309147
GOLGA8B	-0.08934	0.30914
LDHD	-0.77662	0.309124
AC078802.1	-0.5669	0.309067
TMEM200B	-0.13149	0.309062
AP000317.1	1.328604	0.30902
BX255925.1	0.658171	0.30902
AC069257.2	-1.04501	0.309013
HIP1	-0.09599	0.308963
LINC02412	-1.74688	0.308958
AP006545.1	0.423928	0.308885
EEF1A1P19	0.361846	0.308875
WDR91	-0.13944	0.308836
ATP9B	0.137885	0.308829
AMN1	0.170826	0.308825
DRC3	-0.19078	0.308733
YLPM1	-0.0949	0.308722
ZNF365	-0.2194	0.308686
IGHVIV-44-1	1.766027	0.30868
RPL12P38	-1.7863	0.308643
AC078962.1	-1.7863	0.308643
RPL7AP10	0.613266	0.308617
PLIN2	0.086808	0.308551
PPP1R3F	-0.14387	0.308533
SMAD9	-0.06544	0.308501
C3orf67-AS1	1.085964	0.308497
CRK	-0.0752	0.308463
LINC00667	0.132731	0.308448
HYPK	-0.28624	0.308438
AC084036.1	-0.50741	0.308263
NA	-1.31947	0.308262
ENTR1	0.078021	0.308232
AL353807.2	0.576469	0.308222
RBM23	-0.07728	0.308171
AC010524.1	-1.08639	0.308168
AC008494.3	0.235944	0.308161

AC007899.1	-0.30194	0.308142
AP002812.3	-1.06109	0.308142
NA	-0.20416	0.308136
MIER1	0.096558	0.308136
AL353804.1	0.559907	0.308114
MCM4	0.094824	0.308002
AC087276.1	1.236721	0.307915
ANKZF1	0.088966	0.307874
AC055855.1	0.318174	0.307866
AP000442.2	-0.28242	0.307814
CUL9	-0.09816	0.30781
S100A1	0.288247	0.307789
WAC-AS1	0.102657	0.307751
PI4K2B	0.163903	0.307736
TRIOBP	-0.1567	0.307675
TBCCD1	-0.12534	0.307631
KCNA7	1.878254	0.307606
PLCB3	-0.10864	0.307605
FAF2	-0.072	0.307585
SERINC4	-0.18098	0.307583
PTCD2	-0.17033	0.307576
PPEF1	0.11665	0.30749
IFI44L	0.791857	0.307479
AP006621.2	0.745544	0.307464
AL022329.1	-0.80243	0.307382
MUL1	-0.0937	0.307366
SMU1	0.086791	0.307337
ATP1A1	-0.0817	0.307327
ARHGEF2	0.09811	0.307265
RF00275	1.265194	0.307238
CDC16	0.083979	0.307216
FARSA	0.075488	0.307079
KHSRP	-0.08942	0.306991
AC068831.4	-0.15966	0.306922
RIMS3	0.112739	0.306909
BTF3L4	0.083844	0.306907
ATP2A1-AS1	0.294996	0.306884
AC007292.1	0.116185	0.306855
PPP1R15B	-0.07195	0.306846
PAIP1	0.080357	0.306808
IPO9-AS1	-0.12527	0.306805
PHLDB2	0.196575	0.306788
ZNF559-ZNF177	-0.85201	0.306779
BTBD6	-0.12684	0.306733
NA	0.420289	0.306728
VSIG10L	0.249248	0.306717
NCALD	-0.13936	0.306698
SH3RF3-AS1	0.696726	0.30667
GTF2F1	0.089718	0.306625
AC116552.1	-0.45677	0.306514

NBPF1	-0.07102	0.306461
LINC01963	-0.09726	0.306399
SNAI1P1	-0.80893	0.306392
HMGN2	-0.06934	0.306391
CLCN4	-0.12981	0.30639
IDH1	0.097563	0.306379
AC007314.1	0.137154	0.306331
AC006547.3	-0.2267	0.306255
NA	0.172416	0.306227
AC009927.1	1.870248	0.306226
RNU6-678P	1.870248	0.306226
ABHD17AP4	1.741969	0.306191
TEX41	1.655135	0.306107
SIPA1	-0.10308	0.306053
ZNF713	-0.16016	0.306047
NA	0.206479	0.305994
AL512625.2	0.256636	0.305992
PTGES3	-0.06553	0.305958
CDS1	1.554635	0.305888
HIST1H2BK	-0.09177	0.305879
CDKN3	-0.13987	0.30582
NDUFB8	-0.18229	0.305742
ZNF18	0.20574	0.305712
COA3	0.120172	0.305688
AL158166.1	0.546423	0.305669
AP001021.1	1.197281	0.305631
SYPL1P2	0.452712	0.305597
NA	0.128423	0.305556
PRR5L	-0.53062	0.305541
ZNF286B	-0.20019	0.305454
STN1	-0.13108	0.305371
SMYD1	2.716903	0.305365
TGM3	2.716903	0.305365
MYOD1	2.716903	0.305365
SPINK5	2.716903	0.305365
STX11	2.716903	0.305365
MMD2	2.716903	0.305365
FBN3	2.716903	0.305365
KCNE4	2.716903	0.305365
HPGDS	2.716903	0.305365
RSPO1	2.716903	0.305365
TSGA10IP	2.716903	0.305365
OR5AK3P	2.716903	0.305365
RNF135	2.716903	0.305365
CAV3	2.716903	0.305365
ELOA3	2.716903	0.305365
NA	2.716903	0.305365
RF00019	2.716903	0.305365
PKHD1L1	2.716903	0.305365
AL390955.1	2.716903	0.305365

CBX1P2	2.716903	0.305365
RPL23AP17	2.716903	0.305365
RPSAP43	2.716903	0.305365
AL583835.2	2.716903	0.305365
RNU6ATAC8P	2.716903	0.305365
PSMD4P1	2.716903	0.305365
AL450226.1	2.716903	0.305365
HSPE1P13	2.716903	0.305365
CROCC2	2.716903	0.305365
CENPCP1	2.716903	0.305365
NA	2.716903	0.305365
BTBD6P1	2.716903	0.305365
HMGB1P45	2.716903	0.305365
HMGB1P16	2.716903	0.305365
BCAS2P2	2.716903	0.305365
NDUFB1P2	2.716903	0.305365
AC013429.2	2.716903	0.305365
AL021154.1	2.716903	0.305365
AC093422.3	2.716903	0.305365
YWHABP1	2.716903	0.305365
CASC4P1	2.716903	0.305365
NA	2.716903	0.305365
C1QTNF9	2.716903	0.305365
RN7SL76P	2.716903	0.305365
RN7SL293P	2.716903	0.305365
RN7SL395P	2.716903	0.305365
LINC02428	2.716903	0.305365
AC034245.1	2.716903	0.305365
AC026725.1	2.716903	0.305365
AL445187.1	2.716903	0.305365
AC004066.2	2.716903	0.305365
LINC01843	2.716903	0.305365
LINC01094	2.716903	0.305365
LINC02143	2.716903	0.305365
AC107909.2	2.716903	0.305365
NA	2.716903	0.305365
MAFA-AS1	2.716903	0.305365
AP001775.1	2.716903	0.305365
AP001453.3	2.716903	0.305365
AC126177.3	2.716903	0.305365
AC124947.2	2.716903	0.305365
C20orf141	2.716903	0.305365
AC027237.3	2.716903	0.305365
AC091304.2	2.716903	0.305365
MIR4322	2.716903	0.305365
NA	2.716903	0.305365
AP001094.3	2.716903	0.305365
ARHGAP27P2	2.716903	0.305365
AC021683.2	2.716903	0.305365
AC007998.3	2.716903	0.305365

AC092068.3	2.716903	0.305365
FKBP1AP1	2.716903	0.305365
AC245884.10	2.716903	0.305365
AC005479.2	2.716903	0.305365
AL590235.1	2.716903	0.305365
MARK2P16	2.716903	0.305365
AL109936.2	2.716903	0.305365
TPTE2P2	2.716903	0.305365
Z73429.1	2.716903	0.305365
LINC01990	2.716903	0.305365
FO393411.1	-0.64625	0.30536
AL352979.3	-1.53853	0.305154
MPHOSPH10	0.090883	0.305152
SGPP2	0.167375	0.305145
C7	-0.1003	0.305086
GAS6	-0.10745	0.305084
AIF1L	0.141846	0.305053
HSPBAP1	-0.12636	0.305043
GINS4	-0.14792	0.305024
DEPDC1	-0.12745	0.305015
CARMN	-0.59507	0.305001
NA	-0.4764	0.304997
C2orf72	0.210723	0.304974
MAGI1	0.093019	0.304951
PCID2	0.082419	0.304845
ZNF571-AS1	0.230568	0.304803
SERF2	0.065515	0.304791
HIST1H4J	1.734343	0.30479
OAS2	1.253142	0.304779
ASB16-AS1	-0.17355	0.304703
TMBIM4	0.156794	0.304694
NA	-0.38651	0.304688
EFCAB14-AS1	-0.36217	0.304687
AC006435.1	0.184117	0.304671
PRKD2	-0.10717	0.304661
MAN1B1	-0.09859	0.304611
UXT	0.109986	0.304601
LINC02454	-0.70091	0.30423
NOSIP	0.09148	0.304151
ZNF629	-0.08399	0.304079
WDR76	-0.14394	0.304065
ZNF763	0.6931	0.304033
ZNF789	0.102875	0.304023
AC104365.1	1.134527	0.303981
SMIM19	0.124755	0.303938
NPIPA1	-0.1545	0.303915
AC091153.4	-0.20283	0.303863
STAG1	0.088132	0.303808
ZNF32-AS2	-0.17292	0.303765
BHLHE41	-0.42098	0.303754

AL050343.1	0.134618	0.303693
RPL4P4	0.249791	0.303638
IQSEC3	0.20254	0.303616
Z99716.1	0.470228	0.303569
ZNF280C	-0.11131	0.303459
SESN1	0.118359	0.3034
TMEM91	0.245675	0.303389
NA	-0.48716	0.303365
IL18BP	-0.08698	0.303347
ZBTB12	0.111515	0.303329
AC243919.1	0.30083	0.303306
GEMIN7-AS1	-0.18269	0.303305
ODF2L	0.112963	0.303286
NIPAL4	0.444186	0.303277
ISLR2	-0.12346	0.303186
PROC	0.509015	0.303127
LCMT1	-0.10376	0.303104
AC011498.3	-0.20478	0.303062
NF1	-0.07279	0.303052
OTUD1	0.306979	0.30303
KLRG1	0.221774	0.303021
NA	0.30661	0.302977
CCDC86	-0.08958	0.302966
ZDHHC8P1	0.20687	0.302961
ZFYVE21	0.118526	0.30287
GGACT	-0.31434	0.302859
INPP5J	0.195369	0.302824
PRIM1	0.102189	0.302809
ANG	1.858869	0.302802
MFSD14B	0.080518	0.302746
CXCR4	0.2894	0.302683
RDM1P5	0.709086	0.302677
KRT8P39	-0.54917	0.302655
AC008969.1	-0.17741	0.302654
NA	-0.3859	0.302605
MTIF3	-0.11088	0.302571
NCOA2	0.103567	0.302517
PTPRE	-0.11863	0.302462
HELZ2	-0.20067	0.302445
AC024267.3	-0.67756	0.302425
SPPL2A	-0.10743	0.302376
TSPAN9	0.119969	0.302331
AC093591.1	0.677889	0.302314
CEBPZOS	0.102293	0.30231
MAP6	0.149281	0.302306
PLPPR5	-0.10577	0.30227
ZER1	0.120324	0.302192
ZNF16	0.167532	0.302126
NA	-0.07776	0.30212
ANKRD34C-AS1	1.936049	0.302118

SCG5	-0.09496	0.30211
ITPA	0.078306	0.302067
VPS29	0.09487	0.302024
BMERB1	0.100989	0.301985
NA	1.329779	0.301963
STRN3	-0.10414	0.301933
RIMS1	-0.27765	0.30191
TPPP	-0.27181	0.301813
ULBP3	2.488152	0.301808
RF00019	2.488152	0.301808
AC016716.1	2.488152	0.301808
AC239800.1	2.488152	0.301808
AL360093.1	2.488152	0.301808
OFD1P17	2.488152	0.301808
AL157392.2	2.488152	0.301808
NA	2.488152	0.301808
AP003717.2	2.488152	0.301808
AC092718.6	2.488152	0.301808
RN7SL246P	2.488152	0.301808
FZD7	0.259165	0.301785
AC027601.2	-0.11708	0.301751
AC026202.3	0.258903	0.301749
AC055822.1	-0.75232	0.301732
AC063976.2	-1.31292	0.301722
PCDHB4	-0.14173	0.301709
POMT2	-0.08813	0.301664
ASIP	-1.11412	0.301662
CCT2	0.066971	0.301643
AC025198.1	2.022482	0.301567
KAZN	0.129691	0.30156
LINC02449	0.734824	0.301546
IRF1	0.14446	0.301476
AC006058.2	1.85084	0.301424
AL132639.3	0.233921	0.301388
IFITM3	0.1992	0.30132
SNRPGP14	0.724078	0.301312
ZNF446	-0.11647	0.301253
MIR7-3HG	0.393795	0.301227
DMRTA2	-0.35906	0.301144
AL354740.1	-0.07316	0.301133
FLACC1	-0.72349	0.301131
THUMPD2	0.126889	0.301081
KLHL10	0.490041	0.301032
VIM	-0.09329	0.300932
TNFRSF19	-0.15299	0.300928
GSK3B	-0.07808	0.300904
E2F8	0.16197	0.300812
NA	-0.44676	0.30075
RRS1-AS1	0.746916	0.300745
PTCH1	-0.1068	0.300741

RPL24P8	0.69095	0.300731
MTF1	0.096313	0.300712
AL162231.1	-0.47933	0.300601
FXYD5	-0.76528	0.300563
JADE1	-0.0867	0.300403
CHRM4	1.085777	0.300389
MTMR7	-0.16122	0.30038
MIR320E	-1.76037	0.300353
MIR941-2	-2.13316	0.300284
PRELID1P1	-1.41218	0.300229
ADORA2BP1	-1.00116	0.300203
AL359962.1	2.036841	0.300182
SLC25A45	0.193265	0.300143
EIF4E	-0.08446	0.300099
DUSP11	0.100699	0.300066
AC099522.2	-0.59126	0.300053
LY96	2.036857	0.300014
MANSC4	2.036857	0.300014
RN7SL330P	2.036857	0.300014
AC073349.1	-1.65667	0.300012
AC091563.1	-1.65667	0.300012
CLIP2	0.100824	0.299964
PRKCH	0.284243	0.299961
NUF2	-0.11021	0.299945
HSD17B6	0.153271	0.299928
CCDC85C	-0.12726	0.299861
AC108010.1	-0.16125	0.299842
DLG1	-0.09217	0.299833
AGTRAP	-0.15147	0.299769
TMCC2	-0.15419	0.299714
RNF227	0.21261	0.29969
AC002128.1	-0.93148	0.299671
AC025048.3	-1.6357	0.299628
SH2B1	-0.07927	0.299627
ACLY	-0.06646	0.299538
MIR4477B	-0.20816	0.299521
WASH6P	-0.28741	0.299462
CSNK1G2-AS1	0.6964	0.299442
NA	0.925417	0.299418
AC007570.1	1.191109	0.299366
AC092168.2	1.490519	0.299342
SRSF11	-0.07792	0.299323
SLC2A6	0.12151	0.299319
MNAT1	0.15394	0.299295
ABRAXAS1	0.111509	0.299258
CCZ1B	0.084295	0.299129
ANP32B	-0.07291	0.299098
AP005329.2	-0.43697	0.299082
NTAN1	-0.08723	0.299067
GATAD2A	-0.08127	0.299063

AC013356.2	-0.18373	0.299058
GTF2IRD2P1	1.649815	0.299054
RF00019	2.606087	0.299019
AL512504.1	2.606087	0.299019
AC018511.1	2.606087	0.299019
AC008080.2	2.606087	0.299019
SLC4A1APP1	2.606087	0.299019
AC008750.1	2.606087	0.299019
AC090458.1	2.606087	0.299019
WDFY4	2.67248	0.298982
SPACA3	2.67248	0.298982
IL23R	2.67248	0.298982
RASSF9	2.67248	0.298982
RNU6-501P	2.67248	0.298982
NA	2.67248	0.298982
MIR550A2	2.67248	0.298982
AL590639.1	2.67248	0.298982
AL512593.1	2.67248	0.298982
NDUFB1P1	2.67248	0.298982
AL583824.1	2.67248	0.298982
PTP4A1P7	2.67248	0.298982
NA	2.67248	0.298982
AC078785.2	2.67248	0.298982
EGFLAM-AS1	2.67248	0.298982
AC106772.1	2.67248	0.298982
AC008957.1	2.67248	0.298982
MIR583HG	2.67248	0.298982
RN7SKP5	2.67248	0.298982
RN7SKP98	2.67248	0.298982
TAS2R43	2.67248	0.298982
AC007595.1	2.67248	0.298982
RN7SL230P	2.67248	0.298982
NA	2.67248	0.298982
AC105094.2	2.67248	0.298982
AC011509.2	2.67248	0.298982
AC006262.2	2.67248	0.298982
AC090559.2	2.67248	0.298982
AC092681.3	2.67248	0.298982
TP53I11	-0.19424	0.298959
ASPN	1.161157	0.29894
MRPL38	0.23551	0.298934
AC092198.1	-0.26183	0.298779
AC124016.1	-0.3221	0.29869
SLC25A27	0.284338	0.298649
AC069282.1	-0.35176	0.298638
PLGLA	0.518603	0.298604
AL592148.3	-0.11264	0.298586
LINC02097	1.297527	0.298528
MRPL58	0.094965	0.298459
CSPG5	-0.31277	0.298458

MAGEH1	0.119211	0.298429
TP53INP2	-0.14407	0.298422
ATP2B3	0.208611	0.298396
SRA1	0.091284	0.298364
RPSAP52	-0.80113	0.298362
SENP2	-0.08519	0.298319
LINC01535	-0.51041	0.298308
MAGOHB	-0.12111	0.298297
LINC01952	-1.0462	0.298257
HAAO	-1.18583	0.298147
AC067930.1	0.076001	0.298105
NINJ1	-0.15191	0.29809
C10orf90	1.459118	0.298034
AL008638.1	1.459118	0.298034
RNF150	0.071541	0.298033
CR936218.1	-0.68705	0.298002
C16orf74	-0.37537	0.298
AC004895.1	0.975441	0.297999
AC138393.2	-1.51473	0.297963
NA	-0.10643	0.297926
KLHL28	0.110292	0.29789
FAM86EP	-0.31757	0.297861
AC009107.2	-0.95499	0.297859
AL162724.2	-2.12338	0.297847
NA	1.196583	0.297843
CASKIN2	-0.11983	0.297838
AP000845.1	-0.90409	0.297827
AL645608.3	1.156336	0.297815
SLC2A12	0.233901	0.297806
NA	0.346903	0.297803
NHEJ1	0.607066	0.297793
NA	0.374432	0.297705
ADAR	-0.06829	0.297685
RBM43	0.218381	0.297663
ALOX5	-1.60707	0.297651
DEDD	0.082163	0.297636
PDGFC	-0.26248	0.297618
ZSWIM1	-0.14964	0.297603
ARV1	0.115972	0.297589
AC026367.1	0.809737	0.297579
WDFY1	0.089625	0.297574
OXLD1	0.09244	0.29752
NMT1	-0.0735	0.297493
NA	0.139262	0.297475
MSL1	-0.07777	0.297375
ZKSCAN4	-0.14554	0.297307
LIMD2	-0.07123	0.2973
TSPYL4	0.075765	0.29724
AC012588.1	1.282379	0.297228
ANKRD63	-1.11888	0.297119

RAD51AP1	0.123857	0.297065
RNGTT	-0.08522	0.297049
AC013460.1	-0.83233	0.29702
PLXNB2	-0.09396	0.297018
PACSIN1	1.075458	0.297
AC105020.3	-0.37759	0.296973
LINC01465	0.588716	0.296964
SH3TC1	0.136918	0.296963
THRSP	1.247026	0.296945
SGSH	0.104385	0.296893
AC011676.2	-0.90333	0.296814
SORCS1	-0.08527	0.296755
HNRNPA1P59	-0.5884	0.296754
NA	1.266742	0.296679
BCRP8	-1.54424	0.296661
RAPSN	-1.88391	0.296639
NA	-1.88391	0.296639
RGCC	-1.24036	0.296623
SYNCRIP	-0.07027	0.296572
HCFC1R1	-0.11555	0.296556
EGLN2	0.169634	0.296547
SEC16A	-0.08794	0.296509
ADSSL1	-0.21217	0.296492
PACRG	-0.3716	0.29648
AL356133.1	-2.13305	0.296422
AC007000.1	-0.43843	0.2964
RHOB	0.097632	0.296371
OAZ1	0.061462	0.296328
SLC10A7	-0.19028	0.296308
NA	1.292869	0.296307
NPHP3	-0.09028	0.296305
RTN4	-0.06919	0.296249
ZNF135	-0.21478	0.296246
RNU7-57P	-0.88305	0.296175
AC022098.3	-1.1831	0.296129
KATNA1	0.107602	0.296105
NA	-0.20988	0.295992
MEIOC	-0.12888	0.295958
GJB7	0.52873	0.295911
AL161452.1	-0.11292	0.295895
RNU6-1178P	1.529333	0.295878
AL008729.1	-0.30078	0.295857
DBN1	0.07552	0.295837
AC022400.5	0.504915	0.295818
MRPL53	0.277982	0.295771
ANXA6	0.094936	0.295766
NA	-0.3063	0.295753
ZNF233	0.179788	0.29567
AL133260.1	-1.31303	0.295632
SCAF8	-0.08996	0.29561

ADAMTS14	-0.18207	0.295555
LINC00677	1.899394	0.295405
PTPN1	-0.08697	0.295372
MTR	-0.06882	0.295346
CUL5	-0.10818	0.295317
AC093249.6	0.226981	0.295315
UXT-AS1	0.136934	0.295235
HLX-AS1	0.290789	0.295218
MRPS31	-0.11637	0.295171
PILRB	0.19314	0.295143
AC025588.1	0.716925	0.295125
LINC00327	-0.578	0.295114
AC018752.1	-1.13218	0.295099
FRA10AC1	0.140589	0.295059
PCDHA3	-0.3967	0.295043
LINC01494	-1.5832	0.29503
NA	-1.32771	0.294938
CABLES2	-0.16336	0.2949
AC073863.1	-0.27552	0.294891
NSMCE1	0.122816	0.294891
IGHVII-33-1	1.351288	0.294854
APOL6	0.452633	0.29485
AL929472.2	-0.25166	0.294845
ZNF844	0.229099	0.294816
ZNF849P	-1.01875	0.294813
DCAF16	0.084905	0.294809
TMEM106A	-1.74886	0.294769
AC006970.2	-1.74886	0.294769
IFT74-AS1	-1.74886	0.294769
AL358216.1	-0.87701	0.294765
IQGAP3	-0.08075	0.29471
HIF1A-AS1	0.118306	0.294694
TUBBP1	-0.39728	0.294671
NA	-1.58951	0.294558
STK35	-0.11567	0.294525
CBX1	-0.06467	0.294516
AC005740.1	-0.96103	0.29447
MCF2L	0.142418	0.29447
JUNB	0.097237	0.294459
AL035398.1	-1.21024	0.294416
ZNF684	-0.20098	0.294409
SYNGR1	-0.16721	0.294377
ABHD11	-0.15461	0.294235
NA	0.378985	0.294231
SPDYE5	0.278752	0.294216
RNF6	0.10366	0.294177
AL137847.1	-1.39296	0.294164
WIZ	-0.10214	0.294162
ALG2	0.098439	0.294155
RNF8	-0.11275	0.294138

RBM39	0.069592	0.29412
NA	-1.09355	0.294103
CRACR2B	-0.27456	0.294102
AL158211.2	1.672982	0.294092
CDH23	-0.54486	0.294087
NA	0.132191	0.294059
AC126389.1	1.422689	0.294007
AP001885.1	-2.12325	0.293989
AL034417.2	-2.12325	0.293989
NA	0.574479	0.29392
ALMS1-IT1	-0.71152	0.29391
MTERF4	-0.08138	0.293903
ARHGAP36	0.242488	0.293875
DHRS12	-0.18278	0.293821
PEA15	-0.07984	0.293791
AC005865.1	0.893743	0.293774
DNMT3A	0.07593	0.293753
TES	0.126328	0.293733
AC004988.1	-1.58657	0.293719
XPOTP1	-1.58657	0.293719
TEX38	-2.13297	0.293711
RGS17P1	-2.13297	0.293711
AL354872.2	-2.13297	0.293711
DLX6	0.144201	0.293703
AL691432.2	-0.18756	0.293687
AC093866.1	0.293843	0.293682
AGAP5	-0.91132	0.293667
ODF2-AS1	0.181673	0.293659
ERBIN	0.075048	0.29365
AC124798.1	-0.23796	0.293614
PLEKHB1	-0.20738	0.293597
PPIAP31	0.452789	0.293586
ZNF704	0.081785	0.293577
DCAF6	0.08257	0.293576
SUPT7L	-0.07273	0.293546
SYCE3	-1.5099	0.293533
AC023906.5	-1.5099	0.293533
EIF4A1P5	2.122117	0.293448
TMEM97P1	2.122117	0.293448
C10orf71-AS1	2.122117	0.293448
AC020549.1	2.122117	0.293448
AC005840.4	2.122117	0.293448
AC087241.2	2.122117	0.293448
AL008727.1	2.122117	0.293448
MIR378D2HG	2.122117	0.293448
AC018761.1	2.122117	0.293448
NA	2.122117	0.293448
AC063962.1	2.122117	0.293448
AL445423.1	2.122117	0.293448
POLG2	-0.08203	0.293439

EEF2	0.078167	0.293409
NOP10	0.075471	0.293387
LINC00311	-0.93358	0.29338
ZMYM3	-0.08889	0.293327
AC114947.2	0.148626	0.293239
SLC15A4	0.116739	0.2932
MINK1	-0.09653	0.293155
GVQW3	0.169941	0.293087
AL450263.1	-0.55981	0.293043
CLIP1-AS1	-1.71273	0.292971
AC012442.1	-1.05309	0.292955
SPACA6	-0.25608	0.292877
ZSWIM8-AS1	1.275242	0.292839
HMGN2P15	0.39008	0.292813
CCNT2	0.076553	0.292784
ORMDL2	-0.08722	0.29275
NA	-1.12583	0.292736
ITPKB	0.159792	0.292723
GPRC5B	-0.2222	0.29271
ZDHHC17	0.083057	0.292688
TDRP	-0.12717	0.292682
AC002467.1	-0.24882	0.292638
GRID1	-0.16941	0.29263
MAPK11	-0.09693	0.292613
TRPA1	-0.32089	0.292585
RAP1AP	-1.6506	0.292584
LINC01277	1.899323	0.292584
AL359853.3	1.899323	0.292584
DDHD1	-0.11148	0.292584
USH1G	1.776164	0.29256
CES5AP1	1.776164	0.29256
RPL26L1	-0.09941	0.292547
NID1	-0.11042	0.292523
FAM243A	0.662647	0.292512
DHRS4L1	-0.49867	0.292492
ZNF335	0.125941	0.292476
THRB-AS1	1.899319	0.29241
PRR29-AS1	1.899319	0.29241
AC092755.2	0.198519	0.292335
DBX2	1.125909	0.292322
SFXN5	-0.13133	0.292309
MT-CYB	0.063193	0.292238
HMGB1P37	1.624781	0.292194
EPS15L1	-0.10704	0.292139
IGFBP7	0.124942	0.292107
DUSP26	0.158194	0.292092
PINK1-AS	-0.10911	0.292092
HSPH1	-0.0681	0.292052
COPS6	-0.06641	0.292015
MIR324	0.429461	0.291971

HSD11B1	0.372202	0.291935
CNOT9	0.081472	0.291934
NA	-1.23086	0.291902
AL359397.1	1.058783	0.291884
CCNG1	0.085082	0.291872
KCNK12	-1.27171	0.291862
AC025682.1	-0.11104	0.291802
AC132219.1	-0.47057	0.291749
ZNF879	-0.2341	0.291721
SLC25A2	1.406825	0.291664
HMGN1P36	0.763461	0.291599
NA	-0.14662	0.291526
PPIL6	0.223986	0.291498
PTBP1	-0.06373	0.291486
TNS1	-0.11234	0.291448
AC009120.1	0.370309	0.29144
AC009803.2	-1.00883	0.291414
SLC37A1	-0.20196	0.291364
MIR503HG	-0.32653	0.291344
RF00019	1.038055	0.291303
NA	0.213481	0.29128
MYOM3	-2.12315	0.29128
POM121L6P	-2.12315	0.29128
AC008972.1	-2.12315	0.29128
AL645940.1	-1.06951	0.291261
PCCB	0.078298	0.291229
DOP1A	0.082067	0.291173
AC139887.4	0.693476	0.291116
AC107464.1	0.343114	0.291081
NA	-0.18741	0.291045
IL16	-0.59304	0.291024
CRAT	-0.14238	0.291016
HACD2	-0.10657	0.291016
AC111186.1	-1.21025	0.291016
CAPZB	-0.07736	0.290989
PROB1	-0.32526	0.29098
HERC2P9	0.111357	0.290949
P2RX6	-0.19421	0.290909
AC073072.1	2.122457	0.290865
IGHV4-31	2.122457	0.290865
RPS29P16	2.122457	0.290865
AC087501.2	2.122457	0.290865
RIC3-DT	-1.15061	0.290835
AC007216.2	1.165903	0.290832
SH3BP5L	0.082718	0.290807
AP003721.1	0.367414	0.290735
LINC02440	0.926324	0.290729
TM4SF18	2.122478	0.290706
BEND3P2	2.122478	0.290706
AC091729.2	2.122478	0.290706

AL360091.2	2.122478	0.290706
AC092651.1	2.122478	0.290706
AC092809.2	2.122478	0.290706
AL356273.1	2.122478	0.290706
NSRP1P1	2.122478	0.290706
RNU5F-4P	2.122478	0.290706
SEPT10P1	2.122478	0.290706
SINHCAFP1	2.122478	0.290706
AP005403.1	2.122478	0.290706
NUCB1-AS1	0.09348	0.290671
NOTCH4	0.173568	0.290648
SYNE2	0.073985	0.290557
GTPBP2	0.073857	0.29055
AL391335.1	-0.27123	0.290501
MTG1	0.132633	0.290461
AC090912.1	-0.91412	0.290456
SIAH2-AS1	-0.31317	0.290425
YTHDF2	-0.06931	0.290418
APBB2	-0.11163	0.290404
ACOT11	0.14127	0.290393
ZNF184	0.124067	0.290328
TCAP	0.255643	0.290301
RPL13AP6	1.321421	0.290262
AL031284.1	1.321421	0.290262
FAM66D	-0.39499	0.290237
GLYATL1P2	-0.66238	0.290223
FTH1	0.087677	0.290214
AC005829.2	0.423361	0.290175
DCST1-AS1	0.351561	0.290146
AP002026.1	-0.21621	0.290144
RAB12	0.102396	0.290113
AC079949.1	-0.71552	0.290092
CR786580.1	-0.40316	0.290032
ANKRD20A11P	-0.4486	0.290022
SHC2	0.132367	0.290005
VN1R1	-0.89482	0.290004
AC127024.5	-0.21539	0.289995
RASSF1	0.106711	0.289978
IPO11	-0.10572	0.289965
TTC9C	-0.0998	0.289946
LINC01289	-1.88411	0.289944
TMEM128	-0.09021	0.289935
ACP6	-0.14338	0.289934
PVRIG	0.327942	0.289911
SNORD11	-1.64082	0.289864
AC010168.2	0.261246	0.289771
ZNF575	0.187334	0.289771
NAB2	0.199992	0.289758
ZNF606	0.126875	0.289726
PCDHB8	0.406924	0.289724

RPL7P16	1.127414	0.289677
SENP1	0.088354	0.289658
AL356488.2	0.754449	0.289652
LHFPL6	0.19732	0.289619
AC073869.1	-0.13034	0.289593
AC091564.6	-0.23416	0.289561
SNHG26	1.439407	0.289491
UBQLN4P1	-0.92612	0.289447
PIAS1	-0.09228	0.289228
AC019077.1	-0.28182	0.289198
DYNC2H1	-0.14856	0.289197
JAK3	-0.37667	0.289181
AC079907.1	0.288104	0.289174
RF00012	-1.16974	0.289122
IL3RA	0.285067	0.289042
ITGB4	-0.44951	0.28904
AC012360.1	1.260631	0.289027
JUN	0.116418	0.289025
MRPS17	0.1521	0.289025
NA	-0.26751	0.288899
TMEM87B	0.101998	0.288825
AC116903.1	-0.44132	0.288777
AC004540.1	0.455548	0.288763
DNAJC30	0.140087	0.288676
TRMT13	0.099541	0.288673
SLC25A3	0.075315	0.288631
AC083862.2	0.106311	0.288589
GEN1	-0.10133	0.288551
UBAC2	0.087559	0.288547
CLPSL2	1.193733	0.288525
GATM	0.264074	0.288517
DDX51	0.118605	0.288449
PIGC	-0.09295	0.288406
DFFBP1	-0.35781	0.288339
MBNL2	0.099128	0.288299
AC010998.2	-0.64941	0.288226
PYM1	0.093832	0.288199
GTPBP4	0.065006	0.288176
ACO2	-0.09187	0.288152
NA	0.409164	0.288146
NA	-0.75646	0.288065
PYY	1.30884	0.288036
ADAM22	-0.11123	0.288036
CNOT11	-0.08814	0.287993
NA	-0.18453	0.287959
PLCB2	-0.32602	0.287959
DPY19L3	0.072521	0.287827
AL031963.1	0.156707	0.287812
TMEM69	0.094528	0.287766
AC084024.3	0.122245	0.287751

GRM3	-0.42051	0.28768
SPATA33	0.151452	0.287656
DHFR	-0.11543	0.287639
RFX1	-0.15412	0.287615
NA	-1.17951	0.287585
DDX39B	-0.06928	0.287473
TTBK1	0.152362	0.287445
SSH1	0.110996	0.287414
CPZ	2.558453	0.287388
PXT1	2.558453	0.287388
RF00017	2.558453	0.287388
ST13P5	2.558453	0.287388
FP700111.1	2.558453	0.287388
AC009955.1	2.558453	0.287388
Z99127.1	2.558453	0.287388
NEK2P4	2.558453	0.287388
LINC01160	2.558453	0.287388
NDFIP2-AS1	2.558453	0.287388
AC007969.2	2.558453	0.287388
AC079594.1	2.558453	0.287388
AC139426.1	2.558453	0.287388
AC010325.1	2.558453	0.287388
AC005736.1	2.558453	0.287388
MIR3680-1	2.558453	0.287388
TMEM181	-0.06847	0.287297
ERAL1	-0.08554	0.287282
ANKRD34C	-1.84816	0.287282
AL162377.1	-0.67584	0.287274
PODN	0.5048	0.287185
NA	0.646999	0.287141
GOLT1B	0.081112	0.287139
SNORA3B	0.647365	0.287101
AC016831.1	0.365066	0.287091
TMEM63B	-0.10271	0.286988
ANKRD45	0.244223	0.286965
PDE7B	0.350211	0.28694
JMJD7	-1.27462	0.286935
POLR2K	-0.09126	0.286924
NA	0.198966	0.286867
NA	-0.13938	0.286865
HPGD	1.326871	0.286846
RN7SL49P	1.149609	0.286843
ZNF501	-0.19766	0.286833
RAB3IP	0.133139	0.286812
GTF2H4	-0.96351	0.286803
RPL23AP42	0.175473	0.286749
AC011365.1	-0.13025	0.286745
AL354710.2	-1.8459	0.2867
LINC01331	1.324717	0.28668
FAM78B	-0.20206	0.286665

TMEM132B	0.658207	0.28666
UTP6	-0.08073	0.286637
SHARPIN	-0.09459	0.286501
AC069224.1	0.383857	0.286408
AC009779.2	-0.16715	0.286405
NPM1P39	0.796581	0.286382
KIAA1522	0.142687	0.286322
NA	0.29137	0.286307
HMGN1P7	-1.71642	0.286297
PNMA3	1.486023	0.286272
SCRT2	-0.22717	0.286251
AL512770.1	-0.09674	0.286216
AFMID	-0.10013	0.286111
RNF13	0.084576	0.286109
NA	-0.07033	0.286034
TMEM205	0.109106	0.285956
ABCA12	0.126003	0.285917
SEPT3	0.298398	0.285847
USP42	0.088653	0.285821
MSI1	0.154485	0.285817
GZF1	0.10476	0.285802
AL121845.1	-0.48166	0.285775
MT-TC	1.270022	0.285696
MICOS10	0.175625	0.285693
KIAA0232	0.091829	0.285667
QPCTL	0.144997	0.285647
AC007318.1	0.41547	0.28563
SNN	-0.1118	0.285618
CCDC178	0.799255	0.285578
GTF2IP4	-0.12068	0.285562
PLEKHH3	-0.11231	0.28556
MIR593	1.681901	0.285535
ATP5F1CP1	1.681901	0.285535
RF00019	-0.48817	0.285524
RNF219-AS1	0.14335	0.285468
APC2	-0.11199	0.285463
CHRNA10	0.330261	0.285447
SRMS	-2.40634	0.285412
DDX53	-2.40634	0.285412
MARCKSL1P1	-2.40634	0.285412
AL365184.1	-2.40634	0.285412
PCDHGB8P	-2.40634	0.285412
AP000442.1	-2.40634	0.285412
AC106886.1	-2.40634	0.285412
AC020663.2	-2.40634	0.285412
AL157904.1	-2.40634	0.285412
BOLA3	0.152955	0.285399
PRPF38A	-0.0766	0.285383
C8G	0.685483	0.28537
NA	-1.68889	0.285331

RPL9P7	-1.68889	0.285331
SNORD3B-2	-1.68889	0.285331
C6orf62	0.069649	0.285221
CNTNAP5	0.265655	0.285215
ANKRD46	0.123322	0.285175
AL445228.2	-0.59756	0.285126
AP002981.1	-1.023	0.285121
LINC01119	0.588205	0.28509
KLHL25	0.111323	0.285059
PBX2P1	-1.37935	0.285012
LRRCC1	-0.1545	0.284999
DNAJC19P9	1.746783	0.284982
DPP7	0.082386	0.284971
CEP44	0.066166	0.28494
LDB2	0.197424	0.284915
LINC02236	-0.95991	0.284903
DPAGT1	-0.11218	0.284886
SDHAP3	0.129662	0.284863
DELE1	-0.0785	0.284842
AC022149.1	0.396699	0.284829
SPIB	-0.55159	0.284812
TLK1	0.095317	0.284805
NFATC2	0.581238	0.284801
LINC01537	-1.55278	0.284735
AC124067.2	-1.55278	0.284735
NA	1.562049	0.284695
ADGRA3	-0.07281	0.284552
NA	-0.53471	0.284539
ZNF582	0.20217	0.284455
LMF1-AS1	-0.69678	0.284442
MIGA2	-0.10799	0.284424
AC104564.3	-0.68988	0.284387
POLR3E	0.078186	0.284378
NA	-0.38261	0.284378
KCTD11	0.131358	0.284359
AL592293.1	-0.84704	0.284293
SBDSP1	0.098946	0.284291
MPDU1	0.070378	0.284276
RASGRF2-AS1	1.198263	0.284241
KBTBD3	0.227492	0.284218
C7orf61	-1.3472	0.284214
DBF4P1	-0.84095	0.284206
PAQR9	0.58151	0.284177
POM121	-0.08922	0.284154
AL355312.2	0.818913	0.284141
BAIAP2-DT	0.134269	0.284076
CAMK2G	-0.09124	0.284049
HTR2C	1.558443	0.28401
C2orf27A	0.269686	0.283998
AC007216.4	-0.70028	0.283955

SEC62	0.080278	0.283898
C3orf33	-0.29852	0.283866
AL591866.1	0.967615	0.283865
AL109766.1	1.972947	0.283786
SUB1P3	1.972947	0.283786
AC243562.3	-1.19224	0.283768
SQLE	0.113211	0.283718
AC068700.1	0.535094	0.283677
TTC4	0.539167	0.283671
ZNF91	0.098041	0.283554
SNF8	0.077712	0.283403
RNF103	-0.11389	0.283374
PAK6	-1.84813	0.283362
C12orf74	-1.84813	0.283362
CNN3	0.075198	0.283358
DNAJA3	-0.08062	0.283263
MIR4497	1.463673	0.283185
AC073517.1	-0.80958	0.283119
RBMX2	0.092342	0.283062
COL9A3	-0.28621	0.283059
EIF4A3	0.069563	0.283017
TUSC3	0.083267	0.282997
ABLIM1	-0.06928	0.282983
TYK2	-0.09009	0.282964
POLR1A	-0.086	0.282944
AC008543.5	1.333662	0.282939
OIP5-AS1	0.06997	0.282848
AC073476.3	0.84105	0.282834
RNF212B	-0.53838	0.282749
AC138932.1	-0.16408	0.282736
AL391058.1	0.797754	0.282719
ABCD2	0.104397	0.282717
RAB8B	-0.10581	0.282671
STRIP1	0.085317	0.28262
LINC01278	0.12314	0.282614
LYSMD1	0.101446	0.282602
SERPINH1	0.076574	0.282579
PAGE2	1.024184	0.282489
PARP11	0.174577	0.282439
PPM1G	-0.05985	0.282427
A4GNT	2.538418	0.282419
DDI1	2.538418	0.282419
RF00418	2.538418	0.282419
RNF152P1	2.538418	0.282419
RF00019	2.538418	0.282419
LINC00571	2.538418	0.282419
AC007879.1	2.538418	0.282419
RPS4XP2	2.538418	0.282419
LINC01760	2.538418	0.282419
AC096576.3	2.538418	0.282419

AC004585.1	2.538418	0.282419
AL356423.1	2.538418	0.282419
AC087257.1	1.276053	0.28241
TEN1	-1.31198	0.282353
CDT1	0.119448	0.2823
PDGFB	-0.52062	0.282202
FAM86C1	0.169568	0.282169
TRIM29	2.080695	0.282142
SKAP1	2.080695	0.282142
ZNF177	2.080695	0.282142
SMIM10L2B	2.080695	0.282142
FAM83C-AS1	2.080695	0.282142
AC020910.1	2.080695	0.282142
LINC02266	2.080695	0.282142
CUTC	-0.11869	0.282052
RNF39	-0.1706	0.28195
ONECUT2	-0.10348	0.281913
CXCL3	-0.65203	0.281899
SGCD	0.404217	0.281845
FLT1	-0.39511	0.281836
ACTR3	0.07025	0.281799
LAP3	0.079567	0.281768
AL031722.1	0.610752	0.281717
AL590560.2	1.123602	0.281688
FNDC11	0.471866	0.281659
ZBTB47	-0.11913	0.28155
AC100830.1	-0.67416	0.281505
ANKRD9	0.146091	0.281451
AC092447.5	1.019909	0.281431
ZSWIM8	-0.08921	0.281378
PBX2	-0.0771	0.281358
RHOT1P2	1.195323	0.281357
ROR1-AS1	-0.21377	0.281295
MAST4	-0.15762	0.281258
TMEM245	-0.07872	0.281254
AC246680.1	-0.36353	0.281235
ECHS1	-0.07407	0.281235
CPEB1	0.339972	0.281222
PPARA	-0.09457	0.281198
AC079313.1	0.461246	0.281184
PSPHP1	-0.25368	0.281149
ZNF192P1	0.202557	0.2811
AC068057.1	-0.6939	0.281078
IL11	-0.15896	0.281076
PFDN1	0.09036	0.281074
TNFSF13B	0.990298	0.281045
RCN1	0.069039	0.281029
POU5F1P3	1.380407	0.281012
RALGAPA1	-0.09495	0.280953
RPUSD2	-0.13194	0.280953

MED23	-0.08196	0.280943
RNF214	-0.09729	0.280929
LONP2	-0.07724	0.280923
EMC1-AS1	-0.22567	0.2809
SYP	0.081993	0.280898
ANKRA2	0.107226	0.280882
TSPAN31	0.066065	0.280872
Z94057.1	-1.03442	0.280833
ATF2	0.086248	0.280821
AC100835.1	-1.11936	0.280781
INTS9-AS1	1.053748	0.280765
BCORL1	-0.11413	0.28075
HDHD5-AS1	0.681348	0.280714
IRF3	0.10065	0.280707
ANKRD24	-0.83131	0.280694
AC026412.3	0.360214	0.280683
AC079336.5	1.95094	0.28066
RPS27L	-0.08935	0.280654
DDHD2	0.080091	0.280622
SNRPB2	0.095911	0.280617
GOLGA6L7	-0.48585	0.280616
SMG1P2	0.252086	0.280609
THOC7	0.120608	0.280608
SULT1A2	-1.8481	0.280594
NA	-1.8481	0.280594
MIR378B	-1.8481	0.280594
MGAT5B	-0.13689	0.280589
IP6K2	0.074573	0.280538
AC093752.1	0.124244	0.280434
DTX3	0.096175	0.280416
GMFB	0.078659	0.280405
FHL1	0.093238	0.280376
ADORA2A-AS1	0.139986	0.280373
TLR2	-0.30372	0.28036
TOR1AIP1	-0.08035	0.280351
GNAL	0.1927	0.280283
NRARP	0.189285	0.280263
SUPT16HP1	0.954834	0.280228
BAZ1A	0.07973	0.280166
CDK10	-0.07765	0.28015
KCP	0.112354	0.280136
AC096586.1	0.244631	0.280067
DIRC3	0.656862	0.280055
AC092301.1	0.178516	0.280014
C17orf58	-0.1298	0.280007
BMS1P8	0.152214	0.279918
AC011365.2	-0.12501	0.279898
STPG1	0.182564	0.279888
FOXR2	-0.12142	0.279812
GSTP1	0.067698	0.279797

APOL4	-0.20115	0.279777
AL844908.2	-0.79075	0.27973
RUNX2	-1.79459	0.279723
NA	-0.78598	0.279688
POLR2J	0.078963	0.279577
SPCS2	0.070532	0.279558
MARK1	0.126488	0.279545
DPF2	-0.09037	0.279512
AL451074.1	1.077049	0.279511
LRRC41	0.078303	0.279461
NSUN6	-0.13813	0.279446
AL390728.4	0.102137	0.279424
AC091185.1	0.666336	0.279371
NA	0.730348	0.279341
GPAM	0.09016	0.27925
SLC15A3	0.237507	0.279196
LIPE-AS1	0.265538	0.279175
AC006042.4	-0.89392	0.279138
LRRC55	0.390291	0.279091
NA	-0.1529	0.279087
AC099508.1	-0.13658	0.279076
TUBB6	-0.09059	0.279057
GRPR	-1.00149	0.279045
AC027601.1	1.542239	0.278997
AC009630.3	0.098913	0.278944
ITPRIPL2	-0.09464	0.278923
KANSL1	-0.07438	0.27891
PRRC2C	-0.06603	0.278819
AC138409.1	0.458024	0.278703
AC002347.2	0.160171	0.278683
BCKDHA	-0.99115	0.278676
TFE3	0.099751	0.278642
AP003550.1	-0.22607	0.278607
PTCD1	-0.08374	0.278596
NA	1.180017	0.278563
BAIAP2	0.175996	0.278533
CBX4	0.116625	0.278511
G2E3	0.097392	0.278507
BANF1P3	0.929681	0.278497
LINC02623	-0.44405	0.278479
RECK	-0.11599	0.278444
C19orf53	0.086868	0.278428
SNORD9	-1.16724	0.278343
TFR2	-0.13121	0.278327
EPB41L5	-0.09616	0.278275
IMMP1L	0.14577	0.278245
SPACA9	-0.25354	0.278192
PER2	-0.22178	0.278164
TMEM18	0.079179	0.278126
TRBV26OR9-2	-0.57787	0.27811

SRSF7	0.070902	0.278062
C15orf65	0.358448	0.27801
PDCD4-AS1	-0.19866	0.277944
NUDT21	0.07521	0.277921
UPF3AP2	0.483817	0.277855
LMO1	0.112655	0.277832
RAPGEF6	0.127656	0.277794
ZNF574	-0.11415	0.277757
TMEM92-AS1	-1.6328	0.277742
CNNM3-DT	0.70492	0.277727
NMNAT1	-0.1795	0.277725
FRS3	0.13832	0.277696
NA	-1.07957	0.277662
EXOG	-0.15538	0.277618
ZNF346	-0.11208	0.277588
RN7SL81P	0.522126	0.277577
IPO8P1	0.894212	0.277509
PHAX	-0.0877	0.277459
MTMR2	-0.07223	0.277433
SLC22A14	1.6488	0.277315
RPL7P24	1.6488	0.277315
AC092647.5	1.112307	0.277299
NUP210L	0.76233	0.27721
GSDMD	-0.85738	0.277194
MTCO2P2	-0.8308	0.277127
TRMT2A	0.079861	0.27711
NA	-0.11441	0.277079
SLC35F6	0.098405	0.276973
NA	-1.21015	0.276926
CBFA2T2	-0.08364	0.276892
MGST1	0.229112	0.276842
RTL8B	-0.11227	0.276785
TRPC1	-0.10954	0.27676
RPS7P3	-1.17123	0.276744
B4GALT4	0.102624	0.276727
AC013451.2	1.253396	0.276686
SLC30A4	-0.15388	0.276686
ARRB1	-0.11097	0.276669
CFB	0.840508	0.276666
SMC2-AS1	-0.44835	0.276626
KIAA1958	-0.0842	0.276575
LMO3	0.242576	0.276574
AC018521.5	0.279952	0.276566
RPL39L	0.119891	0.276554
SBDS	-0.08507	0.276538
LYPLA1	-0.08249	0.276526
NA	0.412145	0.27652
A2ML1	-1.63708	0.276377
LINC01621	-1.63708	0.276377
MAFTRR	0.921212	0.276372

PTPRJ	0.197602	0.276334
TM9SF3	-0.06533	0.276276
DLEC1	-0.17019	0.276257
STK32B	-2.50852	0.276213
RNU6-103P	-2.50852	0.276213
RNU6-647P	-2.50852	0.276213
RF00443	-2.50852	0.276213
MIR572	-2.50852	0.276213
AC009303.1	-2.50852	0.276213
AC079790.1	-2.50852	0.276213
LINC00159	-2.50852	0.276213
AC105450.1	-2.50852	0.276213
IFITM4P	-2.50852	0.276213
BX293535.1	-2.50852	0.276213
RPS14P8	-2.50852	0.276213
RNY4P37	-2.50852	0.276213
RNU6-1153P	-2.50852	0.276213
AC078852.2	-2.50852	0.276213
AP003385.3	-2.50852	0.276213
AC048382.4	-2.50852	0.276213
AL023803.1	-2.50852	0.276213
NA	-2.50852	0.276213
NA	-2.50852	0.276213
NA	-2.50852	0.276213
KRT18P6	1.345248	0.276188
TM7SF3	-0.07235	0.27617
AC087164.1	-0.17076	0.276165
UBE2V1	0.238497	0.27616
C20orf204	0.261657	0.27613
PHKG1	-0.28165	0.276126
NDUFV1	0.073194	0.276019
TAFA3	-1.53043	0.27601
ARID3C	-1.00314	0.275924
AL512306.1	-1.61013	0.27592
PRPH	0.16301	0.275884
KIAA1586	0.100927	0.275863
FGF11	-0.57155	0.275856
CD72	0.206994	0.275738
SP140	-1.73272	0.27571
AC092343.1	-1.73272	0.27571
SLC29A2	-0.14608	0.275669
ADPRHL1	-0.23709	0.275583
ESPNP	-1.923	0.275544
FBF1	-0.19196	0.275466
MFN2	-0.06977	0.275458
CYCSP45	1.467038	0.275455
XRCC3	-0.08985	0.275435
NA	0.2627	0.275389
CHST2	-0.6411	0.275377
ZNF526	0.131753	0.275356

WDR11-AS1	0.950699	0.27531
ZNF577	-0.19167	0.275239
CFAP126	-0.14017	0.275173
AL391832.2	-1.60715	0.275125
SGCB	-0.09271	0.275122
NA	-0.3734	0.275093
AC004264.1	-0.35663	0.275092
RF00019	-1.92079	0.275006
AL079352.1	-1.92079	0.275006
AC211433.1	-0.10257	0.274919
EML6	-0.10535	0.274903
NOM1	-0.07984	0.274871
MAGED4B	-0.93749	0.274859
PLPP4	0.141149	0.274835
LHX4	-0.11744	0.274801
SNPH	-0.14208	0.274756
GLTPD2	-0.45736	0.274717
NRSN2-AS1	0.202911	0.274665
AC025576.2	0.860627	0.27466
ZDHHC21	-0.08639	0.274624
MYCBP	0.125891	0.274624
RAP2C	0.086795	0.274597
SNRK-AS1	-0.2166	0.274596
AL353743.2	-1.25597	0.274551
LINC01229	1.473186	0.274549
DERA	0.095111	0.274511
FAM89A	0.155075	0.27449
DPM2	-0.06972	0.274479
AC079140.2	1.043811	0.274452
AP000426.1	0.282949	0.274428
DMGDH	0.937165	0.274413
OGFR	-0.08896	0.274334
YPEL1	0.11059	0.274312
G0S2	0.102713	0.274144
AC066613.2	-0.18343	0.27405
NA	-1.36991	0.27403
MAFK	0.162155	0.27401
TJAP1	-0.09432	0.273898
PTOV1	0.078389	0.273787
TEX15	0.142479	0.273718
GUCY1A2	-0.60119	0.273717
RAB4B	0.3395	0.273703
NDOR1	-0.11673	0.273636
AC020892.2	-1.26875	0.273616
YAE1	0.081167	0.273607
CREBRF	0.113364	0.273502
AC005197.1	-0.73154	0.273411
ZNF473	-0.10708	0.273381
AP001528.2	-1.56458	0.273293
MXI1	0.080846	0.273291

NDUFB1	0.144422	0.273253
MIR553	-1.7229	0.273216
AC020651.1	-0.25909	0.273197
CNTN4	-0.1305	0.273195
AP002893.1	-1.04659	0.273156
PABPN1	0.067537	0.273078
RPL7P21	1.516804	0.273067
BBS12	-0.20081	0.273064
AC002310.3	0.94314	0.273022
CBLN1	-0.17858	0.273011
AC108673.2	-1.25862	0.272973
NA	-1.04122	0.272887
LPAR5	-1.92316	0.272887
AL591885.1	-1.92316	0.272887
AC104532.2	-0.29767	0.272882
AL355032.1	1.276428	0.272875
SBF2-AS1	0.117855	0.272869
AL117329.1	0.636827	0.272861
AC007620.3	-0.78096	0.272853
GK-AS1	-0.38237	0.272853
KIAA0930	-0.08767	0.272843
AP003170.3	-0.77782	0.272812
AP001628.2	-1.65771	0.272731
RBL1	-0.136	0.272702
AC093151.3	1.651387	0.27267
XIAPP2	1.651387	0.27267
RPS6KA6	-0.13597	0.272574
PABPC3	0.939988	0.272543
GASAL1	-0.59569	0.272535
NA	0.705872	0.272529
AANAT	1.372652	0.272486
NAV2	-0.08781	0.272464
ELP5	0.076261	0.272434
AC008280.2	1.513191	0.272392
MMP21	-1.92095	0.272349
NA	-1.92095	0.272349
AC139256.1	-0.36708	0.27233
BYSL	0.084994	0.272229
HDHD5	0.077174	0.272224
JAK2	-0.12938	0.272219
AL391807.1	0.179489	0.272141
ITGA11	0.18052	0.272098
AC104117.5	0.97981	0.272085
AL356740.1	0.472514	0.272071
SGCE	0.083004	0.272045
NUDT16L1	0.094053	0.272035
LINC00562	-0.62238	0.272017
ABHD12B	-1.11907	0.271912
RCL1	-0.08897	0.271876
PRPSAP2	0.09038	0.27187

LYVE1	1.450542	0.27186
MLLT1	-0.09691	0.271803
TRIM26	-0.08722	0.271758
NDUFB10	0.081408	0.271741
AC005476.2	-0.88071	0.271733
STAM-AS1	0.3483	0.271733
ACP1	0.07188	0.27172
OPA3	-0.11065	0.27172
APEX1	0.065163	0.271693
CIAPIN1P	-1.88236	0.271619
TAF11	-0.07779	0.271598
CTNNAL1	0.111511	0.271589
WWTR1-AS1	-0.31286	0.271511
IL34	0.382308	0.271508
ZNF90	-0.19997	0.27149
AMFR	-0.11357	0.271354
SLC22A4	0.298759	0.271345
PIK3R1	-0.10955	0.2713
MIR320B2	-1.63627	0.271248
ABHD1	-0.26354	0.271203
TMCO4	-0.20503	0.271197
TRIM36	0.12612	0.271155
FREM2	-0.69863	0.271103
FAM124B	1.187901	0.271043
CFAP65	0.350109	0.271019
URAHP	0.130108	0.270998
IRF6	0.08528	0.270987
B4GALT6	-0.1372	0.270973
JAKMIP1	-0.13971	0.27097
AL645941.3	0.096634	0.270872
CLDN6	-1.46888	0.2707
MAP3K7	-0.07451	0.270673
AC007298.1	0.14111	0.27066
SPATA1	0.165173	0.270576
SYCP2	-0.19074	0.270567
RELL1	-0.22303	0.270519
TRIQK	0.118071	0.270439
NOA1	0.084685	0.270399
DICER1-AS1	0.248888	0.270395
GIPC3	0.744384	0.270328
LIN7A	-0.49218	0.270238
AL050341.2	0.152514	0.270229
AL132989.1	-0.40493	0.270199
CGREF1	-0.12006	0.270121
FABP2	2.467515	0.270115
IL20	2.467515	0.270115
MT1H	2.467515	0.270115
RF00019	2.467515	0.270115
NUDT19P3	2.467515	0.270115
RNU6-1025P	2.467515	0.270115

NDUFB4P8	2.467515	0.270115
LINC02052	2.467515	0.270115
NA	2.467515	0.270115
RPS7P4	2.467515	0.270115
TNPO1P3	2.467515	0.270115
ZBTB46-AS1	2.467515	0.270115
RPS26P42	2.467515	0.270115
AC246785.2	2.467515	0.270115
EEF1GP5	2.467515	0.270115
NA	2.467515	0.270115
GAPDHP38	2.467515	0.270115
AC104090.1	2.467515	0.270115
AC026700.1	2.467515	0.270115
IGHV3-32	2.467515	0.270115
AC092862.1	2.467515	0.270115
SHLD2P2	2.467515	0.270115
AC022166.1	2.467515	0.270115
AC106745.1	2.467515	0.270115
AC103988.1	2.467515	0.270115
AC100832.2	2.467515	0.270115
NA	2.467515	0.270115
NA	2.467515	0.270115
AC008759.2	2.467515	0.270115
PDE4A	-0.21229	0.270049
TULP4	-0.08431	0.27003
GLRB	0.181756	0.269999
CNTNAP3	-0.47305	0.269993
DCST1	0.321958	0.269978
SNRPA1	0.086185	0.269977
AL139397.1	0.924769	0.269931
SNORD100	-0.8518	0.269903
AC008771.1	-0.20843	0.269876
AC009630.2	-0.17576	0.269861
ARNT	-0.07255	0.269851
AMOTL2	-0.11912	0.269813
NA	-0.89057	0.269735
NA	-1.14774	0.269684
LRRC3	-0.26402	0.269665
TAS2R50	1.621986	0.269627
ADORA2A	-0.20015	0.269603
SUPT4H1	0.073409	0.269578
BNIP3P17	0.381112	0.269529
PTPRT	-0.83875	0.269523
NEFM	-0.07811	0.269503
LOH12CR2	-0.42659	0.269499
AC018511.2	0.287938	0.269479
ST6GALNAC3	0.084477	0.269454
CCL17	-2.45973	0.26944
NA	-2.45973	0.26944
MIR335	-2.45973	0.26944

GGNBP1	-2.45973	0.26944
AC104758.1	-2.45973	0.26944
AL513122.1	-2.45973	0.26944
NA	-2.45973	0.26944
AC073957.1	-2.45973	0.26944
AC009502.1	-2.45973	0.26944
AC098826.2	-2.45973	0.26944
ATP11A-AS1	-2.45973	0.26944
PCBP2P1	-2.45973	0.26944
NA	-2.45973	0.26944
SNORD13P1	-2.45973	0.26944
AC020917.1	-2.45973	0.26944
RPL5P3	-2.45973	0.26944
AC007370.2	-2.45973	0.26944
AC016705.1	-2.45973	0.26944
RN7SL793P	-2.45973	0.26944
TTC39C	0.123699	0.269427
AC008440.1	0.741898	0.269405
BST1	0.268287	0.269386
AL138921.1	-0.19366	0.269351
ACTR2	-0.07346	0.269309
ZNF483	-0.27522	0.269261
LRFN4	0.095397	0.26923
MAN1A1	0.221008	0.269222
AP002852.1	0.488669	0.269172
POU5F1	1.112462	0.269146
UBE2I	0.065337	0.269058
AC126407.1	-0.34575	0.269031
AL591686.1	-2.45667	0.269016
LINC01058	-2.45667	0.269016
AC093155.1	-2.45667	0.269016
AC068647.1	-2.45667	0.269016
RPL23AP63	-2.45667	0.269016
AC114741.1	-2.45667	0.269016
CAB39P1	-2.45667	0.269016
AL137804.1	-2.45667	0.269016
LINC02294	-2.45667	0.269016
AC027455.1	-2.45667	0.269016
AC019080.3	-2.45667	0.269016
AL392183.1	-2.45667	0.269016
DYNLL1	0.074256	0.268999
CFAP54	-0.9191	0.268992
AL078621.1	0.881824	0.268903
AC104564.5	0.649276	0.268901
AHNAK	0.241818	0.268888
SNORA74D	-1.88698	0.268887
AC007750.1	-0.28867	0.268779
RINL	0.217292	0.268751
SLC22A5	-0.18472	0.268664
ATP6V1C1	-0.07117	0.268638

MAD1L1	0.091653	0.268629
DYRK1B	-0.11124	0.268616
NUDT16	-0.07881	0.268613
TENT5B	-0.61842	0.268594
NPIPB4	-0.2013	0.268562
NA	0.271408	0.268557
MIR3074	0.334309	0.268547
AC004471.1	1.013652	0.268494
ATMIN	-0.0687	0.268484
ACTL8	0.699741	0.268427
AC009533.1	0.094129	0.268426
AKAP9	0.07697	0.268391
SGCG	0.857042	0.268377
AC011978.2	-0.45312	0.268376
NA	-0.34929	0.268375
AC022601.1	0.343792	0.268374
KLC1	0.084482	0.268372
BTBD3	-0.07591	0.268365
NA	0.271571	0.268295
ARHGAP19-SLIT1	1.604003	0.268276
PPID	0.083753	0.26826
QTRT2	0.086864	0.268239
ATF7IP	-0.08485	0.268208
LYPD6B	0.539444	0.268154
GCNT2	0.152278	0.267993
GSTA4	0.079926	0.267916
PCDH17	0.23192	0.267903
CBWD6	-1.88246	0.267798
NA	-1.88246	0.267798
NA	0.265687	0.267795
KITLG	-0.13271	0.26779
MROH9	-0.8144	0.267785
TXNDC16	-0.11142	0.267752
PCDHGB7	-0.25063	0.267725
GDF7	0.232934	0.267621
ARRDC1	0.114046	0.267602
NA	-0.44677	0.267575
WNT10B	1.007635	0.267557
AC112722.1	-0.57169	0.267546
HAGLR	0.113107	0.26754
AC011773.1	0.31946	0.267429
ZSCAN5A	0.174001	0.267412
PEX6	-0.09854	0.267313
ABRACL	0.150837	0.267285
AC063926.1	-0.33356	0.267241
AC104117.2	0.182529	0.267237
AC078883.1	-0.36806	0.267177
SLC16A4	0.25623	0.26713
WEE2-AS1	-0.21372	0.267122
IKBIP	-0.09834	0.267039

LY6K	0.901869	0.267013
NAP1L1P1	0.924386	0.266977
GPRIN3	0.322501	0.266964
NGEF	-1.04313	0.266928
GCKR	-1.04313	0.266928
AC023055.1	-0.17049	0.266897
DIP2C	-0.1052	0.266794
PLEK	-1.16718	0.266784
LRP5L	0.182963	0.266766
AC107982.2	-0.61501	0.266735
CEBPZ	0.084869	0.266726
CNOT10-AS1	-0.21202	0.266721
AL009174.1	0.687931	0.266712
S100A6	-0.09358	0.266693
NA	-0.19241	0.266681
BET1L	-0.0879	0.266625
AC012306.2	0.186802	0.266587
AIRE	-0.36644	0.266585
NA	1.284225	0.266573
OSCAR	0.898204	0.266473
RTF1	0.069152	0.26647
AL353689.2	-0.14965	0.266445
FABP3	0.412835	0.266428
C21orf62	0.863036	0.266325
SLC44A2	-0.10391	0.26629
PSD	0.108754	0.266245
FOXD4	-0.26297	0.266227
CEL	-0.39434	0.266199
ABCC6	-0.31601	0.266188
PZP	1.138524	0.266163
CCDC107	0.133725	0.266107
FBXW4P1	-0.86977	0.266057
SNORA15B-2	-1.11683	0.266038
RAB11B	-0.07252	0.266024
PRXL2A	-0.07157	0.266006
DPY19L1	-0.09274	0.266003
CAST	-0.07531	0.265929
PCTP	0.119986	0.265906
SPTLC2	-0.07757	0.265903
NPFFR1	2.43692	0.265888
MEI1	2.43692	0.265888
PRSS57	2.43692	0.265888
GOLGA6L3	2.43692	0.265888
RNU6-136P	2.43692	0.265888
RNU2-22P	2.43692	0.265888
AC012512.1	2.43692	0.265888
AC092168.1	2.43692	0.265888
AC017007.2	2.43692	0.265888
FGFR3P5	2.43692	0.265888
LINC01735	2.43692	0.265888

ALG1	-0.08474	0.265867
AC108063.1	-1.51068	0.265825
DNAJA4	0.323948	0.265818
TSPAN3	0.060973	0.265799
USP7	-0.06826	0.265758
AC000068.1	0.302925	0.26572
AL031772.1	0.505047	0.265691
ID2-AS1	0.284267	0.265688
ACTB	-0.06932	0.265676
NDRG2	-0.12024	0.265671
AC009902.2	0.124324	0.26566
SRPRA	-0.08796	0.26565
GOLGA1	-0.0883	0.265643
CCL20	1.049349	0.265592
AC083964.1	0.23358	0.265579
AL035427.1	0.204258	0.26555
ENOX2	0.114434	0.265549
AC023389.1	-0.36005	0.265542
YPEL5P2	1.716272	0.265528
RPGR	0.126653	0.265506
HERC2P3	0.096956	0.265501
CLTRN	-0.58112	0.265488
PROCA1	-0.19811	0.265476
AC010136.1	0.51561	0.265443
ATF6B	-0.07888	0.265401
AL441992.1	-0.42588	0.265387
SNORA2A	-1.08643	0.26537
AL353150.1	0.256441	0.26527
AL354893.2	0.62837	0.265251
CCDC90B	0.076404	0.265176
HOMER1	0.113148	0.265141
MB21D2	0.118002	0.265139
MAP3K9	-0.1278	0.265131
USP39	-0.07604	0.265117
MT-ATP6	0.062017	0.265112
RN7SL128P	-1.88253	0.265104
PYURF	0.097807	0.265091
VDAC2	-0.06283	0.26506
ZNF486	-0.12681	0.265018
NUDT12	0.10489	0.264949
HNRNPLL	0.063541	0.264935
LCMT2	-0.0899	0.264915
MYH7B	-0.34142	0.264866
AL096870.1	-0.09026	0.264836
AL671883.2	0.324126	0.264765
AKAP12	-0.09499	0.264749
WNT5A-AS1	-0.60385	0.264701
HSPA8P15	0.437077	0.264681
MTG2	-0.08164	0.26463
ZFP14	0.108511	0.264592

UBE2QL1	-0.10208	0.26454
AC009292.1	0.855259	0.264481
ADGRF4	0.664414	0.264461
AOAH	-0.94902	0.264446
AC012464.1	0.790363	0.264373
AC104333.3	-1.11236	0.264344
WASL	-0.06795	0.264321
GSR	-0.07231	0.264264
PNPLA1	1.487015	0.26423
ATP6V0E2-AS1	0.112412	0.264177
RAC2	-0.36085	0.264176
AC004969.1	-0.79267	0.264128
DNAH7	-0.34425	0.264071
AL359715.2	-0.3436	0.264001
AL121574.1	1.109383	0.263995
TMEM173	-0.37639	0.263974
EVC	-0.07673	0.263968
ITM2A	0.666169	0.263873
IFT22	0.087991	0.26386
GPR137B	0.118224	0.263857
Z83844.1	0.088882	0.263854
Z98884.1	-0.49649	0.263841
ATG12	0.078294	0.263746
DOCK9-DT	0.767052	0.263732
NPM1P24	1.12673	0.263672
RPL21P11	1.231381	0.263669
CNTN1	0.096551	0.263666
AC012313.6	-0.42128	0.263656
MSL3	0.085311	0.263618
SAXO2	-0.33061	0.263593
SOCS5	0.084037	0.263532
ZNF195	-0.07024	0.263522
AC008870.3	-0.72276	0.263515
ZNF256	0.183574	0.263476
ABHD17C	0.153976	0.263423
SPOCD1	-0.18327	0.263404
AC093582.1	-1.1252	0.26337
NDC80	-0.11527	0.263352
ANKRD11	-0.08965	0.263346
ARMCX5-GPRASP2	0.221908	0.263345
PTGS2	0.200931	0.2633
ALS2CL	0.287445	0.263248
SERPINI1	0.194806	0.263247
SUZ12P1	0.115724	0.263214
R3HDM1	-0.07916	0.263141
GLO1	0.077236	0.263124
AC024361.1	0.488337	0.263068
NA	0.128266	0.263066
RPL5P4	0.541574	0.263064
ZNF767P	0.09974	0.263062

Z69733.1	-0.33705	0.263054
CUX1	-0.0746	0.263024
MIA3	0.065343	0.262989
DAW1	2.415394	0.262926
CXCL13	2.415394	0.262926
RF00494	2.415394	0.262926
RF00432	2.415394	0.262926
MIR576	2.415394	0.262926
CYCSP24	2.415394	0.262926
AL033504.1	2.415394	0.262926
AL391840.1	2.415394	0.262926
AC107072.2	2.415394	0.262926
NA	2.415394	0.262926
RPSAP5	2.415394	0.262926
RNA5SP462	2.415394	0.262926
SUGT1P4	2.415394	0.262926
AC092868.3	2.415394	0.262926
AC140912.1	2.415394	0.262926
NA	2.415394	0.262926
NA	-0.74995	0.262845
PNPLA6	-0.09818	0.26283
MGMT	0.146062	0.262758
C5orf51	0.069062	0.262739
NA	0.798381	0.262735
RPSAP15	0.257349	0.262719
AC105275.1	-1.24819	0.262704
RAVER1	0.113055	0.26267
SPRY2	0.090763	0.262665
SLC44A1	-0.07729	0.262647
WIPF2	-0.09309	0.262616
TCIRG1	-0.33435	0.262613
CHEK2	0.112002	0.262574
ORC6	-0.11125	0.262536
PPIAP16	-2.40956	0.262529
C16orf90	-2.40956	0.262529
SNORD111B	-2.40956	0.262529
SNRPFP4	-2.40956	0.262529
AL161747.1	-2.40956	0.262529
AC243547.1	-2.40956	0.262529
AL133153.1	-2.40956	0.262529
AC079075.1	-2.40956	0.262529
AC027237.5	-2.40956	0.262529
AC006116.4	-2.40956	0.262529
AC011447.4	-2.40956	0.262529
NA	-2.40956	0.262529
AL360012.1	-2.40956	0.262529
WASH3P	0.110061	0.26241
SUGT1	0.078472	0.262391
RNASEH1-AS1	0.095541	0.262317
LRGUK	0.264437	0.262308

AP000802.1	-0.20054	0.26228
AC110285.4	-0.42164	0.262279
AC079226.1	-0.28632	0.262214
AL031848.2	-0.57131	0.262214
TXNL4B	-0.09242	0.262201
CRH	-1.19413	0.262185
AL138787.2	-0.3842	0.262121
RASSF8	0.068801	0.26205
DPH6-DT	0.346891	0.262048
AC021231.3	1.883432	0.262025
AC139769.2	-0.1306	0.262012
CLDND2	0.634165	0.262004
UBE2V2P3	0.780626	0.26199
UTP23	-0.07834	0.261964
RNF130	-0.08663	0.261949
KIF25-AS1	-0.41375	0.261935
OLFM3	-0.13625	0.261899
CDKN2D	-0.18564	0.261893
USP18	0.335344	0.26187
RPS18P9	0.605121	0.261866
SNORD53B	-1.85026	0.261866
PAK4	-0.08588	0.261822
SEC24B	-0.08102	0.261806
AL050343.2	-1.21611	0.261684
PLA2R1	-2.40324	0.261663
RF00019	-2.40324	0.261663
HNRNPCP6	-2.40324	0.261663
RPL7L1P9	-2.40324	0.261663
RPEL1	-2.40324	0.261663
NA	-2.40324	0.261663
MTND4LP14	-2.40324	0.261663
AC109454.1	-2.40324	0.261663
RF00019	-2.40324	0.261663
AC012213.2	-2.40324	0.261663
DHX40P1	-2.40324	0.261663
RRP7BP	-0.17878	0.261624
NA	0.752044	0.261609
FBXW9	0.107793	0.261594
NA	-0.54131	0.261557
PCDHGA8	-1.05457	0.261556
NA	0.77511	0.261394
AL512310.4	0.540795	0.261343
AC046134.2	0.290254	0.261189
AP002498.1	-0.51072	0.261184
CMYA5	0.511269	0.261177
TMEM267	0.151035	0.261169
ZCCHC9	0.097943	0.26115
PSMA7	0.068471	0.261124
PLXNC1	-0.18237	0.261112
NA	0.876287	0.261091

MKLN1-AS	-0.20571	0.261089
ZNF671	0.500721	0.261083
RF00019	1.136346	0.261065
AL589765.5	-0.37535	0.261063
SRD5A1	-0.08717	0.261044
SLC12A2	0.093567	0.260921
AL591623.1	1.230784	0.260841
CINP	-0.09095	0.260836
CELF1	-0.05878	0.260765
AC090695.1	0.921397	0.260756
AC015961.2	0.412354	0.260708
SPDYE2	0.181194	0.260699
Z95115.1	0.150977	0.260695
KCTD7	0.110059	0.260636
NA	0.051182	0.260599
HIST1H2AC	-0.09806	0.260569
MYBL1	-0.14314	0.260482
SOX4	0.076927	0.260385
ZNF552	-0.16449	0.260367
CASK	0.09397	0.260335
LINC01767	1.397208	0.260335
AC079944.2	1.397208	0.260335
TMEM179	-0.13672	0.260332
SKOR1	0.25301	0.260298
AC012676.1	0.180343	0.260281
CCDC36	-1.96018	0.260272
RNA5SP354	-1.96018	0.260272
NA	-1.96018	0.260272
UBE2V1P13	-1.96018	0.260272
E2F6P2	-1.96018	0.260272
GMCL2	-1.96018	0.260272
AC114488.3	-1.96018	0.260272
AC091167.4	-1.96018	0.260272
BBIP1P1	-1.96018	0.260272
Z93930.3	-1.96018	0.260272
SCOCP1	-1.84332	0.26024
RENBP	-0.29061	0.26023
CLMAT3	0.151603	0.260177
STRADA	0.136379	0.260155
CSAG1	-0.12086	0.260111
GLYCTK	0.115162	0.260064
MSRA	0.172996	0.260028
SCNM1	0.085111	0.260025
DRAP1	-0.06473	0.26001
RUSC1-AS1	0.062341	0.259977
ABRAXAS2	0.091696	0.259948
USP5	-0.07618	0.259826
ERICH1	-0.10906	0.259824
CDKN2A	-0.26493	0.259687
AL353743.1	-0.20552	0.259609

OSTC	0.079795	0.259522
ZFP36	0.197991	0.259464
DDX5	0.055734	0.259454
AL365223.1	0.71746	0.259449
IDUA	0.129473	0.259449
ICAM5	0.25145	0.259434
LATS2	-0.37183	0.259425
AP1S2	0.086975	0.259425
AL160270.2	1.860316	0.259387
KRT8P36	-1.37537	0.259369
TMEM74	0.941638	0.259368
AC006557.4	-0.6994	0.259294
NA	0.462703	0.259172
TMEM215	-0.25296	0.259169
DDX11L10	-1.32068	0.259167
AC027644.3	-0.16569	0.259148
NSA2	0.078177	0.259086
AC022509.1	-0.27202	0.259084
RNPS1	-0.06449	0.259067
LINC01376	-0.55034	0.259062
PPIP5K1	-0.1277	0.259024
AL445248.1	1.020662	0.25902
GOLGA7	0.083883	0.259017
ASNS	0.083262	0.258969
ZNF559	-0.12225	0.258952
AP3B2	-0.08206	0.258946
ADGRL2	-0.06535	0.258887
NDNF	-0.32083	0.258882
ELMOD3	0.117625	0.258871
MIR762HG	-0.1118	0.258871
JSRP1	0.07327	0.258698
APOL3	-0.71658	0.258698
AC025031.1	1.197539	0.258677
AL078612.1	-0.11929	0.258638
SAMD15	-0.2707	0.25859
AC009113.1	-0.17034	0.258572
NKX3-1	-0.47061	0.258527
DIP2B	-0.09597	0.258524
AL133410.2	0.142029	0.258518
LINC02315	0.619252	0.258505
OXR1	0.169447	0.258439
UBE2O	-0.08923	0.258436
ATP5PBP5	-0.71293	0.258375
TOM1L2	-0.10558	0.258363
RN7SKP74	0.769459	0.258362
HYLS1	-0.17417	0.258359
ATG9A	-0.09525	0.258353
ALOXE3	-1.19616	0.258302
AC079922.2	0.447482	0.25822
AC114956.1	1.040892	0.258172

AC084756.1	0.22918	0.25808
ARRB2	0.088536	0.258073
KLHL17	-0.09958	0.258071
GABPB1-AS1	0.083477	0.258047
AL354983.1	-1.27027	0.258033
PSMG1	0.093702	0.258033
E2F6P3	-1.66048	0.258024
ASAH2	-0.22811	0.257992
AL157938.2	0.177954	0.257963
C16orf71	0.350695	0.257885
AL136984.1	1.189942	0.257879
AC010547.1	-0.58724	0.257817
AC023983.1	-0.64179	0.257809
AL356489.2	0.161829	0.257795
RBMX	-0.06498	0.257792
AP000757.1	-0.10355	0.25777
AC009690.2	0.091268	0.257707
AC007229.1	-1.33956	0.257699
LRRC8C-DT	0.208429	0.257662
PPIAP29	-0.52189	0.257603
ZNF146	0.06984	0.257528
AC026471.2	-1.02762	0.257512
LRRIQ1	-0.27861	0.257508
AC025884.2	-0.57234	0.257487
AL117336.2	-0.28613	0.257452
SLC27A2	-0.49426	0.257443
TARID	-0.92936	0.257435
RBPJ	0.062669	0.257412
AL442663.3	-0.1626	0.257339
RF00019	0.346363	0.257318
EMP1	-0.26655	0.257242
CAPRIN1	0.05939	0.257223
ASH2L	-0.07923	0.257207
RDH14	-0.0987	0.257131
EPC2	0.084076	0.257072
GRIA4	-0.25351	0.257056
AC007336.1	0.605217	0.257039
C1orf112	-0.08313	0.256996
AC009061.2	-0.32298	0.256974
PIGB	-0.13789	0.256919
AC145207.8	0.145987	0.256906
ZNF578	-0.29754	0.256904
CAMSAP3	-0.3093	0.256838
RN7SL333P	-0.94291	0.256837
ASMTL	-0.129	0.256835
RUNDC3A-AS1	0.142021	0.256809
AK3	-0.07323	0.256789
AL359073.1	-1.29515	0.256777
AKR7A2P1	0.902871	0.25677
AC105137.1	1.860162	0.256711

PPARGC1A	-1.96046	0.256711
C5orf52	-1.96046	0.256711
RNU6-529P	-1.96046	0.256711
AP000580.1	-1.96046	0.256711
SEPT14P24	-1.96046	0.256711
HNRNPA1P29	-1.96046	0.256711
RNU6-893P	-1.96046	0.256711
RF00573	-1.96046	0.256711
QRSL1P3	-1.96046	0.256711
AC005520.1	-1.96046	0.256711
SPDYE22P	-1.96046	0.256711
AL157871.2	0.765658	0.256688
AC010931.2	0.090927	0.256663
PTGES3P1	0.367276	0.256634
PALMD	-0.17669	0.256556
LYPD3	1.860153	0.256546
NR5A1	1.860153	0.256546
AC087632.1	1.860153	0.256546
HMGN2P17	-1.84327	0.2565
AC127522.1	-1.84327	0.2565
AC079328.1	-1.84327	0.2565
AC108451.1	-0.3102	0.256426
Z97634.1	0.257395	0.256307
AL049552.1	-0.48107	0.256275
AL022313.4	0.568988	0.256271
PSAT1	0.082225	0.256239
SLCO4A1	-0.15718	0.25623
LSM8	0.085321	0.256218
AC006213.2	-0.299	0.256179
AL139286.2	0.526506	0.256112
NA	1.188444	0.256061
AC016999.1	-0.29758	0.256023
SNHG19	0.151384	0.256001
AC006058.3	-0.39734	0.255993
AC024560.3	-0.6873	0.255977
SESN2	0.128054	0.255955
AL589765.7	0.110598	0.255894
AC004943.1	0.937186	0.255825
TTC36	0.446078	0.255806
STAG3L1	-0.20202	0.255689
TCF3P1	-1.43054	0.255659
HDAC6	-0.08169	0.255654
NA	-1.05733	0.2556
NGB	2.361668	0.255581
NPIPA5	2.361668	0.255581
AC064847.1	2.361668	0.255581
ASS1P1	2.361668	0.255581
AL354890.1	2.361668	0.255581
EFCAB6-AS1	2.361668	0.255581
PNMA6A	2.361668	0.255581

BX664727.3	2.361668	0.255581
AL157935.2	2.361668	0.255581
AC126773.2	2.361668	0.255581
AC011247.2	2.361668	0.255581
ERP44	0.078108	0.255574
C12orf80	-1.85022	0.255484
PON3	-2.35793	0.255477
TRPM2	-2.35793	0.255477
CYBB	-2.35793	0.255477
PTCHD3	-2.35793	0.255477
TNFAIP8L3	-2.35793	0.255477
FBXL22	-2.35793	0.255477
RNU6-780P	-2.35793	0.255477
AURKAP1	-2.35793	0.255477
AL512633.1	-2.35793	0.255477
AC011447.1	-2.35793	0.255477
AL663023.1	-2.35793	0.255477
CHL1-AS1	-2.35793	0.255477
AL139339.1	-2.35793	0.255477
BCRP5	-2.35793	0.255477
AL589765.3	-2.35793	0.255477
NA	-2.35793	0.255477
AL445190.1	-2.35793	0.255477
RPL31P58	-2.35793	0.255477
AC022101.1	-2.35793	0.255477
AC124276.1	-2.35793	0.255477
AC126177.4	-2.35793	0.255477
AC099518.2	-2.35793	0.255477
NA	-2.35793	0.255477
NA	-2.35793	0.255477
MIR3609	-2.35793	0.255477
NA	-2.35793	0.255477
NA	-2.35793	0.255477
ATP1B3P1	-2.35793	0.255477
DCXR-DT	-0.86121	0.255468
AL163540.1	-0.1888	0.255466
BLOC1S6	-0.07449	0.255435
STARD4-AS1	-0.06559	0.255425
OTOR	0.290914	0.25541
VWA1	0.203419	0.255381
DLK2	-0.27145	0.255374
MCM7	0.056377	0.25532
LRRC8B	0.103431	0.255291
CXorf56	0.079754	0.25529
LINC01252	0.37475	0.255279
STIM2	-0.07996	0.255239
RNF216P1	-0.10013	0.255178
NFE2L1	-0.06101	0.255177
NYAP2	0.980773	0.255164
PSMC3IP	0.118017	0.255162

AQP1	0.129701	0.255152
ATF7-NPFF	-0.72362	0.255145
EDC4	0.080897	0.255117
VCAN-AS1	0.124778	0.255081
MIR4307HG	-1.11099	0.255075
UBR2	-0.07506	0.25506
PDZRN4	-2.35468	0.255035
NA	-2.35468	0.255035
RF00019	-2.35468	0.255035
NA	-2.35468	0.255035
AL121983.2	-2.35468	0.255035
AL354928.1	-2.35468	0.255035
CCDC39-AS1	-2.35468	0.255035
RPL35P6	-2.35468	0.255035
LINC02112	-2.35468	0.255035
NA	-2.35468	0.255035
AC239800.3	-2.35468	0.255035
C1QTNF3-AMACR	-2.35468	0.255035
PTRH1	-0.14467	0.255012
AC026470.1	-0.08766	0.255002
COMMD1	-0.14564	0.25498
FBXW7	0.080733	0.254971
TAC3	-0.34436	0.254948
DERL1	-0.06562	0.254892
AL133415.1	-0.08407	0.254872
ANKRD53	1.486729	0.254848
AC012358.1	-0.60489	0.254848
SUMO2P19	1.551668	0.254833
TM4SF19	-1.42741	0.254828
AC105001.1	-0.79427	0.254819
AL049597.1	1.489048	0.254811
NADK2	0.088612	0.254755
RAB27A	-0.14581	0.254705
AC005828.6	-1.15901	0.254703
PEX16	0.105963	0.254666
CCDC192	0.373896	0.254666
GMPR	-0.28081	0.254653
SEC61G	0.097552	0.254642
NA	-2.35142	0.254593
MIR320D1	-2.35142	0.254593
PTCHD3P2	-2.35142	0.254593
AC092423.1	-2.35142	0.254593
NA	-2.35142	0.254593
NA	-2.35142	0.254593
RNU6ATAC24P	-2.35142	0.254593
AP001266.2	-2.35142	0.254593
MIR548AO	-2.35142	0.254593
NA	-2.35142	0.254593
AC003682.1	-2.35142	0.254593
AC069148.1	-2.35142	0.254593

AL162151.2	0.651023	0.254582
POLE3	-0.07209	0.254565
RSL24D1	0.074541	0.254545
FBXL3	0.077884	0.254499
ATP11A	-0.10736	0.254434
ZMYND11	-0.06841	0.254399
MMP1	-0.48819	0.254374
MDH1	0.076212	0.254354
CLDN11	-0.17774	0.254347
ZNF292	0.074421	0.254287
LUC7L	0.061501	0.254266
WWP1	-0.10829	0.254221
FOXJ1	-1.39322	0.25422
ACAP2-IT1	-0.55013	0.254212
CPNE6	-1.96066	0.254206
FGL2	-1.96066	0.254206
KCNS2	-1.96066	0.254206
LENEP	-1.96066	0.254206
OXGR1	-1.96066	0.254206
ATP4B	-1.96066	0.254206
RNY1P6	-1.96066	0.254206
MIR199B	-1.96066	0.254206
NA	-1.96066	0.254206
RN7SKP151	-1.96066	0.254206
IL9RP3	-1.96066	0.254206
AC013287.1	-1.96066	0.254206
AP000695.1	-1.96066	0.254206
NA	-1.96066	0.254206
NA	-1.96066	0.254206
AC008013.2	-1.96066	0.254206
AC004494.1	-1.96066	0.254206
VN1R80P	-1.96066	0.254206
AL161669.2	-1.96066	0.254206
DNAJB2	0.080742	0.25417
SNORA46	-1.20942	0.254168
AQP7	-2.34816	0.25415
TBATA	-2.34816	0.25415
C19orf33	-2.34816	0.25415
XIAPP3	-2.34816	0.25415
COL18A1-AS1	-2.34816	0.25415
FAM86MP	-2.34816	0.25415
RNU1-47P	-2.34816	0.25415
NA	-2.34816	0.25415
RPS10P14	-2.34816	0.25415
AL356124.1	-2.34816	0.25415
AC009238.1	-2.34816	0.25415
RPS26P39	-2.34816	0.25415
AL157834.1	-2.34816	0.25415
Z99943.1	-2.34816	0.25415
ST13P18	-2.34816	0.25415

AC114814.3	-2.34816	0.25415
LINC01010	-2.34816	0.25415
RPL21P88	-2.34816	0.25415
NA	-2.34816	0.25415
RPS3AP14	-2.34816	0.25415
NA	-2.34816	0.25415
AC104806.2	-2.34816	0.25415
RNU6-1150P	-2.34816	0.25415
AP003469.1	-2.34816	0.25415
AC022217.3	-2.34816	0.25415
AC125616.1	-2.34816	0.25415
RPL29P33	-2.34816	0.25415
LINC02313	-2.34816	0.25415
AL049870.1	-2.34816	0.25415
SPCS2P1	-2.34816	0.25415
AC073429.2	-2.34816	0.25415
NA	-2.34816	0.25415
MIR4771-2	-2.34816	0.25415
NA	-2.34816	0.25415
MAB21L1	0.065028	0.254129
AKAP13	0.066991	0.254084
AC006942.1	-0.11955	0.254079
FCHO1	-0.21349	0.254053
TLE1	-0.07546	0.253996
TNPO3	-0.06565	0.253986
NA	-1.80807	0.253962
AL022157.1	-0.77895	0.253897
AL513523.2	-1.28138	0.253873
PVT1	-0.20553	0.253868
RNU6-1266P	-1.84324	0.253864
AC245140.1	-1.84324	0.253864
AC091516.1	-0.9554	0.253853
SNRPB	0.075378	0.25385
AC109597.2	-0.48372	0.253782
AL033527.2	1.260293	0.253776
HOMEZ	-0.14434	0.253769
AC005332.1	-0.61259	0.253684
MUS81	0.078584	0.253673
FBXO6	-0.41766	0.25367
DAP3P1	-1.02481	0.253658
NSMF	-0.06008	0.253576
CMTM4	-0.08007	0.25357
UBA3	0.079644	0.253537
NA	1.396842	0.253491
CMC1	-0.07502	0.253483
GLB1	-0.08487	0.253383
ARID1B	-0.07287	0.253306
RAD18	-0.09885	0.253298
TESMIN	0.203247	0.253274
MED31	-0.09617	0.253184

CAPS	-0.13774	0.253142
DDAH1	-0.09073	0.253136
CTSV	-0.14572	0.253124
LVRN	0.84124	0.253117
RNU4-4P	1.459715	0.253038
AC116914.1	1.260179	0.253037
AC008267.3	-0.60426	0.253029
AL162727.1	-0.90124	0.25299
TMEM256	0.18099	0.25293
VPS41	0.061562	0.252926
HEXIM1	-0.07657	0.252921
LINC02210	0.123409	0.25291
NA	0.947766	0.252854
AC234582.1	-0.07633	0.25283
LURAP1	0.290869	0.252812
CASP3	-0.08596	0.252782
TIAL1	-0.06431	0.252769
NIPSNAP2	0.075244	0.252684
MT-ND3	0.080257	0.252655
SEPT7P3	-0.9116	0.252653
NA	-0.21786	0.252623
CFAP58	1.457344	0.252592
VN1R48P	1.457344	0.252592
TBCD	-0.06645	0.252549
HNRNPA3P11	0.831654	0.252548
FNTA	0.066933	0.252539
CD46	-0.05942	0.252355
AL590133.2	0.058994	0.252345
PTPRC	-1.03385	0.252323
OXER1	1.060783	0.252319
RBM3	-0.05957	0.252293
TSC22D2	0.106216	0.252277
POLR1B	0.06282	0.252264
AC025180.1	1.294071	0.252259
HNRNPH3	0.063142	0.252109
AL031717.1	0.536618	0.25208
RHOG	0.095325	0.252014
NREP	0.066137	0.251963
RWDD4P2	0.184886	0.251958
TCEAL7	0.080368	0.251945
CLDN3	1.201202	0.25194
MIR548N	1.453659	0.251927
EXOC6	-0.11745	0.251925
SUB1P1	-1.43113	0.251893
RNU6-856P	-1.43113	0.251893
METTL4	0.115418	0.251888
PYROXD1	0.084183	0.251877
CARD9	0.09946	0.251862
CYHR1	-0.07667	0.251814
PAXIP1-AS1	0.169519	0.251776

SOX12	0.087069	0.251764
PFKFB1	-0.68952	0.251758
FGF14-AS2	0.329294	0.251752
ZNF180	-0.12433	0.251712
MIER2	-0.10121	0.251701
RSPO4	0.433824	0.251584
AL033379.1	-0.76899	0.251527
AC023794.6	0.424287	0.251473
LONRF1	0.163798	0.251466
TTC8	0.060515	0.251465
CUEDC1	0.087441	0.251441
PUM1	0.061187	0.251421
GAPDHP20	1.656994	0.251419
FUNDC2P1	1.656994	0.251419
AC068722.1	1.656994	0.251419
NA	1.656994	0.251419
MT-TE	-0.11925	0.251379
LINC01619	0.362968	0.251379
KCNIP1	1.195572	0.251371
AC020978.3	-1.36076	0.251363
ARID3B	0.110497	0.251273
CCNE2	-0.11761	0.251269
ALG1L	0.390049	0.251107
TCP1P3	1.039162	0.251088
PDGFRA	-0.30797	0.251055
SMYD5	-0.0979	0.251015
FAM91A1	-0.06954	0.251012
DCXR	-0.08464	0.250992
HPCAL4	-0.12309	0.250982
MRPL35	-0.07709	0.250969
AC093635.1	0.465052	0.250921
PITPNA	-0.0746	0.250912
AC020658.1	1.226548	0.250859
ATP5MDP1	1.07654	0.250859
ERP29	0.065106	0.250846
LYPD1	-0.18181	0.25083
AC018647.1	-1.04277	0.250828
ESPN	-0.56356	0.250816
BRMS1	0.083198	0.250814
CYP2W1	-0.51298	0.250804
NA	0.743186	0.250802
NA	-0.28307	0.250799
CCDC61	0.194547	0.250714
NALCN	0.832387	0.250701
PDE8B	-0.14333	0.250683
GNPDA2	-0.07959	0.250642
NA	0.085868	0.250631
PANX1	-0.08304	0.25063
ABCC6P1	-0.85498	0.250595
MTRNR2L4	0.992624	0.250551

NDUFA13	-0.21494	0.250519
EGFLAM	0.639683	0.250505
AC133548.2	0.961988	0.250491
ABCC3	0.129409	0.250466
BGLAP	0.52707	0.250457
HOXD10	0.394642	0.250414
SEMA4D	0.108874	0.250383
AC012074.1	1.088613	0.250379
IRF7	-0.15966	0.25036
NEURL1B	0.126858	0.250337
NA	0.205415	0.250289
MBOAT4	-0.34166	0.250253
CHCHD2P6	0.876961	0.25024
PNRC1	0.108161	0.250187
CROCCP2	0.081793	0.250184
GTF2H1	-0.07892	0.250179
PKP4	-0.07497	0.250145
HPSE	1.648811	0.250135
MIA2	-0.44328	0.250125
CCL2	-0.06584	0.250109
RF00019	-1.37001	0.250101
LLPH-DT	-0.29029	0.250099
YBEY	0.121575	0.250097
HNRNPUP1	0.952934	0.250089
CHM	0.074279	0.250089
MYLK-AS2	-1.15704	0.250057
C1orf229	-0.26154	0.250049
NA	-0.85946	0.25004
BID	0.073722	0.250033
ARFGEF2	-0.08129	0.249992
GPR176	-0.10188	0.249991
URB1	-0.07112	0.249975
ZNF425	0.147008	0.249974
NA	-1.50663	0.249958
LINC01248	1.15781	0.249921
RPL21P43	1.15781	0.249921
LUARIS	-1.26104	0.249878
SELENOO	0.09742	0.249869
WRN	0.080368	0.249861
HIST1H1C	-0.12026	0.249815
RUNDC3B	0.216825	0.249805
SEC11A	0.071696	0.249788
C12orf75	-0.09551	0.24978
CENPT	0.066949	0.24978
LRRC36	0.906403	0.249775
TMEM254-AS1	0.298298	0.249704
HIST1H3J	0.595071	0.249701
TUBB2BP1	-1.03505	0.249623
TRIM54	-1.02041	0.24954
ST6GAL2-IT1	0.67443	0.249522

RNF25	-0.09423	0.249515
TOMM5	-0.25426	0.249512
RIMKLBP1	-1.47232	0.249511
ACADM	-0.07554	0.249422
CYP2D7	-0.75737	0.249417
MEIS3	0.107732	0.249407
CISD1	-0.11299	0.249402
FANCD2OS	-0.16114	0.2494
HNRNPDL	0.057504	0.249377
NOP56	0.062614	0.249343
NA	-1.19766	0.249342
GNS	-0.07487	0.249319
AC145285.3	1.13853	0.249295
AL592114.3	0.734043	0.249261
AP3S1	0.081598	0.249255
KTI12	0.122266	0.249176
MDM2	-0.06205	0.249126
NOXA1	-0.41277	0.249104
IQCG	-0.1599	0.249084
PURA	-0.10283	0.249059
NA	-1.30123	0.249048
AC046195.1	1.502053	0.249006
RIC1	0.079413	0.249004
NA	-0.37753	0.248943
NA	0.772677	0.248927
WDR60	-0.08472	0.248918
AL592161.1	0.887791	0.24888
LINC00944	-0.82073	0.248853
RBP2	-1.81017	0.24884
WHSC1L2P	-1.81017	0.24884
AC009955.4	-0.24444	0.24883
SF3B6	0.076716	0.248766
STX16-NPEPL1	-0.50628	0.248721
EXOC1L	-1.27829	0.248681
GPAA1	-0.0842	0.248677
PRKG1	-0.12409	0.248586
MIR3685	-0.44958	0.248556
DAD1	0.073582	0.248542
NA	0.473632	0.248542
ACADS	0.247288	0.248527
PEG10	-0.07159	0.248472
KLHL42	-0.06154	0.248471
RPL7AP66	0.783525	0.248433
CRCP	0.081206	0.248398
MED28P3	-1.1643	0.248395
CEP89	-0.08713	0.248384
MGME1	0.092898	0.248346
EI24	-0.08078	0.248243
SCN7A	-0.15781	0.248216
IDI1	0.094533	0.248159

CRYBA2	-0.47265	0.248086
ADGRE3	2.306259	0.248076
SEPT12	2.306259	0.248076
RF00019	2.306259	0.248076
NA	2.306259	0.248076
RNY4P17	2.306259	0.248076
SNORA30	2.306259	0.248076
NA	2.306259	0.248076
RNU4-69P	2.306259	0.248076
AL590762.3	2.306259	0.248076
AF064860.1	2.306259	0.248076
OSTCP8	2.306259	0.248076
AL450263.2	2.306259	0.248076
NA	2.306259	0.248076
AC113340.1	2.306259	0.248076
BX284632.1	2.306259	0.248076
AC024560.1	2.306259	0.248076
NA	2.306259	0.248076
NA	2.306259	0.248076
AC112482.1	2.306259	0.248076
CEBPA	2.306259	0.248076
OSMR-AS1	2.306259	0.248076
AC007370.1	2.306259	0.248076
AP003696.1	2.306259	0.248076
AC120193.1	2.306259	0.248076
AP003467.2	2.306259	0.248076
CDH12P1	2.306259	0.248076
PSMA2P1	2.306259	0.248076
AL136146.1	2.306259	0.248076
LINC01479	2.306259	0.248076
AC078962.2	2.306259	0.248076
NPM1P43	2.306259	0.248076
AP002761.4	2.306259	0.248076
AP005233.2	2.306259	0.248076
MIR548AX	2.306259	0.248076
NA	2.306259	0.248076
MIR4520-1	2.306259	0.248076
MIR4307	2.306259	0.248076
AC068473.2	2.306259	0.248076
NA	2.306259	0.248076
AC091393.1	2.306259	0.248076
NA	2.306259	0.248076
TRAM1L1	0.152061	0.248064
AL035071.1	-0.20294	0.248012
SPINK4	1.341601	0.247994
KCNJ2	-0.12064	0.247992
MRPL54	0.092991	0.247975
SVOP	0.160605	0.247975
BBS5	0.161874	0.247955
FAM47E	-0.22614	0.247847

GDF6	0.308386	0.247831
STAG3L4	0.097085	0.247777
AC023115.1	-1.80541	0.247756
LINC02559	-1.22504	0.247749
LZTS3	0.09541	0.247706
TMEM216	-0.10634	0.247697
NEMP1	0.070499	0.247666
ATP2A3	-0.30872	0.247647
ISL2	0.149466	0.247613
CTU1	0.135068	0.247523
FBXL17	-0.09181	0.247523
AC239802.2	-1.80775	0.247498
MYL12BP1	-1.80775	0.247498
CARMIL3	-0.16419	0.247471
ZNF530	-0.15245	0.247469
AP002812.1	0.941489	0.247465
IDI2-AS1	0.140582	0.247401
KCTD13	-0.1076	0.247384
WAC	0.067442	0.24736
TFPI2	-0.08067	0.247344
SCML1	-0.12077	0.247325
TRANK1	0.228656	0.247299
LINC01337	-0.97952	0.247273
C19orf24	0.092803	0.247225
ATAD5	-0.11098	0.247136
PAIP2B	-0.17516	0.247136
TREX1	0.135121	0.247117
FRMD3	-0.35556	0.247111
PXDC1	0.146778	0.24708
PQLC3	-0.18257	0.247035
NA	0.898437	0.246995
AC138956.2	-0.56535	0.246973
SNAPC1	0.113376	0.246971
LINC02202	1.248296	0.246963
AP003068.1	-0.11822	0.246959
AL359853.2	-0.80087	0.246934
AP000345.2	-0.55014	0.246887
CKB	0.058481	0.246829
AP002812.5	-0.19055	0.246778
ALOX12	-0.29006	0.246746
FAM83A	1.920987	0.246732
TPPP2	1.920987	0.246732
MIR499A	1.920987	0.246732
UBA52P6	1.920987	0.246732
AL031283.3	1.920987	0.246732
MRPS36P1	1.920987	0.246732
CTSLP1	1.920987	0.246732
LINC00901	1.920987	0.246732
TUB-AS1	1.920987	0.246732
NA	1.920987	0.246732

CYCSP38	1.920987	0.246732
MIR548S	1.920987	0.246732
BNIP3P38	1.920987	0.246732
APLNR	-0.44021	0.246692
STK17B	0.127731	0.246667
ARMCX3-AS1	0.166535	0.24664
NA	-1.44624	0.246623
ABI3	-1.15747	0.246609
SEMA6B	0.541388	0.246597
KPTN	-0.11339	0.246594
IGSF11	0.676418	0.246583
MIR5692A1	1.80499	0.246561
FLI1	-0.26264	0.246558
NCDN	-0.07406	0.246474
CDC73	0.07089	0.246465
DCX	-0.09895	0.246414
SRSF12	0.106416	0.246412
COLEC10	1.52445	0.246409
AC021443.1	1.52445	0.246409
ABHD15	-0.19754	0.246377
AL591368.1	0.892177	0.246348
AL121987.2	0.102632	0.246345
COL6A6	-0.3018	0.246298
SUSD4	0.500064	0.24627
AL157896.1	-1.81004	0.246212
RPS27AP10	-1.81004	0.246212
ATP5IF1	-0.08106	0.246204
MARCH9	0.092244	0.246199
MBIP	0.12223	0.246171
AC129510.2	-1.50969	0.24611
FNIP1	0.085568	0.24611
IFI27L1	0.138221	0.246093
ATOH8	0.145226	0.246077
HNRNPR	0.061022	0.246033
RBSN	-0.07654	0.245972
POLR1C	0.073269	0.245916
UTP14C	-0.08297	0.245871
TCEA3	-0.45549	0.245867
DQX1	0.087833	0.245865
CPVL	-0.13186	0.245855
CFAP58-DT	-0.88184	0.245819
CYB5R4	0.085266	0.245734
AC008747.1	0.646358	0.245699
AL031432.3	-0.62454	0.245689
AC005586.1	0.119547	0.245683
RABAC1	0.077337	0.245666
DAP3P2	0.943026	0.245628
SMIM14	-0.09598	0.245585
SMAD4	-0.06771	0.245582
AC015914.1	1.019932	0.245566

INIP	-0.0868	0.245544
AL031728.1	-1.19229	0.245504
POT1	0.085075	0.245503
TBRG1	-0.09024	0.245479
CNPY3	-0.0681	0.245408
CHERP	-0.08486	0.245386
AC068385.1	-0.12499	0.245343
AC022210.1	1.304827	0.245303
DR1	0.059249	0.245287
AL590729.1	-0.81575	0.245262
SEC24A	-0.08393	0.245228
NA	0.720908	0.245223
CDC45	-0.11068	0.245217
AL132780.2	0.215604	0.245159
AC087521.3	0.675001	0.245157
RPS3AP54	-1.80527	0.245129
AC063938.1	-1.80527	0.245129
PQLC2	0.119021	0.245115
ZNF69	0.21167	0.245098
NA	0.412403	0.245083
CIRBP	0.057325	0.245066
HHEX	-0.21836	0.245057
SPG7	0.068773	0.245029
AC009948.2	0.301026	0.245021
AC046185.2	0.507388	0.24501
POLQ	-0.09158	0.24485
AC008494.1	-0.85228	0.244843
DNAAF2	0.083912	0.244799
INSIG1	0.242292	0.244793
SLC4A9	1.524537	0.244785
CYBC1	-0.07508	0.24477
ZDHHC8	-0.09031	0.244769
VPS4A	-0.07703	0.24474
HSPE1	0.098207	0.244731
ROS1	-0.63355	0.244729
AC092295.2	0.323365	0.244715
AC105053.1	0.688749	0.244677
GPR84	1.282058	0.244658
SNHG18	1.282058	0.244658
SPEG	0.086962	0.244657
VPS35L	0.072143	0.244644
PRDX2P3	1.085983	0.244594
AL157871.4	-0.12528	0.244587
EMC7	0.07464	0.244544
RFTN1	-0.11825	0.244503
MLEC	0.064779	0.244476
PDZD11	-0.09231	0.244439
STARD10	0.132885	0.244382
SLC1A1	0.317654	0.244355
AC233280.1	-0.72235	0.24434

AC009299.2	0.900639	0.244313
ACIN1	-0.05912	0.244278
VN1R107P	1.084119	0.244242
ALPL	-0.27514	0.244215
MPL	1.920948	0.244208
TAS2R9	1.920948	0.244208
PNOC	1.920948	0.244208
KCNS3	1.920948	0.244208
RF00019	1.920948	0.244208
ANAPC10P1	1.920948	0.244208
LINC02158	1.920948	0.244208
AL162393.1	1.920948	0.244208
AC009970.1	1.920948	0.244208
RN7SL40P	1.920948	0.244208
AC107032.1	1.920948	0.244208
AC084783.1	1.920948	0.244208
AC020917.2	1.920948	0.244208
AC119396.2	1.920948	0.244208
NR1H3	-0.17453	0.244195
NA	0.480593	0.244176
AC090589.2	1.582867	0.244167
RNU6-94P	1.582867	0.244167
ASB3	0.276976	0.244144
DEUP1	1.920945	0.244052
AL356776.1	1.920945	0.244052
KRT8P8	1.920945	0.244052
SCARNA22	1.920945	0.244052
AC037450.1	1.920945	0.244052
AC022306.1	1.920945	0.244052
AC090518.1	1.920945	0.244052
AC010266.2	1.920945	0.244052
SH3BP2	-0.08726	0.244039
IP6K3	-0.23683	0.244035
AC064850.1	0.64568	0.244032
RTBDN	-0.21854	0.244007
CCAR1	-0.06511	0.243971
AC024060.1	-0.16237	0.243922
MYEOV	0.772906	0.243921
AC093390.1	1.804653	0.243895
RPS24P17	1.804653	0.243895
IQCN	-0.16455	0.243868
ZNF319	0.17301	0.243849
HSF4	0.11785	0.243828
LINC01829	0.978046	0.243828
AL118511.1	-0.58132	0.243784
AMACR	0.309504	0.243754
AC134407.1	1.804632	0.243731
GRM8	0.288476	0.243728
AL133410.1	0.349372	0.243715
BMPR1B	-0.15111	0.243697

AL590369.1	-0.17133	0.243608
STPG3	-0.50528	0.243598
NA	0.419848	0.243583
AC091153.2	0.562255	0.243542
CST7	1.516654	0.24348
HEPACAM	1.516654	0.24348
AL080276.2	0.386464	0.243396
ATXN2-AS	0.661472	0.243355
CADM2	-0.11735	0.243333
LINC01518	-0.23967	0.243331
AL449212.1	-0.22046	0.243327
MFSD8	0.116632	0.243326
MAP1S	-0.10759	0.243319
FAM180A	-0.69866	0.243318
KCNC3	-0.12347	0.243188
AC122108.1	-0.53772	0.243173
NRDE2	-0.09	0.243106
AL365217.1	0.878824	0.243095
AP3M2	0.083872	0.243088
SRF	0.102138	0.24306
LSAMP	0.061475	0.24304
UPP2	0.745283	0.24302
THRAP3	-0.05462	0.242987
AP000527.1	0.785605	0.242978
OMA1	-0.18381	0.242934
AC023794.1	0.296873	0.242921
DDX20	-0.08167	0.242894
ARHGAP10	0.108667	0.242869
SAMD9L	0.191772	0.242792
AL020996.1	-0.13701	0.242782
HDAC11	0.16338	0.242762
TSSK1A	-1.09012	0.242746
ITIH2	-0.94956	0.242725
HDAC7	0.098944	0.242724
AL139147.1	-0.32998	0.2427
FAM168B	-0.05816	0.242683
FANCI	-0.08174	0.242676
AC011625.1	-1.12958	0.242656
UBE2G1	-0.07009	0.242641
HAPLN3	0.264758	0.242488
TRA2A	0.075403	0.242481
KCTD19	0.302926	0.242437
AC093162.2	0.677008	0.242432
OXNAD1	0.117631	0.242401
ATG4B	0.072425	0.242373
NA	0.359557	0.242332
ACTBP2	-0.96867	0.242319
AC002310.1	0.171537	0.24231
MCEE	-0.18317	0.242261
BSPRY	1.02845	0.242254

SCOC-AS1	-0.30889	0.242231
NA	0.158712	0.2422
AC092171.1	1.291853	0.242192
NIT2	0.090061	0.242152
ECHDC3	-0.23207	0.242112
CACNG4	-0.08673	0.241996
LSM5	0.082257	0.241988
RNF2	-0.07772	0.241962
AC087289.2	-0.16892	0.241958
NA	-1.12607	0.241958
SNHG31	0.522202	0.24195
DUSP22	0.112844	0.241893
MYO6	0.083009	0.24187
NA	1.007976	0.241869
ZNF493	0.134089	0.241824
SLC7A10	-0.35381	0.241782
BX324167.1	1.006043	0.241762
TAS2R14	0.294	0.241712
MAFG-DT	-0.13961	0.241694
FAM96AP2	-0.86612	0.24168
TNNI2	-0.94666	0.241677
LINC00319	0.813721	0.241675
MEAK7	-0.11873	0.241649
DMTN	0.145721	0.241648
TPO	-1.25541	0.241589
MIR1260B	0.742696	0.24157
PSMB10	-0.27452	0.241569
AC023509.3	-0.33004	0.241499
MEMO1	-0.14756	0.241429
SUSD3	-1.63713	0.241414
HNRNPA1P5	-1.63713	0.241414
AC053503.5	0.561392	0.241372
NAA10	-0.08088	0.241369
SPATA2L	-0.12635	0.241339
ZFYVE27	0.083249	0.241338
FAM53A	0.187452	0.241304
SERF1B	0.309126	0.241268
ACTR1B	0.077459	0.241236
C11orf58	0.076226	0.241199
ZMYND8	0.072737	0.241118
AC019097.1	0.525114	0.241086
TMEM147-AS1	0.07935	0.241082
DRAXIN	0.31282	0.24106
SPART	-0.10044	0.240968
ZCWPW1	-0.13879	0.240942
ZBTB6	-0.07493	0.240824
PDE6B	0.146318	0.240816
CHCHD2P9	0.668577	0.240811
KRI1	-0.08434	0.240765
SMIM15	0.068247	0.240732

FAUP1	1.192725	0.240656
TRUB2	-0.07145	0.240633
LDHAL6A	0.262142	0.2406
AC211476.2	0.520347	0.2406
AL592078.1	-0.11673	0.240581
AC016737.1	-0.59929	0.240529
PABPC1	-0.05313	0.240485
CXCL10	-0.2091	0.240467
AC037459.2	0.065121	0.240466
PTOV1-AS2	0.078858	0.240444
SNORA38B	-0.9779	0.240418
CD34	-0.69569	0.240413
NUP37	0.104651	0.240383
ACOT2	0.091016	0.24035
SMAD9-IT1	0.235685	0.240339
BOLL	1.427918	0.240338
UBE2MP1	1.427918	0.240338
KCTD12	-0.07064	0.240304
NA	0.893701	0.240295
NA	0.779394	0.240153
VAT1	0.071295	0.240133
PPP1R14B	0.054713	0.24012
AC078899.2	-0.98917	0.240113
AC092287.1	1.241048	0.240106
ATP5F1A	0.055939	0.240043
CABCOCO1	0.283453	0.239976
TNFRSF13C	-1.03624	0.239916
DLC1	0.124439	0.239912
EDARADD	-0.72604	0.239905
ARMCX2	-0.07194	0.239863
PDZD4	-0.06915	0.239845
NA	0.253832	0.239781
DDA1	0.061085	0.239778
RN7SL800P	1.117759	0.23977
AC005034.3	-0.1272	0.239767
PLEKHG4	-0.10773	0.239666
GJA1	-0.36234	0.239627
SNAI2	-0.09069	0.239548
HPS1	-0.09229	0.239468
FGF13	0.617851	0.239467
CCND3	-0.08436	0.239466
AL135786.2	1.235398	0.239448
CEP95	0.072429	0.239445
APCDD1L	-1.01794	0.239445
MPRIP	-0.06583	0.239437
AC093158.1	1.11756	0.23935
COX6B1P5	1.11756	0.23935
AL356056.2	0.504542	0.239333
DTNBP1	-0.12651	0.239318
PPM1K	0.090142	0.239308

DDR1	0.105925	0.239297
CAPSL	-1.48954	0.239287
AC104115.1	-1.48954	0.239287
NA	-1.48954	0.239287
LLGL2	0.19149	0.239253
CBWD5	0.169055	0.23922
CYB561D1	-0.1094	0.239141
WDSUB1	-0.14449	0.239129
EEF1D	0.059155	0.239008
ELK1	0.076653	0.238976
TRIL	0.553048	0.238867
FBL	0.06171	0.238861
AP001107.6	-0.25694	0.238845
PTPRG	0.101467	0.238789
AKR1B1	0.055668	0.238758
RPS2P32	0.154921	0.238757
KAT14	-0.09489	0.23875
PCDHB11	-0.60002	0.238713
ATP11C	0.065539	0.238614
GIN1	-0.16141	0.238611
NLRP9	-0.86888	0.23855
AC004584.3	-0.74759	0.238533
WWP2	-0.09702	0.238475
SESTD1	0.081622	0.238456
AC008764.5	-0.85898	0.238436
PKNOX2	-0.75135	0.238402
POLK	-0.0909	0.238373
VEGFB	0.095761	0.23837
AC091564.2	-0.16637	0.238349
GOPC	0.06886	0.238333
NA	0.295036	0.238297
RPL12P4	0.26269	0.238254
DUSP5	0.204315	0.238232
GLIS2	0.129634	0.238227
LAMTOR4	0.06328	0.238191
PPIL2	-0.07061	0.238189
UBTF	-0.05782	0.238139
MLH1	-0.07204	0.238121
CLYBL	0.186195	0.238119
AC025423.1	0.862788	0.238116
MFNG	-0.84944	0.238114
AC008808.2	0.24337	0.238093
PRKRA-AS1	-0.08158	0.238086
BABAM2	0.085104	0.238082
FEM1A	-0.06486	0.238081
EXD1	0.972232	0.23807
MAPK1	-0.07381	0.238056
SNORD108	-0.39114	0.238019
RPL7P32	1.312636	0.238016
HMGB1P21	1.312636	0.238016

IKBKB	0.095944	0.238015
DUOX1	-0.13507	0.237993
Z84485.1	-0.6037	0.237985
THEM6	-0.08275	0.237969
AC105389.3	0.099597	0.237913
CREG2	-1.31415	0.237894
PPME1	-0.06123	0.237878
ANXA5	-0.34431	0.237784
AC066613.1	-0.16169	0.237728
AC107214.2	-0.5154	0.237723
GTF2IP20	-0.11665	0.23766
NT5DC4	-0.18272	0.237569
AL358074.1	0.607404	0.237559
FIBCD1	1.595556	0.237537
AC091057.1	-0.09912	0.237471
ZNF705E	-0.59842	0.237468
BX088651.4	-0.34452	0.237464
MARK2P9	1.309122	0.23736
AC018362.1	-0.10014	0.237358
ODAPH	0.566349	0.237345
LANCL3	0.670265	0.237334
SNAPC3	-0.06234	0.237325
IL15	0.191059	0.23732
AC112184.1	-0.37184	0.237319
LNX2	-0.11975	0.237319
VAMP8	0.775034	0.237303
ITGA9	-0.08721	0.237284
REEP5	-0.08227	0.237251
RPH3A	-0.07076	0.237246
PHPT1	0.085396	0.237244
DDX23	0.064864	0.237157
PHACTR3	-0.20516	0.237144
MAPRE3	-0.10813	0.237137
COX11	-0.06972	0.237093
FAM53B	0.098031	0.23709
PCDH18	0.101451	0.237089
QRFP	1.325382	0.237068
AC020558.1	-0.17466	0.237053
AC008105.3	0.650829	0.237047
CKAP5	-0.06249	0.237013
EPHA1	0.128804	0.236996
TPBG	-0.09961	0.236914
TRIM63	0.428301	0.236907
ISG20L2	-0.06259	0.236838
OCIAD1-AS1	-0.21052	0.236834
MELTF	-0.11827	0.236817
A1BG-AS1	-0.12193	0.236806
SNX18P13	-1.22017	0.236741
SRP14-AS1	0.159457	0.236698
ZNF730	0.202995	0.236693

ANKRD37	-0.12344	0.236688
RSPRY1	-0.07753	0.236645
TBP	0.077176	0.236606
DHRSX	-0.10865	0.236601
NOD2	0.274356	0.23659
AL136126.1	-1.21602	0.236578
WDR5B	0.094067	0.236558
CLN3	-0.36359	0.236546
ZBTB41	0.07922	0.236533
STAM	-0.07863	0.236514
AC027290.1	-0.13013	0.236506
MOAP1	0.066181	0.236452
NDRG3	-0.07818	0.236442
NA	-0.3261	0.236414
MARCH5	-0.06936	0.236402
LIN28B	-0.10612	0.236383
MARK2P17	-1.25047	0.236383
HSF2	0.086405	0.236381
AC073343.2	0.483479	0.236346
GOLGA2P7	-0.10009	0.236317
RN7SL262P	1.587316	0.236271
AL359881.3	1.587316	0.236271
NAGPA	-0.16195	0.236249
SYT4	0.067556	0.236187
ATG7	0.064963	0.236176
FBXL20	0.126828	0.236099
MRPL20-AS1	-0.08384	0.236089
AC026347.1	-0.59429	0.236074
NA	1.072873	0.236062
AC016747.1	-0.12951	0.236059
MIR4258	-0.45892	0.236054
LINC00630	0.180382	0.236048
CFLAR	0.09964	0.236024
AC068620.2	-0.1545	0.235952
CLDN10	-0.27051	0.235903
AP003059.1	-1.39747	0.235897
CST3	-0.10353	0.235897
PTPA	0.059204	0.235844
NA	0.254135	0.235839
AC027807.1	1.01657	0.235831
AC022445.1	-0.23209	0.235703
ZNF544	-0.33391	0.235641
TAS2R5	0.275305	0.235601
INTS8	-0.09083	0.235534
CD320	-0.07735	0.2355
RTRAF	-0.07425	0.235485
RPS25	0.083032	0.235419
LRRN3	0.078556	0.235331
EPN1	-0.07332	0.235311
ESAM	-0.29844	0.235226

AL807752.3	-0.10504	0.235225
GNG10	-0.73631	0.235187
RPL22P12	-0.6647	0.235181
SHISA4	-0.11251	0.235181
SLC10A1	1.325261	0.235166
NA	1.325261	0.235166
RPL7P57	0.68534	0.23514
AC078785.1	0.178306	0.235135
KRIT1	-0.06431	0.235119
ELF4	0.152897	0.235025
AC012645.3	0.348103	0.234941
SNORD12C	0.682362	0.234921
LINC00957	0.285404	0.234913
NRL	-0.24121	0.234903
HS3ST2	0.88721	0.234854
PPTC7	-0.08064	0.234851
COPS5	0.074843	0.23482
STMP1	0.070869	0.234819
EDIL3	-0.34732	0.234688
MPC1	0.093363	0.234662
SLC30A7	0.07254	0.234575
NA	-0.46211	0.234544
DNMBP	0.122811	0.234538
CAPN10-DT	-0.14322	0.234531
AC010261.2	-0.26963	0.234527
MTMR8	0.38953	0.234508
RNA5SP18	0.968053	0.234493
MRPS22	0.076339	0.234492
CRYM-AS1	0.563119	0.234453
CAPS2	0.153876	0.234445
NA	0.970403	0.234412
AC012615.5	-0.13621	0.23438
NA	-0.26479	0.23434
RFX3-AS1	-0.35765	0.234268
HAUS6	-0.07521	0.234256
POMP	0.068675	0.23422
AC010201.2	-0.23821	0.234136
ARMC2	-0.22335	0.234118
CORO7	-0.16959	0.233965
ARID4B	-0.06354	0.233911
IRS1	-0.16261	0.233907
ZIM2-AS1	-0.90632	0.233902
SPDYE21P	-0.47841	0.233792
MCL1	-0.0516	0.233756
SNORA31	0.770576	0.233743
TMX4	-0.0718	0.233739
CTH	-0.18064	0.233731
PPT2-EGFL8	-0.07245	0.233722
DCTN3	-0.08313	0.233697
LMNA	0.066898	0.233646

RBAK	-0.07479	0.233643
FGD6	0.11085	0.233638
AC008610.1	-0.48339	0.233637
AC242426.2	-0.26005	0.233634
GPD1L	-0.08448	0.233593
HS2ST1	0.075581	0.233585
RF00019	1.493099	0.23353
NA	1.493099	0.23353
AC246785.3	1.493099	0.23353
CSPG4P13	1.493099	0.23353
SSTR2	-0.0961	0.23348
SNHG3	0.071366	0.233478
MFAP3	-0.0645	0.233464
TMA16	0.087454	0.233436
NA	0.448797	0.233425
TERF2	0.075399	0.233411
EEF1A1P5	0.089657	0.2334
C1GALT1	-0.09985	0.233387
ZRANB1	-0.06929	0.233369
RPL21P10	-0.95414	0.233365
MAP9	-0.0848	0.233348
AC009119.3	1.207049	0.233314
PRRX2	0.138574	0.233283
FLT3LG	0.601217	0.233248
UBE2F	0.101946	0.233244
SLC35G6	-0.64604	0.233184
SLC11A2	0.06801	0.233176
CRAMP1	-0.11857	0.233165
LBX2-AS1	0.2388	0.233128
MIR3648-2	0.490666	0.233096
RUVBL1-AS1	1.191033	0.233074
NA	-1.18318	0.233073
PCGF5	0.091206	0.233047
PPP1R3G	1.066585	0.23304
GOLGA8VP	-0.65142	0.232949
WDCP	-0.07726	0.232945
TLCD1	0.203264	0.232904
HIRA	-0.11396	0.232869
ZIC4	-0.14244	0.232824
MED12	-0.07621	0.23282
OXCT1-AS1	0.242997	0.232806
CYSLTR2	-0.45845	0.232798
HCFC1-AS1	-1.49554	0.232791
AC025752.1	-1.49554	0.232791
FAM90A25P	-1.49554	0.232791
CAP2P1	-1.49554	0.232791
AC092127.1	-1.49554	0.232791
NRG1-IT1	1.231388	0.23276
PRKRIP1	0.072501	0.232743
TET1	-0.10824	0.232743

VARS	-0.07345	0.232709
LSM7	0.064558	0.232704
NA	-0.05421	0.232683
MEGF10	-0.45505	0.232676
AMH	-0.08925	0.232635
AL451070.1	0.132169	0.232613
AC105389.1	0.631355	0.232602
POLR2E	-0.0624	0.232602
FILIP1L	0.07897	0.232599
ARRDC3	0.069373	0.232575
COX7A1	-0.70297	0.232539
KCNMB4	0.136997	0.232404
MCAT	-0.15947	0.232353
GMDS	-0.13213	0.232352
AC006557.3	-0.38478	0.232317
NHLH1	-0.71275	0.232302
RN7SKP80	-0.98673	0.232264
NA	-0.24761	0.232226
TBC1D14	-0.06401	0.232178
C19orf44	-0.08344	0.232133
AC018638.5	-0.14775	0.232117
TMEM41A	-0.09947	0.232058
FBXW8	0.083323	0.231916
SH3BGRL3	-0.07121	0.23189
TGOLN2	-0.05688	0.231865
PPP1R21	0.111683	0.231855
C9orf163	0.290958	0.231824
KCNIP4	-0.1675	0.23179
TUNAR	-0.1869	0.231743
AP000355.1	-0.9333	0.23166
SMG6	-0.08288	0.231655
MIR218-2	0.33204	0.231615
CTR9	0.056551	0.231587
CCNI	-0.05441	0.231548
MAT2A	0.056632	0.231546
SGSM3	0.091726	0.231508
RGPD1	0.443511	0.231483
AC020911.1	0.468772	0.231455
AC004854.2	0.128144	0.231448
MYORG	-0.15806	0.231447
AC040174.2	1.45683	0.231386
AP005899.1	0.582661	0.231287
UBE2Q2L	0.517869	0.231256
TTLL7-IT1	1.141027	0.231204
HMX2	1.042169	0.23118
AL049840.4	0.159808	0.231159
C12orf76	0.084681	0.23115
CEP350	0.061835	0.231109
NA	0.602559	0.231094
NAT14	0.073659	0.231069

AURKAIP1	0.07065	0.230965
DYRK2	-0.06379	0.230909
EEF1A1P11	0.281637	0.230846
ALX4	-0.6357	0.230802
TRPV1	0.841212	0.23072
IRGM	-1.11896	0.230699
MGRN1	-0.08549	0.23069
RPL18AP2	-1.22918	0.230674
MAP3K2-DT	0.397221	0.230669
AC012640.4	0.069717	0.230662
LAMP1	-0.05969	0.230642
GNAS	-0.05058	0.230623
RASSF10	-1.22762	0.230583
PLCXD1	-0.08823	0.230545
AL355472.2	-0.38718	0.230542
AL359504.1	-1.13042	0.230528
TMC6	-0.14893	0.230513
NA	1.52271	0.230392
TBX1	-0.31651	0.230391
PPIH	0.085743	0.230388
TSPAN32	0.405734	0.23037
ATP11B	0.069278	0.230324
HMGA1	-0.05427	0.230313
LINC01060	-0.33174	0.230254
LINC02367	0.608945	0.230241
AC015909.4	-0.62632	0.23018
PHC1P1	0.179079	0.23014
FAM90A2P	1.009792	0.230103
XIRP1	1.086941	0.230075
CDC42EP3	0.059418	0.230039
DAXX	-0.05692	0.229999
AC139795.3	0.567192	0.229987
TMEM63A	-0.11679	0.229973
NUFIP1	0.111553	0.229927
NPM1P21	1.42273	0.229895
CFAP61	0.559536	0.229891
NPM1P13	0.456088	0.229797
AC130456.5	-0.15084	0.229781
NA	1.167913	0.229776
NA	0.545401	0.229772
POM121L9P	-0.36768	0.229736
PLEKHM3	-0.18109	0.229698
PRDX1	-0.06734	0.229683
SPDL1	0.065965	0.229657
CTSK	0.139477	0.229626
CDC25C	0.126689	0.229617
AC010761.2	0.06148	0.229611
AC008554.1	0.130778	0.229554
SVEP1	-0.21266	0.229549
AC011313.1	-1.09973	0.22954

RPL7L1	0.056225	0.229481
RPS4XP16	-0.47546	0.229434
BNIP3P27	-0.62925	0.22941
AC018868.1	-0.90305	0.229308
NAE1	0.067647	0.229252
TPRKB	-0.0913	0.229228
NA	0.222768	0.229219
NA	-1.16309	0.229175
SLC25A44	-0.05933	0.229164
AP000911.1	0.398999	0.229136
WDR6	-0.05969	0.229019
REEP2	0.095704	0.229011
NA	-0.97665	0.228976
SHISA3	0.415438	0.228968
FDX1	-0.12482	0.228963
NT5C3B	0.068542	0.228938
AC119674.1	-1.42733	0.228912
AC010525.1	-1.42733	0.228912
ETS1	-0.19568	0.228857
COQ9	0.0792	0.228839
ZFAND1	0.091971	0.228825
TRAPPC8	0.080194	0.228759
CDKN2AIP	0.090718	0.228727
CSAG4	0.347672	0.228688
PCNA	0.062408	0.228673
CDC42BPB	-0.06198	0.228578
BX088651.1	0.549735	0.228565
SLC16A14	-0.3821	0.228556
AHSA1	0.055669	0.228554
PTGR2	0.107033	0.228544
MAGOH	-0.07562	0.228489
GRPEL2-AS1	0.172042	0.228416
NA	-1.0415	0.228413
C2orf88	0.305275	0.228351
NAB1	0.071767	0.228342
SLC24A1	-0.10458	0.228269
AC112694.1	-0.42366	0.228213
CTSO	0.121132	0.228208
AC002563.1	-0.38496	0.22816
PRDM1	-1.81162	0.228141
HAS1	-1.81162	0.228141
ATP8B5P	-1.81162	0.228141
RNU6-824P	-1.81162	0.228141
RNU6-335P	-1.81162	0.228141
AC006019.1	-1.81162	0.228141
OR2W2P	-1.81162	0.228141
AC104333.2	-1.81162	0.228141
RNU6-277P	-1.81162	0.228141
AC104655.1	0.134843	0.228105
FANCG	0.075745	0.22808

KATNAL1	-0.08973	0.228075
CHIC2	0.114531	0.227855
RAB11A	0.069651	0.227777
PKN3	-0.10309	0.227776
JAM2	0.092542	0.227752
KCNAB1	-0.22234	0.227704
KIF9-AS1	-0.24605	0.227683
HSPD1P11	-0.5009	0.227662
AC005759.1	-0.21024	0.227634
CKS1BP7	-1.36407	0.227624
CELSR1	-0.29425	0.227612
SLC35G2	-0.10427	0.227553
GTSF1	-0.16042	0.227551
ARHGEF10L	0.083239	0.227518
RSRP1	0.064908	0.227512
FCRL6	-1.19019	0.227487
AC105277.1	-0.75085	0.227452
AC100786.1	-0.19476	0.227358
EAF1	-0.06821	0.227351
ZFPM2-AS1	0.351061	0.227329
ZNF543	-0.12919	0.227301
ATP2C1	0.055823	0.227267
AC024293.1	0.245253	0.227252
SDHAF3	0.107502	0.227211
STRADB	-0.08012	0.2272
ZSCAN12	-0.0902	0.227147
RNASEH1P1	-0.18528	0.227147
PC	0.075635	0.227106
AC139769.1	-0.50218	0.227095
TMEM60	-0.10724	0.227058
CD109	-0.50323	0.227053
TTC39A-AS1	-1.11014	0.227011
TP53	-0.06817	0.227004
PPM1F	-0.0654	0.226958
AL353719.1	-1.03398	0.226915
TRIM52	-0.08729	0.226832
AL365356.1	0.219425	0.226715
TSPOAP1	0.172999	0.226665
AKT1	-0.05196	0.226655
TFPI	-0.30715	0.226648
TM4SF20	-1.41766	0.226635
AL121652.1	-1.41766	0.226635
ARMT1	-0.10514	0.226625
NXT1	-0.09113	0.226605
MAP3K2	0.061984	0.226593
RANGRF	-0.0833	0.226587
USP15	0.078686	0.226549
AL031963.3	0.236387	0.226536
KF459542.1	0.152814	0.226528
AC211433.2	1.290101	0.226499

GEMIN7	-0.14742	0.226484
TMX3	-0.06324	0.226417
SOWAHD	1.100452	0.226401
KMT5B	-0.06613	0.2264
SMIM10L1	0.119164	0.226286
SLFNL1	-0.35171	0.226268
KCNJ9	0.308746	0.226257
CNTRL	-0.08252	0.226196
C2CD4B	0.622603	0.226183
SF3A3	-0.05476	0.226179
AC004160.2	0.19451	0.226162
RHEBP1	0.9303	0.226156
STRIP2	-0.1597	0.226145
ARMC10P1	-1.80191	0.226144
AC011742.2	-1.80191	0.226144
HSPA8P18	-1.80191	0.226144
AC017099.1	-1.80191	0.226144
IL20RB-AS1	-1.80191	0.226144
AC093367.1	-1.80191	0.226144
LINC01570	-1.80191	0.226144
NA	-1.80191	0.226144
GSKIP	0.084904	0.226111
AC133552.3	0.909852	0.22602
FAM111B	0.179111	0.225948
NPPA	0.282399	0.225921
NUP62	-0.06517	0.22592
NA	0.239226	0.22591
CYP51A1	0.173803	0.225846
AC013468.1	0.466002	0.225807
ALLC	-0.48226	0.225764
AC007663.3	1.160066	0.225732
ENAH	0.049271	0.225727
INTS14	0.068527	0.225715
ARIH2	0.058816	0.225698
SLC22A23	0.203428	0.225692
ZDHHC13	0.080677	0.225681
PEX11B	-0.0919	0.225662
MT-ND4	0.052379	0.225662
AC087163.2	-0.45438	0.22564
SEC62-AS1	-0.53333	0.225629
MLF1	0.121203	0.225598
C1orf147	-0.45225	0.225548
AC097468.1	0.502658	0.225545
CCND1	-0.06166	0.225516
NA	0.507112	0.22549
AC021148.1	-0.85353	0.225447
ECM2	0.77989	0.225414
PLAAT2	-1.34199	0.225399
AP002414.1	-1.34199	0.225399
YOD1	-0.09301	0.225363

SPOP	-0.06292	0.225341
VHLL	-1.24755	0.22534
PHIP	0.070272	0.225313
AC020978.5	-0.07706	0.225308
KIRREL3	-0.67509	0.225277
MLANA	-0.46399	0.22527
AC098820.1	0.504832	0.225243
OGA	0.063745	0.225237
AC118549.1	0.066985	0.225223
ARL2BP	-0.07126	0.225213
AC010834.1	-0.1326	0.225206
SSSCA1-AS1	-0.2998	0.22511
SNX11	-0.07376	0.225098
IL23A	-0.44803	0.225092
GLIPR1L1	-0.42561	0.225054
ADCY5	-0.23796	0.22502
AL021068.1	-0.99358	0.224972
EIF4BP7	-0.37064	0.224925
AC084125.2	-0.28275	0.224887
TSTD1	0.126603	0.224838
C1QTNF4	0.490219	0.224829
ZNF611	-0.14018	0.22471
USP48	0.062608	0.2247
MORN5	-1.81147	0.224663
AC092447.1	-1.81147	0.224663
RNU6-1043P	-1.81147	0.224663
AL022238.1	-1.81147	0.224663
AL592437.1	-1.81147	0.224663
AC119751.1	-1.81147	0.224663
ERP29P1	-1.81147	0.224663
MIR3150BHG	-1.81147	0.224663
RNU11-2P	-1.81147	0.224663
NA	-1.81147	0.224663
RPL23AP57	-1.19148	0.224634
RPF1	0.071122	0.224592
SIAE	-0.10153	0.22456
NOP2	0.059902	0.224556
C9orf43	0.119588	0.224535
TCF4	0.065378	0.22445
NA	-0.10629	0.224375
PINK1	0.073122	0.224267
LSM11	0.088608	0.224233
RPSAP72	1.102324	0.224225
BCL2L2-PABPN1	-0.46738	0.224178
MIR23A	1.003438	0.224137
GBA	-0.07317	0.224133
LUM	0.075929	0.2241
VSTM4	-0.18214	0.22409
CRYGN	1.094717	0.224015
PYROXD2	-0.20096	0.224007

AP2S1	0.061186	0.223931
NDUFB8P2	0.656283	0.223929
ARCN1	-0.08122	0.223917
SERINC3	0.058296	0.223906
MANBAL	-0.06806	0.223817
ATG101	0.074447	0.22378
AC005280.1	0.294298	0.223754
TFAM	0.064578	0.223754
TMEM99	-0.16015	0.223746
CCNL2	-0.05547	0.223696
SLC22A17	0.080253	0.223632
DENND6B	-0.17496	0.223598
NAGS	0.272016	0.223587
AP002336.2	-0.40704	0.223559
POR	0.08532	0.22355
CHRM3	0.158804	0.223501
COPS8	-0.06708	0.223457
PRR7-AS1	-0.18928	0.223432
NA	0.473896	0.223428
AC053527.1	0.397984	0.223422
GABARAPL3	1.39522	0.223413
SLMAP	-0.07471	0.223377
KIAA1755	-0.51658	0.223366
ENTPD6	-0.08587	0.223302
CSNK1A1P1	1.215498	0.223301
ZWILCH	0.091027	0.223226
IFT57	-0.07687	0.223216
ING1	0.082365	0.223175
NA	1.434412	0.22317
ARHGEF7-AS2	0.380316	0.223148
MAPK8IP1	-0.07511	0.223105
LIF-AS1	0.806054	0.22309
COX20	-0.06501	0.22305
WFDC3	-1.51087	0.223046
PDE3A	-0.41936	0.223027
AC020978.6	0.938433	0.222995
DEGS2	1.427202	0.222985
NA	0.676041	0.22297
SNRNP200	-0.05175	0.222953
PRAME	0.052463	0.222922
RNF139	-0.08184	0.222884
NA	-0.78041	0.222866
MORN4	0.100268	0.22285
AC008013.1	0.759892	0.222824
VCPIP1	-0.0789	0.222766
TMUB1	-0.07099	0.222758
FAM76A	0.130302	0.222736
AC090948.2	-0.631	0.222713
B4GALT3	-0.06148	0.222703
TTLL4	-0.08804	0.222677

PRICKLE2	-0.70791	0.222677
AQP8	-1.80174	0.222673
SHISAL2A	-1.80174	0.222673
RNU1-59P	-1.80174	0.222673
AC004840.1	-1.80174	0.222673
AL445183.2	-1.80174	0.222673
LINC01968	-1.80174	0.222673
NA	-1.80174	0.222673
AC093520.1	-1.80174	0.222673
AC092378.1	-1.80174	0.222673
NA	-1.80174	0.222673
DSG1-AS1	-1.80174	0.222673
AL357568.2	-1.80174	0.222673
EEF1E1P1	-1.16127	0.222671
AC007431.1	-1.16127	0.222671
CBX6	-0.08059	0.222668
PTCD3	0.06471	0.222662
AC007362.1	-0.26411	0.222651
NT5DC3	0.141489	0.22265
POLI	0.080416	0.222626
SBF1	-0.0748	0.222614
CCDC14	-0.08005	0.222579
CNBD2	0.459412	0.222557
AC078880.1	-0.48855	0.222554
AL450311.1	-0.89588	0.222512
RSF1	0.066475	0.222457
MAP3K14	-0.15863	0.222426
UBE2L6	-0.07867	0.222407
DPH1	0.091405	0.222362
NA	-0.05953	0.222358
MYBPC3	0.624442	0.22235
AC104653.1	1.265554	0.222344
RN7SL851P	1.265554	0.222344
LRRC37A7P	1.265554	0.222344
KIF20A	-0.07552	0.22234
AL136987.1	-1.34456	0.222333
ABHD8	0.100817	0.222296
MEGF9	-0.13261	0.222293
DCAF11	-0.0722	0.222274
NTPCR	-0.06956	0.222265
SLC16A1-AS1	0.28153	0.222265
IFITM10	-0.21199	0.222264
PRMT1	0.056804	0.222233
PDZD3	-1.81137	0.222214
C10orf62	-1.81137	0.222214
MIR181A2	-1.81137	0.222214
AC099794.1	-1.81137	0.222214
CCND2P1	-1.81137	0.222214
AL049833.2	-1.81137	0.222214
PLEKHO2	-0.11688	0.222135

DYNC1LI1	-0.06513	0.222124
TEP1	-0.09265	0.222123
SALL4	-0.24758	0.222122
PDCL3P4	-0.484	0.222104
MAMSTR	0.220321	0.222095
E2F1	-0.08861	0.222058
PDXK	0.075797	0.222017
GLMP	0.065678	0.222009
URM1	0.061911	0.221973
AC007608.1	-0.58981	0.221862
AC018755.3	0.259423	0.22182
CDKN2AIPNL	0.069048	0.221772
AL845552.2	0.122198	0.221763
RARS	-0.06401	0.221761
AC112220.2	0.417088	0.221747
AP5M1	0.075615	0.22172
LRRC46	0.209388	0.221703
AC021205.3	-0.55226	0.221683
AC095055.1	-0.58366	0.221643
RGS17	-0.11745	0.22163
NA	-0.15517	0.221612
POLR3F	0.083403	0.221592
CBARP	0.105272	0.221565
MME	-0.58168	0.221537
U73169.1	-0.75303	0.221475
FLG	0.925641	0.221453
MACO1	0.066808	0.221451
GMFG	0.859261	0.221413
HSBP1	0.066467	0.221411
AC133528.1	0.385884	0.221385
PSMB4	0.055028	0.221364
NUBP1	-0.08197	0.221319
TYW1	-0.06765	0.221315
AC079921.1	0.098162	0.221312
AC008736.1	0.803137	0.221296
CACNA2D3	0.82326	0.221292
LGALS1	-0.06387	0.22124
INTS6-AS1	-0.22191	0.22123
SLC25A46	0.065807	0.221227
AL049749.1	1.268407	0.221152
CIB1	-0.09006	0.221145
HMGXB4	-0.06939	0.221144
AP003559.1	0.827401	0.221132
AC096537.1	-0.292	0.221124
AC026355.1	1.336232	0.221108
AC010336.1	0.741449	0.221106
FAM135B	-0.16535	0.221102
C1orf226	0.510909	0.221084
RF00019	-0.84436	0.221082
KLF13	-0.07081	0.22107

NCEH1	-0.12627	0.221049
AC012313.5	-0.15313	0.221002
SLC35E2A	-0.19598	0.221001
AC111170.1	-0.80091	0.220985
DNAJB1	-0.05659	0.220985
MT-ND6	-0.06534	0.220971
C9orf85	0.105202	0.220911
SHMT1	0.103835	0.22091
XRCC2	-0.1018	0.220896
AC012499.1	-1.5011	0.220872
PTP4A2P1	-1.5011	0.220872
NA	-0.201	0.220828
NA	0.884101	0.220801
AC026150.1	-1.27906	0.220797
APOOL	0.135648	0.220786
STPG2	0.940568	0.220707
RNU7-40P	0.940568	0.220707
DUSP15	-0.22069	0.220697
ELP4	-0.16464	0.220686
B2M	0.056347	0.220681
LAMA4	-0.33187	0.220663
SPATA18	0.152605	0.220586
LINC00173	0.706082	0.220575
FHIT	0.222714	0.220566
AC010595.1	0.347666	0.220484
WASIR1	1.179885	0.220481
AL008721.1	1.179885	0.220481
GPR137	-0.07154	0.220435
AC096733.2	0.323745	0.220404
AL589182.1	-0.68371	0.220381
SMG1	-0.06028	0.220381
ELK4	-0.0682	0.220372
CFAP36	0.077743	0.220359
TBC1D19	0.138759	0.220298
HSPA4	-0.05695	0.22028
THPO	-1.80162	0.220227
TBX15	-1.80162	0.220227
COL8A1	-1.80162	0.220227
CELP	-1.80162	0.220227
CCR4	-1.80162	0.220227
MYL4	-1.80162	0.220227
NA	-1.80162	0.220227
DDX18P3	-1.80162	0.220227
NA	-1.80162	0.220227
RPL15P14	-1.80162	0.220227
AL449283.1	-1.80162	0.220227
GDI2P2	-1.80162	0.220227
AL359313.1	-1.80162	0.220227
NA	-1.80162	0.220227
RN7SL12P	-1.80162	0.220227

TEX35	-1.80162	0.220227
CPNE8-AS1	-1.80162	0.220227
AC022819.1	-1.80162	0.220227
NA	-1.80162	0.220227
AP002387.2	0.192959	0.220222
CYP7B1	0.163824	0.220199
VILL	-0.18781	0.220197
IST1	0.052237	0.220194
RNF43	-0.60319	0.220172
SATB1-AS1	0.090337	0.220167
ADIPOR1	-0.05406	0.220134
ITFG2	0.072469	0.220127
COPZ1	0.05927	0.220077
PIF1	0.126865	0.220064
RPS18P12	-0.59449	0.220045
MTFR2	-0.15745	0.22
CHD1	0.067603	0.21999
NA	-0.47822	0.219968
RABEP1	0.06265	0.219967
AC002094.2	-0.09877	0.219942
DNASE1L2	-0.18212	0.219893
CALB2	-1.60533	0.219838
CIDECP1	0.147064	0.219801
HOMER2	-0.09772	0.219714
AC012073.1	-0.15655	0.219695
SH3GL1P1	-0.2948	0.219608
USP19	-0.06522	0.219588
NA	0.533586	0.219544
GPRC5C	0.457872	0.219524
MPV17L2	0.102211	0.219513
ZBTB34	-0.07295	0.219512
PAK3	-0.09491	0.219448
NA	0.7076	0.219441
MTCO2P12	0.280946	0.219377
ZNF211	-0.09761	0.219335
LRRC57	0.111409	0.219264
TCTN2	-0.12669	0.219227
SORBS3	-0.09922	0.219225
ATRX	-0.06287	0.219222
PCBD1	0.084187	0.219215
ST7-AS1	0.158937	0.219215
AC020913.3	-0.80876	0.219203
SOS2	0.065825	0.2192
BBOX1	0.971413	0.219174
CUTA	0.055063	0.219165
CD302	-0.10891	0.219162
TMEM50A	-0.06714	0.219082
MSH5	-0.19177	0.219056
NUTM2D	-0.26755	0.218998
LDAH	0.070654	0.218949

ZNF667	0.09895	0.21892
RPL5P5	1.249154	0.218884
AL512844.1	-1.21486	0.218868
SNORD60	-0.4286	0.218849
CLEC18B	0.419434	0.218821
HRNR	-1.53439	0.218801
AK3P5	-1.53439	0.218801
AL023806.2	-1.53439	0.218801
AC005020.1	0.757126	0.218794
AC012368.1	0.341401	0.218788
CLUAP1	0.105498	0.218786
RALGAPB	-0.0564	0.218778
LYPLAL1	0.13151	0.218761
ROPN1L	1.088337	0.218745
ALYREF	0.051607	0.218739
NA	0.814774	0.218736
NA	0.694746	0.218711
AC010280.1	0.750816	0.218651
TMEM202-AS1	-0.1081	0.218591
DAAM1	-0.08173	0.21858
NA	0.195359	0.218572
ZDHHC24	0.105984	0.218548
AC004951.3	-0.64159	0.218508
MISP3	-0.23216	0.218498
BCDIN3D-AS1	0.122662	0.218383
CRYZP1	0.327879	0.218378
ABCB10	-0.08439	0.218331
AL161729.4	-0.2929	0.218318
FAM217A	-0.44724	0.218187
ERBB3	-0.67843	0.218158
ENPP2	0.770265	0.218128
ANKRD7	-0.31967	0.218124
MOB1B	-0.07307	0.218122
PLSCR4	2.07972	0.21812
RHOJ	2.07972	0.21812
BCL2L10	2.07972	0.21812
KCNH5	2.07972	0.21812
LMOD3	2.07972	0.21812
PCSK9	2.07972	0.21812
GYPA	2.07972	0.21812
TGIF2LY	2.07972	0.21812
CYP4F12	2.07972	0.21812
PABPC1L2A	2.07972	0.21812
AQP7P1	2.07972	0.21812
CYP2A7P2	2.07972	0.21812
RNU4-36P	2.07972	0.21812
RF00019	2.07972	0.21812
AL353740.1	2.07972	0.21812
NA	2.07972	0.21812
RF00019	2.07972	0.21812

MIR627	2.07972	0.21812
IGHD3-9	2.07972	0.21812
BTF3L4P3	2.07972	0.21812
CAPNS1P1	2.07972	0.21812
NA	2.07972	0.21812
KRT18P22	2.07972	0.21812
AL031133.1	2.07972	0.21812
NA	2.07972	0.21812
RF00019	2.07972	0.21812
WWC3-AS1	2.07972	0.21812
UBE2D3P2	2.07972	0.21812
AC002472.1	2.07972	0.21812
STARD13-IT1	2.07972	0.21812
VDAC1P6	2.07972	0.21812
AC006028.1	2.07972	0.21812
MTCO1P27	2.07972	0.21812
AC012065.2	2.07972	0.21812
AC114737.2	2.07972	0.21812
AL159169.1	2.07972	0.21812
AL450309.1	2.07972	0.21812
NA	2.07972	0.21812
RF00019	2.07972	0.21812
OR1J4	2.07972	0.21812
AC125618.1	2.07972	0.21812
RN7SL34P	2.07972	0.21812
AL627309.2	2.07972	0.21812
NA	2.07972	0.21812
RN7SL284P	2.07972	0.21812
RN7SL684P	2.07972	0.21812
AC010395.1	2.07972	0.21812
AC093827.2	2.07972	0.21812
RNU7-79P	2.07972	0.21812
RF00322	2.07972	0.21812
RN7SKP12	2.07972	0.21812
RF00424	2.07972	0.21812
RF00212	2.07972	0.21812
NA	2.07972	0.21812
RNU6-1098P	2.07972	0.21812
NA	2.07972	0.21812
RNU6-1028P	2.07972	0.21812
NA	2.07972	0.21812
LINC00346	2.07972	0.21812
ITFG2-AS1	2.07972	0.21812
GCSHP4	2.07972	0.21812
NDUFA5P6	2.07972	0.21812
CACNA1C-IT1	2.07972	0.21812
OR7E47P	2.07972	0.21812
AC068993.1	2.07972	0.21812
AC083805.2	2.07972	0.21812
AC073569.2	2.07972	0.21812

SRMP2	2.07972	0.21812
AL583722.4	2.07972	0.21812
AC025580.1	2.07972	0.21812
AC020661.2	2.07972	0.21812
AL353708.2	2.07972	0.21812
MIR5590	2.07972	0.21812
MIR3193	2.07972	0.21812
NA	2.07972	0.21812
MIR3944	2.07972	0.21812
AC015910.1	2.07972	0.21812
NA	2.07972	0.21812
MIR4326	2.07972	0.21812
NA	2.07972	0.21812
AC129492.5	2.07972	0.21812
AC016493.1	2.07972	0.21812
NA	2.07972	0.21812
AL162730.1	2.07972	0.21812
DUXAP4	2.07972	0.21812
RPL23AP92	2.07972	0.21812
AL109918.3	2.07972	0.21812
NA	2.07972	0.21812
AC016405.2	2.07972	0.21812
AC016575.1	2.07972	0.21812
KIAA0319	0.490428	0.218077
VPS52	-0.06178	0.218067
OAZ3	0.074795	0.218066
GRIN2C	0.26822	0.218058
AC005954.3	-0.26351	0.218045
CCPG1	0.122178	0.218036
MRPL9	0.060472	0.218023
CCIN	0.810333	0.21794
SAE1	-0.056	0.217923
GPC2	0.100151	0.217922
SMIM1	0.37681	0.217866
ARFGEF1	-0.06273	0.217841
APPL1	0.064665	0.217828
GINS2	0.092655	0.21782
MAFF	0.13273	0.217818
CFAP70	0.262574	0.217791
TOR3A	-0.07443	0.217733
NTM	-0.45328	0.217632
RO60	0.061824	0.217605
MAPKAPK5	0.079875	0.217554
SFT2D2	0.053805	0.217554
ALDH3B1	-0.21685	0.217549
RAP2B	-0.11303	0.217531
PCDHB1	1.161835	0.217456
CHN1	0.073104	0.217448
TMEM164	0.080615	0.21744
TAF3	-0.09297	0.217422

ZNF197	-0.07177	0.217419
FST	-0.47127	0.217411
ZNF449	0.127898	0.217355
NUP93	0.062501	0.217339
CCDC191	-0.13275	0.217313
AC011495.3	0.555012	0.217312
UGGT1	-0.05255	0.217243
TMEM44	-0.09809	0.217162
IRF2BP1	0.075101	0.217159
GPC6-AS1	-0.94557	0.21713
CDHR2	-0.12562	0.21711
CYP8B1	-1.02388	0.217049
COX14	-0.09094	0.216956
MAP3K15	0.138486	0.216956
NR3C1	0.09241	0.216955
PGK1	0.070942	0.216937
AC004034.1	-0.84326	0.216921
EXT2	-0.06342	0.216918
AL356488.3	0.222271	0.216897
GALE	-0.0751	0.216875
GPR137C	-0.09999	0.216848
AC055855.2	1.090009	0.216807
GAPDHP40	1.460321	0.216804
LIME1	0.143474	0.216803
AC108863.1	0.212562	0.216793
MIR186	0.39164	0.216733
AC008429.3	-0.63842	0.216694
ATAD2	-0.09037	0.216692
AC015795.1	0.321071	0.21668
FSIP2-AS1	0.841043	0.216675
TPGS2	0.057896	0.216661
GRINA	0.082117	0.216642
AP000763.4	-0.15405	0.216641
HCFC2	0.111829	0.216625
STAMBP	0.064606	0.216621
SLC35F5	-0.08409	0.216577
TIGD2	0.147303	0.216501
ICOSLG	-0.1743	0.216455
MRM3	-0.09406	0.216447
AL162431.4	0.635736	0.216432
AL133406.1	-1.12156	0.216419
CCDC65	0.16675	0.216352
TBXA2R	-0.37963	0.216339
EXTL3-AS1	-0.22266	0.216289
FBXL4	0.09056	0.216281
PSMC2	0.066173	0.21626
F2RL3	1.225052	0.216081
BLOC1S1	-0.17251	0.216078
LSP1P4	0.093459	0.216071
KIAA1328	-0.15625	0.216016

AC017083.1	0.33183	0.215961
OMG	-0.7493	0.215914
ZNF428	0.069691	0.215881
NANOS3	0.341372	0.215837
BCL10	0.106872	0.215785
PSPC1P1	1.072995	0.215776
AASDHPPT	-0.09	0.215729
MAK	-0.22794	0.215713
AC068768.1	0.723228	0.215709
TEX14	-0.57451	0.215682
RAB1A	0.061193	0.215566
RTKN2	-0.09621	0.215564
CCNB3	0.308576	0.21552
SLC44A3-AS1	0.360816	0.21549
AC024267.6	0.116222	0.215489
CLDN15	-0.0738	0.215468
SLC27A5	-0.09457	0.215393
ZZEF1	-0.07824	0.215393
LDHBP2	0.921977	0.215364
AL359924.1	-1.28352	0.215357
ZNF131	0.07318	0.215349
NIF3L1	-0.06837	0.215339
ZNF519	-0.12416	0.215236
NR4A1	0.127426	0.215207
SELENOH	0.061713	0.215207
AL133375.1	-0.16884	0.215193
KDM8	0.143407	0.215135
CHEK1	-0.10177	0.215066
P2RY4	-1.28742	0.215046
CSNK2A3	0.327451	0.215026
NA	-1.28178	0.214943
NA	0.655876	0.21494
TSACC	-0.2844	0.214922
MMD	0.056229	0.214905
RFESD	-0.21455	0.21486
AL355353.1	-0.97831	0.214856
NA	0.247809	0.214839
PPM1J	-0.69849	0.214826
ZBED5-AS1	0.188393	0.214817
COL5A3	-1.19948	0.21473
AL031864.1	1.495934	0.214727
NA	1.495934	0.214727
AC034111.1	1.121696	0.214694
MOB3B	0.239281	0.214677
YY1	0.055383	0.214664
LINC02021	0.428178	0.214653
STEAP1	-0.1623	0.214616
TPD52L2	-0.05095	0.214606
PABPC4-AS1	-0.06882	0.214587
SLC7A5P1	0.503236	0.214566

LAMP3	0.431404	0.21455
KIF1A	-0.07655	0.214534
ZNF808	0.139991	0.21441
NA	0.347485	0.214393
NA	-0.21445	0.214363
FLII	-0.06916	0.214331
SNORD124	-1.07699	0.214315
DLST	-0.0527	0.21429
BBC3	0.096265	0.214285
DCLK2	0.09283	0.214267
CSPG4P11	0.185849	0.214241
AL135905.1	0.420652	0.214201
AC017083.3	-0.13004	0.214193
AC060780.2	-0.39635	0.214139
PEX10	0.083043	0.214125
FAM168A	-0.06097	0.214099
SRPK3	-0.30994	0.214082
ETFBKMT	0.10798	0.214071
PPP2CB	0.062557	0.214003
SMARCD3	0.0942	0.213991
PTH1R	0.213367	0.213933
TMIGD2	-0.21198	0.213919
CYP27A1	-0.54532	0.213903
NA	0.068185	0.213889
NA	0.173992	0.213874
TANGO6	-0.10853	0.213814
FOXH1	0.125945	0.21375
HIST2H2BF	0.347848	0.213717
ERF	-0.06187	0.213669
GALNT11	-0.06644	0.213621
SDR39U1	0.067972	0.213615
AL603910.1	0.303814	0.213614
MED8	0.075119	0.213605
RF00560	0.854263	0.213599
AC106771.1	0.440775	0.213583
CCDC148	-0.67374	0.213583
ARNTL	0.084391	0.213556
RPF2	0.070467	0.213519
APOBEC3H	1.48764	0.213513
ZBTB45P1	1.48764	0.213513
AC011933.2	1.48764	0.213513
NFE2L3	-0.07132	0.213506
AC091180.3	0.060325	0.213495
SEZ6L2	-0.07218	0.213487
PDRG1	-0.08327	0.213466
IAPP	1.760136	0.213451
GALR3	1.760136	0.213451
FAM189A2	1.760136	0.213451
FAM178B	1.760136	0.213451
PFN1P10	1.760136	0.213451

RNU6-761P	1.760136	0.213451
PHBP7	1.760136	0.213451
RPL7AP26	1.760136	0.213451
PHBP11	1.760136	0.213451
LINC01351	1.760136	0.213451
ELL2P2	1.760136	0.213451
FBXL5	0.081945	0.21343
AL139128.1	0.981311	0.213421
MXD4	-0.07294	0.213412
ZNF286A	-0.0799	0.213381
PLS3	-0.08146	0.21336
AL161909.2	-1.41129	0.213337
AC010680.3	0.426451	0.213323
SHROOM4	1.298895	0.21328
TFAMP1	1.298895	0.21328
ERVFRD-1	1.298895	0.21328
LAMB3	0.173473	0.213269
RPL41P2	-0.57481	0.213266
MRFAP1	0.048011	0.21325
MRPL50	-0.07593	0.21321
SNAPC4	-0.07655	0.213203
ARHGAP18	-0.43283	0.213183
CYB5D2	0.097577	0.213168
RFC2	-0.06702	0.213118
ATP5MF	-0.06298	0.213051
LLPH	-0.09296	0.213046
PYCR3	0.113228	0.21303
TBL3	-0.07856	0.213029
NA	-0.07962	0.213003
AC090425.1	0.855731	0.212989
DHX36	0.052179	0.212967
ARHGAP33	-0.13366	0.21296
MT-CO1	0.048161	0.212952
AC009137.1	0.30197	0.212944
PSMD11	0.053823	0.212918
PSMD1	-0.06038	0.2129
CEP41	-0.07457	0.212831
PTAR1	0.065434	0.212813
AC097460.1	0.089072	0.212812
CCDC39	0.092874	0.212789
RPL5P1	0.644796	0.21278
RAI2	0.218388	0.21277
SCARA3	-0.11913	0.212767
ATE1	0.065297	0.212746
CLOCK	0.075679	0.21272
AF106564.1	0.08274	0.212692
ARL8B	0.059031	0.212658
ATP2A1	-0.1725	0.212653
NHSL1	1.29516	0.21265
AC092798.1	1.29516	0.21265

MIR1249	1.29516	0.21265
PARK7	-0.06656	0.212611
NFKBIB	0.074352	0.212515
AC004024.1	-1.09056	0.212512
ULK4P2	0.664869	0.212463
PLAGL2	-0.08649	0.21242
NEK3	-0.09625	0.21242
AC100827.2	0.687334	0.212362
PYCR2	0.082622	0.212295
ZEB2-AS1	0.294354	0.212277
ATP1A1-AS1	-0.07462	0.212257
AC020611.2	-0.52924	0.212239
TM4SF19-AS1	-0.73563	0.212188
RPL13AP25	0.294418	0.212178
BPNT1	-0.07486	0.212164
CLEC18A	-0.51967	0.212156
FGFR1	-0.08477	0.212156
MPP6	-0.07903	0.212149
ZNF570	-0.12925	0.212107
NA	-0.27814	0.21206
AL355987.4	-0.24876	0.212049
LHX6	-0.68694	0.21203
NA	1.219036	0.21203
EIF3J	0.061029	0.212027
NQO1	-0.08885	0.211955
PKD1L1	0.582949	0.211946
MAGI1-IT1	-1.3643	0.211934
LINC02244	1.63831	0.211911
NA	1.63831	0.211911
COBLL1	0.162184	0.211862
NA	0.147966	0.211858
Z95624.1	1.309807	0.211836
DGAT1	0.081588	0.21183
AC090607.1	0.168754	0.211805
YTHDF3-AS1	0.434245	0.211803
SNX18	-0.10502	0.211777
STARD3NL	-0.06653	0.211768
AL358937.1	-0.32191	0.211759
NA	0.088692	0.211746
RNU6-446P	-1.40394	0.211691
AL732414.1	-1.40394	0.211691
SKP2	0.066953	0.211674
NEK7	-0.08269	0.211596
NA	0.101328	0.211576
APMAP	-0.05361	0.211536
CHRNA7	-0.09549	0.211516
TPMT	-0.09475	0.211478
PTGS1	-0.1271	0.211474
SCFD1	0.078524	0.21145
TMEM52	0.829537	0.211449

ELOVL3	1.916913	0.211424
ANO8	0.098556	0.211332
CCT3	0.052918	0.211317
FILNC1	-0.55156	0.211214
AC099791.2	0.618493	0.211212
SGK2	0.57736	0.211166
UBE2E3	-0.05931	0.211099
A4GALT	0.265379	0.211075
CDC37P2	-0.99216	0.211067
ZNF622	-0.06716	0.211064
DYRK1A	-0.06157	0.211045
SLFN5	0.198791	0.21103
SSB	-0.06233	0.211025
TAF1A-AS1	-0.14574	0.21101
TRAF7	-0.05999	0.211009
NXF3	1.759739	0.21099
NPPC	1.759739	0.21099
ANKRD33	1.759739	0.21099
MIR643	1.759739	0.21099
LINC00412	1.759739	0.21099
AL139142.1	1.759739	0.21099
GTF2F2P1	1.759739	0.21099
AF235103.2	1.759739	0.21099
SAMD11P1	1.759739	0.21099
AC004551.1	-0.13866	0.210972
AGAP3	-0.05152	0.210954
AC067750.1	0.151159	0.210912
FUT2	0.291374	0.210911
AL022337.1	0.883428	0.21086
TBX10	1.759715	0.210838
TPSAB1	1.759715	0.210838
NA	1.759715	0.210838
AC073583.1	1.759715	0.210838
GJA6P	1.759715	0.210838
HDAC11-AS1	1.759715	0.210838
AC005479.1	1.759715	0.210838
AC026401.2	1.759715	0.210838
NA	1.759715	0.210838
MYBL2	-0.07146	0.210836
KCTD21	-0.08842	0.210824
PIP5KL1	0.186798	0.210711
TSSK2	-0.19956	0.210672
PEX1	0.068972	0.210594
DCAF13	0.061015	0.210584
LINC00525	-0.77757	0.210529
TMEM259	0.067235	0.210522
NA	0.878088	0.210515
AC099786.1	-0.77504	0.210492
COMP	0.283873	0.210461
DHX58	-0.797	0.210446

RNF7	-0.07344	0.210431
MAP2K7	0.075183	0.210423
PRPF19	-0.05176	0.2104
SLC6A12	0.483871	0.210396
AL365330.1	-0.12947	0.210387
ADAMTS10	-0.10489	0.210379
PROSER3	-0.10333	0.210331
NT5C	0.071019	0.210328
AL157791.1	-1.18598	0.210261
RHBDF2	0.109896	0.210244
FAM45BP	0.495174	0.210234
DIPK2A	1.074624	0.210221
NA	0.339524	0.210219
HSPA8P8	-1.27341	0.210192
GOLGA7B	-0.11628	0.210189
AC019118.1	-1.08943	0.210172
CEP104	-0.09268	0.210162
EIF2A	0.05456	0.21011
AL031727.1	-0.39032	0.210044
PAPSS1	-0.0521	0.210008
WWOX	0.130064	0.209973
CHAF1A	-0.07608	0.209951
ZNF124	-0.09352	0.209884
AL365361.1	-0.28606	0.209863
PFN1P3	0.41329	0.209717
AC012360.3	0.116877	0.209697
ESYT3	-0.34829	0.209694
SIL1	-0.09957	0.209681
MED10	0.061521	0.209664
PSKH1	-0.06696	0.209641
FRRS1L	0.130322	0.209638
WDR74	0.063987	0.209618
AC006111.1	0.289066	0.209604
AC034187.1	1.638695	0.209604
RPS3P2	1.638695	0.209604
AC024592.1	0.359489	0.209601
NOL9	-0.09498	0.209597
AC100810.1	-0.12718	0.209596
NTMT1	-0.07326	0.209574
GGA3	-0.0621	0.209572
MRPS18AP1	0.432544	0.209552
VDAC1P8	0.091272	0.209516
SLC40A1	-0.13734	0.209467
CRYM	-0.55217	0.209467
BCLAF1P1	1.638718	0.209461
PDXDC2P	1.638718	0.209461
CCDC91	0.077273	0.209438
MED19	-0.08792	0.209421
BTF3	0.057269	0.209368
AL359643.3	0.20648	0.209365

MFGE8	-0.08185	0.20936
GLS	-0.06301	0.209348
SDHAF2	-0.12101	0.209319
PSMD2	-0.04642	0.209319
POMGNT2	-0.09443	0.209296
AP005329.3	0.366424	0.209233
ZNF628	0.164691	0.209225
RAG2	0.189986	0.209167
AC108025.1	-0.51873	0.209163
ST20-AS1	0.353575	0.209156
SNU13	0.05739	0.209146
TAB3	0.072102	0.209138
SIN3B	-0.06857	0.209127
SDHDP6	-0.66451	0.209121
TRIM24	0.05082	0.20911
FSD1L	0.106783	0.209096
SH3BGR	0.233196	0.209047
CAPN9	0.921097	0.208983
PIDD1	0.076802	0.20894
NOS3	-0.23207	0.208926
AC135048.2	-0.53088	0.208903
MAPK8	0.06555	0.208864
DMWD	-0.07765	0.208852
HSPB1P2	1.070582	0.208843
DTX2P1	0.499403	0.208841
BOK-AS1	0.253695	0.208837
AL359878.1	0.890678	0.208832
FKBPL	-0.11283	0.208828
GNRHR2	-0.07966	0.20881
CALU	-0.0615	0.208809
DZIP3	-0.08204	0.208784
LINC01561	-0.28692	0.208775
PSMD10P1	1.086847	0.208733
THAP7-AS1	0.098795	0.208723
SNORA80E	-0.61821	0.208722
SH2D5	0.391998	0.208682
SMCO3	-0.89425	0.208664
SPATA32	-1.06614	0.208615
AC003986.2	0.106332	0.208595
NA	0.511961	0.208553
AC080013.5	0.809972	0.208522
NPNT	0.592821	0.208521
NA	-0.27135	0.208488
C19orf71	0.133412	0.208458
AC107222.1	-0.87228	0.208447
AC007728.1	-0.32717	0.208434
GPAT4	-0.05345	0.208423
TBC1D10C	-0.42509	0.208344
SNORD20	-0.60278	0.208334
RASL11B	-0.06894	0.208324

NEK5	-0.62407	0.208323
NUDCD3	0.051853	0.208273
NDUFV2P1	-0.15261	0.208262
LINC02268	0.126849	0.208239
ALKBH8	-0.13712	0.208233
OGDHL	0.087277	0.208219
C2CD5	0.057112	0.208217
CLDN4	0.243867	0.208177
MRPL22	0.087095	0.208156
AL731563.3	0.353504	0.208147
AC116347.1	-0.45757	0.20812
ZFYVE9	-0.0833	0.208119
MTA3	-0.0615	0.208078
RANBP1	0.053182	0.208074
MVK	0.094544	0.208064
JAKMIP3	-0.24393	0.208055
KDM5A	-0.05377	0.208026
AC109460.3	-0.59447	0.207987
SPAG16	0.110601	0.207982
DDC	-0.07312	0.207903
AC005498.3	-0.3989	0.207894
C17orf82	-0.3313	0.207888
AL357140.2	0.490089	0.207874
AC144548.1	0.515441	0.207866
AL353608.2	-0.62167	0.207852
GJC1	0.065999	0.207848
AC120042.2	0.900911	0.207837
ZIC2	-0.20603	0.207777
RHBDD2	0.075009	0.207766
AC009630.1	0.234714	0.207746
DBNDD2	-0.47106	0.207741
MRPS28	-0.13259	0.207721
SYNGR2	0.084912	0.207713
TRIM60P18	-0.16649	0.207602
MYLPF	-0.85807	0.207572
LRRC45	0.072519	0.207554
PELP1	-0.07309	0.20752
SLC11A1	0.616326	0.207493
FIP1L1	0.066633	0.20748
ANGPTL2	0.091172	0.20745
SLC2A4RG	0.084541	0.207365
LINC02259	1.120479	0.207313
NA	-0.15718	0.207293
NSD3	0.057546	0.207292
LINC02154	-0.63894	0.207271
MFSD6	-0.08338	0.20722
RNF24	0.083773	0.207217
IER5	-0.07506	0.207209
UBA2	0.048864	0.207198
IFT122	0.071358	0.207134

LINC02615	-0.25303	0.207106
NSMCE1-DT	-0.36505	0.207083
KIF22	-0.05347	0.207066
RNU6-986P	1.525444	0.207031
RNU6-504P	1.525444	0.207031
NOVA1	-0.15411	0.207026
KBTBD7	-0.09035	0.207003
NFKBIA	-0.06184	0.206999
AGPAT2	0.090812	0.206949
AC051619.6	0.435927	0.20694
PRELID2	-0.14079	0.206907
RPS27AP16	0.192101	0.206897
LONP1	0.054823	0.206877
TSPAN15	0.110852	0.20685
RNF219	-0.07836	0.20683
RAB38	0.820672	0.206826
KCNK7	0.468601	0.206794
AL731533.2	-0.67835	0.206788
ZSWIM5P2	1.114621	0.206759
AC107419.1	0.298674	0.206729
AC093323.1	0.087975	0.206698
ZBTB37	0.062223	0.206691
MAGEA2B	-0.26798	0.206651
CPSF2	-0.06792	0.206649
E2F3	0.0557	0.206626
RPL7A	0.056623	0.206622
MPG	0.084135	0.20661
ALG13-AS1	-0.72317	0.206588
PRSS36	0.488534	0.206524
RPL21P75	0.407962	0.206458
FOS	-0.27117	0.206453
AC139495.3	0.380555	0.206427
GUSBP4	-0.65239	0.206415
EPB41L4A	-0.0982	0.20639
TUG1	-0.04909	0.206363
PRPF38B	0.060387	0.206342
IFT74	-0.09398	0.206319
TRBV29OR9-2	-1.37969	0.206283
ZNF295-AS1	-1.37969	0.206283
ISLR	-0.13356	0.206276
CEP170	-0.05242	0.20626
GTF2IRD2	-0.11754	0.206232
APOM	0.118713	0.206197
AL807752.2	0.18217	0.206182
AC011451.1	0.358851	0.206131
GRIPAP1	0.072319	0.206128
LBX2	-0.19849	0.205999
NA	-0.06022	0.205986
F11R	0.120657	0.205963
AC007969.1	0.26179	0.205931

SPATA41	-0.55826	0.205931
AC141424.1	-0.3249	0.205923
RSAD1	-0.05838	0.20592
RPUSD3	0.092342	0.205897
AC093503.3	-0.06808	0.205896
AC019197.1	0.248829	0.205855
NDUFB4P12	1.984008	0.205818
CELA2A	1.984008	0.205818
MYO1A	1.984008	0.205818
CNBD1	1.984008	0.205818
F2	1.984008	0.205818
RF00019	1.984008	0.205818
RANP6	1.984008	0.205818
HSPD1P10	1.984008	0.205818
MIR1269A	1.984008	0.205818
TVP23CP1	1.984008	0.205818
AC097638.1	1.984008	0.205818
AL513008.1	1.984008	0.205818
NA	1.984008	0.205818
TIMM8BP2	1.984008	0.205818
AL357315.1	1.984008	0.205818
ASH1L-IT1	1.984008	0.205818
RPS29P17	1.984008	0.205818
AL590006.1	1.984008	0.205818
MYLKP1	1.984008	0.205818
AL450322.2	1.984008	0.205818
AC078842.2	1.984008	0.205818
AC027612.3	1.984008	0.205818
OR7L1P	1.984008	0.205818
AL117329.2	1.984008	0.205818
AC023051.1	1.984008	0.205818
RPS20P24	1.984008	0.205818
AC051618.1	1.984008	0.205818
GAPDHP61	1.984008	0.205818
TMED7-TICAM2	1.984008	0.205818
RNU6-795P	1.984008	0.205818
NA	1.984008	0.205818
RNU6-897P	1.984008	0.205818
AC102945.1	1.984008	0.205818
PTMAP15	1.984008	0.205818
HIGD1AP9	1.984008	0.205818
AC011120.1	1.984008	0.205818
AC010615.3	1.984008	0.205818
TP53TG3HP	1.984008	0.205818
AC090950.1	1.984008	0.205818
AP000436.1	1.984008	0.205818
AC104958.2	1.984008	0.205818
AL451074.5	1.984008	0.205818
FSCN1	-0.0566	0.205814
FBN1	-0.08908	0.205798

AC133473.1	1.355295	0.20574
DPM1	0.081205	0.2057
ZNF793	-0.09884	0.205696
ZNF394	0.068707	0.20569
STK32A-AS1	-0.98241	0.205675
RPAIN	0.055785	0.205674
TUBGCP6	-0.06404	0.205635
ARAF	-0.07882	0.205586
TP53I3	-0.08511	0.205558
RAD23BP1	0.603329	0.205549
NACA3P	0.717279	0.20552
RNU6-1272P	-1.25834	0.205519
NA	-1.25834	0.205519
AC092809.4	-0.17248	0.205508
NOL6	-0.06787	0.205466
FOXRED2	-0.08054	0.20546
MRPL32	0.070013	0.205458
ITGBL1	0.117094	0.205385
MICAL3	-0.08165	0.205381
DENND3	0.129451	0.205356
RAB14	-0.05666	0.205345
UPB1	0.263039	0.205198
C12orf10	0.068863	0.205156
ADAM15	0.057156	0.20514
AC068647.2	-0.83614	0.205086
ZNF85	0.139093	0.205028
AC007663.1	-0.56675	0.205024
NA	-0.11378	0.20496
AL627309.5	0.387997	0.204939
SLC27A6	0.075884	0.204936
SLC9A1	0.157786	0.204909
NOC2L	0.054583	0.204904
PSMD4	0.051715	0.204891
ERCC4	0.080682	0.204887
ZNF727	0.853204	0.20488
AC004771.1	-0.16169	0.204874
ACOXL-AS1	0.707283	0.204873
AL138799.2	-1.10007	0.204851
NEK10	0.334886	0.204838
HAT1	-0.07797	0.204829
LINC00641	-0.06169	0.204814
CAGE1	0.745307	0.204801
AC134312.1	0.62684	0.204779
NA	0.518927	0.204746
AL357874.1	0.944082	0.204738
ICE1	0.059553	0.204709
AC092143.3	1.525505	0.204705
TNFRSF11A	0.586142	0.204692
AC005559.1	-0.27149	0.204658
LRRC63	0.562185	0.204653

NA	-0.32305	0.204637
AGBL3	0.153279	0.204632
FRY	0.087644	0.204621
RPL23AP53	0.194411	0.204574
NA	-0.63999	0.204561
AL157871.3	1.525508	0.204561
SLC27A4	-0.08443	0.204508
GUSBP2	0.266746	0.204505
WDR93	-0.55936	0.204438
AC012485.1	0.932521	0.204435
NA	-0.46674	0.204422
HCAR1	0.159671	0.204405
AC104350.1	-0.37455	0.204404
JADE2	-0.07833	0.204343
FAM131C	0.385544	0.204323
AL391421.1	0.226884	0.204299
RF00591	0.844052	0.204298
C6orf120	0.069832	0.204239
FUCA2	-0.0711	0.20423
AC234582.2	0.222839	0.204177
AL365181.2	0.42452	0.204167
AC012379.1	0.311158	0.204149
AC005606.2	0.102149	0.204143
SPATA13	0.131043	0.204125
AC007424.1	1.176036	0.2041
CD81-AS1	0.13831	0.204078
C6	-0.20462	0.204072
ZNF847P	1.704988	0.204059
RNU6-1267P	1.704988	0.204059
AC099063.1	1.704988	0.204059
PRDM4	-0.07585	0.204032
DUBR	-0.12352	0.204018
DCLRE1B	-0.08052	0.204005
AC007066.2	-0.17402	0.204003
AC008937.3	-0.15704	0.204001
AC025884.1	-0.37801	0.203915
ARL6IP5	-0.06723	0.203818
TCF12	0.0621	0.203804
AC006064.3	0.575699	0.203769
SLC5A7	0.303174	0.203761
MOCS1	-0.08939	0.203744
SNRPGP2	0.195135	0.203742
COMMD2	0.065076	0.203728
CCDC142	0.08787	0.203728
CLK2	-0.05906	0.203706
SMYD2	-0.06494	0.203669
SETD7	-0.07192	0.203669
AL731563.2	0.211538	0.203604
LMF2	0.075165	0.203602
TTC31	0.064774	0.203554

HSPB3	0.520674	0.203535
AC006978.1	0.203712	0.203535
RETREG3	-0.0644	0.203514
FAM135A	0.094524	0.203481
GCOM2	0.612674	0.203455
ZNF17	0.136979	0.203343
C2CD4C	0.096493	0.203337
AC007406.3	-0.72245	0.203259
PDE1A	-0.65653	0.203238
LINC02593	0.080014	0.203217
FAM192A	-0.05832	0.203181
AC079305.1	0.17133	0.203177
ITGAV	0.050634	0.203164
DSE	-0.06667	0.203149
ASGR1	-0.17179	0.203136
AL691432.1	0.408311	0.203119
ABL1	-0.06469	0.203118
AC023043.1	0.251302	0.203103
NBAS	-0.07178	0.203097
TEDC2	-0.08197	0.20308
RHPN1-AS1	-0.75816	0.203078
SENP3	0.074966	0.203071
ANKLE2	-0.06082	0.202986
AL353807.4	0.393795	0.202969
HBE1	-0.87958	0.202958
RPL22	0.058037	0.202822
AC105935.1	0.926498	0.202803
NA	-1.29257	0.202798
AC002401.2	-0.89306	0.202764
PIWIL2	1.185104	0.202753
SEMA4G	-0.08396	0.202744
SCAT8	0.24404	0.202743
PTPRG-AS1	-0.14007	0.202719
HAL	0.375172	0.202718
AL109615.1	1.592313	0.202702
AC005237.1	1.592313	0.202702
C5orf58	1.592313	0.202702
PRXL2B	-0.08749	0.202664
RPL7P6	1.332601	0.202656
HMCN2	0.12916	0.202642
ETF1P2	1.097446	0.202608
VIM-AS1	0.065637	0.202597
SMARCC2	-0.06549	0.20259
PARP1	-0.04843	0.202579
TTF1	0.069916	0.202576
AL162431.2	0.173587	0.202564
AL035681.1	0.157333	0.202551
DCLRE1A	0.084873	0.202551
AL138963.1	-0.92661	0.202532
YTHDC1	-0.06252	0.202516

ASCL1	0.192737	0.202497
ELOCP28	1.304057	0.202449
AC254562.1	-1.30951	0.202429
AC010969.2	0.130368	0.20241
SHD	0.066311	0.202397
SYP-AS1	0.162632	0.202361
MTMR9	-0.07113	0.202351
LMNB1	-0.05312	0.20234
IRAK1BP1	0.088735	0.202274
GORASP1	0.065802	0.202273
MCU	0.095075	0.202269
AC009275.1	0.173221	0.202266
ERGIC1	-0.05258	0.202236
EFCAB7	0.107773	0.202221
ENOSF1	-0.0717	0.202219
ZNF547	-0.2257	0.202189
TRAPPC5	0.54032	0.202174
AC004492.1	0.269261	0.202145
AC136632.1	-0.29663	0.202096
SRGAP3-AS1	1.250055	0.202016
TEX264	-0.0743	0.202002
YY1AP1	0.069753	0.201983
TUBGCP4	-0.05952	0.201945
GTF2E1	-0.08533	0.201898
SRSF9P1	1.705452	0.201886
RNF138P1	1.705452	0.201886
AC009754.1	1.705452	0.201886
RNU4ATAC	1.705452	0.201886
RBM19	0.072709	0.201874
AP5S1	0.133723	0.201845
FAM86B2	-0.72729	0.201826
AC011462.2	1.266723	0.201823
PFDN6	0.062914	0.201818
CCT5	0.049191	0.201804
AC137894.1	1.014626	0.20178
HIC1	0.184395	0.20176
RNVU1-18	1.705481	0.201748
ARL6IP1P2	1.705481	0.201748
HSPB1P1	1.705481	0.201748
CD46P1	1.705481	0.201748
AP001021.3	1.705481	0.201748
AC138150.1	-0.12808	0.201705
AC097534.1	-0.12814	0.2017
CSDC2	-0.11896	0.201684
AC012636.1	0.314998	0.201634
TRIM65	-0.0664	0.201612
TMSB4XP4	-0.83484	0.201607
FOXS1	0.767613	0.201594
KCTD21-AS1	-0.09953	0.201562
ZFP2	-0.3753	0.201558

NSD1	-0.05234	0.201553
BCL3	-0.1906	0.201547
CUL4A	-0.05223	0.201544
AL139099.2	0.427098	0.20153
AC234782.1	-0.79702	0.201494
LINC01722	1.219451	0.201416
MIR212	1.246306	0.201396
CHMP2B	0.095678	0.201363
HAS2	-0.09623	0.201357
RBM42	0.068981	0.201356
NEDD4	0.085885	0.201347
AL391987.2	0.679693	0.20133
TAF1D	-0.05335	0.201327
FTH1P20	0.648282	0.201309
ORC5	0.069976	0.201283
AL445183.3	1.082536	0.201277
MYH10	-0.05618	0.201272
KLLN	-0.1776	0.20122
ANKRD36B	0.201549	0.201203
AL390816.2	1.23488	0.201197
AL445686.2	-0.27468	0.201187
RFXANK	0.082676	0.201162
RPS11P5	0.226886	0.201161
TSEN15	0.075239	0.201145
ECHDC1	0.065531	0.201142
CDCA7L	-0.21816	0.201139
AKR7A2	0.077075	0.201122
EIF4H	0.045912	0.201107
ITPR1-DT	0.289602	0.201093
NA	0.876629	0.20102
AL929601.1	-0.87848	0.200969
HDAC10	-0.22081	0.200946
COA1	0.057225	0.200932
ADCY9	-0.11089	0.200893
KLK2	0.784849	0.200892
PKIA	0.073284	0.20088
MED12L	-0.27041	0.200853
AC099677.4	-0.97859	0.20075
FKBP4	0.050371	0.200716
AC007390.2	0.075905	0.200674
NA	-0.6376	0.200672
CCT7	0.051492	0.20066
NUP88	0.062396	0.200657
PRR11	-0.0618	0.200639
AC140134.1	-0.17246	0.200618
NA	-0.23928	0.200586
NAT2	-0.73646	0.200543
PFKM	0.065332	0.20053
TRNAU1AP	0.084664	0.200525
UIMC1	0.065912	0.200492

SUN1	-0.04811	0.200449
EVI2A	1.592594	0.20042
OR7A5	1.592594	0.20042
RF00019	1.592594	0.20042
RPL23AP34	1.592594	0.20042
NA	1.592594	0.20042
CHI3L1	0.255509	0.200393
DSTNP1	0.247977	0.200383
FOXJ3	-0.05326	0.20037
PSTK	-0.11814	0.200366
CSRNP1	0.111807	0.200355
GABPA	0.066909	0.200331
NA	-0.31504	0.200328
NAPA-AS1	0.111152	0.200279
TYMS	-0.06226	0.200248
TDP1	0.079719	0.200241
MBD5	-0.08431	0.200237
JPH1	0.233577	0.200227
MIR4737	-0.67124	0.200223
AC116913.1	-0.3104	0.200217
PAN3	0.063138	0.200092
MITD1	0.097803	0.200086
ALKBH7	0.085668	0.200084
RNF220	0.053494	0.200064
BMP8B	0.123538	0.200053
TMEM138	0.072881	0.200049
AC122718.1	0.611078	0.200046
AL020997.3	-1.22372	0.200042
RBMXP2	1.322938	0.200034
AC129510.1	0.140657	0.199939
MT1A	-0.92234	0.199929
HMGB1	0.05347	0.199889
Z94721.2	0.472949	0.199845
NA	0.447445	0.199827
AC090572.3	-0.9613	0.199822
PRSS54	-0.32944	0.199814
FBXO48	-0.14297	0.1998
AL591846.1	0.403481	0.199785
MTND4P12	0.165418	0.199747
VRK3	0.079215	0.199674
PMS2P4	-0.14191	0.199642
EIF2B3	-0.08569	0.199634
MRPL48	0.075946	0.199625
CRYGS	0.231795	0.199604
NME3	0.073791	0.199595
MIR17HG	0.141859	0.199572
AC021231.2	-0.80796	0.199531
SPAG5-AS1	-0.06637	0.199499
WDR53	0.110209	0.199459
SVIL-AS1	0.084402	0.199408

MTCO1P28	0.467324	0.199408
NA	-0.90744	0.199393
PLPP2	0.069423	0.199347
PCDHA6	-0.80081	0.199329
ACVR1C	-1.02987	0.199307
ATF4P4	1.013218	0.199299
NDUFA5P11	1.013218	0.199299
RRBP1	-0.08205	0.199283
AC008781.2	-0.87814	0.199278
GTF2IP6	-0.67585	0.199249
AC004221.1	-0.57833	0.199226
CASP8AP2	0.092794	0.199156
AC005329.1	-0.09621	0.199115
SMN1	-0.07255	0.199112
LINC00589	-0.57225	0.199093
RLIMP2	0.201531	0.199087
C11orf54	-0.07841	0.199063
DNMT1	-0.05796	0.199049
DPT	0.150714	0.199046
NTS	-0.52728	0.199029
NELFCD	-0.05207	0.198984
CATSPERE	-0.38938	0.198973
MTRNR2L12	0.296339	0.198969
FAM207A	0.087517	0.198967
AC027031.2	-0.45568	0.198962
ARHGAP6	0.096633	0.198961
SNORA53	1.573818	0.198949
TUSC8	1.573818	0.198949
NA	1.573818	0.198949
AC007569.1	1.573818	0.198949
NA	1.573818	0.198949
AC008737.1	-0.79555	0.198935
NCAPH	0.062214	0.198909
PIK3CB	-0.10656	0.198874
TFEB	0.183159	0.198867
BAP1	0.050811	0.19886
RBM25	-0.04783	0.198859
C3orf52	-0.29391	0.198843
LYRM1	0.075471	0.198821
ZNF603P	-0.96263	0.198796
SMTN	-0.09064	0.198791
FAM201A	-0.44566	0.198759
VPS28	0.062982	0.198745
H3F3AP6	-0.32258	0.198732
RNU6-137P	-1.03582	0.198704
AC018638.2	-0.17393	0.198686
NA	-0.16758	0.198685
AL606760.2	0.128702	0.198644
AC020916.2	1.137351	0.19857
PARL	-0.06388	0.19846

RUFY1	0.05912	0.198456
NPR2	0.117701	0.19837
HYAL1	0.320983	0.198299
NPPB	-1.01322	0.198298
AK7	0.23221	0.198279
CBR3	0.158738	0.198248
RNA5SP315	0.727761	0.198243
RTN4RL2	0.391627	0.198239
LARS2	-0.07849	0.198235
AC027237.4	-0.18395	0.198199
CYB5RL	0.135125	0.198194
CCDC177	0.662426	0.198182
ANXA3	-0.40018	0.198159
AP000229.1	-0.28903	0.198146
RCC2-AS1	-0.06431	0.19812
SRXN1	0.206247	0.198092
NRROS	0.384704	0.198077
LINC01572	0.173581	0.198068
DRD4	0.256104	0.198064
RSU1	0.060352	0.198042
AGR2	-1.91919	0.198011
CEACAM8	-1.91919	0.198011
HOXD11	-1.91919	0.198011
BCL2A1	-1.91919	0.198011
MLIP	-1.91919	0.198011
IGF2BP1	-1.91919	0.198011
FOLR2	-1.91919	0.198011
C15orf54	-1.91919	0.198011
SH2D4B	-1.91919	0.198011
P2RY8	-1.91919	0.198011
ANKRD20A2	-1.91919	0.198011
OPTC	-1.91919	0.198011
SLC22A24	-1.91919	0.198011
RNU6-1147P	-1.91919	0.198011
RN7SKP71	-1.91919	0.198011
RF00019	-1.91919	0.198011
NA	-1.91919	0.198011
RNA5SP259	-1.91919	0.198011
NA	-1.91919	0.198011
POTEKP	-1.91919	0.198011
SNORA15	-1.91919	0.198011
RNU6-520P	-1.91919	0.198011
MIR595	-1.91919	0.198011
MIR623	-1.91919	0.198011
MIR579	-1.91919	0.198011
SNORA49	-1.91919	0.198011
RPL23AP4	-1.91919	0.198011
AL590762.2	-1.91919	0.198011
TBC1D26	-1.91919	0.198011
NA	-1.91919	0.198011

NA	-1.91919	0.198011
RAET1E-AS1	-1.91919	0.198011
AC022022.1	-1.91919	0.198011
PGAM1P6	-1.91919	0.198011
PPIAP8	-1.91919	0.198011
TRMT2B-AS1	-1.91919	0.198011
AL161793.1	-1.91919	0.198011
LYST-AS1	-1.91919	0.198011
AL118523.1	-1.91919	0.198011
AP000533.3	-1.91919	0.198011
SAP18P3	-1.91919	0.198011
PPP1R11P2	-1.91919	0.198011
AC152007.1	-1.91919	0.198011
RASA3-IT1	-1.91919	0.198011
LINC00385	-1.91919	0.198011
LINC01219	-1.91919	0.198011
AL645933.1	-1.91919	0.198011
NA	-1.91919	0.198011
LINC00993	-1.91919	0.198011
AL353718.1	-1.91919	0.198011
AL137847.2	-1.91919	0.198011
AL672277.1	-1.91919	0.198011
NA	-1.91919	0.198011
NA	-1.91919	0.198011
NA	-1.91919	0.198011
MTND1P3	-1.91919	0.198011
NA	-1.91919	0.198011
AC004594.1	-1.91919	0.198011
AC063952.2	-1.91919	0.198011
AL591242.1	-1.91919	0.198011
AC080013.2	-1.91919	0.198011
RN7SL78P	-1.91919	0.198011
AP003733.1	-1.91919	0.198011
NA	-1.91919	0.198011
AC080188.1	-1.91919	0.198011
CBX3P3	-1.91919	0.198011
SEPHS2P1	-1.91919	0.198011
NA	-1.91919	0.198011
AC109811.2	-1.91919	0.198011
NA	-1.91919	0.198011
RNA5SP464	-1.91919	0.198011
ETV3L	-1.91919	0.198011
AC022034.4	-1.91919	0.198011
AC040914.1	-1.91919	0.198011
AP002336.1	-1.91919	0.198011
AC087277.1	-1.91919	0.198011
AL591684.2	-1.91919	0.198011
CCDC58P5	-1.91919	0.198011
LINC02366	-1.91919	0.198011
NA	-1.91919	0.198011

NA	-1.91919	0.198011
Z83847.1	-1.91919	0.198011
NA	-1.91919	0.198011
NA	-1.91919	0.198011
MIR4286	-1.91919	0.198011
NA	-1.91919	0.198011
NA	-1.91919	0.198011
MIR3128	-1.91919	0.198011
NA	-1.91919	0.198011
NA	-1.91919	0.198011
NA	-1.91919	0.198011
SELENOKP1	-1.91919	0.198011
MIR3188	-1.91919	0.198011
NA	-1.91919	0.198011
LYPLA2P2	-1.91919	0.198011
AC062037.1	-1.91919	0.198011
RPL23AP90	-1.91919	0.198011
AL158211.4	-1.91919	0.198011
AC093673.2	-1.91919	0.198011
AC055764.2	-1.91919	0.198011
RRP12	-0.06852	0.197992
IER2	-0.05841	0.197949
UNC119	-0.07631	0.197932
KSR2	0.322213	0.197916
CATSPERG	0.301715	0.197877
AP001160.4	-0.91209	0.19786
EYA1	0.10657	0.197819
AC016582.2	0.468451	0.197816
AC009163.3	-0.512	0.197807
SNHG25	0.224907	0.1978
MARCH1	0.129751	0.197784
AC091429.1	0.821384	0.19778
ITIH4-AS1	0.761157	0.197769
AC007216.3	-0.3473	0.197754
CDKN1C	0.082643	0.197739
AC239802.1	0.711456	0.197716
EIF4HP2	-1.08969	0.197668
TPM3P8	-1.17629	0.19764
AL353708.1	0.775643	0.197638
ZNF696	0.083492	0.197632
PCDHA8	0.195751	0.197582
NA	0.709369	0.197579
AC010618.1	0.064077	0.197548
MRGPRF	-0.60022	0.197481
AC037459.3	0.173993	0.197476
HNRNPA2B1	0.049249	0.197466
UBE2F-SCLY	-0.84782	0.197455
AL133355.1	0.176796	0.197418
ZNF213-AS1	0.103839	0.197406
ALAD	0.074708	0.1974

BLOC1S2	-0.07318	0.197396
AP001767.3	0.269335	0.197348
SCO1	-0.06401	0.197318
AC006378.1	-0.40009	0.197209
AC011491.2	-1.00835	0.197133
ABCC2	-0.33649	0.197132
SLC26A2	0.075115	0.197093
RFX3	-0.06796	0.19706
PDLIM4	0.081982	0.197057
LINC00973	-1.22146	0.197052
HNRNPA1P62	-1.22146	0.197052
NLRC3	0.252563	0.197016
AC021491.2	-1.33838	0.197005
NA	0.066027	0.19694
TVP23C	-0.18133	0.196938
AC244197.2	0.320931	0.196935
TRIAP1	0.081587	0.196915
CDK11B	0.079734	0.196901
AL160313.1	-0.68148	0.196898
RB1	-0.06231	0.196891
GTF2B	-0.08236	0.196824
AC092134.1	-0.23715	0.196804
PLAT	0.064575	0.196792
PLA2G3	1.9124	0.196753
AGXT2	1.9124	0.196753
NKX2-5	1.9124	0.196753
RNU1-72P	1.9124	0.196753
SNORA71D	1.9124	0.196753
RF00019	1.9124	0.196753
NA	1.9124	0.196753
RNU6-1201P	1.9124	0.196753
RAC1P5	1.9124	0.196753
NPIPA8	1.9124	0.196753
TMCO5B	1.9124	0.196753
NA	1.9124	0.196753
TDRD15	1.9124	0.196753
NA	1.9124	0.196753
SNORA77	1.9124	0.196753
RN7SKP240	1.9124	0.196753
TUBB4BP2	1.9124	0.196753
OR5AW1P	1.9124	0.196753
LINC01293	1.9124	0.196753
AC009518.2	1.9124	0.196753
SLC26A4-AS1	1.9124	0.196753
AL136982.3	1.9124	0.196753
AC119800.1	1.9124	0.196753
NA	1.9124	0.196753
SCARNA18	1.9124	0.196753
AC098583.1	1.9124	0.196753
AC113615.1	1.9124	0.196753

RNU6-1206P	1.9124	0.196753
RNU6-1337P	1.9124	0.196753
RNU7-143P	1.9124	0.196753
AC109635.3	1.9124	0.196753
AC136475.3	1.9124	0.196753
AL357153.2	1.9124	0.196753
CERS3-AS1	1.9124	0.196753
AC060809.1	1.9124	0.196753
ACTG1P15	1.9124	0.196753
HCCAT5	1.9124	0.196753
NA	1.9124	0.196753
MIR4731	1.9124	0.196753
MIR4644	1.9124	0.196753
NA	1.9124	0.196753
AC007923.4	1.9124	0.196753
NA	1.9124	0.196753
AL031320.1	1.9124	0.196753
AL355877.2	1.9124	0.196753
AL357054.3	1.9124	0.196753
NA	1.9124	0.196753
ZNF484	0.116921	0.196748
ZNF732	-0.79006	0.196745
NA	0.111115	0.196731
DOC2A	0.080114	0.196684
ZDHHC11	-0.23772	0.196682
AC131206.1	1.574002	0.196667
NFYB	-0.06287	0.196648
ZFYVE16	0.064811	0.196603
AC093525.5	-0.55367	0.196587
DSTN	-0.05244	0.19657
AC093274.1	0.994409	0.196541
AC091100.1	0.864452	0.196528
RPL13P4	1.574014	0.196526
HSPE1P4	1.574014	0.196526
NA	1.574014	0.196526
KIAA0556	0.082779	0.196459
KCNQ3	-0.13034	0.196429
PSMG3	0.061059	0.196395
AC092127.2	-1.16402	0.196392
TSPAN19	-0.42483	0.196385
LAMA5	-0.05837	0.196356
FKBP3	0.079009	0.196341
NA	-1.00222	0.19629
AL138724.1	-0.10073	0.196271
AC092835.1	-0.20065	0.196256
AC021739.3	-0.60724	0.196248
PTMAP9	-0.67364	0.196206
USP30	0.094399	0.196183
NA	-0.10441	0.196168
NDUFB7	0.063756	0.196145

AC093157.2	-0.58237	0.196138
RAB2B	0.057711	0.196101
KLF9	0.097174	0.196076
FAM149B1	-0.05655	0.196071
CARD8	0.067138	0.196065
EIF4G2	-0.04435	0.19602
SMN2	0.089294	0.19601
PKM	-0.04789	0.195998
AL355581.1	-0.49706	0.19598
PAF1	-0.05731	0.195876
EIF3I	-0.04579	0.195863
CORO1B	0.062572	0.195835
AL355488.1	-0.13074	0.19582
CDC7	0.0887	0.195809
KDM5B	-0.06291	0.195781
SERPINA1	-0.4539	0.195775
CDH3	0.637428	0.195738
AP002748.3	-0.15671	0.195722
ANKRD13B	0.071421	0.195628
ATP10D	0.072302	0.195609
AL445250.1	0.381478	0.195584
NA	-0.19065	0.195541
PROKR1	1.090956	0.195538
MT-TP	0.076594	0.195525
SPATA5L1	-0.09735	0.195486
AL031289.1	-0.50383	0.195448
AC090181.2	0.348017	0.195412
AC072022.1	0.974653	0.195358
RF00334	-1.19778	0.195357
NCOA5	-0.06287	0.195348
KRT8	-0.20076	0.195345
BMI1	-0.06687	0.195322
NA	0.207431	0.195293
MYO1D	0.092136	0.195283
AF230666.2	0.903325	0.195279
AC019185.2	-0.98728	0.195256
GGCT	0.058544	0.195206
ETFB	-0.13269	0.195191
LINC01356	0.262525	0.195182
SLC23A3	-0.15743	0.195168
CDK1	0.071477	0.195165
DGCR9	-0.41686	0.195158
CGN	0.161671	0.195132
AC087620.1	0.63112	0.195099
LINC00526	-0.17306	0.195098
PHACTR4	-0.05364	0.195049
GJD4	1.305883	0.195016
LINC02476	1.305883	0.195016
AC091117.2	1.305883	0.195016
TRAPPC9	-0.09534	0.19497

ARHGEF33	-0.42017	0.194969
AL121917.2	-1.32849	0.194918
FIGNL1	-0.07205	0.19491
VPS26B	-0.05429	0.194886
ZNF550	-0.07591	0.194854
RNU6-288P	1.359912	0.19485
AL109615.2	1.359912	0.19485
NA	-0.72328	0.194845
ZC3H10	0.087253	0.194801
AC127537.1	-1.08289	0.194747
AC096992.2	-0.29495	0.194731
ICMT	-0.07736	0.194723
GNA13	-0.05648	0.194699
CYP2E1	0.927587	0.194648
GSTM3	0.077094	0.194643
AC018648.1	-0.30944	0.194643
DND1P1	0.225772	0.194628
SPTLC1	-0.05443	0.194622
BPTF	-0.0514	0.194613
RNA5SP323	1.050042	0.19461
NA	-0.54497	0.194574
SPATA3-AS1	0.561076	0.194564
MED7	-0.09577	0.194561
MED14OS	-0.15675	0.194544
TNFSF18	-0.37985	0.194514
CREBBP	-0.07655	0.194476
PCDHB6	-0.11341	0.194463
MIR600HG	0.105561	0.194463
ELF1	-0.06591	0.194451
AC253536.6	-0.3468	0.194445
PCDHB12	0.220733	0.194437
ZNF33BP1	1.067961	0.194392
U2AF1	0.049988	0.19439
SMG5	-0.05738	0.194373
EXTL1	-0.24922	0.194369
AC008940.1	-1.44432	0.194355
RSL1D1	0.046588	0.194296
LRRFIP1	0.058962	0.19425
CHRDL1	-0.17919	0.194249
THBS4	-0.13661	0.194234
MSRB1	-0.08186	0.194188
NUCB1	0.059605	0.194172
AC108449.1	-0.2869	0.194119
RASD2	-0.33878	0.194113
ARNT2	-0.06441	0.194105
CIRBP-AS1	0.21424	0.194058
CDC25A	-0.07228	0.194015
CCDC173	-0.44279	0.194014
AL669831.1	0.202301	0.194013
NCOR2	-0.08713	0.194004

PPM1B	0.056076	0.193982
ASS1	0.150377	0.193946
C10orf25	-0.27125	0.193941
SPCS3	0.067737	0.193937
CCNB2	0.076316	0.193901
AK2	0.055687	0.193842
CDK2AP2P2	-0.60661	0.193727
AC004156.1	-0.06185	0.193726
AC009065.5	-0.35917	0.193701
AL121871.1	-1.19045	0.193697
TRADD	-0.10579	0.193636
INTS4P1	0.193056	0.193629
GUCD1	-0.05572	0.193621
ZNF726	-0.15242	0.193612
AC012213.1	-0.24705	0.193605
WNK2	-0.08906	0.193603
YWHAE	0.056762	0.1936
CNNM2	-0.06008	0.193586
C2orf70	0.585673	0.193545
ERCC1	-0.05123	0.193523
ZCCHC8	0.060815	0.193486
RNF167	-0.05604	0.193474
RYK	0.064163	0.193474
XBP1P1	0.442877	0.193401
IPPK	-0.06513	0.193376
RNA5SP352	0.779711	0.193364
NPIPA7	-0.81901	0.193351
WDR37	-0.07178	0.193335
GPT2	0.070919	0.193317
TAMM41	-0.11244	0.193283
MEIG1	0.449625	0.193239
SNORD12	0.466949	0.193238
NA	-0.41867	0.193215
RALGAPA2	-0.08504	0.193194
NA	-0.14905	0.193145
GNLY	1.883375	0.193113
CASQ2	1.883375	0.193113
SSTR1	1.883375	0.193113
LCN9	1.883375	0.193113
OR1K1	1.883375	0.193113
WSCD1	1.883375	0.193113
PDSS1P1	1.883375	0.193113
EEF1GP1	1.883375	0.193113
FGF16	1.883375	0.193113
GALP	1.883375	0.193113
RN7SKP185	1.883375	0.193113
RNU6-1112P	1.883375	0.193113
RNU6-770P	1.883375	0.193113
RF00019	1.883375	0.193113
NA	1.883375	0.193113

RNASE13	1.883375	0.193113
RF00019	1.883375	0.193113
NA	1.883375	0.193113
NA	1.883375	0.193113
CBX1P4	1.883375	0.193113
AL354710.1	1.883375	0.193113
CYCSP55	1.883375	0.193113
HMGB1P4	1.883375	0.193113
EIF5P1	1.883375	0.193113
GPX1P2	1.883375	0.193113
LAP3P1	1.883375	0.193113
AC006210.1	1.883375	0.193113
EIF4A2P4	1.883375	0.193113
LINC01691	1.883375	0.193113
SRP14P1	1.883375	0.193113
AC112198.1	1.883375	0.193113
MGAT3-AS1	1.883375	0.193113
SFR1P1	1.883375	0.193113
PSMD8P1	1.883375	0.193113
SMIM10L2B-AS1	1.883375	0.193113
AC004485.1	1.883375	0.193113
TMPRSS11GP	1.883375	0.193113
GK-IT1	1.883375	0.193113
AC087163.1	1.883375	0.193113
AC006994.1	1.883375	0.193113
CLIC1P1	1.883375	0.193113
PHKA1-AS1	1.883375	0.193113
LINC01705	1.883375	0.193113
MTCO1P5	1.883375	0.193113
AL133343.2	1.883375	0.193113
AC009305.1	1.883375	0.193113
GYG2-AS1	1.883375	0.193113
BZW1P1	1.883375	0.193113
AC025822.2	1.883375	0.193113
CATIP-AS2	1.883375	0.193113
NFIA-AS1	1.883375	0.193113
NA	1.883375	0.193113
NA	1.883375	0.193113
RPS4XP22	1.883375	0.193113
BET1P1	1.883375	0.193113
RPL21P54	1.883375	0.193113
PPAN-P2RY11	1.883375	0.193113
RPS12P20	1.883375	0.193113
AC106872.3	1.883375	0.193113
AC025263.1	1.883375	0.193113
HOXC-AS2	1.883375	0.193113
AC105254.1	1.883375	0.193113
AC098591.2	1.883375	0.193113
NA	1.883375	0.193113
RAB5CP2	1.883375	0.193113

NA	1.883375	0.193113
SNORD116-26	1.883375	0.193113
RNU6-314P	1.883375	0.193113
RNU6-1088P	1.883375	0.193113
RNU6-367P	1.883375	0.193113
NA	1.883375	0.193113
CRYZL2P-SEC16B	1.883375	0.193113
HNRNPA1P76	1.883375	0.193113
AC079917.1	1.883375	0.193113
AC103843.2	1.883375	0.193113
AP003174.1	1.883375	0.193113
NA	1.883375	0.193113
AC078962.3	1.883375	0.193113
AL121852.1	1.883375	0.193113
NBEAP1	1.883375	0.193113
DYNLL1P2	1.883375	0.193113
AL133371.1	1.883375	0.193113
AL358333.2	1.883375	0.193113
AC018563.1	1.883375	0.193113
AC105129.2	1.883375	0.193113
AC026951.1	1.883375	0.193113
AC019294.2	1.883375	0.193113
TXNP4	1.883375	0.193113
NA	1.883375	0.193113
NA	1.883375	0.193113
AC090616.2	1.883375	0.193113
AC239868.1	1.883375	0.193113
NA	1.883375	0.193113
RN7SL378P	1.883375	0.193113
NA	1.883375	0.193113
SLC14A2-AS1	1.883375	0.193113
RNFT1-DT	1.883375	0.193113
BNIP3P26	1.883375	0.193113
DDX55P1	1.883375	0.193113
HSPE1P14	1.883375	0.193113
SNX29P2	1.883375	0.193113
AC025754.2	1.883375	0.193113
RBPMS	-0.13671	0.193112
H2AFX	-0.07917	0.193104
DRD2	-0.14564	0.19306
NA	-1.27905	0.193056
RBMS1	0.062904	0.193015
RF00006	-1.14852	0.192994
C20orf194	0.07874	0.192988
PSD2	-0.35704	0.192972
LINC02256	-0.51663	0.192952
TRIM56	0.194952	0.192945
AC008243.1	0.898201	0.192936
LAMC3	0.570211	0.192899
POLR3B	-0.08239	0.192885

SERHL2	0.180816	0.19285
MST1L	-0.3388	0.192843
AC091564.5	0.155556	0.192829
HLA-F	0.418606	0.192826
AC012313.8	-0.40506	0.192825
IQCK	0.111076	0.192823
AL159987.2	-0.38504	0.192802
BRD3	-0.05512	0.192798
CNTNAP4	0.639275	0.192772
PRR13	0.078499	0.192761
CCDC158	0.413439	0.19275
BNC2-AS1	-0.50968	0.192737
TSR3	0.068796	0.192735
ACTG1	0.060097	0.192729
NA	0.696174	0.192723
AC018467.1	1.386856	0.192721
MSC-AS1	0.168383	0.192715
AC006064.2	-0.06402	0.192702
REL	0.112599	0.192672
COX5BP6	-0.67345	0.192668
TBC1D23	0.064971	0.192622
PTPRB	-0.16215	0.192606
RPL35P2	0.460235	0.192587
NA	-0.20233	0.192492
SLC22A10	-0.53584	0.19249
CD58	-0.12233	0.192488
XPNPEP3	-0.07412	0.192483
PDCD7	0.065891	0.19248
SNHG17	0.080561	0.192475
AC016394.1	-0.19027	0.192467
SLC25A36	0.056077	0.192441
FASTKD5	-0.06796	0.192434
EFCAB14	-0.06077	0.19243
AL662791.1	-0.58503	0.192421
ZNF787	0.074408	0.192389
FEN1	-0.06125	0.192323
AC107068.1	0.137699	0.192323
GHRLOS	-0.58758	0.192322
CETN2	0.074983	0.192295
SCAF1	-0.06802	0.19229
MUC19	0.832305	0.192269
GPC5	-0.37818	0.192199
KCTD6	-0.09598	0.192189
AC105036.2	-0.9333	0.192182
TUBB3	-0.12125	0.192181
PDHA1	0.058578	0.19218
DGKG	-0.24268	0.192171
AL392172.1	-0.21894	0.192162
NA	-0.12308	0.192145
NAA38	0.055994	0.192131

AC012254.4	-0.226	0.192095
AC007256.1	-1.30524	0.192053
MICOS10P2	-1.30524	0.192053
EMG1	0.056315	0.191977
CCDC89	0.246538	0.191971
COX20P1	-0.1943	0.191888
AL445991.1	-0.91261	0.191884
MYO5A	-0.07855	0.191884
ABCD1	-0.08848	0.191854
AL513477.2	-0.57718	0.19185
AC002553.1	0.705742	0.191818
AC024563.1	-0.43971	0.191798
SOS1	-0.05665	0.191797
ALDH7A1	0.058982	0.191782
XKR7	-0.08148	0.191762
NSUN5	0.058761	0.191745
ALG1L13P	0.205291	0.191737
FBXW10	0.651671	0.191734
AKIRIN2	0.066365	0.191733
FES	0.36224	0.191712
SRP68	0.049511	0.191698
AK9	-0.14301	0.191647
NA	0.119932	0.191634
FGFRL1	0.203278	0.191549
AC004637.1	-1.08052	0.191548
RELA	-0.06105	0.191546
TMEM165	0.059754	0.19154
ZNF43	-0.07581	0.191537
PLA1A	1.202982	0.191531
AL096803.2	1.202982	0.191531
AC009511.2	-0.11341	0.191512
RNU1-103P	-1.13997	0.191508
WDTC1	-0.08394	0.191489
SFRP5	-1.27162	0.191459
ZNF429	-0.13604	0.191446
AMZ2	-0.06395	0.191438
ITGB3BP	0.074014	0.19141
SLC9B2	-0.06532	0.191377
AL122035.1	-0.13884	0.191286
CAND1	0.049566	0.191276
NA	-0.43302	0.191253
LINC01122	0.410129	0.191237
CADPS	-0.1839	0.191199
AC124944.1	-0.48187	0.191198
MIRLET7D	-0.45934	0.191192
RASL12	-1.44434	0.191174
NA	-1.44434	0.191174
AL136380.1	-1.44434	0.191174
AC008026.3	-1.44434	0.191174
FIGN	-0.07575	0.191142

SNX1	-0.04545	0.191091
SACS	-0.06843	0.191082
RGS12	0.069039	0.191064
MAPKBP1	0.082913	0.191034
TJP1	-0.05591	0.191033
TMEM30A	-0.04998	0.191018
AL359232.1	-0.67286	0.191008
VAMP5	0.135484	0.191007
L34079.2	-0.16091	0.190986
NA	0.870447	0.190945
LPP-AS2	-0.87096	0.190939
RPL13AP5	0.124086	0.190922
PROM2	0.351148	0.190904
TUBG1	-0.07112	0.190893
SLC6A17	0.463978	0.19087
AL049870.3	-1.05861	0.190784
P4HA3	0.130574	0.190783
TAFA5	0.294637	0.190782
CFAP43	0.401203	0.190715
AC138646.1	-0.54778	0.190695
GRIK5	-0.08287	0.190689
ZNF341	0.160902	0.190626
OTX1	0.846917	0.190595
NA	0.221594	0.190546
ARHGEF40	-0.05458	0.190544
NA	0.882901	0.190507
NUTM2B-AS1	-0.16748	0.190492
PAFAH1B1	-0.0482	0.190489
D2HGDH	0.08655	0.190456
EIF5AL1	-0.30941	0.190452
PRKAA1	0.063944	0.190449
NA	-0.23365	0.19042
RPLP1P6	0.776046	0.190395
RHOQP1	-0.36062	0.190375
CORT	0.441812	0.190364
DKK2	-0.06663	0.190358
NA	0.187053	0.190322
IQGAP2	0.200163	0.190321
FCGR1A	0.630186	0.190301
SUCLA2	0.062261	0.190291
MIR1285-1	-0.79316	0.190287
CELSR2	-0.0853	0.190218
DDC-AS1	0.0937	0.190206
ZNF516	-0.09426	0.190203
ZFP36L1	-0.13641	0.190092
CCDC47	0.046309	0.190038
NUDT22	0.072367	0.190027
AP000943.3	-0.34451	0.189966
CTSW	0.510326	0.189953
C11orf49	0.073198	0.189929

AC025419.1	-0.72757	0.189919
AL121992.3	0.302021	0.189915
C10orf91	0.910265	0.189909
SAT2	0.08333	0.189862
PTAFR	0.708158	0.189842
AC008429.2	0.733073	0.189837
AC008014.1	-0.34541	0.189836
QTRT1	-0.06499	0.18982
PRAP1	-0.7715	0.189788
AC025175.1	-0.8315	0.189758
ZBED5	0.066784	0.189754
SHC4	-0.24502	0.189691
NA	0.363735	0.189678
NA	0.146893	0.189663
FANCA	-0.06555	0.189609
ESRRG	-0.07798	0.189599
DLEU1	-0.11786	0.18959
ZRANB2	0.060178	0.189583
AL121658.1	0.440948	0.189573
KCNB1	0.321192	0.189569
CC2D1A	-0.07569	0.189563
KLHDC4	-0.078	0.189551
CHTF8	0.061703	0.189546
TSNARE1	-0.09914	0.189544
POLA1	-0.07166	0.18953
TMEM11	0.07884	0.189527
KIF1C	-0.0778	0.18952
DUSP3	-0.07074	0.189511
ABHD15-AS1	-0.86505	0.189467
AC137695.2	-0.66764	0.18943
C8orf48	-0.18645	0.189345
AVIL	0.187902	0.189323
UBE2FP3	-0.48578	0.18931
AC026412.2	1.017221	0.189258
C11orf72	-0.85451	0.189237
TRAF3IP1	-0.08399	0.189219
CRYBG2	0.768737	0.189197
ZBED3	0.086549	0.189187
BARX2	-1.84846	0.189161
MEFV	-1.84846	0.189161
TP53AIP1	-1.84846	0.189161
ASB15	-1.84846	0.189161
ZNF208	-1.84846	0.189161
LINC01931	-1.84846	0.189161
TDRD6	-1.84846	0.189161
LINC00846	-1.84846	0.189161
RF00554	-1.84846	0.189161
RPL17P34	-1.84846	0.189161
CHCHD2P2	-1.84846	0.189161
AL139039.2	-1.84846	0.189161

MIR1301	-1.84846	0.189161
MIR548O	-1.84846	0.189161
AL590632.1	-1.84846	0.189161
AC092598.1	-1.84846	0.189161
SEC13P1	-1.84846	0.189161
AL500527.1	-1.84846	0.189161
AL355613.1	-1.84846	0.189161
CFL1P3	-1.84846	0.189161
AL158069.1	-1.84846	0.189161
BX284668.2	-1.84846	0.189161
AL034428.1	-1.84846	0.189161
AL359095.1	-1.84846	0.189161
AL035588.1	-1.84846	0.189161
AC104462.1	-1.84846	0.189161
AL078645.2	-1.84846	0.189161
AC074366.1	-1.84846	0.189161
AL356309.2	-1.84846	0.189161
AC004865.1	-1.84846	0.189161
DDX10P1	-1.84846	0.189161
AL445489.1	-1.84846	0.189161
TTLL11-IT1	-1.84846	0.189161
RF00012	-1.84846	0.189161
RF01233	-1.84846	0.189161
NA	-1.84846	0.189161
RF00096	-1.84846	0.189161
AC117422.1	-1.84846	0.189161
AC112206.2	-1.84846	0.189161
AC026774.2	-1.84846	0.189161
RNA5SP165	-1.84846	0.189161
AC146944.3	-1.84846	0.189161
AC012413.1	-1.84846	0.189161
AC068587.2	-1.84846	0.189161
AC126763.1	-1.84846	0.189161
AC127455.1	-1.84846	0.189161
AC022069.1	-1.84846	0.189161
AC009303.3	-1.84846	0.189161
AL157392.4	-1.84846	0.189161
GALNT4	-0.67233	0.189158
SNX33	-0.08682	0.189158
CLSTN3	0.210881	0.189101
RPS4XP3	-0.54418	0.188994
CATSPER2	-0.17016	0.188994
RF00019	-0.98875	0.188982
AC008592.3	-0.98875	0.188982
NA	0.494961	0.188981
RRS1	-0.06307	0.188934
AC073073.2	-0.13684	0.188925
RNU6-141P	-1.44436	0.188915
SPON1-AS1	-1.44436	0.188915
AC107918.3	-1.44436	0.188915

AC008280.3	-1.44436	0.188915
LENG8	-0.06451	0.18886
SLCO4C1	-0.34158	0.188856
GTF2IRD2B	0.099217	0.188824
RNF5P1	-0.60388	0.188795
TMEM186	-0.0751	0.188785
IQUB	-0.52076	0.188781
WBP11	-0.05587	0.188781
TTC21A	0.147472	0.188761
FUT10	0.130422	0.188745
DCTN1-AS1	-0.1505	0.188736
AC114939.1	-0.34707	0.188728
DRICH1	-0.3422	0.18871
OXA1L	0.053851	0.18868
CR392039.1	0.535284	0.188627
GGA1	-0.05469	0.188615
TFCP2L1	-1.84397	0.188603
ACADL	-1.84397	0.188603
SLX1A	-1.84397	0.188603
FAM107A	-1.84397	0.188603
RPL12P13	-1.84397	0.188603
LIPM	-1.84397	0.188603
DES	-1.84397	0.188603
ZNF648	-1.84397	0.188603
TPM3P7	-1.84397	0.188603
RF00019	-1.84397	0.188603
RN7SKP239	-1.84397	0.188603
RNU6-213P	-1.84397	0.188603
SNORA20	-1.84397	0.188603
AP000936.1	-1.84397	0.188603
AC005014.1	-1.84397	0.188603
AP001627.1	-1.84397	0.188603
PDZPH1P	-1.84397	0.188603
AC068535.1	-1.84397	0.188603
HMGN1P2	-1.84397	0.188603
TCP1P1	-1.84397	0.188603
AL022324.1	-1.84397	0.188603
AL596087.3	-1.84397	0.188603
AP001059.1	-1.84397	0.188603
AL078590.2	-1.84397	0.188603
AC016712.1	-1.84397	0.188603
AL590648.2	-1.84397	0.188603
MTND4P14	-1.84397	0.188603
HNRNPA1P54	-1.84397	0.188603
PRR20G	-1.84397	0.188603
RN7SL382P	-1.84397	0.188603
RN7SL681P	-1.84397	0.188603
PPATP1	-1.84397	0.188603
AC099340.1	-1.84397	0.188603
AC096745.1	-1.84397	0.188603

Z99943.2	-1.84397	0.188603
RNU6-1170P	-1.84397	0.188603
AC024451.3	-1.84397	0.188603
RANP3	-1.84397	0.188603
AC023050.4	-1.84397	0.188603
NA	-1.84397	0.188603
AC087481.1	-1.84397	0.188603
AC132825.3	-1.84397	0.188603
AC013565.3	-1.84397	0.188603
AL079343.1	-1.84397	0.188603
AP000919.1	-1.84397	0.188603
AC093484.2	-1.84397	0.188603
NA	-1.84397	0.188603
AC011444.2	-1.84397	0.188603
AL158151.4	-1.84397	0.188603
AL031429.2	-1.84397	0.188603
AC104836.1	-1.84397	0.188603
ZBTB40-IT1	0.733797	0.188534
COG5	0.063033	0.188355
PTCH2	-0.24653	0.188339
AL049869.3	0.231983	0.188325
AC068875.1	0.952536	0.1883
MYO9A	0.07764	0.188299
TMPO	0.048982	0.188284
AC096586.2	-0.28086	0.188214
CTTN	-0.04508	0.18816
AL359636.2	-1.38758	0.188133
CPSF6	-0.04603	0.188107
SLC35B2	0.06092	0.188104
STARD9	0.073294	0.188071
EXOC3	-0.06241	0.188063
FAF1	0.057259	0.188052
AP000781.1	-0.06927	0.188049
USP31	0.082579	0.187985
TXN2	0.06208	0.187978
AC010601.1	0.946925	0.187971
AC004846.2	0.273588	0.187923
B3GALT5	-0.21674	0.187901
AC244517.2	0.165699	0.187801
ATG3	-0.0621	0.187745
TMEM255B	-0.21915	0.187696
HCG4P8	-1.49509	0.187658
AC003001.1	-1.49509	0.187658
RN7SL806P	-1.49509	0.187658
LINC02014	-1.49509	0.187658
MIR181A1HG	1.008659	0.187557
AL133353.1	-0.72967	0.187535
NA	-0.43254	0.187532
AP005717.1	0.904324	0.187488
NA	0.375724	0.187477

CICP16	0.197621	0.187454
PIN4	0.067145	0.187426
ZNF680P1	-0.49441	0.187346
CNKSR3	-0.08394	0.187199
ISM1	-0.42786	0.187174
SIRT2	-0.08027	0.187141
OPA1	-0.05886	0.187106
NA	-1.49217	0.187104
AL138799.4	-1.49217	0.187104
SNORD127	-1.49217	0.187104
AC020900.1	-0.12592	0.187077
CEP57L1	0.103866	0.187057
FBXO36	0.136379	0.187034
SAMD4B	-0.06076	0.187024
FBXL13	-0.11379	0.187022
ZNF451-AS1	-0.11175	0.187011
AL359881.2	0.718771	0.187008
SUMO2P10	0.653807	0.186956
RNU4-25P	0.986287	0.186839
NA	0.720994	0.186832
PPP1R26-AS1	0.158873	0.186828
AC093616.1	0.396784	0.186816
AC096772.1	-0.16969	0.186796
ABCA7	-0.13008	0.186758
TMSB10	-0.0445	0.186754
OR2B6	-0.41917	0.186736
GSTM1	1.269605	0.18672
RNU6-1011P	1.269605	0.18672
NA	1.269605	0.18672
AC020908.1	-0.71059	0.186712
LYRM4	0.068828	0.186709
SOCS6	0.079986	0.186697
IL1B	-0.31657	0.186638
CALCB	-0.124	0.18661
CPT2	-0.07427	0.186606
AC068533.3	0.775507	0.186596
VPS33A	-0.08466	0.186582
SNORA15B-1	0.82729	0.18654
ERCC3	-0.04838	0.186512
AC053503.2	0.645404	0.186509
ANKMY2	-0.09576	0.186504
ARID2	0.059386	0.186443
AC009812.4	-0.29875	0.186405
EIF4HP1	0.360919	0.186395
FCSK	0.098842	0.186391
VWA5A	0.348833	0.186337
NXN	0.079855	0.186276
PPFIA1	0.055118	0.186275
AC007610.2	0.244022	0.186247
OR1Q1	-1.37799	0.186226

MIR619	-1.37799	0.186226
FAM240C	-0.97457	0.186225
MEG9	-0.97457	0.186225
IGLV5-52	-1.2471	0.186216
NA	-1.2471	0.186216
AC010320.3	-1.2471	0.186216
NA	-0.06707	0.186165
PSMA2	0.10807	0.186164
AC011468.4	0.718118	0.186147
CLPB	0.055532	0.186136
WDR59	-0.06869	0.186121
CCDC130	0.072637	0.186078
ZDHHC6	0.062444	0.18607
TGFBR1	-0.06097	0.186047
RPL21	0.051856	0.186038
GRIN3B	-0.15983	0.186027
CPA4	-0.35988	0.186026
AC010761.7	-1.19973	0.185964
FAM57B	0.076241	0.185893
ZNF682	-0.12964	0.185854
KLF16	-0.07574	0.18582
NA	-0.90186	0.185815
UBOX5	-0.11601	0.185797
ZNF865	0.082739	0.185788
CAB39L	-0.10728	0.185787
POLL	0.078041	0.18578
MTCL1	0.074179	0.18573
LMCD1-AS1	0.44642	0.185696
L3HYPDH	-0.09404	0.18569
TMEM208	0.063524	0.185683
JRKL	-0.0749	0.185675
METTL24	0.4458	0.185635
AP002986.1	0.872397	0.185622
C9orf131	-0.73484	0.185621
MYH15	-0.13292	0.185617
PTGIS	-0.27668	0.185601
SEPT6	-0.0593	0.185593
AC124283.2	0.240841	0.18558
AC007347.1	1.261544	0.185522
GOLGA6L5P	-0.22887	0.185502
RF00019	-1.08937	0.185473
C1orf127	-0.95476	0.185447
SDCBP2	0.244135	0.185381
AL161781.2	-0.4881	0.185339
CEP128	-0.10495	0.185298
SHF	-0.06516	0.185294
SGK1	-0.07136	0.185273
BACH1-AS1	-0.56077	0.185259
NA	-0.50381	0.185208
AC005229.4	-0.15126	0.18519

AC011815.1	0.251425	0.185096
AC026803.2	0.549645	0.185093
TMEM141	0.108931	0.185076
B3GALT2	0.521353	0.185073
TWF1	0.050983	0.185069
FAM50B	-0.11893	0.185066
TTYH1	-0.27169	0.185045
KLHL26	0.127856	0.185036
FASTKD1	-0.07116	0.185029
AP001271.1	-1.24142	0.185016
AC016910.1	-0.63145	0.184983
TUBA4A	0.178405	0.184928
LNPEP	-0.08069	0.184908
RNF175	-0.12514	0.184905
MRPL45P2	0.184897	0.184865
AC004590.1	0.997667	0.18485
SLC25A1	-0.05859	0.184817
TPM3	-0.04889	0.184761
CEP57	0.055285	0.184752
AC138356.2	-0.26313	0.18474
CEP120	0.063856	0.184723
ZNF487	-0.20756	0.184712
IGFBP6	-0.11307	0.184679
NA	0.090789	0.184657
PTX3	-0.08902	0.184645
RPS12P26	0.77468	0.184626
NA	0.070552	0.184616
AC104561.2	0.582752	0.184597
CDK2	-0.0517	0.184562
PDPK2P	0.115426	0.184559
AC097381.1	-0.20365	0.184551
DMPK	-0.06462	0.184539
OTOGL	-1.49535	0.184537
LINC00482	-1.49535	0.184537
POU3F1	-1.49535	0.184537
RNU4-24P	-1.49535	0.184537
SNORA70G	-1.49535	0.184537
FABP5P1	-1.49535	0.184537
NA	-1.49535	0.184537
FBP1	0.168015	0.184525
MIEN1	0.069138	0.18452
AC093484.4	-0.30355	0.184511
SLC35A1	0.115121	0.184463
TAF4	-0.08987	0.184336
AP005210.1	-0.31979	0.184323
NFIA-AS2	-0.952	0.184267
TNRC6C-AS1	0.233636	0.184251
GINM1	-0.09149	0.184248
ATP5F1B	0.050857	0.184216
CCDC50	0.054354	0.184177

RPSAP1	0.684089	0.184176
RPL10AP2	0.454344	0.18417
TRMT10C	-0.07073	0.184137
FMC1	-0.31822	0.184133
IMP3	0.06004	0.184105
AC036108.1	0.252664	0.184088
TGIF2	-0.06723	0.184074
RPL30P2	-1.1023	0.184017
ARG1	-1.49241	0.183986
AL122020.1	-1.49241	0.183986
GET4	-0.09752	0.18395
SMG1P6	0.28256	0.183927
C15orf53	1.262958	0.183913
AC016597.1	-0.25543	0.183911
NA	-1.1101	0.183885
AC078881.1	-1.1101	0.183885
DLG2	-0.21468	0.183827
EBF3	0.987776	0.183761
ATP6V0A4	1.807901	0.183738
ADGB	1.807901	0.183738
TTC29	1.807901	0.183738
UTS2R	1.807901	0.183738
ANKRD62	1.807901	0.183738
C2CD4A	1.807901	0.183738
MIR342	1.807901	0.183738
RF00019	1.807901	0.183738
RF00019	1.807901	0.183738
RNU4-85P	1.807901	0.183738
RNU1-36P	1.807901	0.183738
NA	1.807901	0.183738
RNU6-540P	1.807901	0.183738
RF00191	1.807901	0.183738
EWSAT1	1.807901	0.183738
KRT8P40	1.807901	0.183738
AL109947.1	1.807901	0.183738
NA	1.807901	0.183738
AC092755.1	1.807901	0.183738
AL512604.1	1.807901	0.183738
AL158207.2	1.807901	0.183738
MTND5P19	1.807901	0.183738
AC097721.1	1.807901	0.183738
VN1R20P	1.807901	0.183738
AC006019.2	1.807901	0.183738
RBMS3-AS1	1.807901	0.183738
AC068446.1	1.807901	0.183738
NA	1.807901	0.183738
RPL7P15	1.807901	0.183738
RPS15P9	1.807901	0.183738
EEF1A1P25	1.807901	0.183738
AC015911.1	1.807901	0.183738

AC112719.2	1.807901	0.183738
TOX4P1	1.807901	0.183738
AC035140.1	1.807901	0.183738
AC024581.1	1.807901	0.183738
GAPDHP35	1.807901	0.183738
HOXC-AS3	1.807901	0.183738
RN7SKP272	1.807901	0.183738
AC020763.1	1.807901	0.183738
AC140479.4	1.807901	0.183738
AC090618.1	1.807901	0.183738
NA	1.807901	0.183738
NA	1.807901	0.183738
ELOCP33	1.807901	0.183738
AC126283.1	1.807901	0.183738
NOG	-0.74491	0.183718
SEC31A	-0.04342	0.183636
C1orf115	0.147339	0.183613
EOGT	-0.10569	0.183605
ZC4H2	-0.06587	0.183543
SRGAP1	0.086872	0.183526
DTNA	0.0697	0.183516
GPBP1L1	0.055396	0.183485
PVR	-0.06405	0.183467
STRN	-0.05965	0.183454
KNDC1	-0.30201	0.183443
ZNHIT6	-0.06446	0.183339
AL603839.2	-0.17487	0.183328
NA	-0.18828	0.183255
AC092171.3	-0.23662	0.183232
ZPLD1	0.72303	0.183207
AC016727.1	-0.07861	0.183146
THRB	-0.20957	0.183125
EVA1B	0.089854	0.183125
HLCS	0.066724	0.183123
TMEM163	-0.97818	0.183123
AGBL5	0.053115	0.183098
KRT8P46	-0.55859	0.18309
SHLD2P3	-0.35821	0.183052
TRIM69	0.079431	0.182999
EMD	0.060023	0.182978
AC010401.1	-0.15159	0.182976
CTNNA2	-0.06529	0.182961
ANKUB1	0.119365	0.182928
ILDR2	1.100094	0.182912
RNA5SP123	1.100094	0.182912
AC008267.2	-0.23333	0.182889
AC138150.2	-0.12624	0.182874
AL096701.3	-0.16809	0.182869
PEX13	-0.06654	0.182857
TBC1D5	-0.04808	0.182844

FEZ2	-0.06366	0.182796
SZRD1	-0.0502	0.182771
HMGN4	0.062595	0.182755
PACSIN2	-0.06917	0.18275
UBE2D1	0.075806	0.182712
IGHV3-42	0.461754	0.1827
BNIP1	-0.12398	0.18269
AL591135.1	-0.46378	0.182687
ZNF891	0.120505	0.18266
EIF2B1	-0.04666	0.182657
AL160004.1	0.386538	0.182648
ERI1	0.076896	0.182639
H2AFY	0.041109	0.182631
FOXP4-AS1	-0.74316	0.182628
MTUS2-AS1	0.532979	0.182622
PHLPP2	-0.07613	0.182603
NDUFAB1	0.05269	0.182584
ACTR3C	0.123521	0.182582
PRICKLE3	0.118871	0.182566
AC021876.1	-0.92982	0.182564
CXXC1P1	1.177312	0.182559
GID8	-0.05853	0.182516
INPP5K	0.065575	0.182511
ASPSCR1	-0.06783	0.18247
KLHL2	-0.08497	0.182439
TECR	-0.07277	0.182416
EIF4EBP3	-0.56486	0.182414
NA	0.586949	0.182383
EDA	-1.49554	0.182326
CLCN1	-1.49554	0.182326
MIR548L	-1.49554	0.182326
SNORA36B	-1.49554	0.182326
AC087235.2	-1.49554	0.182326
AC010643.1	-1.49554	0.182326
AL078605.1	-1.49554	0.182326
MROH7-TTC4	-1.49554	0.182326
AL139022.2	-1.49554	0.182326
PLEKHA2	-0.09479	0.182308
NA	-0.22841	0.182288
KIF14	-0.06744	0.182245
PTGFR	-0.30213	0.18224
AC243960.2	-0.54038	0.18224
PARPBP	0.084864	0.182206
WDR26	-0.04673	0.182202
AC104971.1	-0.33711	0.182191
C8orf88	-0.10063	0.182151
AP002748.1	-0.91954	0.182148
IL13RA2	-0.15791	0.182144
AL022341.2	0.113048	0.182135
B3GAT3	-0.05598	0.182127

LINC00887	0.754549	0.182113
ABHD13	-0.07186	0.182112
TNFRSF10A-AS1	-0.52617	0.182083
TMEM192	-0.06012	0.182003
AC008686.1	0.086183	0.181973
FBXW11P1	-0.46618	0.18196
SMARCE1P5	0.947719	0.181959
SNORA2C	-0.86816	0.18195
FAM45A	0.065418	0.181941
SLC16A10	-0.15782	0.181937
OPRL1	-0.1432	0.181928
PPP2R2B	0.079695	0.181925
FGL1	0.157183	0.181921
RNU6-1223P	0.708217	0.181913
QKI	0.05477	0.181876
ALAS1	0.084296	0.181836
AC113383.1	0.292286	0.18183
AC100861.2	0.884358	0.18181
RF00019	-1.49259	0.181776
LY6G6C	-1.49259	0.181776
AC008687.2	-1.49259	0.181776
AL022238.3	-1.49259	0.181776
TAF13	-0.08163	0.181711
HTR6	-0.35788	0.181705
SLC4A7	0.061878	0.181642
AP001628.1	0.500746	0.181618
VWF	0.459439	0.181556
AC011445.1	0.908448	0.181552
EXOSC4	0.054435	0.181529
AC231533.1	-0.13995	0.181525
BNIP3L	0.053862	0.181498
FPGT-TNNI3K	-0.56496	0.18149
LRFN3	-0.1148	0.181464
REST	0.050866	0.18145
NPM1P9	0.363152	0.181425
KCTD3	0.049121	0.181372
NA	-1.16817	0.181372
AC096746.1	1.090403	0.181312
NVL	0.06774	0.181299
BEND5	0.121009	0.181293
LDHAP4	-0.13405	0.18129
AC004967.1	0.119202	0.181282
GIMAP2	-0.50104	0.181276
POLR2G	-0.05858	0.181269
PCOLCE	-0.05577	0.181268
GDE1	0.054847	0.181266
SH3GLB1	-0.06665	0.181254
ARPC3	0.046268	0.181247
ARMC10	0.053025	0.181186
PCYT1B	0.088902	0.181186

RNU4-52P	-0.86611	0.181185
GAS8	-0.07241	0.181175
AL645929.1	0.117924	0.181134
DNAJC13	-0.05196	0.181132
NRIP2	-0.12971	0.18107
AC009185.1	0.394109	0.181031
CDR1	0.396114	0.180953
LPXN	-0.15645	0.180922
MCCD1P2	-1.22635	0.180915
AC020703.1	-1.22635	0.180915
VPS16	0.068189	0.180883
NA	-0.25131	0.180847
CR559946.1	0.504612	0.180835
GTPBP6	0.070617	0.180799
CYP3A7	0.618823	0.180751
RANBP3L	0.684955	0.180741
ST7L	0.074163	0.180716
ZMAT5	0.094386	0.180659
PTP4A3	0.11581	0.180637
METTL3	0.053614	0.180625
AC055764.1	0.171114	0.180616
ZNF287	0.099001	0.180601
INSYN2B	0.143151	0.18055
GOLGA8H	-0.30854	0.1805
LZTS1	-0.16572	0.1805
LRRC4C	0.37823	0.180464
RN7SL614P	1.476443	0.180458
MIR4648	1.476443	0.180458
ALDH1A2	-0.45017	0.180387
NAPSA	0.264131	0.180369
AC099568.2	-0.40947	0.180335
RPS27P29	0.731022	0.180334
AC005042.1	1.076412	0.180312
AL023284.4	0.240881	0.180301
AL590627.1	0.819926	0.180296
MEX3D	0.067821	0.18025
PAPPA2	-0.41514	0.180247
AL161757.2	0.206246	0.180241
B4GALT7	0.079021	0.180191
NA	0.139637	0.180184
SUGP2	-0.04772	0.180164
NPM1P19	1.150069	0.180148
TIE1	-1.77508	0.1801
GATA1	-1.77508	0.1801
MTNR1B	-1.77508	0.1801
HAVCR2	-1.77508	0.1801
GPR45	-1.77508	0.1801
C7orf69	-1.77508	0.1801
SLC25A48	-1.77508	0.1801
FAM81B	-1.77508	0.1801

CHODL	-1.77508	0.1801
MAGEA8	-1.77508	0.1801
AQP5	-1.77508	0.1801
TREML1	-1.77508	0.1801
ACTBL2	-1.77508	0.1801
PSAPL1	-1.77508	0.1801
SPEM1	-1.77508	0.1801
TP53TG3	-1.77508	0.1801
FIGLA	-1.77508	0.1801
ANKRD20A19P	-1.77508	0.1801
RF00019	-1.77508	0.1801
RNU5E-1	-1.77508	0.1801
RNU6-1283P	-1.77508	0.1801
AC005342.1	-1.77508	0.1801
NA	-1.77508	0.1801
GOLGA8O	-1.77508	0.1801
RF00019	-1.77508	0.1801
SNORD116-9	-1.77508	0.1801
RNU6-30P	-1.77508	0.1801
RNU6-851P	-1.77508	0.1801
MIR548C	-1.77508	0.1801
NA	-1.77508	0.1801
MIR10B	-1.77508	0.1801
RPL7AP9	-1.77508	0.1801
PGGT1BP1	-1.77508	0.1801
AL121952.1	-1.77508	0.1801
NA	-1.77508	0.1801
RF00019	-1.77508	0.1801
RN7SKP158	-1.77508	0.1801
RF00413	-1.77508	0.1801
AC090044.1	-1.77508	0.1801
GPC6-AS2	-1.77508	0.1801
HMGN1P24	-1.77508	0.1801
AL672291.1	-1.77508	0.1801
AL512590.1	-1.77508	0.1801
AC099788.1	-1.77508	0.1801
B3GNT2P1	-1.77508	0.1801
MEIS1-AS2	-1.77508	0.1801
AC093166.3	-1.77508	0.1801
AC111200.2	-1.77508	0.1801
LINC01516	-1.77508	0.1801
SETP10	-1.77508	0.1801
FAM157B	-1.77508	0.1801
MTND2P29	-1.77508	0.1801
AL357055.1	-1.77508	0.1801
AL391863.2	-1.77508	0.1801
AC011742.3	-1.77508	0.1801
AC130472.1	-1.77508	0.1801
AC116049.1	-1.77508	0.1801
C3orf84	-1.77508	0.1801

AL035420.2	-1.77508	0.1801
AC099566.1	-1.77508	0.1801
TUBB4BP3	-1.77508	0.1801
LINC00857	-1.77508	0.1801
RNU7-50P	-1.77508	0.1801
NA	-1.77508	0.1801
NA	-1.77508	0.1801
NA	-1.77508	0.1801
NA	-1.77508	0.1801
RNU7-170P	-1.77508	0.1801
HLA-DOB	-1.77508	0.1801
AL132838.2	-1.77508	0.1801
AC004917.1	-1.77508	0.1801
RF00156	-1.77508	0.1801
RNU6-1299P	-1.77508	0.1801
RNA5SP282	-1.77508	0.1801
RF00322	-1.77508	0.1801
SCARNA1	-1.77508	0.1801
RF00156	-1.77508	0.1801
AC084346.1	-1.77508	0.1801
MPPED2-AS1	-1.77508	0.1801
AC084859.1	-1.77508	0.1801
AC013549.2	-1.77508	0.1801
STRA6LP	-1.77508	0.1801
HSPD1P3	-1.77508	0.1801
AC010203.2	-1.77508	0.1801
LINC02156	-1.77508	0.1801
SALL4P7	-1.77508	0.1801
AL161669.1	-1.77508	0.1801
IDH2-DT	-1.77508	0.1801
AC099793.1	-1.77508	0.1801
AC126696.1	-1.77508	0.1801
PMF1-BGLAP	-1.77508	0.1801
AC133552.1	-1.77508	0.1801
AC145350.3	-1.77508	0.1801
AC087392.1	-1.77508	0.1801
MTND6P33	-1.77508	0.1801
AC113189.2	-1.77508	0.1801
NA	-1.77508	0.1801
NA	-1.77508	0.1801
AL627309.6	-1.77508	0.1801
NA	-1.77508	0.1801
AC110048.1	-1.77508	0.1801
AC018889.1	-1.77508	0.1801
AC098935.2	-1.77508	0.1801
NA	-1.77508	0.1801
AC091544.5	-1.77508	0.1801
AC008592.5	-1.77508	0.1801
AC022893.3	-1.77508	0.1801
AC020634.1	-1.77508	0.1801

UBL3	-0.0603	0.18009
AC011510.1	0.889587	0.180089
PRKCA-AS1	0.714505	0.18006
AL590666.2	0.496287	0.180022
AL121603.2	0.099504	0.180016
STX17	0.074328	0.179962
AC132812.1	-0.14151	0.179959
FUT4	0.150594	0.179943
ADCY10	-0.76042	0.179912
SERTM1	-0.76042	0.179912
C5	-0.09021	0.179874
MTCO3P29	1.024624	0.179867
AC068338.2	0.128421	0.179859
AL022331.1	-0.80385	0.179852
THAP2	-0.06444	0.179845
LINC01054	0.875789	0.179807
MIPEP	0.112159	0.179803
ABLIM2	0.151398	0.179761
AL365203.2	-0.1525	0.179728
ITFG1-AS1	-0.11637	0.179675
JMJD8	0.048442	0.179671
ADHFE1	-0.26887	0.179665
AC006480.2	0.236567	0.179661
PCDHAC1	-0.11801	0.179617
FABP5P7	0.636246	0.179599
AC008752.2	-0.80955	0.179582
TEX46	1.146273	0.179541
YBX3	-1.77042	0.179528
SLC26A3	-1.77042	0.179528
SLC17A3	-1.77042	0.179528
HORMAD1	-1.77042	0.179528
GPR17	-1.77042	0.179528
TMEM47	-1.77042	0.179528
C15orf48	-1.77042	0.179528
RNASE8	-1.77042	0.179528
DENND2C	-1.77042	0.179528
FOXR1	-1.77042	0.179528
C10orf71	-1.77042	0.179528
CCDC190	-1.77042	0.179528
AKAP14	-1.77042	0.179528
DIO3	-1.77042	0.179528
GPR52	-1.77042	0.179528
ERICH2	-1.77042	0.179528
H2AFZP1	-1.77042	0.179528
AL390728.2	-1.77042	0.179528
AL031599.1	-1.77042	0.179528
NPIPA3	-1.77042	0.179528
SHMT1P1	-1.77042	0.179528
AL596247.1	-1.77042	0.179528
HNRNPA3P4	-1.77042	0.179528

LINC01748	-1.77042	0.179528
NUTM2E	-1.77042	0.179528
CR391992.1	-1.77042	0.179528
PA2G4P6	-1.77042	0.179528
ALDH7A1P2	-1.77042	0.179528
AC007161.1	-1.77042	0.179528
ADAM21P1	-1.77042	0.179528
AL390961.2	-1.77042	0.179528
AL035458.1	-1.77042	0.179528
AC011284.1	-1.77042	0.179528
LINC00347	-1.77042	0.179528
AL354794.2	-1.77042	0.179528
AL034379.1	-1.77042	0.179528
NA	-1.77042	0.179528
RN7SL225P	-1.77042	0.179528
RN7SL587P	-1.77042	0.179528
HNRNPA1P24	-1.77042	0.179528
THAP12P2	-1.77042	0.179528
TERF1P3	-1.77042	0.179528
HMGB1P28	-1.77042	0.179528
RF00322	-1.77042	0.179528
NA	-1.77042	0.179528
NA	-1.77042	0.179528
AC068389.2	-1.77042	0.179528
AC025030.1	-1.77042	0.179528
AF107885.2	-1.77042	0.179528
AC040918.1	-1.77042	0.179528
HNRNPMP1	-1.77042	0.179528
AC015818.2	-1.77042	0.179528
AC099489.2	-1.77042	0.179528
MIR4783	-1.77042	0.179528
NA	-1.77042	0.179528
AC009137.2	-1.77042	0.179528
AC008543.3	-1.77042	0.179528
SRSF10P1	-1.77042	0.179528
AC020908.3	-1.77042	0.179528
AC022022.2	-1.77042	0.179528
NA	-1.77042	0.179528
AL365181.4	-1.77042	0.179528
TUBB1	0.200294	0.179436
AP2A1	0.054071	0.1794
FANCL	-0.07868	0.179398
AC079203.1	0.772005	0.179349
CEP162	0.087219	0.179299
NA	-0.76456	0.179269
ZMYM1	0.088701	0.179235
TAOK1	0.047402	0.179235
AC244453.3	0.503955	0.179178
FGF5	-0.13694	0.179159
NA	-0.10637	0.179119

MBL1P	0.221631	0.179092
ACSBG1	-0.07353	0.179077
CNIH1	-0.05672	0.179061
WRAP53	-0.06247	0.179033
ELAC2	-0.05319	0.179022
TCP11L1	-0.07386	0.178998
SGTA	0.047058	0.178969
TNFRSF17	-1.76574	0.178956
SPI1	-1.76574	0.178956
CCDC170	-1.76574	0.178956
IGLL1	-1.76574	0.178956
PTPN22	-1.76574	0.178956
IFNA21	-1.76574	0.178956
BCRP2	-1.76574	0.178956
LINC01106	-1.76574	0.178956
LINC02583	-1.76574	0.178956
NYX	-1.76574	0.178956
RPS2P4	-1.76574	0.178956
FAM177B	-1.76574	0.178956
RF00019	-1.76574	0.178956
SNORA68B	-1.76574	0.178956
RN7SKP237	-1.76574	0.178956
RNU6-7	-1.76574	0.178956
AL354919.1	-1.76574	0.178956
C1orf185	-1.76574	0.178956
RF00019	-1.76574	0.178956
RF00019	-1.76574	0.178956
NA	-1.76574	0.178956
RNY4P36	-1.76574	0.178956
PPP1R14BP2	-1.76574	0.178956
FTH1P12	-1.76574	0.178956
NA	-1.76574	0.178956
AC010931.1	-1.76574	0.178956
GRK6P1	-1.76574	0.178956
MIR887	-1.76574	0.178956
AL356057.1	-1.76574	0.178956
AL512378.1	-1.76574	0.178956
HNRNPA1P41	-1.76574	0.178956
NA	-1.76574	0.178956
NA	-1.76574	0.178956
AC083900.1	-1.76574	0.178956
AL023581.1	-1.76574	0.178956
RPL21P92	-1.76574	0.178956
IL1R1-AS1	-1.76574	0.178956
C1DP4	-1.76574	0.178956
UMLILO	-1.76574	0.178956
EEF1DP1	-1.76574	0.178956
NBPF21P	-1.76574	0.178956
LARP1BP1	-1.76574	0.178956
CEACAMP5	-1.76574	0.178956

AC010677.1	-1.76574	0.178956
EIF4A1P7	-1.76574	0.178956
AC110995.1	-1.76574	0.178956
C1DP2	-1.76574	0.178956
AL732372.1	-1.76574	0.178956
RAI1-AS1	-1.76574	0.178956
NA	-1.76574	0.178956
DDR1-DT	-1.76574	0.178956
TNFRSF14-AS1	-1.76574	0.178956
KRBOX1	-1.76574	0.178956
CBX5P1	-1.76574	0.178956
RN7SL704P	-1.76574	0.178956
MDFIC2	-1.76574	0.178956
RN7SL127P	-1.76574	0.178956
NA	-1.76574	0.178956
LAMTOR3P2	-1.76574	0.178956
GAPDHP70	-1.76574	0.178956
AC008723.1	-1.76574	0.178956
PSMC1P5	-1.76574	0.178956
AP004147.1	-1.76574	0.178956
AP001999.1	-1.76574	0.178956
RNU7-105P	-1.76574	0.178956
RNU7-183P	-1.76574	0.178956
RNU6-922P	-1.76574	0.178956
NRBF2P4	-1.76574	0.178956
AC027117.2	-1.76574	0.178956
LINC02551	-1.76574	0.178956
NA	-1.76574	0.178956
FTLP6	-1.76574	0.178956
AC010768.2	-1.76574	0.178956
AP003170.4	-1.76574	0.178956
AP003170.5	-1.76574	0.178956
AC044802.2	-1.76574	0.178956
AC015660.1	-1.76574	0.178956
AC013652.1	-1.76574	0.178956
AL136295.4	-1.76574	0.178956
AC009021.2	-1.76574	0.178956
MIR4694	-1.76574	0.178956
AC106037.2	-1.76574	0.178956
AC060766.1	-1.76574	0.178956
NA	-1.76574	0.178956
AF038458.2	-1.76574	0.178956
NA	-1.76574	0.178956
AC018695.2	-1.76574	0.178956
AC130352.1	-1.76574	0.178956
MTRNR2L13	-1.76574	0.178956
AC072026.1	-1.76574	0.178956
ZBTB42	-0.15022	0.178941
AL671277.2	-0.23991	0.178867
TPTE2P1	-0.66111	0.178826

AL157838.1	-0.42042	0.178822
ST6GALNAC2	-0.05212	0.17882
NDUFA3P1	0.563301	0.178817
CEP192	-0.06513	0.178808
AC025171.2	-0.59542	0.178806
STK19	0.068898	0.178768
C11orf45	-0.30776	0.178764
NA	-0.54902	0.178762
ACAD11	-0.10127	0.178746
NR2F6	0.052719	0.178734
PAX7	-1.44744	0.178725
AC006001.1	-1.44744	0.178725
LINC01717	-1.44744	0.178725
SNORD62A	-1.44744	0.178725
AC108112.1	-1.44744	0.178725
AL390816.1	-1.44744	0.178725
MRPS21P6	1.067468	0.178721
AC010173.1	1.067468	0.178721
TDRD3	0.080456	0.178653
SELENOP	0.094452	0.178612
AP000873.3	-0.26745	0.178609
ALDH1L2	0.146065	0.178605
ZNF14	0.13443	0.178592
HSP90B2P	-0.7285	0.178584
HMGN3	0.056986	0.178572
CERS4	0.108141	0.178558
FPGS	0.0635	0.178555
AC003002.2	0.701858	0.17852
GLOD4	-0.05404	0.178493
AC022509.2	-0.6003	0.178484
SLC35D2	-0.07666	0.17848
ZBED9	0.096207	0.178476
PTPN7	0.835017	0.178472
PLEK2	-0.21911	0.178439
GAK	-0.0618	0.178434
NA	-0.05727	0.178411
PMS2P1	-0.07536	0.178403
PWWP2B	0.101429	0.178394
CDV3	0.049224	0.178353
BFSP2	-1.54393	0.178278
TMEM72-AS1	-1.54393	0.178278
P4HA3-AS1	-1.54393	0.178278
AC093827.3	-1.54393	0.178278
AC011481.1	0.508811	0.178255
KLHL12	0.057013	0.178247
SLC35B4	-0.04649	0.178232
RNU6-642P	1.476349	0.178225
RF00019	1.476349	0.178225
AL121900.1	1.476349	0.178225
AC006160.1	1.476349	0.178225

AC004233.1	1.476349	0.178225
AC013553.2	1.476349	0.178225
KLF10	-0.07643	0.178151
EIF1B	0.060255	0.178136
RNASEH2C	0.045185	0.178131
MBNL1	0.068301	0.178105
AL135838.1	1.476343	0.178087
ZNF200	-0.08225	0.178073
C3orf35	-0.59924	0.17804
PRPF6	-0.04818	0.178039
TPR	0.047476	0.178001
AC002550.1	0.072859	0.177981
SNHG7	0.059577	0.177949
NA	0.677903	0.177932
AC106707.1	-0.16537	0.17785
BICRAL	-0.06939	0.1778
CYB5A	0.0791	0.177776
MIR93	-0.38739	0.177773
AC129492.2	0.369028	0.17777
ZNF212	-0.08195	0.17776
ZNF443	0.124351	0.177751
DYNC1LI2	-0.04399	0.17772
AC015853.2	0.794588	0.177705
DNAL1	-0.06579	0.177704
PHOX2B	0.050613	0.177637
ZNF100	0.093611	0.17762
MAGEB1	-1.44141	0.177614
AC010157.1	0.536774	0.177595
MED25	0.06299	0.177583
AC106791.1	-0.24977	0.177565
ASIC3	0.115551	0.177557
DCUN1D5	-0.0773	0.177544
ETFRF1	0.078463	0.177541
MDGA1	-0.13794	0.17753
XRCC6	0.044105	0.177516
SBSPON	0.244145	0.177505
FAM21FP	0.257917	0.17749
AL645608.6	-0.25078	0.177477
SYT15	0.224491	0.177464
RPL23AP65	0.624952	0.177447
NA	-1.00385	0.177441
CFAP77	0.233865	0.177387
AC138207.8	0.8491	0.177387
NAPEPLD	-0.06383	0.17734
FBXO5	0.067284	0.177291
HMG20A	0.047324	0.177263
AC138904.1	0.112175	0.177221
DDX11L2	-0.18067	0.177218
AC007956.1	0.660199	0.17721
INF2	-0.07039	0.17718

ABCA11P	-0.18277	0.177146
SEC61A2	0.065411	0.177143
C2orf42	-0.10844	0.177093
CPXM1	0.059222	0.177048
UBE2Q2	0.064448	0.177029
AL513365.2	-0.48545	0.17695
DNTTIP1	0.066868	0.176924
RAPGEF5	0.051679	0.176883
AL034397.1	1.219988	0.176861
RAB6D	1.219988	0.176861
FOXP3	0.32303	0.17684
HNRNPA3P12	0.708698	0.176831
AP000777.3	-0.13607	0.176828
AC090877.1	-0.93925	0.176755
LINC01588	0.63616	0.176743
TP53BP1	-0.0454	0.176701
CMTM2	1.45658	0.176697
OR11N1P	1.45658	0.176697
NA	1.45658	0.176697
KAT6B	-0.06198	0.176675
SRPK1	0.052291	0.176673
SCN1A-AS1	0.095018	0.176636
CD4	0.59048	0.176606
ANKRD39	0.139347	0.176601
NA	-0.62557	0.176588
NUTM2A	0.21779	0.176563
ZNF474	-0.91427	0.176555
NA	1.068267	0.176551
AC092032.1	-0.57727	0.176546
NA	-1.07509	0.176501
C21orf58	-0.08038	0.176474
LINC01515	-0.11725	0.176406
LINC01664	0.578604	0.176399
AL390728.6	0.086589	0.176391
ARAP1-AS1	0.153809	0.176386
PM20D2	-0.06167	0.17637
LRRN4	0.764693	0.176274
AC005618.1	0.587526	0.176253
AC091685.2	0.506791	0.176246
ZNF850	0.074576	0.176234
KLHL29	-0.09098	0.176232
SH3BP4	-0.06661	0.176212
ZNF133	0.064611	0.176194
NA	-0.36462	0.176163
AC098829.1	0.252155	0.176062
COX15	0.049507	0.176053
TTC24	-0.80774	0.176028
NRGN	-0.14294	0.176018
UBAC2-AS1	0.25802	0.175994
TAS2R15P	-0.48947	0.175968

LINC02352	0.580881	0.175912
COX16	-0.09071	0.175896
AL024497.2	-0.66399	0.175879
AC112484.3	0.272094	0.175863
TCAF1P1	0.102556	0.175834
STRBP	0.063037	0.175813
AC090236.1	-0.28248	0.175735
ZIK1	-0.11276	0.175724
AC009021.1	0.525637	0.175698
AL390039.1	1.211858	0.175668
AC019186.1	1.211858	0.175668
AC021028.1	-1.44748	0.175638
AC005828.1	-1.44748	0.175638
AC027097.2	-1.44748	0.175638
AL132780.1	-0.13614	0.175629
AL031709.1	-0.16853	0.175607
CLGN	-0.07224	0.175606
ANAPC15	0.075941	0.175567
NDE1	-0.06818	0.175556
STX6	0.055229	0.175531
IGBP1-AS1	0.636353	0.175527
NA	-0.07703	0.175515
XPR1	-0.06344	0.17551
AC008966.2	-0.8795	0.175505
KIDINS220	0.04363	0.175501
TBC1D8	0.089736	0.175475
AC027796.2	0.673577	0.175433
AKT3	-0.04646	0.175387
NOVA2	-0.09494	0.175268
AC036214.1	-0.49655	0.17524
GOLGA6L9	-1.54434	0.175239
HSP90AA5P	-1.54434	0.175239
RN7SL477P	-1.54434	0.175239
ME1	-0.06965	0.175233
AC015922.2	-0.18478	0.175169
AL450326.1	-0.41733	0.175165
CARM1	-0.05432	0.175163
DOCK4	-0.06119	0.175097
C19orf18	-0.85048	0.175082
AP001107.8	-0.14758	0.175006
HEXD	0.061185	0.174993
STMN1	0.045291	0.17498
CCL11	1.071373	0.174976
LRRD1	1.071373	0.174976
QSOX1	-0.06176	0.174965
TIPIN	-0.08216	0.174904
GNAZ	-0.0778	0.174822
SMARCD1	0.05599	0.174815
ZKSCAN7-AS1	-0.20397	0.174801
MRTFB	-0.05944	0.174791

GALM	-0.08059	0.174779
CCT8	-0.05306	0.174766
CD2AP	-0.07718	0.174737
MAZ	0.051254	0.174733
RAB26	-0.12014	0.174716
RNU4-92P	0.43266	0.1747
BRD4	-0.07692	0.174672
NAT1	0.179022	0.174665
NA	-0.40898	0.174637
BRD9	-0.05121	0.174601
BDP1	0.055158	0.174594
AC002524.1	1.257859	0.174561
ZNF367	0.081752	0.174553
LINC01270	0.102482	0.174547
DNAH8	-0.75818	0.174535
TUBBP5	-1.44141	0.174533
KHSRPP1	-1.44141	0.174533
NA	-1.44141	0.174533
AL355483.2	-1.44141	0.174533
NA	-1.44141	0.174533
RN7SL236P	-1.44141	0.174533
AC134775.1	-1.44141	0.174533
NA	0.292418	0.174532
AC147651.4	-0.35948	0.174531
AC144450.1	1.199332	0.17452
BRD7P4	1.199332	0.17452
AP000757.2	1.199332	0.17452
LINC02035	0.105496	0.174475
AC092535.4	-0.63405	0.174472
FAM216B	1.456359	0.174469
GLRA4	1.456359	0.174469
RNU6-853P	1.456359	0.174469
LINC02528	1.456359	0.174469
ZNF823	0.121999	0.174464
NA	1.133463	0.174458
MTCO2P15	1.133463	0.174458
PPP2R5D	-0.05277	0.174446
MOSPD1	0.10284	0.174445
C1QTNF2	0.893102	0.174431
ZNF791	0.06206	0.174358
AC008514.1	-1.06536	0.174351
NA	-1.06536	0.174351
PRPF40A	0.055293	0.174344
MTCH2	-0.05238	0.174334
DUX4L9	1.456346	0.174331
TXNP6	1.456346	0.174331
AC104073.2	1.456346	0.174331
NA	-0.83431	0.174299
RN7SL861P	-0.83431	0.174299
MT-TW	0.292818	0.174284

EFCAB10	0.077618	0.174251
RPP25	-0.06612	0.174242
AC005041.1	-0.09097	0.174242
CXXC1	-0.05336	0.174241
AL513523.4	-0.14327	0.174225
SNORD4A	-0.37355	0.174215
NEURL4	-0.07802	0.174208
AC138969.1	-0.33107	0.174188
NA	-0.54064	0.174158
TNMD	1.72937	0.174124
TRPC7	1.72937	0.174124
HCK	1.72937	0.174124
TPSG1	1.72937	0.174124
PADI2	1.72937	0.174124
BHMT2	1.72937	0.174124
GPR6	1.72937	0.174124
FRMPD3	1.72937	0.174124
POU4F2	1.72937	0.174124
SLITRK5	1.72937	0.174124
MUC15	1.72937	0.174124
HSPB2	1.72937	0.174124
HSF5	1.72937	0.174124
ODF3	1.72937	0.174124
AC068473.1	1.72937	0.174124
EXOC5P1	1.72937	0.174124
HLA-V	1.72937	0.174124
ODF3L2	1.72937	0.174124
TNFRSF18	1.72937	0.174124
CRYBA4	1.72937	0.174124
SMIM11P1	1.72937	0.174124
RNU6-1309P	1.72937	0.174124
RNU6-598P	1.72937	0.174124
RNA5SP493	1.72937	0.174124
IGFL4	1.72937	0.174124
AC105345.1	1.72937	0.174124
RF00019	1.72937	0.174124
RF00012	1.72937	0.174124
SNORD116-13	1.72937	0.174124
MIR633	1.72937	0.174124
MIR181C	1.72937	0.174124
MIR124-2	1.72937	0.174124
SNORD41	1.72937	0.174124
AL731733.1	1.72937	0.174124
NA	1.72937	0.174124
RNU4-26P	1.72937	0.174124
RN7SKP235	1.72937	0.174124
SAMSN1-AS1	1.72937	0.174124
DIP2A-IT1	1.72937	0.174124
AC110602.1	1.72937	0.174124
MTCO3P19	1.72937	0.174124

BTF3P4	1.72937	0.174124
BX571846.1	1.72937	0.174124
AC007237.1	1.72937	0.174124
NOTCH2P1	1.72937	0.174124
FABP7P1	1.72937	0.174124
AL359644.1	1.72937	0.174124
NA	1.72937	0.174124
LINC01649	1.72937	0.174124
AC011995.1	1.72937	0.174124
AL357552.2	1.72937	0.174124
LIMS1-AS1	1.72937	0.174124
ELOCP20	1.72937	0.174124
AC006355.1	1.72937	0.174124
NA	1.72937	0.174124
AC123900.1	1.72937	0.174124
AL121829.1	1.72937	0.174124
BX284668.3	1.72937	0.174124
RPL4P3	1.72937	0.174124
AC007349.2	1.72937	0.174124
NMD3P2	1.72937	0.174124
Z97180.1	1.72937	0.174124
AC092802.3	1.72937	0.174124
AL031773.1	1.72937	0.174124
AC012370.1	1.72937	0.174124
LINC01251	1.72937	0.174124
NAP1L4P3	1.72937	0.174124
FYTTD1P1	1.72937	0.174124
AL355994.4	1.72937	0.174124
AL358176.5	1.72937	0.174124
AC009495.3	1.72937	0.174124
AL139156.3	1.72937	0.174124
AL035634.1	1.72937	0.174124
NA	1.72937	0.174124
CAMTA1-IT1	1.72937	0.174124
LINC01856	1.72937	0.174124
AC067940.1	1.72937	0.174124
NA	1.72937	0.174124
RNA5SP311	1.72937	0.174124
RF00019	1.72937	0.174124
NA	1.72937	0.174124
NA	1.72937	0.174124
SNORA84	1.72937	0.174124
AC092958.1	1.72937	0.174124
RN7SL116P	1.72937	0.174124
NA	1.72937	0.174124
LINC01324	1.72937	0.174124
AC104653.2	1.72937	0.174124
AC026316.3	1.72937	0.174124
LINC02265	1.72937	0.174124
AK3P2	1.72937	0.174124

ARPC4-TTLL3	1.72937	0.174124
AP001363.1	1.72937	0.174124
AC146944.2	1.72937	0.174124
NA	1.72937	0.174124
PCAT4	1.72937	0.174124
RNU6-863P	1.72937	0.174124
NA	1.72937	0.174124
NA	1.72937	0.174124
RNA5SP473	1.72937	0.174124
RNU6-902P	1.72937	0.174124
NA	1.72937	0.174124
AC008708.1	1.72937	0.174124
AC027312.1	1.72937	0.174124
AP003469.3	1.72937	0.174124
AC087854.2	1.72937	0.174124
AL355432.1	1.72937	0.174124
AP003086.3	1.72937	0.174124
AC120036.4	1.72937	0.174124
AC108516.2	1.72937	0.174124
NA	1.72937	0.174124
AP001363.2	1.72937	0.174124
AC126615.2	1.72937	0.174124
AC009320.1	1.72937	0.174124
AF123462.1	1.72937	0.174124
SUB1P2	1.72937	0.174124
HNRNPA1P71	1.72937	0.174124
AC113208.2	1.72937	0.174124
EIF4BP5	1.72937	0.174124
AC011939.2	1.72937	0.174124
AC007151.1	1.72937	0.174124
NA	1.72937	0.174124
AC007861.1	1.72937	0.174124
RYKP1	1.72937	0.174124
NA	1.72937	0.174124
MIR4729	1.72937	0.174124
RN7SL753P	1.72937	0.174124
AC090616.3	1.72937	0.174124
NA	1.72937	0.174124
ALOX12P1	1.72937	0.174124
AC079336.3	1.72937	0.174124
MIR4782	1.72937	0.174124
NA	1.72937	0.174124
NA	1.72937	0.174124
NA	1.72937	0.174124
NA	1.72937	0.174124
NA	1.72937	0.174124
NA	1.72937	0.174124
AC020907.3	1.72937	0.174124
AL365205.3	1.72937	0.174124
AC110792.2	1.72937	0.174124

SCGB2B3P	1.72937	0.174124
MKI67P1	1.72937	0.174124
AC104785.1	1.72937	0.174124
AC107031.1	1.72937	0.174124
HMGB1P50	1.72937	0.174124
RNU6-87P	1.72937	0.174124
AC112250.2	1.72937	0.174124
ABCE1	0.047895	0.174121
KLKB1	-0.5551	0.174116
GPR89A	0.099899	0.1741
AC006116.8	0.350924	0.174093
MTMR6	0.062039	0.174028
NOXRED1	-0.24243	0.174019
LINC02175	0.318274	0.173983
FAM21EP	0.435136	0.173953
TMEM237	0.051607	0.17394
RPL4P6	1.19537	0.173933
PCDHA10	1.19537	0.173933
AC124947.1	1.19537	0.173933
ATP13A3	0.049083	0.173905
TAFA2	-0.59438	0.173877
RN7SKP271	-0.71443	0.173871
HSD17B1	0.059168	0.173871
ATP5MPL	0.061673	0.17383
AC090739.1	-0.25563	0.173802
AC025430.1	-0.43675	0.173756
ZMYND10	-0.15795	0.173706
MBD4	0.06207	0.173698
CLEC4F	0.429509	0.173695
RXRB	-0.05028	0.173597
SNORA33	0.212797	0.173583
APBA1	-0.11449	0.173541
ST8SIA3	-0.06416	0.17352
HIPK1	-0.05567	0.173503
PPP4R1	0.04942	0.173486
CARMIL2	-0.08556	0.173486
CPLANE2	-0.15911	0.173484
SLC47A1P2	-1.4475	0.17345
EIF2S2P3	-1.4475	0.17345
AC245052.7	-1.4475	0.17345
SAFB2	-0.05005	0.173429
LINC02560	0.428706	0.173425
NUMA1	-0.049	0.173419
JARID2-AS1	-0.95205	0.1734
BACE2	-0.5716	0.173336
PTPN3	0.103417	0.173288
NA	-0.62097	0.173287
LINC02137	0.957761	0.173279
CFAP53	0.281524	0.173258
TIMM8B	0.087727	0.173244

CSTF2T	-0.05061	0.173243
TDH	-0.25882	0.173211
PRB2	-1.54461	0.173136
SCARNA20	-1.54461	0.173136
MYO7A	0.34117	0.173134
NA	-0.41714	0.173059
SRPX2	0.426147	0.173052
LRRC1	-0.1509	0.173038
RBM15	0.073796	0.173004
PCED1B	-0.35367	0.172999
AL356966.1	0.595494	0.172978
SOWAHC	0.091024	0.172905
NA	-0.63719	0.172905
AC021088.1	-0.17705	0.172901
NA	-0.53247	0.172882
MIR4271	0.269437	0.172879
NXF1	-0.05	0.172868
PDLIM3	0.145883	0.172796
NDUFA4	0.05347	0.172726
TRMT12	0.07006	0.172725
DNAAF5	-0.0449	0.172681
ZACN	-0.04676	0.17268
ZNF658B	0.459476	0.172665
KREMEN2	-0.15647	0.172662
COLQ	0.186691	0.172648
CA9	0.523782	0.172633
RPL7P23	0.581676	0.172629
FRMPD1	0.22492	0.172587
AC022150.3	0.502432	0.172574
CTRL	-0.13848	0.172562
AC009244.1	-0.29571	0.172561
FAM186A	0.643313	0.172537
CAPNS2	-0.98118	0.17252
AC106820.4	0.200775	0.17249
DNAJB7	-0.17431	0.17248
TEF	0.164448	0.172479
FBXO34	0.062357	0.172475
DIS3L2	-0.07036	0.172371
MROH2A	-1.44141	0.172348
NA	-1.44141	0.172348
NPIPA9	-1.44141	0.172348
LINC00940	-1.44141	0.172348
AC108210.1	-1.44141	0.172348
AC007406.4	-1.44141	0.172348
AC046168.1	-1.44141	0.172348
ZCCHC24	0.080545	0.172336
ZFAND2A	0.084339	0.172309
AGRP	0.406034	0.172269
AC090198.1	-0.11029	0.172266
AC092368.3	0.237855	0.172197

NDUFA9	0.10045	0.172131
TENM3-AS1	-0.1237	0.172121
RRP15	-0.05264	0.172107
MAVS	-0.06976	0.172065
ARTN	0.483394	0.172045
VPS50	0.066624	0.172004
METTL16	-0.05952	0.171971
AC098848.1	-0.41794	0.171953
MYCBP2-AS1	0.099205	0.171947
AL589990.1	0.441321	0.171922
CLASRP	-0.05598	0.171922
ZNF436	-0.06042	0.171918
NA	0.65282	0.171906
TRIM16	-0.07888	0.171903
PHF7	0.140111	0.1719
AL512306.2	0.78002	0.171894
ARID5A	0.175139	0.171857
AC104135.1	-0.64194	0.171854
SLC49A3	0.176551	0.171829
NCBP3	-0.05059	0.171818
PPP2R2D	-0.06052	0.171775
AF131215.4	0.456207	0.171719
NBPF2P	-0.20577	0.171695
OPTN	0.06449	0.171653
CEP126	-0.13484	0.171627
AC006033.2	-0.84453	0.171615
AC137630.4	-0.84453	0.171615
EFHC2	-0.38404	0.171603
AC004233.2	0.768431	0.1716
GCLC	0.054615	0.171596
BSCL2	-0.14646	0.17158
MMP25-AS1	0.148589	0.171579
WSCD2	0.296254	0.171575
FSIP2	-0.47081	0.171573
ZDHHC15	0.107963	0.171552
BEND3	-0.0964	0.171547
MAIP1	-0.08782	0.17148
NA	0.484935	0.171454
ZNF790	-0.10423	0.171443
AL161756.2	0.776615	0.171412
POLM	-0.09892	0.171403
SNORA60	-0.40375	0.171364
NCKIPSD	-0.0739	0.171359
CAP2	0.132178	0.171354
RWDD2B	-0.07335	0.17132
HNRNPA1P7	0.131804	0.171298
UBE2S	0.053884	0.171296
ZGLP1	-0.27705	0.171218
MED15P1	-0.4799	0.171182
CYCS	0.060659	0.171132

SACM1L	0.055726	0.171127
AC010306.1	1.035231	0.171107
AC004156.2	-0.35824	0.171081
C1GALT1P1	0.574357	0.171047
NA	0.470111	0.171046
AC073109.1	-0.59061	0.17104
AC083843.2	1.099489	0.171029
AGTR1	0.126945	0.170981
TXNDC11	0.082562	0.170976
RBBP5	-0.05224	0.17094
PRR26	0.8363	0.17092
RPS27AP11	-0.9658	0.170888
NA	-0.9658	0.170888
IQCC	-0.12439	0.170868
SFPQ	-0.04015	0.170851
BDNF-AS	-0.19337	0.17085
ZNF217	-0.05848	0.170849
AC027627.1	-0.7553	0.170832
ITGB1-DT	0.429327	0.170823
HSPB7	-0.12015	0.170789
ME2	-0.05888	0.170776
FSTL4	0.435964	0.170775
ARFIP2	0.049283	0.170691
NA	-0.04631	0.170654
E2F2	-0.07748	0.170653
RAD21-AS1	-0.09661	0.170649
LNCOG	-0.28881	0.170609
BUD23	0.045979	0.170607
FBXW5	-0.05768	0.170595
AC092954.1	-0.65647	0.170586
TNRC18P1	-0.59703	0.170514
NADK	-0.06198	0.17047
AC011676.1	0.94944	0.17047
AC016168.1	0.94944	0.17047
CALHM5	-0.32848	0.170434
HSPB1	0.046288	0.17041
IQCH	0.163269	0.170385
AGBL4-IT1	1.272985	0.17038
AC012349.1	1.272985	0.17038
AC018607.1	1.272985	0.17038
AC007952.6	1.272985	0.17038
ABI1P1	1.272985	0.17038
KLF6	0.084224	0.170279
AP001324.1	0.290894	0.170247
LINC01679	0.819242	0.170243
AKT2	0.050724	0.170139
AL355355.2	1.186033	0.170034
AC021171.1	1.186033	0.170034
TYMSOS	0.064573	0.169995
ZNF761	0.091152	0.169953

AC007608.3	-0.66922	0.169949
BBS1	0.213936	0.169931
ZNF326	-0.05066	0.169917
CPNE3	0.05666	0.169915
SEPT2	0.041811	0.169906
PCDHGA1	0.362787	0.169852
LYPD6	-0.09367	0.169845
PRMT8	-0.75576	0.16984
CLUHP3	0.092863	0.169824
MAF1	0.05218	0.169809
EMC8	0.061996	0.169757
AL512408.1	0.315034	0.169731
KIAA0355	-0.05898	0.169704
PPARD	-0.06087	0.16969
AC093423.2	0.976714	0.169668
DND1	-0.0775	0.169655
CTNNA1	0.041597	0.169625
RPL12P6	1.116552	0.169584
STX1A	-0.07027	0.169509
KLRA1P	-0.13456	0.169486
DRG2	-0.06881	0.169484
SH3BGRL2	-0.05675	0.169455
POLD4	-0.19299	0.169452
LINC01771	-1.15337	0.169439
IGSF8	0.075879	0.169422
GGT5	0.310946	0.169413
AC134312.4	-0.97112	0.169403
AL391994.1	0.547748	0.169359
MC1R	-0.16521	0.169314
AL022328.1	-0.23564	0.169306
PPP2R2C	-0.11677	0.169237
NA	1.264503	0.169234
AC239798.2	-1.39523	0.169223
NA	-0.10456	0.169221
MAP3K20	-0.06933	0.16921
SSC4D	0.160223	0.169195
SLC29A3	-0.09989	0.169186
ELMO1	-0.04396	0.169184
GLDC	0.101781	0.169169
ATF5	0.067767	0.169162
NA	-0.15577	0.169141
AL390879.1	-0.2636	0.169124
KIF19	0.727729	0.169083
RAET1G	-0.47142	0.169056
ELOVL6	-0.06032	0.169037
ALS2	0.059358	0.169034
AC013565.1	-0.56001	0.16901
TRABD	-0.06646	0.168971
FGF22	-0.25806	0.168906
NA	-0.18868	0.168897

CFAP44-AS1	0.270332	0.168887
PPHLN1	-0.04541	0.168881
AC019131.1	-0.95362	0.168872
SAC3D1	-0.07732	0.168812
NA	0.220529	0.168788
GRM1	0.407574	0.168787
FYCO1	-0.08496	0.168765
BRD1	-0.06079	0.168756
PPOX	0.057228	0.168702
LSM6	-0.08265	0.168701
NCSTN	-0.04446	0.16869
SENP8	-0.16655	0.168684
PRDM11	-0.14446	0.168677
HNRNPC	0.044776	0.168668
AC006581.1	-1.39211	0.168667
AP000553.3	-1.39211	0.168667
TOMM40L	-0.06819	0.168589
TOP3BP1	1.172039	0.168563
SUMO2P6	1.172039	0.168563
C16orf87	-0.07691	0.168525
KIAA1671	-0.08703	0.168515
SPA17	-0.1025	0.168508
FN3KRP	-0.05181	0.168438
SHOC1	-0.86677	0.168432
AC109460.1	0.117878	0.168401
EXOSC9	-0.06455	0.168401
NOL8	0.057528	0.168389
FAM136A	-0.05379	0.168383
IP6K1	-0.0583	0.168382
IGBP1-AS2	0.108914	0.168326
LMAN2	0.049942	0.168294
AC068620.1	0.405243	0.168292
ACTR5	0.076472	0.168289
YWHAQ	0.037255	0.168285
GPR160	0.385365	0.168271
ZNF561-AS1	0.112022	0.168269
SULT6B1	-0.24373	0.168264
AP1B1	-0.05552	0.168228
DNAJC24	0.078151	0.168214
THBS2	-0.3182	0.168194
NA	0.139614	0.168108
WASF4P	1.50616	0.168079
RF00017	1.50616	0.168079
AL023284.3	1.50616	0.168079
CATIP-AS1	1.50616	0.168079
RPL21P134	1.50616	0.168079
MED28P8	1.50616	0.168079
RPL21P116	1.50616	0.168079
AC008700.1	1.50616	0.168079
AC104027.1	1.50616	0.168079

NA	1.50616	0.168079
LINC02073	1.50616	0.168079
MCF2L-AS1	0.282974	0.168078
AL118556.1	0.139016	0.168073
PROX2	-0.2433	0.168053
NA	-0.55776	0.168048
TDRD9	0.958844	0.168027
RNF114	-0.04942	0.167963
SNRPGP4	-0.88041	0.167948
FTSJ3	-0.03732	0.167935
AL139807.1	-0.48299	0.167896
LDOC1	0.119847	0.167886
COX8C	1.169272	0.167865
AC010598.1	1.169272	0.167865
ACRV1	0.457302	0.167858
ANXA9	0.136424	0.16785
AKR1A1	0.050039	0.167848
VIRMA	-0.05063	0.167827
EML2-AS1	0.248153	0.167809
CYP1A1	-0.25035	0.167786
TASOR	0.050797	0.167784
POU3F2	0.20939	0.167753
UBE2R2	-0.05157	0.167717
MCRIP1	-0.08286	0.167671
FAM66A	0.490694	0.167651
AC025277.1	-0.77675	0.167608
CEMIP	-0.08949	0.16759
AC008915.2	-0.12836	0.167569
AC107464.3	-0.24818	0.167569
MTMR3	-0.06069	0.167526
SAMD8	-0.05382	0.167501
TTC38	-0.06087	0.167484
FUBP3	0.046325	0.167469
GALNT1	-0.04848	0.167421
OCM	1.404907	0.167413
NA	1.404907	0.167413
FAM53B-AS1	1.404907	0.167413
AC092756.1	1.404907	0.167413
AL353593.1	0.232481	0.167398
AC104964.1	-0.39126	0.167387
Z98749.1	1.163913	0.167385
IL12RB1	1.165319	0.16729
AL662844.3	1.165319	0.16729
FBXO30	-0.05241	0.167287
NA	-1.23882	0.167287
ZRANB2-AS1	0.073086	0.167214
TBCC	0.06863	0.167213
FAAP24	-0.12358	0.167206
GNGT2	-0.70206	0.167108
MPDZ	0.046444	0.167086

SNAPC5	-0.08734	0.167067
KDM2A	-0.05402	0.16705
MPZL3	0.552115	0.167029
SLC38A7	0.075436	0.167015
AC023906.4	0.13979	0.167008
SMIM29	0.090105	0.166985
TRAPPC6B	0.061224	0.166982
AL355075.2	0.194249	0.166975
AL008718.3	-0.73601	0.166974
RNPC3	-0.08446	0.166958
Z82214.1	0.355667	0.166957
BAALC-AS1	-0.29882	0.166872
AL137024.1	-0.52385	0.166864
ADCY6	-0.06353	0.166862
AC010973.1	-0.2516	0.166859
LINC01914	-0.6181	0.166728
LYRM2	-0.05161	0.166586
AOC1	1.094626	0.166542
AL359538.1	-0.80918	0.166525
M6PR	0.042692	0.166521
PIRT	0.444974	0.166518
STEAP4	-0.67189	0.166472
RPS3AP25	1.021387	0.166441
RBM5	0.047608	0.166435
MTRR	-0.06449	0.166389
SUMO2P3	-0.9972	0.166383
AC091057.3	-0.56824	0.166377
MTUS2	0.245995	0.166343
RPL31P52	-0.5079	0.166337
DBH-AS1	-0.07815	0.166307
FBLN7	-0.09712	0.166307
WLS	-0.0642	0.166306
PPP1CB	0.050222	0.166274
ALDH5A1	0.088389	0.166273
MIR570	-0.26904	0.166262
IL2RB	-0.29998	0.166246
TPM3P9	-0.09287	0.166215
RPL4P5	0.542748	0.166214
CYSTM1	-0.07712	0.166192
STXBP1	-0.05466	0.166188
THA1P	0.290706	0.16617
POU4F3	-1.39498	0.166169
LINC01402	-1.39498	0.166169
AC004584.2	-1.39498	0.166169
LINC00951	0.43226	0.166165
PLEKHG4B	0.101153	0.166122
AC004893.1	0.931498	0.166045
AL355803.1	-0.93688	0.166038
AC023830.1	0.960962	0.166019
KDSR	-0.05761	0.165999

POT1-AS1	0.241739	0.165994
UBL7	0.050975	0.165987
MMP7	1.506108	0.165982
MIR339	1.506108	0.165982
DEFB134	1.506108	0.165982
PABPC1P1	1.506108	0.165982
AL023775.1	1.506108	0.165982
Z93022.1	1.506108	0.165982
AL356000.1	1.506108	0.165982
ELOCP2	1.506108	0.165982
LINC01387	1.506108	0.165982
AC104695.4	1.506108	0.165982
AC108676.2	1.506108	0.165982
NA	-0.64146	0.165952
AC004918.4	-0.15499	0.165933
AL132657.1	-1.10409	0.165905
PRKAG1	0.060847	0.165894
TSPAN6	0.056378	0.16589
SNAI3	-1.09834	0.165886
PAXBP1	-0.04977	0.165875
AC017035.1	1.506104	0.165851
AC008080.4	1.506104	0.165851
NA	1.506104	0.165851
AL137782.1	1.506104	0.165851
COX7A2	0.055114	0.165832
MAN2A1	0.072384	0.165827
CSDE1	0.044028	0.165803
RBBP9	0.064177	0.165785
AC138028.3	-0.16325	0.165755
AC090559.1	-0.68404	0.165743
TEKT4P2	-0.09671	0.165738
ANKRD36C	-0.1381	0.165722
AC107956.1	-0.68901	0.16571
ODF2	0.050099	0.165697
NUS1P1	0.292456	0.165671
KCNE2	0.492333	0.165666
TIPARP	0.09009	0.165649
LNPK	0.074697	0.165628
GRHL2	-1.39185	0.165615
AL365357.1	-1.39185	0.165615
RPL7P17	-1.39185	0.165615
NACA4P	-1.39185	0.165615
RPL21P39	-1.39185	0.165615
MTND2P26	-1.39185	0.165615
AC104046.1	-1.39185	0.165615
CAMK4	-0.0875	0.165604
AC010336.5	0.096348	0.165591
AC007068.1	-0.33257	0.165566
APPBP2	-0.04926	0.165555
AC073335.1	0.95823	0.165527

LINC00115	0.414748	0.165526
ZFPL1	0.08141	0.165492
CLEC3B	1.017266	0.165459
PPP1R8	0.048802	0.165445
AC010680.2	0.19596	0.165438
SETD3	0.051362	0.16543
NA	-0.40737	0.165389
ZSCAN16-AS1	0.102889	0.165387
NECAP2	-0.04566	0.165322
THAP4	0.0655	0.165318
VMP1	-0.04672	0.165304
PDCD6	0.044385	0.165291
AC022858.1	-0.20049	0.165272
TAF6L	-0.06544	0.165229
SDAD1	0.047596	0.165225
RAB19	1.4045	0.165213
EIF4EP1	1.4045	0.165213
NA	1.4045	0.165213
TPRA1	-0.0714	0.165202
IFIT1P1	0.707041	0.165195
MUC1	-0.18946	0.165176
ZNF830	-0.07146	0.165172
KRR1	0.054499	0.16514
KPNA6	0.045152	0.165122
MED4-AS1	-0.07711	0.165119
AC091057.2	0.276722	0.165087
CAPNS1	-0.04313	0.165087
C6orf99	1.404475	0.165077
EEF1A1P30	1.404475	0.165077
LINC00636	1.404475	0.165077
AC124319.2	1.404475	0.165077
NA	1.404475	0.165077
AKR7L	0.341821	0.165002
RPL7AP50	0.749607	0.164961
ZNF354B	-0.09098	0.164912
GGT7	0.054106	0.164906
LAMC1	-0.04994	0.164903
ZNF764	0.082794	0.164855
ZNF644	-0.05429	0.164849
ZNF675	-0.11146	0.164837
NAMA	0.490479	0.164698
INO80	0.052909	0.164683
CIAPIN1	0.046458	0.164663
EDNRB	0.321836	0.164643
RAB9A	-0.07261	0.164633
PECR	0.101873	0.164614
AL132655.1	0.511905	0.164612
CHMP1A	-0.05213	0.164588
PAX6	-0.20526	0.164585
GPR63	-0.14111	0.164487

MAOB	0.096066	0.164474
HTR5BP	0.14178	0.164449
AC055720.2	0.188156	0.164424
CR383656.4	0.780474	0.164306
AC073136.1	-0.57264	0.164293
NA	-0.20526	0.164291
SETP20	0.67569	0.164289
PSPN	-0.14704	0.16426
ACSS1	-0.30319	0.164239
SNHG16	-0.04222	0.16423
ACOX1	0.050637	0.164203
ATP6V0E2	-0.05171	0.164168
TECTB	-0.91898	0.164155
RARRES2P2	-0.79075	0.164149
CHD6	0.046131	0.164143
AC079742.1	-0.06838	0.164115
FAM151A	-0.12516	0.16408
HECTD1	0.044166	0.164068
RAB5IF	0.088788	0.164066
AP001469.2	-0.32856	0.164038
AC125494.1	0.085808	0.164029
PRKD1	0.607198	0.164014
FAM173A	0.068295	0.164008
AC106870.1	-1.39481	0.164007
RF00586	-1.39481	0.164007
EGOT	-1.39481	0.164007
NA	-1.39481	0.164007
HERPUD2	-0.05688	0.164002
SMCO4	-0.15894	0.163997
ENTPD5	-0.06222	0.163975
TRA2B	0.043543	0.163937
EARS2	-0.05676	0.163924
ANXA7	-0.05533	0.163924
NA	0.357906	0.163892
NA	0.474153	0.163889
PSMC4	0.041463	0.163888
CERS1	0.133326	0.163853
STIM1	-0.09046	0.163812
ZMYND19P1	0.750542	0.163727
EIF3EP1	-0.15146	0.163721
AP001160.2	-0.16844	0.163706
TSPAN5	-0.04907	0.163661
CLRN3	-1.17192	0.163647
UNC45A	0.048357	0.163645
EMSY	-0.06236	0.163633
CLEC2D	-0.19119	0.163601
NR1D2	0.064055	0.163589
GPN1	0.044693	0.163582
AC096649.2	-1.02328	0.16357
NA	-1.02328	0.16357

NRXN2	-0.09274	0.163552
EXOC8	-0.06865	0.163542
EPHA8	0.244674	0.163526
LCP2	-0.57958	0.163508
SP6	-1.39167	0.163455
RNU6-1091P	-1.39167	0.163455
RNA5SP435	-1.39167	0.163455
LINC01435	-1.39167	0.163455
NA	-1.39167	0.163455
DYNLL1P4	-1.39167	0.163455
AARS2	-0.05763	0.163448
LINC01611	-0.71777	0.163388
SLC16A6P1	-0.33431	0.163358
TMOD3	-0.06522	0.163355
SAP30L	-0.04679	0.163346
GSTM4	0.088061	0.163294
DNAH17	0.091871	0.163284
EHHADH	0.113124	0.163282
FANCM	0.100935	0.16323
AC008481.1	-1.16976	0.163229
NA	0.313765	0.163162
AC242376.1	-0.59331	0.163119
BIN1	-0.05562	0.16308
NA	1.077107	0.163063
SLC35A2	-0.05527	0.163056
TIMM17B	0.057187	0.163035
SNRPGP15	-0.16847	0.16301
RAB3IL1	0.096981	0.162989
MIR27B	0.336748	0.162976
PPIAP41	0.997694	0.162974
EPDR1	-0.05937	0.16295
ZNF558	0.058352	0.162923
LINC01638	-1.34803	0.162911
AC006148.2	-0.67407	0.16291
AC130343.1	0.144275	0.162889
BRPF1	0.056301	0.162875
ULK2	0.063	0.162853
COL4A2-AS1	-0.1082	0.162837
FAH	0.088489	0.162726
DDX18	0.039468	0.16271
NADSYN1	-0.05428	0.162704
PCDHGA4	-0.22838	0.162673
KCNAB2	0.1184	0.162649
AC073585.1	-0.26871	0.162643
C18orf25	-0.06194	0.162607
CYB5B	0.048503	0.162601
AC010525.2	-0.57306	0.16258
KCNJ8	-0.09654	0.162562
CCSAP	-0.05797	0.162531
THOC6	0.069222	0.162524

PITPNC1	-0.06126	0.162493
AC098851.1	0.647361	0.162491
NA	0.581454	0.16249
CYP4F29P	-0.17456	0.162459
CLCN6	0.069965	0.162438
NA	-1.09209	0.162428
AL139161.1	-1.09209	0.162428
GATA2-AS1	-0.06706	0.162415
MYEF2	0.059475	0.162402
AC105052.2	0.268884	0.162399
WASF1	-0.04729	0.162371
BTN2A2	-0.09171	0.162368
SLC35D1	-0.06913	0.162355
PIAS2	0.06212	0.162346
HLA-G	0.661531	0.162345
SMARCB1	0.050118	0.162333
PRR16	0.061852	0.162299
SPEF1	-0.73237	0.162265
AC090519.2	0.757399	0.162254
AC051619.5	-0.90469	0.162227
LINC00304	0.391979	0.162157
PSMA6P1	0.599716	0.162157
RPSAP58	0.833232	0.16208
DUS2	-0.09511	0.162062
RALBP1	-0.04538	0.162061
AC005562.1	0.401885	0.162049
FBXO4	0.141372	0.162048
MED29	-0.05297	0.162038
AK6	0.054055	0.162032
SLC44A5	-0.08471	0.162015
C12orf43	-0.07375	0.161987
AC025627.1	-0.53283	0.161986
TECPR1	0.061291	0.161985
RF00019	-1.08989	0.161981
CLCP2	-1.08989	0.161981
AC026202.2	-1.08989	0.161981
SETD5	-0.0493	0.161961
SDS	-0.54565	0.161922
AC087276.3	0.12838	0.16191
PBLD	-0.09217	0.16191
LRRC73	-0.17823	0.161895
CNRIP1	-0.05059	0.161882
AC141002.1	0.507043	0.161839
TAPBP	0.067771	0.161805
KIAA2026	0.059941	0.161779
MTATP6P1	0.157469	0.16172
MIR762	0.101089	0.161703
KIF16B	-0.0869	0.161684
NR0B2	0.791295	0.161678
C12orf60	-0.11928	0.161589

AC020928.2	-0.14696	0.161587
AC093388.1	-0.46814	0.161559
SLU7	0.046583	0.161555
AL391684.1	-0.19169	0.161503
APEH	-0.04536	0.161503
STARD7	-0.04028	0.161471
RARS2	0.052516	0.161417
ECD	-0.05239	0.161346
GJC3	-0.64706	0.161296
DBNL	-0.05201	0.161293
ITPKC	0.078631	0.161257
AC135731.1	-0.79133	0.161249
MAOA	0.0764	0.161248
CLASP2	0.042919	0.161247
RN7SL316P	-0.79166	0.161179
WDR5	0.061384	0.161107
CARHSP1	0.044796	0.161093
GAREM1	-0.07727	0.161086
AL591438.1	-0.83884	0.161079
DTX4	0.304402	0.161058
RPL37P2	0.600816	0.161048
DDB1	0.038719	0.161028
ADD3-AS1	-0.48891	0.161016
ALG1L8P	0.243411	0.160969
TRIM39	0.070793	0.160964
ANKRD18B	-0.12443	0.160934
AC106782.2	-0.21749	0.160915
AC067930.2	0.426535	0.160897
CDK13	-0.04603	0.160857
ESM1	-0.89869	0.160836
MMP9	0.147841	0.16081
AP4E1	-0.06307	0.160801
AL512631.2	0.218435	0.160747
SVIP	-0.0755	0.160739
ALG1L11P	-0.48558	0.160703
LPAR2	-0.10511	0.1607
AC107027.3	0.154713	0.160686
ATP8A2	-1.01076	0.160673
DARS	0.054535	0.160647
DDN	0.100968	0.160639
GCNA	0.223526	0.160638
STRCP1	-0.36628	0.160619
RPP14	-0.05751	0.160593
C15orf62	0.119435	0.16057
PRELP	0.386854	0.160551
LSS	0.055875	0.160551
MTHFD1	-0.04597	0.16055
HK2P1	1.075669	0.160538
DPYSL4	-0.06265	0.160534
TM2D3	0.053827	0.16052

AP003680.1	-0.21683	0.160506
AL021807.1	-0.22051	0.160475
STXBP4	-0.06913	0.160472
PBXIP1	0.05444	0.160456
HMGA1P8	0.374353	0.160427
CDH24	-0.08141	0.160427
RNF11	-0.05739	0.160424
NA	0.135734	0.160409
CEBPA-DT	-0.80717	0.16039
PCDHGB1	-0.57449	0.160362
ALPK2	-0.24823	0.160334
CDIPT	0.050739	0.160311
CTBP1-DT	0.069233	0.160297
ACER3	0.060923	0.160282
AC090948.3	0.401626	0.160276
AL683813.1	0.223566	0.160258
NHP2	0.04067	0.160256
KCNE5	1.055474	0.160226
ZFP28	0.082889	0.160207
IGDCC4	-0.14201	0.160133
VIPR1-AS1	-0.78691	0.160116
KCNT2	-0.06784	0.160104
RF01233	-0.45605	0.160069
SERINC1	0.040231	0.160037
RBMS3-AS2	-0.19497	0.160027
ZBTB18	0.062871	0.159994
ATP6V1G2	-0.07046	0.15996
OR10AD1	0.817708	0.159931
ACYP2	-0.10141	0.15993
AC004987.1	-0.84215	0.15991
AL513327.1	0.217334	0.1599
AC005330.1	0.403873	0.159889
AL451165.2	-0.0948	0.159852
MARCH6	-0.03824	0.159832
RBM12B	0.055671	0.159816
KCNK13	-0.20498	0.159804
AC104365.3	0.308773	0.159803
AC096536.2	0.100056	0.159796
AL513175.1	-0.61421	0.159795
UQCRFS1P1	0.2701	0.159788
AL158151.2	-0.29652	0.159701
AC002398.1	-0.20163	0.159686
PLEKHM1P1	-0.0723	0.159673
AC024361.2	0.728946	0.159648
C8orf82	0.067234	0.159603
GZMM	-0.71803	0.159587
AC124045.1	-0.1252	0.159582
SPDYE2B	0.364938	0.159574
KCNAB3	-0.08752	0.159573
AL445465.1	0.931619	0.159556

POC5	-0.06665	0.159545
BRF2	0.062241	0.159542
FRMD4B	-0.18858	0.159431
FAM72D	0.113679	0.1594
ST3GAL6-AS1	0.109657	0.159389
LRRC17	0.071475	0.159376
ZSCAN12P1	0.087558	0.159332
WRNIP1	0.051205	0.159287
PTPRF	-0.06624	0.159243
AC245060.4	-0.21357	0.159218
LRRC27	0.104673	0.159192
DDX39A	0.041513	0.15916
EP400	-0.06226	0.159124
AC138466.2	-0.25925	0.1591
AC007225.1	-1.13837	0.15907
Z84480.1	-1.13837	0.15907
PABPC4	0.042957	0.159068
TMIE	-0.23381	0.159022
AL355297.3	-0.46447	0.159006
ZNF506	0.070601	0.158968
AC010913.1	-0.567	0.158963
SERBP1P5	-0.36127	0.158927
OCIAD1	-0.04729	0.158831
AL390198.1	0.335416	0.158831
CCER2	-0.68697	0.158825
AC020934.1	0.203941	0.158802
PLD5	-0.09126	0.158768
STAU2-AS1	0.652555	0.158727
DLSTP1	0.253187	0.158717
B4GALNT3	-0.09854	0.158704
LSMEM2	-0.09293	0.158678
CENPI	-0.07587	0.158623
LINC00843	0.157492	0.15859
NA	0.341857	0.158541
CARMIL1	0.126156	0.158529
ACD	-0.07011	0.158518
NA	-0.66477	0.158277
ZNF750	0.306734	0.158261
RPL34-AS1	0.419252	0.158259
CALML4	0.081004	0.158242
HDAC4-AS1	0.591884	0.158225
SIRT1	0.050177	0.15821
AP001011.1	-0.21358	0.158202
CA10	-0.33739	0.158202
INTS9	-0.05469	0.15817
NA	0.225279	0.158169
CARD10	-0.16161	0.158148
MARK4	0.069492	0.158119
RPLP0P6	-0.1089	0.158059
FZD2	0.069177	0.158044

AP000255.1	-0.69017	0.158041
RPS26P3	-1.13624	0.158026
AL390208.1	0.138397	0.158018
AL139095.4	-0.09207	0.158011
LINC01977	0.289251	0.157949
CYP2D6	0.333695	0.15794
NA	0.767661	0.157918
ZNF136	0.095003	0.157898
KLHL41	0.982003	0.157895
CHRFAM7A	0.162126	0.157845
MEIS2	0.047827	0.157717
TIMM9	-0.06474	0.15769
AC007881.1	1.123487	0.157677
AC034223.2	1.123487	0.157677
AC093525.4	0.284948	0.157663
AC048344.1	0.180754	0.157609
AC005262.2	-0.09744	0.157595
ALG5	-0.06383	0.157593
ARAP3	-0.04704	0.157441
CFL1P1	-0.12074	0.157431
NA	-0.15927	0.157408
SH3GL1	0.047834	0.157373
UQCC1	0.046753	0.157359
LINC02525	0.912686	0.157341
ASB2	0.857213	0.157334
AC092800.1	1.086743	0.157332
AC090971.3	-0.38653	0.157245
KTN1	0.047301	0.157227
LINC01018	-0.31131	0.157223
RF00019	1.033767	0.157218
NCBP2	0.038032	0.15721
IFIT3	0.217764	0.15721
PTPN11	0.039231	0.15719
QARS	0.043585	0.157186
ZNF815P	-0.08166	0.157172
KIAA0895L	0.053237	0.157124
AC004908.2	0.2202	0.157107
DFFB	0.10621	0.157105
CLDN22	0.2198	0.157097
PA2G4P4	0.516228	0.157076
ZDHHC16	-0.05008	0.156965
AC079601.1	0.07288	0.156963
CPNE5	0.362504	0.156957
EIF5A	-0.03545	0.156936
AL355312.3	-0.15755	0.156898
SLC35G5	0.552298	0.156855
ITM2B	-0.03917	0.156833
AC135178.2	-0.53164	0.156817
AC110491.1	0.592406	0.156791
AL121655.1	0.608967	0.156786

HSP90AB1	-0.03624	0.156727
AC073834.1	0.237137	0.156705
NA	-0.10315	0.156698
RF00019	-0.72946	0.156691
UBXN4	-0.04524	0.156624
GLE1	-0.05148	0.156559
AC079250.1	0.463074	0.156542
AC005261.3	-0.22805	0.156465
ELFN2	0.767856	0.156453
NAPSB	-0.52371	0.15642
APOLD1	0.117363	0.156413
TRIM2	-0.04604	0.156394
EP400P1	-0.09001	0.156368
BAALC	-0.18157	0.156365
TUBB2B	0.042706	0.156354
NSG2	0.037514	0.156321
ISCA2	0.071613	0.1563
TARBP2	0.067385	0.156295
BAHD1	0.067164	0.156285
ZEB1-AS1	0.08428	0.156266
NME7	0.056499	0.156238
DUXAP9	0.056808	0.156227
AL031985.3	0.105447	0.156225
PRSS50	-0.62619	0.156206
AC002543.1	0.731112	0.156196
AL512625.1	0.096751	0.156175
AL499616.1	-0.30983	0.156106
SYNJ2	0.076566	0.156086
MAP4K1	-0.32196	0.156069
AL512306.3	-1.21548	0.156045
AC244090.3	-1.21548	0.156045
AP001880.2	0.521309	0.156036
EXOSC10-AS1	0.130844	0.156034
RBPMS-AS1	0.270206	0.156019
DSCAML1	-0.6331	0.156001
SLC25A26	0.060358	0.155997
AC093752.2	0.85182	0.155996
OBSCN	0.06382	0.155994
MTHFS	0.273679	0.155975
CSE1L	-0.05312	0.155959
TMEM183A	0.044031	0.155951
SLC25A5	0.03791	0.155946
MIR4766	0.878138	0.155943
XXYLT1	0.052491	0.155943
GRIP1	-0.37198	0.155907
RBIS	0.078208	0.155836
SLC12A8	-0.16331	0.155822
EID1	-0.05126	0.155803
SNORA22	-0.45027	0.155789
AC011450.1	0.76482	0.155752

PFN2	0.041251	0.155733
TYW3	-0.05654	0.155727
AL008729.2	-0.17547	0.155724
MAB21L2	-0.2073	0.155676
RPL24P2	-0.75064	0.155674
ADAMTSL4-AS1	-0.48472	0.155658
POMZP3	-0.06437	0.155618
AC092802.2	0.315225	0.155613
RAB3D	0.077467	0.155602
SIRT7	-0.05221	0.155554
PDLIM1P4	0.284003	0.155553
NA	0.598611	0.1555
TMEM120B	0.058518	0.155391
POLD1	-0.05472	0.155372
KLF1	0.243209	0.155368
C1QL3	-0.33273	0.155312
NPM2	-0.54668	0.155304
MINDY1	-0.07651	0.155248
NA	0.973398	0.15524
PSMB6	-0.04643	0.155237
AC245452.1	0.115827	0.155195
B3GAT1	-0.09064	0.155194
SULT1A1	-0.62008	0.155184
SERPINA5	-0.14547	0.15518
OVCH1	0.775116	0.155122
KCNRG	-0.53847	0.155118
CTSB	-0.04552	0.155113
TSPY26P	-0.11166	0.15508
AC144831.1	0.228261	0.155067
AC073534.1	-0.48103	0.155029
TIRAP	-0.09738	0.15502
AC012618.3	-0.9734	0.154995
AP001107.7	0.1476	0.154969
ZNF251	-0.0651	0.154931
WDR13	0.054893	0.15491
PLA2G4B	0.498365	0.1549
PRORSD1P	0.149137	0.154878
RGS7	0.244693	0.154874
ZNF852	-0.09127	0.154869
AL355472.3	-0.36477	0.154842
SIGLEC16	0.751806	0.15483
NDUFA6-DT	0.195809	0.15479
CELF2	0.365932	0.154786
AL008726.1	-0.08676	0.154724
HBEGF	0.106934	0.154646
SOX30	1.005902	0.154614
CPLANE1	0.064776	0.154572
GPBP1	0.050825	0.154571
AC005954.1	0.073313	0.154546
NA	0.642092	0.154504

TMEM220-AS1	0.162828	0.154498
SPIN4	-0.07536	0.154487
IGHV4-61	-0.86324	0.154429
AL390195.1	-0.44403	0.154412
SOD1	0.045448	0.154386
PIK3CA	0.049012	0.154303
TMCC1	-0.05472	0.154296
HLA-DMA	0.197873	0.154285
ID1	0.054704	0.154284
FOXN4	0.154747	0.154259
ZNF610	0.182494	0.154168
FNDC4	0.065723	0.154141
LRRC53	-0.90328	0.154117
SLC35F2	-0.06997	0.15409
FAAP100	-0.0634	0.154083
PLCXD2	-0.20462	0.154081
THAP12P7	-0.54549	0.154056
ZNF766	-0.0891	0.154052
AP002008.1	-0.61943	0.15405
AL731556.1	0.96938	0.154024
TADA1	-0.0558	0.154021
ZNF736	0.050401	0.153993
AC084880.1	-0.39056	0.153992
CENPN	0.064198	0.153981
TOE1	0.063503	0.15397
NA	-0.05298	0.153944
PRELID3B	0.054054	0.153929
TMEM161B-AS1	-0.10857	0.153909
MYBBP1A	-0.05769	0.153882
GAR1	-0.05422	0.153846
ZNF26	0.226012	0.153796
AP000254.1	-0.21607	0.153774
SP110	0.094104	0.153766
SNAP47	0.046773	0.153759
STRC	-0.40876	0.153758
KDM4A	-0.04997	0.153735
CREB1	0.04758	0.153734
NOXO1	0.116851	0.153703
HMBOX1	0.055788	0.153695
TRAK1	-0.06303	0.153647
AC010768.1	0.206112	0.153644
STIL	0.063866	0.153623
ABCC11	0.150084	0.153615
UCHL1-AS1	1.093271	0.153608
GTF3A	-0.04593	0.153605
RPL3L	0.460811	0.153584
C1orf21	-0.05084	0.153584
CD101	-0.32985	0.153579
GFM1	0.043834	0.153577
MMADHC	-0.04866	0.153569

ANAPC5	0.041314	0.153556
PSAP	-0.03632	0.153538
ZNF839	-0.06458	0.153531
AL359715.3	0.283395	0.153529
IL7	-0.06968	0.153525
FLVCR1-DT	-0.11894	0.153499
RN7SKP180	-1.39715	0.153484
BTNL10	-1.39715	0.153484
AC008073.1	-1.39715	0.153484
NA	-1.39715	0.153484
RPSAP21	-0.90695	0.15347
UFSP1	0.132357	0.153457
AC061992.1	-0.42261	0.153445
PI4KA	-0.05019	0.15344
RCCD1	-0.05511	0.153436
STRAP	-0.04521	0.153416
C5orf22	-0.0568	0.153409
NA	0.162463	0.153388
DNAJC2	-0.04915	0.153387
TONSL-AS1	-0.13212	0.153358
NA	0.045749	0.153322
GSS	0.048161	0.153299
STT3B	0.040119	0.153296
NA	0.363518	0.153278
SCN3A	0.055724	0.153274
ALKBH5	-0.06013	0.15326
AP001458.1	-0.15701	0.15324
AL049844.2	-0.22358	0.153238
RNU6ATAC3P	0.959197	0.153219
MSX2	-0.07798	0.153199
ZNF248	0.056927	0.153175
AL360181.2	-0.22387	0.153157
U62317.3	-0.61496	0.153141
RGMB	-0.05419	0.15312
DNAJC8	0.045777	0.153094
TMEM246-AS1	-0.24926	0.153084
ZNF454	-0.18454	0.153038
STAT6	0.150919	0.153011
AC090515.4	-0.37635	0.153002
LINC01006	-0.33025	0.152974
LINC00184	-1.01296	0.15295
UBL5	0.051926	0.152945
RPS6KL1	-0.07238	0.152935
AL022345.1	-0.39266	0.152928
AC113404.1	0.314601	0.152905
RASL10A	0.470559	0.152899
BNIP3P11	-0.39989	0.152874
AC112721.2	-0.79369	0.152828
AC093484.3	0.041534	0.152819
PPP1CA	0.041307	0.152813

COX7A2L	0.050351	0.152775
NA	0.47133	0.152758
GHITM	0.041684	0.152726
NA	0.03144	0.152708
DLEU2	0.12035	0.152655
ISCA1	0.059727	0.152635
NAP1L4P1	-0.40469	0.152613
CDCA5	-0.0576	0.152601
HDDC2	-0.05774	0.15259
AC103952.1	-0.32796	0.152587
AL354702.1	-0.70442	0.152565
SH3YL1	-0.05201	0.152536
CUX2	-0.06695	0.152528
CRYBA1	-1.01082	0.152522
SHISA2	0.746032	0.152502
PLEKHF2	-0.13239	0.152501
METAP1	-0.05402	0.152466
SPDYA	0.107265	0.152466
AKNA	-0.18142	0.152446
THAP9-AS1	-0.05798	0.152439
MCCC1	0.063222	0.152412
PPP2R3B	-0.06151	0.152412
ENDOU	0.89137	0.152402
SNAI3-AS1	0.195028	0.152386
SIX6	-0.18133	0.152378
PLA2G6	-0.09553	0.152375
CENPM	0.097224	0.152362
FKBP14	-0.05022	0.15235
AL008635.1	0.815525	0.152337
AL049874.3	-0.95666	0.152331
AL139008.1	-0.89343	0.152322
TIMP3	-0.09492	0.15227
DNAJB12	0.056062	0.152248
CLDN12	0.042901	0.15222
LRMDA	-0.55266	0.152218
ENPP7P2	-0.75113	0.152211
FAM186B	0.093459	0.152196
TBCA	0.047579	0.152189
PPARG	0.275885	0.152154
SSBP3	0.049105	0.152142
AC068888.2	-0.94502	0.152111
SMIM27	0.110767	0.15211
SHLD2	0.049553	0.152073
ST3GAL1P1	-0.57621	0.152029
AC016717.2	0.205767	0.152015
CREB3L2	0.06178	0.152
TRAPPC4	-0.0614	0.15198
SF1	-0.04962	0.151972
ZNF835	-0.42284	0.151969
RF00019	-1.38757	0.151946

Z82206.1	-1.38757	0.151946
LINC02392	-1.38757	0.151946
LAGE3	0.072781	0.151938
AC007038.1	0.210621	0.151936
COX18	0.057899	0.151902
WDR7	0.07213	0.151892
CDC42P6	0.340621	0.151891
CTSL	0.05612	0.151841
CWC15	0.047776	0.151827
DTWD1	0.073405	0.151812
KRT8P30	0.225766	0.15179
CRLF3	0.05571	0.151783
RYBP	-0.04613	0.151782
LGMN	-0.06455	0.151781
RPS6KB1	0.044775	0.151736
TANC2	0.045985	0.151698
OAZ2	-0.04779	0.151692
MOGAT1	0.686571	0.151689
FTH1P7	0.686571	0.151689
APOBEC3F	-0.25427	0.151661
IL1RAPL2	0.567332	0.151659
ZFX-AS1	-0.26324	0.151652
STK24-AS1	0.576539	0.151647
COMMD5	0.052	0.151646
MTERF1	0.064542	0.151642
TCTA	-0.06434	0.15164
ALOX15B	1.133921	0.151612
RN7SL154P	1.133921	0.151612
ZNF572	0.092449	0.151607
AC011825.4	0.2263	0.151602
RRP8	-0.063	0.151585
ENPEP	-0.14025	0.151549
PTPRR	-0.16179	0.151537
NUBP2	-0.05155	0.151519
GLIPR1	0.057517	0.151487
AL136310.1	0.619218	0.151462
KPNA2	-0.04751	0.151453
SPIRE2	-0.06965	0.151451
AL137845.2	0.885999	0.151445
SNRNP25	0.052789	0.151421
MCM8	-0.05304	0.151401
U2AF2	0.038137	0.15139
TAS2R13	-0.76072	0.151356
EDA2R	0.075159	0.151345
SPATS2L	-0.07219	0.151328
PARD6G	0.106596	0.151283
ZNF496	-0.05096	0.151266
SPRED1	0.049837	0.151227
NOB1	0.050805	0.151206
EPHX3	-0.40838	0.151123

AC012467.2	0.101256	0.151119
AAK1	0.091276	0.151111
MAP3K10	0.064355	0.151092
AC063948.1	0.660305	0.15109
GAD1	0.799215	0.151069
DUT	-0.04875	0.151057
RAB40C	0.05939	0.151054
KDM1B	-0.05751	0.15105
SNORD1B	0.574891	0.151016
HNRNPA1L2	0.090941	0.151001
LINC00638	0.15561	0.150999
CYP4A22-AS1	-0.92697	0.150986
NA	-0.92697	0.150986
AC114488.2	-0.2211	0.150979
NUP54	0.057264	0.150979
MIR328	-0.67188	0.150977
AL158071.3	0.763547	0.150943
AC002456.1	-0.70395	0.150893
AC034102.2	0.240887	0.150859
RNU6-387P	0.422904	0.150837
AC092567.1	-0.18862	0.15083
ADAMTS16	-0.32082	0.150768
DHX32	0.050282	0.150759
BAZ1B	-0.03484	0.150739
DDX50P1	0.225519	0.150737
GPS1	0.044016	0.150728
AC018682.1	0.352272	0.150721
WASHC5	-0.04731	0.15072
CFAP157	-0.08383	0.150701
LENG1	-0.07719	0.150653
AC022217.1	-1.39696	0.150634
RPL15P20	-1.39696	0.150634
C3orf86	-1.39696	0.150634
AC034236.1	0.098691	0.150632
NA	-0.11122	0.150614
AC009061.1	-0.6248	0.150588
PXK	0.09158	0.15055
AL772363.1	0.381241	0.150483
AL136309.4	0.152572	0.150475
PPP1R27	0.460451	0.150454
POLH-AS1	0.193366	0.150453
GUK1	-0.03795	0.150444
LETMD1	0.054483	0.150421
AL512353.1	-0.13239	0.150406
KDM2B	0.057763	0.150404
EIF1AX	-0.04676	0.150392
ORC2	-0.04456	0.150364
AL139132.1	-0.94716	0.150349
ATP6V1G3	0.642656	0.15031
LINC00877	0.450854	0.150278

AL049612.1	0.873254	0.150265
AC005062.1	-0.49342	0.150261
TOMM70	-0.04161	0.150231
UCHL1	0.042884	0.150227
CARD8-AS1	-0.13139	0.150214
AC100803.3	0.653404	0.150207
KRT18P63	-0.44444	0.150198
C1orf54	0.060783	0.150119
NA	-0.40344	0.150067
CGGBP1	-0.0424	0.150052
HRAS	-0.06211	0.150038
KIRREL2	0.185112	0.15002
HEATR5B	0.044112	0.150013
IDNK	0.144802	0.14997
SNORA59A	-0.64509	0.14996
CSK	-0.05275	0.149896
KHNYN	0.044471	0.14989
NA	-0.21538	0.149884
GRHL1	-0.14951	0.149864
PFDN4	0.060304	0.149858
DCAF10	-0.05011	0.149854
PPP4R3A	-0.0466	0.149781
AC005532.1	0.588702	0.149772
SELENBP1	0.36599	0.149764
PRSS51	0.798429	0.149749
HARS	0.037508	0.149704
GEMIN8	-0.06162	0.149685
AC002064.1	-0.84985	0.149669
NA	-0.08026	0.149658
AC112229.3	0.215624	0.149643
ARRDC4	-0.09638	0.149627
TREX2	0.294205	0.149613
GUF1	-0.0541	0.149609
AC139149.1	0.40701	0.149596
WDR33	0.04237	0.149595
AC100854.1	0.297731	0.149565
RPA3	-0.05807	0.149523
TLN2	-0.0571	0.149499
PPIAP53	0.354825	0.149439
AL353807.3	-0.14696	0.149438
TRAIP	0.063243	0.149435
ACOX2	0.796754	0.149412
RAC1	-0.03631	0.149406
IFIT2	-0.22206	0.149404
ODF3B	0.115771	0.149393
IVNS1ABP	0.037495	0.149322
PPP2R1B	-0.06882	0.149319
PCM1	-0.04577	0.149306
CSNK2A2	0.045169	0.149302
DHX16	-0.05261	0.149301

SOX5	-0.14391	0.14928
AC013268.1	1.038894	0.149274
AL355802.1	-0.3121	0.14927
AC009053.1	-0.08696	0.149253
ZDHHC9	0.059389	0.149237
LINC02363	0.645006	0.149232
COX10-AS1	0.077405	0.149227
OST4	0.045732	0.149178
AC000068.3	-0.16182	0.149163
PIGS	-0.0483	0.149132
AL136368.1	0.204035	0.149119
CCDC196	-1.38733	0.149104
AC008687.3	-1.38733	0.149104
RPL10P12	-0.48594	0.149097
AC099786.2	-0.2438	0.149059
AL603832.1	0.157077	0.149043
AP003717.1	0.320574	0.14904
HMMR	0.067174	0.149032
FN3K	-0.08836	0.148941
CSTF3-DT	-0.91718	0.148931
ZC3H11B	0.553695	0.148924
RNF19B	0.095571	0.148901
RAP1A	-0.05851	0.148896
AC008403.2	0.094167	0.148879
LYPLA2	0.040999	0.148857
AC139530.2	0.054839	0.148854
MIR103A2	0.342997	0.148846
AP001267.3	-0.10574	0.148809
LINC02421	-0.63202	0.148807
NPHP4	-0.08848	0.148805
RDH10-AS1	0.551695	0.148703
DVL3	-0.04854	0.148696
DBIL5P	0.307751	0.148681
NLN	-0.04801	0.148679
PDCD2	0.041084	0.148657
NA	0.564897	0.148651
AC246787.2	-0.76509	0.14859
RPS15AP36	-1.39681	0.148569
AL513343.1	-1.39681	0.148569
AC011995.2	-1.39681	0.148569
AL139300.2	-1.39681	0.148569
AC008770.2	-1.39681	0.148569
KIAA1109	-0.0532	0.148495
AC007773.1	0.250246	0.148491
MPI	-0.06439	0.14849
VPS54	-0.0589	0.148485
AL139317.3	0.054228	0.148475
AC092685.1	-0.39018	0.148471
NOL10	0.047442	0.148451
AL359265.1	-0.838	0.148383

PLIN5	0.28357	0.148365
ZNF197-AS1	0.285078	0.148357
HSD17B4	0.040891	0.148351
POMT1	-0.06698	0.148326
CDIP1	-0.07882	0.14832
WDR31	0.176389	0.148319
AC010632.1	0.204952	0.148317
PRMT1P1	0.685998	0.148267
AL139317.4	-0.36695	0.148257
AC073508.2	-0.20249	0.148245
NA	-0.5586	0.148228
RF00019	0.786501	0.148195
NUP85	0.047922	0.148166
WDR90	-0.0627	0.148136
TUBB2A	-0.07374	0.148107
AC023310.1	-0.91736	0.148096
CTCF	-0.05786	0.148072
AC104561.1	-0.37724	0.148063
NUDT1	0.05932	0.148058
COX19	0.070219	0.148006
AC026462.1	-0.38574	0.147997
AC108002.1	-0.12223	0.147988
AL136131.3	0.108781	0.14796
MIOX	0.844218	0.147947
COA6	0.061309	0.147941
SFMBT2	-0.06105	0.147933
AC004151.1	-0.10199	0.147912
AC006026.3	-0.15126	0.147893
PKHD1	-0.36103	0.147891
FAM13C	0.056759	0.147883
PMS2CL	0.06693	0.147872
FUS	0.034194	0.147871
UBXN11	-0.05489	0.147866
UBE2R2-AS1	-0.10216	0.147862
AC009948.1	0.492686	0.147822
AP006621.3	0.152624	0.147817
RPL23AP74	-0.61609	0.14781
CCDC102A	-0.08767	0.147809
ZNF92	-0.0593	0.1478
ARFGAP1	-0.04608	0.147767
AC012615.4	-0.4569	0.14776
RASAL2-AS1	0.285903	0.147704
EIF2B4	0.046876	0.147695
RAMAC	-0.07781	0.147676
TCEAL1	0.073669	0.147664
PARS2	0.09389	0.147639
NSUN5P2	-0.05225	0.147622
RNA5SP162	-0.59609	0.147593
SMCR5	0.505314	0.147569
STK33	-0.08587	0.147564

AL353693.1	-0.29938	0.147535
WASHC5-AS1	-0.11913	0.14752
GPR153	-0.08257	0.147498
AC079140.3	-0.37147	0.147435
NFKBIE	0.070451	0.147401
AL663070.1	-0.47524	0.1474
DNAH14	-0.06005	0.147399
UTP20	0.044852	0.147359
PRDM12	-0.52688	0.147356
SHROOM2	0.427785	0.147341
AC093110.1	0.113576	0.147334
LMBR1L	0.059455	0.147329
AC104794.2	-0.22626	0.147265
SNORD15B	-0.48792	0.147262
MALAT1	0.057594	0.14726
USP12	0.049897	0.147233
NA	0.370386	0.147204
ATIC	-0.04335	0.147201
RBM38	0.071228	0.147184
AC008569.2	0.840023	0.147171
AL138787.1	0.793373	0.147153
MCRIP2	0.068199	0.147148
SIK3	-0.06337	0.147146
RUFY3	0.039404	0.147111
RPL12P17	0.715121	0.14709
CRTC1	0.061084	0.147087
PGAM1P4	-1.38715	0.147042
Z97832.1	-1.38715	0.147042
NIP7P3	-1.38715	0.147042
LINC00691	-1.38715	0.147042
AC114684.1	-1.38715	0.147042
AL121761.1	-1.38715	0.147042
UMPS	0.050916	0.147033
CHST14	-0.0543	0.147027
EPCAM-DT	0.10981	0.147026
RNA5SP187	0.52815	0.147016
AC008011.2	0.288858	0.14699
MSMO1	0.149135	0.146984
CIITA	-0.09423	0.146959
AL139294.1	0.648276	0.146952
PSD3	0.051566	0.146942
MR1	0.162971	0.146869
AC073349.2	-0.14052	0.146853
SCLT1	-0.07341	0.146822
BX322234.1	0.352349	0.146781
AL365295.1	-0.38354	0.146771
PTPRVP	-0.43845	0.14677
AD001527.1	0.500771	0.146756
RNA5SP203	-0.66406	0.146755
KIF2C	0.048028	0.146747

AC108471.2	-0.23857	0.146722
NA	0.604112	0.146702
LINC01023	-0.22609	0.146694
PI15	0.061404	0.146674
DUS3L	0.061738	0.146663
YWHAH	-0.04691	0.1466
TSIX	-0.04009	0.146586
AC008750.2	-0.72926	0.146581
MIR615	0.852751	0.146536
MDM1	-0.06559	0.14653
TMEM121	0.113426	0.146525
AC073878.1	0.744667	0.146493
OVGP1	0.223481	0.146486
C1orf100	-1.0483	0.146467
RNU6-45P	-1.0483	0.146467
LTA	-1.0483	0.146467
AC114967.1	-1.0483	0.146467
AC027288.3	0.717603	0.146445
E2F3-IT1	0.78638	0.146408
ATP6V1B2	-0.04093	0.146403
BX322635.1	-0.32218	0.14637
SNTB1	0.180914	0.14637
AC103923.1	0.394234	0.146348
WDYHV1	0.074753	0.146331
COQ3	-0.09127	0.146316
RHBDD3	-0.06078	0.146314
JKAMP	-0.04419	0.146264
NUDT4B	-0.73042	0.146235
NA	-0.12588	0.146232
WDR77	-0.05373	0.146216
B3GNT10	-0.09357	0.146145
AL451042.2	0.684194	0.146141
LAMTOR5-AS1	0.066542	0.146131
CPT1A	-0.04691	0.146111
PNPLA2	0.055479	0.146086
NA	-0.07175	0.146063
HNRNPA3P6	-0.17311	0.146036
BACH2	0.074241	0.146001
TM2D2	-0.06055	0.145996
NA	1.069086	0.145993
GMPPB	1.069086	0.145993
RNU6-1305P	1.069086	0.145993
SCARNA10	1.069086	0.145993
HSPA2	-0.09275	0.14598
PPP1CC	0.034752	0.145967
LUCAT1	-0.41975	0.145962
AFAP1	0.047207	0.14595
GUCA1A	0.099636	0.145923
AC017074.1	0.573338	0.145829
RPS21P4	0.30278	0.14582

LGALS3	-0.05698	0.145728
FAIM2	0.198998	0.145713
SMIM10L2A	0.286723	0.145705
ZNF548	0.077763	0.145693
MND1	-0.09544	0.145683
MTM1	0.093067	0.145683
ANGEL2	-0.04721	0.145668
MFSD10	0.048284	0.145668
AC106795.2	-0.36325	0.145667
AC079140.4	-0.61731	0.14566
GOLPH3L	0.052401	0.145656
RTF2	0.047182	0.145634
AC108451.2	-0.15123	0.14562
AC068790.4	-0.60793	0.14562
ESRP2	-0.33811	0.145595
MRPL57	0.051927	0.145591
MDH1B	0.181569	0.14559
GLUL	-0.03599	0.145586
CEMP1	0.076128	0.145555
SEMA5B	0.122507	0.145539
CBS	-0.03598	0.145537
TUBGCP2	-0.04156	0.145525
AL021707.4	0.510285	0.145517
RHOXF1	0.914787	0.145497
AL645608.4	-0.57767	0.14547
PNPLA4	0.096767	0.145412
AC008443.5	0.353641	0.145387
SMG1P4	-0.71047	0.145385
SUOX	0.057048	0.145372
VDAC1	0.038035	0.145372
ABHD11-AS1	-0.45013	0.145368
KYAT3	-0.05149	0.145351
IGBP1	0.051166	0.145344
AC005884.1	-0.32327	0.145324
PTOV1-AS1	-0.08198	0.145298
TNNT1	0.210278	0.145295
AC060780.1	-0.09546	0.145292
NSMAF	0.046144	0.145251
MEX3C	0.042205	0.145239
DIO2-AS1	-0.61794	0.145226
SNORA14B	0.395055	0.145166
MTFR1	0.064455	0.145157
SEC1P	0.201793	0.145133
AC245052.1	-1.07235	0.145123
PKN2-AS1	-1.07235	0.145123
EIF3B	-0.03738	0.145097
PRPF39	-0.04831	0.145066
TMEM143	-0.07991	0.145058
BCAM	0.909827	0.145043
ZNF384	-0.04354	0.145033

LINC01909	0.473642	0.145031
ECH1	0.053401	0.144992
NA	0.692556	0.144991
RIF1	0.04971	0.144985
HNRNPRP1	-0.72006	0.144977
TSPO	-0.05373	0.144951
ING5	-0.04726	0.14493
DCAF17	0.052451	0.144924
DNHD1	-0.06677	0.144917
CNOT1	0.034502	0.144916
ZNF250	0.058334	0.144896
ZFP82	0.086828	0.144846
AC025259.1	-0.05935	0.144835
SCYL3	0.056059	0.144811
WDR36	0.046967	0.14477
ANAPC2	-0.05246	0.144748
DEFB109D	0.473266	0.144735
SCAMP2	0.060245	0.144683
RF00272	-1.0365	0.144668
PNKD	-0.05523	0.144647
TWF2	0.071863	0.144624
AL139011.1	-0.10738	0.144547
AC012307.1	0.150041	0.144532
SLC30A9	0.054195	0.144473
MIR3936HG	0.143431	0.144439
CD5	1.096531	0.144417
ADAMTSL2	0.11921	0.144391
PTPDC1	-0.07236	0.144364
JAGN1	0.04654	0.144308
CCSER2	0.057486	0.144306
PFN1P8	-0.3925	0.144259
NA	0.173653	0.144256
NUP133	-0.04059	0.144244
FGF1	-0.20689	0.144225
RPS2P5	0.065885	0.144218
AC009387.1	-0.38541	0.144169
SLC13A4	-0.05554	0.144152
ANO7L1	0.319032	0.144148
GMIP	0.079631	0.144145
NA	-0.80465	0.144108
AC005154.2	-0.22484	0.144059
PIK3C2A	0.043361	0.144031
MRPL4	0.049976	0.144008
RNU2-46P	0.905786	0.144002
CLIC3	-0.37177	0.143999
TLX1	0.843874	0.143966
AP000265.1	0.843874	0.143966
ZFR	0.037226	0.14393
HSF2BP	0.227982	0.143903
ABTB1	0.068715	0.143863

AL354989.1	0.377139	0.143862
SUGT1P3	-0.25904	0.14385
AC004596.1	0.096257	0.143792
PPP1R35	0.052449	0.143779
XPNPEP2	0.206697	0.143752
NA	0.36827	0.143742
VN1R81P	0.194194	0.143643
ATPAF1	0.053415	0.143639
MBNL1-AS1	-0.13223	0.143635
NA	0.77794	0.143614
GPR173	-0.05359	0.143562
RNA5SP298	-1.05938	0.143561
SNORD8	-0.98626	0.143537
AP005131.2	-0.66875	0.14353
GIPR	0.171315	0.143518
AC021851.1	-0.37806	0.143516
LINC00645	0.308005	0.143492
UBAP2L	-0.0375	0.14348
SCOC	-0.04929	0.143445
BRD2	0.041582	0.143436
EGFL7	0.076579	0.143434
AL021407.1	-0.78549	0.143401
SPRYD7	-0.08532	0.143387
ANP32E	-0.04415	0.143377
NA	-1.06256	0.143354
DHTKD1	0.047214	0.143334
CITED1	0.127834	0.143317
TCEAL4	0.049333	0.143303
ZNF649-AS1	-0.23849	0.143283
AL008723.2	0.617751	0.143282
NKAP	0.062175	0.143277
LCMT1-AS1	-0.3458	0.143271
SUDS3P1	0.375236	0.143262
SUPT16H	0.035381	0.14326
EIF2B5	-0.04265	0.143238
AC103591.3	0.727882	0.14321
NA	0.604024	0.143177
PPP1R12A-AS1	0.14226	0.14317
AC010997.4	-0.30578	0.143167
AC010894.2	0.451877	0.143072
MFSD5	0.059049	0.143064
L3MBTL4	0.087726	0.143041
FAM219B	0.043522	0.142994
MRPL40	-0.06267	0.142985
LPAR6	-1.06214	0.142961
EXT1	0.063684	0.142936
DDI2	-0.05644	0.142933
KCNJ3	-0.1753	0.14293
IFI16	-0.26507	0.142898
PCDHB19P	0.475465	0.14288

NR2C2AP	-0.05671	0.142862
STARD5	-0.20032	0.142847
AC135457.1	0.053466	0.142799
AC068790.2	-0.39778	0.142795
SPP1	0.300356	0.142794
PMPCA	0.041762	0.14278
FKTN	0.05515	0.142764
AC104058.1	0.837329	0.142762
LINC01352	0.783629	0.142761
MLLT11	-0.0333	0.142748
NOL4	-0.07105	0.142695
PTMA	0.031228	0.142626
C12orf71	-0.96014	0.14262
SLC1A2	0.295576	0.142616
NOTUM	0.138777	0.142584
AL118558.3	0.358164	0.142513
CPED1	-0.33123	0.142499
EIF4G3	-0.03849	0.142498
TAF1	-0.04212	0.142493
CSNK1D	-0.03984	0.142491
ITGB2-AS1	-0.4808	0.142484
NA	-0.04961	0.142453
LLGL1	0.047359	0.142441
AC125611.2	0.659056	0.142434
RRH	0.800963	0.142394
RNU6-689P	1.347108	0.142388
ARHGEF7-IT1	1.347108	0.142388
AC005410.1	1.347108	0.142388
NA	1.347108	0.142388
AC087257.2	1.347108	0.142388
AC021755.2	1.347108	0.142388
MIR3125	1.347108	0.142388
AC011462.3	1.347108	0.142388
TMEM266	-0.18104	0.14238
EVC2	0.132988	0.142336
HP1BP3	-0.03624	0.142274
TCEA1P2	0.236656	0.142266
OIP5	0.081152	0.14226
C16orf70	0.058542	0.142236
SEC24B-AS1	0.305556	0.142236
AC243562.1	-0.43911	0.142226
COL4A4	0.549791	0.142214
FANCF	0.055071	0.142167
RDH11	0.045483	0.142154
TIMM10B	0.046777	0.142154
RF00019	-1.05751	0.14212
AC009097.4	0.651725	0.142103
KDM6A	0.053497	0.142065
ZNF75D	0.066689	0.142036
MARK2P8	-0.88393	0.142036

AC020658.2	0.781035	0.142024
NA	0.336997	0.142011
SYTL4	0.133641	0.142006
FANCE	-0.08521	0.141992
MIR621	0.120491	0.141984
FMR1	-0.04885	0.141965
C4B	-0.53073	0.141964
FAAH	0.124659	0.141951
VN1R34P	-0.71445	0.141908
NA	-0.71445	0.141908
F3	0.158587	0.141901
RPS12P5	0.914512	0.141881
AC135050.4	-0.56207	0.141786
AC022916.1	-0.61717	0.141781
ANKHD1-EIF4EBP3	0.187179	0.141754
CPE	0.039652	0.141728
SF3B5	0.0516	0.141724
PHF6	-0.04228	0.141712
AC024896.1	0.087524	0.141689
CREM	-0.06687	0.141643
PAICS	0.041308	0.141636
TRIM8	-0.05349	0.141629
ART3	-0.92306	0.141619
AL133499.1	-0.92306	0.141619
SERTAD2	-0.05236	0.141608
ZNHIT1	0.037244	0.141603
AL033528.2	-0.11224	0.141597
ZNF331	-0.05404	0.141593
NA	0.639702	0.14159
EBP	-0.05141	0.14159
TP53BP2	0.036262	0.141587
IMPA1	-0.0555	0.14157
SURF4	-0.04125	0.141533
PDAP1	-0.0326	0.14152
PSD2-AS1	-0.43274	0.14148
PYGM	0.494104	0.141477
APOO	0.080695	0.141448
C1QTNF1-AS1	-0.14764	0.141439
SPON2	-0.16031	0.141419
CLDN7	0.479547	0.141413
AL590428.1	0.675857	0.141406
ABCC5	0.039468	0.141353
TMED8	-0.04688	0.141297
PIKFYVE	-0.04319	0.141281
LANCL2	0.046995	0.141274
PRELID1	0.035925	0.141268
MBD3	-0.03999	0.141257
ZNF252P-AS1	0.197299	0.141251
AC015921.1	0.110165	0.141214
CRLF1	-0.06767	0.141202

EEF1DP3	0.255394	0.141199
NA	-0.30678	0.141128
ZNRF1	-0.04942	0.141115
NA	-0.44317	0.141108
PATL1	0.058805	0.141101
PHTF2	-0.05425	0.141097
AC008443.1	-0.10336	0.141045
AC010327.4	-0.24572	0.141042
KAT2A	0.043636	0.141014
NKAPD1	-0.05633	0.141013
SNORA80A	0.847425	0.14101
SIDT2	-0.07955	0.141004
FABP6	0.534882	0.140951
P2RX4	0.09426	0.140925
NFYA	-0.04225	0.140905
SMS	-0.03946	0.140901
PRRC1	0.047588	0.140885
MYLK3	0.284383	0.140843
LECT2	0.164293	0.140809
AC115676.1	-0.60256	0.140732
SLC25A39	0.038134	0.140726
ARHGAP25	-0.61003	0.140704
ZNF470	0.065205	0.140671
SUGCT	0.130846	0.14065
AP003486.1	-0.10407	0.140628
CAMSAP1	-0.03845	0.140607
SUMO2	0.040011	0.140594
FAM72A	-0.10831	0.140552
MED17	-0.04364	0.140541
MAF	0.844675	0.140537
RCC2	-0.0361	0.140515
ADH5P4	-0.88078	0.140514
IPMK	-0.06083	0.140513
MBD2	0.052918	0.140463
AC090774.2	0.50966	0.140444
CLNS1A	0.037705	0.140439
FYB2	-0.56557	0.140432
MLN	1.346567	0.140371
SLC22A6	1.346567	0.140371
ADH4	1.346567	0.140371
RNU6-50P	1.346567	0.140371
SNORD116-6	1.346567	0.140371
RPL3P6	1.346567	0.140371
AC092839.1	1.346567	0.140371
MIR3939	1.346567	0.140371
NA	1.346567	0.140371
AL359736.1	1.346567	0.140371
CXCL12	0.145173	0.140353
MSH4	0.711484	0.140328
RNF34	0.047024	0.140249

GABRA5	1.346534	0.140246
AL590723.1	1.346534	0.140246
RPSAP76	1.346534	0.140246
LINC02060	1.346534	0.140246
NA	1.346534	0.140246
RNF225	1.346534	0.140246
AC008121.1	1.346534	0.140246
ZNF232	-0.06509	0.14024
HSP90AB3P	-0.19103	0.140235
ELOVL1	0.054732	0.140219
RFC1	-0.04262	0.140172
NAA20	0.047348	0.140161
HNRNPA0	0.037732	0.140156
MAP3K20-AS1	-0.17082	0.140152
GNA14	0.508651	0.140137
GNG12-AS1	0.190595	0.140128
SMIM2	-0.41831	0.140124
RASA1	0.042607	0.140092
BANF1	0.034234	0.140077
SIKE1	0.047747	0.140037
UBR3	-0.05154	0.14002
KCNH7	-0.43662	0.14
SLC29A4	0.109915	0.139971
AP001462.1	0.364724	0.139961
TRIM7	0.193232	0.139958
LZIC	0.058864	0.139938
TAPT1	0.055129	0.139924
ARPP19	0.034638	0.139923
RHOA-IT1	0.66204	0.139894
RPS26P13	-0.67037	0.139893
AC026471.3	-0.83713	0.139873
PCBP4	0.044659	0.139869
ATP13A1	-0.05067	0.139852
NA	0.10055	0.139839
TNFRSF9	0.117163	0.139818
RTTN	-0.0685	0.139811
CALM2P3	-0.59844	0.139806
NA	-0.04545	0.139806
AC104793.1	0.198759	0.139777
ZNF627	-0.06417	0.139773
LINC01224	-0.089	0.139743
AC006023.2	0.593213	0.139735
AC005104.1	-0.05973	0.139684
INSM2	0.197008	0.139678
SRP9P1	0.235713	0.139627
SRRD	-0.0479	0.139622
UTP18	0.040736	0.139603
NA	0.39412	0.139587
IGHV3-38	0.284519	0.139567
TEX21P	-0.45488	0.139456

SAMD9	0.060974	0.139416
FAM89B	-0.11102	0.139408
TBCB	0.043053	0.139393
CCN4	-0.52697	0.139359
AC008443.6	-0.59114	0.13935
ANK3	0.103762	0.139342
KALRN	-0.064	0.139339
DNAH2	-0.50256	0.139338
ATXN7L3B	0.040063	0.139322
AC008736.2	-0.23421	0.139319
STX17-AS1	0.670172	0.139272
CHURC1	-0.06708	0.139253
TATDN2	0.051981	0.139248
AC005009.1	-0.35425	0.139191
KIAA0100	-0.03687	0.139164
AC145285.2	-0.32662	0.13916
NA	0.714381	0.139111
CHST12	0.053271	0.139103
CDC123	0.037445	0.139062
CERS6	-0.04958	0.139043
IL31RA	-0.39315	0.139006
MAD2L2	0.053478	0.139002
NDP	-0.25202	0.138947
BCL2L12	0.058454	0.13893
RCBTB2	-0.06918	0.138907
PPL	0.212573	0.13889
PRKXP1	0.433848	0.138813
NBDY	0.060012	0.138767
C8orf34	0.989319	0.138755
CIB2	0.060811	0.138746
TBXAS1	0.153717	0.138744
RTCA	-0.05268	0.138729
UQCRHL	0.124783	0.138717
DNAJC6	0.044313	0.138711
AC093503.1	-0.24028	0.138703
CSNK2B	-0.07232	0.138696
MED13	0.036945	0.138679
ZNF44	0.095283	0.138679
BCRP1	0.478074	0.138659
AC022150.1	0.253332	0.138652
AC104506.1	0.171871	0.138598
AL078581.1	0.426076	0.13856
LINC02601	0.551276	0.13856
TRMT112	0.039948	0.138526
VTI1B	-0.03907	0.138509
AC093627.4	0.145047	0.138504
AC124319.3	0.085929	0.138487
MIF4GD	-0.05752	0.138482
CYP2R1	0.061933	0.13848
KIF25	0.355145	0.138458

PRH2	0.931595	0.138407
CDCA3	-0.05724	0.138402
SEMA6A-AS2	-0.43922	0.138379
ZNF765	0.087573	0.138377
AC010186.1	0.539782	0.13837
NA	-0.11097	0.13832
CLASP1	-0.0449	0.138305
EML4	-0.03602	0.138299
LOXL4	-0.12663	0.138287
VEPH1	0.127787	0.138264
CYTH2	0.052514	0.138248
FAR1	-0.04773	0.138243
AC016027.5	0.61316	0.138199
NA	0.942869	0.138188
RNU6-341P	0.942869	0.138188
WDR62	-0.06278	0.138153
ATOH7	-0.45134	0.138117
CCN5	0.589199	0.138101
AL139424.1	0.197796	0.138097
C7orf50	-0.05244	0.138074
AP001619.1	-0.39358	0.138064
RF00422	0.70144	0.138005
SRRM3	0.050361	0.13799
KIF15	-0.05095	0.137968
SNORA72	-0.26748	0.137925
TMEM98	-0.03688	0.137922
MXRA5Y	-0.91672	0.137901
AL035461.1	-1.07419	0.137901
LINC00460	-1.07419	0.137901
HMGN2P40	-1.07419	0.137901
AC007161.2	-1.07419	0.137901
ABCF3	-0.04684	0.137897
PRDM10	0.059214	0.137877
RIPK1	0.048181	0.137875
DNAAF4	-0.22301	0.137842
SV2A	-0.04672	0.137836
AC008434.1	-0.51167	0.137831
ZYG11A	0.187024	0.137815
MMP14	-0.18461	0.137787
DDX24	0.034193	0.137782
WFS1	-0.07468	0.137713
RMI2	0.063767	0.137698
NA	-0.1221	0.137643
SCARB1	0.054454	0.137631
AP001468.1	0.434613	0.137625
NA	0.920606	0.137621
AC012236.1	0.920606	0.137621
NA	-0.30885	0.137607
HERC2P10	-0.6035	0.137592
TRMT10A	-0.10019	0.137551

NA	-0.19472	0.137522
TTLL2	0.353486	0.13752
HSPA9	0.033108	0.137515
TNFRSF1B	-0.20207	0.137485
Z69720.1	0.40058	0.137478
SETD1A	-0.05384	0.137468
RPL39P36	-0.4818	0.137423
AC110597.3	0.075944	0.137417
PAOX	-0.111	0.137392
PAN2	-0.05204	0.137381
AC105446.1	-0.98398	0.137374
AC010531.7	0.20615	0.137367
AL133255.1	0.602834	0.137359
CCL21	0.221547	0.13734
CDH22	0.353993	0.137328
TBX2-AS1	0.056281	0.137326
CCDC96	-0.17267	0.137282
CYP21A1P	-0.39989	0.137279
SKA1	-0.06655	0.137275
EIF2S2P4	-0.28324	0.137251
PNO1	0.046624	0.137208
DCHS1	-0.05591	0.137201
AP005062.1	-0.52077	0.137197
MMUT	0.054092	0.137186
AL669983.1	1.004527	0.137165
NA	1.004527	0.137165
NA	1.004527	0.137165
CCDC144NL-AS1	-0.03902	0.137162
ABHD10	0.066719	0.137143
AC136475.2	0.337327	0.137124
NA	0.932646	0.13712
HMGB1P31	0.932646	0.13712
HEATR1	0.03647	0.13711
AL358176.4	1.012786	0.137091
UBE2D2	-0.04337	0.137073
TDRKH	0.051367	0.137037
ABAT	-0.04491	0.137021
NA	-0.48036	0.136947
RARRES2P6	0.738403	0.136927
LARP1B	0.066585	0.136906
ZXDA	0.145046	0.136896
STX18	-0.05855	0.136819
AP003392.4	-0.21735	0.136812
NA	-0.49454	0.136802
ADAM23	0.071733	0.136774
ANGPTL3	-0.63107	0.136769
CSNK2A1	0.03741	0.136754
RABGAP1L	0.066809	0.136733
AC022968.1	0.696869	0.136723
AP006621.1	0.079004	0.136696

RNASEH2B-AS1	-0.28003	0.136695
CTBP2P8	0.594573	0.136685
CDC27	0.043359	0.136664
DECR2	0.080042	0.136636
TENM1	1.000518	0.136614
TNFSF14	1.000518	0.136614
AL512303.1	1.000518	0.136614
AC105914.2	1.000518	0.136614
AC100788.1	0.605558	0.136592
TCAIM	0.05413	0.136592
AC084026.2	-0.88437	0.136556
COL6A4P2	-0.73288	0.136541
AC083843.3	0.104802	0.136525
PARD6G-AS1	-0.17946	0.13652
TTC17	-0.03577	0.136509
C14orf93	-0.07038	0.136497
NSD2	-0.03967	0.136497
AP002449.1	-0.08892	0.13645
NMU	0.130717	0.136362
ERVK9-11	0.161876	0.136357
SERINC2	0.067664	0.136357
HSD17B12	0.049906	0.136346
SPRED3	0.141797	0.136341
HNRNPA1P10	0.202296	0.136266
SPAG1	-0.08602	0.136248
CHRM1	0.204528	0.136243
AL355483.3	-0.74787	0.136235
AC010761.4	-0.29069	0.136234
MIR133A1HG	-0.76509	0.136191
EIF2AK4	-0.04588	0.136187
RNU6-481P	0.634684	0.136179
CEP97	-0.05514	0.136176
AC087071.2	0.091761	0.136174
FAM192BP	0.629116	0.136103
AC013489.1	0.746582	0.136097
MCM5	-0.05067	0.136093
RUNX1T1	0.112555	0.136068
AC010997.5	0.447631	0.136066
SMG8	0.048205	0.136039
GPR108	0.063659	0.136036
IL10RB-DT	-0.09718	0.136024
MAP3K12	0.053417	0.135993
PLCG1-AS1	-0.06582	0.135945
TMEM190	-0.4296	0.135911
MICU1	-0.04773	0.135883
RBM14	-0.05051	0.135851
CXXC5-AS1	-0.25178	0.135843
SLC16A9	-0.06314	0.135837
MTAP	-0.04724	0.135837
FBXL19	-0.04346	0.13583

AC007009.1	0.326169	0.135819
IMPACT	-0.05056	0.135817
AC004112.1	-0.29474	0.135787
PHF21A	-0.04154	0.135778
FRMD4A	-0.04775	0.135776
NRM	-0.06767	0.135755
NA	0.30251	0.135738
ZNF143	0.054219	0.13571
RPSAP47	0.239005	0.135682
NAGA	-0.07708	0.135623
NA	-0.1258	0.135609
SNORD104	0.161678	0.13559
DUSP4	0.049112	0.135588
APLN	-0.32733	0.135573
CLPTM1L	-0.0369	0.135566
U91328.2	-0.28913	0.135557
LINC02293	0.482633	0.135526
NA	-0.05285	0.135466
AC138305.1	0.786521	0.135455
PDE1C	0.200254	0.135441
RIMKLB	-0.04095	0.135399
CRTC3-AS1	-0.18983	0.13539
CLDN5	-0.30497	0.135382
AC016559.2	0.997743	0.135366
FAM47E-STBD1	0.997743	0.135366
REEP3	-0.04852	0.135337
PRMT5	0.039452	0.135232
SARNP	0.13247	0.135229
AC021087.3	-0.23553	0.135222
SLC9A3	-0.12899	0.135198
AL049780.1	0.06786	0.135195
PRRG4	-0.28536	0.135188
URB1-AS1	-0.10113	0.135185
LINC00685	-0.28892	0.135163
AC015987.1	0.736834	0.135157
PDE4D	0.071403	0.135141
MAGED1	-0.03724	0.135132
CYP11A1	-0.61537	0.135127
PDIA2	-0.20501	0.135051
RN7SL535P	0.309556	0.134953
AL353801.3	0.49795	0.134936
NEDD1	-0.04806	0.134924
RAN	0.037934	0.134918
NDUFS1	0.035881	0.13489
TCTN3	-0.04947	0.134876
AURKC	0.465273	0.134818
AC012651.1	-0.66334	0.134785
ZNF517	-0.06079	0.134764
RBBP8	0.048654	0.134751
DCSTAMP	1.07476	0.134726

AC067942.2	1.07476	0.134726
AC084757.1	1.07476	0.134726
RRM2	-0.05967	0.134725
SPNS1	-0.23148	0.134697
ZFP62	0.045228	0.134675
AGAP2-AS1	0.052028	0.134672
GAPVD1	0.042152	0.134665
NA	-0.87471	0.13465
MAK16	0.042939	0.13462
NHLRC4	0.16791	0.13461
STARD13	-0.09453	0.134607
AC007448.3	-0.42855	0.134591
ZSCAN30	-0.06932	0.134569
RNU6-652P	-0.51413	0.13456
TMEM86A	0.080832	0.134549
C16orf58	0.040957	0.134531
ARHGEF6	-0.10663	0.134527
ITGB1	-0.03738	0.134496
AC020659.2	0.111959	0.134449
DGKD	-0.05582	0.134448
ARHGAP21	0.044834	0.134436
CHTOP	0.034703	0.134383
LTB4R	-0.07162	0.134367
RBM22P2	-0.75674	0.134355
RNU4-14P	-0.75674	0.134355
PIGM	-0.04444	0.134329
SNTB2	-0.0427	0.134316
AC005726.2	-0.04558	0.134302
AC020922.2	-0.09919	0.134281
AL928711.1	-0.40239	0.134278
HGC6.3	0.727284	0.13427
SGO1	0.075933	0.13426
FAM221B	0.989515	0.134253
NA	0.989515	0.134253
AC011472.2	0.989515	0.134253
SUMO1P3	0.317967	0.134249
TNFRSF10C	0.307457	0.134248
EMP2	-0.06048	0.134206
LINC00642	0.301074	0.134191
FBXO15	-0.18411	0.134125
ASAP3	-0.06654	0.134112
TRIB1	-0.06511	0.134091
RPL36A	0.065077	0.134087
RN7SL268P	-0.35322	0.134059
FKRP	-0.04324	0.134055
ST6GAL1	-0.05245	0.134042
FAM49B	0.041058	0.13403
AL049873.1	0.270063	0.134023
COPA	0.032507	0.134018
AC099066.2	0.779427	0.133999

NA	0.145511	0.133993
PAXX	-0.05414	0.13399
NCOA3	0.050033	0.133986
SMPDL3A	-0.65727	0.133965
SLC12A5	-0.12068	0.133961
CDC23	0.045137	0.133959
DAGLA	-0.06614	0.133954
NA	-0.20843	0.133953
PAX8	0.381671	0.133947
AP001803.2	0.157071	0.133933
GFOD1-AS1	0.28875	0.133916
JPH4	-0.05785	0.133902
RIDA	-0.0723	0.133897
AC011498.6	0.080108	0.133892
NA	0.036865	0.133826
MAP2K4	-0.04604	0.133814
P4HA1	0.058499	0.133794
PDHX	0.049901	0.13378
ZBTB17	-0.05397	0.133768
ALMS1P1	-0.32488	0.133749
MRPS10	-0.04949	0.133746
MYOT	-0.19377	0.13372
SCN2B	0.747536	0.133681
SALL1	1.066165	0.133653
LY86-AS1	1.066165	0.133653
NA	1.066165	0.133653
TRAPPC3L	-0.65812	0.13365
ZNF7	-0.04653	0.13364
MRPS12	0.053122	0.133596
DEFB124	0.853818	0.133595
MED9	-0.06735	0.133575
MYOM1	0.322306	0.133558
LRP3	0.050151	0.133544
SELPLG	0.434714	0.133519
AC008555.4	0.299258	0.133514
NPM1P29	0.354841	0.133495
DEDD2	-0.06056	0.133461
ZNF525	0.087585	0.133458
SLC23A1	0.264298	0.133399
NSRP1	-0.06661	0.133386
CCDC114	0.261799	0.13332
RANGAP1	0.035526	0.133283
C4orf33	0.103394	0.133281
CENPJ	0.051407	0.133254
TMEM17	0.117144	0.133249
PARP14	-0.05876	0.133247
THAP8	0.101212	0.133247
TAF12	0.056235	0.133211
AC005253.2	0.056632	0.133176
EFNB1	0.072451	0.133122

WDR82	0.033886	0.133098
CHTF18	0.04978	0.133059
SRFBP1	-0.06954	0.133058
VN1R108P	0.460265	0.13304
NA	-0.35417	0.133011
SCML2P2	-0.48241	0.132904
SMAP2	-0.05352	0.132893
BET1	0.05266	0.132842
AC073343.1	-0.15602	0.132804
SMARCA4	-0.04368	0.132792
HDAC3	-0.03938	0.132773
IL4	-0.99541	0.132762
NA	-0.99541	0.132762
RN7SL724P	-0.99541	0.132762
AC091965.1	-0.99541	0.132762
AC116036.2	-0.99541	0.132762
AL359710.1	-0.56631	0.132713
RNVU1-15	0.798831	0.132677
CASC3	-0.04071	0.132642
MIR302C	0.798227	0.132636
AC090150.1	0.556705	0.132595
DYNLL2	0.034641	0.132592
AC099778.1	-0.13041	0.132591
AC016769.2	0.587456	0.132585
AP000553.2	0.450285	0.132581
AC004706.1	-0.57073	0.132578
ZNF674-AS1	0.107531	0.132552
MTX2	-0.04896	0.132533
TPST2	-0.07156	0.132484
LINC01237	0.165127	0.132467
NA	0.624785	0.132449
TSEN2	0.062896	0.132433
MAPK8IP2	-0.04685	0.132398
H3F3AP4	0.040008	0.132369
ACKR1	-0.40422	0.132367
EHBP1L1	-0.05392	0.132361
NPL	0.081099	0.132349
PQLC2L	0.220404	0.132347
AC018761.2	0.049151	0.132339
GRIP2	0.087108	0.132335
SERPINA10	-0.38118	0.132316
EXO1	0.052903	0.132284
ARL17A	-0.09396	0.132256
DNASE1	0.037243	0.132223
TTK	-0.05997	0.132116
Z99774.1	0.061152	0.132087
OLMALINC	0.095988	0.132074
SNORD19	-0.3559	0.132073
ZNF79	-0.0875	0.132056
CD200	0.10393	0.132031

AC003956.1	0.540921	0.132023
AC016888.1	0.07529	0.131939
AC022182.3	0.735936	0.13192
NFIB	-0.03444	0.131891
PUDP	-0.08383	0.131856
PRDX4	0.046982	0.131854
PRKAA2	-0.08545	0.131814
ZNF592	-0.05245	0.131783
CEP85L	0.08488	0.13178
AGER	-0.11967	0.131761
RNY4P10	0.364607	0.131744
KLB	0.211455	0.131725
AC080013.3	-0.67345	0.131697
RAB39A	0.087699	0.131693
RAB11FIP1P1	0.087713	0.131682
TMEM68	0.064809	0.131662
ABCB1	0.044651	0.131659
HDAC4	-0.05489	0.131654
LINC01285	0.304045	0.131642
TENT2	0.041452	0.131631
SNX15	-0.20714	0.13162
LRTOMT	0.051431	0.131602
C3orf62	-0.06147	0.1316
SGTB	-0.06269	0.1316
NA	-0.32015	0.131552
TNKS2-AS1	-0.50632	0.131535
CCDC171	0.102926	0.131531
PRKAR1A	0.031838	0.13151
NT5M	0.088615	0.13149
CCDC6	0.038711	0.131474
MRPS9	-0.05687	0.131467
NA	0.31245	0.131464
SCCPDH	-0.04233	0.13145
SNHG20	0.055264	0.131449
DHCR7	0.055022	0.13141
L34079.3	0.117731	0.131381
AC008764.2	0.179754	0.131354
PPP1R2B	-0.40964	0.131353
GCFC2	0.044467	0.131277
AC141586.1	0.101992	0.131274
AC103810.1	-0.48127	0.131267
PDE11A	0.21449	0.13124
MRTO4	0.03784	0.131233
AC010731.2	-0.32408	0.131183
ZNF583	-0.10781	0.131183
AC068831.1	-0.09174	0.13117
NBEA	0.046987	0.131167
NA	-0.1972	0.131162
RAD17	-0.04205	0.131159
AC010618.3	-0.40773	0.131157

DNAJC21	-0.05293	0.131156
NA	0.391746	0.131151
RASA4DP	0.130071	0.131123
VASH1	-0.05282	0.131116
LINC00163	0.679374	0.131107
NA	-0.68902	0.131104
AC069061.2	-0.37733	0.131094
AKAP10	-0.04565	0.131067
AC105402.1	0.729684	0.13106
FAM171A2	0.060628	0.131022
AL356292.1	0.781438	0.130983
AL139423.1	-0.10568	0.130982
BRAT1	-0.04174	0.130972
HMCES	-0.05094	0.130968
MRPL27	0.042581	0.130963
NA	0.039244	0.130943
GPR182	-1.07001	0.130932
RNU6-484P	-1.07001	0.130932
CICP5	-1.07001	0.130932
GAPDHP33	-1.07001	0.130932
AC100850.1	-1.07001	0.130932
UBE2Q2P2	-1.07001	0.130932
AL450270.1	-1.07001	0.130932
SH2D7	0.109901	0.130893
NA	-0.22138	0.130888
ZNF263	0.044724	0.130884
AC015982.1	0.187364	0.130855
NA	-0.25065	0.130854
PRKD3	0.03651	0.130835
PPIL1	0.042333	0.130831
SH3BP1	0.069348	0.13082
TMEM45A	0.061742	0.13079
NA	0.764427	0.13079
AC040174.1	0.764427	0.13079
DCTN4	0.035365	0.13079
NA	0.629356	0.130789
RPL22P24	0.200272	0.130735
DNLZ	0.130259	0.13073
GLRX5	-0.03887	0.130724
ITGA6-AS1	-0.58997	0.130699
PPP1R26	-0.04557	0.130653
RAB6B	-0.06562	0.130651
AL139289.1	-0.08375	0.130638
METTL2B	0.049244	0.130622
CIDEB	-0.05311	0.130618
NIPA1	0.040788	0.130608
FTH1P2	-0.54483	0.130604
HSF1	-0.04211	0.130589
OSBPL1A	0.048101	0.130545
NA	-0.25671	0.13054

UPF3AP1	-0.46755	0.130512
PSMG2	0.045605	0.130498
CDC42SE1	0.034176	0.130482
RAB40AL	-0.43528	0.130449
AC012123.1	-0.43528	0.130449
CYP2T1P	0.384181	0.130423
AC007879.2	-0.79091	0.130417
AC096576.2	-0.40799	0.130377
GPER1	0.079387	0.130351
ORC4	0.040758	0.13035
APBB3	0.059871	0.130347
AC005696.1	0.238546	0.13031
SEC23IP	-0.04164	0.130307
FLNA	-0.04114	0.130295
ZNF790-AS1	0.089903	0.13026
FER	0.056377	0.130259
NSFP1	-0.79554	0.130216
COL22A1	-0.31901	0.130129
RBBP6	-0.03863	0.130115
AC023632.2	-0.31002	0.130097
DMXL2	0.041814	0.130009
AC025178.1	0.586013	0.130008
SAA1	0.724697	0.129989
FAM200A	0.05373	0.129976
SRD5A3-AS1	-0.11037	0.129941
AL136309.2	-0.52398	0.129931
SLC18A1	-0.08403	0.129926
MIR199A2	0.440166	0.129913
ERVFRD-3	-0.51493	0.129872
SCAPER	0.079859	0.129851
TBC1D4	-0.05111	0.129843
ZC3HAV1L	0.060284	0.129829
AC104083.1	-0.0983	0.12982
NA	0.763816	0.129786
AL356599.1	0.076003	0.129785
AC090877.2	-0.09578	0.129773
ZNF77	-0.08509	0.129712
NSUN5P1	-0.04908	0.129699
KLF7-IT1	-0.72417	0.129686
NA	-0.13742	0.129669
ADARB2	-0.32516	0.129601
AC098820.2	0.213174	0.129589
ALDH2	-0.04755	0.129558
C9orf129	0.125078	0.129558
PAQR3	-0.04207	0.129548
MBOAT7	0.065615	0.129532
AC018413.1	-0.38985	0.129503
SMIM8	-0.09186	0.129474
RPSAP17	0.69231	0.129437
SNHG28	0.232054	0.129434

AL121929.2	0.287028	0.129421
AC006460.1	0.068072	0.129412
CHUK	0.044826	0.129393
NA	0.253669	0.129383
CDC42EP2	0.096509	0.129373
AC106794.1	-1.06051	0.12937
AC002558.3	-1.06051	0.12937
AC008750.3	-1.06051	0.12937
CACNA2D1	0.04111	0.129358
TTC28-AS1	0.063349	0.129347
IMPG1	0.335041	0.129319
SIN3A	-0.03739	0.129301
AC073842.2	0.166524	0.129203
BAIAP2L1	0.087573	0.129201
LINC00926	-0.14811	0.129159
SLC25A41	0.552448	0.129152
TREH	0.079003	0.129148
AC091304.3	0.56196	0.129093
ZBTB5	0.040741	0.129032
BCCIP	0.045243	0.129031
BCLAF1	0.042473	0.128976
LINC01801	0.189555	0.128961
SQOR	-0.40211	0.128953
MYO18B	-0.22182	0.128949
SLC35F1	0.630795	0.128943
AP001619.2	0.630795	0.128943
CDKN2C	-0.05077	0.128938
SYNE3	-0.10417	0.128882
GRXCR1	0.515811	0.128868
EIF4E3	0.096756	0.128832
TGIF1	0.069587	0.128816
NA	0.213668	0.128781
ZKSCAN3	-0.076	0.12876
ZNF875	-0.04289	0.128747
ZNF500	-0.0602	0.128738
NUBPL	0.071154	0.12871
MIR1180	-0.83847	0.128708
INE1	-0.15432	0.128686
AC233280.2	-0.29049	0.128666
NANOGNBP3	-0.37692	0.128595
CDRT4	0.422065	0.12859
ESYT2	-0.04246	0.128565
L3MBTL2	-0.06238	0.128552
NA	-0.12543	0.12854
EMC10	0.036359	0.128489
GRIK4	0.196714	0.128469
ASCC1	0.04793	0.12846
LINC00174	-0.09667	0.128456
MYB	0.079398	0.128436
AP001412.1	-0.17602	0.128416

PPFIBP1	0.046086	0.128414
GRAMD1C	-0.27665	0.128401
CABLES1	0.079748	0.128398
TTC30B	-0.07971	0.128397
RF00019	-0.73244	0.128376
AC112487.1	-0.75695	0.128327
COQ4	-0.04691	0.128235
IARS2	-0.03347	0.128231
CLN6	0.051918	0.128223
LRRC14B	0.890251	0.128221
MIR4449	0.890251	0.128221
NA	-0.56971	0.128198
SELENOM	0.051263	0.128166
PARP2	-0.05215	0.128158
NA	-0.14336	0.12814
AL512622.1	-0.26895	0.128088
THAP11	0.039953	0.128086
CDK3	-0.21983	0.128056
LRSAM1	-0.05358	0.128
ZNF280D	0.049227	0.127961
LEMD2	0.045671	0.127958
AKAP3	0.521809	0.127947
SLC6A11	-0.10321	0.127919
VN1R54P	0.341022	0.127892
NYNRIN	0.093258	0.127875
AC009245.1	0.213153	0.127869
NBR1	0.036161	0.127868
UNG	0.035386	0.127854
RC3H2	0.038054	0.127852
UCP3	-0.12718	0.127846
LINC01545	0.606047	0.127839
ZNF503	0.067178	0.127829
CCDC25	-0.043	0.127817
LXN	0.095724	0.127807
NA	0.521668	0.127784
AL589765.4	-0.12446	0.12778
RIBC1	-0.23886	0.127769
HM13-IT1	-0.21963	0.12772
HECW1	0.652421	0.127697
NA	0.733893	0.127642
AL954705.1	0.733893	0.127642
WDR47	0.050761	0.127626
RAB31	-0.04246	0.127516
CHMP3	0.043059	0.127515
NA	-0.58863	0.127502
CLDN23	-0.76099	0.127477
DHDDS	0.061401	0.127455
AL451042.1	0.958732	0.127453
AC074194.1	0.474068	0.127451
YBX1	0.028166	0.127396

TRDMT1	0.081257	0.127388
KIAA0391	0.101808	0.127371
HSPA14	0.041646	0.127362
MORC3	-0.04087	0.127312
PDSS1	0.057615	0.127282
AC011383.1	-0.78526	0.12727
PCMTD2	-0.03513	0.127261
AC016168.2	-0.35028	0.12726
ZNF549	0.062892	0.127236
EEF1B2P1	0.665417	0.127181
CSNK1G2	0.03618	0.127138
AC127496.1	0.173575	0.127127
PICALM	0.035692	0.127076
CASD1	0.039464	0.127059
P3H3	0.054031	0.127006
EPN2-AS1	0.48864	0.126942
AC232271.1	0.139791	0.126935
AC078993.1	0.238522	0.126892
SUCLG1	0.043013	0.126879
PCNT	-0.04057	0.126862
SRR	-0.04232	0.126862
AC108488.1	0.118397	0.126834
FAM133DP	0.292147	0.126829
TCOF1	-0.03731	0.126802
PTGDS	-0.14498	0.126769
NA	-0.49225	0.126762
AC037487.3	0.640074	0.126753
RASSF4	-0.06792	0.126731
AP001178.3	-0.22908	0.126634
AC096719.1	0.648135	0.126624
RF00019	-0.43393	0.126613
AC013403.2	-0.28786	0.12653
TIGD4	0.153749	0.126515
NSF	0.043549	0.126453
RMND5A	0.0387	0.126422
ZFHX2	0.075443	0.126407
ARHGAP30	-0.6471	0.126368
NA	0.344278	0.126322
NAIP	0.137712	0.126297
PLEKHA8	0.035685	0.126256
AP001970.1	0.776014	0.126247
DNAJC8P1	0.463338	0.126244
RALGPS1	-0.08876	0.126238
STIMATE	0.26975	0.126236
NASP	-0.03951	0.126215
CBX7	-0.13015	0.126207
TRNP1	-0.15403	0.126196
AC116666.1	0.282469	0.126187
OLFML2B	-0.21905	0.126144
CASP6	-0.08036	0.126129

NECAB2	0.192202	0.126115
GOLGA8R	0.641723	0.126064
DDIAS	-0.06049	0.126051
COX5A	0.038309	0.126036
MECR	-0.05658	0.126036
ZNF414	0.085844	0.126022
OR7E14P	0.574309	0.126005
AP001267.1	0.048481	0.126005
AC007842.1	0.127118	0.125986
NA	-0.31923	0.12596
AC009413.1	0.759169	0.125888
DNAJB13	-0.24644	0.12584
SYT16	0.337088	0.125823
HMGB3	0.041991	0.125817
MLYCD	-0.06895	0.125806
NA	0.176926	0.125733
NA	-0.67613	0.125731
DNALI1	0.047419	0.125677
KCTD2	0.03598	0.125677
LINC01088	0.336173	0.125661
GALNS	-0.06744	0.125656
ESYT1	-0.04563	0.125639
CCDC58	-0.06981	0.12563
AC004890.3	-0.28148	0.125583
AC129507.4	-0.44756	0.125571
FAXC	0.048926	0.125546
OXCT2	-0.33401	0.125437
KDM1A	-0.0365	0.125429
RAB20	-0.12861	0.125416
TTC32	0.069432	0.125415
SEC61A1	-0.03001	0.125316
AC016252.1	-0.55408	0.125286
CNOT8	-0.03525	0.125277
ST5	-0.05606	0.125244
UQCRFS1	-0.04134	0.125205
MTCO3P39	-0.7559	0.125201
RPL12P44	-0.92341	0.125192
ALG1L7P	0.271232	0.125175
DOT1L	0.059638	0.125164
MTCP1	0.185706	0.125155
SLC37A4	0.051515	0.125153
CREBL2	0.046445	0.125121
EHMT2-AS1	-0.06111	0.125043
AC046130.1	-0.14672	0.124974
AC005162.3	-0.24828	0.124939
TP53I13	0.058749	0.12486
AL645949.1	0.635754	0.124832
FAM241A	0.202205	0.12483
OPN1SW	0.107649	0.124823
NA	0.619981	0.1248

AC113404.3	-0.25591	0.12479
BLOC1S4	-0.05825	0.124758
TSSC2	0.251425	0.124755
KIF5C	0.034621	0.124746
ABCD4	-0.0474	0.124741
GFOD2	-0.04271	0.124738
AL132800.1	0.435829	0.124657
LINC02381	0.05828	0.124638
CENPX	0.036352	0.124626
MCTS1	0.042642	0.124622
PKMYT1	-0.05136	0.124592
CEACAM19	-0.28267	0.124569
MIR4253	0.225574	0.124567
AC073896.3	0.205176	0.124564
PDP2	-0.06216	0.124556
MARCKSL1	-0.03538	0.124543
ZC3H12C	-0.08247	0.124515
AC073389.1	0.25407	0.124494
AC069185.1	-0.5795	0.124425
AMD1	-0.04066	0.124408
KPNB1	-0.02928	0.124371
AC127502.1	0.175133	0.124333
SLC45A2	-0.46178	0.12432
ZNF84	0.169345	0.124267
TMEM35A	0.054578	0.124259
ACTR10	0.043729	0.124213
UMAD1	0.058332	0.124179
RFPL4B	-0.66177	0.124156
IFI35	0.220564	0.124156
MMAB	0.045908	0.124156
AC009171.2	-0.59126	0.12415
PCBP1-AS1	0.03331	0.124128
CDK6	-0.03123	0.124093
OPA1-AS1	0.121014	0.124068
MCTS2P	0.135153	0.124062
GRN	-0.04583	0.124041
AC023794.2	-0.4849	0.124038
AL049597.2	0.272136	0.124021
SEMA3B	0.125909	0.123988
ZNF445	-0.03496	0.123965
AC021483.2	-0.48555	0.123951
WDR34	0.038564	0.123925
AL021707.7	-0.50462	0.123918
ARHGEF18	-0.0576	0.123914
PYY2	-0.5924	0.123864
PTGES3P4	-0.92101	0.123822
AL031658.1	0.251197	0.12382
SNORD19C	0.228341	0.123773
TCEAL9	-0.04515	0.123741
RGL1	-0.04336	0.123722

RF00019	-0.38336	0.123697
NA	-0.16019	0.12369
SPIN3	-0.06291	0.123686
AIG1	0.06252	0.123625
GEMIN8P4	0.210819	0.12357
PTRH2	-0.03719	0.123551
NA	-0.66041	0.12355
RNF31	-0.05476	0.123525
AL158151.3	-0.1292	0.123505
NA	-0.09395	0.123497
AC093797.1	0.548196	0.123495
CNTN4-AS1	-0.13102	0.123464
POLR2J4	0.087425	0.123452
RNU4-22P	0.668652	0.123402
XYLT2	-0.05303	0.123391
PPP1R13B	0.079758	0.123376
FKBP1B	-0.11299	0.123338
SLC12A6	0.060856	0.123327
NA	-0.7466	0.123323
PRELID1P4	-0.7466	0.123323
AL670729.2	-0.7466	0.123323
NTRK1	-0.06043	0.123322
PTBP3	0.043116	0.123321
IRX5	-0.11924	0.123302
PGK1P2	0.616192	0.123293
AD000671.3	0.078614	0.12327
NA	0.289947	0.123226
ARRDC2	0.06531	0.123217
SAPCD1	-0.35313	0.123205
AL357992.1	0.562142	0.123198
SYNJ2BP	-0.04355	0.123198
LEMD3	-0.04871	0.123183
GPAT2P2	0.381309	0.123178
LRCH2	0.056687	0.123147
AC008453.1	-0.20984	0.123122
NA	-0.25726	0.123091
AC090510.1	-0.06647	0.123068
EXOC5	-0.03589	0.123052
NA	-0.4335	0.123045
MEF2D	-0.05229	0.123011
CNOT4	-0.04664	0.12301
GNG5	0.039837	0.122987
RNF187	0.046318	0.122976
TNFRSF21	-0.0735	0.122963
UBR4	-0.04262	0.122945
C9orf135	0.25799	0.122943
ADGRE5	-0.07491	0.122935
MAGEA6	-0.03918	0.12293
AC106895.1	0.189034	0.122925
DSTYK	0.048148	0.122914

SLC6A4	0.52946	0.122873
ACTG1P1	-0.55661	0.122871
EP300	-0.0461	0.12286
LINC02609	0.196966	0.122858
NA	-0.2255	0.122835
CXCL8	-0.15629	0.122827
AL592435.2	0.900885	0.12281
ADAL	-0.06307	0.122806
RNA5SP434	0.637489	0.122748
NA	0.746869	0.122736
AC006001.2	-0.19172	0.122736
NA	-0.06471	0.122723
MED28	0.036429	0.122721
NCBP1	-0.03661	0.122672
RHEB	0.035032	0.122654
CES2	0.048793	0.122646
ZNF345	-0.07668	0.122641
AHCYL1	0.03777	0.122637
RANBP17	-0.07668	0.122625
NDFIP1	-0.03256	0.122621
AC083829.1	0.755087	0.122604
TMEM8B	0.067549	0.122583
IPO7P2	0.487623	0.122574
AC009065.6	-0.11428	0.122549
PICK1	-0.06368	0.122546
FTL	0.033089	0.122504
AL359541.1	0.675258	0.122495
LINC01132	-0.81119	0.122452
DCP1B	-0.05746	0.122419
ZNF322	0.059667	0.122404
AC090617.5	0.19722	0.122396
DAPK1	-0.06212	0.12239
RARRES1	0.134974	0.12237
CTDSPL2	0.04131	0.122322
AL592424.1	0.308202	0.122308
AC136475.8	-0.62393	0.122297
BRIX1	0.048976	0.122253
AL390955.2	-0.51107	0.122238
NA	0.444302	0.122228
AC004908.1	-0.17643	0.12221
NA	0.066743	0.122209
PPP3R1	0.038559	0.122181
MYOSLID	-0.41106	0.122166
AC009237.14	0.173895	0.122146
MYL12B	-0.03613	0.122144
ADAP2	-0.29599	0.122139
MFN1	0.038217	0.122128
RPARP-AS1	0.0741	0.122104
MIR3164	-0.60824	0.122095
ACBD6	-0.05846	0.122083

RNA5SP474	0.633596	0.122063
TRIP4	0.054502	0.122029
SETDB1	-0.04023	0.121986
AC104463.2	-0.5848	0.12198
AC007298.2	-0.11297	0.121928
AC114811.2	-0.21492	0.121902
SEPHS1	0.031769	0.12187
AC012462.3	-0.75056	0.121865
ENSAP2	-0.75056	0.121865
EEF1A1P6	0.118874	0.121799
NA	0.035465	0.121774
JTB	0.03438	0.121754
USP4	0.039709	0.12172
AC025575.2	-0.17573	0.121702
KEAP1	0.035476	0.12168
AP001437.1	-0.62458	0.121666
AP002490.1	-0.04433	0.121665
AC026254.2	0.289022	0.121651
THEM4	0.052921	0.12164
FTOP1	0.797073	0.121635
PGGT1B	-0.04596	0.121608
CTNNBIP1	0.048047	0.121604
CCDC120	-0.07791	0.121602
AC010186.2	-0.10743	0.121564
ULK3	0.042435	0.12156
STAT3	-0.04365	0.121542
AP001107.2	-0.03827	0.121511
HIST1H1PS1	0.318131	0.121484
AC103736.1	0.197126	0.121481
AC019185.1	0.669965	0.121473
BRSK1	0.050438	0.121469
ITSN2	0.054523	0.121461
TSPEAR-AS2	0.101007	0.121424
STAT1	0.043995	0.121404
AC096533.1	-0.36299	0.12137
MIR302CHG	0.055331	0.121356
RABGGTB	-0.04102	0.121344
IGHV4-39	-0.24295	0.121343
NA	0.105406	0.121336
STIP1	0.029021	0.121276
ZFP91	0.035585	0.121236
BEX2	0.054679	0.121217
MEF2C-AS1	0.356568	0.121216
GTF2A2	-0.04398	0.121193
PIN1	0.034414	0.121193
ZNF37BP	0.042855	0.121173
NA	0.11677	0.121167
TTC26	0.052621	0.121163
CC2D1B	0.044751	0.121156
KBTBD11-OT1	0.806479	0.121144

TCHP	0.03566	0.121141
TMEM214	0.0357	0.121115
AC012085.2	-0.75464	0.121092
UROS	0.04954	0.121083
GCC2	-0.04552	0.121066
AC022966.1	-0.03728	0.121048
RABL6	0.037594	0.121047
KCNK10	0.265062	0.121035
VPS9D1-AS1	-0.0601	0.121004
EEA1	0.049354	0.120963
PAMR1	-0.36735	0.12096
GAA	-0.10587	0.120957
AKTIP	-0.04905	0.120928
NAGLU	0.062732	0.120911
NAALADL1	0.448519	0.120848
STXBP3	-0.05358	0.12084
TSPAN7	0.119302	0.120774
INSC	-0.22884	0.12077
AGO4	-0.0424	0.120769
NA	0.105012	0.120765
AP005019.1	-0.22306	0.120737
HECTD4	-0.05034	0.120701
LINC02298	-0.30064	0.120697
AC067930.3	-0.90436	0.120689
LDLRAD4-AS1	-0.90436	0.120689
SIGLEC15	-0.59604	0.120678
PUS7L	-0.06281	0.120674
LINC02347	-0.29368	0.120636
EZH1	0.048011	0.120577
AP000879.1	0.054629	0.120548
PKMP3	0.372294	0.120543
HAS2-AS1	0.198453	0.120528
AC083880.1	0.198381	0.12044
ZDHHC12	-0.06033	0.120423
ARHGEF3	-0.09101	0.120423
AGAP2	0.04416	0.120406
AP001496.3	-0.18563	0.120362
CPT1C	-0.03635	0.120361
TTLL5	0.04463	0.120357
AL133485.1	0.154343	0.120328
ALDH6A1	-0.05301	0.120322
TPT1-AS1	-0.07818	0.120316
RBCK1	0.050613	0.120304
CASP16P	-0.86305	0.1203
HEATR3	-0.05033	0.120291
CBR1	-0.03986	0.120256
AGAP10P	-0.35309	0.120254
WDR83	-0.03667	0.120241
RNU6-834P	0.339324	0.120209
IPO4	0.196746	0.120204

MRPS7	0.036777	0.120154
AC016629.2	-0.21011	0.120142
PAH	0.141032	0.120131
SCN4B	-0.63023	0.120112
ZNF638	-0.04206	0.120092
PTPN9	0.03721	0.120086
HSD3BP4	-0.80357	0.120026
GCK	0.167426	0.120016
TTC39C-AS1	0.27112	0.120014
RFNG	0.050495	0.11999
AC012447.1	0.33534	0.119952
CTDP1	0.050897	0.119947
ANKS1B	0.096461	0.119914
ZNF343	0.04453	0.119911
AL356481.1	-0.10639	0.119903
AL442663.4	-0.63794	0.119863
CNIH3	-0.05841	0.119836
FAM104A	0.038219	0.119831
GALNTL6	-0.19123	0.119826
MIR3176	0.297812	0.119761
ZIC1	-0.06528	0.119697
IFT81	0.036982	0.11969
LINC02585	-0.26426	0.119663
AC138393.1	0.618437	0.119655
DPYSL5	-0.03984	0.119636
XPO1	-0.03743	0.119621
TRAPPC2B	0.12796	0.11959
NA	0.107311	0.119581
SLC5A4-AS1	-0.39973	0.119567
COPE	0.039077	0.119528
TNIP2	-0.05115	0.119509
INVS	-0.05329	0.119495
BX322650.1	0.735419	0.119479
RAB11FIP5	-0.06397	0.119459
AL158071.5	0.364103	0.119429
SLF1	0.059481	0.119391
HEATR4	0.105524	0.119381
MNT	-0.05386	0.119379
ZNF529	-0.04108	0.119363
NA	0.202682	0.1193
MDM4	0.035092	0.1193
AC012557.2	0.400615	0.119282
AC084855.1	-0.18812	0.119264
AC083899.1	-0.12117	0.119259
SLC5A2	-0.0393	0.119256
RGS3	0.045504	0.119223
CACYBP	0.038414	0.119211
MTCO1P12	0.121386	0.119198
INHBE	0.497467	0.119173
GPR135	-0.09174	0.119156

AL132639.2	0.419337	0.119124
MEIS1	0.049761	0.119064
RASSF2	-0.09298	0.11905
POLR2M	-0.04705	0.119014
AC108449.2	-0.0906	0.119007
LINC01982	0.617008	0.119005
ZNF266	-0.04017	0.11895
AC010245.2	-0.16895	0.118945
NA	-0.0332	0.118944
AC118282.1	-0.42636	0.118908
AC021224.1	0.110122	0.118888
DNPH1	0.03995	0.118845
USP21	0.039832	0.118839
SNHG30	-0.08093	0.118811
SNX20	0.914353	0.118797
RNU6-1285P	0.914353	0.118797
RAET1K	0.914353	0.118797
AL731537.1	0.914353	0.118797
IREB2	-0.03026	0.11879
AC022098.1	-0.08241	0.118781
SLC1A5	-0.03958	0.118775
SGO2	0.060054	0.118736
AC008667.1	0.295119	0.118732
CCDC116	0.331673	0.11873
TUBA8	-0.2509	0.118704
GATA3	-0.04033	0.118687
IGFBP7-AS1	0.13647	0.118684
CENPA	-0.05796	0.118666
AC136698.1	-0.79864	0.118614
FOXD1-AS1	0.359601	0.118609
RPL39P3	0.106908	0.118598
FAM227B	-0.07446	0.118588
SAV1	0.04622	0.118574
AC113361.1	0.570616	0.118558
HADHA	0.029309	0.118545
ZNF653	0.086852	0.11853
GLA	-0.06281	0.118527
SLC7A4	0.293543	0.118519
PTGIR	-0.17185	0.11851
TSPAN14	-0.04356	0.118439
YWHAZ	0.030764	0.118435
C16orf46	-0.1258	0.118417
CPNE7	0.502819	0.118409
GBA2	-0.03659	0.1184
NTF4	-0.66469	0.118357
AC124856.1	-0.66469	0.118357
HOOK3	0.038943	0.118356
AL138752.1	-0.6472	0.118331
AL139287.1	-0.15244	0.118311
ZNF573	0.081801	0.118306

DENND5A	0.033403	0.118302
VSNL1	-0.09163	0.118294
CMTR2	0.049095	0.118285
ACVR1	-0.04588	0.11826
NOLC1	-0.02799	0.118246
AC138028.4	-0.1138	0.11824
PPP4R1L	-0.10534	0.118222
LRP12	0.041815	0.118209
SENP6	-0.0317	0.118208
SUPT3H	0.084499	0.1182
MESD	0.039969	0.11819
AC098934.3	-0.45721	0.118189
HIST2H2BD	-0.17206	0.118189
MAGED2	0.031755	0.118166
GULP1	0.042287	0.118157
DPYSL2	-0.03247	0.118147
ZNF585A	-0.06546	0.11814
TCERG1	0.031611	0.118122
COPS7B	-0.03629	0.118038
LIAS	-0.07897	0.118012
AL354893.1	-0.56068	0.11801
PROSER2-AS1	-0.66298	0.117987
AC026894.1	-0.61837	0.117977
PTMAP11	-0.28932	0.117975
DNAAF1	0.066356	0.117951
AL135818.1	-0.26931	0.117904
NA	-0.46355	0.117898
DRC7	-0.34852	0.117855
AC008897.1	0.30427	0.117802
COG8	0.037118	0.1178
ICE2	-0.04663	0.117785
B3GAT2	0.047008	0.117741
RPRML	-0.7423	0.117718
LSR	0.163721	0.117712
ZNF807	-0.41497	0.117696
HRK	0.710493	0.117676
ZNF219	0.048917	0.117667
AC012173.1	0.054995	0.117651
LIN28B-AS1	-0.33324	0.117651
PKDCC	-0.05934	0.117645
AL592182.2	-0.44041	0.11758
PPP2CA	-0.03909	0.117569
ANGPTL1	0.313322	0.117561
POLR3GL	-0.05084	0.117554
EXOC7	0.03324	0.117496
USP54	0.045685	0.117494
PPIG	0.042224	0.11749
LINC00963	-0.04722	0.11748
VTI1A	-0.04599	0.117466
CTXN1	-0.0394	0.117439

NA	-0.27602	0.117433
NDUFS8	-0.04701	0.11739
AL357079.1	0.189292	0.117386
NTNG1	-0.0535	0.117336
AC145098.1	-0.20053	0.117244
MTSS2	0.053939	0.117213
BRWD1	-0.03597	0.117169
PPIAL4D	0.933969	0.117139
UBE2L4	0.933969	0.117139
PDHA1P1	0.933969	0.117139
PGM2	-0.04319	0.117101
LINC00870	-0.72369	0.117095
C11orf80	-0.07834	0.117062
P3H1	-0.03772	0.117039
AL162171.3	-0.20681	0.117033
NIPA2	0.032037	0.11703
AC011773.4	-0.37657	0.11703
HEY1	-0.05518	0.116994
DENND5B	-0.049	0.116989
FRG1	-0.03898	0.116989
LITAF	-0.03382	0.11696
CLUH	-0.04008	0.116956
CCNB1	0.041541	0.116945
DDX11-AS1	-0.14685	0.11693
AC022540.1	-0.67349	0.116904
AL512656.1	0.071637	0.116866
BTBD19	-0.06869	0.116854
UNC93B2	0.60636	0.116831
ZDHHC20-IT1	0.60636	0.116831
PTHLH	-0.11778	0.116816
ITCH	0.039832	0.116806
MIR3671	0.524712	0.116749
ZSWIM9	0.085569	0.116738
RAPGEF2	-0.04198	0.116734
AC079329.1	0.123708	0.116678
NA	-0.29271	0.116666
IGHV4-28	-0.57652	0.116656
ITGB6	0.507856	0.116642
CICP20	0.727905	0.116637
AL118558.4	0.727905	0.116637
SMPD3	0.077481	0.116615
RCAN1	0.132389	0.116611
DHRS4L2	-0.08409	0.116547
LOXL1-AS1	-0.34139	0.11652
ADAMTS9-AS2	0.13281	0.116481
SMIM2-AS1	-0.2408	0.116458
AC011476.3	0.147308	0.116451
RASSF8-AS1	0.065301	0.116446
MTMR10	-0.04067	0.116405
MOCOS	0.206469	0.116403

ZKSCAN8	0.032727	0.116367
ARPC5L	0.038636	0.116355
NPDC1	-0.03908	0.116325
CKMT1A	-0.11182	0.116317
MAST2	0.04144	0.116316
LINC01521	-0.06181	0.116293
SHCBP1	-0.07282	0.116292
NDUFA11	0.071914	0.116268
UBL4A	0.046366	0.11625
FAM114A1	-0.06307	0.116242
SERBP1P6	0.55754	0.116237
NCS1	-0.03078	0.116226
GNRHR	-0.22886	0.11622
AC233702.7	-0.42973	0.116204
AL360270.2	-0.25087	0.116198
MTA1	-0.0363	0.11612
METTL8	0.052385	0.116109
UBE2N	0.029573	0.11607
PCDH15	-0.16061	0.116064
DIABLO	-0.05473	0.116057
AC107959.3	0.533184	0.116033
NA	-0.29055	0.115963
GPR18	0.636576	0.115962
AL355490.2	-0.07651	0.115958
NA	0.059922	0.11594
GORAB	0.058533	0.115939
AC005253.1	-0.0642	0.115913
ENTPD1	-0.03563	0.11591
AL160006.1	0.048777	0.1159
ATL2	0.043136	0.115855
EIF4BP3	0.141322	0.115827
C6orf47	-0.04413	0.11582
MORF4L2-AS1	-0.1241	0.115811
LTA4H	0.038371	0.115805
LRP4-AS1	0.177474	0.115791
C4orf19	0.352322	0.11578
SPATA46	-0.5385	0.115719
AC096649.1	-0.11708	0.115701
H1FX-AS1	0.038861	0.115686
NA	0.155718	0.115683
AP000593.3	-0.10465	0.115643
AC003991.1	-0.46275	0.115641
ST3GAL4	0.069812	0.11563
TADA3	0.036082	0.115616
ZBTB22	0.053728	0.115596
RALGAPA1P1	0.211589	0.115579
AC022364.1	0.251793	0.115568
TFB2M	-0.03679	0.115509
DIS3L	-0.04295	0.115503
CCDC150	-0.05783	0.115463

LINC00399	-0.83011	0.115411
TTC3	-0.03161	0.115407
ZNF677	-0.10116	0.115381
IDH1-AS1	-0.39616	0.115379
NA	0.117639	0.115365
TELO2	-0.04601	0.115327
AL157756.1	-0.37619	0.115295
ATRN	0.036069	0.115286
AC022148.1	0.219517	0.115274
ZNF415	-0.13503	0.115271
NA	0.651283	0.115248
PHGDH	0.034616	0.115222
CC2D2B	-0.69256	0.115201
AC022150.4	-0.15742	0.115152
OTUD7A	0.159518	0.115142
AC046176.1	0.322687	0.115142
AL136141.1	0.736319	0.115141
COCH	0.041855	0.115125
HNRNPK	0.028949	0.11512
MORF4L1	0.031441	0.115106
NA	-0.15472	0.115079
B3GNT7	-0.3168	0.115073
AC012170.1	-0.68782	0.115029
NA	-0.09252	0.115016
HDAC8	0.045241	0.114989
WWC3	0.061909	0.114984
DNM2	0.037339	0.114983
NA	-0.28141	0.114933
PET100	-0.11996	0.114919
UBE2A	-0.04632	0.114865
AC092675.1	-0.63699	0.11486
UBOX5-AS1	0.147234	0.114857
CSTB	-0.04069	0.114829
AC004837.3	0.353618	0.114804
NA	-0.22386	0.114784
EME2	-0.03967	0.114776
KRT8P33	0.209012	0.114735
SLCO1A2	-0.14168	0.114723
VWA3A	-0.61151	0.114709
NA	0.450453	0.114695
PLGLB1	0.164077	0.114684
AL121894.2	-0.44094	0.114661
RRN3P1	-0.06208	0.114655
AL354956.1	0.078079	0.114654
NAT16	0.071635	0.114632
TST	0.062816	0.114616
G6PD	0.042924	0.114575
LBR	-0.03667	0.11455
STYXL1	-0.05326	0.114537
RORB	-0.04496	0.114531

SSRP1	-0.02796	0.114489
TATDN2P2	-0.11006	0.114438
AP001033.2	0.349139	0.114379
PHKG2	0.04408	0.114378
CEP55	-0.05024	0.114334
TMEM242	-0.06293	0.114313
NOS2	0.2008	0.114313
LYSMD2	0.062681	0.114306
MRPS25	-0.03089	0.114296
GRB2	-0.0313	0.114275
ART5	-0.70221	0.114268
AC100778.2	0.313242	0.114263
AL590399.1	0.129805	0.11426
ADAM11	0.108713	0.114231
AC005529.1	0.700686	0.114228
KDELR2	-0.03766	0.1142
SRSF2	0.029554	0.114173
AC007785.3	0.398659	0.114158
IMPDH1P8	-0.17105	0.114123
INPP5F	0.043682	0.11412
CDC25B	0.11358	0.114106
MED11	-0.06333	0.114061
NDUFB6	0.047646	0.114009
AC107375.1	-0.09635	0.114004
AC144530.1	0.19768	0.113915
ZNF625	-0.38721	0.113913
YWHABP2	-0.50709	0.113901
ZRANB2-AS2	0.186093	0.113851
KIAA1841	0.057591	0.113844
AC114810.1	-0.44563	0.113839
THEM5	-0.30526	0.113834
ATPAF2	-0.06673	0.113824
TM2D1	-0.04858	0.113817
COL4A3	0.452086	0.113812
RPL12P42	-0.6936	0.113802
Z94721.1	-0.28608	0.113788
AC068790.1	0.758474	0.113785
AC006030.1	0.055574	0.113763
AP000777.2	0.071943	0.113709
RPS10P7	-0.1914	0.113702
ADAMTS7P3	-0.53411	0.113691
TMBIM1	-0.14122	0.113688
AC005324.5	0.14156	0.113635
MSMP	-0.05882	0.11363
RPS26P8	-0.92743	0.113612
OR5BK1P	-0.92743	0.113612
ZNF74	0.041873	0.11361
KIAA2012	-0.48478	0.113569
AC008267.5	-0.12985	0.113558
CCDC102B	-0.09843	0.113538

PDHB	0.036058	0.113537
USP45	-0.05305	0.113501
ZNF883	0.082518	0.113498
NPAS4	0.516482	0.11349
NDC1	-0.04121	0.113485
AC008914.1	-0.06267	0.113455
AL590999.1	-0.22405	0.113453
ARPC1A	-0.02962	0.113431
RAP1GDS1	0.03911	0.113418
ZBTB20	-0.24475	0.113415
FARP1	-0.03687	0.113414
CDC14B	-0.04469	0.113404
PCDHGA9	-0.24413	0.113382
SGK3	0.287654	0.113377
FGFR1OP2	0.04036	0.113335
AL391422.4	0.110903	0.113333
TENT4B	0.049092	0.113311
SLC35F3	-0.09074	0.113286
ANKRD18A	0.329902	0.113281
HADHAP1	-0.54146	0.113262
ZNF551	-0.05765	0.113225
IL17RB	0.0757	0.113212
NA	-0.25067	0.113199
ASF1A	-0.0509	0.11315
ERCC6L	-0.06425	0.113147
EFCAB1	-0.21768	0.11314
ALX3	0.072659	0.113075
SLC51A	0.086624	0.113063
AC009065.3	0.352133	0.113058
MLLT3	0.056278	0.113048
RN7SL444P	-0.59455	0.113
HIST1H1E	0.365716	0.112986
RF00561	0.581169	0.112971
AC124312.2	0.231249	0.112917
GTF3C3	-0.03437	0.112911
TOR1A	-0.03227	0.112895
MED15P9	-0.14465	0.112838
ZNF202	0.062501	0.112815
RASD1	-0.35464	0.112808
SMNDC1	-0.04005	0.112798
POMK	0.055519	0.112753
CRB2	0.139603	0.11273
PFN1P4	-0.55986	0.112706
PYGL	0.074011	0.112678
CHRD	0.265669	0.112664
ELP3	-0.04179	0.112653
DCT	-0.3571	0.112631
TEKT3	-0.44102	0.11262
KCND1	0.095072	0.112612
PANK3	-0.03591	0.112599

AL135744.1	-0.45385	0.112596
NDUFA5	0.039524	0.112558
STX19	0.4543	0.112551
DESI2	-0.03399	0.112541
TLR6	0.212107	0.112524
NRXN1	-0.0831	0.112508
ANKRD13D	0.040168	0.112503
NA	-0.28636	0.11245
AL121992.1	0.343411	0.11245
AC106793.1	0.064215	0.112434
ARHGEF19-AS1	-0.15045	0.112413
AC100774.1	-0.53018	0.112412
CAPN15	-0.04455	0.112402
PLA2G12A	0.048437	0.112384
LINC02691	-0.09549	0.112371
PRR14L	0.035584	0.112349
PPP6R1	-0.03877	0.112344
ANKIB1	0.031406	0.112298
DUS1L	-0.0373	0.112293
BUB3	0.033759	0.112284
PRC1	-0.0387	0.112278
CLPTM1	0.041474	0.112268
BZW2	0.030859	0.112267
HECW2	-0.05542	0.112259
ADCK5	-0.08639	0.112229
DDIT4L	-0.23436	0.112218
DYSF	-0.4249	0.112213
MRPS23	0.035417	0.112202
DPH3P1	-0.14742	0.112173
AC015853.1	-0.17472	0.112145
AC012170.2	-0.07469	0.11214
CCDC115	0.044966	0.112136
AL590666.1	-0.21466	0.112125
SEC31B	0.082781	0.112116
HSPA8P1	0.598447	0.112112
PPP3CA	-0.04182	0.112104
GPX2	-0.41798	0.112104
NA	-0.91785	0.1121
RPS29P3	-0.91785	0.1121
RPS3AP5	0.326163	0.1121
FHDC1	0.150807	0.112086
LIX1L-AS1	0.033177	0.112077
AL513365.1	-0.63541	0.112066
PPIAP19	-0.63541	0.112066
ASNSD1	-0.04483	0.112044
SCUBE2	0.259436	0.112039
PITPNM1	0.046893	0.112036
ELFN1	-0.83962	0.112017
AC092115.3	0.049613	0.112014
ATP5MC1	-0.04162	0.112

TGFB3-AS1	-0.31897	0.111968
NA	0.498596	0.111966
NA	0.690228	0.111949
AC023300.1	-0.7314	0.111936
ZSCAN31	0.081496	0.111878
DDX46	-0.03155	0.111864
AC107302.1	0.477252	0.111863
GIT2	-0.03558	0.11186
HIGD1A	-0.04567	0.111828
NA	0.066237	0.1118
AC005021.1	0.093386	0.111796
PPIA	-0.03098	0.111781
MIR3650	-0.68937	0.111761
AC010175.1	-0.66704	0.111735
U73166.1	-0.14942	0.111699
ZNF276	-0.04034	0.11168
NA	-0.17439	0.111667
FLYWCH1	-0.04803	0.111662
RUFY2	0.034483	0.111651
RAB10	-0.02943	0.111643
IDE	-0.05064	0.111641
RDX	-0.06361	0.111633
CCDC66	-0.05815	0.111608
LINC00674	-0.08374	0.11159
C5orf30	-0.05077	0.11158
TMEM176A	-0.36552	0.111538
PATZ1	-0.03443	0.111515
CARD14	0.098949	0.111515
ADPGK-AS1	0.079541	0.111515
AP005131.3	0.687577	0.111507
MAP3K21	-0.08553	0.111439
UBXN2B	0.033948	0.111428
IPO13	-0.04217	0.111363
AC110079.1	0.069553	0.111355
OPRM1	-0.05209	0.111321
FBXL7	0.058066	0.111315
ZNF701	0.078603	0.111309
PSEN1	-0.03201	0.111302
AL031729.1	-0.41796	0.111296
GOLGA4	0.034912	0.111261
NA	-0.63678	0.111254
LDHA	-0.04311	0.111247
DDO	-0.36181	0.11124
RARA-AS1	0.11448	0.111211
ALG12	-0.04757	0.1112
AL353194.1	0.171953	0.111183
AC027373.1	-0.59431	0.111157
FGFR4	0.088274	0.111143
ODR4	0.039228	0.111133
DEF8	-0.0417	0.111117

GNL3	0.029911	0.111101
AC007238.1	-0.33472	0.111067
COG6	0.045985	0.111058
AC010761.6	-0.06583	0.111057
HYAL2	-0.04272	0.111056
MYCT1	-0.15747	0.111055
CDYL2	-0.4852	0.111005
RAB5C	-0.03736	0.110988
PLA2G4A	-0.5526	0.110988
AC087683.1	-0.60656	0.110979
AC034102.1	0.057104	0.110974
NF1P8	0.7142	0.110966
KRT7	-0.29843	0.110901
ALDH16A1	0.042502	0.110897
LINC00863	-0.07967	0.110895
ZDHHC22	-0.10003	0.110881
DGCR5	0.108628	0.110862
GAL3ST4	0.103049	0.110833
S100A2	-0.16839	0.110819
AC004477.2	-0.03875	0.110796
NA	-0.61977	0.110791
CACNB2	-0.10522	0.11079
TTC21B	0.042333	0.110785
MAATS1	0.073255	0.110776
CDAN1	0.054497	0.110646
POGK	-0.03069	0.110644
NA	0.038975	0.110639
TSC2	-0.04341	0.110589
TP53TG3GP	0.804688	0.110573
SNORA11F	0.253999	0.110541
SLX1B	1.116425	0.110508
SPATC1	1.116425	0.110508
SNORA74A	1.116425	0.110508
RNA5SP78	1.116425	0.110508
NA	1.116425	0.110508
AL512430.1	1.116425	0.110508
AC006122.1	1.116425	0.110508
RPL23AP10	1.116425	0.110508
NCOR1P1	1.116425	0.110508
RPL35AP26	1.116425	0.110508
MORF4L2P1	1.116425	0.110508
AL358332.1	1.116425	0.110508
AC130371.1	1.116425	0.110508
AF038458.1	1.116425	0.110508
AC092807.2	1.116425	0.110508
CEP131	0.045376	0.110475
MRPS6P2	-0.8302	0.110437
RN7SL209P	-0.8302	0.110437
GPRIN2	0.471087	0.11041
TUBA3FP	0.051078	0.110409

NA	0.849172	0.110381
MIR2052HG	-0.35182	0.110279
CCDC74BP1	0.125687	0.110272
SDHAP1	-0.05967	0.110262
SC5D	0.056644	0.110258
RNA5SP195	0.529053	0.110174
SYT2	0.135635	0.110142
EFCAB5	0.187483	0.110131
PIGO	-0.04066	0.110112
AAGAB	-0.03512	0.110097
HCG15	-0.2193	0.110091
KIZ	0.060126	0.110086
TEKT5	0.356042	0.110081
ATR	-0.04324	0.110077
KXD1	0.032054	0.110072
IRAK4	-0.05943	0.11003
AP001021.2	0.246476	0.110011
ENO1-AS1	0.374375	0.110007
RARB	-0.19281	0.110005
NA	0.079342	0.110001
AC097515.1	-0.27726	0.109974
METTL18	0.060708	0.109937
LPIN3	0.154815	0.109925
NENF	-0.04009	0.109925
ZNF45	0.03772	0.109903
WIPF1	-0.04432	0.109888
PIGT	-0.03281	0.109882
AP002990.1	0.178677	0.109871
LINC00643	0.845132	0.109857
MIR153-1	0.845132	0.109857
NA	0.845132	0.109857
RF01225	0.845132	0.109857
VPS37C	0.044377	0.109855
SNORC	-0.24525	0.109829
HMGB1P41	0.584888	0.109817
AC022035.1	0.223124	0.10981
DKK1	-0.08854	0.109782
AC021127.1	-0.16712	0.10977
CPAMD8	-0.3926	0.109767
AC067852.2	-0.0541	0.109764
ENTPD4	0.03804	0.109737
AC109635.4	-0.08132	0.109725
GXYLT1	-0.04153	0.109725
ZNF664	0.027947	0.109677
PSMD3	0.033985	0.109658
NA	0.08255	0.109637
MSI2	-0.0292	0.109614
NA	0.144891	0.10961
TGFB2	-0.07645	0.109603
AL844908.1	-0.45302	0.1096

AC090159.1	0.469796	0.109595
CAVIN4	-0.08095	0.109575
SDHA	-0.03981	0.109571
BRD9P2	-0.66462	0.109567
SYNC	-0.03895	0.109558
TMBIM6	0.026485	0.109542
SRC	0.037888	0.109527
AL512380.1	-0.44691	0.109518
NA	0.823199	0.1095
PIEZO1	-0.06394	0.109442
DCAF5	-0.03557	0.109434
AL356056.1	0.321774	0.109418
E2F3P2	0.583183	0.109418
AC092803.2	-0.12488	0.109403
NA	0.409687	0.109362
ZNF20	0.271942	0.109359
PCLAF	-0.05423	0.109346
NA	-0.34085	0.109335
AC011497.1	-0.50062	0.109312
ZNF35	0.044274	0.109301
GFER	-0.04427	0.109293
NEGR1	0.053053	0.109292
NA	-0.81268	0.109291
PMF1	0.04981	0.109272
ANKK1	0.916586	0.10923
AC006441.1	0.916586	0.10923
CLDN20	-0.30377	0.109229
OXCT1	-0.04204	0.109191
NA	0.037873	0.109164
EML3	0.041894	0.109161
PIWIL3	-0.62528	0.109159
AC012368.2	-0.59804	0.10914
PROSER1	-0.03514	0.10911
MYOZ3	0.061757	0.109082
TP53TG1	-0.05631	0.10907
AC021092.1	0.042945	0.109062
ZBTB4	-0.03682	0.108999
CRYBB2	0.309193	0.108998
GRWD1	-0.03488	0.108972
HSD17B11	0.040937	0.108969
YIPF6	0.031357	0.108968
NA	0.287776	0.108968
SNORD105	1.117162	0.108967
HMSD	1.117162	0.108967
POU5F1P4	1.117162	0.108967
RPSAP41	1.117162	0.108967
AC120042.1	1.117162	0.108967
MIR4639	1.117162	0.108967
TRABD2A	0.083054	0.108942
GTPBP8	0.048789	0.108923

SLC9A8	0.051781	0.108905
SUMO2P17	-0.21409	0.108903
DPY19L2	-0.06113	0.108901
SLC26A8	1.117208	0.108871
NA	1.117208	0.108871
AC073346.1	1.117208	0.108871
AC093159.1	1.117208	0.108871
FBXO36-IT1	1.117208	0.108871
AC008040.1	1.117208	0.108871
RPL23AP67	1.117208	0.108871
CRYZL2P	1.117208	0.108871
RNU6-62P	1.117208	0.108871
AC092620.2	1.117208	0.108871
AC091544.4	1.117208	0.108871
MIR4701	1.117208	0.108871
OTUD3	-0.0412	0.108848
GPR146	0.094136	0.108762
GLDN	0.284285	0.108755
FJX1	-0.05071	0.108745
AC110015.1	-0.05087	0.108742
AL162615.1	-0.62253	0.108732
RTN3P1	0.559005	0.108677
DEPDC1B	-0.04993	0.108664
NPR3	-0.16011	0.108657
NA	0.280072	0.10862
LCN12	-0.42525	0.108591
CORO6	-0.06132	0.108586
AL049829.2	-0.50244	0.108574
AC133785.1	0.29036	0.108541
PLIN4	0.223026	0.108541
AC017083.2	-0.04702	0.108495
BX664615.1	-0.09358	0.108456
AC005730.2	0.393399	0.108448
RPL7P47	0.657987	0.108375
MIR548AR	0.657987	0.108375
KIAA0040	-0.1109	0.108313
ACTN1	-0.03447	0.108311
MT-ND2	0.028071	0.10831
NA	0.572638	0.108307
RF00019	-0.56012	0.108289
CLDND1	0.037156	0.108287
AC090386.1	0.670617	0.108269
DMTF1	0.034644	0.108259
NA	0.535991	0.10824
NA	0.157069	0.108222
RCBTB1	0.036387	0.108219
AC003989.1	0.907917	0.108209
AC007272.1	0.907917	0.108209
NA	0.907917	0.108209
RN7SL577P	0.907917	0.108209

C7orf26	-0.03629	0.108184
KHDC1	0.108231	0.108179
TMEM161B	-0.04509	0.108178
RANP1	0.268125	0.108173
B3GALNT2	0.038521	0.108147
TGFBR3L	0.133226	0.108147
AL096711.1	0.845747	0.108143
AC100771.2	0.845747	0.108143
NA	-0.08042	0.108142
RAD50	-0.03639	0.108138
AC093012.1	-0.15391	0.108094
NDRG4	-0.03812	0.108089
MAGEA10	0.04357	0.108081
AC021739.4	0.138765	0.108064
AC005091.1	0.071871	0.108056
TNIK	0.061673	0.108053
CXCL14	-0.32146	0.108037
ZFP3	-0.05446	0.108009
AL049629.1	0.210628	0.107981
NCKAP1	-0.03157	0.107975
PDIA5	0.061128	0.10793
AC010931.3	0.462948	0.107929
DST	0.033037	0.107926
AC016747.2	0.218365	0.107909
ZNF138	0.057652	0.107895
AC110769.2	0.209879	0.10789
AC027319.1	-0.08512	0.107871
MIR3177	0.480016	0.107852
CBWD2	0.046275	0.107842
KIF2A	-0.03252	0.107824
ATP5MG	0.040627	0.10782
SMPD2	0.070453	0.107817
PARP9	0.052328	0.107816
CEP135	0.051955	0.107759
NA	-0.05625	0.107755
MON2	0.038174	0.107751
NA	0.063584	0.107702
BTN2A3P	-0.1068	0.107676
SARS2	0.094972	0.107672
PCDHA12	-0.11099	0.107626
AC099786.3	-0.50094	0.107626
OXSR1	-0.03121	0.107607
AC009549.1	-0.13411	0.107595
AC098798.1	0.393288	0.107587
BNIP2	0.035813	0.107538
TRMO	-0.06303	0.107531
C16orf89	0.384236	0.107527
RRAGC	-0.04536	0.107511
JMJD4	-0.04882	0.107494
AC022165.1	0.156716	0.107476

MAP3K5	-0.06254	0.107475
AL732372.3	0.487877	0.107473
DBH	0.085281	0.107471
YBX1P6	0.717038	0.10744
LYPLA1P3	1.019367	0.107429
AC010884.1	1.019367	0.107429
AC003080.1	1.019367	0.107429
PPIAL4G	1.019367	0.107429
LINC02626	1.019367	0.107429
AC004522.2	1.019367	0.107429
C8orf37-AS1	1.019367	0.107429
NA	1.019367	0.107429
APCDD1L-DT	0.27153	0.107409
ST3GAL2	0.036529	0.10739
ZNF771	-0.03876	0.10737
ZNF503-AS2	0.079215	0.107351
PATL2	-0.36426	0.107342
RBM28	0.030738	0.107319
FAM13B	-0.0315	0.107313
GOLGA2P10	0.345042	0.107288
C16orf72	0.039411	0.107269
RNU6-476P	-0.53487	0.107264
C15orf40	0.039972	0.107224
THSD1	-0.18899	0.107207
PLD3	-0.04034	0.10719
CTPS1	-0.03871	0.10715
LAMTOR1	-0.03364	0.107135
TGDS	-0.05342	0.107107
SEPSECS	0.049633	0.107106
CRIP2	0.032305	0.107089
RF00019	-0.64948	0.107046
VDAC1P2	0.714292	0.107019
LACTB	0.064045	0.107016
ALCAM	-0.11207	0.107012
NOS1	0.200458	0.106983
AC010605.2	-0.13992	0.10698
SCRN2	-0.04064	0.106976
NA	-0.7861	0.106975
AL121957.1	-0.7861	0.106975
ZMAT1	0.075228	0.106953
AC010615.1	-0.14476	0.106938
PIGK	0.046688	0.106815
AC008124.1	0.050675	0.10681
SLF2	-0.04566	0.10679
AC100782.1	0.101496	0.106789
SEPT8	0.03421	0.106758
PER3	-0.08544	0.106706
TBX22	0.212904	0.106698
AC111170.2	0.617608	0.106679
CCL27	0.533027	0.106675

RPL21P120	0.497695	0.106647
PCDHA2	-0.11631	0.106629
AC007383.2	0.079408	0.106602
AP000766.1	-0.13565	0.106599
CACNB3	0.052053	0.10659
UBE2E2	-0.05054	0.106584
SLC16A8	0.220839	0.106549
FSTL5	0.087842	0.106548
NDFIP2	-0.03192	0.106456
SRSF9	0.028374	0.106447
FASN	-0.07813	0.106444
ZNF341-AS1	-0.61279	0.106441
FIBIN	-0.19411	0.106431
TLE2	-0.11958	0.106415
TSKU	-0.04178	0.106407
RSPH3	0.049822	0.106339
CKS2	-0.03977	0.106305
MEN1	0.040305	0.106276
NA	0.256205	0.106253
SMARCD2	-0.02862	0.106247
AC092162.2	0.43566	0.106193
ZNF670-ZNF695	-0.05924	0.106177
SLC7A1	0.036647	0.106172
EPHB2	-0.06403	0.106094
AL162311.3	-0.11223	0.106086
SLC10A3	-0.05839	0.106085
ADAM8	0.13402	0.106051
AL031708.1	0.263362	0.106045
AC078846.1	-0.06253	0.106044
RPP30	-0.04272	0.106023
VPS26A	-0.03232	0.106017
ZC3H7A	0.035252	0.106014
NRIP3	0.106983	0.106001
HIST1H4PS1	0.528915	0.105995
RNU7-82P	0.486014	0.105977
NA	0.089056	0.105975
TAB1	-0.0514	0.105946
XPA	0.055606	0.105931
SLC39A11	0.047799	0.105907
AQP4	1.019807	0.105901
OR2L13	1.019807	0.105901
KRT18P12	1.019807	0.105901
BRAFP1	1.019807	0.105901
AL136298.3	1.019807	0.105901
AC066612.1	1.019807	0.105901
AC138761.1	1.019807	0.105901
EHMT2	0.034508	0.105876
USP13	0.038645	0.105834
PTTG1	0.045591	0.105826
AC006305.1	1.019834	0.105806

HSPA8P7	1.019834	0.105806
AL157400.1	1.019834	0.105806
LINC01169	1.019834	0.105806
AC104984.3	1.019834	0.105806
OR10AC1	-0.57862	0.105782
PCSK6	0.058269	0.10578
AC005674.2	-0.15852	0.105778
NUPR1	0.339595	0.105764
AC124283.3	0.557608	0.105754
ELOVL5	0.031303	0.105749
GOLM1	-0.02971	0.105741
ECSCR	-0.46255	0.105736
DCK	-0.04535	0.105727
ZNF674	0.064163	0.105705
LRRC14	-0.04285	0.105702
MKS1	-0.05115	0.105659
FBXO31	-0.05384	0.105633
SRP54-AS1	0.188153	0.105622
AC099343.3	0.170988	0.105608
AC245595.1	-0.09064	0.105589
ICA1L	-0.06127	0.105582
PRKAB1	-0.04724	0.10558
TUBB	0.029679	0.105547
AL441883.1	-0.13524	0.10554
PRTG	-0.0394	0.105534
TMPRSS9	0.111388	0.105513
ZNF410	-0.2048	0.105509
AC011491.3	0.390953	0.105485
AC011352.3	0.46685	0.105454
PIP4P1	-0.04555	0.105453
AL096678.1	-0.14164	0.105451
CYP46A1	-0.59714	0.105434
MBLAC2	0.054837	0.105393
ZNF567	0.064636	0.105386
ELL	-0.07232	0.105379
SCML2	-0.04881	0.105318
UBB	0.029876	0.105273
SPACA6P-AS	-0.13351	0.105272
CHST5	0.519237	0.10526
RGS5	-0.04511	0.105249
BTG3	0.044988	0.105246
SIX3-AS1	-0.06706	0.105223
SCG3	0.039164	0.105221
BEAN1	-0.65605	0.105205
AKR1C2	0.30097	0.105188
ATP6V0A1	0.036288	0.105184
SP140L	-0.08672	0.10513
NA	0.208959	0.10512
GPX1P1	0.250223	0.105088
CPSF3	-0.03179	0.105074

RAB28	-0.05234	0.105069
RHOBTB2	0.043641	0.105053
AL354726.1	-0.68783	0.105049
Z97832.2	0.145373	0.105047
VAT1L	0.050607	0.105034
KANSL2	0.039702	0.105021
HSPD1	-0.02892	0.105019
AL157823.2	-0.14841	0.105012
NPRL3	-0.0461	0.104977
LINC02076	0.361337	0.104973
TMEM147	-0.0315	0.104945
AL161747.2	-0.09041	0.104926
CHAD	0.173976	0.104912
AC005776.2	-0.24409	0.104906
TBC1D2B	-0.05024	0.104889
SRP72	0.031114	0.104886
ZNRF3	-0.05378	0.104862
GALT	-0.06432	0.104857
HNRNPUL2-BSCL2	-0.61163	0.104851
CSTF1	-0.04746	0.104804
SUMO2P15	-0.49613	0.104784
RPS20P10	-0.53266	0.104734
MFAP1	-0.03791	0.104733
TOX4	-0.02884	0.10473
RRAS2	-0.05234	0.104721
MARC1	-0.04896	0.104711
ARIH1	-0.03101	0.104697
LACC1	-0.07586	0.104689
RN7SKP150	-0.3748	0.104682
AC006960.3	-0.57343	0.10466
EIF4ENIF1	-0.03757	0.104632
DENND2A	0.055089	0.104617
LAS1L	-0.03134	0.104608
RNU4-29P	0.561312	0.104579
NA	0.561312	0.104579
NISCH	-0.03969	0.104567
NA	-0.36853	0.10454
ZNF571	-0.08556	0.104485
AC022217.2	0.434347	0.104477
POLR2C	-0.02805	0.104464
SERTAD4-AS1	-0.08624	0.104449
AL133215.1	-0.12758	0.10444
MANEA-DT	-0.1403	0.104438
SNORD59A	0.416358	0.104432
TERC	0.286931	0.104431
AC009779.3	-0.1827	0.104385
EDC3	-0.03608	0.104348
EXD3	-0.07694	0.104347
AL596087.2	-0.63178	0.104328
RAP1GAP	0.065009	0.104316

ALDOB	-0.47327	0.104299
PTPRD	0.107094	0.104292
RN7SKP70	0.42164	0.104285
VPS13A	-0.0447	0.104274
NA	0.412199	0.104254
AL353746.1	0.088264	0.10425
GOLGA2P5	0.053779	0.104245
AC027682.5	-0.11094	0.104175
NA	-0.11552	0.104172
RTN4R	0.201378	0.104098
NA	-0.65833	0.104062
DTX2	-0.06241	0.104049
TAZ	-0.04738	0.104007
PHKA2	0.041119	0.104
NRBP1	-0.02737	0.10397
MIR127	-0.45234	0.10393
HMGN3-AS1	0.049258	0.103901
VCP	0.025458	0.103874
NA	0.181267	0.103857
TIMELESS	-0.03235	0.103832
LINC00612	-0.40477	0.103821
ADORA1	-0.20102	0.103806
SLC9A3-AS1	0.078681	0.103802
RPS7P10	0.201732	0.103743
DPF3	-0.16443	0.103732
BHLHE40-AS1	0.25731	0.103721
AC137630.1	-0.05394	0.103697
CD47	-0.04491	0.10369
ZDHHC23	-0.05643	0.103677
LILRA6	0.512336	0.103659
RNF19A	0.034668	0.103658
C1orf216	-0.04382	0.103641
FAM189B	-0.03837	0.103615
ST3GAL1	0.052583	0.103614
AC005899.3	-0.29235	0.103606
AP001094.1	-0.53751	0.10359
MMP24	-0.04189	0.103579
AL133367.1	0.098124	0.103566
AC108693.2	-0.14786	0.103535
ZNF528	-0.05169	0.103528
AL731569.1	0.114949	0.103479
AP002383.2	0.058765	0.103464
PHKA2-AS1	0.080595	0.103442
GALC	-0.04215	0.10344
HNRNPA1P50	-0.58288	0.103427
AC023886.2	0.541029	0.103421
ANKS1A	-0.041	0.10342
AC012065.4	0.156249	0.1034
MRPL44	-0.04423	0.103382
NAP1L5	0.047546	0.103382

SATB2	0.271614	0.103371
LIX1	0.089927	0.103296
AC004551.2	-0.14173	0.103295
VPS9D1	0.052357	0.103289
INO80E	-0.02729	0.103262
AC138951.1	-0.39874	0.103228
ZNF800	-0.04019	0.103204
PPP5D1	-0.26221	0.103173
DPP9-AS1	0.069437	0.103168
ISL1	0.034857	0.103163
LRP10	-0.04486	0.103106
SCARF1	-0.15579	0.103102
AAR2	0.033676	0.103084
HCN4	-0.31484	0.103038
ALX1	-0.21966	0.103022
IMP4	-0.03219	0.103019
NA	-0.54453	0.103009
AC138956.1	-0.05809	0.102981
GAS6-AS1	-0.0743	0.102979
SNORD99	0.330553	0.102972
UBTD1	0.059668	0.102932
AL096870.3	-0.24605	0.102909
RITA1	-0.05302	0.102895
CDK8	-0.03845	0.102889
AC027117.1	-0.65208	0.102872
NA	-0.16457	0.102861
PMPCB	0.027879	0.102807
ZHX1	0.042272	0.10278
NA	-0.04955	0.102753
BSN-DT	-0.35624	0.102746
FAM122C	0.090547	0.10274
PWP2	0.035027	0.102726
HIC2	-0.07139	0.102706
FOXP1-IT1	-0.46918	0.102704
SLC35G1	-0.04841	0.102698
RPL35AP32	0.591943	0.102693
P2RY11	-0.04368	0.102642
HMGN1P38	-0.32776	0.102641
AC108449.3	0.096751	0.102607
AC104985.1	0.116656	0.102602
AC020915.3	-0.10147	0.102572
ARL16	-0.03513	0.102567
DGCR11	0.141598	0.102562
GBP1P1	-0.2693	0.102549
ZNF512	0.031738	0.102541
PTENP1	-0.10406	0.102528
USO1	0.029514	0.10251
CBWD3	0.109713	0.102504
NA	0.156557	0.102494
FAM20C	0.226531	0.102483

TMEM127	-0.0342	0.102454
SUPT20HL2	-0.30484	0.102454
NAT8L	-0.05182	0.102431
KRTCAP2	0.071596	0.102421
AEN	0.036332	0.10241
NPY4R	0.417569	0.102389
FOLR1	-0.26046	0.102371
NA	0.053912	0.10237
RF00404	-0.26809	0.102293
FLVCR2	0.150839	0.102247
AQP10	-0.17272	0.102243
RPL23AP2	-0.36694	0.102186
B4GALT5	0.032063	0.102177
ULK1	0.043997	0.102156
NA	-0.58066	0.102148
AC009148.1	-0.18356	0.102146
ALKAL2	-0.2146	0.10214
UBR5	0.027735	0.102136
SLC25A42	-0.1034	0.102131
AC063944.2	0.394472	0.102116
NA	-0.21015	0.102078
PLCG1	-0.02874	0.102072
ZMYND19	-0.03845	0.102041
NA	-0.18936	0.102
AC117498.1	-0.055	0.101989
PRRT3	0.101233	0.101965
IL6R	-0.20377	0.101963
FAM78A	-0.06594	0.101955
TBC1D15	0.035885	0.101932
HNRNPA3P9	-0.23147	0.10191
BLOC1S3	0.068752	0.101886
RRP7A	-0.04211	0.101873
TBKBP1	-0.04044	0.101861
MIR135A1	0.524007	0.101849
METRN	0.042311	0.101848
DCUN1D2-AS	-0.32014	0.101829
TMEM38B	-0.05471	0.101767
NA	0.192285	0.101766
AL160408.2	-0.0444	0.101745
FAM83D	-0.04456	0.101733
TMEM191A	0.364224	0.101708
LRRC37A16P	0.040072	0.10168
NT5DC1	0.04484	0.101675
NTN4	-0.20907	0.101613
LMAN2L	0.053387	0.101558
DDX1	0.027975	0.101557
ABTB2	0.069821	0.101548
AC093249.4	-0.31997	0.101539
HIP1R	-0.04568	0.101513
PALM3	0.074254	0.101499

LANCL1	0.033424	0.101479
GOLGB1	-0.03452	0.10147
PCDH10	-0.37064	0.101464
AL645939.1	-0.52954	0.10146
KIF11	-0.0415	0.101444
AC010478.1	-0.03219	0.101434
ANKRD28	0.033028	0.101434
TRPC4AP	-0.03207	0.101428
NDN	-0.13853	0.101407
SHPK	-0.11499	0.101356
LRRFIP2	0.037744	0.101339
LGALS8-AS1	0.125128	0.101288
LHFPL4	-0.08556	0.101263
PCYT1A	0.032238	0.101228
AC018804.1	-0.36512	0.101225
AC117490.2	-0.12139	0.101189
ERICH3-AS1	-0.23974	0.10113
ZNF497	-0.05973	0.101127
SNHG9	0.176624	0.101119
AC016876.1	0.048922	0.101111
STXBP5-AS1	0.134097	0.101036
PTGES	-0.1093	0.101021
CUL7	-0.03958	0.101018
PCDHB13	-0.19335	0.100997
SUMO1	0.034358	0.100994
CHMP4B	0.033613	0.100992
H3F3C	0.32842	0.100982
FAP	-0.17387	0.100975
FUNDC1	0.053338	0.100968
AC106820.5	-0.07206	0.100936
LINC01414	-0.32319	0.10093
UCKL1	0.037565	0.100921
AC036176.1	0.117934	0.100911
PPIHP1	0.639388	0.100895
AC139491.5	0.575389	0.100869
FBXO38	0.029982	0.100864
FDXR	0.04496	0.10085
AC012508.1	-0.37641	0.100806
UNC13A	-0.03373	0.100799
SUPT20H	-0.03251	0.100793
AC005540.1	0.041256	0.100792
AL121594.1	-0.17371	0.100782
SLC31A1	-0.04093	0.100746
NDUFS6	-0.03833	0.100734
AC104563.1	0.415503	0.100714
RAD1	0.040557	0.100686
TEC	0.167629	0.100673
AC127496.3	0.139751	0.10067
SNORA58B	0.856491	0.100666
AC092810.1	0.856491	0.100666

EZH2P1	0.856491	0.100666
BRWD1-IT1	0.856491	0.100666
METTL27	-0.17215	0.100661
HSPA12B	0.192779	0.100658
NABP2	-0.02871	0.100647
TNFRSF14	-0.40218	0.100643
TMEM254	0.06256	0.100597
ABHD12	-0.04024	0.100587
GADD45GIP1	0.03078	0.10058
AL121672.1	-0.26168	0.10058
KRTAP5-1	0.166701	0.100576
KCNK6	-0.20308	0.100575
CKMT1B	-0.07807	0.100521
AL359532.1	-0.32943	0.100516
MRPS31P5	-0.13442	0.100512
AL356653.1	0.46383	0.100505
AC022087.1	-0.07964	0.100502
XRN1	-0.03693	0.100498
GOLGA8N	-0.33559	0.100485
CTDNEP1	-0.02999	0.100485
BTAF1	0.033412	0.100484
AL121601.1	0.309942	0.100478
AF129075.1	0.186869	0.10047
CNST	0.033185	0.10046
TMSB15B-AS1	0.20781	0.100459
NA	-0.10675	0.100451
AUTS2	0.046075	0.100435
PDS5A	-0.03015	0.100432
AC090371.2	-0.61091	0.100425
TOB1-AS1	0.084963	0.100383
COL8A2	-0.21375	0.10038
PRKCA	-0.03418	0.100355
ACAA2	-0.03659	0.10031
GTF2IP13	-0.1811	0.10028
AP000692.1	-0.36884	0.100278
RPL34P34	-0.68526	0.100257
CD99L2	0.043803	0.100209
RHOT2	0.029752	0.100185
SEPT9	-0.03591	0.100174
AP001372.2	0.093989	0.10017
IHH	-0.37535	0.100157
TLK2	-0.02794	0.100117
DMBX1	-0.48761	0.100105
PTBP2	0.041366	0.100093
CPLX1	-0.04283	0.100055
NSL1	-0.03692	0.100047
AC027607.1	-0.30949	0.100042
WDR92	-0.07741	0.100023
PRELID3A	0.068139	0.100023
SLC22A20P	-0.62047	0.100017

AC009090.1	0.506424	0.100016
PXYLP1	-0.04044	0.099995
INKA2	0.054663	0.099977
SLC15A2	-0.15623	0.099961
AC127502.2	-0.11699	0.099933
WTAPP1	-0.19608	0.099906
SNHG15	-0.03513	0.099879
ASB8	-0.04013	0.099857
GPR3	0.117498	0.099847
SGPL1	-0.03343	0.099846
DCP2	-0.0311	0.099815
AC011477.1	-0.08197	0.099806
FGD5-AS1	-0.02942	0.099794
IFT80	0.04771	0.099794
NA	-0.19706	0.099793
G3BP2	0.028641	0.099792
DOCK11	0.050528	0.099789
DPY19L1P2	-0.16499	0.099777
AC005546.1	0.373021	0.099772
NA	0.605429	0.099644
SLIT1	-0.48186	0.099604
TAS2R10	-0.22574	0.0996
BIRC6	-0.02646	0.099595
RNU6-483P	0.788556	0.099588
AC008572.1	0.788556	0.099588
AC004134.1	0.788556	0.099588
GAS6-DT	0.231981	0.099581
ACP4	0.211463	0.099511
RPRD2	-0.03495	0.099509
NA	-0.21821	0.099432
MRPL28	0.031522	0.099416
SLC25A35	0.039538	0.099368
AL354733.3	-0.02885	0.099321
NA	-0.03327	0.09931
PAXIP1-AS2	0.054228	0.099278
IRF5	-0.22726	0.099271
TBX19	0.106478	0.099259
MPHOSPH8	-0.03344	0.099244
FAM214B	-0.04934	0.099231
AADACL2-AS1	-0.24623	0.099226
NA	0.775669	0.099213
AC011477.4	0.775669	0.099213
DHODH	-0.05081	0.099199
ANAPC10	-0.05565	0.09919
PPIP5K2	0.03124	0.099173
TRIM41	-0.03328	0.099141
AL451124.1	-0.66078	0.099119
NR2F2	0.449873	0.099112
AL353804.2	-0.38946	0.099068
ELOA-AS1	0.04928	0.099062

FAM155A	-0.04249	0.099038
ERLEC1	-0.03301	0.099035
NR1I3	0.067016	0.099021
NA	-0.35713	0.09902
AC002558.2	0.070295	0.099016
CHKA	-0.04256	0.099011
AL133477.1	-0.42466	0.098972
MUC5B	0.734798	0.09896
AC108718.1	0.734798	0.09896
NA	0.070936	0.098945
AC004232.1	0.295053	0.098926
GNAT2	-0.24015	0.098922
MSS51	-0.14118	0.098919
TEN1-CDK3	-0.08497	0.098914
PTK2	-0.02727	0.098906
TULP1	0.680896	0.098875
AC063944.1	0.680896	0.098875
COX4I2	-0.211	0.098835
ARHGAP44	-0.20082	0.098834
KCNQ2	0.039152	0.098819
AC024257.3	-0.25114	0.098809
INA	0.039079	0.098784
AC053503.1	0.538845	0.098784
TP53TG5	0.129503	0.098712
ZNF536	-0.04999	0.098684
HNRNPA3P5	-0.19819	0.098656
NAA35	-0.03661	0.098635
RTEL1-TNFRSF6B	0.101354	0.098633
ANLN	0.036841	0.098572
C19orf67	-0.53487	0.098544
DNAJC19P5	-0.53487	0.098544
ZNF337-AS1	-0.04293	0.098534
MIR3942	0.780115	0.098527
ADGRL3-AS1	-0.66178	0.098527
PGBD2	-0.06869	0.098518
NA	-0.04403	0.098506
ATP6AP1	-0.03386	0.098504
ROR1	0.043824	0.098475
ZBTB10	-0.03327	0.098445
AC022415.1	0.282236	0.098436
TEX9	0.07837	0.098408
ABCG4	-0.31571	0.0984
AL031186.1	0.108833	0.098397
BEND3P1	0.065287	0.098375
R3HCC1	0.045036	0.098365
RPRD1A	0.029391	0.098349
KIAA2013	-0.03439	0.098343
NA	-0.315	0.09828
RXYLT1	0.048802	0.098278
TMEM120A	0.04223	0.098275

WAS	-0.21095	0.098274
DGKZP1	0.394457	0.098252
KRT10	0.049023	0.098242
Z69890.1	-0.32727	0.09821
MORN3	-0.10594	0.098186
EVA1C	-0.25671	0.098184
RAB7A	-0.02461	0.098176
RFK	-0.05061	0.098166
PKN2	0.036213	0.098163
POTEH	-0.27406	0.098157
SNX13	-0.03477	0.098147
PAG1	-0.0798	0.098139
ZC3H18	-0.03123	0.098101
LRRC10B	0.261991	0.098094
NIPAL2	-0.07953	0.098088
TCF19	-0.0573	0.098078
PRKCI	-0.03175	0.098075
KRT80	-0.12357	0.098031
PRRT3-AS1	0.17208	0.097981
NRXN3	-0.14917	0.097926
TRIM33	0.031331	0.09792
NA	0.065306	0.097914
KCTD9P4	0.949718	0.097902
AL591848.1	0.949718	0.097902
KRT18P62	0.949718	0.097902
AL355601.1	0.949718	0.097902
DLX2-DT	0.949718	0.097902
AC007389.4	0.949718	0.097902
LINC02618	0.949718	0.097902
NA	0.949718	0.097902
AC010768.4	0.949718	0.097902
RPS3AP4	0.949718	0.097902
MIR3619	0.949718	0.097902
AC008750.8	0.949718	0.097902
NA	0.949718	0.097902
AC023983.2	0.949718	0.097902
PLCB4	0.034399	0.0979
AC138866.1	-0.1212	0.097863
AC011731.1	0.155787	0.097861
CHRND	-0.36706	0.09784
ZP3	-0.13721	0.097796
UBE2Q2P1	0.148524	0.097795
ALPK3	-0.23112	0.097792
C9orf106	0.71585	0.097756
RPS15AP24	0.71585	0.097756
ARHGAP31-AS1	-0.25527	0.097739
LINC01238	0.274718	0.097721
HCG18	0.029794	0.097717
CP	0.066928	0.097693
AC005703.3	-0.66355	0.097673

AC116158.1	0.14902	0.097668
HERC4	-0.03787	0.097663
AC073320.1	-0.08574	0.097645
ZNF660	-0.04923	0.097622
POLR2D	-0.03448	0.097618
SPC25	0.054351	0.097603
TMEM136	-0.05577	0.0976
AP001330.5	-0.35494	0.09755
RYR1	0.523736	0.09749
MRPL18	0.036093	0.097469
YWHAEP5	0.662733	0.097459
NA	-0.11411	0.097438
FNBP1L	0.032251	0.097434
AP000253.1	-0.29869	0.097431
FGF7P6	0.125247	0.097412
LINC00535	0.236861	0.097399
BTF3P6	0.357217	0.097376
AC135782.3	0.146607	0.097372
AL354760.1	0.626894	0.09737
NA	-0.04612	0.097316
CWF19L1	0.0408	0.097307
NA	0.082634	0.097292
AC093311.1	0.509099	0.097285
MT1F	-0.11326	0.09728
NF1P4	-0.22589	0.097277
AC096677.1	0.217049	0.097276
ALDH9A1	-0.03483	0.097273
MED6	-0.04297	0.097267
BUB1B	0.036559	0.09726
STT3A	0.042031	0.097259
TIPRL	0.032065	0.097259
ERV3-1	-0.04061	0.097228
MGAT4B	-0.03517	0.097226
PNPLA7	-0.10701	0.09722
UBE2Q1	-0.025	0.097211
PRPF40B	-0.03994	0.097197
CCS	-0.06122	0.097184
VPS33B	0.045175	0.097171
ZNF33A	0.035045	0.097158
CENPO	-0.02904	0.097152
RFC3	0.035905	0.097146
ZNF564	0.391597	0.097106
ELAVL2	0.047741	0.097075
AC103726.2	0.452174	0.097003
HERC3	-0.03288	0.097002
TNNI3	-0.24896	0.096996
AC091021.1	0.026136	0.096982
CLPP	0.037651	0.09697
TNPO1	0.037109	0.096948
ZNF821	0.056329	0.096916

NA	-0.33374	0.096908
TRRAP	-0.03942	0.096895
FNBP1	0.035846	0.096881
GPN3	-0.0377	0.096861
EGFL8	-0.11138	0.096856
AC018653.3	-0.15589	0.096856
SDK2	-0.12886	0.096806
AC127070.1	0.366193	0.096792
FUBP1	0.027491	0.096783
NA	-0.14987	0.096738
TMEM184A	0.094085	0.096731
AC007938.3	-0.0688	0.096727
TOMM22	0.031657	0.096713
EIF5B	-0.03396	0.096696
FAM8A1	-0.02914	0.096675
ARF5	0.032634	0.096629
CTSS	0.053544	0.0966
AC022137.2	0.345683	0.096564
ACP2	0.050616	0.096551
PRKCSH	-0.03315	0.096489
PRKAR1B	0.028456	0.096438
AL358472.1	0.949881	0.09643
AC090617.1	0.949881	0.09643
AC103769.1	0.949881	0.09643
NA	0.949881	0.09643
AC007496.1	0.949881	0.09643
NTRK3-AS1	0.949881	0.09643
NA	0.949881	0.09643
CCDC113	0.076978	0.096345
USP43	0.949891	0.096338
NINJ2	0.949891	0.096338
NA	0.949891	0.096338
C2orf73	0.949891	0.096338
RNU2-68P	0.949891	0.096338
PARP1P1	0.949891	0.096338
CHTF8P1	0.949891	0.096338
RASA2-IT1	0.949891	0.096338
ERHP1	0.949891	0.096338
AC112191.2	0.949891	0.096338
AC010754.1	0.949891	0.096338
MTCO2P22	0.949891	0.096338
SLC5A11	0.463065	0.096332
ZNF451	-0.03185	0.096318
GLB1L	-0.07665	0.096313
RALGDS	0.037259	0.096229
MICALL1	-0.05712	0.096216
ST3GAL5-AS1	-0.57378	0.096215
DHX35	0.041061	0.096214
ACOT7	0.031094	0.096211
ZBTB1	-0.03573	0.096205

STX8	0.058855	0.096182
IDS	-0.02697	0.096177
NA	0.044934	0.096154
PERM1	0.717779	0.096104
AL158201.1	0.717779	0.096104
VPS13C	-0.03271	0.096084
LY6G5B	-0.07327	0.096083
AC004982.2	0.116482	0.096072
USP46-AS1	-0.13686	0.096041
AC055811.1	-0.09068	0.096021
KIAA0586	-0.04178	0.096018
BAX	0.027956	0.095985
ZNF589	-0.03896	0.09598
CENPU	0.038414	0.095966
AL669831.5	-0.15604	0.095945
AC016588.1	-0.07904	0.095937
KHK	0.045262	0.095933
NA	0.051571	0.095917
PSMB2	0.030632	0.095855
ERBB2	0.043651	0.095834
AL132640.1	0.302532	0.095834
C1orf35	0.035011	0.095826
AC018809.2	-0.10527	0.09582
PI4K2A	0.040116	0.095817
SCIMP	-0.11752	0.095796
ULK4	0.066565	0.095796
C1orf56	-0.04173	0.095788
TRIM25	-0.03658	0.095787
PLBD2	0.063318	0.095763
MGAT4EP	-0.6641	0.095755
LINC02150	-0.6641	0.095755
PSIP1	0.027891	0.095743
ARNTL2	0.051902	0.095733
ELF3-AS1	-0.22246	0.095733
ZSWIM2	-0.50476	0.095697
NA	-0.10117	0.095679
WDR48	0.030941	0.095673
CRYBG1	-0.13718	0.09565
TAF2	0.033605	0.095631
PI4KB	0.03342	0.095622
LINC02269	0.106618	0.095604
AP000251.1	-0.15828	0.095603
KAT7	0.029009	0.095581
AC007599.1	-0.59952	0.09556
BCR	0.040599	0.095509
AL033384.2	0.325882	0.095499
NCAN	0.072386	0.095441
ZC3H14	0.029114	0.095424
SEC14L1	-0.0317	0.095383
LMLN	-0.06248	0.095359

MMP16	-0.03917	0.095346
RADIL	0.075262	0.095344
PAXBP1-AS1	-0.05193	0.095336
CPEB3	-0.08622	0.095334
TEX2	0.033461	0.095306
ELOF1	-0.02863	0.095283
AC011632.1	0.374475	0.095273
FOXK1	-0.03546	0.095262
TMEM201	0.051341	0.095254
GPATCH11	-0.03774	0.095248
AC009336.1	0.149322	0.09524
PMS2	-0.03396	0.095168
NIP7	0.031671	0.095168
FCMR	-0.11049	0.095147
NIPSNAP3A	0.047588	0.095098
ANKRD18EP	-0.07681	0.095094
GLT8D1	0.032316	0.095076
DDX31	0.033478	0.095072
KNSTRN	-0.03262	0.095051
CCDC92B	-0.10384	0.095019
AGGF1	-0.03345	0.094993
ITGA10	-0.18305	0.09498
NELL1	0.174948	0.094975
AFG3L1P	0.040052	0.094975
DVL1	-0.03872	0.094968
CMTM1	-0.09368	0.094962
CDH26	0.436573	0.094951
CDC34	0.029106	0.094947
TM9SF1	0.063556	0.094929
RF00019	0.669578	0.094929
LINC01440	0.669578	0.094929
ZNF780B	-0.03232	0.094886
SNX17	-0.02548	0.094869
JMY	-0.04112	0.094861
CEND1	0.046646	0.094847
MIR589	0.377748	0.094816
ZNF512B	0.042998	0.094805
NA	-0.5455	0.094801
AC026304.1	0.212219	0.094785
BLOC1S5	0.05034	0.094784
RMDN1	0.035301	0.094761
GDPD5	-0.14408	0.094704
TCEAL8	-0.03549	0.094695
NA	0.098065	0.094692
PSMC3	0.028708	0.094665
TM4SF4	0.25386	0.094645
AC024405.2	-0.17024	0.094606
ARMC1	-0.04195	0.094601
AL117339.4	-0.22798	0.094527
IQGAP1	-0.03568	0.094525

MYO5C	-0.10432	0.094509
GPR162	-0.05356	0.094497
LINC01311	0.108566	0.094453
DIS3	-0.0281	0.094432
AL133351.1	0.202288	0.094385
PCDH9	0.03718	0.094378
ST20	-0.09686	0.094345
LARS	-0.02872	0.09434
AC011503.2	-0.13805	0.094335
ENY2	-0.03148	0.094331
IFIT5	-0.04662	0.094242
RNU6-1330P	0.172841	0.094231
SLC36A1	-0.04847	0.094186
EAPP	0.033011	0.094153
NA	-0.25924	0.094114
AL645568.2	-0.65452	0.094095
SLC6A13	0.596811	0.094049
OR11H13P	0.596811	0.094049
SBNO2	0.042905	0.093953
TMEM248	-0.02764	0.093922
PLA2G4C-AS1	-0.36161	0.093921
CYTH4	0.480515	0.09391
AC006027.1	0.087609	0.093908
CICP3	0.307213	0.093905
RAB6A	-0.02618	0.09383
TMEM43	0.029065	0.093801
ZADH2	0.029434	0.0938
AC087623.3	-0.18768	0.093764
NDUFA1	0.036761	0.093723
AC104564.4	-0.44917	0.093723
SMARCAL1	-0.04029	0.093721
AHCY	-0.02716	0.093717
IKZF4	-0.04386	0.093701
AL391839.2	-0.08507	0.093699
TMEM183B	0.187657	0.093678
RNF185	0.031683	0.093677
ANO4	-0.17595	0.093668
RPAP3	-0.03372	0.093664
NA	0.03057	0.093658
PSMA6	0.086342	0.093645
YIPF4	0.027142	0.093644
TRPM7	0.028277	0.093637
TPX2	-0.02775	0.093632
ITIH4	0.654394	0.093626
CCDC152	-0.05743	0.09361
AC084757.2	-0.53549	0.093587
RBM18	0.036533	0.093579
PA2G4	0.02588	0.093524
TMEM198B	0.035958	0.093496
CCDC151	-0.04644	0.093484

NYAP1	0.060545	0.09346
VPS37A	0.033326	0.093446
CCT8L1P	0.999871	0.093435
GABRA1	0.999871	0.093435
STAP1	0.999871	0.093435
CHRDL2	0.999871	0.093435
TGFBR3	0.999871	0.093435
TRPC5	0.999871	0.093435
WNT8B	0.999871	0.093435
ATP12A	0.999871	0.093435
FGF20	0.999871	0.093435
TRPM3	0.999871	0.093435
NME8	0.999871	0.093435
LHX5	0.999871	0.093435
TBX5	0.999871	0.093435
APOH	0.999871	0.093435
SMIM24	0.999871	0.093435
PGC	0.999871	0.093435
TPTEP1	0.999871	0.093435
NKAIN4	0.999871	0.093435
TLR8	0.999871	0.093435
CPQ	0.999871	0.093435
CGB3	0.999871	0.093435
RSPH6A	0.999871	0.093435
CD33	0.999871	0.093435
THEG	0.999871	0.093435
HAMP	0.999871	0.093435
MOGAT3	0.999871	0.093435
ANXA10	0.999871	0.093435
HTATIP2	0.999871	0.093435
ELMOD1	0.999871	0.093435
KCNA1	0.999871	0.093435
RERGL	0.999871	0.093435
GLP1R	0.999871	0.093435
GCG	0.999871	0.093435
KISS1R	0.999871	0.093435
ADRA1A	0.999871	0.093435
CSTA	0.999871	0.093435
KIAA0087	0.999871	0.093435
C4BPB	0.999871	0.093435
NA	0.999871	0.093435
OPN5	0.999871	0.093435
AMELX	0.999871	0.093435
NKX2-4	0.999871	0.093435
CSRP3	0.999871	0.093435
NA	0.999871	0.093435
ACE2	0.999871	0.093435
KLHDC7B	0.999871	0.093435
HRC	0.999871	0.093435
OR11H1	0.999871	0.093435

IDO1	0.999871	0.093435
ENAM	0.999871	0.093435
MYBPH	0.999871	0.093435
EPSTI1	0.999871	0.093435
KL	0.999871	0.093435
REG4	0.999871	0.093435
SPX	0.999871	0.093435
CGA	0.999871	0.093435
IL36G	0.999871	0.093435
KIF12	0.999871	0.093435
POU2F3	0.999871	0.093435
CLCA2	0.999871	0.093435
ABCG5	0.999871	0.093435
CHRNA1	0.999871	0.093435
MMRN1	0.999871	0.093435
CELF6	0.999871	0.093435
MEP1B	0.999871	0.093435
CIB3	0.999871	0.093435
GPR32	0.999871	0.093435
CFAP74	0.999871	0.093435
FCN3	0.999871	0.093435
TMEM61	0.999871	0.093435
HAPLN1	0.999871	0.093435
FGD2	0.999871	0.093435
CYP39A1	0.999871	0.093435
ZAN	0.999871	0.093435
STRA8	0.999871	0.093435
DPYS	0.999871	0.093435
PLPPR1	0.999871	0.093435
OR5C1	0.999871	0.093435
A1CF	0.999871	0.093435
SLC22A9	0.999871	0.093435
FXYD4	0.999871	0.093435
LGI4	0.999871	0.093435
NAA11	0.999871	0.093435
ADGRG4	0.999871	0.093435
CIB4	0.999871	0.093435
PAGE5	0.999871	0.093435
PLA2G4D	0.999871	0.093435
M1AP	0.999871	0.093435
UROC1	0.999871	0.093435
PGLYRP2	0.999871	0.093435
USP41	0.999871	0.093435
LEXM	0.999871	0.093435
FCAMR	0.999871	0.093435
XIRP2	0.999871	0.093435
TGFA	0.999871	0.093435
CRYGC	0.999871	0.093435
AIM2	0.999871	0.093435
CAMP	0.999871	0.093435

FAM170A	0.999871	0.093435
TMEM200A	0.999871	0.093435
FAM183BP	0.999871	0.093435
SSMEM1	0.999871	0.093435
CFAP47	0.999871	0.093435
NCF1C	0.999871	0.093435
UCMA	0.999871	0.093435
FRMD7	0.999871	0.093435
RNASE7	0.999871	0.093435
BTNL9	0.999871	0.093435
MYRFL	0.999871	0.093435
SLC38A8	0.999871	0.093435
SCNN1G	0.999871	0.093435
AP004243.1	0.999871	0.093435
GOLGA6C	0.999871	0.093435
CA4	0.999871	0.093435
NOS2P2	0.999871	0.093435
PSCA	0.999871	0.093435
OR2W6P	0.999871	0.093435
CX3CR1	0.999871	0.093435
IL13	0.999871	0.093435
TAS2R1	0.999871	0.093435
FRMPD4	0.999871	0.093435
FABP4	0.999871	0.093435
FRMPD2	0.999871	0.093435
RBMXL2	0.999871	0.093435
KRT9	0.999871	0.093435
SPACA5	0.999871	0.093435
PTGER4	0.999871	0.093435
GPR82	0.999871	0.093435
SNTG2	0.999871	0.093435
RHOD	0.999871	0.093435
CD7	0.999871	0.093435
TOPAZ1	0.999871	0.093435
GRIK1-AS1	0.999871	0.093435
VN2R1P	0.999871	0.093435
PROP1	0.999871	0.093435
LONRF3	0.999871	0.093435
FO393415.1	0.999871	0.093435
SAMD12	0.999871	0.093435
SPATA31E1	0.999871	0.093435
AC092656.1	0.999871	0.093435
PIK3CD-AS1	0.999871	0.093435
GOLGA8J	0.999871	0.093435
TMEM150B	0.999871	0.093435
ZSCAN4	0.999871	0.093435
ENTR1P2	0.999871	0.093435
CCL13	0.999871	0.093435
OR2B8P	0.999871	0.093435
Z97985.1	0.999871	0.093435

EPGN	0.999871	0.093435
C1orf116	0.999871	0.093435
NA	0.999871	0.093435
ODF3L1	0.999871	0.093435
TRIM61	0.999871	0.093435
GOLGA8G	0.999871	0.093435
NA	0.999871	0.093435
BPIFC	0.999871	0.093435
NA	0.999871	0.093435
C11orf87	0.999871	0.093435
NKAIN3	0.999871	0.093435
CYP4F2	0.999871	0.093435
TAS2R42	0.999871	0.093435
CD300E	0.999871	0.093435
DEFB132	0.999871	0.093435
AC019294.1	0.999871	0.093435
HEPACAM2	0.999871	0.093435
PDCD1	0.999871	0.093435
GPR21	0.999871	0.093435
ASTL	0.999871	0.093435
NA	0.999871	0.093435
LINC00523	0.999871	0.093435
NA	0.999871	0.093435
UGT2B15	0.999871	0.093435
NA	0.999871	0.093435
NA	0.999871	0.093435
ADGRA1	0.999871	0.093435
ELANE	0.999871	0.093435
MPEG1	0.999871	0.093435
AC007952.1	0.999871	0.093435
TMPRSS11F	0.999871	0.093435
NA	0.999871	0.093435
ZNF534	0.999871	0.093435
FAM3D	0.999871	0.093435
F5	0.999871	0.093435
EPS8L3	0.999871	0.093435
MIR26A1	0.999871	0.093435
RF00019	0.999871	0.093435
RF00019	0.999871	0.093435
RNA5SP477	0.999871	0.093435
RF00019	0.999871	0.093435
RNU6-1301P	0.999871	0.093435
RNU6-859P	0.999871	0.093435
RN7SKP95	0.999871	0.093435
RF00019	0.999871	0.093435
RF00019	0.999871	0.093435
RNA5SP491	0.999871	0.093435
RNU6-495P	0.999871	0.093435
RF00019	0.999871	0.093435
RF00019	0.999871	0.093435

RNY1P16	0.999871	0.093435
RF00019	0.999871	0.093435
RNA5SP197	0.999871	0.093435
SNORD114-10	0.999871	0.093435
RNA5SP393	0.999871	0.093435
RNU6-527P	0.999871	0.093435
RNU1-49P	0.999871	0.093435
RF00019	0.999871	0.093435
RNU6-618P	0.999871	0.093435
RF00019	0.999871	0.093435
RNY4P14	0.999871	0.093435
RNU6-640P	0.999871	0.093435
RF00019	0.999871	0.093435
RNU6-226P	0.999871	0.093435
RN7SKP160	0.999871	0.093435
RNU6-379P	0.999871	0.093435
RNU6-595P	0.999871	0.093435
RF00019	0.999871	0.093435
RNU6-681P	0.999871	0.093435
RF00019	0.999871	0.093435
RN7SKP118	0.999871	0.093435
RF00019	0.999871	0.093435
RNA5SP513	0.999871	0.093435
RNA5SP194	0.999871	0.093435
RNU1-93P	0.999871	0.093435
RNU6-621P	0.999871	0.093435
RNY1P2	0.999871	0.093435
RNU6-384P	0.999871	0.093435
RNA5SP488	0.999871	0.093435
VTRNA1-2	0.999871	0.093435
RNU6-742P	0.999871	0.093435
RF00019	0.999871	0.093435
RNY3P12	0.999871	0.093435
RF00019	0.999871	0.093435
RF00091	0.999871	0.093435
RN7SKP292	0.999871	0.093435
SNORD82	0.999871	0.093435
RF00019	0.999871	0.093435
RF00019	0.999871	0.093435
RNA5SP151	0.999871	0.093435
NA	0.999871	0.093435
NA	0.999871	0.093435
AC015969.1	0.999871	0.093435
NA	0.999871	0.093435
NA	0.999871	0.093435
NA	0.999871	0.093435
NA	0.999871	0.093435
NA	0.999871	0.093435
CAPN8	0.999871	0.093435
SPATA42	0.999871	0.093435

C1orf141	0.999871	0.093435
COL5A1-AS1	0.999871	0.093435
CXorf65	0.999871	0.093435
PAGE3	0.999871	0.093435
OR2H2	0.999871	0.093435
FAM153CP	0.999871	0.093435
LINC00654	0.999871	0.093435
AL356414.1	0.999871	0.093435
SLCO6A1	0.999871	0.093435
AC083864.1	0.999871	0.093435
NA	0.999871	0.093435
KRTAP5-6	0.999871	0.093435
AC108134.1	0.999871	0.093435
ONECUT3	0.999871	0.093435
AC091951.1	0.999871	0.093435
MAPK6P1	0.999871	0.093435
RNU6-946P	0.999871	0.093435
SNORD116-14	0.999871	0.093435
RNU6-1062P	0.999871	0.093435
RF00019	0.999871	0.093435
RNU6-147P	0.999871	0.093435
RNU6-545P	0.999871	0.093435
RNU6-767P	0.999871	0.093435
RNU5A-6P	0.999871	0.093435
RNU6-5P	0.999871	0.093435
RNU6-662P	0.999871	0.093435
RNU6-610P	0.999871	0.093435
SNORA36C	0.999871	0.093435
RF00019	0.999871	0.093435
RF00019	0.999871	0.093435
RNA5SP122	0.999871	0.093435
SNORD116-29	0.999871	0.093435
SNORA70C	0.999871	0.093435
RF00019	0.999871	0.093435
RF00019	0.999871	0.093435
RF00096	0.999871	0.093435
RNU6-1311P	0.999871	0.093435
RF00019	0.999871	0.093435
NA	0.999871	0.093435
RF00019	0.999871	0.093435
MIR649	0.999871	0.093435
MIR7-3	0.999871	0.093435
AP000553.1	0.999871	0.093435
MIR642A	0.999871	0.093435
AL359091.1	0.999871	0.093435
MIR181B1	0.999871	0.093435
MIR3065	0.999871	0.093435
TRAJ19	0.999871	0.093435
IGHD	0.999871	0.093435
IGHV3-48	0.999871	0.093435

MIR550A3	0.999871	0.093435
RF00096	0.999871	0.093435
SNORD89	0.999871	0.093435
RNA5SP339	0.999871	0.093435
RF00019	0.999871	0.093435
RNU6-739P	0.999871	0.093435
RF00072	0.999871	0.093435
RNU6-530P	0.999871	0.093435
RNA5SP465	0.999871	0.093435
NA	0.999871	0.093435
Z97192.1	0.999871	0.093435
RPL23AP80	0.999871	0.093435
LPAL2	0.999871	0.093435
AC012362.1	0.999871	0.093435
RF00017	0.999871	0.093435
EEF1A1P16	0.999871	0.093435
YWHAZP5	0.999871	0.093435
RPL18P11	0.999871	0.093435
AKR1B1P2	0.999871	0.093435
AC093816.1	0.999871	0.093435
TPT1P13	0.999871	0.093435
AL138847.1	0.999871	0.093435
AC093270.1	0.999871	0.093435
AC098590.1	0.999871	0.093435
KRT18P17	0.999871	0.093435
BX842568.1	0.999871	0.093435
ACTN4P2	0.999871	0.093435
AL390728.1	0.999871	0.093435
NA	0.999871	0.093435
NA	0.999871	0.093435
EIF4EP5	0.999871	0.093435
FAM149B1P1	0.999871	0.093435
NOTO	0.999871	0.093435
RPSAP6	0.999871	0.093435
AC090921.1	0.999871	0.093435
USP8P1	0.999871	0.093435
EEF1A1P29	0.999871	0.093435
IGLV8OR8-1	0.999871	0.093435
LINC01020	0.999871	0.093435
NA	0.999871	0.093435
MRPL3P1	0.999871	0.093435
RPL27AP	0.999871	0.093435
MIR933	0.999871	0.093435
MIR450B	0.999871	0.093435
NA	0.999871	0.093435
AL139039.1	0.999871	0.093435
RPS3AP24	0.999871	0.093435
CCT7P1	0.999871	0.093435
ZNF192P2	0.999871	0.093435
AL078601.1	0.999871	0.093435

SUMO2P13	0.999871	0.093435
ACTG1P18	0.999871	0.093435
AL162578.1	0.999871	0.093435
HSPD1P16	0.999871	0.093435
NUFIP1P1	0.999871	0.093435
RPS3AP2	0.999871	0.093435
NA	0.999871	0.093435
AL359694.1	0.999871	0.093435
GCNT1P4	0.999871	0.093435
RPS24P12	0.999871	0.093435
Z86062.1	0.999871	0.093435
MIR1296	0.999871	0.093435
SNORD111	0.999871	0.093435
NA	0.999871	0.093435
MIR1253	0.999871	0.093435
NA	0.999871	0.093435
NA	0.999871	0.093435
SNORD3J	0.999871	0.093435
MIR1827	0.999871	0.093435
NA	0.999871	0.093435
NA	0.999871	0.093435
RF00604	0.999871	0.093435
MIR1267	0.999871	0.093435
NA	0.999871	0.093435
NA	0.999871	0.093435
MIR1256	0.999871	0.093435
RN7SKP110	0.999871	0.093435
RF00045	0.999871	0.093435
RN7SKP26	0.999871	0.093435
RNA5SP178	0.999871	0.093435
RN7SKP139	0.999871	0.093435
RF00019	0.999871	0.093435
RN7SKP125	0.999871	0.093435
RF00019	0.999871	0.093435
RNU2-62P	0.999871	0.093435
RNA5SP120	0.999871	0.093435
RNU2-25P	0.999871	0.093435
RNU5A-5P	0.999871	0.093435
RN7SKP137	0.999871	0.093435
RF00019	0.999871	0.093435
MIR1538	0.999871	0.093435
RNA5SP450	0.999871	0.093435
RF00019	0.999871	0.093435
RF00019	0.999871	0.093435
RN7SKP30	0.999871	0.093435
AL589642.1	0.999871	0.093435
MAPK6P2	0.999871	0.093435
AC083862.1	0.999871	0.093435
AC114402.1	0.999871	0.093435
EEF1A1P8	0.999871	0.093435

AL592045.1	0.999871	0.093435
RBMX2P3	0.999871	0.093435
KRT8P51	0.999871	0.093435
EEF1A1P24	0.999871	0.093435
LINC01208	0.999871	0.093435
AL109659.1	0.999871	0.093435
LINC00114	0.999871	0.093435
BOLA3P4	0.999871	0.093435
VN1R40P	0.999871	0.093435
HLA-DPB1	0.999871	0.093435
SAP18P2	0.999871	0.093435
LINC02561	0.999871	0.093435
AC092106.1	0.999871	0.093435
AMMECR1-IT1	0.999871	0.093435
MRRFP1	0.999871	0.093435
RPS29P31	0.999871	0.093435
IFIT6P	0.999871	0.093435
AC019181.1	0.999871	0.093435
AC011239.1	0.999871	0.093435
LINC01280	0.999871	0.093435
AC010886.1	0.999871	0.093435
IFNA22P	0.999871	0.093435
MKRN5P	0.999871	0.093435
NF1P6	0.999871	0.093435
PIGUP1	0.999871	0.093435
RSL24D1P6	0.999871	0.093435
AL109933.1	0.999871	0.093435
SAMM50P1	0.999871	0.093435
RPS17P13	0.999871	0.093435
AC006026.1	0.999871	0.093435
AL022326.1	0.999871	0.093435
NA	0.999871	0.093435
AC107079.1	0.999871	0.093435
AL158207.1	0.999871	0.093435
AL583808.1	0.999871	0.093435
AL365194.1	0.999871	0.093435
AC018643.1	0.999871	0.093435
OSTM1-AS1	0.999871	0.093435
RPL5P25	0.999871	0.093435
AC005105.1	0.999871	0.093435
AL583804.1	0.999871	0.093435
AC022080.2	0.999871	0.093435
AC078942.1	0.999871	0.093435
AC015977.2	0.999871	0.093435
AC010878.1	0.999871	0.093435
C9orf135-DT	0.999871	0.093435
PGAM1P2	0.999871	0.093435
FCF1P6	0.999871	0.093435
PACRG-AS3	0.999871	0.093435
AL008633.1	0.999871	0.093435

AC114737.1	0.999871	0.093435
NPM1P18	0.999871	0.093435
AC087501.1	0.999871	0.093435
AC009299.1	0.999871	0.093435
AL035090.1	0.999871	0.093435
NIPA2P1	0.999871	0.093435
NA	0.999871	0.093435
LINC00368	0.999871	0.093435
AL136231.1	0.999871	0.093435
NA	0.999871	0.093435
AL441989.1	0.999871	0.093435
AL807761.3	0.999871	0.093435
PCMTD1P1	0.999871	0.093435
AC073283.2	0.999871	0.093435
RPS27AP3	0.999871	0.093435
SUPT4H1P1	0.999871	0.093435
ARHGAP26-AS1	0.999871	0.093435
AL359182.1	0.999871	0.093435
LINC00375	0.999871	0.093435
AC093642.1	0.999871	0.093435
AL358613.1	0.999871	0.093435
USF1P1	0.999871	0.093435
KIRREL1-IT1	0.999871	0.093435
AC087499.2	0.999871	0.093435
AC074375.1	0.999871	0.093435
RPL7P22	0.999871	0.093435
NOP56P2	0.999871	0.093435
AL391647.1	0.999871	0.093435
C13orf42	0.999871	0.093435
AL117382.1	0.999871	0.093435
AL390036.1	0.999871	0.093435
AL035416.1	0.999871	0.093435
AC093019.1	0.999871	0.093435
NA	0.999871	0.093435
AL035410.1	0.999871	0.093435
AC025946.1	0.999871	0.093435
AL445433.1	0.999871	0.093435
NA	0.999871	0.093435
LINC00866	0.999871	0.093435
LINC01806	0.999871	0.093435
AC114402.2	0.999871	0.093435
PCGEM1	0.999871	0.093435
AL592301.1	0.999871	0.093435
AL160163.1	0.999871	0.093435
AC002542.1	0.999871	0.093435
RPS3AP53	0.999871	0.093435
AL121877.1	0.999871	0.093435
RPS3AP37	0.999871	0.093435
FRMPD3-AS1	0.999871	0.093435
ISCA1P6	0.999871	0.093435

AL359915.1	0.999871	0.093435
AL590128.1	0.999871	0.093435
MTND5P1	0.999871	0.093435
NGF-AS1	0.999871	0.093435
AC023469.2	0.999871	0.093435
AL139008.2	0.999871	0.093435
ST13P19	0.999871	0.093435
NLGN1-AS1	0.999871	0.093435
AC096639.1	0.999871	0.093435
LINC02048	0.999871	0.093435
AL731568.1	0.999871	0.093435
EFCAB14P1	0.999871	0.093435
GAS2L1P2	0.999871	0.093435
AL109741.2	0.999871	0.093435
AL353689.1	0.999871	0.093435
AC013436.1	0.999871	0.093435
NA	0.999871	0.093435
AL157385.1	0.999871	0.093435
NANOGP2	0.999871	0.093435
AL023755.1	0.999871	0.093435
SRGAP3-AS2	0.999871	0.093435
AC003101.1	0.999871	0.093435
AC005077.3	0.999871	0.093435
OR1H1P	0.999871	0.093435
AC006483.1	0.999871	0.093435
EXOSC3P1	0.999871	0.093435
NA	0.999871	0.093435
AC114488.1	0.999871	0.093435
AL096701.2	0.999871	0.093435
AC009495.1	0.999871	0.093435
AL033527.1	0.999871	0.093435
RPL37P18	0.999871	0.093435
HMGN1P37	0.999871	0.093435
HNRNPA1P8	0.999871	0.093435
ST13P13	0.999871	0.093435
PES1P2	0.999871	0.093435
AC069154.1	0.999871	0.093435
BX322557.1	0.999871	0.093435
KSR1P1	0.999871	0.093435
GAS2L1P1	0.999871	0.093435
AC003986.1	0.999871	0.093435
LINC01739	0.999871	0.093435
AL391822.1	0.999871	0.093435
AC018644.1	0.999871	0.093435
CRPPA-AS1	0.999871	0.093435
AL109924.1	0.999871	0.093435
AC140481.1	0.999871	0.093435
RPL7P46	0.999871	0.093435
FAM90A26	0.999871	0.093435
MTND2P2	0.999871	0.093435

AL590683.1	0.999871	0.093435
AC004854.1	0.999871	0.093435
AL451074.3	0.999871	0.093435
LINC01349	0.999871	0.093435
AC060773.1	0.999871	0.093435
AC124057.1	0.999871	0.093435
AC073370.1	0.999871	0.093435
CCT5P2	0.999871	0.093435
PPIAP40	0.999871	0.093435
RSL24D1P2	0.999871	0.093435
AL445933.1	0.999871	0.093435
AC140479.2	0.999871	0.093435
AL807761.4	0.999871	0.093435
ARHGAP26-IT1	0.999871	0.093435
AL160175.1	0.999871	0.093435
C2orf27AP3	0.999871	0.093435
RPL23AP15	0.999871	0.093435
AL137803.1	0.999871	0.093435
LINC01507	0.999871	0.093435
AP000356.1	0.999871	0.093435
AC005703.1	0.999871	0.093435
ARHGAP42P2	0.999871	0.093435
AC011752.1	0.999871	0.093435
TRERNA1	0.999871	0.093435
AC010745.3	0.999871	0.093435
SBK3	0.999871	0.093435
AC007395.1	0.999871	0.093435
AC004941.2	0.999871	0.093435
LINC01353	0.999871	0.093435
E2F6P4	0.999871	0.093435
LINC01698	0.999871	0.093435
NANOGP5	0.999871	0.093435
COX6CP2	0.999871	0.093435
AL596223.2	0.999871	0.093435
NA	0.999871	0.093435
HS1BP3-IT1	0.999871	0.093435
AF131215.1	0.999871	0.093435
AL590103.1	0.999871	0.093435
NA	0.999871	0.093435
FAAHP1	0.999871	0.093435
AL691447.2	0.999871	0.093435
AL122008.2	0.999871	0.093435
AL390728.5	0.999871	0.093435
AF241726.1	0.999871	0.093435
SKA2P1	0.999871	0.093435
AL583856.1	0.999871	0.093435
RPL5P6	0.999871	0.093435
AC004882.1	0.999871	0.093435
NA	0.999871	0.093435
ADH5P2	0.999871	0.093435

HNRNPDP1	0.999871	0.093435
SDCBPP3	0.999871	0.093435
LINC01189	0.999871	0.093435
AC110994.1	0.999871	0.093435
HMGN2P35	0.999871	0.093435
AC083864.2	0.999871	0.093435
DIRC3-AS1	0.999871	0.093435
RPL12P29	0.999871	0.093435
MTND6P9	0.999871	0.093435
AL139120.1	0.999871	0.093435
AL356124.2	0.999871	0.093435
AL035401.1	0.999871	0.093435
AL133480.1	0.999871	0.093435
AL035414.1	0.999871	0.093435
AC005162.2	0.999871	0.093435
AL356981.1	0.999871	0.093435
BTF3P5	0.999871	0.093435
JTBP1	0.999871	0.093435
C4A-AS1	0.999871	0.093435
AL451062.2	0.999871	0.093435
OR6L1P	0.999871	0.093435
AC107083.1	0.999871	0.093435
LINC00284	0.999871	0.093435
AC104339.1	0.999871	0.093435
AL589843.2	0.999871	0.093435
KCNQ5-IT1	0.999871	0.093435
AL513188.1	0.999871	0.093435
AC016907.2	0.999871	0.093435
AL118496.1	0.999871	0.093435
AL606517.1	0.999871	0.093435
LINC01761	0.999871	0.093435
ATP8A2P3	0.999871	0.093435
IGKV3OR2-268	0.999871	0.093435
AL157834.2	0.999871	0.093435
AL596275.1	0.999871	0.093435
RPL7P36	0.999871	0.093435
LINC01868	0.999871	0.093435
AC243772.3	0.999871	0.093435
COX7BP2	0.999871	0.093435
AL109809.2	0.999871	0.093435
GAPDHP49	0.999871	0.093435
AC073587.1	0.999871	0.093435
AL161719.1	0.999871	0.093435
FABP5P14	0.999871	0.093435
RPL7P11	0.999871	0.093435
AC053503.4	0.999871	0.093435
ACTBP13	0.999871	0.093435
LINC02092	0.999871	0.093435
AP002381.1	0.999871	0.093435
NDUFA5P3	0.999871	0.093435

MTND5P26	0.999871	0.093435
GRID1-AS1	0.999871	0.093435
SDHCP3	0.999871	0.093435
NA	0.999871	0.093435
AL441963.1	0.999871	0.093435
AL731661.1	0.999871	0.093435
AL513331.1	0.999871	0.093435
AC073909.1	0.999871	0.093435
GAPDHP52	0.999871	0.093435
AL445235.1	0.999871	0.093435
AL022318.1	0.999871	0.093435
OR6E1P	0.999871	0.093435
Z97353.2	0.999871	0.093435
MRPS10P1	0.999871	0.093435
AL135902.2	0.999871	0.093435
AC104837.1	0.999871	0.093435
AC114501.2	0.999871	0.093435
KATNBL1P3	0.999871	0.093435
CASP17P	0.999871	0.093435
FTLP1	0.999871	0.093435
AGAP1-IT1	0.999871	0.093435
AC018442.1	0.999871	0.093435
NA	0.999871	0.093435
AC010148.1	0.999871	0.093435
AL139231.1	0.999871	0.093435
AP001625.2	0.999871	0.093435
AC079779.3	0.999871	0.093435
NA	0.999871	0.093435
AC108472.1	0.999871	0.093435
AC007405.2	0.999871	0.093435
HMGN2P28	0.999871	0.093435
PRKX-AS1	0.999871	0.093435
AL356108.1	0.999871	0.093435
AL122008.4	0.999871	0.093435
NDUFB9P2	0.999871	0.093435
AL831737.1	0.999871	0.093435
RPL5P9	0.999871	0.093435
AC092573.2	0.999871	0.093435
AC007881.2	0.999871	0.093435
PKMP1	0.999871	0.093435
AL390026.1	0.999871	0.093435
STARD13-AS	0.999871	0.093435
AP004290.1	0.999871	0.093435
S100A11P2	0.999871	0.093435
LINC02556	0.999871	0.093435
AL590302.2	0.999871	0.093435
AL136529.1	0.999871	0.093435
RPL31P63	0.999871	0.093435
LINC01186	0.999871	0.093435
LINC00299	0.999871	0.093435

BX321878.1	0.999871	0.093435
AC126124.2	0.999871	0.093435
NA	0.999871	0.093435
MTND2P11	0.999871	0.093435
AC007098.2	0.999871	0.093435
AC099796.2	0.999871	0.093435
AC019080.2	0.999871	0.093435
IGHD1-7	0.999871	0.093435
AC104777.2	0.999871	0.093435
AL157832.2	0.999871	0.093435
PPIAP67	0.999871	0.093435
MTCO1P19	0.999871	0.093435
AC104849.1	0.999871	0.093435
LINC00407	0.999871	0.093435
AF064858.2	0.999871	0.093435
VDAC1P11	0.999871	0.093435
NA	0.999871	0.093435
NA	0.999871	0.093435
RPL21P109	0.999871	0.093435
RPL23AP33	0.999871	0.093435
RPS15AP29	0.999871	0.093435
AL606489.2	0.999871	0.093435
LINC01885	0.999871	0.093435
AC024559.1	0.999871	0.093435
NA	0.999871	0.093435
AL356121.1	0.999871	0.093435
AL121904.1	0.999871	0.093435
AL445465.2	0.999871	0.093435
AC017079.2	0.999871	0.093435
TARDBPP2	0.999871	0.093435
MKRN4P	0.999871	0.093435
AL512288.2	0.999871	0.093435
TBC1D3P1	0.999871	0.093435
NA	0.999871	0.093435
RF01210	0.999871	0.093435
RNU7-48P	0.999871	0.093435
NA	0.999871	0.093435
NA	0.999871	0.093435
NA	0.999871	0.093435
NA	0.999871	0.093435
NA	0.999871	0.093435
NA	0.999871	0.093435
NA	0.999871	0.093435
NA	0.999871	0.093435
NA	0.999871	0.093435
NA	0.999871	0.093435
NA	0.999871	0.093435
RNU7-113P	0.999871	0.093435
SCARNA18B	0.999871	0.093435
NA	0.999871	0.093435

NA	0.999871	0.093435
NA	0.999871	0.093435
NA	0.999871	0.093435
RNU7-45P	0.999871	0.093435
NA	0.999871	0.093435
NA	0.999871	0.093435
NA	0.999871	0.093435
NA	0.999871	0.093435
NA	0.999871	0.093435
NA	0.999871	0.093435
NA	0.999871	0.093435
NA	0.999871	0.093435
NA	0.999871	0.093435
NA	0.999871	0.093435
NA	0.999871	0.093435
NA	0.999871	0.093435
AC093484.1	0.999871	0.093435
AC069439.1	0.999871	0.093435
NA	0.999871	0.093435
AC146507.1	0.999871	0.093435
RPL9P5	0.999871	0.093435
RPL32P34	0.999871	0.093435
RN7SL668P	0.999871	0.093435
RN7SL688P	0.999871	0.093435
RN7SL825P	0.999871	0.093435
RPS10P16	0.999871	0.093435
DDX50P2	0.999871	0.093435
AC069431.1	0.999871	0.093435
RN7SL771P	0.999871	0.093435
AF305872.1	0.999871	0.093435
KIR3DL2	0.999871	0.093435
NA	0.999871	0.093435
AC091544.1	0.999871	0.093435
AP001024.1	0.999871	0.093435
RPS27P21	0.999871	0.093435
PLCXD2-AS1	0.999871	0.093435
AC026877.1	0.999871	0.093435
AC009153.1	0.999871	0.093435
RPS29P22	0.999871	0.093435
AC025271.1	0.999871	0.093435
AC020633.1	0.999871	0.093435
NA	0.999871	0.093435
NA	0.999871	0.093435
AL035454.1	0.999871	0.093435
CYP51A1P1	0.999871	0.093435
RN7SL160P	0.999871	0.093435
RPL39P38	0.999871	0.093435
AC092468.1	0.999871	0.093435
AC092757.1	0.999871	0.093435
NA	0.999871	0.093435

NA	0.999871	0.093435
RN7SL434P	0.999871	0.093435
RPS29P21	0.999871	0.093435
WWTR1-IT1	0.999871	0.093435
AC114982.2	0.999871	0.093435
RPL36AP41	0.999871	0.093435
RN7SL152P	0.999871	0.093435
SERBP1P3	0.999871	0.093435
AC099542.2	0.999871	0.093435
ABCF2P1	0.999871	0.093435
RPL5P29	0.999871	0.093435
AK2P2	0.999871	0.093435
AC022400.3	0.999871	0.093435
AC048334.1	0.999871	0.093435
AC121764.1	0.999871	0.093435
NA	0.999871	0.093435
AC107398.1	0.999871	0.093435
AC127024.1	0.999871	0.093435
RN7SL37P	0.999871	0.093435
OR7E100P	0.999871	0.093435
DCAF13P1	0.999871	0.093435
RPL12P21	0.999871	0.093435
RPL5P12	0.999871	0.093435
AC104687.1	0.999871	0.093435
RPS26P21	0.999871	0.093435
RN7SL413P	0.999871	0.093435
AL390726.1	0.999871	0.093435
RPS3P7	0.999871	0.093435
AC093663.2	0.999871	0.093435
AC106707.2	0.999871	0.093435
RPS29P19	0.999871	0.093435
NA	0.999871	0.093435
AMY2A	0.999871	0.093435
AC078785.3	0.999871	0.093435
MIR1302-2HG	0.999871	0.093435
IGLVIVOR22-2	0.999871	0.093435
AL121721.1	0.999871	0.093435
RN7SL67P	0.999871	0.093435
RN7SL487P	0.999871	0.093435
TUBA4B	0.999871	0.093435
RN7SL430P	0.999871	0.093435
AC125604.1	0.999871	0.093435
AC026410.2	0.999871	0.093435
LINC01322	0.999871	0.093435
AC128688.1	0.999871	0.093435
AC005532.2	0.999871	0.093435
AC113367.1	0.999871	0.093435
FOXP1-AS1	0.999871	0.093435
AC133134.1	0.999871	0.093435
FAM3D-AS1	0.999871	0.093435

KRTAP5-7	0.999871	0.093435
AC087884.1	0.999871	0.093435
AC108676.1	0.999871	0.093435
RN7SL47P	0.999871	0.093435
RN7SL153P	0.999871	0.093435
RPL15P21	0.999871	0.093435
PPM1K-DT	0.999871	0.093435
LINC01498	0.999871	0.093435
AL359220.1	0.999871	0.093435
AL136537.1	0.999871	0.093435
STX18-IT1	0.999871	0.093435
FOSL1P1	0.999871	0.093435
AC114781.1	0.999871	0.093435
LINC01962	0.999871	0.093435
AC084024.2	0.999871	0.093435
LINC02115	0.999871	0.093435
ARL4AP2	0.999871	0.093435
LINC02432	0.999871	0.093435
AC079226.2	0.999871	0.093435
NA	0.999871	0.093435
ICE2P1	0.999871	0.093435
AL136360.1	0.999871	0.093435
AL159163.1	0.999871	0.093435
AL359273.1	0.999871	0.093435
NCOA4P3	0.999871	0.093435
ATP6V1G1P6	0.999871	0.093435
AC117383.1	0.999871	0.093435
MLLT10P2	0.999871	0.093435
LINC01179	0.999871	0.093435
AC117383.2	0.999871	0.093435
AC113385.1	0.999871	0.093435
CCDC37-DT	0.999871	0.093435
AC026434.1	0.999871	0.093435
AC108210.2	0.999871	0.093435
AC097467.1	0.999871	0.093435
AC007016.1	0.999871	0.093435
NCOA4P2	0.999871	0.093435
AC106864.1	0.999871	0.093435
RARRES2P4	0.999871	0.093435
SH3TC2-DT	0.999871	0.093435
MRPL22P1	0.999871	0.093435
LINC01340	0.999871	0.093435
GYPB	0.999871	0.093435
TRIM75P	0.999871	0.093435
AC097460.2	0.999871	0.093435
AC117532.1	0.999871	0.093435
AC093909.3	0.999871	0.093435
AC104685.1	0.999871	0.093435
AC092436.4	0.999871	0.093435
AC055733.2	0.999871	0.093435

AC098679.1	0.999871	0.093435
AC114781.3	0.999871	0.093435
AC008629.2	0.999871	0.093435
AL121796.1	0.999871	0.093435
AC010255.3	0.999871	0.093435
LINC01181	0.999871	0.093435
AC113155.1	0.999871	0.093435
AL391280.1	0.999871	0.093435
AC133961.1	0.999871	0.093435
AC093677.1	0.999871	0.093435
NUDT19P5	0.999871	0.093435
LINC02228	0.999871	0.093435
NA	0.999871	0.093435
MAGI2-AS1	0.999871	0.093435
COQ10BP2	0.999871	0.093435
PRB1	0.999871	0.093435
NA	0.999871	0.093435
RN7SKP67	0.999871	0.093435
RNU2-13P	0.999871	0.093435
RNU5E-3P	0.999871	0.093435
RNA5SP385	0.999871	0.093435
RNU6-1294P	0.999871	0.093435
RF00019	0.999871	0.093435
MIR1343	0.999871	0.093435
RN7SKP179	0.999871	0.093435
NA	0.999871	0.093435
RNU6-1333P	0.999871	0.093435
RNU7-154P	0.999871	0.093435
RF00438	0.999871	0.093435
RF00322	0.999871	0.093435
RF00019	0.999871	0.093435
NA	0.999871	0.093435
RF00090	0.999871	0.093435
RNU6-1338P	0.999871	0.093435
MIR2278	0.999871	0.093435
RNU6-479P	0.999871	0.093435
RNU6-781P	0.999871	0.093435
RF00019	0.999871	0.093435
RNU6-977P	0.999871	0.093435
RF00611	0.999871	0.093435
SNORD116-30	0.999871	0.093435
RNY4	0.999871	0.093435
RNY4P20	0.999871	0.093435
RNU6-1171P	0.999871	0.093435
NA	0.999871	0.093435
RNU6-250P	0.999871	0.093435
RF00569	0.999871	0.093435
NA	0.999871	0.093435
RNU6-1096P	0.999871	0.093435
NA	0.999871	0.093435

NA	0.999871	0.093435
RNU6-731P	0.999871	0.093435
MIR2116	0.999871	0.093435
RPL12P22	0.999871	0.093435
AC124069.1	0.999871	0.093435
AC023632.1	0.999871	0.093435
AC104997.1	0.999871	0.093435
AC090281.1	0.999871	0.093435
AC004080.1	0.999871	0.093435
AC018992.1	0.999871	0.093435
AC016868.1	0.999871	0.093435
AC023200.1	0.999871	0.093435
CDC42P5	0.999871	0.093435
AC022915.1	0.999871	0.093435
AF121898.1	0.999871	0.093435
AC136628.3	0.999871	0.093435
PYDC2	0.999871	0.093435
SUMO2P18	0.999871	0.093435
AC008456.1	0.999871	0.093435
AC120036.2	0.999871	0.093435
AC144568.2	0.999871	0.093435
HIGD1AP18	0.999871	0.093435
NA	0.999871	0.093435
ALG1L10P	0.999871	0.093435
AC105150.1	0.999871	0.093435
AC021613.1	0.999871	0.093435
AC022733.2	0.999871	0.093435
AC011676.3	0.999871	0.093435
AC068189.1	0.999871	0.093435
ANK3-DT	0.999871	0.093435
TDGF1P5	0.999871	0.093435
AC087203.1	0.999871	0.093435
SF3A3P2	0.999871	0.093435
AC090592.1	0.999871	0.093435
AC015689.1	0.999871	0.093435
AP003097.1	0.999871	0.093435
AC027018.1	0.999871	0.093435
NAV2-AS4	0.999871	0.093435
AP002370.1	0.999871	0.093435
FAM8A2P	0.999871	0.093435
GVINP2	0.999871	0.093435
AF131215.3	0.999871	0.093435
AC009806.1	0.999871	0.093435
AP000867.2	0.999871	0.093435
MORF4L1P3	0.999871	0.093435
AP003110.1	0.999871	0.093435
AP003385.4	0.999871	0.093435
AP004833.2	0.999871	0.093435
AC023442.3	0.999871	0.093435
AP000907.1	0.999871	0.093435

AP002370.2	0.999871	0.093435
SMILR	0.999871	0.093435
AP002008.3	0.999871	0.093435
AL161668.2	0.999871	0.093435
AC036111.3	0.999871	0.093435
AP003123.1	0.999871	0.093435
AP003733.3	0.999871	0.093435
AC018653.1	0.999871	0.093435
NA	0.999871	0.093435
AL583722.1	0.999871	0.093435
AC022080.3	0.999871	0.093435
AP001880.1	0.999871	0.093435
MTRNR2L3	0.999871	0.093435
SMIM3	0.999871	0.093435
AC007450.1	0.999871	0.093435
AP006289.1	0.999871	0.093435
AC007655.1	0.999871	0.093435
MRGPRF-AS1	0.999871	0.093435
LINC02393	0.999871	0.093435
AC025423.3	0.999871	0.093435
AP002991.1	0.999871	0.093435
STH	0.999871	0.093435
ATP5MFP4	0.999871	0.093435
AC092821.2	0.999871	0.093435
LINC02417	0.999871	0.093435
AC126178.1	0.999871	0.093435
AC008147.1	0.999871	0.093435
AC084364.1	0.999871	0.093435
AC133555.1	0.999871	0.093435
AC009248.1	0.999871	0.093435
ST13P3	0.999871	0.093435
AC090525.2	0.999871	0.093435
AC078776.1	0.999871	0.093435
AC063947.2	0.999871	0.093435
AC079600.3	0.999871	0.093435
AC007513.1	0.999871	0.093435
AC138932.2	0.999871	0.093435
SLC25A3P2	0.999871	0.093435
AL158801.3	0.999871	0.093435
EIF3LP1	0.999871	0.093435
AL132712.1	0.999871	0.093435
AC007376.2	0.999871	0.093435
SNRPGP1	0.999871	0.093435
AL583810.1	0.999871	0.093435
AL133368.2	0.999871	0.093435
LINC00637	0.999871	0.093435
AL691403.1	0.999871	0.093435
AL133153.2	0.999871	0.093435
OR11H7	0.999871	0.093435
AL110505.1	0.999871	0.093435

FDPSP3	0.999871	0.093435
LINC02321	0.999871	0.093435
NA	0.999871	0.093435
AC243965.1	0.999871	0.093435
AL049869.1	0.999871	0.093435
AL356804.1	0.999871	0.093435
AC091544.3	0.999871	0.093435
LINC00911	0.999871	0.093435
NDUFA3P4	0.999871	0.093435
AC022710.1	0.999871	0.093435
LINC00927	0.999871	0.093435
AC009997.1	0.999871	0.093435
AC025040.2	0.999871	0.093435
TGIF2-RAB5IF	0.999871	0.093435
AC093334.1	0.999871	0.093435
NA	0.999871	0.093435
KRT8P9	0.999871	0.093435
AC084882.1	0.999871	0.093435
AC013489.2	0.999871	0.093435
AC012050.1	0.999871	0.093435
UBE2Q2P8	0.999871	0.093435
AC021351.1	0.999871	0.093435
NCAPGP2	0.999871	0.093435
AC023825.1	0.999871	0.093435
AC007342.1	0.999871	0.093435
AC103876.1	0.999871	0.093435
ZNF720P1	0.999871	0.093435
AC007494.1	0.999871	0.093435
MRPS21P7	0.999871	0.093435
PCMTD1P2	0.999871	0.093435
AC068135.2	0.999871	0.093435
SUB1P4	0.999871	0.093435
ACTG1P16	0.999871	0.093435
TPRKBP2	0.999871	0.093435
AC004381.1	0.999871	0.093435
AC092681.2	0.999871	0.093435
AC010207.1	0.999871	0.093435
AC020658.3	0.999871	0.093435
AC010542.1	0.999871	0.093435
AC093536.1	0.999871	0.093435
RN7SKP176	0.999871	0.093435
AL121839.2	0.999871	0.093435
NA	0.999871	0.093435
AC019294.3	0.999871	0.093435
AC025678.2	0.999871	0.093435
LINC01228	0.999871	0.093435
LINC02178	0.999871	0.093435
LINC01989	0.999871	0.093435
AC133919.2	0.999871	0.093435
HMGB1P33	0.999871	0.093435

RNF126P1	0.999871	0.093435
AC136944.2	0.999871	0.093435
LINC02252	0.999871	0.093435
AL353803.4	0.999871	0.093435
PYCARD-AS1	0.999871	0.093435
AC136619.2	0.999871	0.093435
AL591222.1	0.999871	0.093435
DPPA2P4	0.999871	0.093435
AC135048.1	0.999871	0.093435
AL158211.1	0.999871	0.093435
AGGF1P8	0.999871	0.093435
KRT18P18	0.999871	0.093435
MIA	0.999871	0.093435
AC004148.1	0.999871	0.093435
BCAR4	0.999871	0.093435
MTND4LP24	0.999871	0.093435
MCUR1P1	0.999871	0.093435
AC027763.1	0.999871	0.093435
LINC00621	0.999871	0.093435
AC127521.1	0.999871	0.093435
AC099684.3	0.999871	0.093435
EIF4A1P9	0.999871	0.093435
AC087501.3	0.999871	0.093435
HNRNPCP4	0.999871	0.093435
AC015853.3	0.999871	0.093435
NA	0.999871	0.093435
MIR3973	0.999871	0.093435
MIR4753	0.999871	0.093435
AC015908.2	0.999871	0.093435
HID1-AS1	0.999871	0.093435
NA	0.999871	0.093435
MIR4802	0.999871	0.093435
SNORA70D	0.999871	0.093435
AC026620.1	0.999871	0.093435
NA	0.999871	0.093435
AC079336.1	0.999871	0.093435
MIR3115	0.999871	0.093435
NA	0.999871	0.093435
NA	0.999871	0.093435
LINC01543	0.999871	0.093435
NA	0.999871	0.093435
NA	0.999871	0.093435
RN7SL678P	0.999871	0.093435
AC024267.1	0.999871	0.093435
MIR3928	0.999871	0.093435
NA	0.999871	0.093435
NA	0.999871	0.093435
AC055811.2	0.999871	0.093435
NA	0.999871	0.093435
NA	0.999871	0.093435

MIR4786	0.999871	0.093435
NA	0.999871	0.093435
MIR4671	0.999871	0.093435
MIR3183	0.999871	0.093435
NA	0.999871	0.093435
NA	0.999871	0.093435
NA	0.999871	0.093435
NA	0.999871	0.093435
MIR4294	0.999871	0.093435
NA	0.999871	0.093435
NA	0.999871	0.093435
AC015845.1	0.999871	0.093435
NA	0.999871	0.093435
NA	0.999871	0.093435
RN7SL622P	0.999871	0.093435
MIR3202-1	0.999871	0.093435
MIR4790	0.999871	0.093435
RN7SL693P	0.999871	0.093435
NA	0.999871	0.093435
NA	0.999871	0.093435
MIR3139	0.999871	0.093435
NA	0.999871	0.093435
NA	0.999871	0.093435
MIR4460	0.999871	0.093435
MIR4284	0.999871	0.093435
KYNUP2	0.999871	0.093435
AC069366.2	0.999871	0.093435
NA	0.999871	0.093435
NA	0.999871	0.093435
MIR4735	0.999871	0.093435
MIR4659A	0.999871	0.093435
NA	0.999871	0.093435
MIR5192	0.999871	0.093435
AC007431.2	0.999871	0.093435
MIR4525	0.999871	0.093435
NA	0.999871	0.093435
RN7SL163P	0.999871	0.093435
AC121320.1	0.999871	0.093435
NA	0.999871	0.093435
AC053481.3	0.999871	0.093435
RN7SL619P	0.999871	0.093435
GAPLINC	0.999871	0.093435
AC104996.2	0.999871	0.093435
AC006116.1	0.999871	0.093435
SLC25A6P4	0.999871	0.093435
KRT18P61	0.999871	0.093435
RPL10P15	0.999871	0.093435
AC008738.2	0.999871	0.093435
AC006116.3	0.999871	0.093435
AC005777.1	0.999871	0.093435

LINC01140	0.999871	0.093435
AC004672.2	0.999871	0.093435
AC011511.4	0.999871	0.093435
AC060766.3	0.999871	0.093435
ZNF833P	0.999871	0.093435
EIF5AP3	0.999871	0.093435
AC006305.2	0.999871	0.093435
AC005180.1	0.999871	0.093435
NA	0.999871	0.093435
AC008759.1	0.999871	0.093435
AC104423.1	0.999871	0.093435
AC010632.2	0.999871	0.093435
AP002414.5	0.999871	0.093435
AC107896.1	0.999871	0.093435
NA	0.999871	0.093435
NA	0.999871	0.093435
AC010616.1	0.999871	0.093435
FMR1-AS1	0.999871	0.093435
NA	0.999871	0.093435
AC123912.1	0.999871	0.093435
ZNF725P	0.999871	0.093435
PCGF7P	0.999871	0.093435
NA	0.999871	0.093435
NIFKP6	0.999871	0.093435
AIRN	0.999871	0.093435
AC123912.3	0.999871	0.093435
BNIP3P25	0.999871	0.093435
NA	0.999871	0.093435
NA	0.999871	0.093435
AC005261.5	0.999871	0.093435
NA	0.999871	0.093435
NA	0.999871	0.093435
NA	0.999871	0.093435
AL161896.1	0.999871	0.093435
NA	0.999871	0.093435
NA	0.999871	0.093435
CCDC194	0.999871	0.093435
AC092070.4	0.999871	0.093435
MIR1470	0.999871	0.093435
AL162424.1	0.999871	0.093435
Z93241.1	0.999871	0.093435
NA	0.999871	0.093435
AC136475.7	0.999871	0.093435
TSNAX-DISC1	0.999871	0.093435
NA	0.999871	0.093435
AL022097.1	0.999871	0.093435
AC104692.2	0.999871	0.093435
NA	0.999871	0.093435
AC008725.1	0.999871	0.093435
AC145423.1	0.999871	0.093435

AL365400.2	0.999871	0.093435
KF455155.1	0.999871	0.093435
AC136443.5	0.999871	0.093435
AC092120.2	0.999871	0.093435
NA	0.999871	0.093435
AC008277.2	0.999871	0.093435
HSPE1P7	0.999871	0.093435
MAGOH3P	0.999871	0.093435
AC087045.1	0.999871	0.093435
AL513548.2	0.999871	0.093435
IMMP1LP3	0.999871	0.093435
AF146191.1	0.999871	0.093435
AC023206.1	0.999871	0.093435
RBISP2	0.999871	0.093435
NA	0.999871	0.093435
AL109809.4	0.999871	0.093435
AC005070.2	0.999871	0.093435
AC005183.1	0.999871	0.093435
AC069304.3	0.999871	0.093435
AC019072.1	0.999871	0.093435
AC008555.6	0.999871	0.093435
AC104771.1	0.999871	0.093435
AC012640.3	0.999871	0.093435
AF287957.1	0.999871	0.093435
RN7SKP4	0.999871	0.093435
MIR4791	0.999871	0.093435
MIR4466	0.999871	0.093435
NA	0.999871	0.093435
RNU6-85P	0.999871	0.093435
AC098614.3	0.999871	0.093435
NA	0.999871	0.093435
AC025171.4	0.999871	0.093435
RN7SL803P	0.999871	0.093435
NA	0.999871	0.093435
AC096721.1	0.999871	0.093435
AP003355.2	0.999871	0.093435
AC092354.1	0.999871	0.093435
AL160408.5	0.999871	0.093435
AL591167.1	0.999871	0.093435
AC016405.3	0.999871	0.093435
AC113194.1	0.999871	0.093435
AC254633.1	0.999871	0.093435
AC016542.1	0.999871	0.093435
STAG3L5P-PVRIG2P-PILRB	0.999871	0.093435
AP000864.1	0.999871	0.093435
AL022334.2	0.999871	0.093435
AP001269.4	0.999871	0.093435
AC092954.2	0.999871	0.093435
NA	0.999871	0.093435
TM4SF19-TCTEX1D2	0.999871	0.093435

AC004921.1	0.999871	0.093435
AC006566.1	0.999871	0.093435
AL137796.1	0.999871	0.093435
AL139124.1	0.999871	0.093435
CNP	-0.02681	0.09343
CCDC92	-0.0349	0.093409
PTGES3L	0.093175	0.093408
SELENOF	0.029112	0.093357
CAVIN2	0.249036	0.093329
GFAP	0.391861	0.093311
NAF1	-0.05138	0.093295
AC131392.1	-0.1387	0.093278
GTPBP3	0.033599	0.093274
AC105339.4	0.264426	0.093259
MACROD2	0.144863	0.093239
CDCA7	0.04378	0.093211
ATP6V1D	0.033755	0.093205
NA	-0.50578	0.093189
ZNF491	-0.11952	0.093184
COMMD8	-0.0523	0.093181
AC010542.2	0.116397	0.09317
AC016586.2	0.189332	0.093142
SECISBP2L	-0.03157	0.093105
FAM228B	0.044398	0.093076
BTBD1	0.031035	0.093071
TCF15	0.141235	0.093026
LRRC37A17P	0.048897	0.093023
AC008427.1	-0.57816	0.093016
GCOM1	0.441157	0.093013
RMDN2	-0.05255	0.093007
FAM193B	-0.03591	0.092997
AC015908.3	-0.25268	0.092991
CHGA	0.067946	0.092988
ETV4	0.040294	0.092973
AP000753.2	-0.12757	0.092969
RUVBL1	0.027084	0.092953
ACSL5	-0.23351	0.09295
CHMP1B-AS1	-0.0443	0.092947
RNA5SP494	-0.40457	0.092944
PALD1	-0.05219	0.092926
RESF1	0.038845	0.092925
MAST1	-0.04066	0.092923
NA	0.4928	0.092912
SULF2	0.050455	0.092834
CENPH	-0.03469	0.092777
UNKL	-0.03478	0.092768
CXCL11	0.221704	0.092761
RPL41P5	-0.29516	0.092758
STK4	-0.0271	0.092749
ZNF518A	0.041037	0.092696

AC018738.1	-0.51607	0.092689
ADAMTS3	-0.12504	0.092682
AL136038.4	-0.22222	0.092666
AL162413.1	0.172666	0.092647
RPL29P14	0.529736	0.092638
MMS19	-0.02767	0.092632
KCNIP3	0.174147	0.092609
BBX	0.031835	0.092605
GNA15	0.317046	0.092574
AC027612.1	0.743315	0.092571
AC007731.3	0.743315	0.092571
PDSS2	0.052696	0.092563
MINDY3	-0.03885	0.092544
TGFBI	-0.07182	0.092539
NA	0.386525	0.092538
CXXC4-AS1	-0.39966	0.092524
DPP10	0.774667	0.092487
RPL7AP65	0.774667	0.092487
NA	0.774667	0.092487
TANC1	0.029847	0.092485
ID2	0.027127	0.092482
SLAIN2	-0.03159	0.092432
NA	-0.05894	0.092431
HOXC10	0.297548	0.092405
DPY19L1P1	-0.05915	0.092401
TPTEP2	-0.10485	0.092372
CASP2	0.030125	0.092365
C2orf92	-0.08754	0.09235
ACY1	-0.10584	0.092328
SH2D3C	0.056276	0.092308
AC135731.2	0.456026	0.092282
WDR89	-0.04591	0.092282
STAR	-0.13457	0.092275
NDUFAF5	0.047229	0.09226
CLUL1	-0.17889	0.092248
NIN	0.027195	0.092235
SYCE1L	-0.10329	0.092198
PNRC2P1	-0.36374	0.092194
AC008555.2	-0.13601	0.092186
NA	0.128795	0.09218
RBMS2P1	0.456721	0.092157
NTN1	0.333604	0.092155
RCSD1	0.301789	0.092149
STX16	0.028225	0.092131
AC112236.2	-0.32936	0.092129
BHLHA15	0.278277	0.092085
TBR1	-0.95686	0.092077
RNA5SP213	-0.95686	0.092077
AP000354.1	-0.95686	0.092077
MTND4LP9	-0.95686	0.092077

AL162419.1	-0.95686	0.092077
AL033539.1	-0.95686	0.092077
BX284668.5	-0.95686	0.092077
KRT8P13	-0.95686	0.092077
NA	-0.95686	0.092077
AC112496.1	-0.95686	0.092077
AC092903.2	-0.95686	0.092077
RNA5SP20	-0.95686	0.092077
AC016245.1	-0.95686	0.092077
MIR5091	-0.95686	0.092077
NA	-0.95686	0.092077
IGKV1OR2-118	-0.95686	0.092077
AC011481.2	-0.26703	0.092076
ACAP3	-0.03517	0.092073
INPPL1	-0.03651	0.092071
SYS1	-0.04667	0.09205
TARS2	-0.03735	0.092024
AC132938.2	-0.31213	0.091987
AL353751.1	0.155471	0.091943
PLCXD3	0.042793	0.09192
AC015971.1	-0.0497	0.091903
ELL2	-0.06427	0.091875
ADAMTS20	-0.2357	0.091844
AL591806.3	0.185309	0.091835
LINC00941	-0.21383	0.091808
TMTC4	0.038419	0.091806
KIAA2012-AS1	-0.35835	0.091773
EVA1A	0.039407	0.091768
AL034550.1	0.086698	0.091754
RBM22	-0.0286	0.091748
LINC01301	0.314648	0.09174
COG7	-0.0441	0.091718
AC008443.3	0.095386	0.091715
OGFRP1	-0.74727	0.091688
LINC02362	-0.74727	0.091688
SCYL1	0.034558	0.091671
ZNF616	-0.04857	0.091655
KIAA1324L	0.029974	0.091643
SLC39A6	0.025509	0.091596
PSMD5	-0.03199	0.091536
ZNF284	0.055393	0.091535
AC024937.2	0.380535	0.091527
AL354707.2	0.734852	0.091524
ROPN1	-0.88573	0.091494
SMARCE1P6	-0.88573	0.091494
AL137856.1	-0.88573	0.091494
AC104794.1	-0.88573	0.091494
UBE2CP1	-0.88573	0.091494
NA	-0.88573	0.091494
SUV39H2	0.040244	0.09149

HERC1	-0.02815	0.091485
AC097263.1	0.379345	0.091482
AC093752.3	-0.24158	0.091461
INSM1	0.463877	0.091455
RPS15AP11	-0.60191	0.091451
NA	0.14089	0.091395
PHF10	-0.0285	0.091395
MTLN	-0.06648	0.091373
ZNRD1	-0.04457	0.091355
AL391244.2	-0.06972	0.091348
POGZ	-0.02583	0.091338
ZNF317	-0.03724	0.091316
DNAJC9-AS1	-0.0387	0.091312
ZBED8	-0.06202	0.09131
CRISPLD2	0.063989	0.091283
TNXA	0.399376	0.091257
TICRR	0.038308	0.091249
UFC1	0.026779	0.091234
AP005136.2	-0.21942	0.091226
RNU6-322P	-0.38212	0.091222
UNC119B	0.027842	0.091215
RRN3	0.026293	0.091197
GGH	0.043121	0.091108
NA	-0.41294	0.091106
RPS4XP17	-0.42597	0.091105
HCG9	-0.57586	0.09109
SLC16A2	-0.05193	0.091062
TRMT61B	0.033448	0.091055
METTL15P1	-0.74648	0.09102
AL591479.1	-0.74648	0.09102
METTL21EP	-0.74648	0.09102
ZNF19	0.077539	0.090962
PCYOX1L	-0.05232	0.090955
FSCN2	-0.17564	0.090939
RASAL1	-0.88137	0.09092
CCR9	-0.88137	0.09092
LINC02036	-0.88137	0.09092
LINC01450	-0.88137	0.09092
RN7SL795P	-0.88137	0.09092
RN7SL798P	-0.88137	0.09092
NA	-0.88137	0.09092
RF01293	-0.88137	0.09092
AC007598.1	-0.88137	0.09092
NOP14-AS1	-0.03235	0.090895
KIF20B	0.041763	0.090878
AC068481.1	-0.2853	0.090857
AC253536.3	0.268099	0.090807
ZNF431	0.04376	0.090803
AC010503.1	0.198642	0.090777
WDR41	0.033154	0.090773

EZR	0.027384	0.09077
KIF18A	0.049829	0.090761
TOMM20P4	-0.28956	0.090746
RNFT1	-0.05591	0.090733
SLC39A9	0.024152	0.090697
OSBPL5	-0.07038	0.090686
NNMT	0.13248	0.090677
AC132008.2	-0.04332	0.090671
RNA5SP108	0.360532	0.090651
PSMC6	0.03225	0.090649
AC025165.2	0.039275	0.090608
DOC2B	-0.09298	0.090605
NADK2-AS1	0.254091	0.090603
IGHMBP2	0.050412	0.090577
SPDYE12P	-0.11412	0.090572
AL022324.2	-0.21575	0.090571
SPTBN2	-0.03388	0.090563
AC007541.1	-0.11621	0.090535
AC007375.2	-0.27061	0.090527
FUT7	-0.39181	0.090476
AF131215.6	-0.06241	0.090473
SHANK3	0.056095	0.09046
CCDC103	-0.10648	0.090456
SNX18P3	0.453124	0.090446
PTCHD4	0.149668	0.090445
RUSC2	-0.03832	0.090377
MLKL	-0.11606	0.090376
CFL2	-0.03433	0.09036
AC017079.1	-0.95741	0.090346
SRP72P2	-0.95741	0.090346
CFAP299	-0.95741	0.090346
RF00019	-0.95741	0.090346
RNVU1-6	-0.95741	0.090346
AC021218.1	-0.95741	0.090346
GAGE10	-0.95741	0.090346
ABHD17AP6	-0.95741	0.090346
LRRC3-DT	-0.95741	0.090346
TEX53	-0.95741	0.090346
XXYLT1-AS2	-0.95741	0.090346
RPL36AP15	-0.95741	0.090346
AC007283.2	-0.95741	0.090346
NA	-0.95741	0.090346
RN7SL569P	-0.95741	0.090346
RN7SL213P	-0.95741	0.090346
AC083982.1	-0.95741	0.090346
TRPC7-AS1	-0.95741	0.090346
RNA5SP395	-0.95741	0.090346
RNA5SP268	-0.95741	0.090346
AC090136.3	-0.95741	0.090346
AC234917.1	-0.95741	0.090346

HNRNPA1P45	-0.95741	0.090346
HSPE1P5	-0.95741	0.090346
AL590084.3	-0.95741	0.090346
AC103810.4	-0.95741	0.090346
AL161729.2	-0.95741	0.090346
AL353135.1	-0.95741	0.090346
MIR421	0.32718	0.0903
AP003031.1	0.290883	0.090293
SLC25A14	-0.042	0.090284
AC091390.1	0.441249	0.090282
MOSMO	-0.03488	0.090255
RPL7AP31	-0.73761	0.09024
NA	-0.73761	0.09024
RPS3AP29	-0.69378	0.09021
FEV	0.047544	0.090201
PCCA	0.042917	0.090196
UBQLN1	-0.02651	0.090173
KBTBD6	-0.04237	0.090152
GHR	0.680172	0.090147
BRPF3	0.032671	0.090139
ZC3HC1	0.033328	0.090126
LY6E-DT	0.322429	0.09008
ERCC6L2	-0.03809	0.090068
C1D	0.028501	0.090024
AL157702.2	-0.55692	0.090014
MUC20-OT1	-0.0515	0.090013
RAB41	-0.13233	0.089981
HSP90AA4P	0.497519	0.089962
MLH3	-0.03684	0.089933
DNAL4	0.064727	0.089924
PLET1	0.667489	0.089917
RF00139	0.667489	0.089917
KLHL21	0.052327	0.089914
RF00019	0.528137	0.0899
AC006017.1	0.05556	0.089886
ATP7A	-0.04025	0.089846
ZNF282	0.032728	0.089845
CHCHD4	-0.0431	0.089802
HIST3H2BA	-0.32409	0.089759
AL161911.1	-0.33427	0.089712
ZMIZ2	-0.03552	0.089704
PRSS37	0.578638	0.089684
AC092953.2	0.092242	0.089669
COX8A	0.030032	0.089648
AC007193.3	-0.05633	0.089639
SNCB	-0.31189	0.089639
HNRNPCP1	-0.12973	0.089635
CUL4B	-0.02598	0.08963
CCDC188	-0.04566	0.089623
CTAGE3P	-0.41368	0.089579

HIF1AN	0.023617	0.089571
PFKL	0.030489	0.089557
TMEM8A	-0.03747	0.089547
LINC00266-1	-0.88595	0.08953
IL1RAP	-0.88595	0.08953
MIR1250	-0.88595	0.08953
AC113391.2	-0.88595	0.08953
NA	-0.88595	0.08953
AP001189.3	-0.88595	0.08953
AC061975.7	-0.88595	0.08953
GDI2	0.028689	0.089527
AL645728.1	-0.46011	0.089495
RHOF	0.045393	0.089487
NPIPB3	0.092776	0.089471
LIMCH1	-0.03053	0.089469
CXCL2	-0.13891	0.089461
RNU6-48P	0.545383	0.089445
NDUFB11	0.025458	0.089428
CASP8	-0.08478	0.089413
AL031777.1	-0.23895	0.089411
TMEM59	0.025475	0.089409
TRAF3IP2	-0.09666	0.089383
ERICH6-AS1	0.188319	0.089344
TMEM79	0.036603	0.089343
PLEKHM1	0.050186	0.089318
DTL	-0.04201	0.089306
RNF165	0.041493	0.089302
LBH	-0.08058	0.089301
AC136604.3	-0.26541	0.089291
TBC1D25	0.054648	0.089289
ASAP2	-0.02773	0.089288
ORMDL1	0.032539	0.089279
AC011611.4	0.354927	0.08925
EPHB1	-0.12976	0.089237
ZCCHC18	-0.06373	0.089234
COPB1	0.028776	0.08923
NA	0.675993	0.089225
AC013470.3	0.675993	0.089225
RPL7P42	0.675993	0.089225
AL132656.3	0.675993	0.089225
NPIPP1	-0.11147	0.089204
AL158834.1	0.33792	0.089195
MAPK15	0.075262	0.089189
LDB1	-0.0299	0.08918
RPS6KA4	-0.04469	0.089175
GTF2H2B	0.061256	0.089141
CASTOR3	-0.04945	0.089134
RF00019	-0.17151	0.089134
NA	-0.06048	0.089131
LINC00504	0.242898	0.089121

LTN1	0.032897	0.089111
BMS1P15	0.346954	0.08911
AC007622.2	-0.06647	0.089106
SMCO2	-0.72377	0.089082
AC107294.3	-0.72377	0.089082
DSTNP2	0.100649	0.089029
RPS6KA1	-0.05174	0.089028
FAM32A	0.025701	0.089027
RNU4-32P	-0.88156	0.088964
RF00019	-0.88156	0.088964
RNU6V	-0.88156	0.088964
RF00019	-0.88156	0.088964
CHL1-AS2	-0.88156	0.088964
UBE2D3P3	-0.88156	0.088964
ETF1P1	-0.88156	0.088964
RNY4P25	-0.88156	0.088964
WDR45P1	-0.88156	0.088964
PIGFP1	-0.88156	0.088964
AC020978.4	-0.88156	0.088964
AL022476.1	-0.12348	0.08896
AC005486.1	-0.34429	0.08895
NA	-0.67329	0.088943
AHSA2P	0.025566	0.088935
RBM6	-0.02396	0.088918
SART1	-0.03166	0.088917
PEAK3	0.465531	0.088871
ANKRD42	-0.04037	0.088856
PAICSP1	0.249865	0.08879
Z74021.1	0.272091	0.088771
GATAD1	0.032176	0.088768
RNU6-623P	0.716981	0.088763
RPS3AP49	0.716981	0.088763
NA	-0.02676	0.088761
NA	-0.43037	0.088727
C8orf37	-0.08151	0.088585
ACSF3	0.037847	0.088565
UPK2	0.279365	0.088515
PDCD11	-0.0251	0.088471
ECE2	-0.04466	0.08845
LRPAP1	-0.02999	0.088436
AL137186.2	0.043948	0.088383
AC016394.2	0.078187	0.088382
NA	-0.20889	0.088382
SNW1	0.028127	0.088361
ALG1L9P	0.104023	0.08836
DBF4	0.034814	0.088326
ZNF607	-0.05132	0.088309
RF00019	0.505923	0.088308
DYNC1I2P1	0.34323	0.088271
FAM238B	-0.05368	0.088263

AC010531.6	-0.25701	0.08826
AC007326.1	0.471307	0.08824
ZNF678	0.041474	0.088217
LMNB2	-0.02315	0.088188
CSTF2	0.033144	0.088172
AC145207.1	-0.43984	0.088161
RGS20	0.088343	0.088157
RPL13AP7	0.350392	0.08815
STK32A	0.330341	0.088149
NPC1L1	-0.88611	0.088127
PROK2	-0.88611	0.088127
GPR141	-0.88611	0.088127
RF00019	-0.88611	0.088127
AC013549.1	-0.88611	0.088127
GUSBP11	-0.88611	0.088127
AC099548.2	-0.88611	0.088127
AL138752.2	-0.88611	0.088127
TSPAN9-IT1	-0.88611	0.088127
AL391261.2	-0.88611	0.088127
SNORD53	-0.88611	0.088127
AL445209.1	-0.88611	0.088127
AC008750.4	-0.05771	0.088126
DGUOK-AS1	0.034684	0.088106
SEMA4F	0.046875	0.088103
BMP2K	-0.0361	0.088093
SORD	-0.0291	0.088074
TMEM81	-0.07704	0.088055
METTL7B	-0.20653	0.08804
SLC26A6	-0.04881	0.088025
PTPMT1	0.037098	0.087998
H2AFJ	0.461136	0.087992
CLIP3	-0.04709	0.087972
AP002907.1	0.067176	0.087965
SMIM20	0.049235	0.087961
SLC25A24	0.032475	0.087959
LINC01776	-0.27463	0.087951
AP001062.2	0.503903	0.087943
LAT2	-0.10894	0.087943
CENPP	-0.03171	0.0879
VPS13D	-0.03445	0.087891
ATP5F1EP2	0.437182	0.087882
THAP6	0.05152	0.087871
RN7SKP38	0.549665	0.087855
NA	-0.17014	0.087838
RBBP7	0.025302	0.087818
GLCCI1	0.034554	0.087801
CARS2	-0.02941	0.087784
DHRS2	0.312476	0.08775
DEPDC7	-0.08375	0.087735
C22orf23	0.10794	0.087697

LINC02604	0.073802	0.087696
RBX1	0.069842	0.087685
RPS6KC1	0.025575	0.087645
AP006287.2	-0.19274	0.08763
SOGA3	0.14326	0.087596
SPRYD3	-0.0428	0.087593
ANKRD16	0.054893	0.087585
FAM209A	-0.8817	0.087562
MATN4	-0.8817	0.087562
SPDEF	-0.8817	0.087562
LRIT3	-0.8817	0.087562
AL031283.1	-0.8817	0.087562
RPL23AP50	-0.8817	0.087562
ST6GALNAC4P1	-0.8817	0.087562
HNRNPLP1	-0.8817	0.087562
CICP13	-0.8817	0.087562
NA	-0.8817	0.087562
LINC02631	-0.8817	0.087562
AL121820.1	-0.8817	0.087562
AC007787.1	-0.8817	0.087562
VIL1	-0.04787	0.087538
FIZ1	-0.03318	0.087537
GSDMB	-0.05746	0.087506
DHRSX-IT1	-0.21622	0.087501
LDLR	0.10624	0.087445
AC023161.2	0.563979	0.087422
AC239804.1	-0.14733	0.087413
HNRNPH1P1	0.208557	0.08741
AC007285.1	-0.21092	0.087405
NA	-0.06531	0.087402
EFCAB12	0.307082	0.087392
NA	-0.16128	0.087388
GPR161	-0.03461	0.087381
PELI3	-0.05256	0.087357
ERMARD	-0.04309	0.087356
AC011337.1	-0.19486	0.087351
NA	0.233277	0.087343
PSMC5	-0.02245	0.087298
AC007193.1	0.146869	0.087294
AC104596.1	-0.16313	0.087287
EPG5	0.030633	0.08727
AL121753.1	-0.41129	0.087235
AL355483.1	0.135856	0.087151
TMEM119	0.041435	0.08715
NCK1	0.043578	0.08715
PLEKHG2	0.038866	0.087122
HIPK1-AS1	0.650417	0.087113
FGFBP3	0.070118	0.087103
HES7	0.115502	0.087103
RPS6KA3	-0.029	0.0871

NA	0.29441	0.087085
LINC01703	0.169311	0.087084
TMEM204	0.079774	0.087076
OSGEPL1-AS1	-0.11468	0.087074
ADGRE2	-0.49179	0.087072
RPUSD1	0.034468	0.08702
ANAPC1	-0.03075	0.08697
PSMD14	-0.02691	0.086915
PIK3R2	0.591969	0.086903
RF00019	0.591969	0.086903
GPR34	-0.62189	0.086901
LTB4R2	-0.62189	0.086901
SCN9A	0.034403	0.086896
MRPL52	0.029468	0.08687
TAF1A	0.034323	0.086859
AC016773.1	0.130312	0.086839
LINC02193	-0.18385	0.086815
AC233728.1	-0.316	0.086804
NA	-0.23606	0.086804
AL360181.1	0.376999	0.086781
EIF1AXP1	0.176265	0.086749
AC069234.2	-0.03231	0.086729
ZSCAN9	-0.03467	0.086713
COPS3	-0.02734	0.086692
AL359317.1	-0.27172	0.086692
AC011479.3	-0.59403	0.086689
LINC00862	0.372495	0.086683
RABIF	0.041085	0.086653
AL050404.1	-0.10386	0.086562
USP49	0.037901	0.086546
NA	0.211681	0.086507
AL158152.1	-0.08874	0.08648
USF1	0.026298	0.08647
TBC1D32	0.048665	0.086458
MIR302D	0.434142	0.086458
CPO	0.921602	0.086435
CXCR6	0.921602	0.086435
HNRNPCL4	0.921602	0.086435
RNU4-23P	0.921602	0.086435
HIST2H2AA3	0.921602	0.086435
SNORA69	0.921602	0.086435
ANXA2P1	0.921602	0.086435
AL360271.1	0.921602	0.086435
PGGT1BP2	0.921602	0.086435
BTF3P7	0.921602	0.086435
C7orf71	0.921602	0.086435
NA	0.921602	0.086435
ADGRF5P1	0.921602	0.086435
AL162734.1	0.921602	0.086435
AC011005.1	0.921602	0.086435

PSMC1P3	0.921602	0.086435
AL162385.2	0.921602	0.086435
AC092652.1	0.921602	0.086435
AC074183.1	0.921602	0.086435
NA	0.921602	0.086435
FTH1P25	0.921602	0.086435
AF165147.1	0.921602	0.086435
DNM3-IT1	0.921602	0.086435
ELOBP4	0.921602	0.086435
LINC01505	0.921602	0.086435
LDHAP3	0.921602	0.086435
OR2L9P	0.921602	0.086435
AC097533.1	0.921602	0.086435
AC090602.1	0.921602	0.086435
RN7SL239P	0.921602	0.086435
AC100803.1	0.921602	0.086435
AC016642.1	0.921602	0.086435
AC010280.2	0.921602	0.086435
ALG1L12P	0.921602	0.086435
AC087359.1	0.921602	0.086435
LINC01605	0.921602	0.086435
AC023232.1	0.921602	0.086435
AP002989.1	0.921602	0.086435
PPIAP43	0.921602	0.086435
AK6P2	0.921602	0.086435
NA	0.921602	0.086435
RN7SL605P	0.921602	0.086435
AC090415.1	0.921602	0.086435
AC015818.5	0.921602	0.086435
MIR744	0.921602	0.086435
MIR4254	0.921602	0.086435
AC040904.1	0.921602	0.086435
HNRNPA1P52	0.921602	0.086435
AC008972.2	0.921602	0.086435
LRRC59	-0.02285	0.086424
SDF4	0.030456	0.086408
KCNIP2-AS1	-0.13582	0.086389
AC009227.1	0.195766	0.086377
AC016820.1	-0.46423	0.08637
MRPL35P2	0.294793	0.086366
AC092587.1	0.598325	0.086365
AC004898.1	-0.42161	0.086363
BCAR3	-0.0507	0.086361
ARSB	-0.03923	0.086348
HABP4	0.036687	0.086335
AC112907.2	0.396183	0.086328
AC008802.1	0.608859	0.086325
LAMP5-AS1	-0.06399	0.086316
ME3	0.062246	0.086279
RBM41	-0.04155	0.086271

AL513327.2	-0.1973	0.08627
FAM227A	-0.07833	0.086269
TRMT6	-0.02764	0.086251
ANKH	-0.03371	0.086246
LY6E	-0.0323	0.086245
CALM2	-0.03454	0.086205
FADS2	-0.03198	0.086191
NA	-0.06275	0.086183
AL353743.4	-0.54179	0.086182
FBXO9	0.029842	0.086162
ZEB1	-0.06904	0.086129
AC026689.1	-0.2275	0.086114
LILRB3	-0.48707	0.08611
ADPRH	-0.11429	0.086099
AC067930.4	0.089679	0.08609
AC020922.3	0.34037	0.086057
FRMD5	0.048231	0.086039
AC023824.3	-0.34992	0.086038
EIF4A1	0.053411	0.086013
FSCN3	-0.33376	0.086008
TOPORS	0.037887	0.085962
WDR55	0.024206	0.085929
BORCS5	0.078005	0.085929
HLA-DPA1	-0.4119	0.085924
ACOT9	0.037729	0.085921
TSPAN10	-0.10248	0.085916
COPS4	-0.0323	0.085896
MRPL23	0.029718	0.08589
KIAA1191	-0.0258	0.085877
NA	-0.08027	0.085875
CDC42BPA	-0.0285	0.08587
AC019069.1	-0.06086	0.085835
KCNH8	-0.05826	0.085829
SEPT7P2	0.036612	0.085823
PRPF4B	-0.02784	0.085788
GRAP	0.389417	0.085765
ANKRD2	0.655104	0.085724
FGR	-0.92368	0.085701
SLC4A1	-0.92368	0.085701
PRSS22	-0.92368	0.085701
ANOS1	-0.92368	0.085701
CYP3A43	-0.92368	0.085701
C8B	-0.92368	0.085701
FLT4	-0.92368	0.085701
CDH10	-0.92368	0.085701
ANO2	-0.92368	0.085701
COL17A1	-0.92368	0.085701
TRHDE	-0.92368	0.085701
ARHGAP15	-0.92368	0.085701
RDH8	-0.92368	0.085701

CACNA1S	-0.92368	0.085701
GP6	-0.92368	0.085701
NXPE1	-0.92368	0.085701
TREM2	-0.92368	0.085701
SLC17A9	-0.92368	0.085701
CACNA1F	-0.92368	0.085701
NA	-0.92368	0.085701
CGB2	-0.92368	0.085701
ATP4A	-0.92368	0.085701
HPN	-0.92368	0.085701
MEOX2	-0.92368	0.085701
SH3GL2	-0.92368	0.085701
FGF8	-0.92368	0.085701
P2RX1	-0.92368	0.085701
CCL1	-0.92368	0.085701
NA	-0.92368	0.085701
SLC16A6	-0.92368	0.085701
IL2	-0.92368	0.085701
APOC3	-0.92368	0.085701
PPM1H	-0.92368	0.085701
SCNN1A	-0.92368	0.085701
SLC17A2	-0.92368	0.085701
LY86	-0.92368	0.085701
IL5	-0.92368	0.085701
PRKAG3	-0.92368	0.085701
SLC9A2	-0.92368	0.085701
CD80	-0.92368	0.085701
SASH3	-0.92368	0.085701
BTN1A1	-0.92368	0.085701
OVOL2	-0.92368	0.085701
RFPL2	-0.92368	0.085701
KRT17	-0.92368	0.085701
GALNT8	-0.92368	0.085701
CRB3	-0.92368	0.085701
TNS4	-0.92368	0.085701
IQCA1	-0.92368	0.085701
ALDH3B2	-0.92368	0.085701
GPR12	-0.92368	0.085701
GRP	-0.92368	0.085701
EHF	-0.92368	0.085701
GPR55	-0.92368	0.085701
RTP4	-0.92368	0.085701
RBP4	-0.92368	0.085701
MSTN	-0.92368	0.085701
C4orf17	-0.92368	0.085701
ERP27	-0.92368	0.085701
IL22RA1	-0.92368	0.085701
PROK1	-0.92368	0.085701
HMCN1	-0.92368	0.085701
LEFTY2	-0.92368	0.085701

SLC10A6	-0.92368	0.085701
NKD2	-0.92368	0.085701
SPINK7	-0.92368	0.085701
GLRA1	-0.92368	0.085701
CRIP3	-0.92368	0.085701
CHRNA6	-0.92368	0.085701
DOK2	-0.92368	0.085701
ST8SIA6	-0.92368	0.085701
MPZL2	-0.92368	0.085701
GPHA2	-0.92368	0.085701
CCDC83	-0.92368	0.085701
IL18	-0.92368	0.085701
SLC25A31	-0.92368	0.085701
MARVELD2	-0.92368	0.085701
SAXO1	-0.92368	0.085701
SLC28A1	-0.92368	0.085701
KCNJ15	-0.92368	0.085701
PALM2-AKAP2	-0.92368	0.085701
SLC34A2	-0.92368	0.085701
GRHL3	-0.92368	0.085701
OR10J6P	-0.92368	0.085701
GOLGA6A	-0.92368	0.085701
SIGLEC11	-0.92368	0.085701
CD300LG	-0.92368	0.085701
WDR63	-0.92368	0.085701
MT2P1	-0.92368	0.085701
INHBB	-0.92368	0.085701
SLC6A20	-0.92368	0.085701
CALHM4	-0.92368	0.085701
RAET1E	-0.92368	0.085701
EN2	-0.92368	0.085701
ASPG	-0.92368	0.085701
CYYR1	-0.92368	0.085701
CCDC182	-0.92368	0.085701
AC010323.1	-0.92368	0.085701
KLHL30	-0.92368	0.085701
MUCL3	-0.92368	0.085701
ZBBX	-0.92368	0.085701
IL1RAPL1	-0.92368	0.085701
CD52	-0.92368	0.085701
AL391987.1	-0.92368	0.085701
TRH	-0.92368	0.085701
OR1L8	-0.92368	0.085701
RXFP1	-0.92368	0.085701
CD8B	-0.92368	0.085701
SLC9C1	-0.92368	0.085701
PRL	-0.92368	0.085701
GPR148	-0.92368	0.085701
XCR1	-0.92368	0.085701
AC095040.1	-0.92368	0.085701

NA	-0.92368	0.085701
ZNF80	-0.92368	0.085701
EVX2	-0.92368	0.085701
INHBC	-0.92368	0.085701
HIGD2B	-0.92368	0.085701
SFN	-0.92368	0.085701
HMGB4	-0.92368	0.085701
OR10AB1P	-0.92368	0.085701
LY6H	-0.92368	0.085701
BEND2	-0.92368	0.085701
AC021054.1	-0.92368	0.085701
VCX2	-0.92368	0.085701
TEX44	-0.92368	0.085701
AC112247.1	-0.92368	0.085701
ZNRF3-AS1	-0.92368	0.085701
SPINK6	-0.92368	0.085701
FAM230I	-0.92368	0.085701
CPN2	-0.92368	0.085701
RPS2P28	-0.92368	0.085701
ARL14	-0.92368	0.085701
AC079741.1	-0.92368	0.085701
GPR139	-0.92368	0.085701
FER1L6-AS1	-0.92368	0.085701
MTHFD2P7	-0.92368	0.085701
TNFSF15	-0.92368	0.085701
TIGIT	-0.92368	0.085701
TMEM30B	-0.92368	0.085701
ENPP7	-0.92368	0.085701
NCF1B	-0.92368	0.085701
NA	-0.92368	0.085701
AC091046.1	-0.92368	0.085701
WFDC10B	-0.92368	0.085701
FREM3	-0.92368	0.085701
CTNNA3	-0.92368	0.085701
CCDC60	-0.92368	0.085701
REC114	-0.92368	0.085701
OVCH2	-0.92368	0.085701
RIPK4	-0.92368	0.085701
CCR3	-0.92368	0.085701
AC026410.1	-0.92368	0.085701
RALYL	-0.92368	0.085701
UNC93B5	-0.92368	0.085701
RPL7AP49	-0.92368	0.085701
FLRT2	-0.92368	0.085701
AQP12B	-0.92368	0.085701
C12orf56	-0.92368	0.085701
LINC00158	-0.92368	0.085701
SMIM23	-0.92368	0.085701
SOWAHB	-0.92368	0.085701
CYP4X1	-0.92368	0.085701

SPATA12	-0.92368	0.085701
CXCR3	-0.92368	0.085701
KRT16	-0.92368	0.085701
TERF1P2	-0.92368	0.085701
FAM205C	-0.92368	0.085701
OR7D2	-0.92368	0.085701
ALOX15P2	-0.92368	0.085701
FAM230G	-0.92368	0.085701
TMCO2	-0.92368	0.085701
CALHM6	-0.92368	0.085701
C9orf152	-0.92368	0.085701
SBSN	-0.92368	0.085701
GRAPL	-0.92368	0.085701
ZAR1L	-0.92368	0.085701
LINC00994	-0.92368	0.085701
CXCL17	-0.92368	0.085701
ZNF676	-0.92368	0.085701
COLCA1	-0.92368	0.085701
NA	-0.92368	0.085701
GGT3P	-0.92368	0.085701
MDS2	-0.92368	0.085701
AADACL2	-0.92368	0.085701
NA	-0.92368	0.085701
NA	-0.92368	0.085701
NA	-0.92368	0.085701
TATDN2P1	-0.92368	0.085701
LINC02692	-0.92368	0.085701
NA	-0.92368	0.085701
ARHGEF15	-0.92368	0.085701
POU3F3	-0.92368	0.085701
MIRLET7C	-0.92368	0.085701
MIR135B	-0.92368	0.085701
MIR30C2	-0.92368	0.085701
MIR346	-0.92368	0.085701
MIR374A	-0.92368	0.085701
MIR331	-0.92368	0.085701
RN7SKP25	-0.92368	0.085701
RNU6-766P	-0.92368	0.085701
RNY4P19	-0.92368	0.085701
RNU6-562P	-0.92368	0.085701
RNY4P24	-0.92368	0.085701
RF00019	-0.92368	0.085701
RF00019	-0.92368	0.085701
RF00019	-0.92368	0.085701
RNU6-1233P	-0.92368	0.085701
RNA5SP452	-0.92368	0.085701
RF00019	-0.92368	0.085701
RF00019	-0.92368	0.085701
VTRNA1-1	-0.92368	0.085701
RNU6-181P	-0.92368	0.085701

NA	-0.92368	0.085701
RNU6-485P	-0.92368	0.085701
RNU6-1331P	-0.92368	0.085701
RNU6-414P	-0.92368	0.085701
RF00019	-0.92368	0.085701
RF00019	-0.92368	0.085701
RNU1-124P	-0.92368	0.085701
RNA5SP161	-0.92368	0.085701
RF00019	-0.92368	0.085701
RF00019	-0.92368	0.085701
NA	-0.92368	0.085701
RNY1P12	-0.92368	0.085701
NA	-0.92368	0.085701
RF00019	-0.92368	0.085701
RNU4-59P	-0.92368	0.085701
RF00019	-0.92368	0.085701
RNU6-646P	-0.92368	0.085701
RF00019	-0.92368	0.085701
RN7SKP78	-0.92368	0.085701
RNU6-1187P	-0.92368	0.085701
RF00019	-0.92368	0.085701
RF00019	-0.92368	0.085701
RF00019	-0.92368	0.085701
RN7SKP9	-0.92368	0.085701
RF00432	-0.92368	0.085701
RN7SKP153	-0.92368	0.085701
RF00139	-0.92368	0.085701
RF00019	-0.92368	0.085701
NA	-0.92368	0.085701
NA	-0.92368	0.085701
NA	-0.92368	0.085701
NA	-0.92368	0.085701
Z99756.1	-0.92368	0.085701
NA	-0.92368	0.085701
AC106827.1	-0.92368	0.085701
LOR	-0.92368	0.085701
LCN1P2	-0.92368	0.085701
LINC01123	-0.92368	0.085701
C10orf113	-0.92368	0.085701
SPDYC	-0.92368	0.085701
AL935212.1	-0.92368	0.085701
IGFL2	-0.92368	0.085701
NAT8B	-0.92368	0.085701
DEFB136	-0.92368	0.085701
HCP5	-0.92368	0.085701
XKR4	-0.92368	0.085701
RNU6-877P	-0.92368	0.085701
SNORA80B	-0.92368	0.085701
RNU6-444P	-0.92368	0.085701
RNU6-1074P	-0.92368	0.085701

RNU6-1056P	-0.92368	0.085701
RF00019	-0.92368	0.085701
RNU6-142P	-0.92368	0.085701
RF00019	-0.92368	0.085701
RF00019	-0.92368	0.085701
RF00019	-0.92368	0.085701
RNU6-1200P	-0.92368	0.085701
RNY3P14	-0.92368	0.085701
RF00019	-0.92368	0.085701
RNU6-937P	-0.92368	0.085701
RF00019	-0.92368	0.085701
RF00212	-0.92368	0.085701
RNU6-800P	-0.92368	0.085701
RF00019	-0.92368	0.085701
RNU6-900P	-0.92368	0.085701
SNORA37	-0.92368	0.085701
RF00019	-0.92368	0.085701
RNU6-1005P	-0.92368	0.085701
RNY1P4	-0.92368	0.085701
RNA5SP64	-0.92368	0.085701
RF00019	-0.92368	0.085701
RF00019	-0.92368	0.085701
RNU6-541P	-0.92368	0.085701
RNU6-19P	-0.92368	0.085701
NA	-0.92368	0.085701
MIR598	-0.92368	0.085701
MIR554	-0.92368	0.085701
MIR562	-0.92368	0.085701
MIR588	-0.92368	0.085701
MIR648	-0.92368	0.085701
MIR27A	-0.92368	0.085701
MIR34B	-0.92368	0.085701
MIR221	-0.92368	0.085701
MIR624	-0.92368	0.085701
NA	-0.92368	0.085701
SNORA40B	-0.92368	0.085701
MT-TN	-0.92368	0.085701
RNU6ATAC4P	-0.92368	0.085701
TRGC1	-0.92368	0.085701
MIR548U	-0.92368	0.085701
RNA5SP372	-0.92368	0.085701
RNU6-298P	-0.92368	0.085701
RNU6-1154P	-0.92368	0.085701
RF00586	-0.92368	0.085701
RNU6-817P	-0.92368	0.085701
RNU6-1111P	-0.92368	0.085701
RF00554	-0.92368	0.085701
RF00568	-0.92368	0.085701
RNA5SP53	-0.92368	0.085701
AL583805.1	-0.92368	0.085701

AL627422.1	-0.92368	0.085701
NA	-0.92368	0.085701
PDCL3P5	-0.92368	0.085701
AC007066.1	-0.92368	0.085701
IFITM9P	-0.92368	0.085701
SLC25A5P6	-0.92368	0.085701
AC104297.1	-0.92368	0.085701
NA	-0.92368	0.085701
CFL1P2	-0.92368	0.085701
AC108039.1	-0.92368	0.085701
AC103591.1	-0.92368	0.085701
AL137074.1	-0.92368	0.085701
FAM210CP	-0.92368	0.085701
EIF1P7	-0.92368	0.085701
AC107956.2	-0.92368	0.085701
EEF1B2P2	-0.92368	0.085701
RPL22P16	-0.92368	0.085701
SEPHS1P6	-0.92368	0.085701
AC005840.2	-0.92368	0.085701
AC112187.1	-0.92368	0.085701
SNRPEP9	-0.92368	0.085701
CTAGE14P	-0.92368	0.085701
COLCA2	-0.92368	0.085701
RPLP2P1	-0.92368	0.085701
NA	-0.92368	0.085701
SEC14L6	-0.92368	0.085701
AC142086.1	-0.92368	0.085701
POLR3DP1	-0.92368	0.085701
AC010168.1	-0.92368	0.085701
AC114744.1	-0.92368	0.085701
APOC1P1	-0.92368	0.085701
SEM1P1	-0.92368	0.085701
AL772337.1	-0.92368	0.085701
AC011825.1	-0.92368	0.085701
PRDX2P1	-0.92368	0.085701
RPS12P16	-0.92368	0.085701
KRT18P60	-0.92368	0.085701
AL355493.1	-0.92368	0.085701
AL138807.1	-0.92368	0.085701
NA	-0.92368	0.085701
NA	-0.92368	0.085701
AL512637.1	-0.92368	0.085701
KRT18P57	-0.92368	0.085701
ZNF859P	-0.92368	0.085701
NA	-0.92368	0.085701
NA	-0.92368	0.085701
NA	-0.92368	0.085701
AL136968.2	-0.92368	0.085701
AL136116.2	-0.92368	0.085701
AL121949.1	-0.92368	0.085701

NDUFS5P1	-0.92368	0.085701
RPS27P15	-0.92368	0.085701
B3GALNT2P1	-0.92368	0.085701
DBIP1	-0.92368	0.085701
CATSPERZ	-0.92368	0.085701
YAP1P1	-0.92368	0.085701
PGAM1P10	-0.92368	0.085701
AL583834.1	-0.92368	0.085701
DNAJC19P6	-0.92368	0.085701
HIST1H3PS1	-0.92368	0.085701
AL023807.1	-0.92368	0.085701
NA	-0.92368	0.085701
NA	-0.92368	0.085701
MIR548E	-0.92368	0.085701
RNU6ATAC27P	-0.92368	0.085701
NA	-0.92368	0.085701
MIR1208	-0.92368	0.085701
NA	-0.92368	0.085701
NA	-0.92368	0.085701
NA	-0.92368	0.085701
NA	-0.92368	0.085701
MIR1205	-0.92368	0.085701
NA	-0.92368	0.085701
RNU6-1165P	-0.92368	0.085701
NA	-0.92368	0.085701
RNU2-17P	-0.92368	0.085701
RNU6-554P	-0.92368	0.085701
RN7SKP56	-0.92368	0.085701
RF00409	-0.92368	0.085701
RNU2-15P	-0.92368	0.085701
RNU2-42P	-0.92368	0.085701
RN7SKP51	-0.92368	0.085701
RNA5SP25	-0.92368	0.085701
RN7SKP97	-0.92368	0.085701
RF00019	-0.92368	0.085701
RNA5SP155	-0.92368	0.085701
RNA5SP247	-0.92368	0.085701
NA	-0.92368	0.085701
RNU6-1217P	-0.92368	0.085701
RNU6-57P	-0.92368	0.085701
RNU6-954P	-0.92368	0.085701
NA	-0.92368	0.085701
RAD17P2	-0.92368	0.085701
DAZAP2P1	-0.92368	0.085701
AC005006.1	-0.92368	0.085701
NBEAP3	-0.92368	0.085701
AP001048.1	-0.92368	0.085701
AC019070.1	-0.92368	0.085701
LINC01441	-0.92368	0.085701
AC116917.1	-0.92368	0.085701

LINC01449	-0.92368	0.085701
AC025594.1	-0.92368	0.085701
OR2L1P	-0.92368	0.085701
LINC01832	-0.92368	0.085701
WARS2-IT1	-0.92368	0.085701
AC005077.2	-0.92368	0.085701
HMGB3P21	-0.92368	0.085701
LINC01087	-0.92368	0.085701
AL139158.2	-0.92368	0.085701
AC010907.1	-0.92368	0.085701
ATP6V1G1P3	-0.92368	0.085701
NA	-0.92368	0.085701
GAPDHP46	-0.92368	0.085701
AC096541.1	-0.92368	0.085701
AL355336.1	-0.92368	0.085701
TOMM22P5	-0.92368	0.085701
LINC00092	-0.92368	0.085701
CHCHD4P5	-0.92368	0.085701
RPS27AP12	-0.92368	0.085701
AC117490.1	-0.92368	0.085701
AL451074.2	-0.92368	0.085701
NA	-0.92368	0.085701
AL158212.1	-0.92368	0.085701
RPL23AP18	-0.92368	0.085701
AC079630.1	-0.92368	0.085701
RPF2P1	-0.92368	0.085701
MIPEPP1	-0.92368	0.085701
CCRL1P1	-0.92368	0.085701
AL590233.1	-0.92368	0.085701
AL807761.2	-0.92368	0.085701
HNRNPA3P15	-0.92368	0.085701
AL118511.2	-0.92368	0.085701
TAF13P2	-0.92368	0.085701
AC004899.1	-0.92368	0.085701
FAM66E	-0.92368	0.085701
AC017002.2	-0.92368	0.085701
AL122001.1	-0.92368	0.085701
PSG8-AS1	-0.92368	0.085701
BANF1P5	-0.92368	0.085701
AL109741.1	-0.92368	0.085701
C5orf67	-0.92368	0.085701
AC102953.1	-0.92368	0.085701
ZNF503-AS1	-0.92368	0.085701
PHF2P2	-0.92368	0.085701
AC016772.1	-0.92368	0.085701
USP12PX	-0.92368	0.085701
LINC00937	-0.92368	0.085701
AC099342.1	-0.92368	0.085701
FTCDNL1	-0.92368	0.085701
AC007790.1	-0.92368	0.085701

MRPL35P3	-0.92368	0.085701
PTP4A1P3	-0.92368	0.085701
AL022310.1	-0.92368	0.085701
AL365214.2	-0.92368	0.085701
FAM138B	-0.92368	0.085701
MYL6P1	-0.92368	0.085701
SCDP1	-0.92368	0.085701
AC018735.1	-0.92368	0.085701
RPL21P110	-0.92368	0.085701
RPL23AP30	-0.92368	0.085701
AC079341.1	-0.92368	0.085701
LINC00840	-0.92368	0.085701
EEF1GP3	-0.92368	0.085701
LHFPL3-AS1	-0.92368	0.085701
AC092801.1	-0.92368	0.085701
GSTM3P2	-0.92368	0.085701
AC098935.1	-0.92368	0.085701
LINC01203	-0.92368	0.085701
HSPD1P14	-0.92368	0.085701
OR7E101P	-0.92368	0.085701
GTF2IP7	-0.92368	0.085701
Z82205.1	-0.92368	0.085701
SDHDP2	-0.92368	0.085701
RBMX2P5	-0.92368	0.085701
AC011742.1	-0.92368	0.085701
IL21-AS1	-0.92368	0.085701
AL023581.2	-0.92368	0.085701
MIR663AHG	-0.92368	0.085701
AL035551.1	-0.92368	0.085701
AC067942.1	-0.92368	0.085701
PSME2P6	-0.92368	0.085701
TSSK5P	-0.92368	0.085701
AL360013.1	-0.92368	0.085701
MTND6P32	-0.92368	0.085701
AL022100.1	-0.92368	0.085701
SRGAP3-AS3	-0.92368	0.085701
LINC01738	-0.92368	0.085701
IL6R-AS1	-0.92368	0.085701
HMGN2P21	-0.92368	0.085701
AL160408.1	-0.92368	0.085701
AL512504.2	-0.92368	0.085701
LINC01820	-0.92368	0.085701
MYL6P5	-0.92368	0.085701
C1orf143	-0.92368	0.085701
UBBP2	-0.92368	0.085701
GAPDHP45	-0.92368	0.085701
AC079154.1	-0.92368	0.085701
AL929236.1	-0.92368	0.085701
NA	-0.92368	0.085701
AL049792.1	-0.92368	0.085701

PCDH9-AS2	-0.92368	0.085701
KPNA2P2	-0.92368	0.085701
SNRPD2P1	-0.92368	0.085701
SLC25A6P5	-0.92368	0.085701
AC023137.1	-0.92368	0.085701
SLC47A1P1	-0.92368	0.085701
AC234781.3	-0.92368	0.085701
SEPT7P8	-0.92368	0.085701
RPS29P14	-0.92368	0.085701
AL137026.1	-0.92368	0.085701
CYCSP4	-0.92368	0.085701
CR769776.2	-0.92368	0.085701
AL157388.1	-0.92368	0.085701
REV3L-IT1	-0.92368	0.085701
AL031963.2	-0.92368	0.085701
AC007563.1	-0.92368	0.085701
USP24P1	-0.92368	0.085701
AC007349.1	-0.92368	0.085701
SPINK8	-0.92368	0.085701
RLIMP1	-0.92368	0.085701
RPL39P18	-0.92368	0.085701
AC010163.1	-0.92368	0.085701
AL353597.2	-0.92368	0.085701
AL356753.1	-0.92368	0.085701
AC044781.1	-0.92368	0.085701
AL031736.2	-0.92368	0.085701
AL354868.1	-0.92368	0.085701
SNRPFP2	-0.92368	0.085701
ATP8A2P2	-0.92368	0.085701
CCNB1IP1P1	-0.92368	0.085701
AL133412.1	-0.92368	0.085701
AC093899.1	-0.92368	0.085701
AL034418.1	-0.92368	0.085701
AL157373.2	-0.92368	0.085701
AC104695.2	-0.92368	0.085701
SNAP47-AS1	-0.92368	0.085701
UBBP1	-0.92368	0.085701
LINC02643	-0.92368	0.085701
EEF1A1P39	-0.92368	0.085701
AC005518.1	-0.92368	0.085701
AC007952.3	-0.92368	0.085701
AC010422.1	-0.92368	0.085701
MTCO1P42	-0.92368	0.085701
LINC01799	-0.92368	0.085701
RPSAP8	-0.92368	0.085701
TEX48	-0.92368	0.085701
TSEN15P2	-0.92368	0.085701
NA	-0.92368	0.085701
TNPO1P2	-0.92368	0.085701
AL353803.2	-0.92368	0.085701

AC011753.2	-0.92368	0.085701
AC012456.2	-0.92368	0.085701
SRGAP2-AS1	-0.92368	0.085701
CBX1P1	-0.92368	0.085701
RPEP3	-0.92368	0.085701
GOT2P2	-0.92368	0.085701
AL353614.1	-0.92368	0.085701
AC009987.1	-0.92368	0.085701
HMGB1P7	-0.92368	0.085701
COX11P1	-0.92368	0.085701
NA	-0.92368	0.085701
CFAP97D1	-0.92368	0.085701
AL122008.1	-0.92368	0.085701
AL357874.2	-0.92368	0.085701
AC097359.1	-0.92368	0.085701
HMGN2P34	-0.92368	0.085701
RPL34P20	-0.92368	0.085701
RPS6KA2-AS1	-0.92368	0.085701
AL159166.1	-0.92368	0.085701
NA	-0.92368	0.085701
AC012354.2	-0.92368	0.085701
OR5K2	-0.92368	0.085701
AL359885.1	-0.92368	0.085701
MTND5P15	-0.92368	0.085701
HMGB3P31	-0.92368	0.085701
AL731567.1	-0.92368	0.085701
AC096949.1	-0.92368	0.085701
LINC01031	-0.92368	0.085701
AC108059.2	-0.92368	0.085701
LINC00384	-0.92368	0.085701
MTATP6P29	-0.92368	0.085701
CNOT7P2	-0.92368	0.085701
AL139415.1	-0.92368	0.085701
RPS29P8	-0.92368	0.085701
AL138830.2	-0.92368	0.085701
RAD17P1	-0.92368	0.085701
AC009495.2	-0.92368	0.085701
AC112198.2	-0.92368	0.085701
TIMM9P2	-0.92368	0.085701
HSPE1P9	-0.92368	0.085701
MTND3P8	-0.92368	0.085701
TRIM80P	-0.92368	0.085701
AL356320.1	-0.92368	0.085701
MRPL51P2	-0.92368	0.085701
SCAND3P1	-0.92368	0.085701
AL353596.1	-0.92368	0.085701
AL592463.1	-0.92368	0.085701
IPO7P1	-0.92368	0.085701
AL133351.3	-0.92368	0.085701
CICP26	-0.92368	0.085701

NALCN-AS1	-0.92368	0.085701
AL353621.1	-0.92368	0.085701
AL391422.2	-0.92368	0.085701
NA	-0.92368	0.085701
RPL23AP25	-0.92368	0.085701
AC015922.1	-0.92368	0.085701
AC095030.1	-0.92368	0.085701
AL356134.1	-0.92368	0.085701
AL691449.1	-0.92368	0.085701
AC009238.2	-0.92368	0.085701
USP32P2	-0.92368	0.085701
LINC00028	-0.92368	0.085701
U40455.1	-0.92368	0.085701
NA	-0.92368	0.085701
AL391845.2	-0.92368	0.085701
NFU1P2	-0.92368	0.085701
GOT2P3	-0.92368	0.085701
H2AFZP6	-0.92368	0.085701
MTCO1P20	-0.92368	0.085701
NA	-0.92368	0.085701
AP000695.2	-0.92368	0.085701
AC017078.1	-0.92368	0.085701
SETP5	-0.92368	0.085701
GAPDHP22	-0.92368	0.085701
NA	-0.92368	0.085701
AC099066.3	-0.92368	0.085701
AL445224.1	-0.92368	0.085701
COX6CP13	-0.92368	0.085701
LINC01039	-0.92368	0.085701
AC006372.3	-0.92368	0.085701
AL024508.1	-0.92368	0.085701
LINC01426	-0.92368	0.085701
NA	-0.92368	0.085701
VDAC1P3	-0.92368	0.085701
HDAC1P1	-0.92368	0.085701
NA	-0.92368	0.085701
AL162394.1	-0.92368	0.085701
CDC42P1	-0.92368	0.085701
DPP10-AS1	-0.92368	0.085701
PPIAP3	-0.92368	0.085701
VDAC1P4	-0.92368	0.085701
SLC25A5P2	-0.92368	0.085701
TMEM30CP	-0.92368	0.085701
LINC01366	-0.92368	0.085701
TMSB10P2	-0.92368	0.085701
GAPDHP34	-0.92368	0.085701
AC083875.1	-0.92368	0.085701
AC007422.2	-0.92368	0.085701
LLPHP2	-0.92368	0.085701
AC011196.1	-0.92368	0.085701

LINC00533	-0.92368	0.085701
AC073150.1	-0.92368	0.085701
AL365258.2	-0.92368	0.085701
SNTG2-AS1	-0.92368	0.085701
PA2G4P2	-0.92368	0.085701
LINC02250	-0.92368	0.085701
AC012308.1	-0.92368	0.085701
AC138655.1	-0.92368	0.085701
KIF3AP1	-0.92368	0.085701
OR7E145P	-0.92368	0.085701
AC005237.2	-0.92368	0.085701
FAR1P1	-0.92368	0.085701
APOA1-AS	-0.92368	0.085701
MTCO2P11	-0.92368	0.085701
AC008280.1	-0.92368	0.085701
MCCD1P1	-0.92368	0.085701
LINC01814	-0.92368	0.085701
AL357146.1	-0.92368	0.085701
ELFN1-AS1	-0.92368	0.085701
AL136097.2	-0.92368	0.085701
AL031659.1	-0.92368	0.085701
EI24P2	-0.92368	0.085701
SHANK2-AS2	-0.92368	0.085701
NRADDP	-0.92368	0.085701
LINC00608	-0.92368	0.085701
AC074286.1	-0.92368	0.085701
KLF2P4	-0.92368	0.085701
RPS27AP7	-0.92368	0.085701
AC025038.1	-0.92368	0.085701
BAK1P2	-0.92368	0.085701
NA	-0.92368	0.085701
RPL21P23	-0.92368	0.085701
BX322784.1	-0.92368	0.085701
AC244035.2	-0.92368	0.085701
TIMM9P1	-0.92368	0.085701
NA	-0.92368	0.085701
AC130710.1	-0.92368	0.085701
AL121601.2	-0.92368	0.085701
EIF3IP1	-0.92368	0.085701
AC020594.1	-0.92368	0.085701
HUNK-AS1	-0.92368	0.085701
AL445072.1	-0.92368	0.085701
HNRNPA1P2	-0.92368	0.085701
AL356320.2	-0.92368	0.085701
RNF223	-0.92368	0.085701
AL606807.1	-0.92368	0.085701
AL645634.2	-0.92368	0.085701
CECR7	-0.92368	0.085701
AC007384.1	-0.92368	0.085701
AL391832.1	-0.92368	0.085701

AC073284.1	-0.92368	0.085701
UQCRHP2	-0.92368	0.085701
HLCS-IT1	-0.92368	0.085701
LINC00316	-0.92368	0.085701
RPS15AP38	-0.92368	0.085701
Z97987.1	-0.92368	0.085701
AC002075.2	-0.92368	0.085701
LINC01143	-0.92368	0.085701
HNRNPA1P39	-0.92368	0.085701
AL023584.2	-0.92368	0.085701
RPL23AP32	-0.92368	0.085701
AC009227.2	-0.92368	0.085701
AL360169.1	-0.92368	0.085701
AC104170.2	-0.92368	0.085701
AL121908.1	-0.92368	0.085701
ETDA	-0.92368	0.085701
MTND5P20	-0.92368	0.085701
PAGE2B	-0.92368	0.085701
RF01210	-0.92368	0.085701
SNORD126	-0.92368	0.085701
NA	-0.92368	0.085701
NA	-0.92368	0.085701
RNA5SP233	-0.92368	0.085701
NA	-0.92368	0.085701
NA	-0.92368	0.085701
RNU7-54P	-0.92368	0.085701
NA	-0.92368	0.085701
NA	-0.92368	0.085701
NA	-0.92368	0.085701
RNU7-141P	-0.92368	0.085701
RF00019	-0.92368	0.085701
NA	-0.92368	0.085701
NA	-0.92368	0.085701
NA	-0.92368	0.085701
NA	-0.92368	0.085701
NA	-0.92368	0.085701
RNU1-80P	-0.92368	0.085701
NA	-0.92368	0.085701
NA	-0.92368	0.085701
NA	-0.92368	0.085701
RNU7-187P	-0.92368	0.085701
NA	-0.92368	0.085701
NA	-0.92368	0.085701
NA	-0.92368	0.085701
RNA5SP310	-0.92368	0.085701
NA	-0.92368	0.085701
POM121L7P	-0.92368	0.085701
COX6CP14	-0.92368	0.085701
RPL9P30	-0.92368	0.085701
AL023653.1	-0.92368	0.085701

CPHL1P	-0.92368	0.085701
AC091805.1	-0.92368	0.085701
RPS12P27	-0.92368	0.085701
RPL39P26	-0.92368	0.085701
CDKN2B-AS1	-0.92368	0.085701
RPL21P123	-0.92368	0.085701
AL138976.1	-0.92368	0.085701
AC082651.1	-0.92368	0.085701
RN7SL328P	-0.92368	0.085701
RN7SL751P	-0.92368	0.085701
AL161785.3	-0.92368	0.085701
CEACAMP10	-0.92368	0.085701
AC010301.1	-0.92368	0.085701
AC008379.1	-0.92368	0.085701
IQCJ-SCHIP1-AS1	-0.92368	0.085701
ZBTB20-AS2	-0.92368	0.085701
RN7SL344P	-0.92368	0.085701
RN7SL338P	-0.92368	0.085701
RN7SL482P	-0.92368	0.085701
AC091564.1	-0.92368	0.085701
ZNF90P1	-0.92368	0.085701
RN7SL788P	-0.92368	0.085701
DENND6A-DT	-0.92368	0.085701
RPL32P26	-0.92368	0.085701
ALG1L15P	-0.92368	0.085701
AC093583.1	-0.92368	0.085701
RN7SL416P	-0.92368	0.085701
TCP10L	-0.92368	0.085701
AC023906.1	-0.92368	0.085701
KCNAB1-AS1	-0.92368	0.085701
NA	-0.92368	0.085701
ZBTB20-AS4	-0.92368	0.085701
RN7SL749P	-0.92368	0.085701
RPL7P49	-0.92368	0.085701
RPL12P7	-0.92368	0.085701
RPL7P39	-0.92368	0.085701
RN7SL452P	-0.92368	0.085701
RN7SL8P	-0.92368	0.085701
AC084198.1	-0.92368	0.085701
RN7SL370P	-0.92368	0.085701
AC106820.1	-0.92368	0.085701
SMIM34A	-0.92368	0.085701
RN7SL105P	-0.92368	0.085701
RN7SL610P	-0.92368	0.085701
AC087752.1	-0.92368	0.085701
RN7SL735P	-0.92368	0.085701
RPS23P3	-0.92368	0.085701
WWP1P1	-0.92368	0.085701
ILF2P1	-0.92368	0.085701
AC063955.1	-0.92368	0.085701

RN7SL168P	-0.92368	0.085701
RN7SL762P	-0.92368	0.085701
RN7SL242P	-0.92368	0.085701
NA	-0.92368	0.085701
AL049714.1	-0.92368	0.085701
AC025566.1	-0.92368	0.085701
MTHFD2P1	-0.92368	0.085701
AL357153.1	-0.92368	0.085701
LINC01096	-0.92368	0.085701
PLA2G4E-AS1	-0.92368	0.085701
AP000977.1	-0.92368	0.085701
AC010255.1	-0.92368	0.085701
AC068446.2	-0.92368	0.085701
AC010307.2	-0.92368	0.085701
LINC02275	-0.92368	0.085701
AC022447.1	-0.92368	0.085701
LINC02220	-0.92368	0.085701
AC119751.2	-0.92368	0.085701
LINC00992	-0.92368	0.085701
ARHGAP22-IT1	-0.92368	0.085701
THUMPD3P1	-0.92368	0.085701
AC116362.1	-0.92368	0.085701
AL645949.2	-0.92368	0.085701
AC137810.1	-0.92368	0.085701
AL133372.2	-0.92368	0.085701
AC004672.1	-0.92368	0.085701
AC026414.1	-0.92368	0.085701
HNRNPA1P44	-0.92368	0.085701
AC133963.2	-0.92368	0.085701
LINC01333	-0.92368	0.085701
AC023794.3	-0.92368	0.085701
SPATS1	-0.92368	0.085701
AC008629.1	-0.92368	0.085701
AC098799.2	-0.92368	0.085701
RCC2P4	-0.92368	0.085701
AC010280.3	-0.92368	0.085701
BMPR1B-DT	-0.92368	0.085701
AC022092.1	-0.92368	0.085701
HOXC13-AS	-0.92368	0.085701
OR7E94P	-0.92368	0.085701
AC105384.1	-0.92368	0.085701
AC079942.1	-0.92368	0.085701
AC008691.1	-0.92368	0.085701
AC093809.1	-0.92368	0.085701
AC067942.3	-0.92368	0.085701
AC093248.1	-0.92368	0.085701
AC093214.1	-0.92368	0.085701
AC106795.3	-0.92368	0.085701
LINC02509	-0.92368	0.085701
ZNF718	-0.92368	0.085701

IGBP1P4	-0.92368	0.085701
DUTP7	-0.92368	0.085701
AP000808.1	-0.92368	0.085701
AC021678.1	-0.92368	0.085701
LINC01303	-0.92368	0.085701
SMAD1-AS2	-0.92368	0.085701
AC068658.1	-0.92368	0.085701
AC106865.1	-0.92368	0.085701
AC137770.1	-0.92368	0.085701
AC010261.1	-0.92368	0.085701
AL035458.2	-0.92368	0.085701
AC008629.3	-0.92368	0.085701
CUL1P1	-0.92368	0.085701
CTBP2P4	-0.92368	0.085701
AC091885.2	-0.92368	0.085701
AC139491.4	-0.92368	0.085701
AC108159.1	-0.92368	0.085701
AC091849.2	-0.92368	0.085701
AC106760.2	-0.92368	0.085701
AC096711.2	-0.92368	0.085701
AC018680.1	-0.92368	0.085701
AC079140.5	-0.92368	0.085701
NA	-0.92368	0.085701
RF00537	-0.92368	0.085701
SCARNA23	-0.92368	0.085701
RNU4-49P	-0.92368	0.085701
SCARNA11	-0.92368	0.085701
RNU6-189P	-0.92368	0.085701
NA	-0.92368	0.085701
NA	-0.92368	0.085701
RNU6ATAC14P	-0.92368	0.085701
NA	-0.92368	0.085701
RF00598	-0.92368	0.085701
RNU6-1045P	-0.92368	0.085701
RF00019	-0.92368	0.085701
RNU2-58P	-0.92368	0.085701
NA	-0.92368	0.085701
RNU6-589P	-0.92368	0.085701
RNU6-875P	-0.92368	0.085701
NA	-0.92368	0.085701
RNU6-1256P	-0.92368	0.085701
RNU5A-4P	-0.92368	0.085701
NA	-0.92368	0.085701
NA	-0.92368	0.085701
RNU6-307P	-0.92368	0.085701
RN7SKP82	-0.92368	0.085701
NA	-0.92368	0.085701
RNU6-122P	-0.92368	0.085701
RNU7-110P	-0.92368	0.085701
RNU6-1258P	-0.92368	0.085701

NA	-0.92368	0.085701
NA	-0.92368	0.085701
RNU6-64P	-0.92368	0.085701
RNU6-218P	-0.92368	0.085701
RF00019	-0.92368	0.085701
NA	-0.92368	0.085701
NA	-0.92368	0.085701
AC027698.1	-0.92368	0.085701
AC084082.1	-0.92368	0.085701
AC083841.1	-0.92368	0.085701
AC015743.1	-0.92368	0.085701
AC079209.1	-0.92368	0.085701
AC022826.1	-0.92368	0.085701
AC016405.1	-0.92368	0.085701
SRPK2P	-0.92368	0.085701
NDUFA5P2	-0.92368	0.085701
CDH12P3	-0.92368	0.085701
AC009908.1	-0.92368	0.085701
AC018442.2	-0.92368	0.085701
IGHV3-52	-0.92368	0.085701
AC011978.1	-0.92368	0.085701
CERNA3	-0.92368	0.085701
AC144568.1	-0.92368	0.085701
AC093331.1	-0.92368	0.085701
LINC01484	-0.92368	0.085701
AC022973.2	-0.92368	0.085701
AC091182.2	-0.92368	0.085701
AC112191.1	-0.92368	0.085701
AC114550.3	-0.92368	0.085701
AC008619.1	-0.92368	0.085701
IGHVIII-47-1	-0.92368	0.085701
AC131025.1	-0.92368	0.085701
PKMP4	-0.92368	0.085701
AC009597.1	-0.92368	0.085701
AL451137.1	-0.92368	0.085701
AC011726.3	-0.92368	0.085701
GLULP3	-0.92368	0.085701
LINC02235	-0.92368	0.085701
AP000722.1	-0.92368	0.085701
AC104009.1	-0.92368	0.085701
AP001893.2	-0.92368	0.085701
AP001893.3	-0.92368	0.085701
NPIPA2	-0.92368	0.085701
AP003396.4	-0.92368	0.085701
AC090707.1	-0.92368	0.085701
AP001372.3	-0.92368	0.085701
ARL6IP1P3	-0.92368	0.085701
AC036111.2	-0.92368	0.085701
AC099687.1	-0.92368	0.085701
AL136146.2	-0.92368	0.085701

AC108136.1	-0.92368	0.085701
AP003062.1	-0.92368	0.085701
NOX5	-0.92368	0.085701
AC021006.2	-0.92368	0.085701
NA	-0.92368	0.085701
ENPP7P6	-0.92368	0.085701
RAB44	-0.92368	0.085701
AC007406.1	-0.92368	0.085701
AC127070.3	-0.92368	0.085701
LINC01489	-0.92368	0.085701
AP000812.2	-0.92368	0.085701
AC148477.1	-0.92368	0.085701
HSPD1P12	-0.92368	0.085701
AC006927.1	-0.92368	0.085701
ELOCP31	-0.92368	0.085701
CACNA1C-AS4	-0.92368	0.085701
AC008033.1	-0.92368	0.085701
LINC01152	-0.92368	0.085701
AC026333.3	-0.92368	0.085701
AC079866.1	-0.92368	0.085701
LINC02468	-0.92368	0.085701
OR13A1	-0.92368	0.085701
TRDV3	-0.92368	0.085701
AC008115.1	-0.92368	0.085701
LINC02368	-0.92368	0.085701
AC078962.4	-0.92368	0.085701
AC006518.2	-0.92368	0.085701
CACNA1C-AS3	-0.92368	0.085701
AP002383.3	-0.92368	0.085701
AC022509.3	-0.92368	0.085701
NA	-0.92368	0.085701
AC148477.3	-0.92368	0.085701
AC026369.3	-0.92368	0.085701
NA	-0.92368	0.085701
NA	-0.92368	0.085701
AC090709.1	-0.92368	0.085701
AC025161.1	-0.92368	0.085701
AL162311.2	-0.92368	0.085701
AC063924.1	-0.92368	0.085701
AC011595.1	-0.92368	0.085701
OVCH1-AS1	-0.92368	0.085701
BTBD10P1	-0.92368	0.085701
AC090115.1	-0.92368	0.085701
LINC02401	-0.92368	0.085701
AC020656.1	-0.92368	0.085701
NA	-0.92368	0.085701
AC025030.2	-0.92368	0.085701
AC126614.1	-0.92368	0.085701
LINC02396	-0.92368	0.085701
AC089998.4	-0.92368	0.085701

AC073655.1	-0.92368	0.085701
AC125603.3	-0.92368	0.085701
KLF17P1	-0.92368	0.085701
NA	-0.92368	0.085701
AL117190.1	-0.92368	0.085701
NA	-0.92368	0.085701
AC005225.1	-0.92368	0.085701
AL049835.1	-0.92368	0.085701
BANF1P1	-0.92368	0.085701
AL157912.1	-0.92368	0.085701
LINC01500	-0.92368	0.085701
LINC02274	-0.92368	0.085701
LINC01629	-0.92368	0.085701
LINC-ROR	-0.92368	0.085701
AL356020.1	-0.92368	0.085701
AL117192.1	-0.92368	0.085701
LINC02289	-0.92368	0.085701
AL355916.2	-0.92368	0.085701
AL049775.3	-0.92368	0.085701
CHORDC2P	-0.92368	0.085701
AL049869.2	-0.92368	0.085701
PTBP1P	-0.92368	0.085701
AL109628.1	-0.92368	0.085701
AL355075.5	-0.92368	0.085701
AC025040.1	-0.92368	0.085701
AC021739.1	-0.92368	0.085701
LINC02345	-0.92368	0.085701
AC087878.1	-0.92368	0.085701
ST20-MTHFS	-0.92368	0.085701
AC021231.1	-0.92368	0.085701
AC087286.2	-0.92368	0.085701
AC090970.1	-0.92368	0.085701
AC046168.2	-0.92368	0.085701
AC010247.2	-0.92368	0.085701
AC048383.1	-0.92368	0.085701
LINC00052	-0.92368	0.085701
AC068397.1	-0.92368	0.085701
KRT8P23	-0.92368	0.085701
NA	-0.92368	0.085701
EEF1A1P22	-0.92368	0.085701
AC013391.1	-0.92368	0.085701
AC069029.1	-0.92368	0.085701
NA	-0.92368	0.085701
AC097374.1	-0.92368	0.085701
AC011939.1	-0.92368	0.085701
NA	-0.92368	0.085701
AL049838.1	-0.92368	0.085701
AC007608.2	-0.92368	0.085701
AL162632.3	-0.92368	0.085701
AC008567.1	-0.92368	0.085701

MMP2-AS1	-0.92368	0.085701
AP000842.3	-0.92368	0.085701
AC007346.1	-0.92368	0.085701
AL132996.1	-0.92368	0.085701
AC109597.1	-0.92368	0.085701
NA	-0.92368	0.085701
AC009145.1	-0.92368	0.085701
AC126696.3	-0.92368	0.085701
EIF5A2P1	-0.92368	0.085701
Z98885.2	-0.92368	0.085701
CYCSP39	-0.92368	0.085701
AC120498.4	-0.92368	0.085701
AC007494.2	-0.92368	0.085701
AC007224.1	-0.92368	0.085701
AC026150.2	-0.92368	0.085701
AC099506.1	-0.92368	0.085701
AC009107.1	-0.92368	0.085701
AC126323.5	-0.92368	0.085701
AC244034.2	-0.92368	0.085701
NA	-0.92368	0.085701
AC113418.1	-0.92368	0.085701
AP006547.1	-0.92368	0.085701
AC016396.2	-0.92368	0.085701
CLEC19A	-0.92368	0.085701
AC090651.1	-0.92368	0.085701
AC009120.4	-0.92368	0.085701
AL160286.3	-0.92368	0.085701
AC007906.1	-0.92368	0.085701
AC134312.5	-0.92368	0.085701
AC100756.2	-0.92368	0.085701
AC144833.1	-0.92368	0.085701
AC096921.2	-0.92368	0.085701
EEF1A1P38	-0.92368	0.085701
PRSS46P	-0.92368	0.085701
AC022872.1	-0.92368	0.085701
LINC02168	-0.92368	0.085701
LINC00922	-0.92368	0.085701
AC023824.5	-0.92368	0.085701
DNM1P49	-0.92368	0.085701
AC005920.4	-0.92368	0.085701
AC007014.1	-0.92368	0.085701
TVP23CP2	-0.92368	0.085701
MAPK8IP1P1	-0.92368	0.085701
AL354943.1	-0.92368	0.085701
AC007638.2	-0.92368	0.085701
AC009127.2	-0.92368	0.085701
MIR4660	-0.92368	0.085701
NA	-0.92368	0.085701
NA	-0.92368	0.085701
MAPK8IP1P2	-0.92368	0.085701

MIR5582	-0.92368	0.085701
NA	-0.92368	0.085701
NA	-0.92368	0.085701
AF186192.3	-0.92368	0.085701
RF00157	-0.92368	0.085701
OOSP1P2	-0.92368	0.085701
NA	-0.92368	0.085701
MIR4635	-0.92368	0.085701
AC011840.3	-0.92368	0.085701
AC053481.2	-0.92368	0.085701
AC100778.1	-0.92368	0.085701
NA	-0.92368	0.085701
RN7SL850P	-0.92368	0.085701
MIR4672	-0.92368	0.085701
NA	-0.92368	0.085701
NA	-0.92368	0.085701
MIR4762	-0.92368	0.085701
NA	-0.92368	0.085701
NA	-0.92368	0.085701
RN7SL644P	-0.92368	0.085701
MIR3122	-0.92368	0.085701
RN7SL208P	-0.92368	0.085701
AC096708.2	-0.92368	0.085701
MIR4705	-0.92368	0.085701
AC006270.1	-0.92368	0.085701
NA	-0.92368	0.085701
NA	-0.92368	0.085701
NA	-0.92368	0.085701
MIR4257	-0.92368	0.085701
MIR23C	-0.92368	0.085701
SNX19P3	-0.92368	0.085701
RN7SL131P	-0.92368	0.085701
AC103808.2	-0.92368	0.085701
MIR3173	-0.92368	0.085701
MIR4685	-0.92368	0.085701
MIR4504	-0.92368	0.085701
MIR4638	-0.92368	0.085701
VN1R71P	-0.92368	0.085701
RN7SL404P	-0.92368	0.085701
RN7SL45P	-0.92368	0.085701
AL358013.1	-0.92368	0.085701
SNORD3C	-0.92368	0.085701
MIR5094	-0.92368	0.085701
NA	-0.92368	0.085701
AC104996.1	-0.92368	0.085701
NA	-0.92368	0.085701
NA	-0.92368	0.085701
MIR5697	-0.92368	0.085701
NA	-0.92368	0.085701
MIR4421	-0.92368	0.085701

NA	-0.92368	0.085701
MIR548AK	-0.92368	0.085701
RN7SL708P	-0.92368	0.085701
CYP4F35P	-0.92368	0.085701
NA	-0.92368	0.085701
MIR3174	-0.92368	0.085701
MIR4748	-0.92368	0.085701
NA	-0.92368	0.085701
NA	-0.92368	0.085701
AC233702.6	-0.92368	0.085701
SNORA59B	-0.92368	0.085701
AC015908.4	-0.92368	0.085701
NA	-0.92368	0.085701
NA	-0.92368	0.085701
MIR3178	-0.92368	0.085701
MIR5093	-0.92368	0.085701
AC016383.1	-0.92368	0.085701
AC079915.1	-0.92368	0.085701
AP002472.1	-0.92368	0.085701
MIR5002	-0.92368	0.085701
MIR3157	-0.92368	0.085701
NA	-0.92368	0.085701
NA	-0.92368	0.085701
AP001381.1	-0.92368	0.085701
MIR4530	-0.92368	0.085701
AP001025.1	-0.92368	0.085701
AC103808.5	-0.92368	0.085701
MIR4742	-0.92368	0.085701
MIR4642	-0.92368	0.085701
NA	-0.92368	0.085701
AC233702.8	-0.92368	0.085701
AC005304.3	-0.92368	0.085701
AL031428.2	-0.92368	0.085701
AC068254.1	-0.92368	0.085701
TCF4-AS1	-0.92368	0.085701
AC005757.1	-0.92368	0.085701
LINC02080	-0.92368	0.085701
PCAT19	-0.92368	0.085701
SCAT1	-0.92368	0.085701
AC092296.3	-0.92368	0.085701
AC067968.2	-0.92368	0.085701
AC011447.2	-0.92368	0.085701
AC008567.2	-0.92368	0.085701
AC011444.3	-0.92368	0.085701
LINC01764	-0.92368	0.085701
AC021504.1	-0.92368	0.085701
ATP5MGP6	-0.92368	0.085701
AC006130.2	-0.92368	0.085701
AC011509.1	-0.92368	0.085701
NFE2L3P1	-0.92368	0.085701

AP001029.3	-0.92368	0.085701
AC090699.1	-0.92368	0.085701
AC022517.1	-0.92368	0.085701
AC008742.1	-0.92368	0.085701
LINC01482	-0.92368	0.085701
NA	-0.92368	0.085701
AL121989.1	-0.92368	0.085701
AC087645.2	-0.92368	0.085701
NA	-0.92368	0.085701
NA	-0.92368	0.085701
NA	-0.92368	0.085701
NA	-0.92368	0.085701
AC093503.2	-0.92368	0.085701
AC011468.3	-0.92368	0.085701
AP002884.4	-0.92368	0.085701
NA	-0.92368	0.085701
AC136469.1	-0.92368	0.085701
KRT18P40	-0.92368	0.085701
NA	-0.92368	0.085701
BRI3BPP1	-0.92368	0.085701
AC092329.2	-0.92368	0.085701
NA	-0.92368	0.085701
NA	-0.92368	0.085701
AC011492.1	-0.92368	0.085701
BICRA-AS1	-0.92368	0.085701
NA	-0.92368	0.085701
AP000962.1	-0.92368	0.085701
NA	-0.92368	0.085701
AC068491.3	-0.92368	0.085701
NA	-0.92368	0.085701
COX6CP16	-0.92368	0.085701
AL390123.1	-0.92368	0.085701
AL451085.1	-0.92368	0.085701
AC007566.2	-0.92368	0.085701
AL355297.2	-0.92368	0.085701
AL450336.1	-0.92368	0.085701
AL162713.1	-0.92368	0.085701
AC006023.1	-0.92368	0.085701
PPIAP52	-0.92368	0.085701
PRELID3BP8	-0.92368	0.085701
AC018737.1	-0.92368	0.085701
AC073413.2	-0.92368	0.085701
AC114878.2	-0.92368	0.085701
AL512430.4	-0.92368	0.085701
AC087763.1	-0.92368	0.085701
AL157899.1	-0.92368	0.085701
AC139700.1	-0.92368	0.085701
AC020910.3	-0.92368	0.085701
AC009314.1	-0.92368	0.085701
PPIAP75	-0.92368	0.085701

VDAC3P1	-0.92368	0.085701
BRDTP1	-0.92368	0.085701
AC011458.2	-0.92368	0.085701
AL035417.2	-0.92368	0.085701
PRR13P3	-0.92368	0.085701
AL031281.1	-0.92368	0.085701
AC013244.2	-0.92368	0.085701
LINC02333	-0.92368	0.085701
AC104763.2	-0.92368	0.085701
AC103858.1	-0.92368	0.085701
Z98200.1	-0.92368	0.085701
AL121992.2	-0.92368	0.085701
AC139792.1	-0.92368	0.085701
NA	-0.92368	0.085701
AC012467.1	-0.92368	0.085701
AL512413.1	-0.92368	0.085701
AC091614.1	-0.92368	0.085701
AC100812.1	-0.92368	0.085701
AC009686.2	-0.92368	0.085701
AL020996.3	-0.92368	0.085701
AC012511.1	-0.92368	0.085701
U62317.1	-0.92368	0.085701
AC011899.3	-0.92368	0.085701
AC113189.4	-0.92368	0.085701
NA	-0.92368	0.085701
NA	-0.92368	0.085701
NA	-0.92368	0.085701
AP000322.2	-0.92368	0.085701
AL365434.2	-0.92368	0.085701
PACERR	-0.92368	0.085701
LINC02091	-0.92368	0.085701
AL049839.2	-0.92368	0.085701
AC097381.3	-0.92368	0.085701
U62317.4	-0.92368	0.085701
AL008718.2	-0.92368	0.085701
CYP2F2P	0.141124	0.085697
MIMT1	0.291499	0.085653
INO80C	0.056358	0.08565
DPCD	0.043839	0.085644
ADAMTS4	-0.03356	0.085624
LNCTAM34A	-0.2276	0.08561
SPECC1L	-0.02757	0.085609
POLB	0.04115	0.085574
AC012213.4	-0.16518	0.085562
RTL6	-0.0337	0.085541
RF00410	-0.37011	0.085515
DNAJC19	-0.03378	0.085413
UBE2J2	0.037166	0.08541
AC087392.4	-0.24314	0.085401
AC022400.7	-0.08177	0.085393

AL592546.1	-0.23078	0.085387
HRCT1	0.475394	0.08538
NA	0.603037	0.085365
NA	0.072195	0.085365
AC093525.6	-0.12718	0.085347
AC036108.3	-0.2097	0.085346
GEMIN6	-0.03262	0.085333
MYL6	0.022446	0.085324
PHF5A	0.034456	0.08532
AC090578.1	-0.16834	0.085284
SLC4A1AP	0.028708	0.085278
CNN2	0.082474	0.085274
ZNF222	-0.06181	0.085262
MPHOSPH9	-0.02995	0.08525
PHACTR2-AS1	0.546415	0.085211
PRSS27	0.058448	0.085209
BANP	0.03642	0.085206
AC004951.4	0.307843	0.085142
ZNF433	-0.11898	0.085126
CHRAC1	0.037831	0.085123
AC016644.1	-0.10661	0.085112
FAM226B	0.442137	0.085073
GPR156	0.191122	0.085071
BMP2	-0.40193	0.08507
COP1	-0.03152	0.085058
LINC01422	0.589139	0.08501
FAM242C	-0.22014	0.084992
CRYBB3	0.424424	0.084989
AC073210.1	-0.19755	0.084984
NCL	-0.02136	0.084982
AL807752.1	0.380946	0.084942
FABP5	0.037397	0.084936
AKIP1	0.038916	0.084903
TRPV2	-0.25763	0.084886
BNIPL	0.151881	0.084874
AC114956.2	0.216741	0.084869
ATXN2L	0.032861	0.084868
PYGB	-0.03045	0.084861
AC006141.1	0.282121	0.084852
AC092364.2	0.322516	0.084849
MRPL14	-0.03015	0.084814
BVES	0.036071	0.084814
AC063965.1	-0.28041	0.084812
FTH1P11	0.299682	0.084792
AC022784.5	0.22496	0.084762
PAICSP4	-0.41755	0.084756
AL450384.2	0.080033	0.084721
DSCC1	-0.03939	0.084708
NA	0.235084	0.084662
NA	-0.27933	0.084659

AC093833.1	0.586513	0.084627
NNT-AS1	-0.04133	0.084626
PFKFB4	-0.05216	0.084604
KLC4	0.040173	0.084591
CCDC54	0.848044	0.084579
SOSTDC1	0.848044	0.084579
PRR18	0.848044	0.084579
TMEM89	0.848044	0.084579
RPL7AP60	0.848044	0.084579
AL157884.3	0.848044	0.084579
AC084033.1	0.848044	0.084579
AL138690.1	0.848044	0.084579
AC087749.1	0.848044	0.084579
AC010487.2	0.848044	0.084579
BLNK	-0.70292	0.084538
AC008758.2	-0.70292	0.084538
HNRNPLP2	0.180944	0.0845
WNT2B	0.06312	0.084451
NOSTRIN	-0.13465	0.084362
DUSP8P5	-0.09235	0.084347
AGPAT5	-0.03058	0.084346
GNB1L	-0.05762	0.084325
RNU1-82P	0.448892	0.084324
IFT52	0.032547	0.084308
CIAO3	0.030226	0.084281
RNU6-611P	0.347473	0.084249
AL590714.1	-0.04196	0.084211
NA	0.128204	0.084199
LSM10	-0.03725	0.084196
AC073529.1	0.12329	0.084169
STK36	-0.02799	0.084141
LBHD1	-0.02819	0.084125
SHE	0.364381	0.08412
CCDC88B	0.105604	0.084112
NA	0.229547	0.084036
NA	0.111487	0.084026
AL021707.6	-0.15211	0.084015
KLHL18	-0.03299	0.084014
ATG14	0.034126	0.084003
PABPC1L	-0.03202	0.083991
AC079385.3	-0.36415	0.083938
CHADL	0.138827	0.083821
MIR4512	0.424305	0.083803
NA	-0.2944	0.083798
BLZF1	0.035902	0.083785
MBLAC1	-0.08176	0.083776
ZNF655	-0.02325	0.08375
DCDC1	-0.44009	0.083736
MIR4633	-0.32503	0.083734
CCDC81	0.150795	0.083731

CCDC144A	0.54554	0.083699
MZT1P2	0.54554	0.083699
FAM160A2	-0.04007	0.083687
NA	-0.29824	0.083679
HNRNPA1P14	-0.69704	0.083676
AF213884.1	-0.69704	0.083676
AL353768.1	-0.47229	0.083654
AL391069.2	0.091761	0.083649
ZBTB7B	0.033363	0.083642
FAM174A	0.036236	0.083625
AC125232.1	0.074899	0.083623
CNN1	0.095937	0.083602
AC069542.1	-0.10448	0.083596
SS18L1	0.02943	0.083594
AC009404.1	-0.06712	0.083589
C14orf132	-0.0235	0.083586
RTN2	-0.04076	0.08357
RNU6-327P	-0.40367	0.083566
AC006206.2	0.140432	0.083539
TTI2	-0.03284	0.083533
SCNN1D	0.132734	0.08352
TTLL1	-0.05745	0.083492
B3GNT5	-0.03762	0.083481
AP001318.1	-0.11584	0.08347
AC005523.2	-0.02965	0.083458
CRBN	-0.03227	0.083436
AC079465.1	-0.34608	0.083435
VEZF1	-0.02289	0.083425
ITK	0.498087	0.083414
AC005670.3	0.043256	0.083411
JPT1	0.025219	0.083399
ABHD14B	-0.0368	0.083386
CTDSP1	-0.03232	0.083373
NECTIN3	-0.04891	0.083372
AC008403.3	0.198131	0.083359
AC010336.3	-0.12982	0.083346
AF279873.1	0.474281	0.083319
GALR2	0.538266	0.083313
AC092471.1	0.335545	0.08331
AC010326.4	-0.06237	0.0833
LYL1	-0.06737	0.083244
SNORA55	0.279029	0.083241
AP000873.4	0.16302	0.083238
LINC01357	0.135189	0.08322
CD27-AS1	-0.05345	0.083211
ZNF271P	0.031692	0.083206
NA	0.188984	0.083173
LIN28A	0.847582	0.083172
ATP1A4	0.847582	0.083172
BCRP4	0.847582	0.083172

NA	0.847582	0.083172
RPL17P43	0.847582	0.083172
PCDHA13	0.847582	0.083172
AC093620.1	0.847582	0.083172
HSPA8P19	0.847582	0.083172
YWHAQP7	0.847582	0.083172
RNU4-89P	0.847582	0.083172
RF00019	0.421699	0.083156
CNTD2	0.26685	0.083156
SGPP1	-0.04922	0.083134
PCNPP1	-0.23885	0.083108
PRMT2	-0.02901	0.083099
LDHAL6B	0.847553	0.083084
RN7SKP163	0.847553	0.083084
NA	0.847553	0.083084
RNU6-301P	0.847553	0.083084
AC020688.1	0.847553	0.083084
AC010342.1	0.847553	0.083084
AP004245.1	0.847553	0.083084
CTXND1	0.847553	0.083084
SLC23A2	0.035899	0.083084
NUTM2B	0.168688	0.083046
ACBD5	-0.03287	0.083024
MAP10	-0.19686	0.083014
AC021683.3	-0.35912	0.082977
USP20	-0.03675	0.082954
NA	-0.07827	0.082953
CYP2D8P	-0.54541	0.082946
TNK2	-0.03707	0.082942
VPS72	0.027018	0.082934
PTK6	-0.11051	0.082879
PARD3	0.029129	0.082863
PCMTD1	0.033937	0.08286
AK4	-0.0816	0.082855
NPAT	-0.04554	0.082813
MAGEF1	0.02424	0.082807
AL358933.1	-0.31622	0.082787
CLVS1	0.060838	0.082774
NA	0.188404	0.08276
ABCA10	-0.3623	0.082719
ZNRD1ASP	0.037097	0.082678
TYW5	0.044239	0.082666
CSRNP2	0.031838	0.082653
SNORD125	-0.20059	0.082652
SAP30L-AS1	-0.18109	0.082619
NA	-0.22944	0.082615
AL589765.6	0.103255	0.082612
LRRTM4	-0.31821	0.082583
ZNF793-AS1	0.126941	0.082582
WDR35	0.025119	0.082563

AC009005.1	0.10992	0.082535
GRM4	-0.37881	0.082528
MYO18A	-0.04171	0.082523
DLL3	0.100727	0.082485
AC018463.1	0.580562	0.082474
ZDHHC1	-0.05562	0.082472
STEAP1B	-0.10558	0.082471
SHC3	-0.04831	0.082463
MEPCE	-0.02452	0.082459
USP10	0.024378	0.082409
CEP72	-0.05143	0.082403
IQCB1	-0.03346	0.082349
CISD2	-0.03036	0.082341
KAT5	-0.02429	0.082333
GLRX	0.041397	0.082318
TPRN	-0.0414	0.082315
ACAD9	-0.03257	0.082307
CEP78	0.028509	0.082299
LARP7	0.030629	0.082298
KLHDC3	0.022126	0.082288
AL390066.1	-0.21036	0.082247
IRGQ	-0.02974	0.082228
NA	0.650385	0.082218
AC100793.2	-0.18927	0.082151
NSDHL	-0.03585	0.082144
SLC7A6	-0.028	0.082143
NUP58	0.027389	0.082131
GTPBP1	0.038315	0.082118
SELENOS	0.029363	0.082113
AC133552.4	0.25624	0.082104
EPHA6	-0.14761	0.082074
ZNF663P	-0.12049	0.082071
MID1IP1	0.03132	0.082055
FAM163B	-0.09191	0.081975
MIR590	-0.20361	0.081954
BRAF	-0.02505	0.081911
FGGY	-0.08398	0.081898
AF241728.1	-0.29049	0.081888
RNU6-312P	-0.38649	0.081862
TG	-0.28558	0.08183
ESCO2	0.05064	0.081826
XIST	-0.02448	0.081822
RF00322	-0.231	0.081821
LRRC47	-0.02992	0.081803
AP001267.2	-0.62807	0.081798
NA	0.52835	0.081759
AL355607.2	0.52835	0.081759
CCT4	0.025049	0.081715
AC114803.1	-0.08381	0.081715
ARSE	-0.80995	0.081706

HMGB3P6	-0.80995	0.081706
AC092839.2	-0.80995	0.081706
MTATP6P26	-0.80995	0.081706
AC025518.1	-0.80995	0.081706
LINC02270	-0.80995	0.081706
LINC02384	-0.80995	0.081706
RPL21P5	-0.80995	0.081706
AC013391.2	-0.80995	0.081706
AL133383.1	-0.80995	0.081706
AC008906.2	-0.80995	0.081706
CFDP1	0.030018	0.081657
AC006145.1	0.302232	0.081623
POLD3	0.038779	0.081612
AC137932.3	0.062157	0.081598
AL136980.1	0.118671	0.081559
FNDC8	0.090767	0.08155
RNU6-118P	-0.63851	0.081545
ANKRD10	0.028454	0.081539
MED1	-0.02422	0.081528
NARS	0.021696	0.081517
CPEB4	0.032667	0.081446
GRAMD2B	0.082501	0.081444
GAS2L3	-0.03839	0.081433
NA	-0.0695	0.081404
SERPINB2	-0.294	0.081389
NAALADL2	0.197505	0.081383
NUDT2	-0.0483	0.081317
IFI27	0.143287	0.081292
HIBCH	-0.0417	0.08128
AC120049.1	-0.16309	0.081275
NEXMIF	0.070263	0.081252
IWS1	0.030964	0.081242
LPL	-0.53565	0.081227
HIF1A	0.020638	0.081159
TRPM4	-0.06857	0.081109
RASIP1	-0.09064	0.081099
AC099513.1	0.497124	0.081077
SLC50A1	-0.03596	0.08106
ADGRV1	0.038057	0.081056
LCN1	-0.19905	0.081055
MPV17	-0.02367	0.081032
AL132656.2	-0.18192	0.08103
SMURF2P1	0.10423	0.081022
PACSIN3	0.045736	0.081013
VANGL2	-0.0256	0.080997
RPL34P6	0.342872	0.080995
STX2	-0.03413	0.080987
NA	-0.58116	0.080963
NA	-0.58116	0.080963
AC021097.1	-0.05768	0.080926

AC007560.1	-0.62232	0.080905
TM4SF1	-0.07821	0.080903
GYG1	0.049541	0.080896
AL135999.1	0.037282	0.080888
FCF1P2	-0.09197	0.080887
AC087639.1	0.476633	0.080878
AC004584.1	-0.33705	0.080852
ABCD3	-0.03048	0.080831
RSF1-IT1	-0.50744	0.080746
NAP1L3	0.084799	0.080727
AC105402.3	0.395028	0.080718
KDM4B	0.037408	0.080704
UBE2D3P1	0.355187	0.080682
NUDT14	-0.06994	0.080673
AC008581.1	-0.06398	0.080668
MTPN	0.023099	0.080664
AC091167.3	0.476011	0.080661
AC009955.3	0.089358	0.080566
AGPS	0.026511	0.080527
CAMK2B	-0.12188	0.080526
MRPL1	-0.03668	0.080524
USP34	0.02341	0.080506
CASC1	0.284113	0.080447
BMT2	0.033037	0.080417
AC018845.3	-0.29031	0.080412
DBT	-0.03444	0.08041
DKC1	-0.02176	0.080387
FXN	-0.06307	0.080376
FASTK	0.02408	0.080353
AC016705.2	-0.12442	0.080347
AC007786.1	0.519426	0.080345
RBM17	0.025095	0.080329
AL583810.2	-0.36749	0.080325
AC006042.2	0.125422	0.080325
TNK2-AS1	-0.1037	0.080272
FAM114A2	0.040886	0.080255
ELOC	-0.02687	0.080253
SRSF1	0.020768	0.080251
FBXW12	0.244548	0.080222
SEC22C	-0.02805	0.080221
PPWD1	-0.03397	0.080211
ZNF485	0.048029	0.080205
AC079148.1	-0.29567	0.080197
ADGRL3	-0.09169	0.080147
SPIN2A	-0.32849	0.080131
LINC00886	-0.17015	0.080116
POLR3A	-0.02361	0.080114
MAN1A2	-0.02368	0.080094
TLE4	-0.0265	0.08009
AC018868.2	-0.50003	0.080072

AC068790.6	-0.50003	0.080072
IGFBP2	-0.02328	0.080063
HCCS	-0.03379	0.080063
NA	-0.31661	0.080056
SYNE1-AS1	0.36119	0.080048
SHLD3	0.081951	0.080048
NA	0.326293	0.080007
RNA5SP383	0.381394	0.079997
PSMD9	-0.07036	0.079958
LINC01770	0.327027	0.079951
NDST2	-0.22703	0.079938
CCN2	0.117874	0.079904
TIGD1	0.042842	0.079897
CTBP1	0.027172	0.07989
TIMM13	0.025365	0.079883
RF00019	-0.1782	0.079869
MUSK	0.493095	0.079863
STOM	0.093556	0.079855
PDCD6IPP2	0.059953	0.079841
LINC00933	0.244701	0.079829
HMGB3P7	-0.80958	0.079825
AC005884.2	-0.80958	0.079825
AC064871.1	-0.23172	0.079816
TSTA3	0.030109	0.079809
PEX19	0.027797	0.079804
PRR36	-0.0693	0.079791
AC027309.2	0.201274	0.079771
MTHFD2L	0.04428	0.079768
IFNLR1	0.277768	0.079759
AL513327.3	0.192754	0.07975
TLCD2	-0.18823	0.079735
AC104986.1	-0.20455	0.079629
FAM221A	0.034415	0.079627
RPL21P93	0.469956	0.079617
AC093801.1	0.469956	0.079617
STX10	0.025365	0.07961
TAP2	-0.0659	0.079603
TIMM22	-0.03658	0.079594
YWHAEP1	-0.43311	0.079591
CDX1	0.206556	0.079561
ARHGAP31	0.030893	0.079557
VEGFA	0.030303	0.079518
RPL5P11	0.440456	0.079511
BMS1P17	-0.08211	0.079504
AC073073.1	0.160346	0.079495
PSMB1	0.023921	0.079476
AC069277.1	0.269553	0.07947
B3GALT5-AS1	0.168593	0.079456
EFCAB2	-0.03643	0.079455
CSRNP3	0.029188	0.079449

UBE2V2	-0.03089	0.079443
AL583843.1	-0.31368	0.079428
AL513165.1	0.070382	0.07937
HNRNPA3P10	0.203329	0.079365
CFAP161	-0.33549	0.079359
LINC00528	0.548039	0.079353
OR2B2	0.380277	0.079339
HCG20	0.380277	0.079339
MEAF6	0.026881	0.079332
DCPS	-0.04733	0.079332
C3orf49	0.359631	0.079324
GNB2	0.024636	0.079324
MYO9B	-0.0295	0.079321
LARP4	0.02353	0.079302
RPSAP9	-0.2439	0.079302
HSPB11	0.039828	0.079256
IGSF9	0.041518	0.079221
TVP23B	-0.03039	0.079202
DHRS4-AS1	0.038683	0.079195
NA	-0.16426	0.07916
NA	-0.26366	0.079124
SCRN1	-0.02089	0.079095
THTPA	-0.06095	0.079092
PHLDB3	0.06982	0.079059
MRPS5	0.029292	0.07901
NDEL1	-0.0267	0.078996
LIPT1	-0.06769	0.078982
Z97989.1	-0.07794	0.078975
RMND1	0.034102	0.078939
NA	0.098794	0.078916
GRM2	0.086013	0.078904
Z92544.1	0.33151	0.078897
C5orf15	-0.03096	0.078895
ZNF28	0.05224	0.078886
ATP13A2	-0.03802	0.07888
MUC12	0.282057	0.078878
ATP6V1FNB	-0.1292	0.078875
PARN	-0.03346	0.078874
HR	-0.04463	0.078864
AC008264.2	0.033516	0.078844
AGBL4	0.137253	0.078823
AC087276.2	-0.04378	0.078771
PCYT2	0.028546	0.078768
PPCDC	-0.05522	0.07874
AL049543.1	-0.15143	0.078739
ZC3H12D	0.282274	0.078719
ZNF267	0.052197	0.078674
ZNF691	-0.03892	0.078615
RBM27	-0.02893	0.078591
TULP3	0.03069	0.07859

AC243772.2	-0.17367	0.078587
STAT5A	-0.08288	0.078585
LSG1	0.023033	0.078582
AC025809.1	0.411833	0.078564
CALCR	0.400004	0.078558
SHANK2-AS1	0.468284	0.078543
MAP3K7CL	-0.20533	0.078507
AC026954.3	0.116052	0.07849
AC118344.1	0.111502	0.07849
FMO1	-0.80931	0.078479
MSR1	-0.80931	0.078479
LYZ	-0.80931	0.078479
CHIT1	-0.80931	0.078479
LINC01600	-0.80931	0.078479
RNU5B-2P	-0.80931	0.078479
AL606491.1	-0.80931	0.078479
NECAP1P2	-0.80931	0.078479
RPS2P35	-0.80931	0.078479
AC110998.1	-0.80931	0.078479
AC113133.1	-0.80931	0.078479
LINC01613	-0.80931	0.078479
AC007192.1	-0.80931	0.078479
UNGP3	-0.80931	0.078479
AC144521.1	-0.80931	0.078479
MANEAL	0.029219	0.078462
ST6GALNAC6	0.032193	0.078445
CABP4	0.606715	0.078432
API5P1	0.606715	0.078432
TTYH2	-0.06506	0.07842
POF1B	-0.43006	0.07839
NA	0.09829	0.078372
MBD6	0.040753	0.078344
NA	-0.11937	0.078337
AC124283.1	-0.0323	0.078329
ZNF354C	0.032085	0.078295
SKIL	-0.02686	0.078289
SLC35E3	0.034626	0.078283
YY2	-0.08002	0.078281
NUAK2	0.073041	0.078275
AL033527.3	-0.32416	0.078254
AC022336.1	-0.45968	0.078223
FALEC	0.322939	0.078207
PTPRM	0.04203	0.078198
NKIRAS2	-0.0253	0.078155
AL121787.1	-0.15264	0.078132
NA	-0.10757	0.078123
RAB6C	-0.09733	0.078101
RN7SL219P	0.226014	0.078092
B3GALNT1	0.041458	0.078091
TALDO1	0.024312	0.078086

CLN5	-0.038	0.078085
AC084361.1	0.162554	0.078067
AC089999.1	-0.08301	0.078001
YTHDC2	0.029769	0.077989
CPLX2	0.031089	0.077989
FAM214A	-0.03197	0.077983
GSTCD	-0.03745	0.077977
RNF208	-0.06989	0.077973
JOSD2	-0.03765	0.07796
SRSF5	0.023019	0.077873
AC080112.1	-0.08333	0.077796
AC000078.1	-0.31826	0.077794
PYGO1	-0.0355	0.077779
LCA5	-0.0466	0.077769
PTDSS1	-0.02301	0.077743
ZNF582-AS1	-0.12272	0.077723
MIR4519	-0.63031	0.077689
TNFRSF25	0.086169	0.077664
AC091053.1	0.040294	0.077618
XAB2	0.030401	0.077595
ARHGEF26-AS1	0.513608	0.077593
AC024270.2	0.513608	0.077593
CXorf38	0.049088	0.077567
ZNF546	-0.05186	0.077501
LINC01117	0.086958	0.077501
BCLAF3	0.051573	0.0775
ANGPTL4	0.114282	0.077498
POLR3C	-0.028	0.077472
ARHGAP17	0.031819	0.077428
RN7SL689P	0.453371	0.07741
IMPDH1P6	0.505114	0.077396
NA	-0.56089	0.077377
AC112225.1	-0.51225	0.077366
AC068014.1	-0.51225	0.077366
NPR1	-0.22972	0.077303
RNU6-884P	-0.58421	0.077257
AL136115.2	-0.58421	0.077257
CEP68	0.028829	0.077255
AURKB	-0.03123	0.077232
ST13P11	-0.65214	0.077188
AL513323.1	-0.65214	0.077188
RN7SKP287	-0.65214	0.077188
AC005379.1	-0.65214	0.077188
AC005323.2	-0.65214	0.077188
RNU7-49P	0.510807	0.077164
GALK2	-0.04719	0.077149
AGK	0.030456	0.077144
CICP22	0.199519	0.077138
CARD19	-0.03227	0.077115
PLD1	-0.08831	0.077112

AL450124.1	-0.21772	0.077096
AC090114.2	-0.04276	0.077094
BLVRB	0.073506	0.077049
SNED1	-0.03915	0.077003
TMEM106B	-0.02705	0.076981
GPATCH3	0.04468	0.076906
AL392046.1	0.124177	0.076861
EIF2D	0.027838	0.076837
Z97200.1	-0.30127	0.076817
ETF1	-0.02122	0.076769
NA	-0.64911	0.076757
RN7SL559P	-0.64911	0.076757
POLR3H	-0.02834	0.076741
AC013643.2	0.074919	0.076716
MIR3934	-0.14455	0.076657
GBE1	0.033608	0.076647
DANCR	-0.02295	0.076608
CCN6	0.133769	0.07657
NA	-0.02592	0.076566
ATP5F1D	0.026447	0.076559
AC023946.1	-0.18419	0.076518
SLCO4A1-AS1	-0.12599	0.076491
RPL3P2	-0.16432	0.076464
RF00019	0.499226	0.076452
ARF4	-0.02236	0.076447
GPATCH8	-0.02493	0.076437
CHIC1	0.05485	0.076428
AK3P3	0.164634	0.076422
LINC02637	-0.18615	0.076417
MIR3936	-0.31446	0.076415
FUT9	0.048163	0.07641
NEMF	-0.02797	0.076405
AL589993.1	0.364718	0.076348
CKS1BP3	-0.35787	0.076323
ZFP92	0.262207	0.076311
RNU6-658P	-0.40683	0.076263
PRICKLE1	0.032116	0.076245
AC244394.2	0.131002	0.076243
TRIP12	-0.01989	0.076228
FARSB	0.028141	0.07621
AC008507.2	-0.35732	0.07621
GOLGA3	-0.02702	0.076206
PIK3R3	-0.02697	0.076175
GPR183	-0.38698	0.076174
LMCD1	-0.03906	0.076134
NA	-0.0387	0.076106
RPSAP12	-0.26274	0.076074
VMA21	0.02903	0.076057
NA	0.426184	0.076052
C2CD4D-AS1	-0.40382	0.076015

OSGIN2	-0.03531	0.075962
RPL36AP43	-0.21016	0.075921
NA	-0.13816	0.075906
AC104066.1	-0.36743	0.075903
NUSAP1	-0.02681	0.075885
AGMO	0.270563	0.075864
AC145207.3	-0.02081	0.075788
NA	0.100846	0.075763
FAM219A	0.035953	0.075742
AC019129.2	0.630046	0.075739
SLC25A4	0.02743	0.075725
HMGN2P6	0.352753	0.075713
AC011498.2	0.123642	0.07567
AC064875.1	-0.23743	0.075648
HAUS4	-0.04257	0.075633
SNAP23	0.026158	0.07563
IRF2BP2	0.02869	0.0756
PPP1R26P1	-0.30429	0.075594
RLN2	0.217663	0.075585
NXPH1	0.132945	0.075571
AC009019.1	-0.07131	0.075535
AC098934.1	-0.044	0.075531
SH2D2A	0.420564	0.075526
AL354892.3	0.170389	0.075521
RPL29P19	0.096652	0.075484
TRAP1	-0.02399	0.075477
AL049712.1	0.481823	0.075471
BTK	-0.8133	0.075447
MYOM2	-0.8133	0.075447
PLEKHG1	-0.8133	0.075447
SLC39A2	-0.8133	0.075447
PLA2G1B	-0.8133	0.075447
TMEM78	-0.8133	0.075447
C3orf22	-0.8133	0.075447
RXFP3	-0.8133	0.075447
RF00019	-0.8133	0.075447
NA	-0.8133	0.075447
RF00019	-0.8133	0.075447
AL022323.1	-0.8133	0.075447
RF00019	-0.8133	0.075447
RF00019	-0.8133	0.075447
SNORA22C	-0.8133	0.075447
RF00019	-0.8133	0.075447
MT-TM	-0.8133	0.075447
HNRNPA1P34	-0.8133	0.075447
MINDY4B	-0.8133	0.075447
NA	-0.8133	0.075447
RN7SKP112	-0.8133	0.075447
BNIP3P42	-0.8133	0.075447
AC126124.1	-0.8133	0.075447

NA	-0.8133	0.075447
AL022724.1	-0.8133	0.075447
UBE2L5	-0.8133	0.075447
LINC01192	-0.8133	0.075447
MARK2P6	-0.8133	0.075447
NA	-0.8133	0.075447
AC026410.3	-0.8133	0.075447
CASC11	-0.8133	0.075447
ABT1P1	-0.8133	0.075447
FCF1P8	-0.8133	0.075447
RF00019	-0.8133	0.075447
NA	-0.8133	0.075447
NA	-0.8133	0.075447
AC104393.1	-0.8133	0.075447
AC084816.1	-0.8133	0.075447
CR383656.1	-0.8133	0.075447
AL139317.1	-0.8133	0.075447
ITPK1-AS1	-0.8133	0.075447
LINC01579	-0.8133	0.075447
NA	-0.8133	0.075447
RN7SL510P	-0.8133	0.075447
NA	-0.8133	0.075447
AC010641.1	-0.8133	0.075447
NA	-0.8133	0.075447
NA	-0.8133	0.075447
AC008739.2	-0.8133	0.075447
AL049840.6	-0.8133	0.075447
AC025171.5	-0.8133	0.075447
PCDH9-AS1	0.20455	0.075426
LINC00315	0.604822	0.075423
AL023284.2	0.604822	0.075423
UHMK1	-0.02392	0.075418
MIR1915	0.410992	0.075388
NA	-0.05644	0.075387
UBE2CP2	-0.35649	0.075366
DUXAP8	0.027211	0.075349
PTGFRN	-0.02521	0.075346
TMEM184C	0.022557	0.075336
MPC2	0.026705	0.075328
SLC39A8	-0.03566	0.075313
ANKS3	0.034971	0.07531
BMP1	-0.03104	0.075289
STAT2	-0.02857	0.075238
SMC6	-0.02979	0.075225
BMP8A	0.087663	0.075223
ANAPC16	-0.02457	0.075215
TMEM139	-0.25434	0.075206
RGS19	-0.03742	0.075164
STKLD1	-0.06778	0.075162
RSF1-IT2	-0.43162	0.075162

C11orf65	0.080161	0.07516
CBWD1	0.039572	0.075154
ZNF461	0.042956	0.075127
WDR11	0.023096	0.075122
AC139100.1	0.116233	0.075114
PREX1	0.050025	0.075093
AC026691.1	0.242941	0.075092
LINC02062	-0.22221	0.075087
AC012184.3	-0.05012	0.075086
CCDC40	0.030355	0.075086
IGSF21	-0.53691	0.075078
RFPL3	-0.53691	0.075078
AC105036.3	0.179401	0.075061
CHMP4A	-0.1154	0.07505
AC091132.3	0.321132	0.075047
NA	0.513197	0.075035
MIS18A-AS1	0.191307	0.075031
SLC9A6	0.034382	0.075026
TRPV4	-0.09608	0.075025
REC8	-0.02397	0.075003
PENK	-0.37085	0.074996
POLD2	-0.02311	0.074926
MC4R	0.131977	0.07487
DDAH2	-0.0246	0.074867
CHRNB1	0.037261	0.074856
LINC00920	-0.12248	0.074852
PTGR1	-0.06205	0.074851
KDM4A-AS1	-0.03773	0.074846
NSUN3	-0.0443	0.07483
NA	-0.22638	0.074822
KIF3B	-0.0229	0.074808
RAI1	0.029104	0.074796
AC010422.6	-0.1453	0.074795
RNU6-88P	0.412579	0.074791
RAD51C	-0.03287	0.074765
PABPC1P3	0.351954	0.074761
NA	0.027631	0.074761
KLHL14	0.116145	0.07476
POP7	-0.02805	0.074737
MIR563	0.467006	0.074729
CXCL16	-0.11072	0.074654
PFKFB2	-0.03654	0.074645
SERBP1P1	0.156888	0.074627
CFAP298	-0.03881	0.074605
AC125807.1	0.157215	0.074589
MIP	0.086585	0.07458
CAMK2D	0.025138	0.074579
FRGCA	0.389684	0.074571
PDIA3P2	-0.24144	0.074541
RNU6-130P	-0.38048	0.07454

AP000926.1	-0.65784	0.074507
AC079035.1	-0.65784	0.074507
AL592148.2	-0.65784	0.074507
LINC02664	-0.65784	0.074507
SIRPB1	-0.80396	0.074497
AC105760.1	-0.80396	0.074497
HBZ	-0.80396	0.074497
RNF180	-0.80396	0.074497
RNASE10	-0.80396	0.074497
GJB5	-0.80396	0.074497
AL031577.1	-0.80396	0.074497
UBE2Q2P12	-0.80396	0.074497
RN7SKP184	-0.80396	0.074497
RNU6-190P	-0.80396	0.074497
RNU6-1263P	-0.80396	0.074497
RF00019	-0.80396	0.074497
NA	-0.80396	0.074497
SNORA79	-0.80396	0.074497
RNU6-920P	-0.80396	0.074497
RNU4-78P	-0.80396	0.074497
AC073136.2	-0.80396	0.074497
AL353572.1	-0.80396	0.074497
AC016700.1	-0.80396	0.074497
CKS1BP2	-0.80396	0.074497
NA	-0.80396	0.074497
AC092155.2	-0.80396	0.074497
NA	-0.80396	0.074497
ARMC2-AS1	-0.80396	0.074497
AC007690.1	-0.80396	0.074497
KRT8P15	-0.80396	0.074497
ESRRAP2	-0.80396	0.074497
AC078991.1	-0.80396	0.074497
AC079760.2	-0.80396	0.074497
AC090543.2	-0.80396	0.074497
AC105250.1	-0.80396	0.074497
RF00019	-0.80396	0.074497
NA	-0.80396	0.074497
AC097173.2	-0.80396	0.074497
AC103681.2	-0.80396	0.074497
LINC01290	-0.80396	0.074497
AC007496.2	-0.80396	0.074497
AC005592.1	-0.80396	0.074497
AC011476.2	-0.80396	0.074497
AC004223.2	-0.80396	0.074497
AP002414.4	-0.80396	0.074497
NA	-0.80396	0.074497
AC011443.1	-0.80396	0.074497
AC011445.2	-0.80396	0.074497
NA	-0.80396	0.074497
AC103724.4	-0.80396	0.074497

AC011816.2	-0.80396	0.074497
AL133215.3	-0.80396	0.074497
AC245884.9	-0.46146	0.074495
HNRNPKP1	-0.60905	0.074471
AC003002.3	-0.60905	0.074471
NA	0.065716	0.074421
EHMT1	0.027008	0.074396
CLINT1	-0.02038	0.074347
TIGD7	-0.05173	0.07434
GSTM5P1	-0.60335	0.074332
FAM160B2	-0.0398	0.074325
PCDHGA3	-0.31181	0.074322
LRR1	0.043947	0.074254
ZNF480	0.03956	0.07423
CLP1	-0.03314	0.074219
PCNX4	0.021082	0.074215
AL159987.1	-0.57869	0.074209
AL139156.2	-0.57869	0.074209
AC103681.1	-0.57869	0.074209
AC105046.1	-0.57869	0.074209
SNHG22	-0.57869	0.074209
RN7SL381P	0.126544	0.074154
MT-TD	-0.49454	0.074131
NA	-0.49454	0.074131
NA	-0.18282	0.074131
SUZ12	0.027051	0.074099
AL136116.3	0.118073	0.074099
RPS4XP11	0.314788	0.074097
AL157871.6	-0.09161	0.074079
AC104561.3	0.334497	0.074054
MIR3132	-0.60617	0.074054
TIMM21	0.0302	0.074053
HNRNPA3	0.021564	0.074037
AC009309.1	0.134974	0.074034
PARVB	0.030647	0.074022
AC109454.2	-0.19215	0.074019
NPRL2	0.033952	0.07398
NBR2	0.0634	0.073948
NA	-0.0754	0.073909
WDR12	-0.02331	0.073892
AC108727.1	0.297553	0.073892
LCORL	0.026574	0.073889
EYA3	-0.02577	0.073888
NSMCE2	-0.03786	0.073886
AC100830.2	0.177486	0.073884
AC022007.1	-0.10551	0.073861
FRG1JP	-0.04309	0.073845
LRWD1	-0.02565	0.073836
KCTD20	-0.02395	0.073833
PPP1R9A	-0.03241	0.073817

AL133299.1	-0.45506	0.073786
C5orf49	-0.57573	0.073763
SMG1P3	-0.06585	0.073754
PTPRA	-0.02197	0.073749
SNUPN	-0.0379	0.073747
ASAH1	0.023955	0.07372
AL732292.2	0.383222	0.073718
MVB12B	-0.04593	0.073707
PSPC1	0.031608	0.073689
AC078795.2	0.072468	0.07366
CLCF1	0.082791	0.073653
AC091117.1	0.040735	0.073652
RARA	0.036431	0.07364
MYO15B	-0.05773	0.073629
AC108047.1	0.139687	0.073621
QSOX2	-0.02363	0.073607
PCDH8P1	-0.51302	0.0736
CNOT3	0.030354	0.073575
AL353708.3	0.06688	0.073532
DDX11L5	-0.41747	0.073515
SLC45A3	-0.04582	0.073509
SREBF2	0.030026	0.073457
TOM1	-0.03497	0.073376
ARL5A	0.025137	0.07336
AP004608.1	0.17717	0.073358
ERCC5	0.047055	0.073324
C2orf68	-0.02466	0.073314
PRMT9	-0.04339	0.073286
PPA2	-0.02879	0.073268
SLC6A6	0.02764	0.073267
AC020663.1	-0.22885	0.073229
SFTPD	-0.22498	0.073225
IPO8	0.024173	0.073211
NA	0.661429	0.073173
NA	0.266732	0.073154
MIR3677	0.214034	0.073148
ODC1-DT	-0.23915	0.073116
TPRG1	0.485748	0.073115
KNOP1	0.021955	0.073094
PLA2G15	-0.05351	0.073092
MYH13	0.06477	0.07309
AC084018.1	0.060338	0.073069
HBS1L	-0.02559	0.073035
CALCA	0.561058	0.073029
EIF3LP3	0.561058	0.073029
PTH2R	0.039498	0.073027
ACAA1	0.032392	0.073025
AC079780.1	-0.36864	0.073004
MIR181D	0.610989	0.072981
NA	0.610989	0.072981

RFWD3	-0.02306	0.072902
LAPTM5	-0.42831	0.072899
DNAJB14	0.027891	0.072869
C18orf32	-0.09508	0.072839
C1QBP	-0.01967	0.072809
NEUROG2	0.216804	0.072809
GRTP1	-0.05099	0.072796
AL136304.1	-0.1175	0.072763
SLC25A11	0.02626	0.072761
RAB1C	0.586873	0.072758
U82695.1	-0.55875	0.07274
MFF	0.025746	0.07268
N6AMT1	-0.02688	0.07266
MIR4513	-0.42706	0.07266
AIDA	-0.02239	0.072542
AC010883.1	-0.06559	0.07254
NPHS1	0.324109	0.072537
BRIP1	0.030368	0.072526
MIR1972-1	0.557097	0.072515
ARL6	0.038866	0.072509
GRK4	0.046492	0.072467
SOCS2-AS1	-0.03543	0.072454
AP000763.2	0.135192	0.072451
TMEM250	-0.02581	0.072439
AC016586.1	-0.03036	0.072397
AP002992.1	0.305668	0.072361
SPATA25	-0.07421	0.072347
ZUP1	-0.03535	0.072328
KCMF1	0.024802	0.072308
KRT19	0.209405	0.072302
AC073167.1	-0.07589	0.072293
TMED6	0.124178	0.072239
KIF28P	-0.11376	0.072219
ATAD1	0.021439	0.072211
AC005865.2	0.652592	0.072209
RNU6-807P	0.652592	0.072209
SRSF6	-0.01857	0.072202
AC000089.1	0.270762	0.072145
AL158825.2	-0.17497	0.072106
AC125257.1	0.039923	0.07208
MYT1	0.042639	0.07207
BMS1	-0.02164	0.072064
MDC1-AS1	0.045143	0.072059
SHTN1	0.041466	0.072056
TMEM246	-0.036	0.072042
DDX6	-0.0276	0.072038
AC006254.1	-0.26465	0.07202
HSPE1P11	-0.50339	0.072012
RPL21P44	-0.50339	0.072012
NA	-0.50339	0.072012

RAB39B	0.031149	0.072012
CKAP2	-0.02658	0.072007
LINC02106	0.08137	0.072004
AC005786.2	0.345577	0.071988
RBMS2	0.045149	0.071981
AC005410.2	0.602478	0.071975
PRR14	-0.03884	0.071974
GFRA3	-0.12936	0.071918
DSCR9	0.237889	0.071915
OSBPL6	0.062574	0.071875
NA	-0.25813	0.07187
PSMA2P3	0.316325	0.071864
ACOT4	0.167218	0.071863
AC027644.2	0.343238	0.071854
RPS26P47	-0.30537	0.071832
NA	0.048426	0.071816
EML1	-0.03378	0.071794
C8orf76	0.075378	0.071758
SYN1	0.046387	0.071754
SNORD72	0.369174	0.071739
UST-AS1	-0.22275	0.071732
MICD	-0.5153	0.071731
SULT4A1	-0.05371	0.071708
INTS6	0.029188	0.071707
S1PR2	0.098827	0.071696
GAB1	-0.02747	0.07168
CBFB	-0.02649	0.071657
MYO1C	0.022213	0.071645
DLG1-AS1	0.142271	0.07163
ZNF829	-0.04153	0.071622
CLCN3	0.026377	0.071596
STUB1	-0.0201	0.071544
NA	-0.10585	0.071539
GDPD3	0.107528	0.071518
CCNJL	-0.03209	0.071516
PRR3	0.024541	0.071438
LPIN2	-0.02821	0.071417
NA	0.096765	0.07141
FOXK2	-0.02105	0.07141
OLA1	0.028037	0.071391
EPRS	0.021313	0.071389
LINC01932	-0.66007	0.071348
AC133555.4	-0.66007	0.071348
PRDX3	-0.02399	0.071347
Z99289.1	0.109153	0.071343
HNRNPUL1	-0.02024	0.071335
GPATCH4	0.023974	0.071313
ILF2	0.018025	0.071297
AC011444.1	0.145281	0.071284
SNORD17	-0.15046	0.071278

TTC33	-0.04141	0.071271
VASP	0.027384	0.071245
IGF1R	-0.02955	0.071226
AC008735.1	-0.18988	0.071189
TSTD2	0.022269	0.071162
NA	0.341541	0.071159
LMF1	-0.06582	0.071157
AL512598.1	0.372753	0.071132
SPAG9	0.019408	0.07112
LINC02376	0.402142	0.071097
PSMG3-AS1	0.046784	0.071052
AC114728.1	0.589124	0.071042
THAP12	0.025104	0.071029
BEST3	0.55777	0.071019
AC147651.3	0.55777	0.071019
LINC00652	-0.15352	0.071004
KIN	-0.02934	0.070968
CCDC159	-0.03329	0.07095
RPL12P8	-0.4276	0.070879
CCNL1	-0.02316	0.070877
HIST2H3D	-0.19737	0.070875
PGAP3	-0.04257	0.070875
VPS18	-0.03014	0.070866
IVD	-0.02631	0.070863
EPB41L4A-AS1	0.02584	0.070845
AL121768.1	0.017753	0.070773
CKMT2-AS1	-0.04842	0.070769
AL139349.1	-0.40003	0.070766
AC007952.4	0.208385	0.070766
ADAM21	0.077432	0.07076
KCNMA1-AS1	0.092437	0.070757
MNS1	0.044523	0.070724
G6PC3	-0.02428	0.070694
OTUD4P1	0.767597	0.070668
GABBR2	0.767597	0.070668
DYDC1	0.767597	0.070668
LMOD2	0.767597	0.070668
AZU1	0.767597	0.070668
TUBB7P	0.767597	0.070668
GOLT1A	0.767597	0.070668
ASCL3	0.767597	0.070668
PRF1	0.767597	0.070668
GPR132	0.767597	0.070668
AC091868.1	0.767597	0.070668
COL14A1	0.767597	0.070668
NCCRP1	0.767597	0.070668
LYPD2	0.767597	0.070668
RNU5A-1	0.767597	0.070668
RF00019	0.767597	0.070668
SNORD31B	0.767597	0.070668

C1orf195	0.767597	0.070668
RNU6-969P	0.767597	0.070668
RNU6-80P	0.767597	0.070668
AL391416.1	0.767597	0.070668
RNA5SP296	0.767597	0.070668
AL121990.1	0.767597	0.070668
AC007386.1	0.767597	0.070668
AC087071.1	0.767597	0.070668
AL390774.2	0.767597	0.070668
AC092431.1	0.767597	0.070668
HMGN2P20	0.767597	0.070668
AC007879.3	0.767597	0.070668
LINC00345	0.767597	0.070668
AL138760.1	0.767597	0.070668
AC073336.1	0.767597	0.070668
AC117944.1	0.767597	0.070668
RPS3AP12	0.767597	0.070668
AP001056.1	0.767597	0.070668
AC137055.1	0.767597	0.070668
NA	0.767597	0.070668
NA	0.767597	0.070668
NA	0.767597	0.070668
RPS12P31	0.767597	0.070668
AC096576.1	0.767597	0.070668
AC012377.1	0.767597	0.070668
AC093879.2	0.767597	0.070668
ZNF969P	0.767597	0.070668
RNU2-69P	0.767597	0.070668
NA	0.767597	0.070668
NA	0.767597	0.070668
AP003469.2	0.767597	0.070668
LINC02099	0.767597	0.070668
AC067904.2	0.767597	0.070668
AP000793.1	0.767597	0.070668
AC127526.2	0.767597	0.070668
AC109635.6	0.767597	0.070668
AC009779.1	0.767597	0.070668
AL008628.1	0.767597	0.070668
GOLGA8S	0.767597	0.070668
AC007922.2	0.767597	0.070668
AC018521.7	0.767597	0.070668
FDX2	0.767597	0.070668
NA	0.767597	0.070668
AL022345.2	0.767597	0.070668
LINC01396	0.767597	0.070668
GDAP1L1	0.038242	0.070654
CSNK1G1	-0.0245	0.070641
RF00409	-0.22962	0.070619
AC245884.3	0.17285	0.070608
C21orf91	-0.03988	0.070596

NA	0.503657	0.070584
RN7SL530P	0.503657	0.070584
TRPT1	-0.03284	0.070553
AC113398.1	0.585007	0.070549
ALG1L2	0.27699	0.070542
MZF1	0.026564	0.070539
AC073611.2	0.031507	0.070519
CUBN	0.102924	0.070516
NA	-0.1981	0.070469
LEPR	0.04835	0.070453
NRN1	0.040893	0.070452
SRI	-0.02182	0.070445
PHACTR2	-0.03201	0.070439
IGHV3-35	0.426377	0.070425
LINC00310	-0.26726	0.070405
NA	-0.03659	0.070397
KATNB1	-0.0279	0.070375
AC092161.1	-0.03762	0.070348
PHB	0.01928	0.07033
EFNA2	-0.05129	0.07033
FBXO10	-0.04275	0.07031
CYTH3	0.031978	0.070309
ENPP7P4	0.366634	0.070306
SART3	0.02408	0.070301
AC097662.1	-0.13433	0.0703
ITPRIP	-0.03669	0.070285
EXOSC10	-0.02241	0.070277
AC004449.1	0.114903	0.070274
RNU6-531P	0.302865	0.070264
KMO	-0.03184	0.070226
SYT12	-0.07804	0.070212
LRRC39	-0.04642	0.070206
RPL15P2	0.22897	0.070192
EDRF1-DT	0.146343	0.070175
SPSB1	0.067419	0.070157
AC002310.2	-0.03833	0.070151
AL161909.1	-0.13913	0.070145
RPS15AP10	0.265544	0.070115
ABHD16B	-0.05626	0.070113
AC068234.2	-0.30543	0.070111
RASGRP1	-0.10663	0.070064
ELAVL3	0.028643	0.070037
AC011447.3	-0.09439	0.070029
AC116612.1	0.464085	0.070024
TPT1P4	-0.38996	0.070008
KBTBD8	0.050893	0.070006
MAEA	0.022507	0.070006
NA	0.045599	0.070004
UPF2	0.026004	0.069992
GRM7	-0.06011	0.06999

AC008537.2	0.093571	0.069984
NA	0.038306	0.069981
PRR29	0.134967	0.069976
CCDC168	-0.28401	0.069958
MOGS	-0.02869	0.069948
FLVCR1	-0.02786	0.069924
FAM200B	-0.02788	0.069917
NA	-0.4059	0.06991
ZFX	-0.02328	0.069894
INTS5	-0.02936	0.069877
NAA50	0.021524	0.06986
ZNF687	0.027697	0.069849
NA	0.056996	0.069843
MYL12A	-0.02115	0.069835
LINC01359	0.200622	0.069817
ZNF775	-0.03702	0.069815
JPX	0.030377	0.069798
AC018761.4	0.062814	0.069759
AC019254.1	0.226459	0.069747
RF00019	-0.46663	0.069745
LINC02317	0.538992	0.069731
NA	0.538992	0.069731
PROS1	-0.03688	0.069714
PRKCZ	0.036495	0.069697
SLC10A5	0.145367	0.069693
TMEM87A	0.025735	0.069685
CES4A	0.078341	0.069671
TAS2R31	-0.28646	0.069593
YTHDF3	0.022762	0.069579
AC012435.1	-0.09525	0.069578
FYN	-0.02136	0.069575
TRHR	-0.36224	0.069561
CEP152	-0.03513	0.069536
AC012557.1	-0.20608	0.069528
XDH	0.416256	0.069521
SMO	0.031279	0.069488
SELENOV	-0.36985	0.069468
AF131216.3	0.153837	0.069466
MT1E	-0.06815	0.069463
ZBTB46	-0.05318	0.069454
GPR89B	-0.09802	0.069432
ARL15	0.047812	0.069424
NA	0.060336	0.069418
ATF6	0.021377	0.069385
AL391840.2	-0.53298	0.069383
NA	-0.53298	0.069383
GTF3C2-AS1	0.027701	0.069381
MDH2	0.019756	0.069377
AC005838.2	-0.28969	0.069363
GNG11	0.031676	0.069353

CCDC7	-0.12886	0.069348
LRRC20	-0.03258	0.069341
SUCO	-0.02104	0.069328
NA	0.07117	0.069322
HM13	-0.02203	0.069309
SLC32A1	0.494495	0.069282
AL160286.1	0.494495	0.069282
NA	0.248484	0.069272
CWC22	0.026041	0.069255
SPTAN1	-0.02094	0.069202
AC116049.2	-0.18517	0.069169
DOHH	0.032219	0.069142
AC084398.2	-0.10252	0.069125
AC004771.5	-0.20463	0.069123
CASP9	0.040073	0.069105
MAGEA5	-0.17193	0.069081
MOB4	-0.03658	0.069076
SRGAP2B	-0.0602	0.069073
HIKESHI	0.03107	0.069061
SSPN	0.059973	0.069052
ATRNL1	0.026523	0.068999
RN7SL449P	0.414419	0.068983
FOXO1	-0.05533	0.068971
AC004160.1	-0.0509	0.068942
GCC1	-0.0237	0.068936
THOC7-AS1	0.419454	0.068905
SLC22A1	0.347895	0.068884
C5orf63	0.041839	0.068866
NA	0.189042	0.068857
AC005154.1	-0.18089	0.068835
AC025766.1	-0.19676	0.068826
MIR3613	-0.58362	0.0688
AC025165.4	0.311768	0.068797
NA	-0.0442	0.068753
UVSSA	0.031521	0.068719
MCC	-0.02625	0.06871
CEP170P1	0.084341	0.068705
NA	-0.45691	0.068686
AC005519.1	-0.1011	0.068662
ZBTB2	0.027372	0.068652
ZNF697	-0.02938	0.068615
RNF207	0.045849	0.068578
HELB	0.057362	0.068558
TEX261	-0.02315	0.068547
GAS5-AS1	0.037372	0.068544
CBLN4	-0.09199	0.068541
AC068987.4	0.069734	0.068536
TPI1P1	-0.10049	0.068527
DEFB109F	-0.16699	0.068516
MTUS1	-0.03958	0.068511

AP1G1	-0.02258	0.068511
GK5	0.040014	0.068484
SYNE1	0.023192	0.068471
PSMD6-AS1	-0.21706	0.068454
MITF	-0.05418	0.068432
HMGB1P14	-0.21669	0.068401
SARDH	-0.04786	0.068394
TRIM73	0.176231	0.068394
ZNF618	-0.02958	0.068392
FREM1	-0.06138	0.068345
KAT8	-0.02619	0.068333
IAH1	0.020651	0.068297
MEIS1-AS3	0.168689	0.068283
ERI3-IT1	-0.3447	0.06826
TMOD1	-0.02098	0.068227
SLC35C1	0.031282	0.068193
AL355472.1	-0.14282	0.068174
AL445183.1	-0.37224	0.068162
TRAPPC3	-0.02655	0.068153
FEZ1	-0.02928	0.068103
SLC22A25	0.209976	0.068079
ZNF236	-0.03268	0.068061
USP32	-0.02232	0.068011
SLC25A34-AS1	0.279756	0.067998
AFDN	-0.0208	0.067982
PLEKHF1	0.077563	0.067978
AC113423.2	-0.23069	0.067977
TSC22D4	0.031521	0.067937
AC139795.1	0.076299	0.067937
MPP4	-0.08775	0.067916
TIAM2	0.036751	0.067916
AC009237.15	-0.33744	0.067914
TFB1M	-0.03444	0.067913
NME5	-0.106	0.067911
IDH3A	0.024331	0.067887
FAM118A	-0.03064	0.067854
AP000350.2	-0.26023	0.06785
AC106820.3	0.138877	0.067846
GTDC1	-0.03474	0.067838
RICTOR	-0.02557	0.067826
LRRC69	0.162224	0.067813
PTTG3P	-0.56234	0.067785
AC008267.4	-0.56234	0.067785
AP000462.1	-0.56234	0.067785
CAPN10	-0.02748	0.067779
NPIPB12	0.112097	0.067771
MCMBP	0.022235	0.06775
AC073592.1	0.382136	0.067736
TMEM14C	0.026494	0.067717
SPART-AS1	-0.15229	0.067711

TMEM151B	-0.03659	0.067687
HIST1H2BN	-0.05739	0.067685
SSR1	0.023088	0.067675
ISOC2	0.026094	0.067668
AC004980.1	-0.03737	0.067541
NKTR	-0.02112	0.067541
TGFBRAP1	-0.02544	0.067538
C1QL1	0.059737	0.067537
AC090286.2	-0.57447	0.067535
AC073050.1	-0.27414	0.067534
AC009087.1	0.080509	0.067531
PEMT	0.030083	0.067518
YEATS2-AS1	0.035461	0.067509
PKP1	-0.42205	0.067484
ZWINT	0.029119	0.067429
TMEM240	0.076497	0.067427
DPPA3P1	-0.12377	0.0674
SMAP1	-0.02551	0.067398
MCRS1	0.024811	0.067391
DMAC2	0.026382	0.067372
DNMBP-AS1	-0.07721	0.067372
AC004069.1	0.211942	0.067356
TXK	0.308376	0.067355
TMEM221	0.199377	0.067325
NA	0.017738	0.067323
NME4	0.018804	0.067316
LINC01948	0.140734	0.067262
KANSL1-AS1	0.128995	0.067241
ZNF813	0.046878	0.067233
ZNF471	0.057301	0.067213
PPM1L	-0.02629	0.067203
NA	-0.10031	0.067195
ALDH3A1	0.176815	0.067193
AC005013.1	0.071073	0.067182
LASP1	-0.02311	0.067176
CETN3	0.035708	0.067172
KHDRBS3	-0.0251	0.06716
TMCC1-AS1	-0.04513	0.067155
CENPS	0.045942	0.067137
NLE1	0.028387	0.067108
ZNF596	0.047897	0.067103
RAE1	-0.02318	0.067075
MYT1L	-0.02986	0.067053
ARIH2OS	-0.04548	0.067041
SLC17A8	0.421593	0.067023
RRAD	0.060199	0.067013
OTULINL	-0.03872	0.066983
AP002336.3	0.082943	0.066976
CRTAP	-0.01994	0.066969
ENTPD7	0.035573	0.066961

PKD2L2	0.03853	0.066957
ARL3	-0.02728	0.066957
ANKLE1	-0.03791	0.066894
POP5	-0.02812	0.066877
AC010680.4	0.094006	0.066867
AL031668.1	-0.38315	0.066859
LIPI	-0.29068	0.066843
SEPT14P12	-0.33072	0.066839
MIRLET7BHG	0.042249	0.066825
CD83	-0.04488	0.066819
NA	-0.11078	0.066807
AC008755.1	-0.0286	0.066779
CDCA4	0.025252	0.066769
RPL12P37	0.354519	0.066765
KATNBL1	0.025325	0.066723
PLCH2	0.155729	0.066718
GNB3	-0.03931	0.066667
BUD13	0.036718	0.066666
CPNE1	-0.02695	0.066606
UFL1	-0.03197	0.066596
WDFY2	-0.03559	0.06659
VPS53	-0.02399	0.066589
LAMA2	0.488588	0.066586
HIGD1AP11	0.488588	0.066586
COL28A1	-0.28313	0.06658
GPLD1	0.060104	0.066577
TLR5	-0.35922	0.066557
ADA2	-0.30344	0.066544
ST13P4	0.442557	0.06653
FADS1	-0.02028	0.066506
WDFY3-AS1	-0.12303	0.066485
ZBTB8OS	0.035134	0.066474
MPP5	-0.02974	0.066464
MGA	0.019295	0.066461
BTBD2	-0.02763	0.066434
AP001471.1	-0.44393	0.066416
AP001350.1	-0.44393	0.066416
AC011498.4	0.508213	0.066413
BDKRB2	0.090934	0.066413
KPNA4	-0.02137	0.066396
GTF2H5	0.030561	0.066385
STAC2	0.079515	0.066333
NA	-0.11959	0.066313
EEF1B2P6	0.160152	0.066304
INTS13	0.028514	0.066302
BICC1	-0.02934	0.066235
ARHGEF10	-0.02867	0.066228
NEPRO	-0.02321	0.066219
AC009812.1	0.085255	0.066199
DNASE2	-0.03138	0.066195

CDK17	0.027088	0.066147
MTCH1	0.022257	0.066122
FAM161B	-0.02811	0.066119
ZGRF1	-0.03336	0.066115
ZCCHC2	0.04144	0.066113
HCST	-0.13479	0.066101
NA	-0.37915	0.066025
AC133435.1	-0.27758	0.065993
NOX4	-0.07966	0.065985
SEPT11	-0.02229	0.065958
MARCH8	-0.02885	0.065953
KLHL23	0.025324	0.065945
AC027808.2	0.560597	0.065938
NA	0.023068	0.065922
AC046143.1	0.043808	0.06591
SOX9-AS1	0.168808	0.065907
AC009220.3	0.193238	0.065886
AL021937.1	0.185773	0.065874
IL6ST	0.020499	0.065857
RASGEF1C	0.448635	0.065849
SREBF2-AS1	0.046611	0.065834
CHP1	0.02542	0.065814
ZYX	0.052479	0.06581
DNTTIP2	-0.02536	0.065795
AC109992.2	0.252102	0.06578
SEC24C	0.017413	0.065767
NALT1	0.241581	0.065721
TMEM104	-0.02723	0.065704
COX6A1	0.02737	0.065676
RANP4	0.409365	0.065672
TSR1	0.017197	0.06566
TFAP4	0.029178	0.065653
WWC2	0.028485	0.065648
NAA25	-0.02129	0.065647
B3GNT9	-0.03075	0.065639
MAST4-AS1	-0.15895	0.065633
AL118516.1	0.056544	0.06563
AC010271.1	0.199563	0.065618
ATP2B1-AS1	-0.0639	0.065603
EEF1A2	-0.02503	0.065558
AC092338.2	-0.35124	0.06555
DPP6	-0.03208	0.065517
KCNJ12	-0.09573	0.065508
MCM10	0.060874	0.065502
RSPH10B	-0.03459	0.065502
LINC01697	-0.40362	0.065463
AP000721.2	0.398867	0.065449
AL590399.4	0.268885	0.065445
RORA	-0.03211	0.065435
CCDC28A	0.033	0.065423

ZNF385A	0.090739	0.065422
RAB30-DT	-0.05796	0.065418
ISX	-0.33792	0.065417
SELENOW	-0.02232	0.065387
HSD17B10	0.019724	0.065282
S100A13	-0.09558	0.065257
XRCC6P2	0.272167	0.065219
SLC25A40	0.034616	0.065208
AC006538.2	0.090662	0.065203
SDSL	0.04703	0.06519
PNMT	-0.07077	0.065173
AC002351.1	-0.08084	0.065149
DHFRP1	0.157704	0.065137
NA	0.06869	0.065103
ANAPC13	0.025774	0.065088
YWHAZP2	0.343649	0.065073
BTRC	-0.02393	0.065041
SARM1	-0.02477	0.065026
MSH3	-0.02913	0.065019
CCDC84	-0.02913	0.065015
AL662797.1	0.017872	0.065008
NA	0.109457	0.064985
POPDC3	0.031309	0.064965
ANP32A	0.019426	0.064942
CDC6	-0.03105	0.064899
ENTPD1-AS1	0.038756	0.064893
PODNL1	0.075596	0.064866
AP001453.2	-0.06433	0.064847
SNORD117	0.386117	0.064842
AC090181.1	-0.11265	0.064832
IGHVIII-38-1	0.206717	0.064823
AC099850.3	-0.04322	0.064805
SNORA71C	-0.16651	0.064789
MAP4K4	-0.0166	0.064783
AL117344.1	-0.28239	0.064773
AC116025.1	-0.39679	0.064754
PLXNB1	0.031475	0.064729
PDCD10	0.025977	0.064714
AC084824.1	-0.10432	0.064711
CCDC24	0.04915	0.0647
DHX9	-0.01909	0.064653
DEK	0.023271	0.064646
FIG4	-0.03005	0.064601
NDUFS4	0.025507	0.064592
AC022211.1	-0.06397	0.064579
HSP90AA2P	0.151137	0.064576
AL162231.4	-0.48288	0.064503
AC104316.1	0.080739	0.06448
ZNF597	-0.06712	0.064478
AC004975.1	0.244922	0.064476

AC107032.2	0.332127	0.064466
CACNA1D	0.084428	0.06446
AC122129.1	-0.13847	0.064436
PPCS	0.030459	0.064422
TXLNA	-0.01932	0.06442
KDM6B	0.034511	0.064418
SNX2	-0.0238	0.064412
TRIB2	-0.02157	0.064387
TSPAN4	-0.02959	0.064383
GTF3C4	-0.02009	0.064381
SYNJ1	0.032382	0.06438
NA	0.094335	0.064344
AC091860.1	0.342593	0.064338
AP003721.4	-0.27779	0.064304
ESD	-0.02326	0.0643
AMOT	-0.03264	0.064291
REPS1	-0.02381	0.06428
AF235103.1	0.091395	0.064254
NFXL1	-0.02459	0.064252
NA	0.248441	0.064252
AL354813.1	0.07965	0.064238
NA	-0.20383	0.064213
SYT17	0.053956	0.064203
LRRC58	-0.01965	0.064162
AC011921.1	-0.12878	0.064162
AL354892.2	0.062787	0.064131
LIPA	-0.02518	0.064128
U47924.1	0.037651	0.064107
VKORC1L1	-0.02097	0.064107
GLUD2	-0.36651	0.064048
ST3GAL6	0.028224	0.06403
AL365205.1	-0.03858	0.064028
THADA	0.022032	0.064026
NA	0.058049	0.064025
AL137003.1	-0.18549	0.063961
CDCA4P4	-0.40444	0.063937
LIMS1	-0.02495	0.063873
AC139426.3	0.423731	0.063864
ENO1	-0.02018	0.063863
FADD	0.025126	0.063859
CYC1	-0.01753	0.06385
RGL3	-0.05048	0.063839
NA	0.34055	0.063811
TXNDC2	-0.29805	0.063793
KDM4C	-0.03818	0.063773
SLC12A9	0.027629	0.063771
MUC6	0.176211	0.063765
TDP2	0.025822	0.063731
TMEM9	0.021622	0.063719
VASH2	-0.02928	0.063716

PPT1	-0.01908	0.063705
RAP1B	-0.02025	0.063681
UBR1	0.021448	0.063675
SFRP4	-0.14708	0.063673
MROH5	-0.3986	0.063661
LINC01970	-0.2001	0.06365
OLFML3	0.057418	0.063637
FAM157C	0.25272	0.063611
AC021087.2	0.232183	0.063604
HSD17B14	-0.04429	0.063585
METTL15	0.03301	0.063538
PUSL1	0.038668	0.063529
MYCN	0.055173	0.063519
RPL22L1	0.019966	0.063513
AC021483.1	0.340591	0.063507
PFN1P11	0.421047	0.063454
PEX2	0.027399	0.063433
NUDT5	-0.01954	0.063429
SFR1	0.049601	0.063416
CRTC3	-0.03002	0.063416
SYAP1	0.019457	0.063414
PIK3CD	0.046537	0.0634
PMS2P7	0.054517	0.063398
MTDH	0.018891	0.063389
CLCN2	0.046906	0.063388
FNBP1P1	-0.06304	0.063382
HIST1H2BD	-0.02828	0.063347
FKBP11	-0.03676	0.063335
EHBP1	-0.02207	0.063333
SESN3	0.021763	0.063333
AP006333.2	-0.4328	0.063322
NA	0.187636	0.063285
FBXO22	0.025854	0.063282
AP000936.3	0.446644	0.063274
MTND1P23	0.114289	0.063215
AC010260.1	-0.08974	0.063214
PLEKHJ1	-0.01968	0.063192
RNFT2	0.033209	0.063161
FGF14	0.023429	0.063122
NA	-0.09437	0.063121
ZNF30	-0.03821	0.063093
XRCC5	0.01845	0.06304
WASHC1	-0.03633	0.06302
AC008761.2	0.249891	0.062982
CCDC9	-0.02755	0.062976
AC106782.1	-0.26749	0.062958
MTRNR2L8	0.246958	0.062949
CYP2B7P	0.493879	0.062943
HSPA8P14	0.493879	0.062943
AC005498.2	0.493879	0.062943

ELK3	-0.02223	0.062921
LINC02588	-0.23457	0.062896
SHLD2P1	0.220831	0.062886
AP000569.1	0.263464	0.06288
RF00017	-0.11951	0.062866
AC008080.1	-0.48916	0.062827
LHPP	0.044107	0.062826
FAM131A	0.021306	0.062788
CCDC57	0.02282	0.062777
TMEM209	-0.02318	0.062771
CIAO2A	0.025897	0.062748
HNMT	0.091184	0.062739
PPP2R5C	-0.01818	0.062722
LINC01597	-0.33707	0.06271
ZNF581	-0.02878	0.062682
AP000704.1	-0.08648	0.062665
CDC42EP4	0.023954	0.06266
CBX3	-0.019	0.062633
NETO2	0.022009	0.062627
GAL3ST2	0.149824	0.062622
KLF3	0.02252	0.062598
AC097467.3	0.038568	0.062547
AC011611.5	0.185089	0.062523
AC022150.2	0.115076	0.062501
PKN1	0.020991	0.062498
AP3S2	-0.03109	0.062486
GTF2E2	-0.02384	0.062474
PTGES2	0.024317	0.062473
NNT	-0.01773	0.062472
AC098820.3	0.286495	0.06246
AL353625.1	0.058722	0.062457
TP53RK	0.029804	0.062449
KEL	0.489911	0.062439
MFSD1P1	0.489911	0.062439
AL356608.1	0.489911	0.062439
ZFYVE1	0.025208	0.062425
AC117503.1	-0.0813	0.062411
APCDD1	0.163534	0.06241
DNAI1	-0.09808	0.062406
ULBP1	-0.48619	0.062389
NA	-0.48619	0.062389
NA	-0.48619	0.062389
SYN2	0.028629	0.062375
WNK4	-0.08384	0.062374
AC104411.1	-0.21516	0.062365
AC068633.1	-0.48887	0.062363
AC005696.3	-0.48887	0.062363
AP003306.2	-0.06355	0.062355
VN1R42P	-0.19029	0.062352
MCOLN1	0.034538	0.062341

AL589763.1	-0.09866	0.062338
ELMOD2	-0.02766	0.062296
MROH6	-0.05243	0.062277
AP4B1	-0.02898	0.062245
MATR3	0.019638	0.062226
POLR3G	0.043297	0.06222
PIK3IP1	0.037265	0.062213
CCDC88A	0.023153	0.062197
TNFRSF4	0.151373	0.062183
NEAT1	0.021287	0.062144
C8orf31	-0.05021	0.06214
SLC41A1	-0.02502	0.062125
ADCY10P1	-0.05096	0.062107
AL357055.3	0.276702	0.062097
COPB2	-0.01939	0.062086
DNAJC1	0.034808	0.062078
AC078889.1	-0.22224	0.06207
AC005747.1	0.061848	0.062046
SERPINB8	0.04413	0.062044
FH	-0.02271	0.06204
RPL23AP64	-0.13307	0.062021
CFAP100	-0.25632	0.062007
CLIC4	0.020567	0.062005
C20orf202	-0.32069	0.062002
MGAM	-0.26794	0.061997
RNU2-27P	-0.44934	0.061987
RPL29P11	-0.12014	0.061852
RNU1-143P	0.23659	0.061834
TBX2	0.021346	0.061807
ACTN2	-0.08876	0.061791
TOP2A	0.021952	0.061789
OTUD6B	-0.02875	0.061775
SCAT2	0.123597	0.061766
AGA	-0.03026	0.061764
TIGD5	0.03351	0.061763
LSM14B	-0.02153	0.061761
MRPS30	0.023591	0.061761
TTC39B	-0.05422	0.06176
KDELR1	-0.01903	0.061736
LRRK1	-0.05009	0.061636
ZNF541	0.337929	0.061629
AC018475.1	0.162387	0.061601
AC091167.1	-0.02435	0.061571
TMEM231P1	0.301277	0.061541
AL049830.3	0.034791	0.061512
EMILIN3	-0.05105	0.061503
CRYL1	-0.06583	0.061478
RNU2-7P	0.344013	0.061447
NA	-0.3247	0.061434
AL355574.1	0.126062	0.061433

EIF4E2	-0.02076	0.061412
ZBTB38	0.022193	0.061357
FBXW11	0.01765	0.061353
GPKOW	0.024408	0.061348
THAP1	-0.03292	0.061347
RF00019	0.269957	0.061332
RSRC1	-0.02738	0.061305
BAK1	-0.03153	0.061284
AC005037.1	0.043822	0.061278
MRPL12	-0.02922	0.061272
EGLN1	0.019636	0.061255
AC020612.3	-0.10934	0.061239
COX6A1P2	0.107351	0.061198
AL162586.1	0.030149	0.061195
SCGN	-0.04663	0.061159
JAZF1-AS1	-0.08462	0.061131
AC098831.1	-0.46921	0.061087
AC098679.2	-0.46921	0.061087
INSR	0.03296	0.061084
MID1	-0.03526	0.061067
NA	-0.27201	0.061054
AGTPBP1	-0.02726	0.061011
RIN1	0.041818	0.060984
SIAH3	0.16259	0.060951
MRPL3	0.019699	0.06095
LHFPL5	0.035035	0.060937
ARF3	0.021173	0.060934
TSHZ1	-0.02954	0.060923
TEAD3	0.049642	0.060917
CDC20P1	-0.16372	0.060903
TRAPPC11	0.023719	0.060889
TMEM178A	0.338069	0.060885
CLMP	-0.03214	0.060884
NCKAP5L	0.058475	0.06087
AC016825.1	-0.07852	0.060869
RN7SL280P	-0.37916	0.060814
ZNF283	-0.03709	0.060812
HAUS3	-0.03021	0.060805
AL590004.3	-0.14522	0.060785
C22orf15	-0.10206	0.060763
DICER1	0.023797	0.060736
PCOLCE-AS1	-0.02266	0.060732
KDM3B	0.019651	0.060726
GNPTG	-0.0216	0.060718
TSSK4	0.051947	0.060715
FZD3	0.024539	0.060706
RBFOX1	-0.30377	0.060701
LINC01778	-0.51126	0.060679
DPP8	-0.0212	0.060669
ABHD6	-0.04165	0.060659

RBM26	0.018553	0.060582
DENND6A-AS1	-0.08371	0.060552
AC073957.3	0.031598	0.060541
IFT172	0.023684	0.060532
SALL2	-0.03198	0.06048
TGFB1	0.023915	0.060435
ISG15	-0.19434	0.060408
SEPT7P6	0.483729	0.060399
Z97653.1	-0.49408	0.060396
THUMPD3	0.020144	0.060373
C5orf46	-0.1137	0.060364
PHACTR1	-0.0373	0.060355
AL031429.1	0.395741	0.060343
APAF1	-0.0257	0.060343
P2RX7	0.055698	0.060336
PCGF6	-0.03006	0.060335
CICP4	0.412204	0.060332
ALKBH4	0.030309	0.06032
ANXA4	0.025897	0.060309
MTIF2	-0.02351	0.060269
GBGT1	-0.06942	0.060267
AC008438.1	-0.09975	0.060257
C1orf194	-0.32944	0.060245
CYP1A2	0.37151	0.060243
OSGEP	-0.02687	0.060218
SYTL3	-0.06918	0.060214
MIR302A	-0.34632	0.060214
AC123768.2	0.144889	0.060186
NUTF2	-0.01671	0.060172
FHL3	0.041801	0.060146
AL359878.2	-0.40956	0.060104
NA	-0.40956	0.060104
TOB2P1	0.154283	0.060104
TRABD2B	-0.12455	0.060092
CACTIN	0.025819	0.060083
IPP	0.032607	0.060078
NA	-0.07538	0.060073
BLCAP	-0.02014	0.060059
GIP	0.365102	0.06005
HNRNPM	-0.01688	0.060048
LINC01993	-0.22589	0.06004
AP002433.1	0.043823	0.060021
ARMH4	0.331604	0.060007
AP002340.1	0.256124	0.059972
AL139274.2	0.082057	0.05997
ADH1A	0.206967	0.059967
PTGER3	-0.04655	0.059945
SORD2P	0.058889	0.059942
AC018521.2	-0.04015	0.059916
CAHM	-0.09059	0.05991

XRCC1	0.024806	0.059872
CCDC33	-0.11963	0.05986
AC138356.1	-0.09874	0.05986
LUC7L2	-0.01859	0.059816
CNNM4	0.032998	0.059799
ANKRD29	-0.19054	0.05979
PCMT1	-0.02291	0.05978
AC008945.1	-0.11124	0.059754
KRCC1	-0.02428	0.059753
RPL7P8	0.404262	0.059752
AL139353.2	-0.07875	0.059735
ACAD8	-0.03192	0.059729
LRRC28	0.035362	0.059708
SIRT4	0.0653	0.059707
SETSIP	0.40713	0.059706
AC022028.2	-0.13573	0.059683
CMPK1	0.024558	0.059663
NA	-0.02785	0.059632
USP9X	-0.01643	0.059607
VBP1	0.02552	0.059595
AP001000.1	-0.0934	0.059595
BRWD1-AS1	0.245889	0.059518
NAPG	-0.02348	0.059498
SLC25A13	0.018034	0.059489
VPREB3	0.436736	0.059479
ARMCX7P	0.436736	0.059479
AC115102.1	0.436736	0.059479
PGAP1	-0.01891	0.059474
ZNF773	0.051762	0.05945
TOP2B	-0.01778	0.059436
TSPAN8	-0.0615	0.059417
KY	-0.11714	0.059409
UTP15	-0.03072	0.059379
HINFP	0.027984	0.059355
PLEKHA8P1	-0.03824	0.059347
AC092354.2	0.23279	0.059295
NPM1P27	0.055963	0.059269
PTPN12	-0.01915	0.059224
HPS3	0.02477	0.059216
KCTD1	0.026912	0.059209
WRB	0.02371	0.059198
RRM1	-0.02167	0.059183
AC009292.2	0.490905	0.059147
FOXN2	0.027582	0.059126
AC007679.1	0.080218	0.05911
NOC3L	0.020936	0.05909
AF186192.2	-0.07296	0.059084
LSM12	0.025854	0.059069
RNU6-722P	-0.2214	0.059068
LYNX1	-0.05415	0.058973

LINC02453	-0.21132	0.058969
ERI2	0.027355	0.058952
TBC1D1	0.023031	0.058918
ECSIT	-0.02558	0.058907
MED27	0.031453	0.058907
NA	0.038603	0.058895
ERAP2	-0.03725	0.058883
NA	0.160128	0.058883
WDR75	0.022094	0.058877
RBMXL1	0.026175	0.058854
NA	-0.36502	0.05885
PDK2	0.028046	0.058816
RAD52	-0.03164	0.058815
ANKRD65	-0.22205	0.058808
ZNF76	0.026171	0.058787
TOGARAM2	0.076247	0.058764
HLA-A	0.016018	0.058763
B3GNT3	0.367046	0.058762
RPS21P1	-0.42302	0.05876
AL096869.2	0.113673	0.058757
AC132008.1	-0.44019	0.058733
AL137159.1	0.287395	0.058723
AC007405.3	0.175962	0.058723
AC134682.1	-0.23769	0.058722
CHD5	-0.07774	0.058714
RPL21P4	0.356225	0.058704
AC112484.2	-0.13073	0.058702
JPH2	0.103015	0.058678
ARL8A	-0.01903	0.058671
AL034347.1	-0.30524	0.058661
PRKRA	0.019704	0.058653
RHOU	0.028128	0.058648
AC245052.2	-0.33717	0.058609
RBM26-AS1	-0.05046	0.058585
PIGBOS1	-0.04831	0.058557
AC010980.1	0.039268	0.058555
KCTD9	0.025772	0.05854
AC091887.1	-0.02672	0.058519
AIMP1	-0.02187	0.058516
SLC25A5P1	-0.2537	0.058478
IQSEC2	0.050533	0.058467
CYTH1	0.016122	0.058462
AC016722.2	0.030745	0.058448
USP8	-0.01872	0.058429
CDK5RAP2	0.021494	0.058422
MYH4	0.26192	0.058415
TUBG1P	0.173869	0.058409
CSF3R	-0.2111	0.058406
GTF2IP14	0.13347	0.058401
CLSPN	-0.06859	0.058398

RBM33	0.022076	0.058394
GRK2	-0.01986	0.058377
RPS15AP1	-0.19107	0.058351
AC017048.3	-0.12285	0.058347
SLC4A5	-0.05725	0.058345
KTN1-AS1	-0.05847	0.058328
AACS	-0.02747	0.058305
NA	0.041415	0.058296
AC005154.3	0.157158	0.058269
DCTD	0.018437	0.058246
AC098484.2	0.193461	0.058241
AC010624.2	-0.17361	0.058218
EIF2AK2	0.021589	0.058176
AC006329.1	-0.16006	0.058164
WHAMMP2	0.058959	0.058162
AC011446.1	0.030805	0.058158
CREG1	0.025486	0.058156
MRPS17P1	0.206394	0.05815
BAMBI	0.024289	0.058089
AC068580.1	0.178337	0.058075
ZNF888	0.257864	0.058071
PPP1R3E	-0.04195	0.058071
FAM86FP	-0.12296	0.058055
AC139887.2	-0.02985	0.058049
AC073052.1	0.312053	0.058038
AC023906.3	-0.06559	0.058032
AC008035.1	-0.37295	0.058019
RF00019	0.50414	0.058005
GACAT3	0.50414	0.058005
GTSCR1	0.50414	0.058005
NA	0.105601	0.058002
HTT-AS	-0.39771	0.05799
AC246793.1	0.166445	0.057934
GLYCTK-AS1	-0.06646	0.057934
NA	0.322799	0.057927
AC027243.1	-0.06413	0.057914
BRD7P2	0.220393	0.05788
RF00156	-0.25501	0.057871
PRADC1P1	-0.4895	0.057834
PNRC2	-0.08964	0.057818
SLC6A16	-0.31758	0.057813
C1orf109	-0.02007	0.057812
PARGP1	0.071806	0.057763
ANKRD31	0.101718	0.057738
LINC00680	0.026927	0.057726
SYNPR	0.188073	0.057726
KIAA1143	0.018709	0.05772
AC016739.1	0.084255	0.057697
DYNLRB1	-0.01837	0.057688
KIF26A	-0.02461	0.057662

ZNF224	0.023248	0.057638
FTLP3	-0.16673	0.057634
UBE2Q1-AS1	-0.02398	0.057591
NA	0.371385	0.057591
LRRIQ3	-0.08693	0.057586
TRMT2B	-0.02981	0.05758
GALNT7	-0.03129	0.057568
MYH3	-0.05444	0.057563
NA	-0.22835	0.057558
SMARCA2	0.022047	0.057554
PES1	0.019042	0.057543
AC027277.1	-0.04399	0.057524
SNORA2B	0.194082	0.057516
AL355001.1	0.287566	0.057487
CACNA2D2	-0.05666	0.057456
FZD9	-0.06421	0.057433
AC135050.5	-0.09628	0.057414
RF00156	0.516838	0.057365
Z97353.1	0.516838	0.057365
AC073864.1	0.516838	0.057365
AC024619.4	0.516838	0.057365
NA	0.516838	0.057365
AC037487.2	0.516838	0.057365
AL355916.1	-0.431	0.057348
KIAA0895	-0.02661	0.057339
MTX3	0.024144	0.057304
NA	-0.04895	0.057298
AC069499.1	0.061536	0.057266
ST13P20	-0.28461	0.057252
AC104791.1	-0.26732	0.057249
PTPRO	-0.17899	0.057227
SNORD67	0.234276	0.057196
AC105942.1	-0.05634	0.057189
SPATA4	-0.26573	0.057188
TMEM42	0.034245	0.05717
VPS26C	0.017709	0.057168
TMEM70	-0.02552	0.057167
EIF2S3	0.016018	0.057073
NT5DC2	0.019503	0.057072
AC109587.1	-0.02574	0.057068
TCF20	0.021799	0.057046
ESR2	0.093722	0.057029
EXOC3L1	-0.07247	0.05699
ANKMY1	0.027313	0.056976
GANC	0.023489	0.056972
EID2B	-0.05184	0.05697
IL17RC	0.038292	0.056944
NA	0.27458	0.056932
CDPF1	-0.0337	0.056923
RETSAT	-0.02464	0.056911

AC097641.1	-0.33099	0.056901
AC087190.3	0.025809	0.056863
GPSM1	-0.02963	0.056799
PURPL	0.046191	0.056771
NA	0.344388	0.056734
BNIP3P37	-0.28854	0.056715
PAPSS2	-0.03369	0.056704
CCL19	0.228538	0.056697
AC012363.1	-0.04491	0.056694
AC007285.2	0.297176	0.05669
TESC	0.167638	0.056687
CARF	0.032165	0.056686
AC106872.2	-0.41205	0.056656
SCARA5	-0.25258	0.056654
MORF4L1P1	0.035608	0.056639
CARD6	-0.1619	0.056634
AC104211.1	-0.48031	0.056594
RF00394	-0.48031	0.056594
NA	-0.48031	0.056594
HNRNPA1P35	0.293036	0.056571
AC009088.2	0.018525	0.056548
GNRH2	0.183224	0.056545
SLC26A4	0.471797	0.056495
NA	0.072919	0.056461
BRAP	-0.02212	0.056456
CD164	-0.0202	0.056449
RNU7-47P	-0.35067	0.056434
TMCO1-AS1	-0.08308	0.056418
SHLD1	0.052255	0.056407
RNASET2	0.034101	0.056406
CDKL3	0.073041	0.05639
CPA1	-0.21311	0.056322
MON1B	0.025496	0.056318
AC092279.1	0.061085	0.056303
AC092691.1	0.050169	0.056277
SNORA65	0.132156	0.056251
AL035446.1	-0.10127	0.056234
LRRN1	-0.0269	0.056219
USP47	0.019884	0.056213
PIGX	0.026194	0.056145
ENHO	0.044296	0.056144
NCAPH2	0.021579	0.056141
SREK1	0.0191	0.056133
PM20D1	-0.17368	0.05613
AL157871.5	0.175042	0.056103
PCAT6	0.075342	0.056101
GCN1	-0.01865	0.056085
ARHGAP4	-0.03636	0.056062
ADRB2	-0.18801	0.056051
GSTZ1	0.026195	0.056031

TNFAIP8L1	-0.03195	0.056017
FXR1	0.019372	0.056007
CTSD	0.019635	0.055991
AC010198.1	-0.2555	0.055973
AP003390.1	-0.12994	0.055962
DPYD-AS1	0.11458	0.055954
SSR2	0.014618	0.055951
PSME4	0.015956	0.05593
AC245884.1	-0.17526	0.055927
PPP2R1A	-0.01725	0.055922
ADGRA2	0.025931	0.055897
RNASEK	0.066561	0.05589
DHRS13	0.027542	0.055886
ZNF814	-0.0219	0.055853
SLC29A1	0.018121	0.055843
TAL2	0.310776	0.055799
RN7SL812P	-0.37302	0.055749
ASCC2	-0.02025	0.055747
GID4	0.027959	0.055745
NA	0.284182	0.05574
ACOX3	0.028135	0.055729
DOCK9	-0.02991	0.055705
SEPT7	0.020819	0.055644
FBXO42	0.024208	0.055635
CCDC136	-0.02557	0.055621
ANKRD13C	-0.02418	0.055602
DAZAP1	0.015138	0.055592
CX3CL1	0.028833	0.055591
GRSF1	0.016294	0.055579
AC107081.2	0.024619	0.05557
PPP4R1-AS1	0.264024	0.055547
RAB5A	0.017981	0.055543
CDIPTOSP	0.204927	0.055534
IGBP1P1	0.330454	0.05552
DHRS7	-0.02313	0.0555
AP3M1	0.022345	0.055492
RORB-AS1	0.077082	0.055491
AC048341.1	-0.09374	0.055456
KCNK9	0.105384	0.055446
RYR2	0.115484	0.05539
ITGB1BP1	-0.01721	0.055386
ZNF837	-0.0481	0.055385
ANKRD20A4	0.319992	0.05534
LURAP1L-AS1	-0.31516	0.055322
TEFM	-0.02933	0.055315
NA	-0.02333	0.055305
NA	-0.0571	0.055297
CACTIN-AS1	0.030127	0.055277
CTIF	0.022178	0.055275
NAALAD2	-0.03056	0.055262

RPL21P1	0.228104	0.055258
PUS10	0.041729	0.055257
MYB-AS1	0.132347	0.055256
CPSF4	0.018629	0.055236
TRIM3	0.027552	0.055218
ANP32AP1	0.30335	0.055173
L2HGDH	0.035595	0.055158
SLC13A3	0.171147	0.055154
ZGPAT	0.037911	0.055111
TLE5	-0.0174	0.055109
IGHV7-34-1	-0.30003	0.055107
RPL7AP30	0.20906	0.055105
TOM1L1	-0.02216	0.055104
SKIV2L	0.01801	0.055089
LRRC43	-0.21983	0.055088
AC016700.2	0.282047	0.055086
CSPG4P12	-0.04703	0.055083
GALNT13	-0.02636	0.055081
LINC02447	-0.28567	0.055076
MAMDC2	-0.25289	0.05507
RNY1P9	-0.37621	0.055068
FGF14-IT1	0.125442	0.055052
WRAP73	-0.02201	0.055051
MIR942	-0.39668	0.055029
MTCO1P31	-0.39668	0.055029
AC093673.1	0.046483	0.055027
CBLN3	0.051062	0.055027
TSHZ3	0.031729	0.055021
PCF11	-0.01989	0.055012
AC123905.1	0.408401	0.055005
ATXN1	-0.03859	0.055002
GLTP	0.029204	0.054988
DUTP6	-0.12323	0.054953
ADAM32	-0.1229	0.054948
AP003392.2	0.16586	0.054921
NA	0.02295	0.054914
AC078860.1	-0.02886	0.054914
NA	-0.10199	0.054864
AL139275.1	-0.18239	0.054843
HIST2H2BE	-0.02663	0.054842
IMMP2L	0.033217	0.05484
AC011484.1	0.096567	0.054833
UBE2FP1	0.162954	0.05483
RFX2	0.041191	0.054802
ZNF565	0.056896	0.054782
ARHGAP5	-0.01979	0.054769
SETBP1	-0.03301	0.054764
FLNC-AS1	-0.0641	0.054753
RUNDC1	-0.02998	0.054702
ANKDD1A	-0.03162	0.054669

RAD9A	0.022994	0.054667
SEMA3D	-0.08138	0.054667
AC124784.1	-0.42532	0.054642
QPCT	0.245706	0.05462
LTBP4	-0.02891	0.054611
PLD6	-0.05432	0.054601
TNNI1	0.306896	0.054597
RAB11FIP4	-0.03301	0.054592
RGS8	-0.24447	0.054582
EGF	-0.50417	0.054546
MIR4285	-0.50417	0.054546
GAN	0.027708	0.054538
AC107398.2	-0.06872	0.054515
RAB37	0.080899	0.054509
AP001775.2	0.052965	0.054508
AL139396.1	-0.12248	0.054487
PGLYRP1	0.369748	0.054485
MSLN	0.369748	0.054485
AC093525.7	-0.0891	0.054475
AL591895.1	-0.15971	0.054474
SLTM	0.017339	0.054448
SPAG8	-0.06574	0.054439
CFP	0.132508	0.054438
NEXN-AS1	0.134412	0.05443
AC010320.4	-0.17259	0.054416
GTF2H2	0.037349	0.054409
NA	0.054443	0.054404
FAN1	-0.01914	0.054373
ARMC5	0.03018	0.05436
VPS39	0.015188	0.054357
MKNK1	0.028863	0.054303
LPAR1	-0.06276	0.054294
AC087393.1	0.315868	0.054267
ADPRM	-0.03031	0.054259
ABR	0.01796	0.054257
IL17RE	-0.08962	0.054232
AL669831.4	-0.42235	0.054212
NAAA	-0.0695	0.054175
RPP25L	-0.03875	0.054167
TMSB4XP8	-0.09641	0.054164
TGS1	-0.02066	0.054157
ATPSCKMT	0.032581	0.054144
TMCO3	0.020131	0.054141
ZNF121	0.023413	0.054109
TMEM51-AS1	0.047899	0.054088
MYD88	-0.02635	0.054071
KBTBD11	-0.03649	0.054061
SNRPA	-0.01921	0.054055
AL109811.2	-0.06723	0.054028
NA	-0.33911	0.054014

LINC01541	-0.33911	0.054014
SEC63	0.015816	0.054007
DLG5	-0.02139	0.054006
TUFT1	0.033258	0.054002
NUDT15	-0.02275	0.053999
RECQL4	-0.02107	0.053975
MVD	0.025923	0.053971
RCC1	0.016806	0.053961
DDX18P5	0.321249	0.053934
TRIP11	0.019065	0.053929
TEAD1	0.015274	0.053896
AC092017.2	-0.23569	0.05389
ACSL6	0.088353	0.053881
CD14	0.108627	0.053875
RBM15-AS1	0.120781	0.053853
ARL9	-0.40346	0.053824
AL390719.1	-0.40346	0.053824
CLCN7	0.024624	0.053815
AL135902.1	-0.13495	0.053803
ELAVL4	-0.01693	0.053776
Z82243.1	-0.16532	0.053774
AC097634.1	0.221205	0.053754
CHAMP1	-0.01867	0.053738
MAPK13	-0.07056	0.053721
C1orf159	-0.03802	0.053718
AC026495.1	-0.15557	0.053716
MAPKAPK2	-0.02118	0.053714
AC131532.1	0.085749	0.053704
AC126177.5	0.394574	0.053703
PGD	-0.01441	0.053685
AL162430.1	0.339322	0.053684
EHD1	-0.02384	0.053679
HIST1H1D	0.208725	0.053641
PET117	-0.11512	0.053635
DHDH	-0.1949	0.053633
PROX1-AS1	-0.03341	0.053614
NA	-0.20305	0.053601
CCT6P2	0.36161	0.053598
PPP5C	-0.01825	0.053588
LINC00689	-0.05474	0.053581
NA	-0.03863	0.053537
AC124242.1	0.070716	0.053507
BLM	-0.03161	0.053488
ATXN7	-0.02363	0.053485
ZNF805	-0.05677	0.053481
SYNPO2	-0.01617	0.053481
ASB9	0.043471	0.053476
ACTG2	-0.06211	0.053459
KCTD18	0.029471	0.053447
AC020916.1	0.039308	0.05344

AC095056.1	-0.12319	0.053438
RF00410	-0.22243	0.053437
NONO	-0.01323	0.053434
TJP3	0.168051	0.05343
COA6-AS1	0.048585	0.053416
CAPN1	0.022407	0.053389
NA	-0.15553	0.053384
AAMP	0.015428	0.053379
AC015909.2	0.247756	0.053365
NA	0.175145	0.05335
ZNF467	-0.11624	0.053337
LYPLA2P1	0.391787	0.053321
HMGB1P27	0.391787	0.053321
PEX7	0.033591	0.053311
CUEDC2	-0.01909	0.053301
MPLKIP	0.02095	0.053279
AL021407.3	0.285866	0.05326
ATP1B2	0.051204	0.053212
ARHGEF7-AS1	0.391278	0.053209
AC093330.1	0.391278	0.053209
AL121928.1	-0.02841	0.053208
AC234775.3	0.332833	0.053196
NA	-0.02852	0.05319
TOP1MT	0.023553	0.053173
C6orf58	0.130954	0.05317
PLP2	-0.02229	0.053142
SH3GLB2	-0.0171	0.053137
NA	0.128564	0.053136
CNOT7	-0.01739	0.053125
EMC6	-0.02396	0.053119
C6orf136	0.035143	0.053093
TBC1D17	0.023644	0.05309
KIFC2	0.02119	0.053088
CALM1	-0.01855	0.053055
TTLL7	-0.02394	0.053045
RPL7P1	-0.06291	0.053039
RF00019	-0.30906	0.053035
AC099336.2	0.110046	0.052988
STK19B	-0.43222	0.052977
SIX1	0.138031	0.05297
MON1A	0.028385	0.052967
AL450384.1	-0.22537	0.052965
FAM156B	-0.03507	0.052898
SLC35E1P1	0.111892	0.052882
AC026741.1	-0.29016	0.052874
NCKAP5	-0.0463	0.052873
AC024075.2	-0.0455	0.052872
AC009690.1	-0.29563	0.052833
CHAF1B	-0.03145	0.05283
CDK5RAP3	-0.01535	0.052809

MRPL16	0.023261	0.0528
MT-TQ	0.03195	0.052787
DNAJA2	0.016954	0.052759
NA	0.032273	0.052755
TP53TG3D	0.09091	0.052739
UBQLN4	0.014952	0.052731
TLR3	0.079741	0.052714
MRRF	-0.02073	0.052695
NUDT4	0.020168	0.052666
DLGAP2	0.184987	0.052654
SCAMP1	0.017334	0.052612
OTUB2	0.048058	0.052611
AP001318.2	-0.04669	0.052608
RAC1P4	-0.33076	0.052607
AC027644.1	-0.10497	0.052597
NFIA	-0.02445	0.052561
NUP210P3	0.121206	0.052542
AC139768.1	-0.05093	0.052541
ARRDC1-AS1	-0.0257	0.052537
AP000688.1	-0.02322	0.052521
ANKRD30BL	0.49696	0.052511
MAP3K19	0.49696	0.052511
PTGDR2	0.49696	0.052511
SLC12A9-AS1	0.358336	0.052488
BCL7A	0.02399	0.052483
COMMD9	0.023053	0.052451
AC084125.4	0.041745	0.052443
TBX3	-0.02261	0.052437
HAUS1	-0.02261	0.05243
FAR1-IT1	0.259873	0.052423
AL354733.1	0.099885	0.052422
AL162258.2	0.110587	0.052421
AC022080.1	-0.39398	0.052412
MIOS	0.017856	0.052406
S1PR1	0.109074	0.052398
SLC38A5	0.14695	0.052396
DOCK6	-0.02428	0.052331
AP001972.1	-0.05861	0.052325
CWF19L2	-0.03696	0.052271
AC112491.1	0.062789	0.052255
ATL1	0.027828	0.052252
AC012055.2	0.272903	0.052246
NANOS2	-0.39175	0.052244
TMEM167A	-0.01886	0.052238
AC004253.1	-0.10288	0.052229
LINC00894	0.072575	0.052224
AP001107.4	0.043951	0.052211
SMARCA1	0.015598	0.052179
SERPIND1	0.239058	0.052164
AC105206.1	0.326453	0.052159

RABL2B	-0.01872	0.052151
AC006441.3	0.030104	0.052072
METTL23	-0.02325	0.052058
AP001781.1	0.237087	0.052056
DEXI	0.027834	0.051987
ATP5MC1P4	0.140529	0.051985
AC008543.1	0.122767	0.051976
HMGN2P46	0.086698	0.051959
ZNF843	0.14733	0.051942
NA	0.070012	0.051933
SGSM2	0.016839	0.051929
EIF2AK1	-0.01414	0.051897
VTA1	-0.02021	0.051895
AL137244.1	-0.18751	0.05189
TMEM262	-0.02629	0.051884
AC138866.2	-0.07606	0.051882
AL359258.2	0.039813	0.051881
EPB41	0.015949	0.051876
CYP27B1	0.057308	0.051818
ILK	0.016504	0.051802
UCK1	-0.01801	0.0518
NA	-0.2918	0.051793
CBX5	-0.01277	0.051736
SMCR8	0.026779	0.051704
UQCR11	0.022464	0.051694
SFTPC	0.300584	0.051692
TRIM17	0.040426	0.051688
SDHAF1	0.026352	0.051638
THYN1	0.023936	0.05161
AKAP8	-0.0182	0.051567
GGTLC4P	-0.32655	0.051553
TMEM131L	-0.02783	0.051551
AL355310.2	0.026569	0.051519
PCDHB17P	0.12849	0.051491
RGS13	-0.14036	0.051484
AP000911.2	0.236111	0.051461
CCNF	-0.02018	0.05144
C3orf67	-0.04211	0.051436
C1QTNF9B	-0.31845	0.05142
PALM	-0.02475	0.051412
PDZK1	0.080361	0.051397
AC104129.1	-0.27246	0.051366
NRBP2	0.028163	0.051335
EDRF1-AS1	0.044763	0.051326
NPY6R	-0.28787	0.051324
C19orf48	0.018146	0.05132
ZNF669	0.024903	0.051307
AC008687.4	0.13899	0.05126
TXNRD1	0.013624	0.051236
AL451050.2	0.171361	0.051232

LINC00888	0.027923	0.05123
CAPZA2	-0.0182	0.051225
GPX3	-0.03569	0.051215
REPIN1	0.017071	0.051208
COA4	0.01926	0.051184
PSMA3-AS1	-0.02085	0.051151
CERS2	-0.01409	0.051122
TMEM14B	-0.02103	0.05109
BRI3BP	0.02425	0.051083
UBN1	0.021106	0.051079
TOPBP1	-0.01686	0.051072
GMPS	-0.01839	0.051054
BNIP3	-0.01964	0.051044
ROGDI	0.025651	0.051041
ACCS	-0.0285	0.051036
YTHDF2P1	0.332863	0.051019
AL162231.2	-0.05117	0.05099
VPS13B	-0.0219	0.050987
ESS2	-0.02221	0.050987
MOSPD2	0.027837	0.050969
THNSL1	0.02861	0.050961
SEL1L3	-0.03198	0.050943
CDK19	-0.01713	0.050941
TMEM170B	-0.02372	0.050937
TMED10P2	0.145872	0.050928
PROCR	-0.07858	0.050924
ANKRD33B	-0.24122	0.050915
PACS1	-0.05018	0.050915
AC010547.4	-0.32866	0.050902
AL669831.3	0.278078	0.050883
RAB34	-0.38255	0.050877
NA	-0.38255	0.050877
TMEM131	0.020251	0.050874
TRIM32	0.024981	0.050869
AL137784.1	-0.32691	0.050869
AC097468.3	0.101292	0.050845
AL451069.3	0.273746	0.050841
CHMP5	0.018004	0.05083
SNRNP48	0.022435	0.050829
SLBP	-0.01822	0.050809
MIEF1	-0.01808	0.050803
TRAF4	-0.01785	0.050787
KIAA0319L	0.016104	0.050786
TM7SF2	0.027631	0.05078
NA	-0.30766	0.050778
CSMD2	0.058249	0.050777
MIR1289-1	0.251971	0.050773
AC073842.1	0.302506	0.050759
XKRX	-0.07177	0.05074
SNORD88A	-0.38878	0.050682

ABCC9	0.169049	0.05068
PRPF3	0.016128	0.050663
NA	0.150187	0.050638
AC012254.1	0.108117	0.050631
AC009238.3	0.144246	0.050598
MAP7D1	-0.01669	0.050584
CNOT6L	-0.02128	0.05056
MAGI2	-0.03291	0.050467
NTRK2	-0.08737	0.050422
AC015802.3	-0.05932	0.050421
NIT1	-0.01744	0.050411
AC125494.2	0.151354	0.050408
AC097376.1	0.195658	0.050398
AC007220.1	0.230938	0.050386
DTD1	-0.02006	0.050365
NEMP2	-0.0288	0.05035
ZNF528-AS1	-0.04998	0.05032
DM1-AS	0.029229	0.050312
USPL1	0.018397	0.05027
NA	0.148993	0.050264
SLC25A38	0.019037	0.050257
TNFAIP8L2	0.163336	0.050242
LYSMD4	-0.0285	0.050231
AL354707.1	-0.18345	0.050217
PNPLA3	0.076744	0.050212
CTNNB1	-0.01564	0.05021
PCDHB3	0.026613	0.050198
ANKEF1	0.051957	0.05019
PARP16	0.034991	0.050146
CHMP1B	-0.01629	0.050145
CENPB	0.01733	0.050081
ZNF816	-0.03928	0.050079
BAZ2A	-0.01824	0.050075
FSD1	-0.01794	0.050071
AL592295.1	0.274487	0.050066
BPGM	-0.02039	0.050062
AL445490.1	-0.4233	0.050055
NCOA1	0.02036	0.050041
NAA15	0.020016	0.050001
NA	-0.07011	0.049997
FBRS	0.02022	0.049985
HIST1H2APS3	0.286017	0.049982
GXYLT2	-0.04883	0.049942
AC008840.1	0.026743	0.049913
BCO1	0.433003	0.049908
RF00019	0.433003	0.049908
AC016717.1	0.433003	0.049908
AGAP4	0.087838	0.04989
ASCC3	-0.01961	0.049859
HEPHL1	-0.09974	0.049845

EFHC1	0.02281	0.049823
AC097717.1	-0.32061	0.049798
PCDH1	-0.04794	0.049787
AC060234.1	0.204805	0.049754
H2AFV	-0.01556	0.049752
KRAS	-0.01591	0.049717
AL161757.5	-0.07926	0.049669
AL513477.1	-0.07244	0.049651
NA	-0.10098	0.049618
NA	-0.30693	0.049616
AC008735.2	-0.03166	0.04959
DEPDC4	0.060698	0.049515
AC025048.4	-0.08184	0.049512
MRPL49	-0.01701	0.049473
AC010536.1	0.093412	0.049467
AC020909.3	-0.22791	0.049456
KIAA1147	-0.01609	0.049437
PSMD13	0.015275	0.049418
ZNF514	-0.02775	0.049392
ETV5	0.020868	0.049384
FLJ45513	0.353648	0.049346
TMEM225B	0.353648	0.049346
AC022400.2	-0.29654	0.049335
CHRNA9	0.179602	0.049329
RAB36	-0.03252	0.049322
NHLRC2	-0.02208	0.049287
LANCL1-AS1	-0.09732	0.049281
RF00019	-0.33013	0.049259
NA	-0.07739	0.049259
AF230666.1	-0.03214	0.049247
FAM122A	0.024027	0.049245
NA	-0.10779	0.049242
ADAM10	-0.01669	0.049233
AC091045.1	-0.03072	0.049209
FAHD2B	0.05045	0.049188
DNM1L	0.01341	0.049176
GTPBP10	-0.01842	0.049093
ASPHD2	0.034236	0.049085
C6orf203	-0.02661	0.04908
EZR-AS1	0.036357	0.049037
EIF4BP6	0.044172	0.049035
C19orf12	-0.0206	0.048992
GNB1	-0.01234	0.048965
AL451069.1	-0.14063	0.048962
EPB41L1	0.023769	0.04896
ARSJ	-0.01607	0.048956
C17orf113	-0.20996	0.048955
LTO1	0.015343	0.048952
ZRSR2	0.027407	0.048946
FGD5	-0.14582	0.048925

NBN	0.020572	0.048919
RNMT	-0.0141	0.048916
POGLUT1	0.016303	0.048898
ERO1A	0.018164	0.048894
AC244453.1	-0.1209	0.048865
AL356488.1	0.369865	0.048861
NA	-0.01469	0.048854
AC093523.1	0.251036	0.04885
STBD1	-0.04794	0.048835
KCNA3	-0.09939	0.048825
MLXIP	-0.02904	0.048809
LEF1	0.019096	0.048808
AC092814.1	-0.17552	0.048804
ATM	-0.02153	0.048745
FBXO46	0.02422	0.048647
RNU6-703P	-0.30754	0.048562
HNRNPH1	0.01433	0.048542
SP1	0.01749	0.048538
LINC02015	0.245949	0.048536
NA	-0.36476	0.048524
GAPDHP2	-0.36476	0.048524
AC226101.1	0.280386	0.048503
HAPLN2	-0.12864	0.048482
RANBP2	-0.01473	0.048424
RARRES2	-0.10521	0.048421
RN7SKP16	0.152898	0.048401
FERMT3	-0.05995	0.048398
B4GALT1	-0.02218	0.048394
PDXDC1	0.015419	0.048386
LINC02614	0.152388	0.048381
TRMU	-0.01529	0.048374
AC008149.1	-0.03684	0.048367
AC245060.2	0.116888	0.048325
ZSCAN18	-0.03377	0.048297
PEX5L	-0.09163	0.04829
TBC1D20	-0.0213	0.048251
GRIA1	-0.07809	0.048247
AC009120.3	-0.05898	0.048243
AC097358.2	-0.28492	0.048225
CCNI2	-0.07549	0.048205
FTCD	0.10872	0.048196
NA	-0.02949	0.048195
ZNF460-AS1	0.126809	0.048129
AC004839.1	0.192738	0.048122
BIRC2	0.023112	0.048121
GNA12	-0.01695	0.048118
POLRMT	-0.01647	0.048111
PSEN2	0.02087	0.048081
AC022167.1	-0.02814	0.048074
VPS37D	0.041055	0.048072

CAPG	0.143184	0.048051
TFCP2	-0.01619	0.048051
MKRN1	0.013875	0.048009
TMEM44-AS1	-0.05222	0.047995
XPNPEP1	0.015902	0.047994
SNRNP70	0.013136	0.04799
TDO2	-0.14903	0.04799
CNGA3	-0.29611	0.047981
ARHGAP24	0.080943	0.047968
TSPAN13	0.04597	0.047953
AUP1	-0.01526	0.04794
RNU1-16P	-0.23006	0.047935
SLC39A7	-0.01483	0.047915
PIP4K2C	-0.02068	0.047904
SERTM2	0.052788	0.047897
ALG1L6P	0.053284	0.047889
KDM7A-DT	-0.04448	0.047884
TMEM219	-0.01797	0.04788
TINCR	0.275271	0.047873
ELMO2	0.016362	0.047872
MIR5695	0.303224	0.047872
MAGEB2	-0.04032	0.047862
MORN1	-0.04678	0.047853
HPX	0.082938	0.047851
ZNF623	-0.01764	0.047843
RIOK3	0.019478	0.047832
RAD54L	-0.02468	0.047824
AUH	0.022796	0.047801
AC012379.2	0.141669	0.047796
CASK-AS1	-0.0586	0.047784
PRSS35	0.04711	0.047783
USP30-AS1	-0.07922	0.047761
CBR4	-0.01949	0.0477
SLX4IP	0.053076	0.047673
DNPEP	0.019645	0.047669
LOX	-0.03579	0.047641
AC109347.2	-0.30214	0.04763
LINC02616	0.065009	0.047616
SAP130	-0.02244	0.047612
SCAMP3	0.013503	0.047604
RN7SL75P	0.221681	0.047603
RGPD2	0.0517	0.047596
TMED3	-0.0156	0.047592
HNRNPAB	0.013488	0.047579
SNX21	0.022767	0.047563
DNAJC3-DT	-0.05349	0.047534
NA	-0.21518	0.047531
FARSA-AS1	-0.02644	0.047521
MIR573	-0.17283	0.047464
USH1C	-0.32389	0.047459

PSMF1	0.019479	0.047423
KRTAP5-2	0.094153	0.047412
LGR4	-0.02509	0.047395
FAAH2	0.148743	0.04739
NMD3P1	0.126299	0.047379
GABPB2	-0.04132	0.047334
AC090425.2	0.025081	0.047319
HPS6	-0.02177	0.047305
MOB3A	0.02344	0.047288
IGSF1	-0.03576	0.047288
AP000708.1	0.107919	0.047261
AFF3	0.043663	0.047241
FBXO44	0.026035	0.047229
HIVEP3	-0.04139	0.047225
LINC-PINT	0.033648	0.047223
DEGS1	-0.01939	0.047222
AC007032.1	0.110839	0.047221
PKD1P6	0.028003	0.047176
C8orf58	0.034798	0.047139
UAP1L1	0.023207	0.04711
HSPA9P1	0.274735	0.047059
DPYSL3	-0.01689	0.047015
MEIOB	-0.08331	0.047011
POLR1D	0.014001	0.046987
NCAPG2	0.016792	0.046975
AL157400.3	0.171972	0.046941
AC007938.2	0.076528	0.046915
PRDM8	0.021413	0.046908
CHML	0.020319	0.046877
AHCYL2	-0.01961	0.046864
FGF7P3	0.07826	0.046849
AL360227.1	-0.30245	0.04683
CCDC34	-0.02048	0.046817
DNAJC7	-0.01546	0.046817
SLC26A10	0.020804	0.046814
RABEPK	-0.01915	0.046779
AC087854.1	0.05966	0.046744
GABRA3	0.031505	0.046744
VCAN	-0.01704	0.046688
DCTN6	0.021295	0.046665
ODCP	-0.15626	0.046661
RMND5B	-0.01469	0.046641
ZNF439	0.031506	0.046638
DEPTOR	-0.07192	0.046623
DEPP1	0.047985	0.04662
PSMD6	-0.01699	0.046591
AFAP1-AS1	0.032256	0.046588
NDUFAF7	-0.01714	0.046583
MIR548AA1	-0.38523	0.046572
NA	-0.38523	0.046572

CSE1L-AS1	-0.38523	0.046572
AC022558.1	-0.38523	0.046572
AP000892.1	-0.24609	0.046559
DGKA	-0.02473	0.046557
FDXACB1	-0.05033	0.046528
RPL14P3	0.197235	0.046515
SPHK2	0.019824	0.046511
DNAJC10	0.015903	0.046483
AL109809.1	0.207559	0.04642
AC119751.3	-0.21028	0.046409
KIF9	-0.02525	0.046372
COG1	0.01493	0.046368
NA	-0.02349	0.046341
NA	-0.12301	0.046339
RPS19P3	-0.2141	0.046325
PPP2R3C	0.021906	0.046321
ZNF260	-0.01799	0.046273
LAMTOR3	-0.0155	0.046262
ZNF296	0.127161	0.046248
WDR43	0.016186	0.046225
RPS13P2	0.118017	0.046222
AF131215.7	-0.18827	0.046175
NA	-0.23016	0.046161
NA	-0.23016	0.046161
GTF2IRD1P1	0.022718	0.04616
AC091860.2	-0.15999	0.046155
RPA2	0.018981	0.04615
VCPKMT	-0.02617	0.046136
AP4B1-AS1	-0.034	0.046123
GRIK1	-0.12116	0.046069
SFXN4	-0.0198	0.046054
NEK2	0.019369	0.045992
DCDC2B	0.05648	0.045968
TAF7L	0.094782	0.045957
ZNF569	0.025187	0.045954
PGM1	0.016196	0.045933
LMLN-AS1	-0.1043	0.04593
FTH1P10	-0.18783	0.045923
GAS8-AS1	-0.12449	0.045902
MIR3682	0.110064	0.045872
AC020915.2	0.263214	0.045846
AC100827.3	0.045303	0.045845
EDEM3	-0.01639	0.04583
NA	-0.24725	0.045818
FANCB	-0.04773	0.045813
PSMA4	-0.01663	0.045812
FTH1P4	-0.31358	0.04581
AC083843.1	-0.31358	0.04581
DMAC1	0.020599	0.045805
UFM1	0.014475	0.045778

AC091729.1	-0.37927	0.045773
AP003119.3	-0.03688	0.045765
C10orf88	-0.02148	0.045762
PPP1R12C	-0.01548	0.045751
MBP	0.175916	0.045677
AP002812.2	0.058838	0.045641
TSC22D1	0.015163	0.045628
AC022382.1	0.057903	0.045626
SAFB	-0.01431	0.045624
TMUB2	0.016176	0.045603
RAD51B	-0.04149	0.045594
COX6B2	0.202189	0.045589
BX649601.1	-0.21103	0.045581
GDPD1	0.028863	0.045569
NA	-0.18276	0.045566
LINC00839	0.017319	0.045566
ATP5PF	-0.01772	0.045563
HGS	0.014844	0.045561
ZNF613	0.041497	0.045533
TIFA	0.025454	0.045522
AC007255.1	-0.1722	0.045516
LMO2	0.063036	0.045505
GSC	0.107746	0.045504
NA	-0.03466	0.045495
PCDHB2	-0.11746	0.045467
NOL7	-0.0147	0.045462
LINC02166	-0.19434	0.045441
AASDH	-0.02183	0.045437
AC008555.1	0.053933	0.045436
DZANK1	-0.03154	0.045436
UBD	-0.19703	0.045431
NA	0.099605	0.04543
TCF25	-0.01556	0.045428
RPLP1P13	-0.26301	0.045426
HPCAL1	0.017494	0.045392
AC068279.1	-0.09794	0.045389
NUP155	0.017483	0.045383
LMBRD1	0.021278	0.045377
LYRM7	0.022764	0.045371
AC079193.2	0.13811	0.045369
VMO1	0.146907	0.045326
AC021188.1	-0.08616	0.045317
ZNF358	-0.0181	0.045291
AL078645.1	0.30917	0.045281
RALGPS2	0.026402	0.045281
EMILIN1	-0.0171	0.045236
USP33	0.014816	0.045218
LINC01715	-0.16273	0.045211
AP001107.3	-0.21776	0.045211
PLB1	0.063735	0.045202

SBK1	-0.02212	0.045191
SEC13	-0.0161	0.045173
RGS11	0.029075	0.045165
SNX16	0.024142	0.045121
AC090587.1	-0.15295	0.045112
NA	-0.13133	0.04511
AC027309.1	0.059406	0.045101
LPCAT4	0.016703	0.045099
PGS1	0.015887	0.045082
MTCO3P43	-0.23493	0.045045
RPL29P12	-0.33765	0.045042
CELF3	-0.02297	0.045041
HDAC2	0.015727	0.044987
ANAPC4	-0.01827	0.044985
DACT3-AS1	0.107886	0.044982
BNIP3P5	-0.2852	0.044969
AL021707.1	-0.07172	0.044933
LINC02328	-0.19994	0.044926
ETNK1	-0.01373	0.044916
NABP1	0.034565	0.04489
NA	-0.12393	0.044879
CCDC43	-0.0186	0.044855
NEUROD1	0.057241	0.044853
ABHD3	-0.03812	0.04481
TMEM161A	-0.02293	0.044799
RRAGA	0.013924	0.044796
DHX9P1	0.271346	0.044756
AC134043.1	0.271346	0.044756
MAPK3	-0.01896	0.044752
EFCAB9	0.209138	0.044724
NPHP3-AS1	-0.04894	0.0447
CDC37L1	0.01958	0.044695
LINC00882	0.096857	0.044647
AC026765.2	-0.27467	0.044616
DYM	0.021667	0.044615
RRM1-AS1	0.028895	0.044615
PDCD4	0.014978	0.044572
ATP5PO	-0.0203	0.04457
AP000759.1	-0.04859	0.044563
SHKBP1	0.022359	0.044559
ZNF440	-0.02775	0.044558
LINC00514	0.141271	0.044534
C5orf56	0.065695	0.044526
PAPOLG	-0.01969	0.044522
SEPT4	-0.0542	0.044496
SDC4	-0.0197	0.044482
PTTG1IP	0.01159	0.044454
ZBTB48	0.029771	0.044438
ACSBG2	-0.12226	0.044431
RAB8A	0.015856	0.044421

TMED9	0.014152	0.044413
NA	-0.02876	0.044397
AL121723.1	-0.31232	0.044386
RANBP10	0.018763	0.04438
HOOK2	0.021515	0.044376
LACTB2	-0.03357	0.044374
AC092718.1	0.176525	0.04435
TBC1D10B	-0.01672	0.04434
SLC2A4	-0.07662	0.044325
MLLT10P1	-0.13187	0.044296
LINC01563	-0.2002	0.04426
CDC20	-0.0164	0.044241
AC008277.1	-0.04235	0.044214
EPHX2	-0.02307	0.044213
TOMM20	-0.01145	0.044208
AC006333.2	0.027482	0.044205
AL355385.1	0.043212	0.044168
AC010761.3	0.077552	0.044164
GPC4	-0.06269	0.044107
AL035530.2	0.056736	0.044103
AC145285.4	-0.2798	0.044044
AL589674.1	-0.2798	0.044044
PSMB8-AS1	-0.04349	0.044012
NA	-0.26273	0.04401
RPL21P16	0.050735	0.043971
AC008878.4	-0.03172	0.043971
AL021937.2	0.300246	0.043961
SMC2	-0.01685	0.043946
TDRG1	-0.09345	0.043931
AP002784.2	0.11827	0.043929
AL133517.1	0.069732	0.043914
PRDX3P2	-0.16672	0.043911
RNU4-5P	0.033049	0.04391
YBX1P2	0.14763	0.043833
DSEL	0.019644	0.043832
SPCS2P4	-0.11296	0.043805
BMPR1AP1	0.336293	0.043782
ZNF460	0.038885	0.043772
CD151	-0.0138	0.043748
CCAR2	-0.01368	0.043734
PCDHB7	-0.07877	0.043692
KNL1	0.019769	0.043681
LINC02495	0.048307	0.043618
PACRGL	-0.02213	0.043615
RNF141	0.020138	0.043611
BX927359.1	-0.04152	0.043598
IKZF5	0.019614	0.043594
TTC39A	-0.03515	0.043589
YKT6	0.011489	0.043583
TBILA	-0.18901	0.043554

AC091965.4	0.201595	0.043551
ZNF416	-0.03581	0.043543
SPR	-0.02097	0.043535
TSPYL1	-0.01582	0.043521
SHISA9	0.162893	0.043507
GMEB1	-0.02829	0.043439
SLC26A11	-0.02165	0.043432
MTND4P26	0.333358	0.043399
SCAMP5	0.014086	0.043386
AC011466.1	-0.05948	0.043352
AC098869.2	0.275786	0.043349
ZNF41	0.020663	0.043339
RNF166	-0.02241	0.043336
LAMA5-AS1	-0.0775	0.043315
LINC00242	0.11136	0.043296
RPL32P3	-0.02108	0.043273
AC109361.1	-0.10947	0.043264
TMCO1	0.014686	0.043257
ACTA2-AS1	-0.02737	0.043252
GALNT16	0.04611	0.043229
MRPS18C	-0.01809	0.043208
GPR75	-0.0362	0.043203
HEATR6	-0.01421	0.043169
AL138789.1	0.040048	0.043168
C15orf32	-0.23459	0.043135
ZMYND12	0.081385	0.043114
CRYZ	0.019213	0.043111
NA	0.23826	0.043072
AC003965.1	0.196476	0.043036
SYT11	-0.01068	0.042997
AL139353.1	0.200982	0.042986
AC126323.1	0.344324	0.042972
DYNLL1P1	-0.24627	0.042968
NA	-0.19338	0.042967
METTL9	-0.01295	0.042961
BCL6B	-0.10931	0.042926
AC104986.2	0.091697	0.042924
AC008378.1	-0.09052	0.042901
RRAS	-0.02757	0.042896
LPCAT3	0.019362	0.042882
CDK18	-0.03688	0.042873
TRAM2-AS1	0.024172	0.042872
IZUMO4	0.059162	0.042868
RPL36P4	0.123146	0.042866
ADGRB2	0.021125	0.042865
NPM1P6	-0.12344	0.04286
RGS9BP	0.15019	0.042833
SNORA22B	0.183792	0.042827
AC010733.1	0.225961	0.042827
ACTG1P10	0.080292	0.042771

C10orf105	-0.20441	0.0427
AL359881.1	-0.19766	0.042695
FAM92A	-0.01944	0.042694
NA	0.239904	0.042692
LSM14A	0.012216	0.04269
VRK1	0.025384	0.042679
TSFM	-0.02023	0.042668
SPEF2	-0.04414	0.042663
UCHL5	-0.01448	0.042657
CXCR5	-0.03911	0.042647
PI4KAP2	0.023284	0.042636
TMEM39A	0.015863	0.042627
PROZ	0.086438	0.042612
AL109613.1	-0.07763	0.042584
ADGRL1	-0.0196	0.042533
CENPBD1	0.024656	0.042498
LYRM9	-0.06564	0.042497
MRPL24	0.014978	0.042468
RPL12P15	0.214004	0.042461
SLC25A16	0.022654	0.042459
RAVER2	0.018583	0.042442
TMEM177	0.021779	0.04244
KCNH1	0.035839	0.042433
LHX9	0.18999	0.042429
NA	0.127846	0.042422
PBX3	-0.01711	0.042417
HIST1H2AG	-0.02856	0.042415
BCL2L2	0.017428	0.042411
TAS2R6P	-0.19229	0.04241
NDUFAF2	0.022533	0.042403
F10	0.032065	0.042396
AL591767.3	0.224766	0.04238
LINC02082	0.113643	0.04235
AL161668.3	-0.02162	0.042319
IL4I1	-0.09268	0.042298
CIAO1	-0.01179	0.042289
TAX1BP1	-0.01201	0.04226
AL158829.1	0.278439	0.042246
ETV5-AS1	0.142382	0.042239
RPL36AL	0.015571	0.042221
PPM1E	-0.02492	0.042206
NA	0.157443	0.04219
TMTC3	-0.0179	0.042167
CRTAC1	0.035124	0.042056
AC105389.2	-0.15485	0.042019
PTGER4P2	0.139229	0.042014
ECI1	0.016546	0.041994
NA	0.02143	0.041976
TPT1P9	-0.10106	0.041971
AURKA	0.021938	0.041939

BSDC1	0.012383	0.041907
SUMO2P1	0.105881	0.041886
KHDC4	-0.01229	0.041884
GSK3A	-0.01502	0.04187
RTN3	-0.01312	0.04183
SUB1	0.014914	0.041798
AL590139.1	-0.12888	0.041784
AC090617.3	-0.01834	0.041783
RNU6-126P	0.127638	0.041766
CGAS	0.12378	0.041699
SPDYE17	-0.22195	0.041667
SLC4A3	-0.03185	0.041626
MSH2	0.015528	0.041614
LINC01979	0.146618	0.041612
CNGB3	-0.13804	0.041606
ADPGK	0.01429	0.041602
PRKAG2-AS1	-0.0363	0.04159
RNU6-395P	0.043857	0.041587
SMIM11A	0.022069	0.041571
ANO6	-0.01309	0.04157
SNHG1	0.012794	0.041552
SUN2	-0.02116	0.041545
AC008760.1	0.063788	0.04154
MAP3K4	-0.01681	0.041538
GTF2H2C	-0.02037	0.041533
LINC01068	0.182756	0.041527
AC006254.2	0.222704	0.041499
PRDM5	-0.02357	0.041486
CYP51A1-AS1	0.082944	0.041474
AC084782.2	-0.2149	0.04145
LCN15	-0.16506	0.041445
IGLVIVOR22-1	0.211869	0.041439
SETD4	0.01666	0.041437
NA	0.111705	0.041416
POLR2B	-0.0116	0.041405
NELL2	0.040302	0.041376
VASH1-AS1	0.019188	0.041371
CIR1	-0.01627	0.04136
AC113382.1	0.043251	0.0413
ZDHHC11B	0.057348	0.041296
SIRPA	0.044001	0.041285
C12orf73	0.020279	0.041281
AC009480.1	0.084852	0.041266
NA	-0.03449	0.04125
HEXIM2	0.03066	0.041243
FANCD2	-0.02213	0.041239
ZNF770	-0.01507	0.04122
INTS6L	0.023601	0.041213
AC027237.2	-0.26187	0.041172
DCAF13P3	-0.26187	0.041172

AP000763.3	-0.03283	0.041171
ZNF37A	0.01701	0.041159
KIF26B-AS1	-0.28081	0.041131
CYP2A7	0.287049	0.041116
AL359091.5	0.155144	0.041111
LINC02067	-0.05802	0.041092
SMARCE1	0.013972	0.041051
RFTN2	-0.069	0.041044
ABHD16A	-0.0759	0.041043
AC233992.2	0.092806	0.041009
MIR3153	-0.14941	0.040982
PARP12	-0.14531	0.040977
NA	-0.09579	0.040973
PPP3CB-AS1	-0.02597	0.040965
TRMT1L	-0.01791	0.040957
CAMTA2	0.020899	0.040946
CCDC157	0.019879	0.040937
DIAPH2	0.02809	0.040911
PLAA	-0.01468	0.040895
PNMA8B	0.044334	0.040888
PHC1	0.012842	0.040817
TXN	0.016279	0.040811
NPC1	-0.0189	0.0408
ZCCHC7	-0.01776	0.040799
GRAMD4	0.017642	0.040781
DBIL5P2	-0.33439	0.040773
AC142381.2	-0.33439	0.040773
TMPPE	-0.048	0.040771
UTS2	0.076793	0.040757
FIBP	-0.01432	0.040744
PSORS1C1	0.284351	0.040727
AL158055.1	-0.17665	0.040707
IDH3G	-0.0202	0.040702
DDOST	0.012392	0.040701
WDR18	0.014819	0.040692
RNU2-28P	0.200604	0.040667
AL591686.2	0.204826	0.040656
CBLB	0.018671	0.040651
DEFB131E	-0.15466	0.04065
SCRN3	0.014203	0.04065
CEP83	-0.01891	0.040643
SLC51B	0.207443	0.04064
SYCP3	0.085603	0.040611
SNORD63B	0.100663	0.040601
ZBED1	-0.01648	0.04059
PARD6B	0.040698	0.040563
MT-RNR2	-0.01285	0.040546
TSPEAR-AS1	-0.0392	0.040538
C4orf46	-0.0151	0.040536
HACD3	0.012383	0.040534

GPALPP1	-0.01581	0.040486
DLD	-0.01278	0.040483
SSX2IP	0.019264	0.040479
BZW1	0.013227	0.040453
NA	-0.17536	0.040452
PLAC9	-0.11032	0.04041
AC010504.1	-0.1567	0.040398
NDUFAF4	0.019962	0.040368
SPRN	-0.01967	0.040362
NA	0.011486	0.040362
COMMD10	-0.02045	0.040314
LINC01411	-0.23274	0.040313
GARNL3	-0.01669	0.040294
FAM228A	-0.22321	0.04029
TMPRSS11D	-0.15523	0.040289
PKDREJ	0.0733	0.040287
AC069120.1	-0.16261	0.040274
ADO	-0.01275	0.040267
RNU6-516P	0.153262	0.040219
NUTM1	0.168682	0.040209
PLEKHN1	0.118174	0.040196
NA	-0.16976	0.040183
LINC01534	0.072698	0.040182
PRMT6	-0.01392	0.040167
AC092115.1	0.086824	0.040163
HMGCL	0.020583	0.040158
TRIM46	-0.01811	0.040149
BMP6	0.198828	0.040148
MAP3K14-AS1	-0.04534	0.040147
NBEAL2	0.020279	0.040147
NA	0.024421	0.040147
LINC01554	-0.23799	0.040132
OSGIN1	0.048584	0.040119
WAPL	0.012691	0.040106
AMIGO1	0.031683	0.04007
NA	-0.01455	0.040045
AJM1	0.050022	0.040027
DARS2	-0.01403	0.04001
ALDH7A1P1	-0.23405	0.040005
PLXNB3	-0.03009	0.039995
ZNF117	-0.01326	0.039987
METTL14	-0.01568	0.039975
AL356299.2	-0.15169	0.039968
PRKCZ-AS1	0.045778	0.039924
AC093010.3	-0.04145	0.039916
LINC01347	0.097163	0.0399
VWA8	-0.01629	0.039897
KCNN4	0.133065	0.039896
EIF3FP3	-0.09645	0.039856
RPL4P2	0.277832	0.039836

CACNA1G-AS1	0.182826	0.03982
CCDC85B	0.015632	0.039819
NA	-0.3339	0.039768
BTF3L4P1	-0.3339	0.039768
AC092747.2	-0.3339	0.039768
TNFRSF10A	0.051834	0.03973
DNAJB8	0.193493	0.039729
HSPBP1	-0.01513	0.039703
FMNL1	-0.06471	0.03969
EXOC1	-0.01444	0.03966
MT-RNR1	0.014226	0.03966
BICD1	0.014112	0.039643
FOCAD	0.014538	0.039634
AC093382.1	0.234479	0.039601
SLC25A51	-0.02246	0.039584
AP4M1	-0.0143	0.039581
TXNDC12	0.013301	0.039576
DGLUCY	-0.02138	0.039556
RXYLT1-AS1	0.041883	0.039551
CRIPT	0.014958	0.039526
DCUN1D1	-0.01512	0.039497
LINC01775	0.124745	0.039469
AC005785.1	0.024794	0.039457
NA	-0.02929	0.039428
GACAT2	0.232523	0.039421
DVL2	0.014592	0.039417
GATD1	-0.01685	0.039411
HNRNPA1P26	0.230161	0.039409
NAXD	-0.01378	0.039408
INTS7	0.014568	0.039401
TESC-AS1	0.247124	0.039387
AL450998.3	0.024061	0.039384
NUCKS1	0.011584	0.039375
RNU6-1024P	-0.33084	0.039368
AC048382.2	-0.33084	0.039368
IL4R	0.025322	0.039359
PNLDC1	-0.20708	0.039358
ZNF106	-0.01324	0.039356
AC008937.1	0.068034	0.039349
AC084125.1	0.037359	0.039301
LINC01121	0.154169	0.039298
RPL24P4	-0.06516	0.039275
RNU6-1262P	0.218045	0.039269
AC012354.1	-0.01934	0.039267
TKFC	-0.0135	0.039239
NOL4L	-0.01781	0.039225
SCARF2	0.016755	0.03922
AL671986.1	-0.20078	0.039195
PKD1	-0.01446	0.039182
STAC3	-0.05354	0.039171

RPS6KA5	-0.02303	0.039131
NECAB3	0.018466	0.039119
MARF1	-0.01416	0.039108
SOCS5P2	0.327414	0.039054
NA	0.327414	0.039054
BAG4	0.014856	0.039037
FOXN3	0.013033	0.039025
HSD3BP5	-0.07596	0.039001
PAQR7	0.02227	0.039001
Z68871.1	0.052462	0.038996
PDE6C	-0.14154	0.038985
PSMD12	0.012795	0.038969
EIF4A2	0.010699	0.038916
SSPO	0.067238	0.038892
AC010468.1	-0.18302	0.038811
LINC01918	0.063719	0.0388
NHP2P1	0.125844	0.038799
AL157392.3	-0.01641	0.038788
ARPC4	-0.01204	0.038777
POLR3K	-0.01668	0.038774
RIMBP2	-0.01255	0.03876
FAM50A	-0.01412	0.038758
MATN3	-0.02713	0.038754
AC025031.2	-0.18086	0.038749
FAXDC2	-0.01922	0.038701
NR1I2	0.220459	0.038694
LINC00634	0.061441	0.038685
KCNIP2	-0.03531	0.038672
EBNA1BP2	0.011522	0.038667
TUBA1B	0.009747	0.038638
DLX6-AS1	-0.02464	0.038626
LAMC2	-0.14433	0.038608
PCDHGA12	-0.15481	0.038592
AGO2	-0.02288	0.038592
IRAK1	0.013501	0.038574
ZNF275	-0.01846	0.038573
ZNF322P1	0.06749	0.038552
FLRT1	-0.04886	0.038514
CENPW	0.021036	0.038509
AC040162.1	-0.05015	0.038505
HTR7	0.180948	0.038505
ZNF708	0.020507	0.038474
EMP3	-0.04448	0.038471
RN7SL263P	-0.18363	0.038468
ZBTB45	0.019254	0.038455
KANSL1L	0.017054	0.03845
RASAL2	0.023695	0.03844
NRAV	0.028589	0.038392
AP001092.1	-0.14054	0.038388
GAU1	0.126431	0.038374

BBIP1	-0.01477	0.038365
LINC01569	0.088834	0.038363
PARP6	0.013329	0.038359
BPIFB9P	-0.2036	0.038331
STS	0.02121	0.038318
AL121917.1	-0.07767	0.038278
TXNDC15	0.015347	0.038276
IGHV3-33	0.209539	0.038272
OR13H1	0.360613	0.038265
BOLA2P3	0.360613	0.038265
SNORD71	0.360613	0.038265
LINC01940	0.360613	0.038265
AP001372.4	0.360613	0.038265
ZNF169	-0.02696	0.038258
SPRTN	0.017687	0.038252
AC092720.1	0.113222	0.038222
ZMYM6	-0.02499	0.038215
SREBF1	-0.01927	0.038208
SNAP25-AS1	0.053251	0.038207
TBL2	-0.01733	0.038183
FKBP1C	0.084891	0.038162
PPP1R12A	0.012627	0.038075
CPPED1	0.017621	0.038062
AL606534.1	-0.02048	0.038049
ENTPD8	0.169258	0.038043
MAP7D2	-0.03956	0.038028
SMIM26	0.017961	0.037985
AC004466.1	0.027813	0.037958
SCRG1	-0.08055	0.037939
GMPPA	-0.01769	0.037909
MCCC1-AS1	0.042673	0.037907
MARCH3	-0.04468	0.037901
CDK16	-0.01362	0.03789
C9orf64	0.019375	0.037864
AC025257.1	-0.01616	0.037862
NA	-0.07515	0.037856
AC002076.1	-0.18006	0.037846
AL596094.1	-0.06115	0.037841
AC254562.2	-0.16416	0.037802
THUMPD3-AS1	-0.01635	0.037777
AC089984.2	0.289984	0.037747
NA	0.289984	0.037747
PROM1	0.029612	0.037723
TMEM126A	-0.01991	0.037722
AL358075.2	-0.12909	0.037721
PRXL2C	-0.02397	0.037721
ZNF334	-0.01995	0.037721
LINC02019	-0.16159	0.03772
SDF2	0.015477	0.037706
PFN4	0.06475	0.037699

TTC37	-0.01251	0.03768
SNX3	0.012793	0.037661
AL158834.2	0.029531	0.037647
NRBF2	-0.01851	0.037647
RPS17	-0.07505	0.037645
CIT	0.016276	0.037634
CATSPERD	-0.18345	0.03763
SUGT1P4-STRA6LP	0.075308	0.037628
AC011369.1	-0.17648	0.037606
ATAD3A	-0.01661	0.037602
NAV3	0.05656	0.037599
HMGN2P5	0.044496	0.037587
PGRMC2	0.013831	0.03758
ERVMER34-1	0.083401	0.037537
TUBAP2	0.109727	0.037532
TRIM35	0.016299	0.037509
DUXAP7	-0.28146	0.037494
DEPDC5	0.019703	0.037489
LINC00271	-0.05287	0.037472
UBTD2	0.01417	0.037448
GMEB2	0.020993	0.037434
THBS3	0.013263	0.037426
DYDC2	-0.08593	0.037421
AC005534.1	0.083965	0.037413
WSB2	0.011305	0.037411
ZNF426	0.021651	0.037374
SRBD1	-0.01606	0.03736
CDADC1	0.027831	0.037357
DDX49	0.013977	0.037356
STAC	-0.01261	0.03734
OSTF1	0.068938	0.037337
HSPE1P3	-0.19801	0.037329
THOC2	-0.01226	0.037326
AC010503.2	0.202738	0.037312
RPL9P29	-0.0678	0.037309
RD3	0.021788	0.037291
DDX60L	0.025646	0.037269
DXO	0.01696	0.037204
ARHGAP12	0.015983	0.037188
AC087752.3	-0.06875	0.037186
MALSU1	0.014045	0.037174
AC025181.2	0.06004	0.037164
LINC01678	0.168814	0.037163
AC005261.1	0.018719	0.037131
NA	0.018606	0.037089
AC138392.1	0.099682	0.037078
KIAA1217	-0.02448	0.037048
AC020659.1	0.067812	0.037027
PTPRD-AS1	0.28426	0.037
AC026688.1	0.28426	0.037

MYHAS	0.28426	0.037
FGF2	-0.0459	0.036986
AC034102.4	-0.26873	0.036973
DALRD3	0.012646	0.036963
ADD3	-0.01369	0.03693
SLC41A2	0.030418	0.036929
AF196972.1	-0.21991	0.036879
VSIG8	-0.21991	0.036879
NA	-0.01452	0.036869
AC145124.1	0.061103	0.036861
PUS1	0.017453	0.036856
AC079354.2	0.27393	0.036844
PKD1P5	-0.03734	0.036842
ZNF101	0.022044	0.036839
SYVN1	0.01768	0.036807
AC022154.1	-0.07986	0.03679
AC003688.2	-0.13862	0.036787
VN1R82P	0.100273	0.036777
ETFDH	-0.01912	0.036768
DTNB	-0.01991	0.036763
AC008655.2	0.220084	0.036714
TMEM9B-AS1	0.041151	0.036697
FZD1	-0.01749	0.036667
PLPP6	-0.02019	0.036624
KMT5A	-0.0136	0.03659
DCDC2	0.152536	0.036588
ZNF432	0.022576	0.036551
AC084757.3	0.096572	0.036536
SSBP1	-0.01336	0.036518
SUCLG2	-0.01696	0.036517
STK39	-0.01457	0.036514
TNKS	-0.01169	0.036512
AL159169.3	-0.18123	0.036477
CAPZA1	0.012993	0.036469
SLC5A5	-0.07563	0.03642
ALG13	-0.01551	0.036407
AL354824.2	-0.19416	0.036386
ZNF783	0.018986	0.036377
TASOR2	-0.01227	0.036376
ZXDB	-0.024	0.036351
AP003392.1	-0.07881	0.036336
NAA40	-0.01358	0.036297
NAT10	-0.01182	0.036293
DYNC1I2	0.013354	0.036282
SLC43A1	0.026945	0.03626
ARL2BPP10	0.088087	0.036246
AC008507.1	0.068042	0.036243
CCDC71	0.015661	0.036217
TET3	-0.01664	0.0362
ZNF840P	0.130922	0.036192

GHDC	-0.02002	0.036181
SNORA23	-0.27223	0.036168
NUTM2G	-0.0594	0.036117
CPTP	-0.02648	0.036114
AC087190.1	0.033196	0.036106
LINC01287	-0.19616	0.036082
RHBDD1	0.017921	0.036066
ERGIC2	-0.01427	0.036032
AGBL5-AS1	0.230934	0.036007
VWA7	-0.13779	0.036006
AC027097.1	-0.0442	0.036
RNU6-1019P	0.274064	0.035992
MIR1302-8	0.274064	0.035992
CYCSP52	0.274064	0.035992
KPNA2P1	0.274064	0.035992
RN7SL397P	0.274064	0.035992
AC011465.1	0.274064	0.035992
SLC35B3	0.01827	0.035985
BTBD11	-0.02994	0.035982
AC110373.1	0.124708	0.035979
ILF3	-0.01047	0.035964
YIF1A	0.012979	0.035956
APRT	-0.01283	0.03595
CPB2-AS1	-0.09057	0.035927
HNRNPKP3	-0.1214	0.035907
CASC2	0.032711	0.0359
OGFOD3	0.0139	0.035895
GTF2IRD1	-0.01533	0.035887
CNTN2	-0.05844	0.035886
TIGD6	0.021036	0.035882
AL078644.2	0.076513	0.035863
LRCH4	-0.01493	0.035858
PRDM2	0.015552	0.035842
AC004951.1	0.03681	0.035816
NA	0.050751	0.03577
ARHGAP28	-0.02481	0.035762
RAB18	0.012634	0.035761
ZNF285	-0.03344	0.035756
B4GAT1	0.013983	0.035738
AC005034.2	-0.16455	0.03569
AC091059.1	-0.05366	0.035689
AC132872.1	-0.02981	0.035686
AC009511.1	0.029833	0.035665
IPO7	0.010303	0.035659
CALCRL	0.066918	0.035623
HOXD-AS2	-0.0279	0.035612
EIF2B2	0.012326	0.035597
PFAS	-0.01457	0.035596
NDUFA12	0.014928	0.035585
GOSR2	-0.01134	0.035572

ACTG1P24	0.162199	0.035569
AC002116.2	0.029453	0.035566
ALDH4A1	0.033827	0.035556
TMEM218	-0.02319	0.035542
ASXL1	0.010165	0.035541
C1orf74	-0.02185	0.035527
CTDSPL	0.016391	0.035513
AC093249.2	-0.08092	0.035508
AP003071.3	-0.20465	0.035486
AC005726.4	-0.02974	0.03548
PAK5	-0.04332	0.035468
MSH6	0.010244	0.035462
RF00019	-0.13467	0.035374
A2MP1	-0.10384	0.035373
RAB3GAP1	0.013338	0.035343
SNRPCP2	-0.24399	0.035336
BBS4	-0.01508	0.035317
UBR5-AS1	0.032523	0.035311
PREP	-0.01322	0.035279
NDUFC1	-0.01505	0.03527
FAM157A	-0.05588	0.035265
AL596202.1	0.024847	0.035263
AC090589.3	-0.07657	0.035255
COMT	0.013282	0.035252
Z97986.1	-0.02406	0.035155
POLDIP2	-0.0102	0.035091
WDR27	0.011969	0.03507
SETMAR	0.015599	0.035064
MOSPD3	0.017169	0.035048
ZNF316	-0.01512	0.035021
TMEM171	0.103329	0.034902
LRRC8A	-0.01171	0.034875
TFRC	-0.01307	0.034865
LIN52	-0.02039	0.034858
AL451062.1	-0.21987	0.034847
GAL3ST3	0.026067	0.034808
CDH18	0.129654	0.034785
LINC00365	0.264745	0.034761
AC090686.1	0.264745	0.034761
AP000904.1	0.264745	0.034761
HNRNPU	-0.0086	0.034743
PLPP3	0.020392	0.034736
ZBTB21	0.014275	0.034733
RPL26P35	0.221007	0.034715
SFRP1	-0.0334	0.0347
ADGRD2	0.196163	0.034699
RPL35P1	0.183472	0.034688
WNT9A	-0.17016	0.034673
ROPN1B	-0.06888	0.03467
CEPT1	-0.01518	0.034668

SLC25A37	0.014903	0.034653
NA	-0.18228	0.034646
PSTPIP2	0.034138	0.034638
NUDCD2	0.013456	0.034637
GFRA2	-0.02627	0.034575
AC021016.2	-0.043	0.034558
HADH	0.013308	0.034557
ATP6V0B	-0.01127	0.034553
RHOV	0.142106	0.034525
AC055876.2	-0.08058	0.034513
AC010336.4	-0.02381	0.034488
PCDHAC2	0.017036	0.034458
NA	0.122486	0.034455
AC093864.1	0.087526	0.034426
LINC01973	0.224536	0.034414
CUL2	0.013363	0.034402
TRAPPC13	0.015484	0.034373
SLC31A2	-0.02553	0.034372
AP001178.2	0.073823	0.034348
CCDC138	-0.01772	0.034336
BRWD3	0.013147	0.034297
AC122710.2	0.106825	0.034281
AC125611.4	-0.02268	0.034263
SMCHD1	-0.0112	0.034261
SYT1	-0.01203	0.034178
SEMA4C	-0.01368	0.034146
KIF5A	-0.01641	0.034113
SNCG	0.195503	0.03411
SLC24A5	-0.01691	0.034095
SSR4P1	-0.06682	0.034087
TSPYL5	-0.01065	0.034072
KIF1B	-0.00979	0.034055
AC010132.4	-0.05174	0.034025
ITPKA	-0.05781	0.033998
AL162411.1	0.113208	0.033964
STK17A	0.01462	0.033943
RPL23AP87	-0.25535	0.033929
AP005131.6	-0.10165	0.033896
AC234781.1	0.079373	0.03388
PML	-0.01817	0.033876
NA	-0.03936	0.033872
AC012313.3	-0.03401	0.033859
ATP1B3	0.0119	0.033857
ATP1B3-AS1	0.057915	0.033846
ARG2	0.013019	0.03384
SERPINB1	0.02342	0.033801
LAPTM4A	-0.01025	0.033799
FBLN2	-0.01745	0.033783
AC137932.1	-0.05968	0.03378
PPM1N	-0.02358	0.033778

AC092821.1	0.047609	0.033748
CCDC27	0.157814	0.033744
HTR7P1	-0.04663	0.033739
HAUS7	-0.04495	0.033737
RFC4	0.015957	0.033722
UHRF1BP1L	0.015533	0.03372
PTEN	-0.01011	0.033708
HTATSF1P2	0.098688	0.033704
VPS35	-0.01039	0.033703
FAM3C	0.012452	0.033692
NA	0.041213	0.033679
INPP5B	0.017693	0.033674
IPO5	-0.01055	0.033665
KLF12	-0.01347	0.033632
GDAP1	0.012333	0.033622
KCNMB1	0.139128	0.033613
INHCAP	0.11775	0.033609
VPS4B	-0.01214	0.033601
RPTOR	0.015135	0.033592
C6orf52	-0.04005	0.033579
NA	-0.04007	0.033549
OBSL1	0.012052	0.033502
RPS9P1	0.087736	0.033499
RTL8A	0.017062	0.033482
AQP3	0.03785	0.033444
WDR4	-0.01648	0.033434
ZNF507	-0.01252	0.033428
HECA	0.021285	0.033425
RF00411	0.261297	0.033418
PTPN5	0.111061	0.033414
AGPAT3	-0.01255	0.033412
P2RX5-TAX1BP3	0.016146	0.033376
TBC1D24	0.01556	0.03337
NUP98	-0.0099	0.033369
PINX1	-0.02635	0.033323
HUNK	-0.01926	0.033307
ZNF710	0.016991	0.033289
STK16	0.013676	0.03328
HACL1	-0.0175	0.033274
EIF2S3B	-0.0903	0.033266
NA	0.129764	0.033256
ZNF285B	-0.08584	0.03322
UCN2	0.101269	0.033206
RNF157	0.013503	0.03312
LRRC8E	-0.1505	0.033118
NA	-0.02943	0.033062
PALM2	0.058611	0.033057
MYPN	0.151783	0.033055
UPF3AP3	-0.13992	0.033052
AC005789.1	-0.10605	0.033038

SND1	-0.00876	0.033033
AP001631.1	0.170098	0.033019
NRG4	-0.02458	0.033004
TRIM45	-0.02321	0.032993
MASTL	0.013687	0.032969
NAPRT	0.028766	0.03296
MAT1A	0.113906	0.032945
ZNF602P	-0.08037	0.032944
ARMCX1	0.015668	0.032923
LINC00899	-0.14843	0.032892
PAIP2	0.009566	0.032871
AC092140.1	0.052439	0.032852
MAGEA3	0.011206	0.032838
MIR222HG	-0.06543	0.032815
ZNF29P	-0.22467	0.032812
GLYATL2	-0.08254	0.032799
CD68	-0.08149	0.032794
TGIF2P1	0.17189	0.03279
CPSF1	-0.01277	0.032782
CYSRT1	0.0485	0.032775
RN7SL130P	0.229378	0.032759
MIR378H	0.229378	0.032759
KSR1	-0.0169	0.032745
ADK	-0.0135	0.032745
CDK11A	-0.0596	0.032698
SH3GL1P3	-0.027	0.032691
AVL9	-0.00949	0.032661
JOSD1	-0.01537	0.032661
PGPEP1	-0.01332	0.032651
AL121749.1	0.135792	0.032647
AP000786.1	-0.05928	0.032608
AC026979.2	-0.05438	0.032588
ACTL6A	0.011862	0.032576
NEB	0.034437	0.032575
RPL22P3	0.208214	0.032565
AL590666.3	0.164968	0.032555
AC092849.1	0.020134	0.032541
C3orf70	-0.10135	0.032506
FBH1	-0.01053	0.032505
AC004908.3	0.066547	0.032501
TARDBP	0.010958	0.032498
LRRC29	0.049198	0.032496
POGLUT2	0.014202	0.032482
CPSF1P1	0.040632	0.032478
BOLA2-SMG1P6	0.070089	0.032467
LINC00909	-0.0163	0.032459
SLC45A4	-0.016	0.032409
FBXO7	0.009831	0.032405
C6orf47-AS1	0.025295	0.032347
SNX12	-0.01155	0.032318

RPL7AP11	-0.14114	0.032313
DNAJB6	-0.00926	0.032308
AC009133.1	0.011279	0.032303
PIGV	-0.02182	0.032303
TRIM28	0.011737	0.032294
GCM1	0.20366	0.032276
SOX6	-0.0289	0.032274
UNC13B	0.012	0.03227
ZNF598	0.012127	0.032251
CEP295	-0.01419	0.032232
NA	-0.20284	0.032232
ADRA2C	-0.01643	0.03222
HMGN1	0.009537	0.032203
AP1S1	0.009461	0.032201
UCK2	-0.00931	0.032189
NA	-0.04624	0.032177
UBE2T	0.013511	0.032164
EPX	-0.12699	0.032154
FAM181B	0.021169	0.032112
MTX1	0.012475	0.03211
LPCAT1	-0.01204	0.032106
DGKE	0.022099	0.032086
NA	-0.06768	0.032067
CPNE2	-0.01378	0.032055
ETV1	-0.03645	0.032039
AC106786.2	-0.0392	0.032033
RSPH10B2	0.023025	0.032033
LGI1	0.122059	0.032028
AL450306.1	-0.01258	0.032004
NA	-0.08915	0.031999
LINC02024	-0.0917	0.031998
LRRC24	0.015821	0.031983
AC107214.1	-0.07995	0.031977
AC083798.1	0.143419	0.031964
NA	0.201616	0.031948
ASH1L-AS1	-0.02628	0.031934
AC026786.2	-0.068	0.031922
RF01241	0.124604	0.031922
CENPQ	0.017849	0.031919
HSDL2	-0.01678	0.031871
AC015845.2	0.208026	0.031868
ABHD4	-0.01973	0.031863
AC002070.1	0.035564	0.031862
AC023024.1	0.024923	0.031823
AC145138.1	-0.05673	0.031817
LINC01711	0.222715	0.0318
LRRTM1	0.025617	0.031795
SYT13	0.082181	0.031777
ZNF83	0.015137	0.031772
AC127496.6	0.02225	0.031769

ZNF580	-0.01481	0.03176
AC073655.2	0.062205	0.031744
SNORD3B-1	0.140194	0.031732
VSIG10	0.014222	0.031718
TYRP1	0.091356	0.031712
KRBA2	-0.04856	0.031706
EPHB3	0.032805	0.031706
RRM2P2	0.146967	0.031639
SIRPB2	0.243339	0.031624
AL031726.1	0.243339	0.031624
A2M-AS1	0.243339	0.031624
ABCA9	-0.13786	0.03162
IFITM2	-0.03175	0.031601
ATN1	-0.0269	0.031586
AP000266.1	0.140496	0.031572
AP000350.6	-0.03727	0.031568
GIPC1	-0.012	0.031548
MCM6	0.015345	0.031541
TRIM59	0.012781	0.031508
NDUFB4	-0.01328	0.031468
RUVBL2	0.009801	0.031456
AC016590.2	0.079667	0.031447
PSME2	0.009417	0.031446
ACACB	0.017185	0.031446
MERTK	-0.01931	0.031443
ZNF703	0.047618	0.031396
TMEM217	0.039984	0.031383
CNNM1	0.026647	0.031381
RPS6KB2-AS1	0.020485	0.031372
RNF10	-0.0119	0.031325
EIF3K	0.010545	0.031299
DYNC2LI1	0.016254	0.031295
AC116021.1	-0.16671	0.031288
PTPN4	0.011696	0.031277
SCUBE1	0.018866	0.031269
SNORA13	0.101843	0.031264
SPATA24	-0.03363	0.031247
WASIR2	-0.03956	0.031215
ATAD3B	0.014106	0.031197
TUB	0.009801	0.031186
ANKRD1	0.03134	0.031167
NA	0.025373	0.031137
PLEKHG5	0.02865	0.031124
TAX1BP3	-0.07289	0.031107
AC023866.1	-0.12623	0.031105
ARPC2	0.010043	0.0311
STYX	-0.01529	0.031098
AC005726.1	-0.02946	0.031082
SLC27A3	-0.01883	0.031071
SOX15	-0.03527	0.031051

AC026348.1	0.085199	0.031012
RPSAP70	0.114476	0.031006
PCP2	0.051324	0.030977
CCDC74B	0.013637	0.03097
PLCE1	0.020807	0.030949
CIZ1	0.01075	0.030933
GUCY1A1	-0.01967	0.030929
SPRY3	-0.02718	0.03092
CALML6	0.137282	0.030891
YME1L1	-0.00905	0.030832
ADAM1B	0.083646	0.030806
COX17	-0.01753	0.03078
NA	-0.05435	0.030778
HOMER3	0.017659	0.030764
AL390067.1	0.123759	0.030747
NFKBIZ	-0.0154	0.030738
HS1BP3	-0.01793	0.03069
GRIN1	-0.01855	0.030669
RNF5	0.010322	0.030642
AL161756.1	-0.0616	0.030628
RPP21	-0.05765	0.030617
DGAT2	0.038079	0.030609
DPY30	0.015315	0.030533
RN7SKP87	-0.16599	0.030526
STRN4	0.010511	0.030513
RHOT1	0.011097	0.030503
ZNF75A	0.02036	0.03046
RPGRIP1	0.061381	0.030455
SLC9C2	0.174866	0.030419
AC087752.2	0.234093	0.030418
TAF9P3	0.234093	0.030418
SSU72	-0.01136	0.030415
WDR83OS	-0.01111	0.030405
GON4L	0.00927	0.030387
ALOX12B	-0.07107	0.030384
PLPP7	0.022207	0.030321
WDR3	0.01068	0.030318
MED16	0.017078	0.030293
SMARCC1	-0.00961	0.030283
CCDC167	-0.01582	0.030255
DNAJA1	-0.01022	0.030251
ZC3H8	0.014236	0.030223
AC013472.3	-0.01896	0.030216
DDX56	0.0085	0.030213
AC139887.1	0.022553	0.030209
UBE2W	-0.01593	0.030185
AP001347.1	0.067932	0.030166
LRP6	-0.0094	0.030166
PLEKHB2	0.009387	0.030132
USP24	0.009523	0.030124

FBXO28	0.009534	0.030113
KCNJ2-AS1	0.05163	0.030082
KCTD5	0.010729	0.030057
LEO1	-0.01197	0.030043
FGF18	0.213063	0.030042
UNC79	0.012396	0.029987
MAGI3	0.010294	0.029953
CFL1	-0.00802	0.029936
SCHIP1	0.01947	0.029926
PFDN2	0.008728	0.029915
PSME3	-0.00865	0.029889
AC023509.2	-0.02042	0.029839
FBXO41	0.016628	0.029838
ZNF518B	0.018631	0.029826
SNX6P1	-0.12267	0.029804
SLC45A1	-0.05566	0.029803
AP000302.1	0.146425	0.029796
AC025580.2	0.126635	0.029769
TFAP2E	0.048854	0.029752
RNF14	-0.01272	0.029751
KLF7	0.009637	0.029737
IGIP	-0.02255	0.029612
AL591846.2	0.040032	0.029597
B3GNTL1	0.01362	0.029573
AC006116.7	0.03159	0.029564
SLC17A5	0.017605	0.029541
MIR29B2CHG	0.026927	0.029525
ARHGAP5-AS1	-0.03843	0.029518
ATG16L2	0.015315	0.029481
HNRNPKP4	0.06751	0.029459
MXD1	0.016341	0.029428
KIF18B	0.011022	0.029399
ZBTB12BP	0.106234	0.029361
POU5F2	0.13104	0.02936
AC114495.2	-0.12537	0.029349
C19orf25	0.011613	0.029282
RBBP4	-0.00792	0.02927
ZDHHC18	0.014137	0.029268
FUNDC2	0.010594	0.029242
RNF40	0.009602	0.029232
AC099542.1	-0.06826	0.029231
CNTFR-AS1	-0.05258	0.029228
AC012640.1	-0.07871	0.029222
HSPD1P4	-0.10483	0.029175
MFAP3L	-0.01688	0.029171
ADIPOR2	-0.0091	0.029147
HSPA12A	0.043558	0.029135
ALDOC	0.016014	0.02912
GINS3	0.017194	0.0291
GPT	0.034328	0.029079

AL512791.1	0.014777	0.029068
COX6CP1	0.125652	0.029059
PHF11	0.127832	0.029032
DNAJC28	0.035056	0.029004
AC006058.1	0.016304	0.028984
HDGF	-0.00824	0.028942
RMRP	0.159426	0.028941
AC025576.1	-0.04809	0.028913
RTCA-AS1	0.067911	0.028907
TTI1	-0.0106	0.028905
SIDT1	-0.22754	0.02889
RF00019	-0.22754	0.02889
BTG1P1	-0.22754	0.02889
HECTD3	-0.01291	0.028844
AC064799.1	-0.05195	0.028826
GSDME	-0.00957	0.028797
UBE2H	0.009364	0.028779
AP3D1	-0.01187	0.028777
BCAS2	0.013006	0.028772
CCDC137	-0.00905	0.028769
CDC42EP1	-0.01738	0.028762
MAP2K1	-0.01036	0.028758
TMEM45B	-0.07272	0.028735
GABRD	-0.12632	0.02873
PPP1R3B	-0.0192	0.028719
NCF2	-0.10132	0.028703
CA15P1	0.159767	0.028695
NIPSNAP3B	-0.03452	0.028693
FAM199X	-0.01232	0.02869
COPS7A	-0.00976	0.028678
KLHL3	0.023237	0.028675
LTF	0.055214	0.028672
POLDIP3	0.010411	0.028669
SCD	-0.0116	0.028666
MTRNR2L2	0.052893	0.028658
DENR	0.009056	0.02864
HPRT1	0.012911	0.02864
NA	-0.1383	0.028639
PLEKHA3	0.012401	0.028612
AL157400.2	-0.08494	0.028609
WDR86	-0.01101	0.028606
AC233266.2	-0.02866	0.028587
SLC30A10	-0.13334	0.028582
NA	0.044801	0.028572
NARS2	-0.01153	0.028561
SCP2	-0.01058	0.028556
LAMA3	-0.11746	0.028548
NA	0.013901	0.02853
ENO2	-0.01111	0.028524
MLPH	0.185373	0.028506

GAB3	0.185373	0.028506
TCEANC2	0.01227	0.028506
DDT	0.014656	0.028506
TMED10	0.008117	0.028493
OR7E128P	-0.07844	0.028469
PDGFD	0.021687	0.028444
LINC00189	0.06935	0.028443
NA	0.021876	0.028429
FTH1P8	0.107296	0.028425
NA	-0.05324	0.028425
AC004890.2	-0.04904	0.028415
ATG16L1	0.011051	0.028399
CCNYL2	0.013038	0.028388
SPTBN5	0.044983	0.028384
AL137779.2	0.110554	0.028368
C16orf91	0.012486	0.028351
ASLP1	0.064031	0.028315
EIF3M	0.009288	0.028297
ACTA2	-0.0297	0.028296
TMEM140	0.011339	0.028295
ALG11	-0.0219	0.028268
AP000344.2	0.059728	0.028257
AL359643.2	-0.04037	0.02824
INKA2-AS1	0.0349	0.028235
NA	-0.05314	0.028234
Z83843.1	0.019461	0.028218
SFT2D1	0.01032	0.02821
AC020978.7	-0.11644	0.028209
THAP3	-0.01387	0.028189
SNX29P1	-0.2042	0.028156
AL356740.2	-0.2042	0.028156
SKA2	0.012235	0.028148
PRDX2	0.009187	0.028139
PDE2A	-0.02149	0.028137
RASA2	-0.01332	0.028133
AC026412.1	-0.01775	0.028103
LUZP2	-0.06691	0.028096
GTF2F2	0.011531	0.028077
AC073896.4	-0.02533	0.028074
SPATS2	0.010528	0.028062
SETP14	-0.04403	0.028058
RNF123	0.0105	0.028042
SLC26A9	-0.21115	0.028033
SWI5	-0.0208	0.028032
GTF2A1	0.010993	0.028021
DIPK1B	0.012326	0.028017
SPRY4-AS1	0.038847	0.02798
C11orf16	0.107915	0.02795
AC012531.1	-0.06259	0.027929
ESF1	0.015859	0.027921

DCAF1	-0.00973	0.027908
UBE2Q2P11	-0.15032	0.027906
GDI1	0.008099	0.027903
NA	-0.01102	0.027898
AC078906.1	-0.12809	0.027896
SNRPEP4	0.076395	0.027872
Z98885.1	0.094655	0.027869
RPGRIP1L	-0.01568	0.027843
ARHGAP40	0.121304	0.027842
CCDC97	-0.01161	0.027833
AC020931.1	0.030537	0.027832
SKIDA1	0.022492	0.027831
CTDSP2	0.012368	0.027823
PTK7	-0.0097	0.027762
LIMK2	-0.01055	0.027759
NAP1L1	-0.00812	0.027757
KCTD15	-0.00863	0.027748
ANXA2R	0.144723	0.027739
CCNDBP1	0.01029	0.027719
MARCH2	-0.01983	0.0277
IGFLR1	-0.11129	0.027677
NEU4	0.071021	0.02767
SSX1	0.256074	0.027649
RF00019	0.256074	0.027649
AL589684.1	0.256074	0.027649
NANOGNBP1	0.256074	0.027649
AC091868.2	0.256074	0.027649
AC087273.2	0.256074	0.027649
AP003032.2	-0.14617	0.027637
RBM34	-0.0449	0.027625
NA	-0.00976	0.027609
MUC4	-0.13717	0.027599
PROSER2	-0.05079	0.027569
AL604028.1	-0.0469	0.027562
TEPSIN	0.013171	0.027532
CA7	0.191522	0.02753
GUCY1B1	0.016398	0.027521
HIST1H3D	-0.04477	0.027517
ASPHD1	-0.01032	0.027515
NA	0.015473	0.027504
AC013472.1	0.147088	0.027498
ZNF302	-0.01124	0.02749
RGS16	-0.01149	0.027478
AC139256.2	-0.06392	0.027471
AF186192.1	0.179732	0.027463
AC008669.1	0.053152	0.027448
CS	0.008627	0.027441
ADCY8	-0.03735	0.027438
TMPO-AS1	-0.00966	0.027428
RNF185-AS1	-0.0204	0.027427

MIR4482	0.114648	0.027415
DCUN1D2	-0.01211	0.02739
UQCRH	0.009361	0.027353
PLCD4	0.011596	0.027347
PDZK1IP1	-0.1192	0.027347
CSNK1E	-0.00872	0.027334
NA	0.056668	0.02733
AC093297.2	-0.01903	0.027305
AC138696.2	0.061398	0.027271
NA	0.189152	0.027261
PTPRN	0.011388	0.027222
TMEM121B	0.024033	0.027205
AC027307.3	0.043785	0.027204
NAP1L4	0.007626	0.027179
AC005746.2	-0.02275	0.027176
LINC01168	-0.07178	0.027176
NA	-0.0599	0.027172
ANGPTL8	-0.06209	0.027172
COL10A1	-0.07337	0.027145
LINC01976	0.179417	0.027108
AC080013.1	0.033394	0.027105
AC011472.3	-0.0247	0.027073
RNF20	-0.00852	0.027057
NA	-0.02434	0.027052
NA	-0.02935	0.027033
NGFR	-0.01539	0.027032
TNIP1	-0.01168	0.027
GPX7	-0.01087	0.026988
MPP3	-0.02069	0.026979
AC138969.2	-0.09475	0.026976
AP000446.1	0.033045	0.026974
AC092295.1	-0.11115	0.026964
ZDHHC5	-0.00882	0.026939
NA	0.016275	0.026907
HSDL1	0.01033	0.0269
BRCA1	0.013657	0.026863
ZNF593	0.013415	0.02686
AC009299.3	0.106113	0.026833
RFLNA	0.081008	0.026819
DUXAP10	0.010685	0.026814
FBRSL1	-0.01472	0.026803
ANKRD61	0.031502	0.026782
OXTR	0.025767	0.026774
SNX5	-0.01095	0.026736
AC012100.2	0.030161	0.026733
RHOQP2	-0.07707	0.026731
AL031775.1	0.0212	0.02673
ELMO3	0.020235	0.026729
KANTR	0.029702	0.026709
RPAP2	-0.01402	0.026694

MFAP2	-0.01076	0.026684
RPS6P16	-0.19792	0.02668
ABHD17A	-0.01041	0.02667
PHLPP1	0.016149	0.026668
TOR2A	-0.01527	0.026641
H2BFM	0.267934	0.026639
CCDC13-AS1	0.267934	0.026639
AL353898.1	0.267934	0.026639
FABP5P2	0.267934	0.026639
NA	0.267934	0.026639
MIR4261	0.267934	0.026639
EIF3A	-0.0072	0.026629
NLRP1	-0.01229	0.026615
FO393418.1	0.163924	0.026594
TCP11L2	0.022718	0.026593
BTN2A1	-0.01079	0.02658
PORCN	-0.02072	0.026569
ZNF780A	-0.01223	0.02656
SNAP91	0.014117	0.026559
R3HDM2	-0.0138	0.026517
EPC1	0.010805	0.026491
NA	-0.13326	0.026481
TOR1AIP2	0.007828	0.026476
SMURF1	0.011626	0.026467
CDC5L	-0.00993	0.026455
NA	0.131489	0.026383
LRRC40	-0.01341	0.026382
WASHC2A	0.018231	0.026369
AC006369.1	0.012411	0.026364
CCDC183-AS1	-0.02796	0.026301
SLC12A7	0.036393	0.026297
FAM3A	-0.01321	0.026283
TRAPPC10	0.008682	0.026262
ZMYM5	-0.01575	0.026258
NA	-0.06288	0.026239
HARS2	-0.00955	0.02622
SLC7A7	0.086267	0.026219
GATC	0.012028	0.026181
SMIM15-AS1	-0.01467	0.026175
HSD17B13	-0.07404	0.026162
AC025171.1	-0.04077	0.026147
ITM2C	0.007292	0.026134
AC005828.5	0.061888	0.026097
LINC02603	0.031239	0.02609
RAB4A	0.008955	0.026078
AL591806.2	-0.06248	0.026058
ACVR1B	0.008852	0.026048
C19orf54	-0.0133	0.026039
PIN4P1	-0.12004	0.026029
RF00019	-0.06527	0.025971

NA	0.089143	0.025964
AL358115.1	-0.05156	0.025945
GNA11	0.008938	0.025916
BRCA2	-0.01541	0.025915
TRIO	0.010466	0.025897
PKNOX1	0.010334	0.025889
SBF2	0.009135	0.025889
GON7	-0.01256	0.025888
AC068580.3	-0.0168	0.025881
AP002954.1	-0.02302	0.025853
FMNL2	0.008694	0.025853
NA	-0.0371	0.025849
MORF4	-0.12875	0.025843
RF00045	-0.13689	0.025816
S100P	0.15793	0.025806
SLC41A3	0.012606	0.025796
WBP1L	-0.00916	0.025775
CCDC62	0.088686	0.025768
PHYH	-0.01278	0.025763
FARS2	-0.01679	0.025759
AC012603.1	0.258841	0.025744
AL031587.3	0.258841	0.025744
BLZF2P	0.124652	0.025736
UVRAG	-0.01265	0.025732
ALKBH3	-0.01495	0.025722
TPCN1	-0.0107	0.02572
CICP8	0.151102	0.025719
RFX5	-0.00794	0.025695
RBMS3-AS3	-0.033	0.025683
DPH5	-0.01301	0.025673
AP000487.2	0.204275	0.025671
POU2F1	0.008469	0.025649
TSR2	-0.01013	0.02564
ZNF438	-0.02179	0.02563
MED24	0.009504	0.025624
ZNF33B	-0.01056	0.025613
AP001605.1	-0.13965	0.02561
DCST2	-0.06358	0.025608
TNKS2	-0.00902	0.025576
COA7	-0.01191	0.025567
AC134050.1	0.184125	0.025534
IL20RB	0.04778	0.025525
LRRK2	-0.06034	0.025519
AP000240.1	0.071409	0.025494
AL357060.1	-0.13901	0.025474
LBP	-0.22457	0.025474
CCDC125	0.013343	0.025473
AC005726.3	-0.04598	0.025468
NA	-0.10829	0.025464
GRAMD1A	0.011212	0.025463

TRIM68	-0.01873	0.025461
WASHC4	0.007816	0.025453
AIMP2	-0.00914	0.025439
GNAI3	-0.00808	0.025421
NECTIN2	-0.02016	0.025388
AP000866.2	-0.05172	0.025383
ABHD14A	0.014621	0.02536
SLC2A3P4	-0.13624	0.025359
EXOC4	-0.00959	0.025337
PEX11A	0.015818	0.025307
SEC14L2	0.01922	0.025299
ZC3H12A	-0.015	0.025295
INAFM2	0.019757	0.025277
AL158212.3	0.013654	0.025277
AC004542.2	0.009587	0.025245
EFHB	0.154445	0.025231
ZNF418	0.154445	0.025231
WDR61	0.008829	0.025183
PNN	-0.00802	0.025183
TRUB1	-0.00993	0.025156
FAM129A	0.016371	0.025134
AC113208.3	0.097873	0.025102
NMRAL1	0.010138	0.025082
AC130462.1	-0.18135	0.025056
AL022324.3	0.146814	0.025039
MTND1P8	0.079531	0.02502
CHMP7	-0.00878	0.025016
PAPOLA	-0.0083	0.02501
ST7-AS2	-0.02566	0.025001
AL354864.1	-0.02169	0.024988
TMX1	-0.01032	0.02497
NOD1	0.013998	0.024962
EP300-AS1	-0.13746	0.024932
SOAT1	-0.01101	0.02493
TEKT2	-0.04406	0.024892
CHCHD7	0.026736	0.024869
FAM98B	-0.01066	0.024853
AC005666.1	0.123136	0.024851
NFATC2IP	-0.00903	0.024839
SNX14	0.009651	0.024815
RNASEH2B	0.009554	0.024812
PPP1R9B	-0.00864	0.024799
TUBB4B	-0.00727	0.024761
AC010273.1	-0.0124	0.024761
TIMMDC1	0.008633	0.024723
ATP5MF-PTCD1	0.016484	0.024688
VDAC3	-0.00921	0.024683
PPP1R11	0.008042	0.024658
HCG25	-0.01364	0.024643
EXOC6B	0.012212	0.024625

PTN	0.025284	0.024617
WIPI2	-0.00732	0.024616
ECT2	0.009181	0.02461
SYDE1	0.010817	0.024585
NPTN	-0.00864	0.024584
CCDC153	-0.07542	0.024563
ZNF181	0.01237	0.024552
DDX17	-0.00713	0.024551
TM9SF2	-0.00725	0.024541
AC011511.3	-0.01727	0.024526
AGPAT4	-0.01098	0.024523
CHD1L	0.009646	0.024519
C9orf78	0.007525	0.024512
VPS13A-AS1	0.058821	0.024502
AC009034.1	-0.03996	0.024484
MAP3K1	0.006641	0.02448
AL355802.2	0.013032	0.024453
TMEM39B	-0.01185	0.024447
ZMYM2	0.006845	0.024418
LINC00484	0.106555	0.024363
RF00019	-0.10831	0.024352
HLA-H	0.021226	0.02434
SFMBT1	0.019288	0.024301
SHPRH	-0.00949	0.024254
NA	-0.07344	0.024248
DNAJC18	0.010794	0.024245
AC098828.2	0.174765	0.024228
PRPS1P2	-0.02549	0.024212
RARG	-0.01068	0.024189
AP006545.2	0.10343	0.024155
JAK1	-0.00793	0.024147
AC025449.1	0.037146	0.02414
IGLON5	-0.01128	0.024111
STX18-AS1	0.02878	0.024079
POLRMTP1	0.033438	0.024036
RNU6-1099P	-0.11033	0.024028
HS6ST2	-0.04954	0.024018
UBBP4	-0.02723	0.024001
CNR1	-0.04979	0.023988
BAK1P1	0.06992	0.023985
AC080023.1	-0.15039	0.02398
MAPKAPK3	0.012645	0.023972
RPL37P23	0.123794	0.023955
AC018695.4	-0.06108	0.023944
GABPAP	0.129251	0.023939
MTCO1P15	0.121769	0.023932
RNU6-216P	0.119306	0.023929
DNAJC12	-0.01036	0.023925
AC005034.4	0.117362	0.023896
TBC1D7	0.012107	0.023889

OTUD4	-0.00854	0.023888
AFAP1L1	0.022702	0.023884
PPP4R2	-0.00926	0.02388
ZNF24	0.007705	0.023877
NA	-0.00744	0.023865
OARD1	0.011162	0.023839
SNORA71B	-0.06311	0.023838
KLF15	-0.01382	0.023837
CD276	-0.01069	0.023831
RABEP2	0.012452	0.023804
SBNO1	-0.00758	0.023794
NUDCD1	0.009398	0.023762
PRPF4	-0.00747	0.023746
TTC1	0.011071	0.023741
SERHL	-0.01979	0.023731
NA	0.016796	0.023726
CFAP69	-0.02021	0.023725
LINC00426	-0.10384	0.023723
MRPS14	0.009135	0.023697
CCP110	-0.00874	0.023679
SNX4	-0.00974	0.023678
ATP6AP2	-0.00785	0.023673
AC006547.1	0.021643	0.023646
CAMTA1-DT	0.075186	0.02362
AC092171.4	0.039196	0.023615
UBLCP1	-0.01011	0.023569
HAGLROS	0.053719	0.023547
L3MBTL4-AS1	0.155893	0.023526
RUNX1	-0.02529	0.023515
ZNF804A	0.010228	0.02351
CYBA	0.009492	0.023488
ZNF420	-0.01214	0.023485
RANBP3	0.008613	0.023468
AKAP11	-0.00859	0.023462
DHRS7B	-0.01363	0.023447
YES1P1	-0.07115	0.023388
TUFM	0.006994	0.023363
ZCCHC10	0.014654	0.023349
PCK2	-0.01165	0.023313
ILRUN	-0.00791	0.023276
CLTA	-0.00682	0.023266
NMB	-0.01519	0.02322
MAP2K6	-0.01267	0.023171
ZNF880	0.016943	0.023167
ENPP6	-0.03145	0.023153
BEND7	0.013824	0.02315
SNX8	-0.01047	0.023116
AC113410.1	-0.10029	0.023088
MYH1	-0.0622	0.023069
WDR97	-0.03228	0.02306

MIR4730	-0.06412	0.023053
NA	0.114349	0.023038
ZSCAN32	0.011889	0.023027
SLC46A2	0.122369	0.023017
NACC1	0.007967	0.02299
NA	0.104485	0.022949
USP35	0.014403	0.022938
AC004771.4	0.020733	0.022935
TBC1D10A	0.020467	0.022896
AL050341.1	-0.10482	0.022846
ULBP2	0.056949	0.022827
AC008663.2	-0.14569	0.022817
BTBD8	0.062162	0.022808
AL445483.1	0.061525	0.022792
RGP1	0.007818	0.022747
SLC47A2	0.077876	0.02271
ATCAY	-0.01278	0.022699
PCDH12	0.029189	0.022689
NA	-0.00766	0.022674
BAG3	-0.0142	0.022673
TSPAN12	-0.05685	0.022617
GAS1RR	0.10706	0.022613
AL031282.1	0.018641	0.022556
AP000919.2	0.027615	0.022488
RN7SL573P	-0.08467	0.022485
G3BP1	0.006978	0.022472
SAR1B	-0.00914	0.022471
PTDSS2	-0.01079	0.022458
EZH2	-0.00767	0.022448
AC089998.3	0.148722	0.022436
NXPH4	-0.05331	0.02242
C1orf189	0.187683	0.022406
CCZ1	0.009494	0.022404
KCNG1	-0.01455	0.022402
RNF2P1	-0.06465	0.022394
NEURL1	0.025375	0.022393
PTGER2	0.028677	0.02239
NSUN4	0.009065	0.022318
AC011461.1	-0.1216	0.022312
AC112512.1	0.134345	0.022307
ATXN10	-0.00652	0.022292
KIAA0825	-0.02255	0.02229
TESK2	0.018253	0.022285
AC090186.1	0.128378	0.022279
ZNF746	0.009754	0.022266
TNFAIP1	-0.00799	0.022254
AC007620.2	0.011994	0.022245
RUBCN	-0.01109	0.022244
TSPEAR	-0.02145	0.022239
FMR1-IT1	-0.10773	0.02223

LST1	0.116528	0.022198
CCDC51	0.010181	0.022196
AC025423.4	0.01186	0.02216
TAB2	-0.00725	0.022156
SNRPC	-0.00803	0.022134
AC104248.1	0.155264	0.022107
PRTFDC1	0.013111	0.022105
ZNF699	0.028551	0.022094
AC012157.1	-0.02346	0.022093
MKRN2	-0.00849	0.022038
DUOXA2	-0.09824	0.022037
AC073367.1	0.132679	0.022028
AL592211.1	-0.10152	0.022009
RPS6KA2	-0.00773	0.022004
SPIN4-AS1	-0.03952	0.021965
CERS5	0.009149	0.021919
SPAG17	-0.01456	0.021916
APC	-0.00891	0.02188
MADD	0.007242	0.021861
MTMR14	0.008881	0.021837
AC013472.2	0.047856	0.021813
ACSF2	0.01132	0.02181
DDX19A	0.007048	0.021797
SPNS3	0.103026	0.021796
NDUFV3	0.009431	0.021791
TMC8	0.101151	0.021745
PRR15L	0.110739	0.021736
H2AFZ	0.008256	0.021706
AP2M1	0.005622	0.021694
STK11	-0.00825	0.02167
CCDC186	-0.01304	0.021667
PEG3	0.013007	0.021663
EEF2K	-0.01096	0.021657
FAM216A	-0.01138	0.021644
NRSN1	0.011774	0.02164
AC106786.1	0.108756	0.02163
NA	0.022433	0.021625
ZNF404	-0.03723	0.021608
CCDC17	-0.04944	0.021572
C8orf33	-0.00705	0.021561
PGBD4	-0.01539	0.021555
CENPBD1P1	0.009131	0.021516
NA	0.08407	0.021513
AC105345.2	-0.04577	0.021487
ACVR2A	0.010881	0.021486
NA	0.097706	0.021442
SMARCAD1	0.007368	0.021396
OR2I1P	-0.03782	0.021384
PEX14	-0.00952	0.021379
FOXD2	0.12139	0.021367

SELENOI	-0.0071	0.021353
EFCAB11	-0.019	0.021325
SEZ6L	-0.01173	0.021322
TPM1-AS	0.014101	0.021315
NA	0.01407	0.0213
PRODH	-0.06493	0.02128
VDAC1P1	-0.11457	0.021268
CHST15	-0.01656	0.021268
RN7SL648P	0.138431	0.021264
DIPK1A	0.014303	0.021254
AC097382.1	-0.221	0.021181
AL354977.1	-0.221	0.021181
NA	-0.221	0.021181
ABHD17AP3	-0.221	0.021181
RNY3P15	-0.221	0.021181
R3HCC1L	0.010742	0.02118
FABP5P3	0.16557	0.021159
STAB2	0.140062	0.021157
HNRNPL	0.006343	0.021141
ITGA2B	0.042219	0.021102
BIRC5	0.00696	0.02108
NA	0.056173	0.021064
AC005288.1	-0.01097	0.021058
RELT	0.010847	0.021034
MPPE1	-0.01254	0.021004
P2RX3	-0.17192	0.020966
AC018558.1	-0.17192	0.020966
MIR3181	-0.17192	0.020966
AC090607.4	-0.17192	0.020966
FSBP	0.074045	0.02096
PODXL2	0.008795	0.020946
AC093879.1	-0.05766	0.020939
LINC01460	0.061167	0.020923
ASPM	0.009521	0.020911
AC009962.1	0.104121	0.02091
SRGAP2C	0.026458	0.020895
HIST1H1B	-0.11653	0.020892
RTL9	0.12511	0.020875
AC118553.1	0.124105	0.020873
NA	0.060127	0.020857
SNURF	0.041812	0.020807
GABPB1	-0.00945	0.020778
NCLN	0.00746	0.020778
MIR3142HG	-0.01756	0.02076
NA	0.054546	0.020757
MRPL55	0.010334	0.020736
NA	0.006552	0.020711
RN7SL431P	0.119402	0.020704
AL136295.2	0.018773	0.020698
ZNF584	0.012318	0.020659

AC026427.1	-0.15119	0.020657
BUB1	0.009003	0.020651
GTF2IP23	-0.04211	0.020608
KIF21A	-0.0058	0.020608
NXNL2	-0.1691	0.020605
OXSM	-0.0128	0.020605
VOPP1	0.008473	0.020538
EPS15	0.007206	0.020534
SRGAP3	0.024172	0.020533
PRDX3P1	-0.07442	0.020518
AC006213.3	0.070424	0.020514
NA	-0.16581	0.020486
SNX6	0.008525	0.020475
CAP1	-0.00587	0.020472
B9D2	0.024749	0.020467
XKR9	0.037169	0.020465
HNRNPA1P4	0.050381	0.020464
LRRC37A5P	0.083078	0.020462
CMC2	0.009532	0.020456
MRPS35	-0.00727	0.020454
COPS2	-0.00731	0.020418
PRRG1	-0.06971	0.0204
ATP6V0A2	0.007606	0.02039
DMAC2L	0.012839	0.020386
RIC3	0.009591	0.020377
SOX11	-0.00918	0.020358
MRPS2	0.007554	0.02035
DDX54	0.008864	0.020342
CNPY4	-0.00883	0.02034
NA	0.01476	0.0203
SUSD1	0.010922	0.020297
AP2A2	0.007118	0.020282
AC104117.3	0.071683	0.020266
AC087741.1	-0.02038	0.020258
AL672207.1	0.187218	0.020253
AC009542.1	0.187218	0.020253
AC004067.1	-0.08346	0.020247
AC006487.1	-0.01743	0.020222
STK26	0.009141	0.020215
PTPRK	-0.08103	0.020214
SILC1	0.033185	0.020201
DDTL	-0.00966	0.020191
ACTG1P17	0.064541	0.020058
NA	-0.14685	0.020039
MYH2	-0.09518	0.020023
ARPP21	-0.05191	0.020008
PEX5	0.009606	0.02
ZNF490	0.02856	0.019981
C4orf3	0.008933	0.01998
TCN2	0.032286	0.019969

AC084782.1	0.041975	0.019961
PCDHGB3	0.061425	0.019956
ZMAT2	0.006739	0.019918
MKLN1	0.00665	0.019901
STRA6	-0.00708	0.01989
ZSCAN22	-0.01301	0.019886
GLS2	-0.0237	0.019871
SRP54	0.007561	0.019858
ZNF185	-0.01931	0.019857
NEBL	0.026049	0.019803
HNRNPA3P3	0.159561	0.019798
BNIP3P16	0.18298	0.019796
ST13	-0.00604	0.019786
DNAH12	0.088498	0.01974
AC091982.1	-0.0905	0.019738
TFAP2B	0.017401	0.019735
KDM5C	-0.00664	0.019729
TDG	0.00741	0.019713
NA	0.020408	0.019695
PITRM1	-0.00753	0.01967
CAPN7	0.007096	0.019639
MAD2L1	-0.00906	0.019639
NA	0.062427	0.019636
PITHD1	-0.00853	0.019618
ALG10B	0.014373	0.019584
ALDH3A2	0.007514	0.019576
NUB1	0.005957	0.019569
IMMT	0.005747	0.019537
ZMPSTE24	-0.00786	0.019517
CDK5R1	-0.00802	0.019511
AL590677.1	-0.07554	0.019477
AL358473.1	0.111332	0.019466
MTF2	0.006687	0.019426
RNF4	0.006159	0.019411
MTND2P28	-0.01328	0.019385
GCH1	0.019809	0.019369
NA	0.012977	0.019366
NUDC	0.006109	0.019356
VPS36	0.006716	0.019354
ARFGAP2	-0.00736	0.019352
HHAT	0.009588	0.019345
GPC6	-0.00965	0.019339
AL022328.4	-0.01365	0.019293
NA	0.126285	0.019269
AC008026.1	-0.09407	0.019268
RAB29	0.008552	0.019266
CKS1BP1	0.083767	0.019244
BBOF1	0.019209	0.019232
ACSL1	0.007133	0.019223
TMX2	0.006349	0.019222

AC078777.1	0.013926	0.019145
GTF3C2	-0.00534	0.019082
AC008761.1	-0.01951	0.019069
ADNP-AS1	-0.02407	0.019045
AL627402.1	0.134751	0.01904
RPL23P2	0.029912	0.019003
ZNF619	0.013657	0.018984
SNORD62B	0.05711	0.018981
NA	-0.12219	0.018975
EFNB3	0.008331	0.018919
AL031775.2	-0.05719	0.018879
AC010240.2	-0.15439	0.018878
ARMCX6	-0.00997	0.018872
TBPL1	-0.00745	0.018867
NPEPPS	-0.00553	0.018848
TMEM67	0.008902	0.01884
AC119403.1	-0.03522	0.018819
TTLL3	0.009664	0.018816
ZSWIM5	-0.01442	0.018798
NR1D1	-0.01388	0.018757
C14orf119	0.007275	0.018735
KCNJ10	0.100854	0.018714
HDAC5	-0.00672	0.018714
CARNMT1-AS1	0.076453	0.018703
LRIF1	0.007927	0.018696
CYP1B1-AS1	0.054836	0.018688
PGA5	0.150609	0.018687
AC010335.1	-0.02373	0.018685
RCN1P2	-0.02324	0.018662
HMX1	0.017052	0.018643
AC004448.3	-0.01613	0.018613
ASTE1	-0.01017	0.018611
SCARNA13	0.028827	0.018597
AL513329.1	0.043006	0.018591
B3GALT1	-0.01555	0.018585
RPL31P61	-0.11567	0.018584
AC129102.1	0.036844	0.018584
LINC01607	-0.04703	0.018567
HIBADH	0.007132	0.018563
SAMD14	-0.00891	0.018561
ARHGAP27P1-BPTFP1-KPNA2P3	0.020438	0.018555
LINC01063	-0.11478	0.018524
ZNF148	0.007734	0.018517
MCM3	-0.00552	0.018515
NDUFAF3	0.00731	0.018513
DOCK7	0.006045	0.018465
NUDT9	-0.0086	0.018451
RPL5P18	0.067014	0.018449
NA	-0.0623	0.018446
ACVR2B	-0.00664	0.018444

NTN3	-0.01904	0.018427
CFD	0.081557	0.01842
AC009716.1	0.087688	0.018417
PF4V1	0.162043	0.018397
NA	0.162043	0.018397
AC012569.1	0.162043	0.018397
ASXL2	-0.00669	0.018391
NFAT5	-0.00765	0.01839
TBX6	0.020314	0.018345
PDZRN3	0.009547	0.018342
HORMAD2-AS1	0.031642	0.018338
OSGEPL1	0.010192	0.018328
DISP1	0.010822	0.01831
COL11A2	-0.02196	0.018301
DAP3	0.005865	0.018254
OS9	0.006716	0.018247
ALAS2	0.110476	0.018193
ATG13	0.006197	0.01819
AC009065.1	-0.02263	0.018176
RASSF3	0.007042	0.018175
MGLL	0.01777	0.018175
AC114730.1	-0.01996	0.018154
AL358781.2	-0.02509	0.018137
PCDHGA6	-0.03546	0.018128
SCGB2B2	0.072209	0.018082
ZNF207	-0.00527	0.018057
AHSG	0.171254	0.018021
AC080125.1	0.171254	0.018021
AC009486.1	0.171254	0.018021
NA	-0.07006	0.018009
AP000842.2	0.108864	0.017971
LMNTD1	-0.01809	0.017966
DDX42	-0.00468	0.017958
ZNF398	-0.00782	0.01794
LCA5L	-0.02324	0.017924
JUP	-0.02562	0.017914
CHAC2	-0.01747	0.017904
EIF4A1P2	-0.05364	0.017901
NA	0.031345	0.017888
EMID1	0.02073	0.017886
DNM1	0.006621	0.017885
C22orf39	-0.0108	0.017882
AC020661.1	0.071851	0.017857
HAS3	0.008174	0.017841
C2orf76	-0.01416	0.017814
GAPDHP14	-0.07515	0.017808
SLCO2A1	0.058397	0.017788
UTP14A	-0.00586	0.017782
CDC40	0.006433	0.017776
NCBP2AS2	0.007024	0.017775

AC006547.2	0.013012	0.017761
AC098824.1	0.111917	0.017745
TMEM263	-0.00647	0.017715
PRSS3	0.009812	0.017686
FXYD2	-0.14492	0.017674
RPSAP13	-0.14492	0.017674
CES1P2	-0.14492	0.017674
AL080317.1	-0.02217	0.01765
ZNF615	0.010817	0.017649
FAM57A	0.007759	0.017646
AL391001.1	0.075859	0.017642
NA	0.039068	0.017639
DPH2	-0.00552	0.017616
FDPSP8	-0.0765	0.017613
LINC02535	-0.09764	0.017582
AC108058.1	-0.06177	0.017582
WASH7P	-0.00976	0.017579
C20orf96	0.008127	0.017571
AMZ1	-0.03116	0.017564
CCNA2	-0.00794	0.017563
PCDHGB4	-0.05632	0.017561
ERCC8	0.010846	0.017541
GNMT	-0.0443	0.017529
MCM9	0.009797	0.017515
CSF2RB	0.127609	0.017509
PLEKHM2	-0.00684	0.017508
SEM1	0.00687	0.017499
AP005329.1	0.007187	0.017491
SURF1	0.006222	0.017459
NLRC4	-0.02557	0.017426
EFNA3	-0.02054	0.017401
CRADD	0.011355	0.017398
EEF1A1P9	-0.03041	0.017391
AKAP1	0.006664	0.01739
ARSD	-0.00881	0.017339
MGAT3	-0.01027	0.017316
TPD52L1	0.122324	0.017306
AL139184.1	-0.07986	0.017299
SLC6A5	-0.09261	0.017282
TAF10	0.00585	0.017282
AP003068.3	0.01343	0.017281
RELCH	0.007458	0.017267
CCDC18	0.007769	0.017252
PRR34-AS1	0.027068	0.017238
CNTROB	-0.00811	0.017237
KREMEN1	-0.00835	0.017217
USP3	0.007295	0.017216
AC005480.1	-0.01095	0.017207
ZHX2	0.019079	0.017206
RBM48	-0.00752	0.017168

REXO4	0.006784	0.017145
TRIM21	0.012044	0.017138
C2CD2	0.008158	0.017133
SLCO3A1	-0.01173	0.017124
CLU	0.009364	0.017117
DACT3	-0.00726	0.017115
ZMYM4	0.005087	0.017037
CEP295NL	-0.0398	0.017025
STK11IP	0.006766	0.016975
SLC22A18	-0.01647	0.016974
CENPL	-0.00878	0.016942
BATF3	0.029178	0.016932
POLR1E	-0.00673	0.016905
DYNLT1	0.006467	0.016905
ABCC6P2	0.052891	0.016886
SPATA20	-0.00744	0.016866
RIMS2	-0.01188	0.016844
LCAT	0.018123	0.016824
HNRNPCP3	-0.10781	0.016824
AC156455.1	0.040357	0.016819
ATG2A	0.01072	0.016787
UFD1	-0.00551	0.016781
NA	-0.0103	0.016765
NUP205	-0.00497	0.016757
SEC61B	0.006251	0.01674
FXR2	-0.00597	0.016733
EDDM13	-0.03387	0.016717
LINC01268	0.068699	0.016704
NA	0.029804	0.016691
AP003086.2	0.018027	0.016679
BOD1L1	-0.00498	0.016656
EXTL2	0.00563	0.016654
IL20RA	0.01488	0.016652
TIA1	0.005462	0.016629
CISH	-0.01628	0.016601
TSSK6	0.015198	0.016597
SLC39A3	-0.008	0.016589
RPL23AP82	0.009377	0.016539
POLE2	0.012155	0.016535
RAI14	-0.00725	0.016519
AC018557.1	0.006412	0.016478
C16orf86	-0.01741	0.016474
AL355075.4	-0.02618	0.016466
P4HA2-AS1	0.024038	0.016463
DHX15	-0.00515	0.016447
PHC3	-0.00716	0.016447
AP001429.1	0.133255	0.016445
PCDHGC4	-0.01136	0.016445
AMBP	0.077091	0.016407
SMUG1	0.005971	0.016398

NA	0.019598	0.016382
LINC00324	0.025097	0.016377
CKAP2L	-0.01023	0.016361
ZNF462	-0.00798	0.01636
ZNF709	-0.06537	0.016343
RFX4	0.056702	0.016338
CCHCR1	0.010396	0.016331
NA	0.061351	0.016311
SMOC2	0.036999	0.016307
AL121944.1	0.083054	0.01628
AC072039.2	-0.06878	0.01628
DHCR24	0.007347	0.016274
ASNA1	-0.00531	0.016272
SIX5	0.008451	0.016247
C1orf174	-0.00929	0.016242
HSP90AA1	0.005069	0.016238
UBE3A	0.005797	0.016212
PYCR1	0.004628	0.0162
NKRF	-0.00818	0.016198
BCAT2	0.008108	0.016188
ZMYND15	-0.11123	0.016162
CCDC175	0.017876	0.016125
AP002495.1	0.018206	0.016123
RPN2	-0.00439	0.016075
CHID1	-0.00525	0.016068
PCBD2	-0.01076	0.016018
RAB33B	0.008729	0.016014
FAM118B	-0.00838	0.016012
NA	0.019296	0.016012
S100A4	0.020017	0.016006
AC112484.1	0.023979	0.015992
AP002957.1	-0.06354	0.015985
TIMP4	0.093573	0.015967
FAM27C	0.083307	0.015962
RNU6-882P	-0.06703	0.015952
AC011346.1	-0.04442	0.015921
RPS19P1	-0.10757	0.015919
ECEL1P3	0.121486	0.015898
SLC22A31	0.121486	0.015898
TAT-AS1	0.121486	0.015898
SNRNP40	-0.00593	0.015837
SIX2	-0.02521	0.015834
HEXA	-0.00664	0.015826
ZBTB9	0.006262	0.015806
WASH8P	-0.00761	0.015782
STX1B	-0.00897	0.015774
VPS33B-DT	0.049886	0.015766
CZIB	0.00614	0.015763
UBE2E1	0.00569	0.015751
AL359094.1	0.020866	0.015743

MRPS16	-0.00497	0.015717
WDR81	-0.00715	0.015697
DLGAP4-AS1	-0.01013	0.015672
EIF3E	0.004744	0.015666
AC012065.3	-0.08241	0.01566
SLK	-0.00608	0.015641
RIMS4	0.005575	0.015629
ZBTB24	-0.00865	0.015612
CLIC1	-0.00484	0.015611
RBPMS2	0.010916	0.015605
SEC23A-AS1	-0.0373	0.015585
ATXN7L1	0.012372	0.015584
TOMM40	0.004824	0.015582
CAT	0.007937	0.015544
CAPN3	0.088169	0.01553
ID3	-0.00516	0.015492
EBI3	0.013106	0.015479
AC135721.1	-0.05536	0.015475
RAB35	0.005915	0.015471
CRIP1	-0.03718	0.015456
EED	0.007277	0.015449
SLC25A25	0.006817	0.01544
AC011290.2	-0.01891	0.015369
FOXC1	-0.00689	0.015302
AL158156.1	-0.07881	0.015295
MARK3P3	-0.11813	0.015273
NCBP2-AS1	0.009658	0.015254
WASHC2C	0.006323	0.015239
LINC02018	-0.04637	0.015237
UTS2B	-0.01347	0.015235
FAM49A	-0.01583	0.015202
PGP	-0.00622	0.015178
CHGB	0.007644	0.015168
ARMCX5	0.011081	0.015135
NA	-0.04057	0.015116
DGCR8	0.005585	0.015104
NMT2	-0.0087	0.015028
SLC26A1	-0.01329	0.014981
SIPA1L1	0.006293	0.014977
C21orf62-AS1	0.028286	0.014962
CSKMT	-0.00961	0.014954
TDRD5	-0.1463	0.014953
FAM90A1	-0.1463	0.014953
NA	-0.1463	0.014953
TNPO1P1	-0.1463	0.014953
AKR1B1P1	-0.1463	0.014953
AL355994.2	-0.1463	0.014953
NA	-0.1463	0.014953
AC131392.2	-0.1463	0.014953
AC015660.2	-0.1463	0.014953

ZNF554	0.011566	0.014939
DNA2	-0.0077	0.014918
THAP10	-0.00945	0.014914
CNTNAP1	-0.00704	0.014913
TXNRD2	-0.00664	0.014895
AL360014.1	-0.11291	0.014894
NET1	-0.00595	0.014879
TCEAL3-AS1	-0.09454	0.014849
NA	-0.09454	0.014849
UBL7-AS1	-0.01405	0.014827
NA	0.037223	0.014792
ZNF586	0.011398	0.014788
ATG9B	0.011891	0.014785
PSRC1	0.007268	0.01477
NIPAL3	0.00761	0.014759
NKX1-2	0.081216	0.014735
USP50	-0.01899	0.014734
NOC4L	-0.00715	0.014716
HK3	-0.02809	0.014713
DPYD	-0.00948	0.01471
UBA52P5	0.091839	0.014683
UEVLD	0.007595	0.014678
GNB4	-0.00766	0.014664
PDE7A	0.006189	0.014638
SYNGAP1	0.007804	0.014636
ADCYAP1R1	0.005058	0.01463
AC011497.2	0.030797	0.014588
TMTC1	0.009261	0.014574
MTURN	0.004728	0.014572
OLFML2A	0.014714	0.014569
NA	0.042373	0.014564
CHORDC1	-0.00539	0.014564
ACAP1	0.029083	0.014561
DHX38	-0.00514	0.014558
PHOX2A	-0.00525	0.014557
EIF3LP2	0.084939	0.014553
SMIM17	-0.06803	0.014533
AC024270.4	-0.06761	0.014524
RGS9	-0.01684	0.01452
ZNF268	-0.00647	0.01452
IFRD2	0.005289	0.01452
CCDC121	0.01153	0.014515
LMBRD2	0.005516	0.014514
NA	-0.14207	0.014504
AL365434.1	-0.14207	0.014504
AC079174.1	-0.14207	0.014504
AC026464.5	-0.14207	0.014504
NA	-0.14207	0.014504
GSTO2	0.032771	0.014479
FGF14-AS1	-0.12037	0.014463

GRHPR	0.005406	0.01446
RNF144A-AS1	0.009943	0.014448
AC107464.2	-0.13813	0.014424
CCNJ	-0.00602	0.014422
PIAS3	0.006956	0.014421
NA	0.056006	0.014405
AC008749.1	-0.03288	0.014376
TAB3-AS2	0.01997	0.014376
AXIN1	0.007333	0.014366
LINC01165	-0.07331	0.014356
R3HDM4	-0.00589	0.014331
AC008074.2	0.053396	0.014331
SET	0.004205	0.014331
PMM2	-0.0067	0.014327
MMACHC	-0.00886	0.014321
AC010624.3	-0.01392	0.014274
RF00019	0.075188	0.014253
ZSCAN26	-0.00681	0.014241
ZNF395	-0.00533	0.014232
CDYL	-0.0071	0.014222
FYTTD1	0.005202	0.014218
NA	0.01196	0.014208
AL354714.2	0.050878	0.014191
SYT7	0.009512	0.014187
ZDHHC4	0.005407	0.01418
JARID2	0.005876	0.014144
ST8SIA1	-0.0687	0.014141
MTATP8P2	-0.03973	0.014138
AC009120.2	-0.00538	0.014099
B9D1	0.009942	0.014095
ZNF383	-0.0107	0.014088
AP000688.4	0.020621	0.014068
EIF4A1P10	0.018464	0.014047
AC009961.1	-0.03488	0.014032
PRKAR2B	-0.00479	0.014028
CCT8P1	0.024947	0.013996
PEBP4	-0.02301	0.013991
AC145422.1	-0.02548	0.013975
MAPK9	0.005635	0.013947
TMEM80	0.007322	0.01393
CDH8	0.042384	0.013895
GNPDA1	0.004751	0.013847
DLEU2L	-0.042	0.013838
DNAH11	-0.02659	0.01383
PUS7	-0.00523	0.013817
MMEL1	0.012977	0.013745
AL355310.1	-0.05602	0.013718
SMPDL3B	0.039263	0.013697
TPBGL	-0.02693	0.013662
FBXO3	-0.00566	0.013651

CAMSAP2	0.004289	0.013641
A1BG	-0.01921	0.013635
FAM133CP	0.100405	0.013634
SPHK1	-0.00887	0.013575
HDLBP	0.00368	0.01357
ASB6	-0.0054	0.01357
WSPAR	0.101018	0.013557
PUM2	-0.00384	0.013545
PCDHB5	-0.02056	0.013543
TIGAR	-0.00715	0.013533
AD000091.1	0.059734	0.013503
EFNA4	0.00959	0.013499
BRICD5	-0.00731	0.013476
PSMC1P1	0.023539	0.013468
AC068987.1	0.033209	0.01345
FRMD6	0.00605	0.013447
DNER	0.023806	0.013412
PEPD	-0.00497	0.013369
WASHC3	-0.00847	0.013366
NA	0.004774	0.013363
AL603839.1	-0.01414	0.013352
SNHG21	0.019627	0.013346
PKD2	0.005102	0.013339
AQP11	-0.01956	0.013294
NKG7	0.096638	0.013284
AC015923.1	0.033865	0.013248
AC068724.1	-0.01578	0.013244
SPPL2B	0.005975	0.013225
MICALL2	-0.00885	0.013198
AC092757.2	-0.05554	0.01316
AP002409.1	-0.02645	0.013158
TPPP3	0.014667	0.013155
UTP25	0.003938	0.013139
S100PBP	0.005276	0.013103
AC019257.1	0.058447	0.013083
NA	-0.04603	0.013043
AL133268.1	0.081891	0.013042
LINC02182	0.051046	0.013021
CCDC122	-0.01311	0.013012
MCCD1	0.048866	0.013005
NA	0.050176	0.012986
AC087521.2	0.01146	0.012969
AC027544.1	-0.07473	0.012965
PARP4	0.00418	0.012957
AZGP1	-0.04609	0.012955
ERBB4	-0.0306	0.01295
NA	-0.02995	0.012943
AC020978.2	-0.01556	0.012929
SLC12A5-AS1	0.011553	0.012907
HNRNPA1P48	-0.0093	0.01289

AKIRIN1	0.004231	0.012887
LIN37	0.015882	0.01288
LINC00884	-0.06163	0.012848
MIR5587	-0.0376	0.012801
GLYR1	0.004352	0.012776
CR382285.1	0.014716	0.012774
KCNG2	0.043985	0.012768
AC103810.2	0.022738	0.012767
AGFG1	-0.00432	0.01275
AC005050.1	0.079343	0.012711
ANKRD20A18P	-0.05188	0.012698
ARAP1	-0.00529	0.012695
SUMO3	-0.0038	0.012691
AC107884.1	0.026832	0.012684
RXRA	0.021348	0.012681
ZNF721	-0.00638	0.012677
CTBS	0.007952	0.012644
NA	0.058261	0.012616
SMG1P5	-0.01643	0.0126
CYTOR	-0.01365	0.012584
RAB11FIP1	-0.00751	0.012551
CMSS1	-0.00601	0.012536
SINHCAFP2	-0.04513	0.012521
AP001029.2	0.006388	0.01252
LAMTOR2	-0.00419	0.012509
AL020993.1	-0.02984	0.012506
AC080013.4	-0.02579	0.012487
PIGQ	-0.00562	0.012478
SELENOTP1	0.088524	0.01246
MLX	-0.00457	0.012456
CLEC11A	-0.01303	0.012405
GLT1D1	-0.0699	0.012383
AC018521.4	-0.00523	0.012373
NA	-0.02813	0.012368
AC017104.1	-0.04073	0.012368
AC074117.1	-0.00487	0.012358
CKAP4	0.00462	0.012346
ZNF141	-0.00667	0.012339
INTS12	-0.00639	0.012339
BMS1P4	0.028203	0.012334
BACH1-IT1	0.026	0.012312
SV2C	-0.00609	0.012301
ASNSP3	0.062102	0.012293
SCAI	0.006801	0.012286
KRT18P5	0.06515	0.012278
CROCCP3	-0.00814	0.01227
PHF20L1	0.003936	0.012265
POP1	-0.00525	0.012217
AC011753.4	-0.0475	0.012213
CHD3	-0.00463	0.012211

AL591212.1	-0.0365	0.01221
AP005061.1	-0.02129	0.012203
GABARAPL2	0.004261	0.012193
ALK	-0.00608	0.01218
AC095057.3	-0.01841	0.012143
CDC42	0.004434	0.012135
LINC00629	-0.09817	0.012133
AC087721.1	-0.09817	0.012133
TMEM123	0.005638	0.012114
CHCHD3	-0.00407	0.012101
AC004471.2	0.085739	0.012068
NMRK1	-0.0366	0.012041
ERVW-1	-0.03106	0.012027
AC010976.1	0.00912	0.012006
PRR15	0.060895	0.012004
NA	-0.03975	0.01198
XPO5	-0.00349	0.011968
ZNF781	0.012966	0.011956
AC107983.1	0.039538	0.011952
PPP6C	0.004065	0.011936
AC087286.3	-0.01526	0.011935
AC002347.1	0.09205	0.011932
MPIG6B	-0.04196	0.011929
CDCP2	0.119263	0.011921
SNORD73B	0.119263	0.011921
AC021016.1	0.119263	0.011921
DNAJB8-AS1	0.119263	0.011921
NECTIN3-AS1	0.119263	0.011921
DNAH10OS	0.010243	0.011921
ERVK13-1	-0.00587	0.011921
NA	0.038092	0.011915
ERAP1	-0.0049	0.011912
ABCA1	0.016483	0.011906
AL355877.1	0.066132	0.011898
NA	-0.03935	0.011886
SOD3	0.0453	0.011878
SP3	0.003843	0.011852
TNFSF13	-0.01673	0.011851
RBM7	0.00713	0.011849
AL359397.2	0.013005	0.011849
CHI3L2	-0.04963	0.011795
RN7SL737P	-0.02245	0.011753
SLC22A13	-0.06745	0.011749
UBE2D3	0.003727	0.011735
SLC20A2	0.004098	0.011729
AMPD2	-0.00483	0.011728
TNN	-0.02037	0.011717
TMEM54	0.011175	0.011711
AP003396.3	-0.07755	0.011703
NA	-0.06857	0.0117

AC004884.2	-0.04039	0.011679
ADGRF3	0.012478	0.011673
NA	-0.04208	0.01162
ZC3H3	0.00752	0.011594
CHN2	-0.01199	0.011575
PNCK	0.011947	0.011495
UQCRC1	0.003626	0.011465
U2SURP	0.003981	0.011464
AP001442.1	-0.05427	0.011442
IDSP1	0.028396	0.011425
MICAL2	-0.00644	0.011391
HAUS6P3	-0.02497	0.011388
ITGA9-AS1	-0.00866	0.011371
AC118754.1	0.010731	0.011369
THSD7B	0.011145	0.011366
DLGAP5	0.00578	0.011354
POLN	-0.03532	0.011341
ARFRP1	0.005099	0.011332
CALCOCO2	0.004632	0.011321
VIPR1	-0.08542	0.011306
FAHD1	-0.00473	0.011303
AC022034.1	0.084636	0.011299
AC092338.1	-0.0056	0.011294
BPHL	0.005728	0.011285
CUZD1	-0.01829	0.011263
ANKRD18CP	0.04407	0.011262
AF111169.3	0.007505	0.011258
GLUD1	-0.0037	0.01123
ACOT8	-0.00521	0.011224
CHRNE	0.012198	0.011207
TIMP1	0.011053	0.011185
CCDC174	-0.00467	0.011157
PSMG4	0.005364	0.011151
NA	0.041179	0.011148
ZNF540	0.016113	0.011145
AC135506.1	-0.02588	0.011138
NA	0.08509	0.011135
AC100821.2	0.075064	0.01113
TPCN2	-0.0062	0.011123
TRIM37	0.004024	0.011118
KRBA1	-0.00716	0.011102
AC005339.1	0.015829	0.011073
LINC01431	0.057186	0.011049
PNMA8A	-0.00425	0.011044
MBTD1	-0.00328	0.011024
AL354751.1	0.047717	0.011017
PLAC9P1	0.022064	0.01099
DDX21	-0.00314	0.010988
FAM241B	-0.00842	0.010979
IRAK2	0.008025	0.010962

PTS	-0.0065	0.010955
IMPDH2	-0.00335	0.010906
AC007364.1	-0.00784	0.010888
MELK	-0.00559	0.010883
GREB1L	0.095692	0.010881
NDUFAF4P2	0.095692	0.010881
AL050338.1	0.095692	0.010881
NA	0.008583	0.010877
PUS3	-0.00704	0.010872
ACADVL	0.003587	0.010863
RCHY1	0.004573	0.010862
AC092634.4	-0.01575	0.010853
AC010864.1	0.018946	0.010841
MT-TF	-0.03565	0.010833
PDLIM2	-0.01145	0.010833
LIN9	0.004958	0.01083
IGHV4-34	0.018976	0.010827
OR7E13P	-0.07693	0.010824
LINC00471	-0.02355	0.010822
OPLAH	0.040851	0.010814
IGHV3-37	0.057507	0.010795
FPGT	-0.00787	0.01079
C12orf40	-0.03039	0.010782
PDCD6IP	0.00328	0.01078
RMDN3	0.005792	0.010754
AP001646.1	0.09628	0.010729
TBC1D31	0.005886	0.01072
PRPF18	-0.00709	0.010693
HMGB1P19	0.103476	0.010686
BSG	-0.00337	0.010684
AL049796.1	0.015303	0.010679
TLR4	-0.00769	0.010619
HEXA-AS1	0.032673	0.01056
CALY	-0.0082	0.010555
MAP2	0.003798	0.01054
NA	-0.0035	0.010526
HS6ST1P1	-0.07059	0.010524
TSEN54	0.004225	0.010503
AGO3	0.003732	0.010494
ZFP37	-0.00667	0.010493
RF00019	0.04718	0.010491
NOP53-AS1	-0.01227	0.010485
RAD23B	0.002823	0.010482
CHRM5	0.02921	0.010481
ATP8B3	0.01366	0.010447
AC097532.2	0.010521	0.010445
AMPD3	0.007804	0.010415
CALHM1	0.09159	0.010415
TMSB4XP2	0.09159	0.010415
UHRF2P1	0.09159	0.010415

AC009220.1	0.09159	0.010415
AC100839.1	0.09159	0.010415
NA	-0.00577	0.010388
AC009487.2	0.030157	0.010374
NA	0.0179	0.010325
SLC39A1	-0.00322	0.01032
SLC52A2	-0.00373	0.01032
NA	0.024687	0.010315
AKT3-IT1	0.076172	0.010301
APBA2	0.003373	0.010298
NA	-0.01978	0.010289
ARPC1B	0.003782	0.010285
AC027514.2	-0.03934	0.010277
TBX4	-0.08429	0.010276
NXPE4	-0.08429	0.010276
KAAG1	-0.08429	0.010276
AC244502.1	-0.08429	0.010276
ATAT1	-0.00402	0.010272
KIAA1324	0.021978	0.01021
AC004837.2	0.044754	0.010182
BNIP3P10	-0.0759	0.010143
AC006116.10	-0.01976	0.010125
AL137002.1	0.039976	0.010121
AC087439.1	0.013019	0.01006
PPP1R14D	-0.076	0.010033
AL359076.1	-0.04982	0.009997
LIG3	0.003908	0.009985
LRRC61	0.005982	0.009933
TASP1	0.005741	0.009921
ZBED6CL	-0.00524	0.009915
MKRN3	0.007374	0.009896
ACBD3-AS1	0.005953	0.009893
FIRRE	0.079512	0.009879
AL445487.1	0.079512	0.009879
SCARNA14	0.079512	0.009879
NA	0.046793	0.009855
ZSCAN20	0.006199	0.009842
FAT2	0.019415	0.009839
GASK1B	0.010239	0.009834
CHRM2	0.010919	0.009829
AC022272.1	-0.03792	0.009803
RBFOX2	0.002652	0.009797
TMEM198	-0.00507	0.009777
PRIMPOL	-0.00526	0.00976
OPHN1	0.008835	0.009759
GNL3LP1	-0.0533	0.009747
SAAL1	0.004062	0.009746
LZTR1	0.003835	0.009744
AC008870.1	0.004609	0.009741
AL354836.1	0.004069	0.009738

ABCA6	0.041191	0.00971
EIF4A1P4	-0.04964	0.009697
AC127024.2	-0.03772	0.009694
XPO7	0.003099	0.009687
ZNF419	-0.0059	0.009683
AVPR1B	-0.03573	0.009683
BRF1	-0.00408	0.009656
LENG8-AS1	-0.01104	0.009639
AL159169.2	-0.02013	0.009629
NARF	0.00293	0.009625
AC004923.4	-0.02764	0.009624
ZNF333	-0.00544	0.009616
AC104758.2	-0.01986	0.009599
CXCL5	-0.01747	0.009599
ZNF700	-0.00608	0.009564
CHD7	-0.0034	0.009546
SPATA17	0.010658	0.009545
ACOXL	-0.03981	0.009532
AC004987.2	-0.03797	0.009526
MRPL39	0.004773	0.009505
AC091806.1	-0.02878	0.009493
SURF6	-0.00319	0.009483
MARCH7	-0.00377	0.009479
NAXE	-0.00316	0.009468
NELFA	-0.00382	0.009456
NT5CP1	-0.04162	0.009428
AC073195.1	0.015257	0.009417
AL445222.1	-0.01106	0.009397
WDR45	-0.00403	0.009391
STAG3	-0.00543	0.009379
AC092164.1	0.021451	0.009377
AP003170.1	-0.03041	0.009376
AC012358.2	-0.014	0.009374
NDST3	0.01827	0.009371
SYT8	-0.04873	0.009347
POLR3D	-0.003	0.009331
RPSAP19	0.024906	0.009303
SMKR1	0.011772	0.009297
CNDP1	-0.07321	0.009274
RNA5SP201	-0.07321	0.009274
AL109618.1	-0.07321	0.009274
AL034405.1	-0.07321	0.009274
GPCPD1	0.006388	0.009263
CCNE1	0.004708	0.009246
DFFA	-0.00293	0.009232
GRPEL1	-0.00374	0.009215
AC026688.2	-0.0115	0.009201
AL022328.2	0.00648	0.009184
REV1	0.00364	0.009181
AL591845.1	-0.01756	0.009174

FCHSD2	-0.00349	0.009163
AC073415.1	-0.03261	0.009163
URI1	-0.00291	0.00912
ADGRG5	-0.07495	0.009114
LINC00452	-0.07495	0.009114
AL158068.1	-0.07495	0.009114
AC009268.1	-0.07495	0.009114
NA	-0.07495	0.009114
TMEM132A	-0.0042	0.009097
HIST1H2BJ	-0.00711	0.009091
RNY3P8	-0.04018	0.009081
AC116345.1	-0.08298	0.009045
MIRLET7F1	-0.07625	0.009045
AF201337.1	0.020716	0.009042
AC012615.3	-0.00558	0.00895
GRIA3	-0.00433	0.008924
KRTAP5-10	-0.05108	0.008883
ZSCAN2	-0.00443	0.008879
VCX3B	-0.05379	0.008878
CENPK	0.00984	0.008877
AC006539.2	-0.02217	0.008829
AL121769.1	0.079091	0.008801
RPSAP3	0.079091	0.008801
ZNF532	0.002689	0.008797
LEFTY1	-0.05032	0.008796
AP5Z1	-0.00393	0.008793
FOXJ2	-0.00564	0.008774
DIMT1	-0.00338	0.008772
ZNF234	0.003903	0.008761
PLCL2	0.00675	0.008746
NT5C3A	0.003542	0.008734
CCDC144CP	-0.01638	0.008723
LNCOC1	-0.02117	0.008723
ARHGEF1	-0.00276	0.008719
NRK	0.070176	0.008719
Z97198.1	0.070176	0.008719
AC010975.1	0.070176	0.008719
AC079866.2	0.070176	0.008719
AC079061.1	-0.04778	0.008718
NA	-0.07326	0.008683
MALINC1	0.019153	0.008659
MAPK8IP3	-0.00362	0.008634
EIF3H	0.002799	0.008627
COG4	0.002782	0.008612
BDKRB1	-0.007	0.008608
NA	-0.06523	0.0086
NINL	0.003135	0.008594
NA	-0.00308	0.008577
FAHD2CP	-0.0075	0.008575
CDRT1	-0.06583	0.008564

NAPA	0.003339	0.008564
ABHD5	0.004989	0.008548
TTN	0.003272	0.008537
DNAJB4	0.004263	0.008534
AC064807.1	-0.01104	0.008527
FERP1	0.006066	0.008525
AC012085.1	0.020083	0.008516
ZNF441	-0.00717	0.008506
AKT1S1	-0.00291	0.008444
MT-TA	-0.0208	0.008436
FRAT1	-0.00668	0.008399
RNA5SP284	-0.02554	0.008396
LRRC37A15P	-0.0242	0.008367
AC006001.3	0.006133	0.008327
LINC02600	-0.01399	0.008317
PRRT1	-0.00535	0.008309
TNFRSF10B	-0.00258	0.008288
AP000941.1	-0.0189	0.008287
JMJD1C-AS1	0.008029	0.008277
RAD21	-0.00256	0.008271
SLC25A12	-0.0059	0.008255
NEU3	0.005619	0.008227
SDCCAG8	-0.00391	0.008222
LDHAP2	0.028016	0.00819
C1orf61	-0.00458	0.008163
AC104116.1	-0.00536	0.008158
ADAP1	0.007158	0.008155
AC092634.5	0.05215	0.008154
SLC37A2	-0.0329	0.008147
ATP2C2	0.025896	0.008129
CD24P4	-0.00365	0.0081
SMG9	-0.00346	0.008096
MIER3	-0.00301	0.008042
MIR191	-0.07374	0.008018
SNORD11B	-0.07374	0.008018
HSPD1P6	0.02694	0.008008
NA	-0.01893	0.007994
ELP2	0.002493	0.007985
HNRNPKP2	0.024393	0.007967
CADPS2	0.046813	0.007957
AC090948.1	0.025626	0.007954
BIVM	0.00357	0.007953
AKNAD1	-0.02197	0.007945
TMEM97	0.002794	0.007931
LRRC37A2	0.00661	0.007925
OTUB1	-0.00259	0.007914
CACNG7	-0.0039	0.007903
USP37	-0.00351	0.007901
AC234781.5	0.032522	0.0079
ZNF714	-0.00372	0.007868

PUM3	0.003128	0.007843
CMTR1	0.002998	0.007842
NA	0.019645	0.007818
INTS6L-AS1	0.044933	0.0078
NA	0.004353	0.007775
AC009127.1	0.069857	0.007775
AL358334.2	-0.01744	0.007746
AC092335.1	-0.00604	0.007726
RTKN	-0.00373	0.007725
AFG1L	-0.00659	0.00769
CIART	0.007353	0.00768
NA	0.025431	0.007672
AC096540.1	0.030044	0.007666
RN7SL809P	-0.03835	0.007658
LINC01772	-0.00618	0.007652
ZNF160	-0.00345	0.007646
SOX10	-0.03294	0.007637
MYCBPAP	-0.01045	0.007633
NA	0.056788	0.007615
MTO1	0.004033	0.007611
LRRC8D	0.00384	0.007601
AP1AR	0.003223	0.007585
PHF21B	0.002759	0.007583
KCNH6	0.010404	0.007562
KNTC1	0.002846	0.007553
AL731577.2	0.015339	0.007518
ZNF57	0.007498	0.007516
SNORA38	-0.08141	0.007507
MIR26A2	-0.08141	0.007507
BMS1P11	-0.08141	0.007507
DYNLT3P2	-0.08141	0.007507
CICP7	-0.08141	0.007507
MIR5189	-0.08141	0.007507
MIR4492	-0.08141	0.007507
MIR3143	-0.08141	0.007507
NA	-0.08141	0.007507
NA	-0.08141	0.007507
CDH1	-0.06782	0.007499
AOC3	-0.02981	0.007498
NA	0.004939	0.007475
LDHAP5	0.072945	0.007475
RPL37P6	0.072945	0.007475
ALDH1L1-AS1	0.072945	0.007475
PILRA	-0.01616	0.007431
AC114491.1	-0.02505	0.007425
ZNF788P	-0.01258	0.007414
NA	0.002285	0.007413
NA	0.078581	0.007411
RF00019	0.078581	0.007411
NA	0.078581	0.007411

HIST2H3C	0.078581	0.007411
MIR490	0.078581	0.007411
AC092681.1	0.078581	0.007411
MTATP6P19	0.078581	0.007411
AC007182.2	0.078581	0.007411
RPL23AP94	0.078581	0.007411
AC113423.1	0.078581	0.007411
AC116903.2	0.078581	0.007411
AC034229.4	0.078581	0.007411
AC022748.2	0.018891	0.00741
RF00019	-0.02662	0.007406
ZNF337	0.002861	0.007391
ZKSCAN5	-0.0028	0.007389
AC010729.2	0.013209	0.007336
PCNP	0.002315	0.007324
CLK3P2	-0.07308	0.007317
AC004083.1	-0.07308	0.007317
RNPEP	-0.00318	0.007312
SCAMP1-AS1	0.005868	0.007307
AC098617.1	0.057952	0.007276
SEPT5	0.00368	0.007264
HSPD1P5	0.045939	0.007262
FEM1C	-0.00424	0.007252
AC012309.2	0.010659	0.00724
EPOR	0.004043	0.007237
GOLGA8Q	0.03794	0.007208
NA	-0.06487	0.007202
AC245014.1	0.049539	0.007198
KCNH2	-0.00276	0.007165
PHOSPHO2	-0.00854	0.007161
CADM3	0.006038	0.00716
NA	-0.03899	0.007146
TECRP1	-0.03899	0.007146
SHOC2	-0.00346	0.007146
CD247	0.032913	0.007144
AC100793.3	-0.00698	0.007134
Z69706.1	0.024643	0.007106
MAP4K3	-0.0029	0.0071
UCN	0.008008	0.007066
UQCC3	0.003031	0.007057
JAKMIP2	0.004224	0.007046
AC023886.1	0.027393	0.007041
PRPF31	-0.00234	0.00703
AL049697.1	-0.015	0.007021
AC072061.1	0.012048	0.006999
NA	-0.02496	0.006983
AL080250.1	-0.01926	0.006973
INO80D	0.003206	0.006957
DAZL	-0.02472	0.00695
TTC19	-0.00257	0.006949

HECW2-AS1	0.037567	0.00694
GDPGP1	-0.00539	0.006939
GIGYF1	0.002336	0.006927
AC021739.5	0.027505	0.006908
AC124312.3	-0.01116	0.006873
CFAP45	0.017814	0.006864
AC012555.1	-0.04257	0.006854
ABCG1	-0.00924	0.006829
AC139495.1	0.017692	0.006826
PHYKPL	0.002448	0.006818
AC018693.1	-0.00895	0.006811
MAP2K2	0.00285	0.006791
TTC23L	0.01766	0.006781
AC146944.1	-0.07026	0.006779
C9orf92	-0.07026	0.006779
AC120349.1	-0.07026	0.006779
RIC8A	0.001994	0.006719
HNRNPH2	-0.0023	0.006709
NA	-0.0041	0.006702
WDR46	-0.00197	0.006702
AC010531.3	-0.05076	0.006699
LINC00240	-0.01165	0.006677
AC011944.1	-0.0723	0.006651
SDR42E2	-0.0723	0.006651
NA	-0.0723	0.006651
AC013270.1	-0.0723	0.006651
AC079296.1	-0.0723	0.006651
AC069503.2	-0.0723	0.006651
AL049872.1	-0.0723	0.006651
NA	-0.0723	0.006651
NA	-0.0723	0.006651
BCAP31	0.002034	0.00665
USP1	0.002559	0.00665
HNRNPA1P49	-0.01846	0.006645
GOLGA2P8	-0.03126	0.006607
NA	-0.02658	0.006603
GM2A	-0.00314	0.006599
AMT	0.002665	0.006586
PPIAP54	-0.03612	0.006565
LIFR-AS1	0.012667	0.006563
ADAMTS8	0.06947	0.006554
YBX1P4	0.06947	0.006554
GNAQP1	0.06947	0.006554
NA	0.06947	0.006554
NA	0.06947	0.006554
RNU6-759P	0.06947	0.006554
WNT10A	-0.00819	0.006528
RBM45	0.003617	0.00647
MYO1B	0.002055	0.006452
NA	0.003879	0.006436

INPP5E	-0.00397	0.006432
AL355990.1	0.030499	0.006416
AC007099.1	-0.04847	0.0064
AC011921.3	-0.04847	0.0064
ANKHD1	-0.00725	0.006399
ROCK2	0.00229	0.006369
NA	0.034444	0.006362
AC093788.1	0.010581	0.006356
VPS11	-0.00282	0.006322
ANGEL1	-0.0029	0.006277
C11orf1	0.004251	0.006271
WDFY3	-0.00294	0.006268
FAM207BP	-0.04231	0.006251
NA	0.039845	0.006245
ARID3A	0.003471	0.006237
RF00019	-0.03508	0.00621
UBFD1	0.00178	0.006176
HELZ	-0.00208	0.006169
EDEM1	-0.00253	0.006121
ASS1P12	-0.01257	0.006115
ZNF362	0.003036	0.00611
SFXN3	0.00284	0.006092
MICU2	-0.00283	0.006089
RPL23AP20	0.01462	0.006078
CCSER1	0.008334	0.006028
PTMAP4	-0.01416	0.006027
ZNF652	0.002379	0.006014
CKS1B	0.00206	0.005976
ORAI3	-0.00623	0.005951
AL022316.1	0.040597	0.005937
FAM167A-AS1	-0.01116	0.005932
GLMN	-0.00338	0.005911
AC234782.3	0.01658	0.005909
UBE2G2	-0.00188	0.005902
PIGG	-0.00215	0.005894
AC010422.2	-0.02396	0.005892
PPIL3	-0.00296	0.005874
MTX1P1	-0.00549	0.005843
DKFZP434H168	-0.00812	0.005808
RND2	0.003195	0.005796
LINC01978	0.044528	0.005793
AP002956.1	-0.00319	0.005777
KLHL35	0.009746	0.005762
NA	-0.01324	0.005723
AL162385.1	0.020562	0.005713
MXRA8	-0.00689	0.005705
SNTA1	0.004363	0.005697
AFF4	-0.00174	0.005681
MSANTD3	0.002401	0.005675
UBE3D	-0.00387	0.005653

SWAP70	0.002846	0.005635
HSPE1P27	0.042083	0.005633
MRPL41	0.002276	0.005589
ANO7	-0.00715	0.005587
TAC4	0.040139	0.00558
AC138512.1	-0.04204	0.005578
COL26A1	-0.02885	0.005575
NA	0.007395	0.005559
GRAP2	0.03059	0.005546
AC005726.5	-0.00636	0.005531
AC012615.6	0.005389	0.005526
NFE2L2	-0.00209	0.005505
AC010904.2	0.019411	0.005473
SLC5A6	0.00168	0.00545
DENND4B	0.002048	0.005429
NA	0.003461	0.005427
PLAUR	0.003545	0.005424
NA	0.041644	0.005417
SRRM4	-0.00365	0.005408
U52111.1	0.012724	0.005401
RAMP2-AS1	0.004674	0.005371
CREBZF	-0.00151	0.005305
NA	-0.02475	0.005303
AC004948.1	-0.03139	0.005293
JCAD	-0.00589	0.005267
GPX4	0.001736	0.005259
SERPINC1	0.035969	0.005244
SHQ1P1	-0.01	0.005219
NUP35	0.00272	0.005214
ZNF175	0.002571	0.005194
ARHGEF4	-0.00307	0.005172
AP001816.1	-0.00533	0.005161
HNRNPD	-0.00134	0.005137
AC135507.1	-0.00281	0.005134
ICAM2	-0.01061	0.005119
AC135279.1	-0.01441	0.00511
PLCB1	0.002702	0.005103
Z99496.1	0.030157	0.005089
AC004803.1	0.005052	0.00508
AL159174.1	0.018227	0.005063
COIL	0.002283	0.005054
LRPPRC	-0.00156	0.005053
TROAP	-0.0023	0.005
UBE2C	0.001932	0.004993
LAPTM4B	0.001437	0.004992
NA	-0.00242	0.004985
RNF168	-0.00214	0.004955
MT-ATP8	0.002282	0.0049
GPSM2	0.001778	0.004891
FAM117B	0.0021	0.004869

SRP9	0.001768	0.004831
TOR1B	0.001596	0.004824
GYPE	0.02876	0.004807
TRAPPC2	-0.00232	0.004788
GSTM2	0.002962	0.004778
LINC02607	0.01799	0.004756
PRKAG2	0.002403	0.004746
SATL1	0.004555	0.004732
NA	0.002385	0.004725
PHF20	-0.00186	0.004712
ZFAT	-0.00289	0.004704
MRPS27	-0.00156	0.004697
MANSC1	0.002223	0.004676
MYC	0.001819	0.004655
PHF14	0.001882	0.00465
TMEM199	0.001934	0.004646
AL049569.1	0.006447	0.004643
AL391834.1	-0.00499	0.004604
RAB2A	0.001588	0.004577
OLFML1	0.028679	0.004568
RNA5SP174	-0.01422	0.004545
FAM87A	-0.02696	0.004532
AC017002.3	0.029971	0.004493
AC108174.1	0.029971	0.004493
SPATA6	0.003459	0.004483
RNU6-945P	-0.02938	0.004479
AC027237.1	-0.00204	0.004479
CHL1	-0.01592	0.004442
NPM3	0.001786	0.004421
DTD2	0.002369	0.004411
EEF1A1P4	-0.02246	0.004399
SMPD5	-0.00873	0.00439
ZNF688	-0.00277	0.004377
AL583832.1	0.01106	0.004368
AL137802.2	-0.00888	0.004328
EPM2AIP1	0.001587	0.004322
TMEM33	-0.00156	0.004321
CFL1P5	-0.0236	0.004315
RFFL	0.002107	0.0043
PGGHG	-0.00341	0.004292
SAP18	-0.00169	0.004286
AC004552.1	0.024933	0.004282
COQ8A	0.001523	0.00428
HIRIP3	-0.0017	0.004275
BMS1P1	0.006789	0.004268
RNF152	-0.00146	0.004258
MGAT4C	-0.04062	0.004258
RPL7AP4	-0.04062	0.004258
CNOT6LP1	-0.04062	0.004258
BAG6	-0.00154	0.004248

NA	0.014785	0.004245
EIF1	-0.00117	0.004231
CAND2	-0.0031	0.004228
MATN1-AS1	-0.01357	0.004219
NA	0.001596	0.004183
AL049840.5	0.009902	0.00413
AC020913.2	0.00476	0.004129
MRPL19	-0.00171	0.004128
CHPT1	0.002937	0.004074
LRP1	-0.00209	0.004056
NA	0.001507	0.004042
EEF1A1P12	0.012812	0.004029
AC008870.2	-0.00649	0.004017
LINC02118	-0.01065	0.004002
DHX8	0.001187	0.003977
EWSR1	0.001033	0.003975
AL359091.3	0.003762	0.003957
AL035587.1	-0.00369	0.003949
SPAST	-0.00144	0.003889
AL021707.2	-0.00301	0.003887
MTATP6P27	0.017399	0.003876
PSMA3	0.001553	0.003864
SCFD2	-0.00221	0.00386
NA	-0.00256	0.003854
QRSL1	-0.0018	0.003852
AC026979.3	0.007019	0.003821
AC010203.1	-0.0077	0.003807
C4orf48	-0.00191	0.003779
SRP14P2	0.027054	0.003776
AC068522.1	0.027054	0.003776
CRYBG3	0.002179	0.003736
DUSP9	-0.01422	0.003735
RASGRF2	0.003376	0.003714
LEPROT	0.001322	0.003711
ZNF689	-0.00183	0.003689
MIR9-3HG	0.00311	0.003684
CHMP2A	0.001448	0.00367
ADGRG1	0.005685	0.003665
RRN3P2	-0.01771	0.003656
NA	-0.00761	0.003652
LINC01719	-0.00304	0.003645
AC010201.1	0.009874	0.00364
AC011446.2	0.001388	0.003622
LRRC71	-0.01131	0.003605
AP3B1	-0.00134	0.003598
RNU6-8	0.01028	0.003596
DDX55	-0.00122	0.003586
SERPINE2	-0.00477	0.003582
PRDX6	-0.001	0.003573
TEX10	0.00115	0.003571

HMGA1P2	0.009417	0.003502
GOT2	-0.00107	0.003502
EME1	-0.00149	0.0035
MINDY4	0.006208	0.003492
DAB2IP	0.00118	0.003483
NA	0.001073	0.003476
SATB1	0.001293	0.003459
OGFOD1	0.001271	0.003408
KF456478.1	0.00218	0.003401
LMOD1	-0.00335	0.003383
DIAPH3-AS1	-0.0058	0.00338
ANKRD50	0.00141	0.003373
RF00019	0.029848	0.003332
HSD11B1-AS1	0.029848	0.003332
AL512604.2	0.029848	0.003332
NA	0.029848	0.003332
MIR3149	0.029848	0.003332
U2AF1L4	0.004461	0.003323
NA	-0.01496	0.003314
CAMKMT	0.002394	0.00331
AF274858.1	0.007712	0.003306
AC084117.1	0.015161	0.003305
CLK3	0.001498	0.003299
NUP160	-0.00098	0.003276
C1orf220	0.011607	0.003268
SLC6A7	0.038098	0.003247
SLC7A9	0.038098	0.003247
OTC	0.038098	0.003247
ITIH1	0.038098	0.003247
IPCEF1	0.038098	0.003247
FKBP6	0.038098	0.003247
CASS4	0.038098	0.003247
MEDAG	0.038098	0.003247
DAO	0.038098	0.003247
AC005840.1	0.038098	0.003247
HDGFL1	0.038098	0.003247
DLX2	0.038098	0.003247
LCT	0.038098	0.003247
EPHA4	0.038098	0.003247
PDC	0.038098	0.003247
TNFSF10	0.038098	0.003247
TCP11	0.038098	0.003247
OMD	0.038098	0.003247
AP1M2	0.038098	0.003247
GATA5	0.038098	0.003247
TBC1D8B	0.038098	0.003247
FAM83F	0.038098	0.003247
ADAM30	0.038098	0.003247
EGR4	0.038098	0.003247
SLC19A3	0.038098	0.003247

IL10	0.038098	0.003247
BAAT	0.038098	0.003247
FITM1	0.038098	0.003247
SYPL2	0.038098	0.003247
FNDC7	0.038098	0.003247
CD53	0.038098	0.003247
PKLR	0.038098	0.003247
OLAH	0.038098	0.003247
PART1	0.038098	0.003247
C2CD6	0.038098	0.003247
LYPD5	0.038098	0.003247
TTLL10	0.038098	0.003247
ASB5	0.038098	0.003247
SLC6A18	0.038098	0.003247
MMP10	0.038098	0.003247
TMEM92	0.038098	0.003247
SLC22A11	0.038098	0.003247
VWA3B	0.038098	0.003247
GRM5	0.038098	0.003247
DLGAP1	0.038098	0.003247
SERF1A	0.038098	0.003247
SLC16A11	0.038098	0.003247
P2RY2	0.038098	0.003247
DCAF4L2	0.038098	0.003247
GPR4	0.038098	0.003247
CDKN2AIPNLP1	0.038098	0.003247
OTOS	0.038098	0.003247
MX2	0.038098	0.003247
NA	0.038098	0.003247
FOXD4L4	0.038098	0.003247
MRPL30	0.038098	0.003247
KLHL34	0.038098	0.003247
NAP1L2	0.038098	0.003247
FOXE3	0.038098	0.003247
PNLIPRP1	0.038098	0.003247
AC100800.1	0.038098	0.003247
RTP5	0.038098	0.003247
GOLGA8M	0.038098	0.003247
NA	0.038098	0.003247
ZNF300P1	0.038098	0.003247
NA	0.038098	0.003247
CNGA1	0.038098	0.003247
MIR30E	0.038098	0.003247
MIR425	0.038098	0.003247
MIRLET7A1	0.038098	0.003247
RNU6-858P	0.038098	0.003247
RNU4-82P	0.038098	0.003247
RNU4-39P	0.038098	0.003247
RF00019	0.038098	0.003247
RF00153	0.038098	0.003247

RF00019	0.038098	0.003247
RF00019	0.038098	0.003247
RNU5D-1	0.038098	0.003247
RF00139	0.038098	0.003247
RF00019	0.038098	0.003247
RNU6-799P	0.038098	0.003247
RF00019	0.038098	0.003247
SNORA63D	0.038098	0.003247
RF00019	0.038098	0.003247
RNU1-91P	0.038098	0.003247
RF00019	0.038098	0.003247
RF00019	0.038098	0.003247
NA	0.038098	0.003247
NA	0.038098	0.003247
NA	0.038098	0.003247
SLC44A4	0.038098	0.003247
LINC01854	0.038098	0.003247
C5orf60	0.038098	0.003247
AC010624.1	0.038098	0.003247
FOXD4L5	0.038098	0.003247
RNU6-32P	0.038098	0.003247
RF00019	0.038098	0.003247
SNORD38C	0.038098	0.003247
RNU1-106P	0.038098	0.003247
SNORD116-12	0.038098	0.003247
RNU6-110P	0.038098	0.003247
RNU6-178P	0.038098	0.003247
SNORA19	0.038098	0.003247
MIR215	0.038098	0.003247
MIR626	0.038098	0.003247
MIR9-1	0.038098	0.003247
MIR561	0.038098	0.003247
MIR454	0.038098	0.003247
MIR708	0.038098	0.003247
RF00554	0.038098	0.003247
SNORA74B	0.038098	0.003247
SNORD70	0.038098	0.003247
RNU6-808P	0.038098	0.003247
CNN2P9	0.038098	0.003247
AC242426.1	0.038098	0.003247
PCNPP3	0.038098	0.003247
NAP1L1P3	0.038098	0.003247
HNRNPA1P33	0.038098	0.003247
AC092447.2	0.038098	0.003247
AC104306.1	0.038098	0.003247
DNAJB1P1	0.038098	0.003247
AC007277.1	0.038098	0.003247
AL096701.1	0.038098	0.003247
RAD51AP1P1	0.038098	0.003247
HSPD1P7	0.038098	0.003247

GLULP6	0.038098	0.003247
ASS1P10	0.038098	0.003247
EFCAB8	0.038098	0.003247
MIR941-4	0.038098	0.003247
RPL7P25	0.038098	0.003247
AL355312.1	0.038098	0.003247
POM121L4P	0.038098	0.003247
HIST1H2BPS2	0.038098	0.003247
POM121L14P	0.038098	0.003247
AL353133.1	0.038098	0.003247
NA	0.038098	0.003247
MIR1303	0.038098	0.003247
NA	0.038098	0.003247
RNU2-37P	0.038098	0.003247
RNU2-38P	0.038098	0.003247
RNA5SP39	0.038098	0.003247
NA	0.038098	0.003247
RF00019	0.038098	0.003247
USP17L2	0.038098	0.003247
AL161935.1	0.038098	0.003247
AL365436.1	0.038098	0.003247
HSPA8P16	0.038098	0.003247
AL354877.1	0.038098	0.003247
LINC02636	0.038098	0.003247
LINC00853	0.038098	0.003247
SUCLA2P1	0.038098	0.003247
SMARCE1P1	0.038098	0.003247
OSTCP2	0.038098	0.003247
SP3P	0.038098	0.003247
STIP1P3	0.038098	0.003247
LINC01623	0.038098	0.003247
P3H2-AS1	0.038098	0.003247
AC002075.1	0.038098	0.003247
AL138767.3	0.038098	0.003247
AL158839.1	0.038098	0.003247
LINC02519	0.038098	0.003247
AC013480.1	0.038098	0.003247
GAPDHP32	0.038098	0.003247
AC007365.1	0.038098	0.003247
AC110926.1	0.038098	0.003247
AC096582.1	0.038098	0.003247
AC244021.1	0.038098	0.003247
NA	0.038098	0.003247
AL136528.1	0.038098	0.003247
CROCCP4	0.038098	0.003247
AL590135.1	0.038098	0.003247
LINC01546	0.038098	0.003247
GRK5-IT1	0.038098	0.003247
AC023271.1	0.038098	0.003247
LYPLAL1-AS1	0.038098	0.003247

CICP9	0.038098	0.003247
AC108059.1	0.038098	0.003247
LPGAT1-AS1	0.038098	0.003247
AC079305.3	0.038098	0.003247
AL139421.1	0.038098	0.003247
RPS4XP5	0.038098	0.003247
AL022238.2	0.038098	0.003247
SEPHS1P4	0.038098	0.003247
AC095033.1	0.038098	0.003247
ATP5MC2P3	0.038098	0.003247
COL6A4P1	0.038098	0.003247
SF3A3P1	0.038098	0.003247
GPAA1P2	0.038098	0.003247
AL590093.1	0.038098	0.003247
AL589745.1	0.038098	0.003247
RPL10P3	0.038098	0.003247
SDAD1P2	0.038098	0.003247
LINC00486	0.038098	0.003247
MTCO1P53	0.038098	0.003247
LINC02541	0.038098	0.003247
AL157884.2	0.038098	0.003247
IGKV1OR2-108	0.038098	0.003247
TBL1XR1-AS1	0.038098	0.003247
AC072052.1	0.038098	0.003247
AL353748.1	0.038098	0.003247
F10-AS1	0.038098	0.003247
AC005537.1	0.038098	0.003247
USP12-AS1	0.038098	0.003247
SLC25A6P2	0.038098	0.003247
AC002366.1	0.038098	0.003247
PPIAP70	0.038098	0.003247
AC073323.1	0.038098	0.003247
AC092810.3	0.038098	0.003247
AC099336.1	0.038098	0.003247
RPL34P27	0.038098	0.003247
NDUFB4P10	0.038098	0.003247
PHKA1P1	0.038098	0.003247
AC244453.2	0.038098	0.003247
NA	0.038098	0.003247
MAPRE1P3	0.038098	0.003247
PPIAP32	0.038098	0.003247
EEF1A1P34	0.038098	0.003247
AC017104.3	0.038098	0.003247
PGAM1P11	0.038098	0.003247
IGHD4-4	0.038098	0.003247
DDX39BP1	0.038098	0.003247
LINC01136	0.038098	0.003247
AC022018.1	0.038098	0.003247
LINC01283	0.038098	0.003247
KBTBD13	0.038098	0.003247

AC006960.1	0.038098	0.003247
PTGES3P5	0.038098	0.003247
LINC01800	0.038098	0.003247
NA	0.038098	0.003247
PHBP13	0.038098	0.003247
AL135937.1	0.038098	0.003247
AC104667.2	0.038098	0.003247
AL031667.2	0.038098	0.003247
AC007041.1	0.038098	0.003247
AC012506.4	0.038098	0.003247
OR2AF1P	0.038098	0.003247
BRD7P5	0.038098	0.003247
Z99127.2	0.038098	0.003247
AC004448.2	0.038098	0.003247
AC073410.1	0.038098	0.003247
AC104076.1	0.038098	0.003247
IGHD1-1	0.038098	0.003247
PPIAP64	0.038098	0.003247
Z82214.2	0.038098	0.003247
AL117381.1	0.038098	0.003247
AC092809.3	0.038098	0.003247
AC069213.3	0.038098	0.003247
BAALC-AS2	0.038098	0.003247
RPS26P41	0.038098	0.003247
AC079905.1	0.038098	0.003247
CYP46A4P	0.038098	0.003247
SULT1C2P1	0.038098	0.003247
AL158071.4	0.038098	0.003247
RPSAP11	0.038098	0.003247
AC012668.3	0.038098	0.003247
RPL30P4	0.038098	0.003247
AC234775.2	0.038098	0.003247
PA2G4P1	0.038098	0.003247
AL445218.1	0.038098	0.003247
FLG-AS1	0.038098	0.003247
DDX39BP2	0.038098	0.003247
Z98742.3	0.038098	0.003247
RNU7-63P	0.038098	0.003247
RNU7-134P	0.038098	0.003247
RF01225	0.038098	0.003247
NA	0.038098	0.003247
NA	0.038098	0.003247
AL354989.2	0.038098	0.003247
CADM2-AS1	0.038098	0.003247
RN7SL513P	0.038098	0.003247
RNF7P1	0.038098	0.003247
AL122013.1	0.038098	0.003247
AC022494.1	0.038098	0.003247
AL031428.1	0.038098	0.003247
KRTAP5-8	0.038098	0.003247

PMS2P11	0.038098	0.003247
RPL7P19	0.038098	0.003247
ARHGAP8	0.038098	0.003247
AC109466.1	0.038098	0.003247
RN7SL124P	0.038098	0.003247
AC083904.1	0.038098	0.003247
RN7SL329P	0.038098	0.003247
NA	0.038098	0.003247
RN7SL516P	0.038098	0.003247
RN7SL846P	0.038098	0.003247
NA	0.038098	0.003247
NA	0.038098	0.003247
RN7SL65P	0.038098	0.003247
AC090589.1	0.038098	0.003247
PARAL1	0.038098	0.003247
RN7SL833P	0.038098	0.003247
AC079193.1	0.038098	0.003247
LINC02016	0.038098	0.003247
RPS4XP10	0.038098	0.003247
RN7SL698P	0.038098	0.003247
RPS3AP35	0.038098	0.003247
MIR4300HG	0.038098	0.003247
AP004609.1	0.038098	0.003247
PCED1B-AS1	0.038098	0.003247
AC108142.1	0.038098	0.003247
KRT8P48	0.038098	0.003247
AC018781.1	0.038098	0.003247
AC022447.2	0.038098	0.003247
AC008883.1	0.038098	0.003247
SNX18P25	0.038098	0.003247
HMGN1P13	0.038098	0.003247
AC025458.1	0.038098	0.003247
AC005920.2	0.038098	0.003247
AC011379.1	0.038098	0.003247
LINC02200	0.038098	0.003247
CCT5P1	0.038098	0.003247
INTS6P1	0.038098	0.003247
ROPN1L-AS1	0.038098	0.003247
REELD1	0.038098	0.003247
LINC01262	0.038098	0.003247
AC136632.2	0.038098	0.003247
TUBB7P	0.038098	0.003247
AC104123.1	0.038098	0.003247
AC021106.2	0.038098	0.003247
AC005324.3	0.038098	0.003247
LINC01267	0.038098	0.003247
TET2-AS1	0.038098	0.003247
FAM160A1-DT	0.038098	0.003247
AC005920.3	0.038098	0.003247
NA	0.038098	0.003247

RNU7-200P	0.038098	0.003247
RN7SKP276	0.038098	0.003247
RNU6-964P	0.038098	0.003247
RNU6-107P	0.038098	0.003247
RNA5SP309	0.038098	0.003247
RNU6-135P	0.038098	0.003247
RNU6-388P	0.038098	0.003247
NA	0.038098	0.003247
MIR2355	0.038098	0.003247
RN7SKP57	0.038098	0.003247
RNA5SP437	0.038098	0.003247
RNU7-161P	0.038098	0.003247
AC117834.1	0.038098	0.003247
AC024568.1	0.038098	0.003247
AC104964.2	0.038098	0.003247
AC105118.1	0.038098	0.003247
LINC00051	0.038098	0.003247
AC100797.1	0.038098	0.003247
AC103760.1	0.038098	0.003247
AC023632.5	0.038098	0.003247
AC113143.1	0.038098	0.003247
PTP4A2P2	0.038098	0.003247
ARMS2	0.038098	0.003247
MTATP6P15	0.038098	0.003247
AP000785.1	0.038098	0.003247
AP003501.2	0.038098	0.003247
OR7E2P	0.038098	0.003247
AC093510.1	0.038098	0.003247
PDCD5P1	0.038098	0.003247
AC069234.1	0.038098	0.003247
PSMC1P9	0.038098	0.003247
AC007688.2	0.038098	0.003247
TAS2R30	0.038098	0.003247
AC023512.1	0.038098	0.003247
NA	0.038098	0.003247
AC012150.1	0.038098	0.003247
GSTP1P1	0.038098	0.003247
AC025154.2	0.038098	0.003247
METTL7AP1	0.038098	0.003247
AC004217.2	0.038098	0.003247
AC089983.1	0.038098	0.003247
AC008125.1	0.038098	0.003247
AC026111.1	0.038098	0.003247
AC073569.1	0.038098	0.003247
AC063950.1	0.038098	0.003247
AC130895.1	0.038098	0.003247
AL442663.2	0.038098	0.003247
SPESP1	0.038098	0.003247
LINC02322	0.038098	0.003247
AL137230.1	0.038098	0.003247

NA	0.038098	0.003247
GTF3AP2	0.038098	0.003247
AL161804.1	0.038098	0.003247
FOXN3-AS2	0.038098	0.003247
DDX18P1	0.038098	0.003247
AC012568.1	0.038098	0.003247
AC019254.2	0.038098	0.003247
AC026770.1	0.038098	0.003247
NDUFAF4P1	0.038098	0.003247
AC126773.1	0.038098	0.003247
AC023825.2	0.038098	0.003247
AC111152.3	0.038098	0.003247
AC009102.1	0.038098	0.003247
DNM1P34	0.038098	0.003247
AC073476.2	0.038098	0.003247
KRT8P50	0.038098	0.003247
AL160286.2	0.038098	0.003247
LINC02516	0.038098	0.003247
AC091167.2	0.038098	0.003247
GOLGA8T	0.038098	0.003247
AC007728.2	0.038098	0.003247
AC073314.1	0.038098	0.003247
AC027796.1	0.038098	0.003247
AC130650.1	0.038098	0.003247
MRPS21P9	0.038098	0.003247
AC027796.5	0.038098	0.003247
MIR5699	0.038098	0.003247
AC017100.2	0.038098	0.003247
MIR5685	0.038098	0.003247
AC103808.1	0.038098	0.003247
RN7SL15P	0.038098	0.003247
NA	0.038098	0.003247
MIR3131	0.038098	0.003247
NA	0.038098	0.003247
NA	0.038098	0.003247
AC104984.2	0.038098	0.003247
RN7SL279P	0.038098	0.003247
MIR4514	0.038098	0.003247
MIR4489	0.038098	0.003247
RHOT1P1	0.038098	0.003247
MIR4323	0.038098	0.003247
UBL5P2	0.038098	0.003247
MIR3657	0.038098	0.003247
RDM1P2	0.038098	0.003247
AC005703.4	0.038098	0.003247
AC044840.1	0.038098	0.003247
AC005224.3	0.038098	0.003247
NA	0.038098	0.003247
AC027514.1	0.038098	0.003247
AC092296.1	0.038098	0.003247

CTBP2P7	0.038098	0.003247
AC025048.1	0.038098	0.003247
AC003070.1	0.038098	0.003247
AP002439.1	0.038098	0.003247
AC005180.2	0.038098	0.003247
AC006116.9	0.038098	0.003247
AP005131.5	0.038098	0.003247
AC048380.1	0.038098	0.003247
AC022726.1	0.038098	0.003247
NA	0.038098	0.003247
AC008395.1	0.038098	0.003247
NA	0.038098	0.003247
LRRC2-AS1	0.038098	0.003247
NA	0.038098	0.003247
CDC42EP3P1	0.038098	0.003247
AC010319.3	0.038098	0.003247
CALR3	0.038098	0.003247
AC011466.2	0.038098	0.003247
NA	0.038098	0.003247
NA	0.038098	0.003247
MAP2K4P1	0.038098	0.003247
MEI4	0.038098	0.003247
AP000769.2	0.038098	0.003247
AC087283.1	0.038098	0.003247
AC011458.1	0.038098	0.003247
AC007029.1	0.038098	0.003247
AC093107.2	0.038098	0.003247
AC108463.3	0.038098	0.003247
NA	0.038098	0.003247
AC010857.1	0.038098	0.003247
AC016745.2	0.038098	0.003247
AL133245.1	0.038098	0.003247
AC007684.1	0.038098	0.003247
AL021707.8	0.038098	0.003247
AL162591.2	0.038098	0.003247
BX323046.1	0.038098	0.003247
NA	0.038098	0.003247
AC090912.3	0.038098	0.003247
AC133644.2	0.038098	0.003247
AC105265.3	0.038098	0.003247
NEDD8	0.001226	0.003216
AC097658.1	0.008375	0.003211
NDUFB5	-0.00117	0.003178
MYADML2	-0.0093	0.003166
RIOX2	-0.0013	0.003162
AC011466.3	-0.00217	0.003114
PRKN	-0.00465	0.003101
ITGAE	0.001488	0.003097
CENPF	0.001303	0.003095
CACNA2D4	-0.02376	0.003095

SNORA73B	-0.00649	0.003085
DCLRE1C	0.001636	0.003074
FER1L5	0.012104	0.003071
MT-ND1	0.000759	0.003069
MCTP2	0.004637	0.003059
FUZ	0.001224	0.003055
TYRO3	-0.00159	0.003043
AC018638.4	-0.00249	0.003036
MIR4292	-0.00514	0.00303
AL360268.1	0.011197	0.003029
HNRNPA1P27	-0.02332	0.002996
MAPRE1P1	-0.02332	0.002996
SLC8A1-AS1	-0.00339	0.002936
PCNX2	0.001367	0.002927
PLPP5	-0.00118	0.002925
AL022328.3	-0.00943	0.002903
NA	-0.02377	0.002899
AC092296.2	-0.02377	0.002899
NA	-0.0111	0.002895
NA	-0.01757	0.002894
ELOBP1	0.027279	0.002884
AC093799.1	0.027279	0.002884
APOBR	0.025744	0.002874
GPR89P	0.025744	0.002874
AC009531.1	0.025744	0.002874
AC025871.2	0.025744	0.002874
AP003354.1	-0.01122	0.002872
DNAJC4	-0.00164	0.002868
CYTL1	0.002442	0.002861
CHST6	0.010804	0.002857
MAU2	0.001142	0.002847
LGALS8	0.000975	0.002841
ECT2L	-0.00718	0.002814
ZFYVE28	0.002859	0.002763
PDE6D	-0.00129	0.002715
NA	-0.00998	0.002658
AC018552.2	0.015662	0.002655
RNU6-26P	-0.01677	0.00265
SLCO1C1	-0.0189	0.002624
FRYL	-0.00109	0.002605
ATG4D	0.00132	0.00258
LEPROTL1	0.000913	0.002563
RPL7AP2	-0.01612	0.00255
AL133481.1	0.004491	0.002548
NA	0.00771	0.002547
STK31	-0.00972	0.002545
EPHX4	0.010056	0.002528
RAD54B	0.00205	0.002525
TAPT1-AS1	0.002313	0.002514
STEAP3-AS1	0.002486	0.00251

MAPK10	-0.00185	0.002509
PKIB	0.015056	0.002507
VKORC1	0.002168	0.002487
PRIM2	-0.00121	0.002483
ZNF324B	0.001538	0.002465
TMEM25	-0.00142	0.00245
AC008738.1	-0.01534	0.00245
GALNT14	-0.00189	0.002426
HINT3	-0.00112	0.002421
BNIP3P1	0.012046	0.002421
KLHL2P1	-0.01119	0.00239
MTPAP	0.001018	0.002389
VPS45	0.000742	0.002381
KLHL11	-0.00191	0.00238
MVB12A	-0.00088	0.002371
CACNG1	0.007905	0.002369
DRAIC	-0.01814	0.002358
HIGD1AP1	-0.01814	0.002358
LINC00928	-0.00423	0.002351
FBXL6	0.00108	0.002326
PITPNM2	-0.0021	0.002325
INTS10	-0.00093	0.002279
PFN1	-0.00063	0.002257
NA	0.002655	0.002254
GRK6	-0.0008	0.002209
ATP9A	0.001181	0.002187
AC007292.3	0.003165	0.002165
POC1B	0.001328	0.002145
ZNF662	0.001543	0.002131
PDE4DIP	0.000871	0.002126
GGT1	-0.00296	0.002121
RECQL	-0.00074	0.002113
FAM229A	-0.00113	0.002083
SLC16A1	-0.00077	0.002078
AQR	0.000721	0.002077
AC048337.1	-0.0095	0.002058
AL033397.2	-0.00224	0.002052
LINC01762	-0.00876	0.002043
AL136172.1	-0.00372	0.002036
KMT5C	0.001194	0.002026
TSG101	-0.00067	0.002009
PSENEN	-0.00128	0.001978
AC091133.1	0.000845	0.001964
BMS1P9	0.009996	0.001955
NA	0.007887	0.001944
OCA2	0.001872	0.00193
AC135586.1	0.00856	0.001916
SETD9	0.001013	0.001913
NUDT3	0.000619	0.001884
PITPNM3	0.003024	0.001871

VAV2	-0.00078	0.001861
Z83844.2	0.001557	0.001858
ADAMTS7	0.000706	0.001847
ZNF890P	-0.00967	0.001809
AC131235.2	-0.00373	0.001763
NAA16	0.000732	0.001762
NA	-0.00763	0.001674
GLI4	0.001056	0.001672
AC005912.1	-0.00133	0.001648
SCAF11	0.000528	0.001631
AC012464.2	-0.00717	0.00162
PTER	0.004547	0.001617
LINC00539	-0.00693	0.00161
MIB2	0.000861	0.001603
LTV1	0.000673	0.0016
ABCC1	0.000802	0.001585
PWP1	0.000587	0.001576
AC092634.3	-0.0034	0.001568
SNCAIP	-0.00606	0.001564
ZNF311	-0.00959	0.001518
TLR1	-0.00284	0.001511
STEAP3	0.000691	0.001508
XAF1	0.004379	0.001489
PDZRN3-AS1	0.006249	0.001479
PAAF1	-0.00071	0.001453
BORA	-0.00085	0.001437
CCT6A	0.000488	0.001405
AC018462.1	0.002584	0.001394
NKD1	0.001641	0.001385
B4GALT2	0.000478	0.001368
ZNF221	-0.00119	0.001336
PPP1R16A	-0.00054	0.001329
SEPT10	0.00062	0.00131
NA	-0.00191	0.001307
ARMC3	-0.00354	0.001299
MROH7	-0.00396	0.001279
FOXO4	0.000698	0.001251
CFAP221	0.00409	0.001222
ZSWIM3	0.000886	0.00121
RHD	0.002966	0.001201
MAML1	-0.00047	0.001189
SPSB2	0.000805	0.001183
MTND5P28	0.005881	0.001162
PWWP3A	0.000387	0.001153
NFYC	0.000431	0.001149
AC092070.2	-0.00496	0.001143
ZNF853	-0.00039	0.001137
AL109811.1	-0.00057	0.001117
ZNF417	0.000727	0.001085
NA	-0.00062	0.00108

PDS5B	0.000351	0.001008
KIF7	0.000571	0.001003
NA	0.006181	0.001002
SMIM7	0.000408	0.000995
RN7SKP173	-0.00331	0.000983
STAU1	-0.00029	0.000971
SCARNA8	0.004429	0.000967
AC008946.1	-0.00269	0.000961
AC010491.1	0.001606	0.00096
NDUFA10	0.00032	0.000959
SLC17A4	-0.00639	0.000946
SUSD2	0.003052	0.000944
AL391650.1	0.000564	0.000939
AC139099.1	0.004033	0.000933
MZT1	-0.00041	0.000932
CTNND1	-0.00029	0.000919
SP2-AS1	0.00078	0.000878
UBXN8	0.000431	0.000862
NA	0.003007	0.00086
CLVS2	-0.00092	0.000809
NA	0.001502	0.000807
PCBP2	0.000248	0.000791
MIEF2	0.000548	0.000776
AC010132.2	0.00122	0.000773
LINC00893	0.001075	0.000708
MKRN2OS	0.001655	0.000706
TBL1XR1	0.000236	0.000691
AC016734.1	-0.00247	0.000645
BLMH	0.000218	0.000642
MMP24OS	-0.00029	0.00064
HMGB1P1	-0.00168	0.000637
ST6GALNAC4	0.000346	0.000618
OR1J1	0.005844	0.000587
CBX3P1	0.005844	0.000587
RNU6-1157P	0.005844	0.000587
AC089998.1	0.005844	0.000587
AC099811.4	0.005844	0.000587
EVL	-0.00025	0.000584
HIST3H2BB	0.001337	0.000583
MFSD12	0.000208	0.000564
KMT2C	-0.00022	0.000562
FAM98C	-0.00035	0.000561
GSTK1	-0.0002	0.000561
AL355312.4	-0.00109	0.000543
TMOD2	-0.0002	0.000528
FANK1	0.000822	0.000525
TAF5	-0.00021	0.000516
NOMO2	0.000254	0.000502
KLF3-AS1	-0.00054	0.00049
AL117336.1	-0.00234	0.000477

MIRLET7G	-0.00275	0.000464
TCEA1	-0.00016	0.000462
MT1P3	0.002135	0.000421
CDCP1	0.001952	0.000414
AC104843.1	0.003937	0.000405
AC005225.3	0.003937	0.000405
HMGA1P3	0.002828	0.000385
AC055733.3	-0.00268	0.000372
MDFI	0.000167	0.000337
MAMLD1	0.00042	0.000313
AC093155.2	-0.00297	0.000299
TMED4	-0.000086	0.000294
MOGAT2	0.00269	0.000292
HS6ST3	0.00269	0.000292
AC022215.1	0.00269	0.000292
RF00092	0.00269	0.000292
PPIAP22	-0.00013	0.000288
AC005306.1	0.000177	0.000265
SLC35D3	0.000278	0.000259
AC006557.1	-0.00075	0.000251
AC091153.3	0.001187	0.000242
ODAM	0.000337	0.000238
P2RY14	0.001347	0.000234
MED13L	-0.00007	0.000194
AL096677.1	0.000732	0.000176
AC015674.1	-0.000055	0.000162
AC105206.2	-0.00018	0.000154
ADAMTSL4	0.000181	0.00014
DDX3X	0.0000401	0.000129
IQSEC3P1	-0.00038	0.000119
RF00012	-0.00089	0.000116
EBLN2	0.000149	0.000101
DDX11L9	0.000262	0.0000848
NA	-0.00035	0.0000536
AC020728.1	-0.0003	0.000031
JHY	0.00001	0.00000967
